# Anti-Cancer Potential of Homemade Fresh Garlic Extract Is Related to Increased Endoplasmic Reticulum Stress

**DOI:** 10.3390/nu10040450

**Published:** 2018-04-05

**Authors:** Voin Petrovic, Anala Nepal, Camilla Olaisen, Siri Bachke, Jonathan Hira, Caroline K. Søgaard, Lisa M. Røst, Kristine Misund, Trygve Andreassen, Torun M. Melø, Zdenka Bartsova, Per Bruheim, Marit Otterlei

**Affiliations:** 1 Department of Clinical and Molecular Medicine, Faculty of Medicine and Health Sciences, NTNU Norwegian University of Science and Technology, N-7491 Trondheim, Norway; onagrus@yahoo.com (V.P.); Anala.Nepal@ntnu.no (A.N.); camilla.olaisen@ntnu.no (C.O.); Siri.Bachke@ntnu.no (S.B.); jonathanhira@gmail.com (J.H.); caroline.d.sogaard@ntnu.no (C.K.S.); kristine.misund@ntnu.no (K.M.); 2 Department of Biotechnology and Food Science, Faculty of Natural Sciences, NTNU Norwegian University of Science and Technology, N-7491 Trondheim, Norway; lisa.m.rost@ntnu.no (L.M.R.); torun.m.melo@ntnu.no (T.M.M.); zdenka.bartsova@ntnu.no (Z.B.); per.bruheim@ntnu.no (P.B.); 3 MR core facility, Department of Circulation and Medical Imaging, Faculty of Medicine and Health Sciences, NTNU Norwegian University of Science and Technology, N-7491 Trondheim, Norway; trygve.andreassen@ntnu.no

**Keywords:** apoptosis, ER stress, allicin, Organo Sulfur Compounds (OSCs), kinome, cancer

## Abstract

The use of garlic and garlic-based extracts has been linked to decreased incidence of cancer in epidemiological studies. Here we examine the molecular and cellular activities of a simple homemade ethanol-based garlic extract (GE). We show that GE inhibits growth of several different cancer cells in vitro, as well as cancer growth in vivo in a syngeneic orthotopic breast cancer model. Multiple myeloma cells were found to be especially sensitive to GE. The GE was fractionated using solid-phase extractions, and we identified allicin in one GE fraction; however, growth inhibitory activities were found in several additional fractions. These activities were lost during freeze or vacuum drying, suggesting that the main anti-cancer compounds in GE are volatile. The anti-cancer activity was stable for more than six months in −20 °C. We found that GE enhanced the activities of chemotherapeutics, as well as MAPK and PI3K inhibitors. Furthermore, GE affected hundreds of proteins involved in cellular signalling, including changes in vital cell signalling cascades regulating proliferation, apoptosis, and the cellular redox balance. Our data indicate that the reduced proliferation of the cancer cells treated by GE is at least partly mediated by increased endoplasmic reticulum (ER) stress.

## 1. Introduction

The complex phytochemistry of garlic (*Allium sativum*) has been the subject of thousands of research papers, more than two thousand in the last decade alone. Numerous studies have confirmed the beneficial effects of garlic on the cardiovascular system [1], immunomodulation and cancer (reviewed in [2,3,4]), as well as its antioxidant [5] or oxidant properties [6]. Direct antibacterial and antiviral properties have also been described, with allicin being regarded as responsible for the antibacterial effects [7]. Multiple recent studies have linked garlic intake with protective effects against a range of cancers, and the conclusion that raw garlic has health benefits is gaining momentum [8,9,10,11,12]. 

Most of the biological effects of garlic are shown to come from organosulfur compounds (OSCs) originated from allicin. Allicin is produced by the enzyme allinase from alliin, and is further processed/degraded. Allinases are part of the defence system of the plant, and are released when the plant is damaged. Garlic-derived OSCs are shown to reduce expression and activation of multiple cell-growth stimulatory proteins and to target most of the cancer hallmarks defined by Hanahan and Weinberg [13], (reviewed in [14]). OSCs are also believed to affect the cellular redox systems—e.g., the activation of cysteinyl S-conjugates in OSCs via β-lyase reactions leads to reactive persulfide or sulfane sulfur progenitors. These may, in turn, react with cysteine moieties on redox-sensitive proteins—for example, in proteins important in cellular signalling [15]. It has been shown that S-allylcysteine from garlic suppresses the growth of human prostate cancer cells [16], and that allicin can induce both caspase-dependent [17,18] and independent [19] apoptosis in various cancer cells, as well as ameliorate the toxic effects of chemotherapeutics [20]. Diallyl sulfides, e.g., diallyl disulfide (DADS) and diallyl trisulfide (DATS), which arise from degradation of allicin, are shown to have anti-cancer activities via the promotion of apoptosis and cell cycle arrest [3,21,22]. A problem when comparing the different reports on the biological effects of garlic is that different types of purified OSCs or garlic extracts have been used. 

The increased interest in garlic for cancer prevention led us to examine the molecular effects of a “kitchen/homemade” garlic extract (GE). In terms of treating cancer, the use of traditional remedies is by no means preferred over established clinical therapies; however, modifications to the patient’s diet, by—for example—including various biological extracts, can be beneficial, and may give better therapeutic outcomes, as well as improved prevention of cancer reoccurrence [4,12,23,24,25]. Here, we demonstrate how a homemade GE produces profound effects on multiple signalling cascades, inhibits proliferation of cancer cells in vitro and in vivo, improves the efficacy of known antitumor drugs in vitro, and contributes to reduced cancer growth in a pre-clinical mouse model for breast cancer. 

## 2. Materials and Methods

### 2.1. Reagents

The chemotherapeutics used were docetaxel (Vnr 513113, Actavis, Parsippany, NJ, USA), cisplatin (Vnr 146262, Hospira and Accord, Forest Lake, IL, USA), and gemcitabine (Vnr 172819, Actavis). Kinase inhibitors against p38 (SB20358), PI3K (LY294002,) and JNK (SP600125) were purchased from Sigma-Aldrich (Saint Louis, MO, USA). Kyolic (Aged Garlic Extract^TM^, Mission Viejo, CA, USA) was purchased at Sunkost, Trondheim, Norway.

### 2.2. Homemade Garlic Extract

Our garlic extract (GE) was made by crushing 350 g garlic cloves (in order to activate allinase) in 250 mL 40% ethanol using a bench-top blender. This crude garlic-ethanol mixture was transferred to a glass jar with an air-tight lid, and stored in darkness at 4 °C for 5 days. The liquid was then squeezed through a cloth or nylon stocking. The dry mass was discarded, while the solution was distributed in 50 mL centrifuge tubes and centrifuged at 5000 rpm for 10 min in a bench-top centrifuge. The supernatant was collected and stored at −20 °C. The extract contained ~22% ethanol (measured using Megazyme enzymatic ethanol concentration kit). 

### 2.3. Cell Culturing and Cell Growth Measurements (MTT-Assay)

Mammalian cancer cell lines (DU145, U2OS and 67NR) were sub-cultured and maintained in DMEM or RPMI-1640 (BioWhittaker, Walkersville, MD, USA) supplied with 10% FBS, 250 µg/mL amphotericin B (Sigma-Aldrich), 100 µg/mL gentamycin (Invitrogen, Carlsbad, CA, USA), and 2 mM glutamine (BioWhittaker). JJN3 and RPMI-8226 cells were grown in RPMI-1640 (Sigma, St. Louis, MO, USA) with the same supplements. Cells were seeded out in 96 well plates (5000 cells/well) and garlic extracts, cytostatic drugs, and combinations of these in given concentrations were added the same day. Cells were harvested on days 1–4 using the MTT (3-(4,5-dimethylthiazol-2-yl)-2,5-diphenyltetrazolium bromide)-assay as described [26]. The average from six wells was used to calculate cell survival.

### 2.4. Comet Assay (Alkaline Single-Cell Gel Electrophoresis) 

67NR cells were treated for 24 h at 37 °C with GE (1:800), alone or in combination with cisplatin (Cis) (2 and 3 μM). Cells were harvested in ice-cold 30% FCS in PBS and subjected to lysis overnight, alkaline DNA unwinding (pH > 13.3), and single-cell electrophoresis, as described [27]. A total of 100 comets were selected randomly from each slide and evaluated using Komet 5.0 imaging software (Andor Technology, Belfast, UK).

### 2.5. Bone Marrow Stromal Cell-Assay 

Fresh CD138-positive myeloma cells were isolated from bone marrow samples obtained from the Norwegian Myeloma Biobank, using a RoboSep automated cell separator and Human CD138 Positive Selection Kit (StemCell Technologies, Grenoble, France). Cell death in primary myeloma cell isolates was measured essentially as described [28]. Briefly, bone marrow stroma cells (BMSC) (2500 cells/well) and primary myeloma cells (5000 cells/well) were seeded in RPMI (Roswell Park Memorial Institute) media containing 2% human serum in a 96 well plate, and incubated with GE as indicated for three days. Cell viability was measured after staining with the apoptotic marker YO-PRO-1 (1 μM, Invitrogen, Carlsbad, CA, USA) and nuclear stain DRAQ5 (2.5 μM, eBioscience, San Diego, CA, USA). Cell staining were quantified as described, applying ScanR automated image acquisition and analysis [28]. Ethics statement: the study on patient myeloma cells were approved by the Regional Committee for Medical and Health Research Ethics Central Norway (REC Central, permit numbers: REK 2011/2029 and REK 4.2007.933) and the patients had given written informed consent.

### 2.6. Orthotopic Mammary Cancer Model in Mice

The 67NR cell line is derived from a spontaneous tumour in a Balb/cfC3H mouse [29]. 67NR cells were kindly provided from Fred Miller, Wayne State University, Detroit, MI, United States. Animal experiments were approved by the Norwegian Food Safety Authority (FOTS application 6874) and performed in the Unit of Comparative Medicine, NTNU. 67NR cells (4 × 10^5^ cells in 20 µL PBS) were injected into the fourth mammary fat pad of 32 female Balb/C mice (8 weeks, Taconic, Rensselaer, NY, USA). The mice were anesthetized with isoflurane during the injections. 200 µL of a solution of 0.90% w/v of NaCl, containing 6.5 µL of GE injected intraperitoneally (i.p.) (*n* = 16). This dose is equivalent to 15 mL/day in humans, based upon surface area calculations. The vehicle group (*n* = 16) was injected daily with 200 µL of a solution of 0.90% *w*/*v* of NaCl, containing 6.5 µL 20% ethanol. When the tumours were palpable, the tumour sizes were measured by electronic Vernier Calipers three times a week. The body weight was measured twice a week throughout the experiment. Tumour volumes were calculated using the formula for a spheroid: $\frac{4}{3}\times a^{2}\times b\times \pi$, where 2*a* is the tumor width and 2*b* is the tumor height. After 28 days, the mice were euthanized using carbon dioxide (2 L per min). In an additional experiment, cisplatin (3 mg/kg) and gemcitabine (0.5 mg/kg) in 200 µL 0.90% *w*/*v* of NaCl solution were injected i.p. (*n* = 15) (FOTS application 7133), alone or in combination with GE (6.5 µL in 200 µL 0.90% *w*/*v* of NaCl solution) (*n* = 15). The vehicle group (*n* = 14) was injected daily with 200 µL of a solution of 0.90% *w*/*v* of NaCl, containing 6.5 µL 20% ethanol. Data from this experiment is shown until day 27. Three animals from the vehicle group were terminated on day 27, because of the tumor sizes exceeded the limit. The remaining animals were terminated on day 29.

### 2.7. Preparation of Cell Extracts and Western Analysis 

The 67NR cells were treated with GE at given concentrations. The cells were harvested after 4 and 24 h, the cell pellet was re-suspended in 1× packed cell volume of buffer 1 (10 mM Tris-HCl pH 8.0, 200 mM KCl), and diluted in the same volume (packed cell volume + buffer 1) of buffer 2 (10 mM Tris-HCl pH 8.0, 200 mM KCl, 10 mM EGTA, 10 mM MgCl_2_, 40% glycerol, 0.5% NP40, 1 mM DTT, 1% phosphatase inhibitor cocktails 1 and 3 (Sigma-Aldrich), 2% Complete EDTA-free protease inhibitor (Roche, Basel, Switzerland), and 2 µL/mL Omnicleave (Epicentre Technologies, Madison, WI, United States). After incubation for 1.5 h at 4 °C, the cell extracts were centrifuged at 14,000 rpm for 10 min. Supernatants were collected and separated on 10% Bis-Tris gels (NuPAGE, Invitrogen). After gel electrophoresis, the polyvinylidene fluoride membranes (Immobilion, Millipore, Burlington, MA, USA) were blocked in 50% Odyssey blocking buffer (LI-COR Bioscience) in TBS (Tris-buffered saline). The primary antibodies against AKT (phospho-Ser473), ERK1/2 (phospho-Thr202/Tyr204/phospho-Thr185/Tyr187), p70 S6 kinase (phospho-Thr389) (Cell Signaling, Danvers, MA, USA), and β-tubulin (Abcam, Cambridge, UK), as well as the fluorescently-labelled secondary antibodies, goat anti-rabbit 680RD and goat anti-mouse 800CW (LI-COR Bioscience) were diluted in 20% Odyssey blocking buffer in TBST (TBS with 0.1% Tween 20). The proteins were visualized with the Odyssey infrared imaging system (LI-COR Bioscience) and quantified using Odyssey Image Studio V2. Protein levels were compared to the protein level in untreated cells, which was set to 100%. β-tubulin was used as reference for data normalization.

### 2.8. Multiplexed Inhibitor Assay and Mass Spectrometry Analysis

Three different kinase inhibitors (Purvalanol B (Tocris Bioscience), Bisindolmaleimide X (Activate Scientific), and SB6-060-05 [30]) were immobilized using ECH Sepharose 4B and EAH Sepharose 4B (GE Healthcare) beads, according to the manufacturer’s instructions and as published elsewhere [31]. The following steps were performed as described [32], using 100 µL (0.1 mg) of cell extract per column (50 µL of mixed inhibitor beads).

### 2.9. Fractionation and Purification of Garlic Extract

GE (1 mL) was diluted 1:10 with distilled water and loaded on a SepPac SPE tC_18_ 1cc 100 mg cartridge (Waters, Milford, MA, USA), preconditioned with ethanol and distilled water. The SPE column was washed with 2 mL distilled water, before eluting off the GE-fractions with a 2 mL stepwise increased ethanol concentration (10, 20, 40, and 60% ethanol), GE10–GE60. These fractions were directly tested for anti-proliferation activity using the MTT-assay. The 20% fraction was further purified for NMR analysis using a Waters Acquity UPLC system. A Waters Acquity BEH C18 column (2.1 × 100 mm) was used with water and methanol as mobile phases A and B, respectively, both added 0.1% formic acid. The gradient was optimized for a rapid 5-min run, where allicin was eluted at 2.0–2.2 min. This was confirmed by the testing of bioactivity in all 0.5-min fractions. Using the optimized gradient, the bioactivity containing a fraction of 100 injections was collected using a divert valve on the HPLC. This pooled fraction was vacuum-dried and dissolved in D_2_O prior to NMR analysis. ^1^H NMR spectra were recorded at room temperature on a Bruker Avance III 600 NMR spectrometer.

### 2.10. Isolation and Stimulation of Peripheral Blood Monocytes

Peripheral blood mononuclear cells were isolated from donor blood, as previously described [33]. Lipopolysaccharide (LPS) (10 ng/mL) (Sigma-Aldrich) and GE (1:1000, 1:2000, and 1:3000) was added to the adherent cells. The cells were incubated for 4 h before the supernatants were harvested and frozen prior to further cytokine analysis by the 27-plex assay (Bio-Plex Pro^TM^ Human cytokine 27-plex assay). Monocytes from two different donors were used. 

## 3. Results and Discussion

### 3.1. Garlic Extract Reduces Cell Growth of Multiple Myeloma and Prostate Cancer Cells

**Figure 1:**
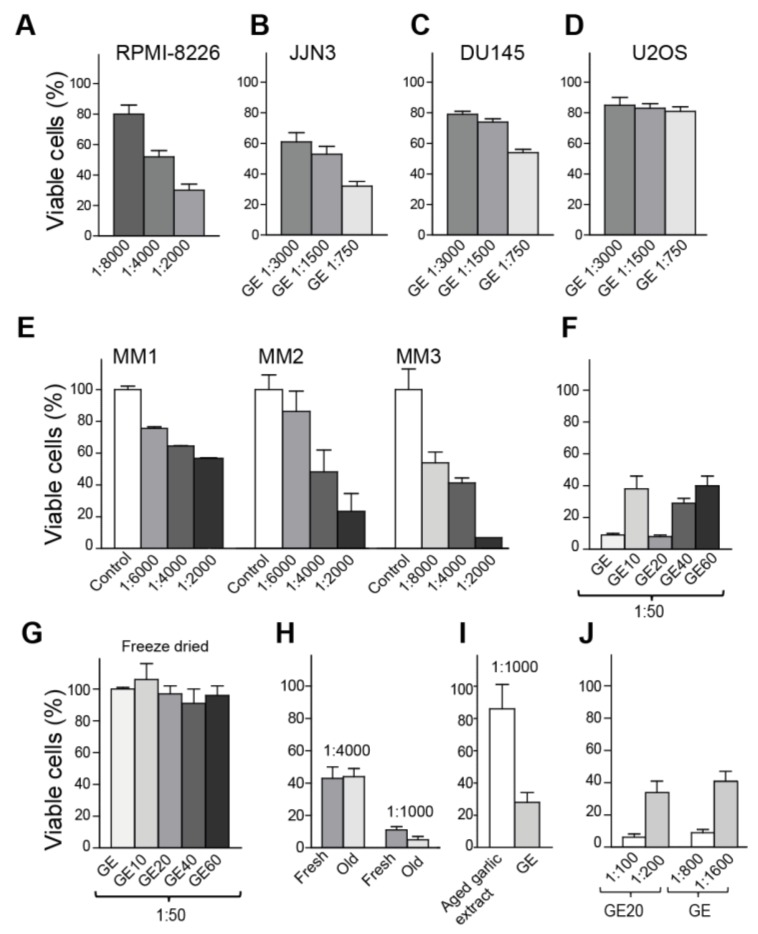
Volatile compounds in garlic extract (GE) inhibit growth of multiple cancer cells. (**A**–**D**) Viability measured by MTT-assay on day four, after the addition of three dilutions of GE for (**A**) RPMI-8226 (multiple myeloma (MM)), (**B**) JJN3 ((MM), (**C**) DU145 (prostate cancer), and (**D**) U2OS (osteosarcoma). The percentage viabilities are relative to vehicle-treated cells. (**E**) Viability of non-proliferating primary MM cells co-cultured with bone marrow stromal cells (BMSC-assay), measured on day three. MM cells from three patients (MM1-MM3). Averages from two parallel samples with SD are shown. (**F**–**J**) Viability (MTT) measured relative to vehicle treated cells in JJN3 cells (**F**) after the addition of unfractionated GE and fractionated GE extracts (GE10–GE60) on day three, (**G**) after the addition of freeze-dried and re-suspended GE and GE10-GE60 on day three, (**H**) after the addition of fresh and old GE (~6 months, stored −20 °C) on day four, (**I**) after the addition of GE and the commercially-available aged garlic extract (Kyolic) on day three, and (**J**) after a dose response of GE and GE20 on day four. The data presented represents one experiment out of at least two similar experiments with the same results. Mean relative viability ± SD of 3–6 technical replicas are shown.

The homemade GE was found to reduce the viability of the two multiple myeloma (MM) cell lines, RPMI-8226 and JJN3, as well as the prostate cancer cell line DU145 in a dose-dependent manner, while the growth of the osteosarcoma cell line U2OS was not affected at the same doses (Figure 1A–D). Treatment with 22% ethanol alone (final ethanol concentration in GE), diluted 1:50 in growth media, had no effect on the viability. These results illustrate variations in sensitivity toward GE for different cancer cell lines, and suggest that MM cells are hypersensitive to GE. We next tested the ability of GE to induce apoptosis in non-proliferating primary MM cells, co-cultured with bone marrow stromal cells in a BMSC-assay [28,34]. Interestingly, GE diluted up to 8000 times reduced the viability of primary MM cells, while not affecting the BMSC in the co-culture (part of the assay criteria) (Figure 1E). These results further support that MM cells are hypersensitive to GE. 

### 3.2. Freeze Drying Deteriorates the Anti-Cancer Activity of the Garlic Extract 

Three-months-old GE (stored at −20 °C) was next fractionated using solid-phase extraction, in an attempt to characterize the nature of the most active compounds. Fractions were tested for growth-inhibitory activities, and the largest reduction in viability, compared to the untreated control, was found in the fraction eluted with 20% ethanol (GE20, Figure 1F); however, the other fractions also reduced the cell growth. Interestingly, freeze-drying of both fractionated and unfractionated extracts abolished their anti-growth activities (Figure 1G). The same was observed with vacuum drying (data not shown). Thus, volatile substances are vital for the anti-growth activities.

Allicin, which is regarded to be one of the most active OSCs, is reported to have low stability and to be highly dependent on pH, temperature, and solvent (water versus alcohol) [35,36]. Thus, next we tested the stability of GE upon storage at −20 °C, 4 °C, and room temperature (RT). The anti-growth activity was found to be stable for more than six months (old GE) when stored at −20 °C (Figure 1H), for at least four weeks at 4 °C, and at least one week at RT (data not shown). Kyolic is a commercially-available, water-based, aged garlic extract, and this extract is stored at RT. We compared the viability of cells treated with GE and aged garlic extract (Kyolic), and found that GE had a considerably stronger activity at same dilution (1:1000) (Figure 1I). This effect is, as discussed above, not due to the ethanol content of the extract. The anti-growth activity of GE was stable upon heating to 100 °C for 1 h, if the lid was used to prevent evaporation, and also under acidic conditions (HCl to final pH = 1) (data not shown). Allicin was identified in the GE20 fraction (purified with HPLC and identified by NMR, data not shown). However, approximately eight times more GE20 was needed to give the same reduction in viability as unfractionated GE (eight times lower dilution, Figure 1J), suggesting that the allicin containing GE20 fraction contains only ~12–13% of the anti-growth activity in unfractionated GE. In addition, because the antigrowth activities were stable after heating and acid addition, most of the activity in GE is likely not due to allicin, but to other volatile OSCs. GC-MS analysis of unfractionated GE supports this, as the volatile fraction contained several OSCs, including DADs and its isomers (Supplementary Figure S1).

### 3.3. Garlic Extract Increases the Activity of Commonly Used Anti-Cancer Drugs

**Figure 2:**
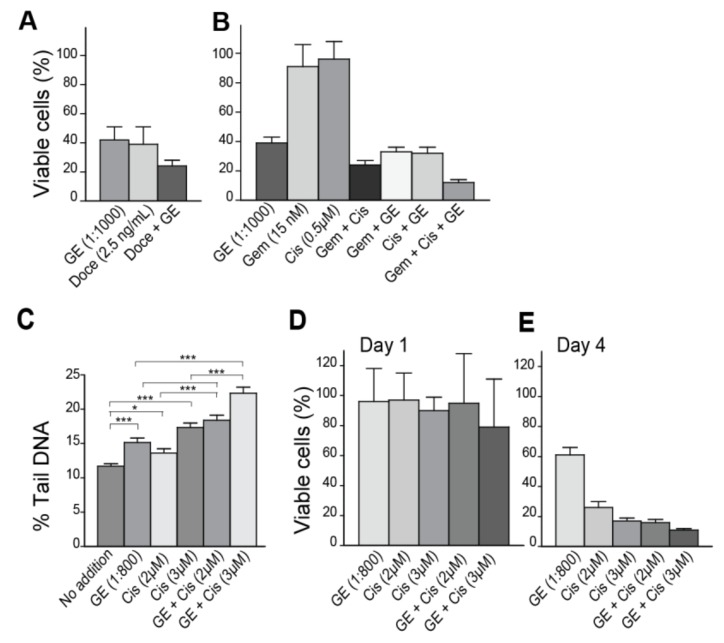
GE inhibits growth and increases the efficacy of genotoxic drugs in breast cancer cells. (**A**,**B**) Viability of the breast cancer cell line 67NR, measured by MTT-assay on day four, after the addition of (**A**) GE (diluted 1:1000), docetaxel (Doce) (2.5 ng/mL), and a combination of these at same concentrations; and (**B**) GE (diluted 1:1000), cisplatin (Cis) (0.5 µM), gemcitabine (Gem) (15 nM), and double and triple combinations of these at the same concentrations. (**C**) Comet assay on 67NR cells on day one (16–20 h), after the addition of GE diluted at 1:800, alone and in combination with high dose cisplatin (Cis) (2 and 3 µM). Data presented are from two independent biological replicas. Two tailed, unpaired *t*-test, *** *p* < 0.001, * *p* < 0.02. (**D**,**E**) Viability of 67NR cells measured by MTT-assay on (**D**) day one and (**E**) day four, after the addition of GE (diluted 1:800), alone and in combination with cisplatin (Cis) (2 and 3 µM). All MTT data presented represent one experiment out of at least two similar experiments with the same results. Mean relative viability ± SD of 3–6 technical replicas are shown.

Garlic is potentially beneficial for cancer prevention, and as such is also recommended as a food supplement for cancer patients. We therefore next examined how unfractionated GE affected the efficacy of some commonly used anti-cancer drugs. A GE concentration leading to 40% viable cells on day four increased the efficacy of the DNA-damaging agents docetaxel, gemcitabine, cisplatin, and the combination of gemcitabine and cisplatin (Figure 2A,B). Others have also found that garlic compounds can increase the activity of chemotherapeutics, e.g., it was found that allicin-sensitized hepatocellular cancer cells to 5-fluorouracil in a xenograft mouse model [37]. 

We next examined if GE affected the levels of DNA damage as a single agent, or in combination with a genotoxic agent, using the Comet assay. GE alone slightly increased the percentage of tail DNA (% Tail) (representing cumulative levels of abasic sites, as well as single- and double-strand DNA breaks) measured at day one, compared to untreated cells (Figure 2C). Cisplatin is a highly genotoxic drug causing intra- and inter-strand crosslinks in DNA; the intra-strand crosslinks were repaired within hours, while repair of the inter-strand crosslinks were more time consuming (>1 day) and required multiple repair pathways [38]. Viability on day 1 after treatment with cisplatin were only slightly affected (Figure 2D) and the corresponding levels of DNA damage were as expected low (~15–18%, Figure 2C), even though cisplatin at the same doses gave only ~20% viable cells on day four (Figure 2E). GE in combination with cisplatin increased the DNA fragmentation, compared to GE or cisplatin alone (*p* < 0.001, Figure 2C). We suspect that the increased fragmentation by GE, both when used as a single agent and in combination with cisplatin, is mediated via changes in cellular signaling, regulating the DNA damage response and apoptosis, in accordance with previous publications on OSCs (reviewed in [14]). The levels of endogenous DNA lesions that occur in each cell per day is estimated to 20,000–50,000 [39]. Cancer cells have even higher levels of endogenous lesions than normal cells, both due to higher levels of replicative stress and a less stringent cell cycle control. Cancer cells are therefore likely to be more affected than normal cells by agents that affect cell cycle control, cell signaling, and DNA repair [40]. This may explain some of the cancer-preventing effects of garlic reported. 

### 3.4. Garlic Extract Causes Large Changes in Cellular Signalling

**Figure 3:**
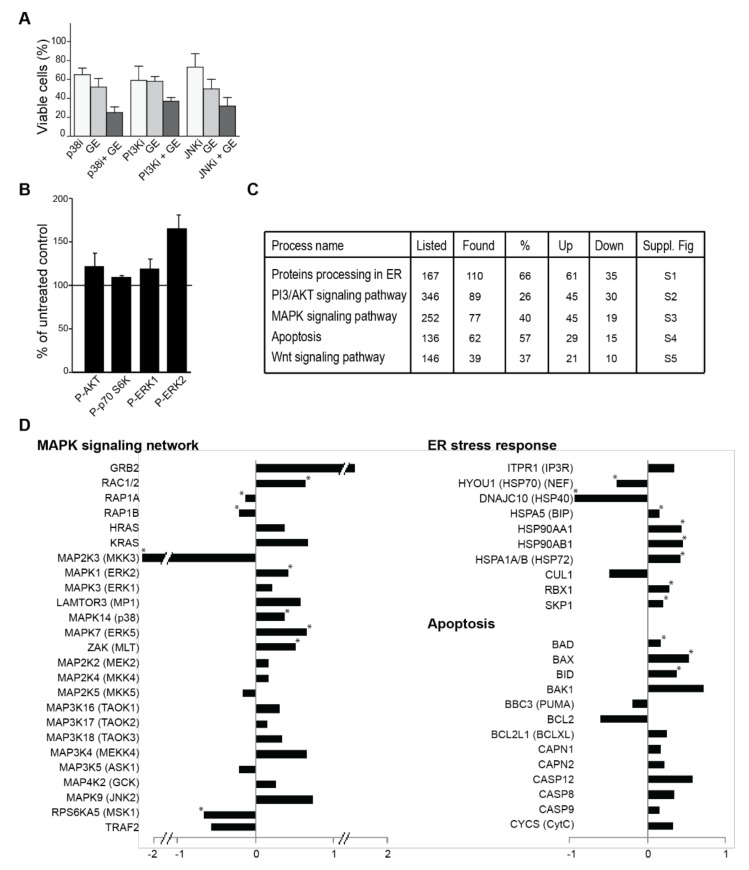
GE influences cellular signalling. (**A**) The viability of JJN3, measured by MTT-assay on day three after the addition of GE diluted 1:4000 in combination with p38 (SB20358, 10 µM), PI3K (LY294002, 7 µM), and JNK (SP600125, 10 µM). The viability of the different cell lines in growth media with equal amounts of DMSO, as in 10 µM kinase inhibitor, are similar to the control. The mean relative viability ± SD of 3–6 technical replicas are shown. (**B**) Quantification of the western analysis of cell extracts from 67NR cells, treated with different dilutions of GE (1:1000) for 24 h, compared to untreated control. The % phosphorylated Akt (Ser473), ERK1 (Thr202/Tyr204), ERK2 (Thr185/Tyr187), and S6K (Thr389) extracts from the untreated control (100%, black horizontal line) are shown. Data represents mean ± SD from three biological replicas. (**C**) Relevant cellular pathways from KEGG. Numbers of proteins listed in each pathway, as well as the number and percentage of found proteins are given. Number of proteins that increased (up) and decreased (down) in the pull down are given. Overlays of our quantitative date into KEGG pathway diagrams are shown in the corresponding Supplementary Figures S1–S5. (**D**) A subset of signalling proteins identified using the MIB-assay from Supplementary Table S1 is shown as log2 values of changes in protein levels relative to the control. * indicate significant changes, according to a two-sided non-parametric Wilcoxon Sign Rank Test, as described in [32] (*n* = 3).

Changes in multiple signalling pathways are reported in cells exposed to various OSCs (reviewed in [14]). We found that GE increased the efficacy of inhibitors against p38 (SB20358), PI3K (LY294002) and JNK (SP600125), and western blot analysis suggested that GE affected the MEK/ERK and PI3K/AKT/S6K signalling pathways (Figure 3A,B), supporting the idea that OSCs can influence multiple signalling pathways. In order to get a more comprehensive view of how cellular signalling was affected by GE, we analysed the extracts from treated cells, using the non-targeted, highly sensitive, and reproducible multiplexed inhibitor bead (MIB)-assay [31,32]. This is an assay in which multiple kinase inhibitors coupled to sepharose beads are used as an affinity matrix for kinases and NTP interacting proteins/protein complexes, prior to identification by mass spectrometry. Using this assay, 4885 proteins were detected and quantified, including 67 signalling phosphatases, 119 ubiquitin ligases, and 189 signalling kinases. Significant changes in GE-treated versus untreated cells were found for over 1000 proteins (quantitative changes of all proteins detected are shown in Supplementary Table S1). KEGG (Kyoto Encyclopedia of Genes and Genomes) analysis of these proteins showed that GE-induced changes in proteins involved in endoplasmic reticulum (ER) processing, PI3K/AKT, MAPK, apoptosis and Wnt signalling (Figure 3C). Quantitative changes of some of the signalling proteins in these pathways are shown in Figure 3D, including multiple proteins in the ER stress response.

In an attempt to further interpret the mechanisms for the reduced growth that was observed, we next used the KEGG pathway diagrams corresponding to the pathways identified to be most affected (Figure 3C), as a basis to overlay our quantified MS data (Supplementary Figures S2–S6). This further indicated that activation of the apoptotic pathways downstream of the ER could be important for explaining the anti-growth activities of GE (Supplementary Figures S2 and S5). OSCs were previously reported to affect protein folding, leading to ER stress; OSCs can both bind to glutathione (GSH), and to cysteine residues on proteins, and thereby modify both the cellular redox state as well as protein folding [14,41,42,43,44]. We could not detect any changes in cellular GSH levels after treatments with GE (Supplementary Figure S7); however, in support of increased ER stress after GE treatment, we detected an increased pulldown of HSPA5 (BIP), a member of the Hsp70 family known to be markedly induced upon conditions leading to the accumulation of unfolded proteins in the ER (also known as GRP78) (Figure 3D, Supplementary Figure S2). HSPA5 (BIP) can lead to apoptosis through several pathways, one of them being activation of the JNK (c-Jun NH2-terminal kinase) pathway. The MAPK (mitogen-activated protein kinase) pathway responds to changes in the redox state of the environment [45,46,47]. Indeed, we detect significant increases in RAC1/2 and multiple MAPK kinases, suggesting that GE affects the redox state (Figure 3D). The detected MAPK9 (JNK2) increase and the BCL2/BAX ratio decrease (Figure 3D) is also reported by others after OSC treatments [14,48]. BCL2 itself is shown to be important for the regulation of the cellular redox capacity, and to be sensitive to the presence of reductive species, such as OSCs [49,50]. Furthermore, we detected a significant increase in several ubiquitin ligases that are involved in the ER stress response, and which target (among others) β-catenin for degradation (RBX1 and SKP1) (Figure 3D, Supplementary Figures S2 and S6). It has been previously reported that OSCs reduce β-catenin signalling [14,21], and we also found a reduction in the β-catenin level (Supplementary Figure S6). In further support for increased ER stress induced by GE, which will lead to elevated ROS-levels and oxidative stress, we found a significant increase in proteins activated by and important for regulating cellular ROS levels, e.g., OXR1, Txnl1, Hmox2, and Sirt1 (Supplementary Table S1). OXR1 is, for example, crucial for cellular protection against ROS, and is shown to regulate the p53 signalling network [51]. 

We could not detect any changes in redox couples important in the cellular defense against oxidative stress (e.g., NAD^+^/NADH and NADP^+^/NADPH) in the GE-treated cells (Supplementary Table S2), despite the changes detected in proteins important for redox regulation. Furthermore, glucose consumption, as well as lactate secretion, were not changed. While the proteome and metabolome in cells are highly dynamic and connected, they are separated in time, and one time point may not detect changes in both the metabolome and proteome simultaneously. 

Many cancer cells have problems with misfolded proteins, and have therefore often developed more efficient proteasome pathways than normal cells. Because MM cells produce high levels of monoclonal immunoglobulins, this particular cancer type is hypersensitive to problems with misfolding in ER, and therefore initially respond very well to proteasome inhibition [52]. Apoptosis mediated by increased ER stress might therefore explain the hypersensitivity towards GE observed in MM cells.

### 3.5. Garlic Extract Reduces Growth of Mammary Tumors In Vivo

**Figure 4:**
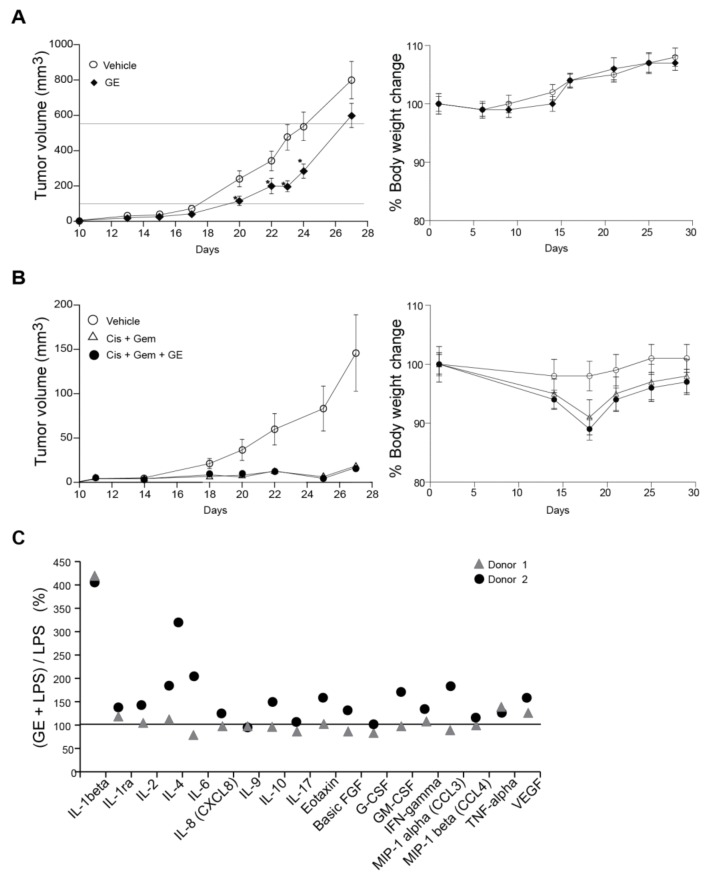
GE reduces breast cancer growth in vivo and partly influences the cytokine response from lipopolysaccharide (LPS)-stimulated monocytes. (**A**,**B**) 67NR breast cancer cells were injected into the fourth mammary fat pad of female BalB-C mice. (**A**) The mice were treated with vehicle (6.5 µL 20% ethanol in 200 µL NaCl, *n* = 16) and GE (6.5 µL in 200 µL NaCl, *n* = 16) intraperitoneally (i.p.) once every day from day one (two-tailed, unpaired, *t*-test, * *p* < 0.05). (**B**) The mice that were treated with vehicle (6.5 µL 20% ethanol in 200 µL NaCl, *n* = 16), Gem + Cis (gemcitabine (0.5 mg/kg) + cisplatin (3 mg/kg), i.p., 200 µL, *n* = 15, given once per week for three weeks, starting at day eight), alone and in combination with GE (6.5 µL in 200 µL NaCl, *n* = 16, i.p., once every day from day one). Mean ± SEM are shown. (**C**) The % increased levels of cytokines released by monocytes stimulated with GE (1:1000) + LPS (10 ng/mL), relative to monocytes stimulated by LPS only (horizontal line). Only cytokines that had >2 folds induction by addition of LPS are shown.

Anti-cancer activities of GE on cancer cell lines in vitro may differ completely from the effects obtainable in vivo. Still, different garlic preparations are shown to have promising anti-cancer activities in multiple animal cancer models (reviewed in [14]). Here, we examined GE’s efficacy using an orthotopic, immunocompetent (syngeneic) mouse model, where the breast cancer cells (67NR) are implanted in the mammary pads of BalB/c mice to mimic solid breast cancer [29]. This is an aggressive and rapidly-growing tumour model, and we therefore started the GE treatment the same day as the tumour cells were implanted. Mice treated with daily i.p. injections of GE showed ~30% delayed cancer growth compared to untreated individuals, and a significant difference between the groups were found on days 20–24, when the average tumour volumes were between 100 and 550 mm^3^ (Figure 4A, between the horizontal lines). We also examined if GE affected the efficacy of a combination treatment of gemcitabine and cisplatin using the same model. The overall tumour growth was slower in this experiment, thus the doses of gemcitabine-cisplatin used were too high to detect any potential increased efficacy of the GE + gemcitabine + cisplatin combination; however, the GE did not reduce the efficacy of the cisplatin + gemcitabine treatment (Figure 4B). Of note, the mice in the GE + gemcitabine + cisplatin combination group appeared more active and vital than the gemcitabine + cisplatin-treated mice (subjective observations). Garlic is reported to reduce cisplatin-induced nephrotoxicity and oxidative stress [53], which could explain the better performance of these animals. 

### 3.6. Garlic Extract Has Limited Effects on Interleukin Signaling

Several studies have reported anti-inflammatory effects of garlic compounds [54]. We isolated peripheral blood monocytes from two human donors, and stimulated the cells with GE, in combination with LPS. IL-1beta secretions were increased about four-fold, and both donors also had a small increase in the secretion of TNF-alpha (tumor necrosis factor alpha) and VEGF (vascular endothelial growth factor) when stimulated with GE + LPS (Figure 4C). The other cytokine changes were inconclusive due to donor variations. A small increase in secretion of IL-1beta, IL-6, IL-9, CCL3, CCL4, and TNF-alpha were also detected from monocytes stimulated with GE alone (~1.5-fold increase, data not shown). Overall, GE induced small changes in the cytokine profile of LPS-stimulated monocytes at doses that strongly affected cell growth of sensitive cancer cells. Nevertheless, such small changes in cytokine and chemokine profiles might also contribute to the observed in-vivo anti-cancer effects via the fine-tuning of the immune system. In agreement with our data, alliin was previously reported to increase the spontaneous and LPS-induced IL1-beta secretion from PBMCs [55]. 

### 3.7. Concluding Remarks

The beneficial effects of garlic have been known for centuries, and our understanding of the molecular mechanisms that underlay these effects is steadily increasing. Here, we show that a homemade garlic extract has anti-cancer activities both in vitro and in vivo, and that the activity is stable for more than six months if stored in a freezer. In agreement with multiple recent studies, our data support a mechanism by which OSCs in GE trigger apoptosis, via ER stress and effects on the cellular redox regulation. However, the molecular mechanisms of the anti-cancer effects mediated by garlic are highly complex, and likely different in different cancer cells.

## Supplementary Materials

The following are available online at http://www.mdpi.com/2072-6643/10/4/450/s1, Figure S1: Headspace GC-MS chromatogram of unfractionated garlic extract (GE); Figure S2: Protein processing in endoplasmic reticulum (KEGG ID mmu:04141); Figure S3: PI3K-Akt signaling pathway (KEGG ID mmu:04151); Figure S4: MAPK pathway (KEGG ID mmu:04010); Figure S5: Apoptosis pathways (KEGG ID mmu:04210); Figure S6: WNT signaling pathway (KEGG ID mmu:04310); Figure S7: GE does not affect glutathione (GSH) levels in multiple myeloma cells (JJN3); Table S1: Quantification of changes in protein levels in GE treated cells; Table S2: Metabolite analysis.
