# Supplementary material for: Anti-Cancer Potential of Homemade Fresh Garlic Extract Is Related to Increased Endoplasmic Reticulum Stress
Source: Nutrients. 2018 Apr 5;10(4):450. doi: 10.3390/nu10040450 (PMC5946235; doi:10.3390/nu10040450)
Supplement: Supplementary file 1 [file nutrients-10-00450-s001.zip › nutrients-283204-Table S1.pdf]

Supplementary Table S1: Quantification of changes in protein levels in GE treated cells

	MS based quantifiaction of proteins pulled down by the MIB-assay. Data is given as log2 values of relative intensity. **Red** indicates increase, **blue** indicates decrease. **Black** indicates no change compared to control.				Wilcoxon test significance	Uniprot			KEGG Pathway Database	Important groups of proteins discussed in the manuscript, "1" indicates protein belonging to group.				
nr	Paralel 1	Paralel 2	Paralel 3	Average	Significant (yellow)	Uniprot ID	Protein Name	Gene	KEGG code	Kinases	Oxidative stress response protein	Phosphatases	Ubiquitin Ligases	Apoptosis related
1	0.2735	0.5011	0.0624	0.2661	1	P41241	Tyrosine-protein kinase CSK	Csk	mmu:12988	1				
2	0.1317	0.2554	0.1521	0.2856	1	O55222	Integrin-linked protein kinase	Ilk	mmu:16202	1				
3	0.3960	0.0493	0.3058	0.3680	1	P47811	Mitogen-activated protein kinase 14	Mapk14	mmu:26416	1				
4	0.1562	0.6205	0.2559	0.3690	1	Q61194	Phosphatidylinositol 4-phosphate 3-kinase C2 domain-containing subunit alpha	Pik3c2a	mmu:18704	1				
5	0.2356	0.3554	0.4045	0.3704	1	P30285	Cyclin-dependent kinase 4	Cdk4	mmu:12567	1				
6	0.3112	0.4525	0.4779	0.3789	1	P34152	Focal adhesion kinase 1	Ptk2	mmu:14083	1				
7	[illegible]	0.2480	0.1410	0.3957	1	Q5EG47	5-AMP-activated protein kinase catalytic subunit alpha-1	Prkaa1	mmu:105787	1	1			1
8	0.1779	0.5581	0.1839	0.4095	1	O70551	SRSF protein kinase 1	Srpk1	mmu:20815	1				
9	0.1458	0.2131	0.3468	0.4245	1	Q2TBE6	Phosphatidylinositol 4-kinase type 2-alpha	Pi4k2a	mmu:84095	1				
10	0.2818	0.1876	0.7366	0.4632	1	Q8BTW9	Serine/threonine-protein kinase PAK 4	Pak4	mmu:70584	1				
11	0.8235	0.4557	0.3463	0.4865	1	P58801	Receptor-interacting serine/threonine-protein kinase 2	Ripk2	mmu:192656	1				1
12	0.3867	0.4231	0.0928	0.4948	1	Q6PHZ2	Calcium/calmodulin-dependent protein kinase type II subunit delta	Camk2d	mmu:108058	1				
13	0.1962	0.2806	0.3059	0.5223	1	Q9ESL4	Mitogen-activated protein kinase kinase kinase MLT	Zak	mmu:65964	1				1
14	1.0821	0.2689	0.4199	0.6247	1	P20444	Protein kinase C;Protein kinase C alpha type	Prkca	mmu:18750	1				1
15	0.4281	0.0453	0.5734	0.6497	1	Q8R4K2	Interleukin-1 receptor-associated kinase 4	Irak4	mmu:266632	1				
16	0.7036	0.2019	0.4113	0.6953	1	Q9WUN2	Serine/threonine-protein kinase TBK1	Tbk1	mmu:56480	1				1
17	1.4806	0.6881	0.0511	0.8128	1	Q03147	Cyclin-dependent kinase 7	Cdk7	mmu:12572	1				
18	0.4041	0.5237	0.4620	0.8235	1	Q99KH8	Serine/threonine-protein kinase 24;Serine/threonine-protein kinase 24 35 kDa subunit;Serine/thr	Stk24	mmu:223255	1	1			
19	1.5061	0.4959	0.8978	1.0519	1	Q4JIM5	Non-specific protein-tyrosine kinase;Abelson tyrosine-protein kinase 2	Abl2		1				
20	-1.2418	-3.6384	-1.4498	-2.5566	1	P18155	Bifunctional methylenetetrahydrofolate dehydrogenase/cyclohydrolase, mitochondrial;NAD-depe	Mthfd2	mmu:17768					
21	-0.0154	-0.0144	-1.8127	-1.8221	1	P63166	Small ubiquitin-related modifier 1	Sumo1	mmu:22218					
22	-0.8880	-0.9121	-1.7116	-1.6991	1	O70585	Dystrobrevin;Dystrobrevin beta	Dtnb	mmu:13528					
23	-0.7734	-1.7908	-0.3951	-1.6011	1	Q8CGP6	Histone H2A type 1-H;Histone H2A,J;Histone H2A type 1-K;Histone H2A type 1-F;Histone H2A type	Hist1h2ah;	mmu:319168					
24	-0.1035	-0.1703	-0.8107	-1.5152	1	Q6QD59	Vesicle transport protein SEC20	Bnip1	mmu:224630					1
25	-0.3524	-1.8097	-2.8079	-1.4653	1	Q6PGF3	Mediator of RNA polymerase II transcription subunit 16	Med16						
26	-1.7944	-1.8816	-0.8445	-1.4602	1	Q2VPQ9	Chromatin modification-related protein MEAF6	Meaf6	mmu:70088					
27	-1.3940	-0.3397	-2.9703	-1.3263	1	Q8BW00	Probable peptidyl-tRNA hydrolase	Ptrh1	mmu:329384					
28	-0.4172	-0.7460	-1.2441	-1.2318	1	Q9Z0P5	Twinfilin-2	Twf2	mmu:23999					
29	-0.3208	-0.9051	-1.8610	-1.2150	1	O88986	2-amino-3-ketobutyrate coenzyme A ligase, mitochondrial	Gcat	mmu:26912					
30	-1.6343	-1.8991	-0.1736	-1.2088	1	Q08509	Epidermal growth factor receptor kinase substrate 8	Eps8	mmu:13860					
31	-0.2595	-0.2641	-2.5339	-1.2085	1	Q9CQV5	28S ribosomal protein S24, mitochondrial	Mrps24	mmu:64660					
32	-1.1228	-0.6805	-0.6200	-1.1477	1	Q9D6J6	NADH dehydrogenase [ubiquinone] flavoprotein 2, mitochondrial	Ndufv2	mmu:72900					
33	-0.4339	-1.8482	-0.5800	-1.1326	1	Q60870	Receptor expression-enhancing protein	Reep5						
34	-0.9002	-0.1455	-0.9085	-1.0747	1	Q9D338	39S ribosomal protein L19, mitochondrial	Mrpl19	mmu:56284					
35	-0.4515	-0.7921	-2.9000	-1.0724	1	O89017	Legumain	Lgmn	mmu:19141					
36	-2.1760	-1.4607	-0.3223	-1.0707	1	P62309	Small nuclear ribonucleoprotein G	Snrpg	mmu:68011					
37	-0.3868	-0.2433	-2.5326	-1.0590	1	B1AZI6	THO complex subunit 2	Thoc2	mmu:331401					
38	-2.1888	-0.6123	-0.3683	-1.0183	1	Q9ES28	Rho guanine nucleotide exchange factor 7	Arhgef7	mmu:54126					
39	-0.7856	-0.3085	-0.9559	-0.9398	1	Q9DC23	DnaJ homolog subfamily C member 10	Dnajc10	mmu:66861					
40	-0.0940	-0.9203	-0.9162	-0.9165	1	Q8VI33	Transcription initiation factor TFIID subunit 9	Taf9	mmu:108143					
41	-0.8967	-0.0966	-0.0410	-0.9062	1	Q64433	10 kDa heat shock protein, mitochondrial	Hspe1	mmu:15528					1
42	-0.4717	-0.6820	-0.0698	-0.8912	1	G3UWQ7		Prc1						
43	-0.2651	-0.9754	-0.5757	-0.8793	1	Q8CI11	Guanine nucleotide-binding protein-like 3	Gnl3	mmu:30877					
44	-0.2029	-0.1194	-1.3544	-0.8784	1	P48962	ADP/ATP translocase 1	Slc25a4	mmu:11739					
45	-0.5638	-0.9832	-1.0664	-0.8669	1	Q7TQK1	Integrator complex subunit 7	Ints7	mmu:77065					
46	-0.1893	-1.0326	-0.4387	-0.8546	1	Q60932	Voltage-dependent anion-selective channel protein 1	Vdac1	mmu:22333					
47	-1.0699	-0.1522	-0.4572	-0.8510	1	P61750	ADP-ribosylation factor 4	Arf4	mmu:11843					
48	-0.1131	-0.8918	-0.6164	-0.8487	1	E9Q4P1		Wdfy1	mmu:69368					
49	-0.5191	-0.1387	-0.3482	-0.8348	1	Q6NZL1		Dhx37	mmu:208144					
50	-0.2446	-0.0115	-2.0330	-0.8341	1	Q8R086	Sulfite oxidase, mitochondrial	Suox	mmu:211389					
51	-0.0335	-0.6481	-1.9314	-0.8320	1	E9Q7G0		Numa1	mmu:101706					
52	-0.7021	-0.1285	-0.5701	-0.8063	1	P57759	Endoplasmic reticulum resident protein 29	Erp29	mmu:67397					
53	-0.1933	-0.5966	-0.1730	-0.8056	1	Q05793	Basement membrane-specific heparan sulfate proteoglycan core protein;Endorepellin;LG3 peptid	Hspg2	mmu:15530					
54	-1.7207	-0.3241	-0.7494	-0.7866	1	P61164	Alpha-centractin	Actr1a	mmu:54130					
55	-1.3803	-0.1267	-0.6762	-0.7739	1	Q9CQY6	Ubiquinol-cytochrome-c reductase complex assembly factor 2	Uqcc2	mmu:67267					
56	[illegible]	-1.0046	-1.4500	-0.7661	1	Q91W67	Ubiquitin-like protein 7	Ubl7	mmu:69459					
57	-0.3061	-0.5293	-0.3057	-0.7624	1	Q9CQ60	6-phosphogluconolactonase	Pgls	mmu:66171					
58	-0.0689	-0.0480	-0.0656	-0.7571	1	Q99N87	28S ribosomal protein S5, mitochondrial	Mrps5	mmu:77721					
59	-0.1224	-0.2233	-1.4632	-0.7486	1	Q8VDS4	Regulation of nuclear pre-mRNA domain-containing protein 1A	Rprd1a	mmu:225283					
60	-0.3738	-0.6530	-0.6301	-0.7166	1	Q6P069	Sorcin	Sri	mmu:109552					
61	-0.1729	-1.2203	-0.6358	-0.6955	1	Q9CPP6	NADH dehydrogenase [ubiquinone] 1 alpha subcomplex subunit 5	Ndufa5						
62	-0.0444	-0.4393	-0.8092	-0.6939	1	D3Z7P3	Glutaminase kidney isoform, mitochondrial	Gls	mmu:14660					
63	-0.1960	-0.1860	-1.4865	-0.6896	1	Q6PD19	UPF0668 protein C10orf76 homolog		mmu:71617					
64	-0.2531	-0.7270	-0.6143	-0.6863	1	Q8R0H9	ADP-ribosylation factor-binding protein GGA1	Gga1	mmu:106039					
65	-0.1140	-0.2776	-0.3778	-0.6850	1	Q63943	Myocyte-specific enhancer factor 2D	Mef2d	mmu:17261					
66	-0.8896	-0.6122	-0.3576	-0.6697	1	Q91VA6	Polymerase delta-interacting protein 2	Poldip2	mmu:67811					
67	-0.9178	-0.0410	-0.0744	-0.6667	1	Q9DB73	NADH-cytochrome b5 reductase 1	Cyb5r1	mmu:72017					
68	-0.1569	-0.1422	-0.4170	-0.6624	1	Q9D7B6	Isobutyryl-CoA dehydrogenase, mitochondrial	Acad8	mmu:66948					
69	-0.1346	-0.0330	-0.8531	-0.6617	1	Q9D0D4	Probable dimethyladenosine transferase	Dimt1	mmu:66254					
70	-0.5443	-0.3470	-0.6342	-0.6481	1	Q56A08	G patch domain and KOW motifs-containing protein	Gpkow	mmu:209416					
71	-1.3672	-0.3049	-0.2688	-0.6450	1	Q8BTX9	Inactive hydroxysteroid dehydrogenase-like protein 1	Hsdl1	mmu:72552					
72	-0.2952	-0.7705	-1.4308	-0.6436	1	Q8K1Z0	Ubiquinone biosynthesis protein COQ9, mitochondrial	Coq9	mmu:67914					
73	-0.3911	-0.2657	-0.5276	-0.6383	1	Q99LI7	Cleavage stimulation factor subunit 3	Cstf3	mmu:228410					
74	-0.7399	-0.3325	-0.9429	-0.6346	1	Q921G7	Electron transfer flavoprotein-ubiquinone oxidoreductase, mitochondrial	Etfdh	mmu:66841		1			
75	-0.3031	-0.9252	-1.1182	-0.6330	1	Q61595		Ktn1	mmu:16709					
76	-0.9400	-0.1885	-0.3206	-0.6311	1	P97461	40S ribosomal protein S5;40S ribosomal protein S5, N-terminally processed	Rps5	mmu:20103					
77	-0.7888	-0.0695	-0.7763	-0.6248	1	Q9CW46	Ribonucleoprotein PTB-binding 1	Raver1	mmu:71766					
78	-0.4569	-0.5244	-1.2479	-0.6117	1	Q9CWE0	Mitochondrial fission regulator 1-like	Mtfr1l	mmu:76824					
79	-0.2565	-0.7439	-1.1113	-0.6065	1	Q9DB15	39S ribosomal protein L12, mitochondrial	Mrpl12	mmu:56282					
80	-0.4023	-1.5767	-0.2783	-0.6061	1	Q8BJZ4	28S ribosomal protein S35, mitochondrial	Mrps35	mmu:232536					
81	-0.1012	-0.2471	-0.3808	-0.5985	1	Q9R0E2	Procollagen-lysine,2-oxoglutarate 5-dioxygenase 1	Plod1	mmu:18822					
82	-0.6063	-0.2692	-0.0580	-0.5904	1	Q9CPZ8	COX assembly mitochondrial protein homolog	Cmc1	mmu:67899					
83	-0.3716	-0.8381	-0.0145	-0.5798	1	Q6NV99		Haus6	mmu:230376					
84	-0.1328	-0.6864	-1.4146	-0.5796	1	Q9WVF7	DNA polymerase epsilon catalytic subunit A	Pole	mmu:18973					
85	-0.4794	[illegible]	-0.0903	-0.5758	1	P12023	Amyloid beta A4 protein;N-APP;Soluble APP-alpha;Soluble APP-beta;C99;Beta-amyloid protein 42	App	mmu:11820		1			
86	-0.0723	-0.1886	-0.0598	-0.5633	1	P53564	Homeobox protein cut-like;Protein CASP	Cux1	mmu:13047					
87	-0.4624	-1.1236	-0.5468	-0.5586	1	O88712	C-terminal-binding protein 1	Ctbp1	mmu:13016					
88	-0.6935	-0.2283	-1.1386	-0.5489	1	P51807	Dynein light chain Tctex-type 1	Dynlt1	mmu:21648					
89	-0.8148	-0.1199	-0.2863	-0.5422	1	Q99JY0	Trifunctional enzyme subunit beta, mitochondrial;3-ketoacyl-CoA thiolase	Hadhb	mmu:231086					
90	-0.8771	-0.3920	-0.1285	-0.5391	1	Q91XX1		Pcdhgc3	mmu:93706					
91	-0.2759	-0.8732	-0.6075	-0.5303	1	P47738	Aldehyde dehydrogenase, mitochondrial	Aldh2	mmu:11669					
92	-0.4336	-0.0743	-0.2847	-0.5252	1	Q9D6R2	Isocitrate dehydrogenase [NAD] subunit alpha, mitochondrial	Idh3a	mmu:67834					
93	-0.2061	-1.4561	-0.3425	-0.5241	1	Q60749	KH domain-containing, RNA-binding, signal transduction-associated protein 1	Khdrbs1	mmu:20218					
94	-0.3172	-0.3149	-0.2149	-0.5142	1	Q921L5	Conserved oligomeric Golgi complex subunit 2	Cog2	mmu:76332					
95	-0.0920	-0.2263	-0.6662	-0.5128	1	P21279	Guanine nucleotide-binding protein G(q) subunit alpha	Gnaq	mmu:14682					
96	-0.0742	-0.3326	-1.3466	-0.5108	1	P17426	AP-2 complex subunit alpha-1	Ap2a1	mmu:11771					
97	-0.3163	-0.6315	-0.9811	-0.5000	1	Q91YN9	BAG family molecular chaperone regulator 2	Bag2	mmu:213539		1			
98	-0.2781	-0.4801	-0.4836	-0.4996	1	Q6NZN0	RNA-binding protein 26	Rbm26	mmu:74213					
99	-0.5612	-0.8573	-0.1415	-0.4931	1	Q63918	Serum deprivation-response protein	Sdpr	mmu:20324					
100	-0.3380	-0.3703	-0.9128	-0.4875	1	Q8C3S2	Transport and Golgi organization protein 6 homolog	Tango6	mmu:272538					
101	-0.6781	-0.5121	-0.2456	-0.4743	1	Q61810	Latent-transforming growth factor beta-binding protein 3	Ltbp3	mmu:16998					
102	-0.1935	-0.1959	-0.0159	-0.4583	1	Q91V12	Cytosolic acyl coenzyme A thioester hydrolase	Acot7	mmu:70025					
103	-1.1248	-0.2611	-0.0422	-0.4519	1	Q91VJ2	Protein kinase C delta-binding protein	Prkcdbp	mmu:109042					
104	-0.4187	-0.4944	-0.5288	-0.4486	1	P62915	Transcription initiation factor IIB	Gtf2b	mmu:229906					
105	-0.4157	-0.5429	-0.3373	-0.4480	1	Q9DBL1	Short/branched chain specific acyl-CoA dehydrogenase, mitochondrial	Acadsb	mmu:66885					
106	-0.4939	-0.5429	-0.3823	-0.4453	1	Q6PB66	Leucine-rich PPR motif-containing protein, mitochondrial	Lrpprc	mmu:72416					
107	-0.4692	-0.3220	-0.3917	-0.4428	1	Q91YR1	Twinfilin-1	Twf1	mmu:19230					
108	-0.7106	-0.4653	-0.4703	-0.4406	1	Q7M6Y3	Phosphatidylinositol-binding clathrin assembly protein	Picalm	mmu:233489					
109	-0.2546	-0.5238	-0.6521	-0.4370	1	P67778	Prohibitin	Phb	mmu:18673					
110	-0.2442	-0.3321	-0.9481	-0.4356	1	Q9CPR8	Melanoma-associated antigen G1	Ndnl2;170(	mmu:66647					
111	-0.0354	-0.0851	[illegible]	-0.4301	1	Q64727	Vinculin	Vcl	mmu:22330					
112	-0.3671	-0.0862	-0.4928	-0.4291	1	Q64378	Peptidyl-prolyl cis-trans isomerase FKBP5;Peptidyl-prolyl cis-trans isomerase FKBP5, N-terminally p	Fkbp5	mmu:14229					
113	-0.6801	-0.6945	-0.1175	-0.4257	1	P35822	Receptor-type tyrosine-protein phosphatase kappa	Ptprk	mmu:19272		1	1		
114	-0.4343	-0.1637	-0.7220	-0.4224	1	Q99LZ3	DNA replication complex GINS protein SLD5;DNA replication complex GINS protein SLD5, N-termin	Gins4	mmu:109145					
115	-0.7920	-0.0820	-0.5181	-0.4216	1	Q3UKJ7	WD40 repeat-containing protein SMU1;WD40 repeat-containing protein SMU1, N-terminally proc	Smu1	mmu:74255					

116	-0.1468	-0.0320	-0.7065	-0.4185	1	Q8VE22	28S ribosomal protein S23, mitochondrial	Mrps23	mmu:64656				
117	-0.4058	-0.4831	-0.0306	-0.4122	1	Q61112	45 kDa calcium-binding protein	Sdf4	mmu:20318				
118	-0.2854	-0.1275	-0.9671	-0.4109	1	Q61687	Transcriptional regulator ATRX	Atrx	mmu:22589				
119	-0.0279	-0.4594	-0.3535	-0.4010	1	Q9JKR6	Hypoxia up-regulated protein 1	Hyou1	mmu:12282				
120	-0.5297	-0.3658	-0.2262	-0.3910	1	P48754	Breast cancer type 1 susceptibility protein homolog	Brca1	mmu:12189			1	1
121	-0.4841	-0.1750	-0.3766	-0.3835	1	O08528	Hexokinase;Hexokinase-2	Hk2	mmu:15277				
122	-0.2911	-0.0710	-0.3056	-0.3825	1	Q8VHR5	Transcriptional repressor p66-beta	Gatad2b	mmu:229542				
123	-0.1659	-0.0401	-0.1776	-0.3797	1	Q7TQI3	Ubiquitin thioesterase OTUB1	Otub1	mmu:107260				
124	-0.7196	-0.2776	-0.2012	-0.3759	1	Q9Z2U1	Proteasome subunit alpha type;Proteasome subunit alpha type-5	Gm8394;P	mmu:26442				
125	-0.1401	-0.6566	-0.2564	-0.3735	1	Q9JHK4	Geranylgeranyl transferase type-2 subunit alpha	Rabggta	mmu:56187				
126	-0.3678	-0.4243	-0.4214	-0.3711	1	Q14CH7	Alanine--tRNA ligase, mitochondrial	Aars2	mmu:224805				
127	-0.3625	-0.3932	-0.4787	-0.3599	1	P70404	Isocitrate dehydrogenase [NAD] subunit gamma 1, mitochondrial	Idh3g	mmu:15929				
128	-0.2242	-0.4157	-0.2774	-0.3565	1	O54724	Polymerase I and transcript release factor	Ptrf	mmu:19285				
129	-0.0332	-0.1784	-0.0371	-0.3503	1	Q9CQ36	DNA polymerase epsilon subunit 4	Pole4	mmu:66979				
130	-0.1417	-0.3225	-0.2912	-0.3469	1	Q8BJ71	Nuclear pore complex protein Nup93	Nup93	mmu:71805				
131	-0.7525	-0.1256	-0.4337	-0.3433	1	Q8BMD8	Calcium-binding mitochondrial carrier protein SCaMC-1	Slc25a24	mmu:229731	1			
132	-0.3723	-0.1713	-0.1582	-0.3428	1	Q80WQ2	Protein VAC14 homolog	Vac14	mmu:234729				
133	-0.4840	-0.4099	-0.2801	-0.3412	1	Q9CYN9	Renin receptor	Atp6ap2	mmu:70495				
134	-0.3699	-0.2534	-0.1521	-0.3231	1	Q99KK9	Probable histidine--tRNA ligase, mitochondrial	Hars2	mmu:70791				
135	-0.2191	-0.9102	-0.1077	-0.3217	1	P33610	DNA primase large subunit	Prim2	mmu:19076				
136	-0.1876	-0.0407	-0.5247	-0.3157	1	O89116	Vesicle transport through interaction with t-SNAREs homolog 1A	Vti1a	mmu:53611				
137	-0.1632	-0.4735	-0.2998	-0.3103	1	Q9ER88	28S ribosomal protein S29, mitochondrial	Dap3	mmu:65111				1
138	-0.0425	-0.1583	-0.2471	-0.2946	1	Q99KX1	Myeloid leukemia factor 2	Mlf2	mmu:30853				
139	-0.0793	-0.0965	-0.2103	-0.2903	1	Q9QYI4	DnaJ homolog subfamily B member 12	Dnajb12	mmu:56709				
140	-0.1587	-0.1567	-0.4086	-0.2802	1	Q99LB2	Dehydrogenase/reductase SDR family member 4	Dhrs4	mmu:28200				
141	-0.1499	-0.1537	-0.1032	-0.2748	1	Q64514	Tripeptidyl-peptidase 2	Tpp2	mmu:22019				
142	-0.0807	-0.3764	-0.1145	-0.2659	1	Q8BR07	Protein bicaudal D homolog 1	Bicd1	mmu:12121				
143	-0.5990	-0.2289	-0.1264	-0.2581	1	Q9Z1Q5	Chloride intracellular channel protein 1	Clic1	mmu:114584				
144	-0.1017	-0.5433	-0.0857	-0.2570	1	Q8R317	Ubiquilin-1	Ubqln1	mmu:56085				
145	-0.3235	-0.0387	-0.3598	-0.2528	1	Q99L13	3-hydroxyisobutyrate dehydrogenase, mitochondrial	Hibadh	mmu:58875				
146	-0.2239	-0.1307	-0.1904	-0.2490	1	P97868	E3 ubiquitin-protein ligase RBBP6	Rbbp6	mmu:19647			1	
147	-0.4556	-0.3287	-0.1374	-0.2476	1	Q9JJG9	Nitric oxide-associated protein 1	Noa1	mmu:56412				
148	-0.3628	-0.1484	-0.2318	-0.2239	1	E9Q8D0		Dnajc21	mmu:78244				
149	-0.1070	-0.0961	-0.4161	-0.2222	1	Q5SXY1	Cytospin-B	Specc1					
150	-0.1756	-0.1109	-0.3297	-0.2221	1	O35465	Peptidyl-prolyl cis-trans isomerase FKBP8	Fkbp8	mmu:14232				
151	-0.0233	-0.0538	-0.7183	-0.2220	1	Q7TMY8	E3 ubiquitin-protein ligase HUWE1	Huwe1	mmu:59026			1	
152	-0.0226	-0.2692	-0.1439	-0.2087	1	Q8CC88	von Willebrand factor A domain-containing protein 8	Vwa8	mmu:219189				
153	-0.1201	-0.2430	-0.1476	-0.2072	1	Q60875	Rho guanine nucleotide exchange factor 2	Arhgef2	mmu:16800				
154	-0.3924	-0.1755	[illegible]	-0.2012	1	Q8BNI4	Derlin-2	Derl2	mmu:116891				
155	-0.0968	-0.2084	-0.2869	-0.2004	1	Q9Z110	Delta-1-pyrroline-5-carboxylate synthase;Glutamate 5-kinase;Gamma-glutamyl phosphate reducta	Aldh18a1	mmu:56454				
156	[illegible]	-0.1279	-0.2813	-0.1963	1	Q8K2Y7	39S ribosomal protein L47, mitochondrial	Mrpl47	mmu:74600				
157	-0.1022	-0.0299	-0.0213	-0.1879	1	P02468	Laminin subunit gamma-1	Lamc1					
158	-0.0556	-0.2927	-0.1443	-0.1650	1	Q3U9G9	Lamin-B receptor	Lbr	mmu:98386				
159	-0.1015	-0.1427	-0.2338	-0.1634	1	P08775	DNA-directed RNA polymerase II subunit RPB1	Polr2a	mmu:20020				
160	-0.0890	-0.0193	-0.2303	-0.1607	1	P54729	NEDD8 ultimate buster 1	Nub1	mmu:53312				
161	-0.1074	-0.0618	-0.3513	-0.1575	1	E9Q6R7		Utrn	mmu:22288				
162	-0.0272	-0.0511	-0.1365	-0.1497	1	P32067	Lupus La protein homolog	Ssb	mmu:20823				
163	-0.0759	-0.0367	-0.0280	-0.1490	1	Q80VJ3	2-deoxynucleoside 5-phosphate N-hydrolase 1	Dnph1	mmu:381101				
164	-0.2048	-0.0399	-0.1332	-0.1474	1	Q3TWF6	WD repeat-containing protein 70	Wdr70					
165	-0.1189	-0.0118	-0.0732	-0.1446	1	Q99MD9	Nuclear autoantigenic sperm protein	Nasp	mmu:50927				
166	[illegible]	-0.0774	-0.0137	-0.0572	1	Q9JKF1	Ras GTPase-activating-like protein IQGAP1	Iqgap1	mmu:29875				
167	0.0664	0.1284	0.0720	0.0857	1	P63024	Vesicle-associated membrane protein 3	Vamp3	mmu:22319				
168	0.0543	0.0836	0.2700	0.1056	1	Q5FWK3	Rho GTPase-activating protein 1	Arhgap1	mmu:228359				
169	0.1315	0.1322	0.1315	0.1079	1	Q9ESX5	H/ACA ribonucleoprotein complex subunit 4	Dkc1	mmu:245474				
170	0.2955	0.0202	0.0223	0.1099	1	Q7TSG2	RNA polymerase II subunit A C-terminal domain phosphatase	Ctdp1	mmu:67655		1		
171	0.2415	0.0477	0.1670	0.1141	1	Q9QXZ0	Microtubule-actin cross-linking factor 1	Macf1					
172	0.0579	0.1142	0.2670	0.1245	1	Q6PGL7	WASH complex subunit FAM21	Fam21	mmu:28006				
173	0.0859	0.2675	0.1246	0.1343	1	Q3TLD5	Unconventional prefoldin RPB5 interactor	Uri1	mmu:19777				
174	0.1709	0.1996	0.0638	0.1363	1	P33609	DNA polymerase alpha catalytic subunit	Pola1	mmu:18968				
175	0.1580	0.1075	0.3029	0.1424	1	Q9R233	Tapasin	Tapbp	mmu:21356				
176	0.1153	0.3082	0.0652	0.1447	1	Q7TPR4	Alpha-actinin-1	Actn1	mmu:109711				
177	0.2906	0.0150	[illegible]	0.1507	1	P16332	Methylmalonyl-CoA mutase, mitochondrial	Mut	mmu:17850				
178	0.1441	0.0354	0.1008	0.1513	1	Q9ES00	Ubiquitin conjugation factor E4 B	Ube4b	mmu:63958			1	
179	0.1571	0.1502	0.1504	0.1539	1	P20029	78 kDa glucose-regulated protein	Hspa5	mmu:14828				
180	0.1717	0.0663	0.2858	0.1614	1	Q8CGZ0	Calcium homeostasis endoplasmic reticulum protein	Cherp	mmu:27967				
181	0.1399	0.1719	0.0887	0.1620	1	P59325	Eukaryotic translation initiation factor 5	Eif5	mmu:217869				
182	0.1607	0.1452	0.0160	0.1623	1	P36916	Guanine nucleotide-binding protein-like 1	Gnl1	mmu:14670				
183	0.1659	0.0489	0.1794	0.1673	1	O54931	A-kinase anchor protein 2	Akap2	mmu:11641				
184	0.0277	0.1121	[illegible]	0.1682	1	Q9CQ22	Ragulator complex protein LAMTOR1	Lamtor1	mmu:66508				
185	0.0694	0.0187	0.0755	0.1740	1	P46471	26S protease regulatory subunit 7	Psmc2					
186	0.0553	0.2857	0.2694	0.1779	1	O35654	DNA polymerase delta subunit 2	Pold2	mmu:18972				
187	0.1179	0.2846	0.3158	0.1844	1	Q8K1J5	Protein SDE2 homolog	Sde2	mmu:208768				
188	0.1214	0.0295	0.0325	0.1870	1	P28658	Ataxin-10	Atxn10	mmu:54138				
189	0.0659	0.3562	0.1542	0.1880	1	Q9D706	RNA polymerase II-associated protein 3	Rpap3	mmu:71919				
190	0.1779	0.4755	[illegible]	0.1941	1	Q9WTX5	S-phase kinase-associated protein 1	Skp1	mmu:21402			1	
191	0.1578	0.3525	0.2251	0.1942	1	Q9R1C7	Pre-mRNA-processing factor 40 homolog A	Prpf40a	mmu:56194				
192	0.1832	[illegible]	0.0821	0.1950	1	Q11011	Puromycin-sensitive aminopeptidase	Npepps	mmu:19155				
193	0.0355	0.3008	0.0937	0.2008	1	P59328	WD repeat and HMG-box DNA-binding protein 1	Wdhd1	mmu:218973				
194	0.3096	0.1584	0.0709	0.2039	1	P62259	14-3-3 protein epsilon	Ywhae	mmu:22627				
195	0.1655	0.5722	0.0867	0.2098	1	Q61249	Immunoglobulin-binding protein 1	Igbp1	mmu:18518				
196	0.1378	0.2313	0.0699	0.2110	1	Q8CIE6	Coatomer subunit alpha;Coatomer subunit alpha;Xenin;Proxenin	Copa	mmu:12847				
197	0.1814	0.4764	0.1752	0.2131	1	P42208	Septin-2	Sept2	mmu:18000				
198	0.1683	0.0649	0.4548	0.2164	1	P80315	T-complex protein 1 subunit delta	Cct4	mmu:12464				
199	0.2335	0.0149	0.4031	0.2179	1	Q9Z1Z0	General vesicular transport factor p115	Uso1	mmu:56041				
200	0.1962	0.1895	0.1913	0.2179	1	Q9R190	Metastasis-associated protein MTA2	Mta2	mmu:23942				
201	[illegible]	0.0720	0.0830	0.2197	1	P46638	Ras-related protein Rab-11B	Rab11b;Ra	mmu:19326				
202	0.0725	0.2854	0.0986	0.2200	1	Q6IRU2	Tropomyosin alpha-4 chain	Tpm4	mmu:326618				
203	0.3143	0.1155	0.0555	0.2233	1	Q8C7X2	ER membrane protein complex subunit 1	Emc1	mmu:230866				
204	0.2849	0.0260	0.2547	0.2242	1	Q9EQH3	Vacuolar protein sorting-associated protein 35	Vps35	mmu:65114				
205	0.1751	0.2321	0.4842	0.2246	1	Q9DBC7	cAMP-dependent protein kinase type I-alpha regulatory subunit;cAMP-dependent protein kinase	Prkar1a	mmu:19084				
206	0.1815	0.1244	0.4701	0.2255	1	Q9CPR5	39S ribosomal protein L15, mitochondrial	Mrpl15	mmu:27395				
207	0.0374	0.7769	0.0675	0.2259	1	Q61686	Chromobox protein homolog 5	Cbx5	mmu:12419				
208	0.3422	0.0475	0.0923	0.2270	1	P62876	DNA-directed RNA polymerases I, II, and III subunit RPABC5	Usp50;Polr	mmu:66491				
209	0.2318	0.4274	0.2043	0.2275	1	Q3THK3	General transcription factor IIF subunit 1	Gtf2f1	mmu:98053				
210	0.2730	0.0694	0.2651	0.2310	1	P61161	Actin-related protein 2	Actr2	mmu:66713				
211	0.5542	0.1823	0.0102	0.2320	1	P56399	Ubiquitin carboxyl-terminal hydrolase;Ubiquitin carboxyl-terminal hydrolase 5	Usp5	mmu:22225				
212	0.5899	0.0228	0.3212	0.2355	1	O35685	Nuclear migration protein nudC	Nudc	mmu:18221				
213	0.2377	0.0440	0.2924	0.2357	1	Q99K01	Pyridoxal-dependent decarboxylase domain-containing protein 1	Pdxdc1	mmu:94184				
214	0.3796	0.0091	0.5423	0.2375	1	Q924Z6	Exportin-6	Xpo6	mmu:74204				
215	0.1179	0.0855	0.2166	0.2375	1	P63101	14-3-3 protein zeta/delta	Ywhaz	mmu:22631				1
216	0.0706	0.2885	0.4753	0.2378	1	Q9DCH4	Eukaryotic translation initiation factor 3 subunit F	Eif3f	mmu:66085				
217	0.3118	0.2738	0.2158	0.2416	1	Q9DBG3	AP-2 complex subunit beta;AP complex subunit beta	Ap2b1	mmu:71770				
218	0.1598	0.0621	0.1897	0.2437	1	P46935	E3 ubiquitin-protein ligase NEDD4	Nedd4	mmu:17999			1	
219	0.0974	0.5129	0.1280	0.2469	1	Q5DTT8	Paraneoplastic antigen-like protein 5	Pnma5	mmu:385377				
220	0.3688	0.1243	0.3924	0.2476	1	Q61768	Kinesin-1 heavy chain;Kinesin-like protein	Kif5b	mmu:16573				
221	0.4951	0.1832	0.1816	0.2498	1	Q62384	Zinc finger protein ZPR1	Zpr1	mmu:22687				
222	0.0216	0.0887	0.4820	0.2499	1	Q9WVJ2	26S proteasome non-ATPase regulatory subunit 13	Psmd13	mmu:23997				
223	0.3711	0.2526	0.1571	0.2567	1	Q01853	Transitional endoplasmic reticulum ATPase	Vcp	mmu:269523				1
224	0.3341	0.0147	0.4638	0.2572	1	Q9CWK8	Sorting nexin-2	Snx2	mmu:67804				
225	0.4524	0.0134	0.3347	0.2580	1	Q61699	Heat shock protein 105 kDa	Hsph1	mmu:15505				
226	0.1942	0.0275	0.5896	0.2614	1	P42225	Signal transducer and activator of transcription;Signal transducer and activator of transcription 1	Stat1					1
227	0.2247	0.1026	0.1015	0.2626	1	Q91W92	Cdc42 effector protein 1	Cdc42ep1	mmu:104445				
228	0.3625	0.4209	0.2417	0.2629	1	Q9D6J3	Coiled-coil domain-containing protein 94	Ccdc94	mmu:72886				
229	0.0737	0.1962	0.1659	0.2686	1	Q8VIM9	Immunity-related GTPase family Q protein	Irgq	mmu:210146				
230	0.0537	0.3254	0.1254	0.2690	1	Q9EP71	Ankycorbin	Rai14	mmu:75646				
231	0.2687	0.1874	0.4566	0.2697	1	Q9WUM3	Coronin-1B	Coro1b	mmu:23789				
232	[illegible]	[illegible]	0.2970	0.2701	1	Q9Z0N1	Eukaryotic translation initiation factor 2 subunit 3, X-linked;Eukaryotic translation initiation factor	Eif2s3x;Eif2	mmu:26905				
233	0.1842	0.2030	0.3436	0.2773	1	Q3V0C5	Ubiquitin carboxyl-terminal hydrolase 48	Usp48	mmu:170707				
234	0.1571	0.2987	0.4038	0.2790	1	Q62159	Rho-related GTP-binding protein RhoC	Rhoc	mmu:11853				
235	0.1750	0.0680	0.1953	0.2801	1	P60229	Eukaryotic translation initiation factor 3 subunit E	Eif3e	mmu:16341				
236	0.2048	0.2610	0.3117	0.2841	1	Q8BIW1	Protein prune homolog	Prune	mmu:229589				
237	0.2333	0.2158	0.0731	0.2858	1	P57780	Alpha-actinin-4	Actn4	mmu:60595				
238	0.3278	0.5070	0.0118	0.2868	1	Q9ES83		Pop1	mmu:23828				
239	0.0665	0.3410	0.2047	0.2912	1	Q9EPN1	Neurobeachin	Nbea	mmu:26422				
240	0.2886	0.1920	0.4427	0.2912	1	P70698	CTP synthase 1	Ctps1	mmu:51797				
241	0.8687	0.0582	0.0931	0.2938	1	Q8BYJ6	TBC1 domain family member 4	Tbc1d4	mmu:210789				

242	0.4511	0.0635	0.2109	0.2960	1	Q8BY87	Ubiquitin carboxyl-terminal hydrolase 47	Usp47	mmu:74996				
243	0.0717	0.7849	0.1408	0.2969	1	Q8CH18	Cell division cycle and apoptosis regulator protein 1	Ccar1	mmu:67500				
244	[illegible]	0.5542	0.1897	0.2978	1	Q6PIU9	Uncharacterized protein FLJ45252 homolog		mmu:102637099				
245	0.2421	0.4243	0.0657	0.3003	1	Q8CHQ0	F-box only protein 4	Fbxo4	mmu:106052			1	
246	0.4947	0.1374	0.0534	0.3041	1	Q9EQC8		Prcc	mmu:94315				
247	0.0878	1.0262	0.0580	0.3066	1	Q6PFR5	Transformer-2 protein homolog alpha	Tra2a					
248	0.4367	0.1668	0.2723	0.3085	1	Q80UG5	Septin-9	Sept9	mmu:53860				
249	0.4224	0.6282	0.1152	0.3090	1	Q04887	Transcription factor SOX-9	Sox9	mmu:20682				
250	0.0659	0.5421	0.1953	0.3101	1	B1ARD6		Slfn9	mmu:237886				
251	0.4865	0.1928	0.2213	0.3106	1	Q921M4	Golgin subfamily A member 2	Golga2	mmu:99412				
252	0.5017	0.4210	0.2514	0.3136	1	P62137	Serine/threonine-protein phosphatase PP1-alpha catalytic subunit	Ppp1ca	mmu:19045		1		
253	0.3088	0.4016	0.1372	0.3153	1	Q9EPU0	Regulator of nonsense transcripts 1	Upf1	mmu:19704				
254	0.4233	0.0715	0.0308	0.3174	1	Q8BUK6	Protein Hook homolog 3	Hook3	mmu:320191				
255	0.0973	0.0698	0.4503	0.3183	1	P49443	Protein phosphatase 1A	Ppm1a	mmu:19042		1		
256	0.0658	0.1519	0.5567	0.3220	1	Q923E4	NAD-dependent protein deacetylase sirtuin-1;SirtT1 75 kDa fragment	Sirt1	mmu:93759	1			
257	[illegible]	0.5269	0.1377	0.3221	1	Q91Z38	Tetratricopeptide repeat protein 1	Ttc1	mmu:66827				
258	0.4048	0.1275	0.2562	0.3235	1	P58871	182 kDa tankyrase-1-binding protein	Tnks1bp1	mmu:228140				
259	0.3008	[illegible]	0.3413	0.3279	1	P63321	Ras-related protein Ral-A	Rala	mmu:56044				
260	0.4386	0.6495	0.0500	0.3300	1	Q9Z0U1	Tight junction protein ZO-2	Tjp2	mmu:21873				
261	0.6284	0.0700	0.1101	0.3392	1	Q9CT10	Ran-binding protein 3	Ranbp3	mmu:71810				
262	0.0615	0.2221	0.6104	0.3397	1	Q99KL7	Ras-related protein Rab-28	Rab28	mmu:100972				
263	0.1108	0.3700	0.7527	0.3404	1	Q9DBR7	Protein phosphatase 1 regulatory subunit 12A	Ppp1r12a	mmu:17931				
264	0.4552	0.1132	0.5105	0.3405	1	Q8CI94	Glycogen phosphorylase, brain form	Pygb	mmu:110078				
265	0.4868	0.0563	0.4090	0.3408	1	Q9CZ04	COP9 signalosome complex subunit 7a	Cops7a	mmu:26894				
266	0.8126	0.3051	0.2438	0.3489	1	Q69ZX6	MORC family CW-type zinc finger protein 2A	Morc2a	mmu:74522				
267	0.6668	0.3662	0.0412	0.3497	1	A2AQ19	RNA polymerase-associated protein RTF1 homolog	Rtf1	mmu:76246				
268	0.1069	0.1081	0.0267	0.3536	1	Q99MN1	Lysine--tRNA ligase	Kars	mmu:85305				
269	0.0586	0.2243	0.4069	0.3545	1	Q8BJU0	Small glutamine-rich tetratricopeptide repeat-containing protein alpha	Sgta	mmu:52551				
270	0.1997	0.3422	0.3776	0.3548	1	Q9CQV8	14-3-3 protein beta/alpha;14-3-3 protein beta/alpha, N-terminally processed	Ywhab	mmu:54401				
271	0.3144	0.0625	0.0314	0.3573	1	Q5SSL4	Active breakpoint cluster region-related protein	Abr	mmu:109934				
272	0.0495	0.5214	0.0289	0.3680	1	O35245	Polycystin-2	Pkd2	mmu:18764				
273	0.4858	0.2081	0.4593	0.3688	1	Q9CZ15	DNA replication complex GINS protein PSF1	Gins1	mmu:69270				
274	0.1583	0.2684	0.4364	0.3714	1	Q9CWM4	Prefoldin subunit 1	Pfdn1					
275	0.1256	0.4436	0.3788	0.3717	1	Q80X50	Ubiquitin-associated protein 2-like	Ubap2l	mmu:74383				
276	0.7869	0.1470	0.0558	0.3736	1	Q9DCL2	MIP18 family protein FAM96A	Fam96a	mmu:68250				
277	0.2191	0.5858	0.3210	0.3754	1	Q9Z266	SNARE-associated protein Snapin	Snapin	mmu:20615				
278	0.5829	0.0975	0.5298	0.3761	1	Q7TSS2	Ubiquitin-conjugating enzyme E2 Q1	Ube2q1	mmu:70093			1	
279	0.3227	[illegible]	0.0840	0.3786	1	Q9CX30	Protein YIF1B	Yif1b	mmu:77254				
280	0.2434	0.5337	0.2358	0.3803	1	Q8CDN6	Thioredoxin-like protein 1	Txnl1	mmu:53382	1			
281	[illegible]	0.5128	0.1095	0.3810	1	A2A8Z1	Oxysterol-binding protein;Oxysterol-binding protein-related protein 9	Osbpl9	mmu:100273				
282	0.5126	0.3942	0.3865	0.3817	1	Q99PG2	Opioid growth factor receptor	Ogfr	mmu:72075				
283	0.0593	0.0798	0.2609	0.3843	1	Q9QWF0	Chromatin assembly factor 1 subunit A	Chaf1a	mmu:27221				
284	0.0432	0.4020	0.4618	0.3844	1	Q3U7U3	F-box only protein 7	Fbxo7	mmu:69754			1	
285	0.1338	0.7091	0.2383	0.3846	1	Q61210	Rho guanine nucleotide exchange factor 1	Arhgef1	mmu:16801				
286	0.0660	0.3553	0.0089	0.3857	1	Q06185	ATP synthase subunit e, mitochondrial	Atp5i	mmu:11958				
287	0.7023	0.3141	0.4886	0.3877	1	E9Q7Q3		Tpm3					
288	0.5241	0.0994	0.1946	0.3896	1	Q9D8B3	Charged multivesicular body protein 4b	Chmp4b	mmu:75608				
289	0.0636	0.0564	0.6410	0.3896	1	Q6PER3	Microtubule-associated protein RP/EB family member 3	Mapre3	mmu:100732				
290	0.7009	0.2790	0.5156	0.3942	1	P68254	14-3-3 protein theta	Ywhaq	mmu:22630				
291	0.7132	0.4156	0.0232	0.3965	1	Q9D7M8	DNA-directed RNA polymerase II subunit RPB4	Polr2d	mmu:69241				
292	0.3281	0.1147	0.6545	0.4084	1	Q99PL5	Ribosome-binding protein 1	Rrbp1	mmu:81910				
293	0.7046	0.3506	0.0538	0.4112	1	Q8CHU3	Epsin-2	Epn2	mmu:13855				
294	0.3034	0.3624	0.7846	0.4118	1	O70194	Eukaryotic translation initiation factor 3 subunit D	Eif3d	mmu:55944				
295	0.3828	0.1046	0.6141	0.4157	1	Q60967	Bifunctional 3-phosphoadenosine 5-phosphosulfate synthase 1;Sulfate adenylyltransferase;Adenyl	Papss1	mmu:23971				
296	0.4875	0.0330	0.2526	0.4162	1	Q80YD1	ATP-dependent RNA helicase SUPV3L1, mitochondrial	Supv3l1	mmu:338359				
297	0.0580	0.1353	0.7596	0.4178	1	Q61696	Heat shock 70 kDa protein 1A;Heat shock 70 kDa protein 1B	Hspa1a;Hs	mmu:193740	1			
298	[illegible]	0.7646	0.2921	0.4216	1	Q3UY34	Uncharacterized protein C12orf43 homolog		mmu:72357				
299	0.7492	0.0511	0.4087	0.4233	1	Q80X73	Protein pelota homolog	Pelo	mmu:105083				
300	0.3017	0.1279	0.5874	0.4259	1	P47713	Cytosolic phospholipase A2;Phospholipase A2;Lysophospholipase	Pla2g4a	mmu:18783				
301	0.3618	0.2226	0.5308	0.4287	1	Q9DCD5	Tight junction-associated protein 1	Tjap1	mmu:74094				
302	0.4876	0.0653	0.6183	0.4330	1	Q9D8N0	Elongation factor 1-gamma	Eef1g	mmu:67160				
303	0.3511	0.7089	0.4994	0.4431	1	Q61166	Microtubule-associated protein RP/EB family member 1	Mapre1	mmu:13589				
304	0.1394	0.0237	0.5678	0.4431	1	P07901	Heat shock protein HSP 90-alpha	Hsp90aa1	mmu:15519	1			
305	0.0265	0.3885	0.5620	0.4434	1	Q05D44	Eukaryotic translation initiation factor 5B	Eif5b	mmu:226982				
306	0.2177	0.1399	0.4855	0.4482	1	P11499	Heat shock protein HSP 90-beta	Hsp90ab1	mmu:15516				
307	0.1577	0.0188	0.6545	0.4505	1	Q80YR7	Claspin	Clspn	mmu:269582				
308	0.7770	0.1044	0.0111	0.4509	1	P61982	14-3-3 protein gamma;14-3-3 protein gamma, N-terminally processed	Ywhag	mmu:22628				
309	0.2049	0.2872	0.2390	0.4514	1	Q8BMI0	F-box only protein 38	Fbxo38	mmu:107035				
310	0.8474	0.5491	0.2305	0.4545	1	Q3UGC7	Eukaryotic translation initiation factor 3 subunit J-A;Eukaryotic translation initiation factor 3 subur	Eif3j1;Eif3j	mmu:78655				
311	0.1919	0.2688	0.5012	0.4558	1	Q9CY18	Sorting nexin-7	Snx7					
312	0.4428	0.2265	0.4694	0.4599	1	Q80TH2	Protein LAP2	Erbb2ip	mmu:59079				
313	0.4957	0.5405	0.4174	0.4636	1	Q3UJD6	Ubiquitin carboxyl-terminal hydrolase;Ubiquitin carboxyl-terminal hydrolase 19	Usp19	mmu:71472				
314	0.1995	0.3857	0.0584	0.4711	1	P51949	CDK-activating kinase assembly factor MAT1	Mnat1	mmu:17420				
315	0.9709	0.2280	0.1786	0.4740	1	A0A0R4J205			mmu:108943				
316	0.6271	0.7749	0.3967	0.4747	1	Q8BHJ5	F-box-like/WD repeat-containing protein TBL1XR1	Tbl1xr1	mmu:81004				
317	0.3412	0.7171	0.1272	0.4752	1	Q9D0F6	Replication factor C subunit 5	Rfc5	mmu:72151				
318	0.0263	0.8864	0.1929	0.4782	1	Q6P3E7	Histone deacetylase 10	Hdac10	mmu:170787				
319	0.5256	0.4603	0.4715	0.4963	1	Q8C650	Septin-10	Sept10	mmu:103080				
320	0.0079	0.5525	0.8992	0.4969	1	Q5SVQ0	Histone acetyltransferase KAT7	Kat7	mmu:217127				
321	0.1714	[illegible]	0.0938	0.4975	1	E9Q368		Gm17296	mmu:212728				
322	0.0191	0.4119	1.1766	0.4984	1	O08553	Dihydropyrimidinase-related protein 2	Dpysl2	mmu:12934				
323	0.8056	0.6716	0.0736	0.4991	1	Q9D6K7	Tetratricopeptide repeat protein 33	Ttc33	mmu:67515				
324	0.2654	0.6811	0.4328	0.5025	1	Q3THJ3	Probable RNA-binding protein EIF1AD	Eif1ad	mmu:69860				
325	0.2862	0.3453	0.3276	0.5099	1	P59438	Hermansky-Pudlak syndrome 5 protein homolog	Hps5	mmu:246694				
326	0.7206	0.1222	0.6671	0.5203	1	Q9ERB0	Synaptosomal-associated protein 29	Snap29	mmu:67474				
327	0.0976	0.0439	0.6733	0.5270	1	Q5ND34	WD repeat-containing protein 81	Wdr81	mmu:192652				
328	0.3477	0.2778	0.1382	0.5326	1	P62984	Ubiquitin-60S ribosomal protein L40;Ubiquitin;60S ribosomal protein L40;Ubiquitin-40S ribosomal	Gm8797;U	mmu:22186				
329	0.0709	0.0493	0.8172	0.5330	1	Q80Y56	Rabenosyn-5	Rbsn	mmu:78287				
330	0.2584	0.5695	1.0257	0.5331	1	Q8CHH9	Septin-8	Sept8	mmu:20362				
331	0.0751	0.4753	0.5118	0.5338	1	Q07813	Apoptosis regulator BAX	Bax	mmu:12028				1
332	0.6548	0.4296	0.6800	0.5387	1	Q6P9Q6	FK506-binding protein 15;Peptidyl-prolyl cis-trans isomerase	Fkbp15	mmu:338355				
333	0.2660	0.2104	0.4494	0.5423	1	Q8BWY3	Eukaryotic peptide chain release factor subunit 1	Etf1	mmu:225363				
334	0.0430	0.4014	1.0843	0.5452	1	Q9EQ61	Pescadillo homolog	Pes1	mmu:64934				
335	0.3894	0.2337	0.9777	0.5454	1	Q9JK48	Endophilin-B1	Sh3glb1	mmu:54673				1
336	0.0650	0.0522	0.2043	0.5515	1	P61025	Cyclin-dependent kinases regulatory subunit;Cyclin-dependent kinases regulatory subunit 1	Cks1brt;Ck	mmu:54124				
337	0.3615	0.3104	0.1455	0.5623	1	Q4KMM3	Oxidation resistance protein 1	Oxr1	mmu:170719	1			
338	0.1675	0.0479	0.3262	0.5668	1	Q9WVE8	Protein kinase C and casein kinase substrate in neurons protein 2	Pacsin2	mmu:23970				
339	0.6091	0.4367	0.3367	0.5677	1	P29391	Ferritin;Ferritin light chain 1	Ftl1					
340	0.5472	0.6429	0.4381	0.5714	1	O09044	Synaptosomal-associated protein;Synaptosomal-associated protein 23	Snap23	mmu:20619				
341	0.3634	0.3245	0.6225	0.5736	1	Q6P549	Phosphatidylinositol 3,4,5-trisphosphate 5-phosphatase 2	Inppl1	mmu:16332				
342	0.4677	0.2440	0.4120	0.5814	1	A2ALS5	Rap1 GTPase-activating protein 1	Rap1gap	mmu:110351				
343	0.2529	0.5962	0.4919	0.5847	1	Q9JLQ0	CD2-associated protein	Cd2ap	mmu:12488				
344	0.2601	0.5743	0.5292	0.5855	1	Q6ZPJ3	E2/E3 hybrid ubiquitin-protein ligase UBE2O	Ube2o	mmu:217342			1	
345	0.2783	0.1310	0.4331	0.5920	1	P53811	Phosphatidylinositol transfer protein beta isoform	Pitpnb	mmu:56305				
346	0.6913	0.0238	0.1498	0.5958	1	O08848	60 kDa SS-A/Ro ribonucleoprotein	Trove2	mmu:20822				
347	0.0666	0.6490	0.6892	0.5959	1	Q8BFW7	Lipoma-preferred partner homolog	Lpp	mmu:210126				
348	0.7468	0.3628	0.8125	0.5970	1	P21981	Protein-glutamine gamma-glutamyltransferase 2	Tgm2	mmu:21817				
349	0.3730	0.1445	0.3565	0.6006	1	O55013	Trafficking protein particle complex subunit 3	Trappc3	mmu:27096				
350	0.8777	0.4634	0.9791	0.6036	1	Q99LJ0	CTTNBP2 N-terminal-like protein	Cttnbp2nl	mmu:80281				
351	0.5349	0.6005	0.2521	0.6053	1	Q99J85	Neuronal pentraxin receptor	Nptxr					
352	0.5716	0.0882	0.9174	0.6073	1	Q9D8T2	Gasdermin-D	Gsdmdc1	mmu:69146				
353	0.6292	0.1679	0.1080	0.6116	1	P28656	Nucleosome assembly protein 1-like 1	Nap1l1	mmu:53605				
354	0.3500	0.6838	0.3600	0.6123	1	Q6ZPU9	KIF1-binding protein	Kbp;25100	mmu:72320				
355	1.0522	0.3453	0.9209	0.6209	1	P57776	Elongation factor 1-delta	Eef1d	mmu:66656				
356	0.1824	0.5882	0.6296	0.6448	1	P63001	Ras-related C3 botulinum toxin substrate 1;Ras-related C3 botulinum toxin substrate 2	Rac1;Rac2	mmu:19353				
357	0.4422	0.4640	0.2397	0.6461	1	Q9ESE1	Lipopolysaccharide-responsive and beige-like anchor protein	Lrba	mmu:80877				
358	0.4251	0.2465	0.5481	0.6529	1	Q61151	Serine/threonine-protein phosphatase 2A 56 kDa regulatory subunit epsilon isoform	Ppp2r5e	mmu:26932				
359	0.8346	0.6765	0.7598	0.6629	1	O09012	Peroxisomal targeting signal 1 receptor	Pex5	mmu:19305				
360	0.2505	0.5589	0.8230	0.6658	1	Q9ER69	Pre-mRNA-splicing regulator WTAP	Wtap	mmu:60532				
361	0.3182	0.3043	0.3699	0.6729	1	O88456	Calpain small subunit 1	Capns1	mmu:12336				
362	0.6648	0.1089	0.9861	0.6829	1	Q91VH2	Sorting nexin-9	Snx9	mmu:66616				
363	0.4168	0.6714	0.7035	0.6912	1	Q6ZPZ3	Zinc finger CCCH domain-containing protein 4	Zc3h4	mmu:330474				
364	0.9204	0.7387	0.2465	0.6926	1	P25799	Nuclear factor NF-kappa-B p105 subunit;Nuclear factor NF-kappa-B p50 subunit	Nfkb1	mmu:18033				1
365	0.0451	0.3713	0.8271	0.6980	1	Q9CQU3	Protein RER1	Rer1	mmu:67830				
366	0.3791	0.8234	0.7980	0.6980	1	Q9CZX7	Type 2 phosphatidylinositol 4,5-bisphosphate 4-phosphatase	Tmem55a	mmu:72519				
367	1.0849	0.4978	0.3311	0.7084	1	Q61189	Methylosome subunit pICln	Clns1a					

368	0.1840	0.1059	1.0065	0.7111	1	Q9EQG9	Collagen type IV alpha-3-binding protein	Col4a3bp	mmu:68018					
369	1.0458	0.7088	0.7016	0.7326	1	Q8R001	Microtubule-associated protein RP/EB family member 2	Mapre2	mmu:212307					
370	0.3957	1.3379	0.5793	0.7400	1	O89032	SH3 and PX domain-containing protein 2A	Sh3pxd2a	mmu:14218					
371	0.1925	0.0494	0.7312	0.7472	1	Q04207	Transcription factor p65	Rela	mmu:19697		1			1
372	0.5533	0.0510	0.4538	0.7522	1	P36993	Protein phosphatase 1B	Ppm1b	mmu:19043			1		
373	1.1697	0.2986	0.3758	0.7648	1	Q7TMQ7	WD repeat-containing protein 91	Wdr91	mmu:101240					
374	0.0243	0.5739	0.3476	0.7784	1	P99027	60S acidic ribosomal protein P2	Rplp2	mmu:67186					
375	0.6517	0.2468	0.1248	0.7825	1	Q3TCJ1	BRISC complex subunit Abro1	Fam175b	mmu:109359					
376	0.4515	0.6470	0.2863	0.7829	1	Q9JLJ2	4-trimethylaminobutyraldehyde dehydrogenase	Aldh9a1	mmu:56752					
377	1.2319	0.2163	0.6608	0.7918	1	O55142	60S ribosomal protein L35a	Rpl35a	mmu:57808					
378	0.0099	0.0075	0.6268	0.7943	1	P08207	Protein S100-A10	S100a10	mmu:20194					
379	0.3386	0.2267	0.4038	0.7965	1	Q5I012	Putative sodium-coupled neutral amino acid transporter 10	Slc38a10	mmu:72055					
380	0.5280	0.9905	0.4285	0.8066	1	Q8R2U4	N-terminal Xaa-Pro-Lys N-methyltransferase 1;N-terminal Xaa-Pro-Lys N-methyltransferase 1, N-te	Ntmt1	mmu:66617					
381	0.7597	0.8765	0.5570	0.8101	1	Q9JIM1	Equilibrative nucleoside transporter 1	Slc29a1	mmu:63959					
382	0.1561	0.6898	0.0590	0.8204	1	P27808	Alpha-1,3-mannosyl-glycoprotein 2-beta-N-acetylglucosaminyltransferase	Mgat1	mmu:17308					
383	0.5724	1.1504	0.7830	0.8215	1	Q64327	Male-enhanced antigen 1	Mea1	mmu:17256					
384	0.1733	0.8589	0.0304	0.8342	1	Q9D2C2	Protein SAAL1	Saal1	mmu:78935					
385	0.4839	0.6691	0.7274	0.8558	1	Q9DBR4	Amyloid beta A4 precursor protein-binding family B member 2	Apbb2	mmu:11787					
386	0.1469	0.6862	0.0774	0.8663	1	Q62193	Replication protein A 32 kDa subunit	Rpa2						
387	0.1824	0.9852	0.2284	0.9081	1	Q9JM76	Actin-related protein 2/3 complex subunit 3	Arpc3	mmu:56378					
388	0.1812	0.9503	1.3610	0.9117	1	Q80W47	WD repeat domain phosphoinositide-interacting protein 2	Wipi2	mmu:74781					
389	0.4134	0.0769	0.6758	0.9218	1	Q9R1X5	Multidrug resistance-associated protein 5	Abcc5	mmu:27416					
390	1.3486	0.2782	0.6571	0.9372	1	P54310	Hormone-sensitive lipase	Lipe	mmu:16890					
391	0.3978	0.5778	0.5594	0.9537	1	P63005	Platelet-activating factor acetylhydrolase IB subunit alpha	Pafah1b1	mmu:18472					
392	1.4122	1.2338	0.4001	0.9659	1	Q91YM2	Rho GTPase-activating protein 35	Arhgap35	mmu:232906					
393	0.0098	0.0792	2.0495	1.0026	1	Q9ES46	Beta-parvin	Parvb	mmu:170736					
394	0.2811	1.2268	1.1520	1.0510	1	Q8C863	E3 ubiquitin-protein ligase Itchy	Itch	mmu:16396				1	
395	1.0789	1.0604	1.9131	1.0511	1	Q8CB44	GRAM domain-containing protein 4	Gramd4	mmu:223752					
396	0.0455	1.7789	0.2529	1.0532	1	Q99L43	Phosphatidate cytidylyltransferase;Phosphatidate cytidylyltransferase 2	Cds2	mmu:110911					
397	0.8820	1.4203	0.6361	1.2140	1	O88413	Tubby-related protein 3	Tulp3	mmu:22158					
398	0.0160	1.3053	3.6511	1.2488	1	Q62448	Eukaryotic translation initiation factor 4 gamma 2	Eif4g2	mmu:13690					
399	0.1181	0.6217	0.1576	1.2683	1	Q7TNL7		Dusp9	mmu:75590					
400	1.1395	0.4973	0.8144	1.2735	1	Q05769	Prostaglandin G/H synthase 2	Ptgs2	mmu:19225		1			
401	1.0068	1.1407	2.0969	1.3141	1	Q8BG95	Protein phosphatase 1 regulatory subunit;Protein phosphatase 1 regulatory subunit 12B	Ppp1r12b						
402	0.4352	1.1462	1.2398	1.3315	1	Q9D0B6	Protein PBDC1	Pbdc1	mmu:67683					
403	4.1657	0.3078	0.6172	1.3538	1	P70188	Kinesin-associated protein 3	Kifap3	mmu:16579					
404	0.6265	0.7007	0.2327	1.3644	1	P52633	Signal transducer and transcription activator 6	Stat6	mmu:20852		1			
405	0.3356	0.0702	0.6603	1.4821	1	Q7TMY7	Importin-8	Ipo8	mmu:320727					
406	0.0411	2.3911	1.9603	1.5261	1	Q6NWW9	Fibronectin type III domain-containing protein 3B	Fndc3b	mmu:72007					
407	0.2531	1.4486	0.4217	1.5632	1	Q9JKN1	Zinc transporter 7	Slc30a7	mmu:66500					
408	0.0285	0.2739	2.8863	1.6716	1	Q8VBZ0	Dehydrogenase/reductase SDR family member on chromosome X homolog	Dhrsx						
409	0.2964	1.2781	0.6557	1.7929	1	P54728	UV excision repair protein RAD23 homolog B	Rad23b	mmu:19359					
410	0.5250	0.2985	0.5272	1.9242	1	Q6NZR5		Skiv2l	mmu:108077					
411	-4.6047	-0.3224	-1.5079	-2.1450	1	O09110	Dual specificity mitogen-activated protein kinase kinase 3	Map2k3	mmu:26397	1				
412	-0.7575	-0.2913	-1.1878	-0.7455	1	Q80TR8	Protein VPRBP	Vprbp	mmu:321006	1			1	
413	-0.5707	-0.7957	-0.6153	-0.6606	1	Q8C050	Ribosomal protein S6 kinase alpha-5	Rps6ka5	mmu:73086	1				
414	-0.1559	-1.4463	-0.2949	-0.6324	1	Q8BM85	TBC domain-containing protein kinase-like protein	Tbck	mmu:271981	1				
415	-0.3680	-0.8472	-0.1348	-0.4500	1	Q8R3L8	Cyclin-dependent kinase 8;Cyclin-dependent kinase 19	Cdk8;Cdk1	mmu:264064	1				
416	-0.2725	-0.1622	-0.7261	-0.3869	1	O35491	Dual specificity protein kinase CLK2	Clk2	mmu:12748	1				
417	-0.1015	-0.5356	-0.3399	-0.3257	1	Q8BK63	Casein kinase I isoform alpha	Csnk1a1		1				
418	-0.4901	-0.0081	-0.4380	-0.3107	1	Q60737	Casein kinase II subunit alpha	Csnk2a1	mmu:12995	1				
419	-0.0852	-0.3397	-0.4544	-0.2931	1	Q00993	Receptor protein-tyrosine kinase;Tyrosine-protein kinase receptor UFO	Axl	mmu:26362	1	1			
420	-0.1775	-0.1107	-0.4793	-0.2558	1	P70268	Serine/threonine-protein kinase N1	Pkn1	mmu:320795	1				
421	-0.4991	-0.0780	-0.0975	-0.2249	1	O54833	Casein kinase II subunit alpha	Csnk2a2	mmu:13000	1				
422	-0.1789	-0.4234	-0.0510	-0.2178	1	Q04736	Tyrosine-protein kinase Yes	Yes1	mmu:22612	1				
423	-0.2341	-0.0793	-0.2359	-0.1831	1	Q64729	Receptor protein serine/threonine kinase;TGF-beta receptor type-1	Tgfbr1	mmu:21812	1				
424	-0.1555	-0.1469	-0.1929	-0.1651	1	Q9ES74	Serine/threonine-protein kinase Nek7	Nek7	mmu:59125	1				
425	-0.0414	-0.1945	-0.1984	-0.1448	1	P05480	Neuronal proto-oncogene tyrosine-protein kinase Src	Src	mmu:20779	1	1			
426	-0.0082	-0.0328	-0.3436	-0.1282	1	Q62371	Discoidin domain-containing receptor 2	Ddr2	mmu:18214	1				
427	0.1436	0.1482	0.0724	0.1214	1	Q60855	Receptor-interacting serine/threonine-protein kinase 1	Ripk1	mmu:19766	1				1
428	0.1534	0.1873	0.3669	0.2359	1	O88643	Non-specific serine/threonine protein kinase;Serine/threonine-protein kinase PAK 1	Pak1		1				
429	0.3445	0.4473	0.0056	0.2658	1	Q8CFE4	SCY1-like protein 2	Scyl2	mmu:213326	1				
430	0.4574	0.2501	0.3407	0.3494	1	Q8CIN4	Serine/threonine-protein kinase PAK 2;PAK-2p27;PAK-2p34	Pak2	mmu:224105	1				
431	0.5862	0.4339	0.1815	0.4005	1	O70589	Peripheral plasma membrane protein CASK	Cask	mmu:12361	1				
432	0.5190	0.1107	0.6352	0.4216	1	P63085	Mitogen-activated protein kinase 1	Mapk1	mmu:26413	1	1			1
433	0.4518	0.2535	0.5626	0.4226	1	O54988	STE20-like serine/threonine-protein kinase	Slk	mmu:20874	1				
434	1.0972	0.6409	0.0768	0.6050	1	P35761	Dual specificity protein kinase TTK	Ttk		1				
435	0.2990	0.6818	0.9484	0.6431	1	Q3UHJ0	AP2-associated protein kinase 1	Aak1	mmu:269774	1				
436	1.1800	0.2334	0.5763	0.6632	1	Q9WVS8	Mitogen-activated protein kinase 7;Mitogen-activated protein kinase	Mapk7	mmu:23939	1	1			
437	0.7954	1.6395	0.7306	1.0552	1	O55098	Serine/threonine-protein kinase 10	Stk10	mmu:20868	1				
438	-0.3208	-5.2024	-0.7443	-2.0892	1	P61957	Small ubiquitin-related modifier 2;Small ubiquitin-related modifier;Small ubiquitin-related modifie	Sumo2;Sur	mmu:170930					
439	-0.2393	-3.2513	-2.6573	-2.0493	1	A2ANY6		Mdn1						
440	-4.1186	-0.0776	-0.7100	-1.6354	1	K3W4Q5		Fam186a						
441	-1.3048	-0.4184	-3.1651	-1.6294	1	Q5SUC9	Protein SCO1 homolog, mitochondrial	Sco1	mmu:52892					
442	-2.2172	-0.9237	-1.4646	-1.5352	1	Q9CQV7	Mitochondrial import inner membrane translocase subunit TIM14	Dnajc19	mmu:67713					
443	-1.2138	-2.2059	-1.0444	-1.4880	1	Q9CQI7	U2 small nuclear ribonucleoprotein B	Snrpb2	mmu:20639					
444	-2.1301	-1.3500	-0.6599	-1.3800	1	Q8BWM0	Prostaglandin E synthase 2;Prostaglandin E synthase 2 truncated form	Ptges2	mmu:96979					
445	-0.9449	-1.0473	-2.0901	-1.3608	1	Q9JJA2	Conserved oligomeric Golgi complex subunit 8	Cog8	mmu:97484					
446	-3.0290	-0.3686	-0.6830	-1.3602	1	Q9QZ73	DCN1-like protein;DCN1-like protein 1	Dcun1d1	mmu:114893					
447	-2.5019	-0.2799	-1.2401	-1.3406	1	Q99MR3	Solute carrier family 12 member 9	Slc12a9	mmu:83704					
448	-0.3951	-1.2854	-2.3122	-1.3309	1	Q9JKW0	ADP-ribosylation factor-like protein 6-interacting protein 1	Arl6ip1	mmu:54208					
449	-0.8106	-1.7832	-1.3509	-1.3149	1	P07742	Ribonucleoside-diphosphate reductase large subunit	Rrm1	mmu:20133					
450	-1.0097	-1.6807	-1.2256	-1.3053	1	Q99J93	Interferon-induced transmembrane protein 2	Ifitm2	mmu:80876					
451	-3.0713	-0.1502	-0.5221	-1.2479	1	Q9WVL1	AP-4 complex subunit sigma-1	Ap4s1	mmu:11782					
452	-0.8642	-1.9894	-0.7849	-1.2128	1	Q9EPL2	Calsyntenin-1;Soluble Alc-alpha;CTF1-alpha	Clstn1	mmu:65945					
453	-0.0956	-1.9227	-1.6002	-1.2062	1	P35601	Replication factor C subunit 1	Rfc1	mmu:19687					
454	-1.8881	-1.0217	-0.6989	-1.2029	1	Q8CGI1	Protein FAM193A	Fam193a	mmu:231128					
455	-1.8334	-1.0042	-0.6940	-1.1772	1	P48755	Fos-related antigen 1	Fosl1	mmu:14283		1			
456	-0.2432	-1.4376	-1.8198	-1.1669	1	Q9D773	39S ribosomal protein L2, mitochondrial	Mrpl2	mmu:27398					
457	-0.5981	-0.5503	-2.2410	-1.1298	1	P04184	Thymidine kinase, cytosolic	Tk1	mmu:21877					
458	-1.3427	-0.3561	-1.4669	-1.0552	1	Q9D8C6	Mediator of RNA polymerase II transcription subunit 11	Med11	mmu:66172					
459	-0.3733	-0.4911	-2.2944	-1.0529	1	Q9Z0W3	Nuclear pore complex protein Nup160	Nup160	mmu:59015					
460	-0.9730	-0.5550	-1.6049	-1.0443	1	Q60648	Ganglioside GM2 activator	Gm2a	mmu:14667					
461	-0.5535	-0.2277	-2.3270	-1.0361	1	Q8R123	FAD synthase;Molybdenum cofactor biosynthesis protein-like region;FAD synthase region	Flad1	mmu:319945					
462	-2.2317	-0.1270	-0.7260	-1.0282	1	Q4LDD4	Arf-GAP with Rho-GAP domain, ANK repeat and PH domain-containing protein 1	Arap1	mmu:69710					
463	-0.1766	-2.4672	-0.4287	-1.0242	1	Q00558	Factor VIII intron 22 protein	F8a;F8a1						
464	-2.1302	-0.2173	-0.7001	-1.0159	1	Q811U4	Mitofusin-1	Mfn1	mmu:67414					
465	-2.0558	-0.5988	-0.3693	-1.0080	1	P70318	Nucleolysin TIAR	Tial1;Tia1	mmu:21843					1
466	-2.8153	-0.0598	-0.1455	-1.0069	1	Q9JIS8	Solute carrier family 12 member 4	Slc12a4	mmu:20498					
467	-1.2458	-0.7898	-0.9713	-1.0023	1	Q61024	Asparagine synthetase [glutamine-hydrolyzing]	Asns	mmu:27053					
468	-0.3324	-1.5976	-1.0604	-0.9968	1	Q91ZV3	Discoidin, CUB and LCCL domain-containing protein 2	Dcbld2	mmu:73379					
469	-0.7509	-0.6400	-1.5734	-0.9881	1	Q8BWY7	Zinc transporter ZIP11	Slc39a11	mmu:69806					
470	-0.1179	-0.2990	-2.5344	-0.9838	1	Q9DC69	NADH dehydrogenase [ubiquinone] 1 alpha subcomplex subunit 9, mitochondrial	Ndufa9	mmu:66108					
471	-0.4920	-0.2397	-2.2088	-0.9802	1	Q9QZD8	Mitochondrial dicarboxylate carrier	Slc25a10	mmu:27376					
472	-0.5241	-1.4544	-0.9373	-0.9719	1	Q8K4L3	Supervillin	Svil	mmu:225115					
473	-1.4423	-0.7706	-0.6837	-0.9655	1	Q3U821		Wdr75	mmu:73674					
474	-0.8574	-1.0380	-0.9864	-0.9606	1	Q9Z2Q5	39S ribosomal protein L40, mitochondrial	Mrpl40	mmu:18100					
475	-1.0113	-0.0316	-1.7988	-0.9472	1	Q8BZH4	Pogo transposable element with ZNF domain	Pogz	mmu:229584					
476	-0.3880	-0.5854	-1.8452	-0.9395	1	P17751	Triosephosphate isomerase	Tpi1	mmu:21991					
477	-2.0722	-0.4592	-0.2496	-0.9270	1	Q5PRF0	HEAT repeat-containing protein 5A	Heatr5a	mmu:320487					
478	-0.2878	-0.1052	-2.3786	-0.9239	1	Q64523	Histone H2A;Histone H2A type 2-C;Histone H2A type 2-A	Hist1h2al;I	mmu:319176					
479	-1.1345	-0.6826	-0.9541	-0.9237	1	Q8BH78	Reticulon	Rtn4						
480	-0.4698	-1.5968	-0.7034	-0.9233	1	P53995	Anaphase-promoting complex subunit 1	Anapc1	mmu:17222				1	
481	-1.5319	-0.5226	-0.6934	-0.9160	1	Q3UE37	Ubiquitin-conjugating enzyme E2 Z	Ube2z	mmu:268470				1	
482	-0.9246	-1.3510	-0.4296	-0.9017	1	P58929	Glucocorticoid modulatory element-binding protein 2	Gmeb2	mmu:229004					
483	-0.8067	-0.6903	-1.1751	-0.8907	1	Q91WD0	Protein GPR108	Gpr108	mmu:78308					
484	-0.4482	-2.0120	-0.1407	-0.8670	1	Q9CYI0	Protein Njmu-R1	5730455P1	mmu:70591					
485	-0.7162	-1.1648	-0.6867	-0.8559	1	Q8BM55	Transmembrane protein 214	Tmem214	mmu:68796					
486	-0.5736	-1.0045	-0.9744	-0.8508	1	Q9D1B9	39S ribosomal protein L28, mitochondrial	Mrpl28	mmu:68611					
487	-0.4763	-1.3912	-0.6826	-0.8500	1	Q91YP0	L-2-hydroxyglutarate dehydrogenase, mitochondrial	L2hgdh	mmu:217666					
488	-1.9852	-0.3539	-0.1928	-0.8440	1	Q9DBU0	Transmembrane 9 superfamily member 1	Tm9sf1	mmu:74140					
489	-1.3188	-0.5047	-0.6944	-0.8393	1	Q8C0N2	Glycerol-3-phosphate acyltransferase 3	Agpat9	mmu:231510					
490	-0.1626	-1.4787	-0.8740	-0.8384	1	Q78YZ6	Short coiled-coil protein	Scoc	mmu:56367					
491	-0.9424	-1.0259	-0.5395	-0.8359	1	Q8R127	Saccharopine dehydrogenase-like oxidoreductase	Sccpdh	mmu:109232					
492	-1.0614	-0.8371	-0.5710	-0.8232	1	F8VPK0		Ttc37	mmu:218343					
493	-1.9633	-0.3920	-0.0779	-0.8111	1	Q8CI04	Conserved oligomeric Golgi complex subunit 3	Cog3						

494	-0.1929	-1.2190	-0.9979	-0.8033		1	F8VPU2	FERM, RhoGEF and pleckstrin domain-containing protein 1	Farp1	mmu:223254				
495	-1.1307	-0.1660	-1.1049	-0.8005		1	Q9WV54	Acid ceramidase;Acid ceramidase subunit alpha;Acid ceramidase subunit beta	Asah1	mmu:11886				
496	-0.7602	-0.0170	-1.6118	-0.7963		1	Q4KML4	Costars family protein ABRACL	Abracl	mmu:73112				
497	-1.0770	-1.0695	-0.1997	-0.7821		1	Q68FE6	Protein FAM65A	Fam65a	mmu:75687				
498	-0.7755	-0.7914	-0.7694	-0.7788		1	Q5DU37	Zinc finger FYVE domain-containing protein 26	Zfyve26	mmu:211978				
499	-0.0886	-0.7132	-1.4801	-0.7606		1	Q9Z2D8	Methyl-CpG-binding domain protein 3	Mbd3	mmu:17192				
500	-0.3822	-0.5358	-1.3303	-0.7494		1	Q9DCT2	NADH dehydrogenase [ubiquinone] iron-sulfur protein 3, mitochondrial	Ndufs3	mmu:68349				1
501	-0.7844	-0.9788	-0.4727	-0.7453		1	Q9EP82	tRNA (guanine-N(7)-)-methyltransferase non-catalytic subunit Wdr4;tRNA (guanine-N(7)-)-methylt	Wdr4	mmu:57773				
502	-0.2724	-0.6025	-1.3490	-0.7413		1	Q9CPQ1	Cytochrome c oxidase subunit 6C	Cox6c	mmu:12864				
503	-1.5213	-0.1266	-0.5667	-0.7382		1	Q6ZQ93	Ubiquitin carboxyl-terminal hydrolase 34;Ubiquitin carboxyl-terminal hydrolase	Usp34	mmu:17847				
504	-1.2679	-0.2400	-0.6783	-0.7287		1	Q80V62	Fanconi anemia group D2 protein homolog	Fancd2	mmu:211651	1			
505	-1.9209	-0.0876	-0.1471	-0.7185		1	Q8BGU5	Cyclin-Y	Ccny	mmu:67974				
506	-0.7404	-0.6299	-0.7852	-0.7185		1	Q9D083	Kinetochore protein Spc24	Spc24	mmu:67629				
507	-0.4156	-0.8819	-0.8560	-0.7178		1	Q9D8P4	39S ribosomal protein L17, mitochondrial	Mrpl17	mmu:27397				
508	-0.5390	-0.5195	-1.0910	-0.7165		1	Q5PSV9	Mediator of DNA damage checkpoint protein 1	Mdc1	mmu:240087				
509	-0.1138	-1.2685	-0.7486	-0.7103		1	Q8C6B2	Rhotekin	Rtkn	mmu:20166				
510	-1.2057	-0.4219	-0.4737	-0.7004		1	Q9D1L9	Ragulator complex protein LAMTOR5	Lamtor5	mmu:68576				1
511	-1.5000	-0.1694	-0.4232	-0.6975		1	Q6GV12	3-ketodihydrosphingosine reductase	Kdsr	mmu:70750				
512	-0.6045	-1.0500	-0.4337	-0.6961		1	Q6P6L6		Gemin4	mmu:276919				
513	-0.6691	-0.7679	-0.6398	-0.6923		1	Q9D8Z2	TP53-regulated inhibitor of apoptosis 1	Triap1	mmu:69076				1
514	-0.9996	-0.4055	-0.6651	-0.6901		1	Q3UGP9	Leucine-rich repeat-containing protein 58	Lrrc58	mmu:320184				
515	-1.3551	-0.2151	-0.4663	-0.6788		1	O88587	Catechol O-methyltransferase	Comt	mmu:12846				
516	-1.0818	-0.3082	-0.6413	-0.6771		1	Q3UI43	BRISC and BRCA1-A complex member 1	Babam1	mmu:68251				
517	-0.4474	-0.8061	-0.7401	-0.6645		1	Q9CX86	Heterogeneous nuclear ribonucleoprotein A0	Hnrnpa0	mmu:77134				
518	-0.4180	-0.6638	-0.9024	-0.6614		1	Q9WV72	Ankyrin repeat and SOCS box protein 3	Asb3	mmu:65257			1	
519	-0.1935	-0.9164	-0.8628	-0.6576		1	P70213	Friend virus susceptibility protein 1	Fv1	mmu:14349				
520	-0.6213	-0.4134	-0.9295	-0.6547		1	Q09143	High affinity cationic amino acid transporter 1	Slc7a1	mmu:11987				
521	-0.0491	-1.0942	-0.8068	-0.6500		1	Q80UY2	E3 ubiquitin-protein ligase KCMF1	Kcmf1	mmu:74287			1	
522	-0.2649	-0.5014	-1.1516	-0.6393		1	P61211	ADP-ribosylation factor-like protein 1	Arl1	mmu:104303				
523	-0.4371	-0.7874	-0.6806	-0.6350		1	Q8CCF0	U4/U6 small nuclear ribonucleoprotein Prp31	Prpf31	mmu:68988				
524	-0.3515	-0.4053	-1.1212	-0.6260		1	Q9Z1E4	Glycogen [starch] synthase, muscle	Gys1	mmu:14936				
525	-0.1708	-0.4858	-1.1613	-0.6060		1	Q8BFZ9	Erlin-2	Erlin2	mmu:244373				
526	-0.9591	-0.5301	-0.3252	-0.6048		1	Q61655	ATP-dependent RNA helicase DDX19A	Ddx19a;Dc	mmu:13680				
527	-0.3695	-0.6702	-0.7688	-0.6028		1	Q3UQ84	Threonine--tRNA ligase, mitochondrial	Tars2	mmu:71807				
528	-0.2518	-0.7013	-0.8488	-0.6006		1	Q9CQ92	Mitochondrial fission 1 protein	Fis1	mmu:66437				
529	-0.3625	-0.6512	-0.7582	-0.5906		1	Q8R3N1	Nucleolar protein 14	Nop14	mmu:75416				
530	-0.2289	-1.0116	-0.5277	-0.5894		1	Q3U898	Myeloma-overexpressed gene 2 protein homolog	Myeov2	mmu:66915				
531	-1.1722	-0.3657	-0.2244	-0.5874		1	Q9D061	Acyl-CoA-binding domain-containing protein 6	Acbd6	mmu:72482				
532	-0.8596	-0.7735	-0.1174	-0.5835		1	Q9QUR6	Prolyl endopeptidase	Prep	mmu:19072				
533	-0.1408	-0.0144	-1.5925	-0.5826		1	P70302	Stromal interaction molecule 1	Stim1	mmu:20866				
534	-0.0996	-0.0459	-1.5926	-0.5794		1	P57716	Nicastrin	Ncstn	mmu:59287				
535	-0.5252	-0.4621	-0.7418	-0.5764		1	Q9CXR1	Dehydrogenase/reductase SDR family member 7	Dhrs7	mmu:66375				
536	-1.1749	-0.1450	-0.3838	-0.5679		1	Q07139	Protein ECT2	Ect2	mmu:13605	1			
537	-0.2066	-0.3253	-1.1554	-0.5624		1	Q6P2B1	Transportin-3	Tnpo3	mmu:320938				
538	-0.7670	-0.8380	-0.0814	-0.5621		1	B2RY04	Dedicator of cytokinesis protein 5	Dock5	mmu:68813				
539	-0.0961	-1.3509	-0.2337	-0.5602		1	P60898	DNA-directed RNA polymerase II subunit RPB9;DNA-directed RNA polymerase subunit	Polr2i	mmu:69920				
540	-0.3663	-0.2520	-1.0546	-0.5576		1	Q8K4F6	Probable 28S rRNA (cytosine-C(5))-methyltransferase	Nsun5	mmu:100609				
541	-0.3668	-0.8017	-0.4732	-0.5472		1	Q148V7	LisH domain and HEAT repeat-containing protein KIAA1468	Kiaa1468;2	mmu:227446				
542	-0.9836	-0.5816	-0.0747	-0.5466		1	P70297	Signal transducing adapter molecule 1	Stam	mmu:20844				
543	-0.5052	-1.0399	-0.0922	-0.5458		1	Q91W34			mmu:233913				
544	-0.2991	-0.0920	-1.2458	-0.5456		1	A2AKU9	ATP synthase subunit gamma	Atp5c1					
545	-0.8275	-0.5284	-0.2713	-0.5424		1	Q8BH86	UPF0317 protein C14orf159 homolog, mitochondrial	9030617O0	mmu:217830				
546	-0.1491	-0.8401	-0.6071	-0.5321		1	Q9D7N3	28S ribosomal protein S9, mitochondrial	Mrps9	mmu:69527				
547	-0.5834	-0.6393	-0.3695	-0.5307		1	Q8VCB1	Nucleoporin NDC1	Ndc1	mmu:72787				
548	-0.0140	-0.5788	-0.9837	-0.5255		1	P49070	Calcium signal-modulating cyclophilin ligand	Camlg	mmu:12328				
549	-0.4248	-0.3436	-0.7750	-0.5145		1	Q8BIQ5	Cleavage stimulation factor subunit 2	Cstf2	mmu:108062				
550	-0.6294	-0.1871	-0.7218	-0.5128		1	Q8C0D7	Inhibitor of growth protein 4	Ing4	mmu:28019				
551	-0.4865	[illegible]	-1.0428	-0.5104		1	Q9ER41	Torsin-1B	Tor1b	mmu:30934				
552	-0.4765	-0.8710	-0.1717	-0.5064		1	Q9EQC1	3 beta-hydroxysteroid dehydrogenase type 7	Hsd3b7	mmu:101502				
553	-0.7517	-0.2676	-0.4985	-0.5059		1	Q9CRT8	Exportin-T	Xpot					
554	-1.2511	-0.2146	-0.0411	-0.5023		1	Q8K201	Keratinocyte-associated transmembrane protein 2	9530068E0	mmu:213673				
555	-0.5532	-0.3452	-0.5969	-0.4984		1	P43406	Integrin alpha-V;Integrin alpha-V heavy chain;Integrin alpha-V light chain	Itgav	mmu:16410	1			
556	-1.1697	-0.0176	-0.3054	-0.4976		1	O88983	Syntaxin-8	Stx8	mmu:55943				
557	-0.0950	-0.7265	-0.6689	-0.4968		1	Q9JLI6	Selenocysteine lyase	Scly	mmu:50880				
558	-0.3383	-0.6886	-0.4386	-0.4885		1	O89001	Carboxypeptidase D	Cpd	mmu:12874				
559	-0.0437	-0.5819	-0.8331	-0.4862		1	P32233	Developmentally-regulated GTP-binding protein 1	Drg1	mmu:13494				
560	-0.2647	-0.6190	-0.5696	-0.4844		1	B1AUR6	Protein MMS22-like	Mms22l	mmu:212377				
561	-0.1177	[illegible]	-1.3229	-0.4804		1	Q9D8X5	CCR4-NOT transcription complex subunit 8	Cnot8	mmu:69125				
562	-0.7820	-0.4705	-0.1706	-0.4744		1	P22892	AP-1 complex subunit gamma-1	Ap1g1	mmu:11765				
563	-0.0369	-0.1295	-1.2469	-0.4711		1	Q9D958	Signal peptidase complex subunit 1	Spcs1	mmu:69019				
564	-0.6835	-0.1671	-0.5624	-0.4710		1	Q6PA06	Atlastin-2	Atl2	mmu:56298				
565	-0.9016	-0.4549	-0.0495	-0.4687		1	Q80Y81	Zinc phosphodiesterase ELAC protein 2	Elac2	mmu:68626				
566	-0.5544	-0.4047	-0.4150	-0.4580		1	Q922Q8	Leucine-rich repeat-containing protein 59	Lrrc59	mmu:98238				
567	-0.5693	-0.1124	-0.6888	-0.4568		1	O35459	Delta(3,5)-Delta(2,4)-dienoyl-CoA isomerase, mitochondrial	Ech1	mmu:51798				
568	-0.3476	-0.9740	-0.0473	-0.4563		1	P51175	Protoporphyrinogen oxidase	Ppox	mmu:19044				
569	-0.3564	-0.8376	-0.1709	-0.4550		1	O35127	Protein C10	Grcc10	mmu:14790				
570	-0.0969	-1.0871	-0.1758	-0.4533		1	P46737	Lys-63-specific deubiquitinase BRCC36	Brcc3	mmu:210766				
571	-0.3055	-0.9691	-0.0773	-0.4506		1	Q9ERE7	LDLR chaperone MESD	Mesdc2	mmu:67943				
572	-0.7637	-0.1502	-0.4264	-0.4468		1	Q7SIG6	Arf-GAP with SH3 domain, ANK repeat and PH domain-containing protein 2	Asap2	mmu:211914				
573	-0.9757	-0.1873	-0.1759	-0.4463		1	Q6P9L6	Kinesin-like protein KIF15	Kif15	mmu:209737				
574	-0.8597	-0.2093	-0.2671	-0.4454		1	Q8BRT1	CLIP-associating protein 2	Clasp2	mmu:76499				
575	-0.3596	-0.7593	-0.2143	-0.4444		1	P58468	Protein FAM207A	Fam207a	mmu:108707				
576	-0.2412	-0.6054	-0.4802	-0.4423		1	Q06335	Amyloid-like protein 2	Aplp2	mmu:11804				
577	-0.7592	-0.1546	-0.4013	-0.4384		1	Q8C5Q4	G-rich sequence factor 1	Grsf1	mmu:231413				
578	-0.0448	-0.5670	-0.6963	-0.4360		1	Q6NVF0	Inositol polyphosphate 5-phosphatase OCRL-1	Ocrl	mmu:320634		1		
579	-0.8149	-0.4392	-0.0503	-0.4348		1	Q8R5M8	Cell adhesion molecule 1	Cadm1	mmu:54725				
580	-0.3384	-0.4120	-0.5427	-0.4310		1	P11370	MLV-related proviral Env polyprotein;Surface protein;Transmembrane protein;Retrovirus-related	Fv4					
581	-0.1587	-0.3909	-0.7298	-0.4265		1	O35857	Mitochondrial import inner membrane translocase subunit TIM44	Timm44	mmu:21856				
582	-0.1886	-1.0597	-0.0310	-0.4264		1	Q6P4T1		Snx19	mmu:102607				
583	-0.3941	-0.6210	-0.2627	-0.4259		1	Q9R087	Glypican-6;Secreted glypican-6	Gpc6	mmu:23888				
584	-0.4417	-0.0602	-0.7748	-0.4256		1	P08556	GTPase NRas	Nras					
585	-0.4715	-0.3747	-0.4237	-0.4233		1	P63038	60 kDa heat shock protein, mitochondrial	Hspd1	mmu:15510	1			
586	-0.5106	-0.0153	-0.7425	-0.4228		1	Q9CQD1	Ras-related protein Rab-5A	Rab5a	mmu:271457				
587	-0.3933	-0.3257	-0.5380	-0.4190		1	Q64737	Trifunctional purine biosynthetic protein adenosine-3;Phosphoribosylamine--glycine ligase;Phospl	Gart	mmu:14450				
588	-0.8670	-0.2959	-0.0697	-0.4109		1	Q8BHJ9	Pre-mRNA-splicing factor SLU7	Slu7	mmu:193116				
589	-0.6865	-0.4520	-0.0925	-0.4103		1	Q9CYZ2	Tumor protein D54	Tpd52l2	mmu:66314				
590	-0.0906	-0.3221	-0.8148	-0.4092		1	Q9D3U0	Putative tRNA pseudouridine synthase Pus10	Pus10	mmu:74467				
591	-0.0557	-0.3178	-0.8487	-0.4074		1	O88856	Protein-tyrosine sulfotransferase 2	Tpst2					
592	-0.1871	-0.8322	-0.1737	-0.3977		1	Q64449	C-type mannose receptor 2	Mrc2	mmu:17534				
593	-0.1187	-0.4975	-0.5669	-0.3944		1	Q9QYC0	Alpha-adducin	Add1	mmu:11518				
594	-0.3445	-0.2977	-0.5401	-0.3941		1	O55033	Cytoplasmic protein NCK2	Nck2	mmu:17974				
595	-0.4107	-0.1322	-0.6239	-0.3889		1	P35276	Ras-related protein Rab-3D	Rab3d	mmu:19340				
596	-0.0159	-0.3380	-0.8105	-0.3881		1	Q9ESJ0	Exportin-4	Xpo4	mmu:57258				
597	-0.2939	-0.1720	-0.6950	-0.3870		1	P23475	X-ray repair cross-complementing protein 6	Xrcc6	mmu:14375				
598	-0.2064	-0.3149	-0.6324	-0.3846		1	P09055	Integrin beta-1	Itgb1	mmu:16412				
599	-0.3731	-0.1711	-0.6004	-0.3815		1	Q99KI3	ER membrane protein complex subunit 3	Emc3	mmu:66087				
600	-0.5202	-0.1537	-0.4648	-0.3796		1	P58742	Aladin	Aaas	mmu:223921				
601	-0.3464	-0.5818	-0.2096	-0.3793		1	Q8R4R6	Nucleoporin NUP53	Nup35	mmu:69482				
602	-0.2752	-0.0818	-0.7783	-0.3784		1	Q8VCF0	Mitochondrial antiviral-signaling protein	Mavs	mmu:228607				
603	-0.5290	-0.3071	-0.2958	-0.3773		1	Q9CQG2	Methyltransferase-like protein;Methyltransferase-like protein 16	Mettl16	mmu:67493				
604	-0.4407	-0.5254	-0.1632	-0.3764		1	Q8VDI7	Ubiquitin-associated domain-containing protein 1	Ubac1	mmu:98766			1	
605	-0.6329	-0.3713	-0.1018	-0.3687		1	Q62465	Synaptic vesicle membrane protein VAT-1 homolog	Vat1	mmu:26949				
606	-0.3608	-0.3557	-0.3881	-0.3682		1	Q8QZY9	Splicing factor 3B subunit 4	Sf3b4	mmu:107701				
607	-0.1450	-0.6983	-0.2554	-0.3662		1	P97434	Myosin phosphatase Rho-interacting protein	Mprip	mmu:26936				
608	-0.2142	-0.2375	-0.6453	-0.3657		1	Q9QZ23	NFU1 iron-sulfur cluster scaffold homolog, mitochondrial	Nfu1	mmu:56748				
609	-0.3752	-0.5936	-0.1275	-0.3654		1	Q8BVA5	UPF0554 protein C2orf43 homolog		mmu:68832				
610	-0.2657	-0.4151	-0.4141	-0.3650		1	Q8CD92	Tetratricopeptide repeat protein 27	Ttc27	mmu:74196				
611	-0.4882	-0.2701	-0.3365	-0.3649		1	Q9CQN1	Heat shock protein 75 kDa, mitochondrial	Trap1	mmu:68015				
612	-0.1032	-0.0404	-0.9479	-0.3638		1	P31324	cAMP-dependent protein kinase type II-beta regulatory subunit	Prkar2b	mmu:19088				
613	-0.2054	-0.8162	-0.0492	-0.3569		1	Q3UPH1	Protein PRRC1	Prrc1	mmu:73137				
614	-0.4079	-0.1148	-0.5449	-0.3559		1	Q9R1J0	Sterol-4-alpha-carboxylate 3-dehydrogenase, decarboxylating	Nsdhl	mmu:18194				
615	-0.5250	-0.2025	-0.3392	-0.3556		1	P36536	GTP-binding protein SAR1a	Sar1a					
616	-0.4397	-0.4142	-0.2128	-0.3556		1	Q9DCJ5	NADH dehydrogenase [ubiquinone] 1 alpha subcomplex subunit 8	Ndufa8	mmu:68375				
617	-0.9405	-0.0629	-0.0628	-0.3554		1	O35381	Acidic leucine-rich nuclear phosphoprotein 32 family member A	Anp32a	mmu:11737				
618	-0.0675	-0.2552	-0.7416	-0.3548		1	P61021	Ras-related protein Rab-5B	Rab5b	mmu:19344				
619	-0.0427	-0.4353	-0.5761	-0.3514		1	Q8BXC6	COMM domain-containing protein 2	Commd2	mmu:52245				

620	-0.4539	-0.3168	-0.2834	-0.3514	1	B1AXD8	Akirin-2	Akirin2	mmu:433693				
621	-0.4841	-0.1815	-0.3830	-0.3495	1	Q8VEA8	Ras-related protein Rab-7b	Rab7b;543	mmu:226421				
622	-0.2445	-0.4596	-0.3412	-0.3484	1	Q5H8C4	Vacuolar protein sorting-associated protein 13A	Vps13a	mmu:271564				
623	-0.3527	-0.3251	-0.3509	-0.3429	1	Q99MS7	EH domain-binding protein 1-like protein 1	Ehbp1l1	mmu:114601				
624	-0.2533	-0.2435	-0.5266	-0.3411	1	Q9CWZ7	Gamma-soluble NSF attachment protein	Napg	mmu:108123				
625	-0.2402	-0.4381	-0.3447	-0.3410	1	Q91W96	Anaphase-promoting complex subunit 4	Anapc4	mmu:52206			1	
626	-0.3071	-0.4730	-0.2356	-0.3386	1	Q80UU2	Ribonuclease P protein subunit p38	Rpp38					
627	-0.3908	-0.3450	-0.2786	-0.3381	1	O55135	Eukaryotic translation initiation factor 6	Eif6	mmu:16418				
628	-0.6496	-0.0398	-0.3236	-0.3377	1	A0A0G2JDL9							
629	-0.0996	-0.5031	-0.4011	-0.3346	1	Q91ZX7	Prolow-density lipoprotein receptor-related protein 1;Low-density lipoprotein receptor-related pr	Lrp1	mmu:16971				
630	-0.0680	-0.4171	-0.5100	-0.3317	1	Q99P88	Nuclear pore complex protein Nup155	Nup155	mmu:170762				
631	-0.0353	-0.5276	-0.4264	-0.3298	1	Q99N16	Leukotriene-B(4) omega-hydroxylase 2	Cyp4f16;Cy	mmu:72054				
632	-0.3065	-0.0101	-0.6690	-0.3285	1	A2AIV2	Protein virilizer homolog	1110037F02Rik;Kiaa1429					
633	-0.6149	-0.0231	-0.3415	-0.3265	1	Q9R0Q9	Mannose-P-dolichol utilization defect 1 protein	Mpdu1					
634	-0.1537	-0.2125	-0.6123	-0.3262	1	Q8VHE0	Translocation protein SEC63 homolog	Sec63	mmu:140740				
635	-0.1506	-0.6870	-0.1354	-0.3243	1	P07214	SPARC	Sparc	mmu:20692				
636	-0.0174	-0.4819	-0.4712	-0.3235	1	Q9CZN7	Serine hydroxymethyltransferase	Shmt2	mmu:108037				
637	-0.0168	-0.5294	-0.4187	-0.3216	1	Q8R5J9	PRA1 family protein 3	Arl6ip5	mmu:65106	1			
638	-0.2966	-0.1761	-0.4889	-0.3205	1	Q8BGS2	BolA-like protein 2	Bola2	mmu:66162				
639	-0.5310	[illegible]	-0.4220	-0.3179	1	D3Z7Q2		Smim20	mmu:66278				
640	-0.2054	-0.2677	-0.4777	-0.3169	1	P24638	Lysosomal acid phosphatase	Acp2	mmu:11432		1		
641	-0.1351	-0.4579	-0.3550	-0.3160	1	Q80TM9	Nischarin	Nisch	mmu:64652				
642	-0.2276	-0.3740	-0.3431	-0.3149	1	Q6P6J9	Thioredoxin domain-containing protein 15	Txndc15	mmu:69672				
643	[illegible]	-0.7235	-0.2160	-0.3145	1	Q8BTG3	T-complex protein 11-like protein 1	Tcp11l1	mmu:320554				
644	-0.0242	-0.1257	-0.7908	-0.3136	1	Q9JHS4	ATP-dependent Clp protease ATP-binding subunit clpX-like, mitochondrial	Clpx	mmu:270166				
645	-0.1279	-0.0502	-0.7594	-0.3125	1	P70195	Proteasome subunit beta type-7	Psmb7	mmu:19177				
646	-0.5500	-0.2030	-0.1812	-0.3114	1	Q9DCN2	NADH-cytochrome b5 reductase 3;NADH-cytochrome b5 reductase 3 membrane-bound form;NAD	Cyb5r3	mmu:109754				
647	-0.3125	-0.3939	-0.2219	-0.3094	1	Q9Z2X8	Kelch-like ECH-associated protein 1	Keap1	mmu:50868			1	
648	-0.2816	-0.1553	-0.4898	-0.3089	1	Q9QXB9	Developmentally-regulated GTP-binding protein 2	Drg2	mmu:13495				
649	-0.2127	-0.2810	-0.4292	-0.3076	1	Q8BK64	Activator of 90 kDa heat shock protein ATPase homolog 1	Ahsa1	mmu:217737				
650	-0.5753	-0.0649	-0.2712	-0.3038	1	P62305	Small nuclear ribonucleoprotein E	Snrpe	mmu:20643				
651	-0.0592	-0.3684	-0.4664	-0.2980	1	E9QAT4		Sec16a	mmu:227648				
652	-0.0612	-0.4826	-0.3471	-0.2970	1	Q8BHC4	Dephospho-CoA kinase domain-containing protein	Dcakd	mmu:68087				
653	-0.2043	-0.0829	-0.5800	-0.2891	1	Q923W1	Trimethylguanosine synthase	Tgs1	mmu:116940				
654	-0.3376	-0.0475	-0.4818	-0.2890	1	Q9D7B7	Probable glutathione peroxidase 8	Gpx8	mmu:69590	1			
655	-0.2137	-0.6031	-0.0497	-0.2888	1	Q8R344	Coiled-coil domain-containing protein 12	Ccdc12	mmu:72654				
656	-0.4706	-0.0279	-0.3669	-0.2885	1	Q9CQM9	Glutaredoxin-3	Glrx3	mmu:30926				
657	-0.4953	-0.1066	-0.2481	-0.2833	1	Q8K1H1	Tudor domain-containing protein 7	Tdrd7	mmu:100121				
658	-0.0933	-0.4979	-0.2541	-0.2818	1	Q9CRA8	Exosome complex component RRP46	Exosc5	mmu:27998				
659	-0.3010	-0.4514	-0.0873	-0.2799	1	Q9D0J4	ADP-ribosylation factor-like protein 2	Arl2	mmu:56327				
660	-0.2908	-0.3868	-0.1604	-0.2793	1	Q99P72	Reticulon-4	Rtn4	mmu:68585				1
661	-0.1995	-0.1621	-0.4746	-0.2787	1	Q66JX5	FGFR1 oncogene partner	Fgfr1op	mmu:75296				
662	-0.1240	-0.3741	-0.3347	-0.2776	1	Q9D0A3	Arpin	Arpin	mmu:70420				
663	-0.2300	-0.0860	-0.5125	-0.2762	1	Q61102	ATP-binding cassette sub-family B member 7, mitochondrial	Abcb7	mmu:11306				
664	-0.1098	-0.1605	-0.5509	-0.2737	1	Q99PM9	Uridine-cytidine kinase 2	Uck2	mmu:80914				
665	-0.3277	[illegible]	-0.4899	-0.2736	1	O35114	Lysosome membrane protein 2	Scarb2	mmu:12492				
666	-0.4336	-0.0299	-0.3552	-0.2729	1	P51863	V-type proton ATPase subunit d 1	Atp6v0d1	mmu:11972				
667	-0.2465	-0.3485	-0.2188	-0.2713	1	O88325		Naglu	mmu:27419				
668	-0.2575	-0.2943	-0.2613	-0.2710	1	Q9CPR7	Suppressor of IKBKE 1	Sike1	mmu:66641				
669	-0.0485	-0.6947	-0.0693	-0.2708	1	Q8VDT9	39S ribosomal protein L50, mitochondrial	Mrpl50	mmu:28028				
670	-0.0287	-0.3520	-0.4307	-0.2705	1	P57784	U2 small nuclear ribonucleoprotein A	Snrpa1	mmu:68981				
671	-0.6520	-0.0296	-0.1285	-0.2700	1	Q9JKP7	DNA polymerase epsilon subunit 3	Pole3	mmu:59001				
672	-0.1009	-0.5367	-0.1702	-0.2693	1	Q9Z2I0	LETM1 and EF-hand domain-containing protein 1, mitochondrial	Letm1	mmu:56384				
673	-0.1180	-0.5864	-0.0947	-0.2664	1	Q64701	Retinoblastoma-like protein 1	Rbl1	mmu:19650				
674	-0.1714	-0.2946	-0.3306	-0.2655	1	Q3U186	Probable arginine--tRNA ligase, mitochondrial	Rars2	mmu:109093				
675	-0.0871	-0.0529	-0.6478	-0.2626	1	Q8BWB6	Metalloreductase STEAP2	Steap2	mmu:74051				
676	-0.0642	-0.1127	-0.6079	-0.2616	1	P97366	Ecotropic viral integration site 5 protein	Evi5					
677	-0.4558	-0.3176	[illegible]	-0.2587	1	Q71LX4	Talin-2	Tln2	mmu:70549				
678	-0.2287	-0.3856	-0.1525	-0.2556	1	Q810B6	Rabankyrin-5	Ankfy1	mmu:11736				
679	-0.3869	-0.0152	-0.3502	-0.2508	1	Q8BLH7	HIRA-interacting protein 3	Hirip3	mmu:233876				
680	-0.3434	-0.0555	-0.3517	-0.2502	1	P38647	Stress-70 protein, mitochondrial	Hspa9	mmu:15526				1
681	-0.3518	-0.3207	-0.0730	-0.2485	1	Q3U3T8	WD repeat-containing protein 62	Wdr62					
682	-0.3804	-0.0266	-0.3370	-0.2480	1	Q0GNC1	Inverted formin-2	Inf2	mmu:70435				
683	-0.0079	-0.4734	-0.2603	-0.2472	1	Q9Z2D3	Non-syndromic hearing impairment protein 5 homolog	Dfna5	mmu:54722				
684	-0.4639	-0.0366	-0.2359	-0.2455	1	Q9CZW4	Long-chain-fatty-acid--CoA ligase 3	Acsl3	mmu:74205				
685	-0.0156	-0.0496	-0.6698	-0.2450	1	P27046	Alpha-mannosidase 2	Man2a1	mmu:17158				
686	-0.5242	-0.0359	-0.1683	-0.2428	1	Q52KE7	Cyclin-L1	Ccnl1	mmu:56706				
687	-0.4409	-0.0328	-0.2485	-0.2407	1	Q9D1G1	Ras-related protein Rab-1B	Rab1b	mmu:76308				
688	-0.0608	-0.1760	-0.4832	-0.2400	1	Q8VDW0	ATP-dependent RNA helicase DDX39A	Ddx39a	mmu:68278				
689	-0.3126	-0.1546	-0.2525	-0.2399	1	Q6P5F9	Exportin-1	Xpo1	mmu:103573				
690	-0.4707	-0.0172	-0.2224	-0.2368	1	P51912	Amino acid transporter;Neutral amino acid transporter B(0)	Slc1a5					
691	-0.1595	-0.2484	-0.3021	-0.2367	1	Q91YH5	Atlastin-3	Atl3	mmu:109168				
692	-0.1579	-0.5068	-0.0429	-0.2359	1	Q9JLZ6	Hypermethylated in cancer 2 protein	Hic2	mmu:58180				
693	-0.1717	-0.1161	-0.4195	-0.2358	1	Q8JZN5	Acyl-CoA dehydrogenase family member 9, mitochondrial	Acad9	mmu:229211				
694	-0.0982	-0.3639	-0.2438	-0.2353	1	Q920A5	Retinoid-inducible serine carboxypeptidase	Scpep1	mmu:74617				
695	-0.3005	-0.1177	-0.2875	-0.2352	1	A2AGT5	Cytoskeleton-associated protein 5	Ckap5	mmu:75786				
696	-0.2187	-0.1813	-0.2997	-0.2332	1	P97822	Acidic leucine-rich nuclear phosphoprotein 32 family member E	Anp32e	mmu:66471				
697	-0.6530	-0.0262	-0.0169	-0.2320	1	P15116	Cadherin-2	Cdh2	mmu:12558				
698	-0.1897	-0.0868	-0.4143	-0.2303	1	Q00560	Interleukin-6 receptor subunit beta	Il6st	mmu:16195				
699	-0.1744	-0.2366	-0.2757	-0.2289	1	Q9CQT1	Methylthioribose-1-phosphate isomerase	Mri1	mmu:67873				
700	-0.0413	-0.2242	-0.4210	-0.2288	1	Q9CZE3	Ras-related protein Rab-32	Rab32	mmu:67844				
701	-0.2298	-0.0116	-0.4439	-0.2284	1	O54692	Centromere/kinetochore protein zw10 homolog	Zw10	mmu:26951				
702	-0.0746	-0.0944	-0.5159	-0.2283	1	O89051	Integral membrane protein 2B;BRI2, membrane form;BRI2 intracellular domain;BRI2C, soluble for	Itm2b	mmu:16432				
703	-0.0168	-0.6506	-0.0087	-0.2254	1	Q61171	Peroxiredoxin-2	Prdx2	mmu:21672	1			1
704	-0.5409	-0.0528	-0.0810	-0.2249	1	B9EJ54		Nup205	mmu:70699				
705	-0.4474	-0.0312	-0.1924	-0.2237	1	O70572	Sphingomyelin phosphodiesterase 2	Smpd2	mmu:20598				
706	-0.1460	-0.4451	-0.0759	-0.2223	1	Q8QZV7	Protein asunder homolog	Asun	mmu:71177				
707	-0.2535	-0.2197	-0.1857	-0.2196	1	Q9DB25	Dolichyl-phosphate beta-glucosyltransferase	Alg5	mmu:66248				
708	-0.1346	-0.0468	-0.4741	-0.2185	1	Q63850	Nuclear pore glycoprotein p62	Nup62	mmu:18226				
709	-0.2465	-0.1512	-0.2546	-0.2174	1	Q99NB9	Splicing factor 3B subunit 1	Sf3b1					
710	-0.0568	-0.1264	-0.4559	-0.2130	1	P52431	DNA polymerase delta catalytic subunit;DNA polymerase	Pold1	mmu:18971				
711	-0.3154	-0.1050	-0.2181	-0.2128	1	Q99JI6	Ras-related protein Rap-1b	Rap1b	mmu:215449				
712	-0.1953	-0.3907	-0.0456	-0.2105	1	P35486	Pyruvate dehydrogenase E1 component subunit alpha, somatic form, mitochondrial	Pdha1	mmu:18597				
713	-0.3328	-0.2149	-0.0774	-0.2084	1	Q3UQN2	F-BAR domain only protein 2	Fcho2	mmu:218503				
714	-0.1624	-0.1725	-0.2895	-0.2081	1	Q9ERI6	Retinol dehydrogenase 14	Rdh14	mmu:105014				
715	-0.2740	-0.2939	-0.0534	-0.2071	1	Q8VE37	Regulator of chromosome condensation	Rcc1	mmu:100088				
716	-0.1477	-0.1015	-0.3636	-0.2043	1	Q921F2	TAR DNA-binding protein 43	Tardbp	mmu:230908				
717	-0.4347	-0.1333	-0.0414	-0.2031	1	Q8R060	Protein zwilch homolog	Zwilch	mmu:68014				
718	-0.2037	-0.0968	-0.3072	-0.2026	1	Q99JB2	Stomatin-like protein 2, mitochondrial	Stoml2	mmu:66592				
719	-0.5174	-0.0324	-0.0452	-0.1983	1	B9EJR8	Dynein assembly factor 5, axonemal	Dnaaf5	mmu:433956				
720	-0.1617	-0.2023	-0.2261	-0.1967	1	Q91YU6	Leucine zipper putative tumor suppressor 2	Lzts2	mmu:226154				
721	-0.0386	-0.3106	-0.2387	-0.1960	1	A2A6Q5	Cell division cycle protein 27 homolog	Cdc27	mmu:217232			1	
722	-0.2020	-0.2568	-0.1241	-0.1943	1	Q924C1	Exportin-5	Xpo5	mmu:72322				
723	-0.2060	-0.1196	-0.2440	-0.1899	1	Q8JZR0	Long-chain-fatty-acid--CoA ligase 5	Acsl5	mmu:433256				
724	-0.1485	-0.3275	-0.0822	-0.1861	1	Q8CHP8	Phosphoglycolate phosphatase	Pgp	mmu:67078		1		
725	-0.0589	-0.2901	-0.1863	-0.1784	1	Q99LI2	Chloride channel CLIC-like protein 1	Clcc1	mmu:229725				
726	-0.1598	-0.3548	-0.0176	-0.1774	1	Q6R891	Neurabin-2	Ppp1r9b	mmu:217124				
727	-0.1500	-0.3485	-0.0251	-0.1745	1	Q8BWZ3	N-alpha-acetyltransferase 25, NatB auxiliary subunit	Naa25	mmu:231713				
728	-0.2038	-0.1952	-0.1136	-0.1709	1	P10639	Thioredoxin	Txn	mmu:22166	1			
729	-0.2248	-0.2340	-0.0518	-0.1702	1	Q61335	B-cell receptor-associated protein 31	Bcap31	mmu:27061				
730	-0.1410	-0.2841	-0.0850	-0.1700	1	P62257	Ubiquitin-conjugating enzyme E2 H	Ube2h	mmu:22214			1	
731	-0.2406	-0.2365	-0.0229	-0.1667	1	Q9D0F3	Protein ERGIC-53	Lman1	mmu:70361				
732	-0.2702	-0.0926	-0.1328	-0.1652	1	F7BJ98		Nbeal1					
733	-0.1392	-0.2196	-0.1357	-0.1648	1	Q8CGK3	Lon protease homolog, mitochondrial	Lonp1	mmu:74142	1			
734	-0.1751	-0.0750	-0.2391	-0.1631	1	Q6PDI5	Proteasome-associated protein ECM29 homolog	Ecm29;AI3	mmu:230249				
735	-0.0577	-0.1172	-0.3094	-0.1614	1	Q8N9S3	Activator of 90 kDa heat shock protein ATPase homolog 2	Ahsa2	mmu:268390				
736	-0.3331	-0.0151	-0.1289	-0.1590	1	P62075	Mitochondrial import inner membrane translocase subunit Tim13	Timm13	mmu:30055				
737	-0.1535	-0.2131	-0.1068	-0.1578	1	O55106	Striatin	Strn	mmu:268980				
738	-0.3372	-0.1100	-0.0259	-0.1577	1	P97386	DNA ligase;DNA ligase 3	Lig3					
739	-0.0437	-0.2447	-0.1777	-0.1554	1	Q8VDM4	26S proteasome non-ATPase regulatory subunit 2	Psmd2;Gm	mmu:21762				
740	-0.0507	-0.2004	-0.2010	-0.1507	1	Q9JJ94	Sjoegren syndrome nuclear autoantigen 1 homolog	Ssna1	mmu:68475				
741	-0.0154	-0.2101	-0.2249	-0.1501	1	Q7TNP2	Serine/threonine-protein phosphatase 2A 65 kDa regulatory subunit A beta isoform	Ppp2r1b	mmu:73699				1
742	-0.0239	-0.0079	-0.4164	-0.1494	1	Q6PDQ2	Chromodomain-helicase-DNA-binding protein 4	Chd4	mmu:107932				
743	-0.1506	-0.2388	-0.0526	-0.1473	1	Q6NZC7	SEC23-interacting protein	Sec23ip					
744	-0.0715	-0.2077	-0.1527	-0.1440	1	Q3TIU4	2,5-phosphodiesterase 12	Pde12	mmu:211948				
745	-0.1789	-0.0749	-0.1766	-0.1435	1	Q99PV0	Pre-mRNA-processing-splicing factor 8	Prpf8	mmu:192159				

746	-0.1189	-0.0881	-0.2220	-0.1430	1	Q3U7R1	Extended synaptotagmin-1	Esyt1	mmu:23943				
747	-0.0358	-0.0098	-0.3762	-0.1406	1	P17427	AP-2 complex subunit alpha-2	Ap2a2	mmu:11772				
748	-0.1665	-0.0937	-0.1501	-0.1368	1	A8C756	Thyroid adenoma-associated protein homolog	Thada	mmu:240174				
749	-0.1471	-0.0659	-0.1868	-0.1333	1	Q8BH50	Uncharacterized protein C18orf25 homolog	8030462N	mmu:212163				
750	-0.2814	-0.0187	-0.0813	-0.1271	1	Q9D1D4	Transmembrane emp24 domain-containing protein 10	Tmed10	mmu:68581				
751	-0.0420	-0.2354	-0.1008	-0.1261	1	P62835	Ras-related protein Rap-1A	Rap1a	mmu:109905				
752	-0.1184	-0.1573	-0.0963	-0.1240	1	Q80X95	Ras-related GTP-binding protein A;Ras-related GTP-binding protein B	Rraga;Rrag	mmu:68441				
753	-0.1159	-0.0595	-0.1787	-0.1180	1	Q9Z120	tRNA (guanine-N(7)-)-methyltransferase	Mettl1	mmu:17299				
754	-0.1632	-0.1028	-0.0648	-0.1103	1	Q9CZ42	ATP-dependent (S)-NAD(P)H-hydrate dehydratase	Carkd	mmu:69225				
755	-0.1252	-0.1659	-0.0224	-0.1045	1	Q60864	Stress-induced-phosphoprotein 1	Stip1	mmu:20867				
756	-0.1691	-0.0796	-0.0584	-0.1024	1	Q9QYF9	Protein NDRG3	Ndrg3	mmu:29812				
757	-0.1313	-0.0234	-0.1501	-0.1016	1	O35648	Centrin-3	Cetn3	mmu:12626				
758	-0.1509	-0.1229	-0.0199	-0.0979	1	Q91YJ2	Sorting nexin-4	Snx4	mmu:69150				
759	-0.0285	-0.0469	-0.2053	-0.0936	1	Q8BG32	26S proteasome non-ATPase regulatory subunit 11	Psmd11	mmu:69077				
760	-0.1088	-0.0150	-0.1338	-0.0859	1	Q3U1J4	DNA damage-binding protein 1	Ddb1	mmu:13194			1	
761	-0.1270	[illegible]	-0.1203	-0.0839	1	Q8BKE6	Cytochrome P450 20A1	Cyp20a1	mmu:77951				
762	-0.0271	-0.0469	-0.1679	-0.0806	1	Q62318	Transcription intermediary factor 1-beta	Trim28	mmu:21849				
763	-0.1500	-0.0291	-0.0582	-0.0791	1	O35841	Apoptosis inhibitor 5	Api5	mmu:11800				1
764	-0.0337	-0.1484	-0.0511	-0.0777	1	Q99L04	Dehydrogenase/reductase SDR family member 1	Dhrs1	mmu:52585				
765	-0.0169	-0.0771	-0.1156	-0.0699	1	Q9Z1F9	SUMO-activating enzyme subunit 2	Uba2	mmu:50995				
766	-0.0314	-0.0474	-0.1287	-0.0692	1	Q9D7S9	Charged multivesicular body protein 5	Chmp5	mmu:76959				
767	-0.0702	-0.0630	-0.0494	-0.0609	1	Q8BL66	Early endosome antigen 1	Eea1	mmu:216238				
768	[illegible]	[illegible]	-0.1438	-0.0488	1	O70456	14-3-3 protein sigma	Sfn	mmu:55948			1	1
769	0.0356	0.1107	0.0474	0.0646	1	A2A4P0	ATP-dependent RNA helicase DHX8	Dhx8	mmu:217207				
770	0.0764	0.0278	0.0965	0.0669	1	Q569Z5	Probable ATP-dependent RNA helicase DDX46	Ddx46	mmu:212880				
771	0.1097	0.0353	0.0691	0.0714	1	Q91YR5	Methyltransferase-like protein 13	Mettl13	mmu:71449				
772	0.0159	0.0306	0.1900	0.0788	1	Q6NZJ6	Eukaryotic translation initiation factor 4 gamma 1	Eif4g1	mmu:208643				
773	0.1456	0.0644	0.0487	0.0862	1	Q8C0V9	FERM domain-containing protein 6	Frmd6	mmu:319710				
774	0.0804	0.1048	0.0748	0.0867	1	Q8CIG9	F-box/LRR-repeat protein 8	Fbxl8	mmu:50788				
775	[illegible]	0.2482	0.0270	0.0931	1	Q8BMP6	Golgi resident protein GCP60	Acbd3	mmu:170760				
776	0.0143	0.1163	0.1633	0.0980	1	P55012	Solute carrier family 12 member 2	Slc12a2	mmu:20496				
777	0.1125	0.1239	0.0748	0.1037	1	Q05186	Reticulocalbin-1	Rcn1	mmu:19672				
778	[illegible]	0.0579	0.2588	0.1065	1	Q99L48	60S ribosomal export protein NMD3	Nmd3	mmu:97112				
779	0.0835	0.0113	0.2472	0.1140	1	Q8C0D5	Elongation factor Tu GTP-binding domain-containing protein 1	Eftud1	mmu:101592				
780	0.2687	0.0271	0.0628	0.1195	1	Q9WTM5	RuvB-like 2	Ruvbl2	mmu:20174				
781	0.0535	0.0187	0.2951	0.1224	1	B2RXW8		Ppfia1	mmu:233977				
782	0.1688	0.2173	[illegible]	0.1297	1	O88487	Cytoplasmic dynein 1 intermediate chain 2	Dync1i2	mmu:13427				
783	0.1043	0.2058	0.1013	0.1371	1	Q8R3C0	Mini-chromosome maintenance complex-binding protein	Mcmbp	mmu:210711				
784	0.2661	0.0239	0.1442	0.1447	1	E9Q634	Unconventional myosin-Ie	Myo1e	mmu:71602				
785	0.0944	0.2368	0.1122	0.1478	1	Q91XI1	tRNA-dihydrouridine(47) synthase [NAD(P)(+)]-like	Dus3l	mmu:224907				
786	0.1202	0.1758	0.1487	0.1482	1	Q0VGB7	Serine/threonine-protein phosphatase 4 regulatory subunit 2	Ppp4r2	mmu:232314				
787	[illegible]	0.3234	0.1286	0.1509	1	G3X972		Sec24c					
788	0.0570	0.1532	0.2443	0.1515	1	P47757	F-actin-capping protein subunit beta	Capzb	mmu:12345				
789	0.0531	0.0915	0.3227	0.1558	1	Q3TMV7	Pyridine nucleotide-disulfide oxidoreductase domain-containing protein 1	Pyroxd1	mmu:232491	1			
790	0.2765	0.1457	0.0508	0.1577	1	P28740	Kinesin-like protein KIF2A	Kif2a	mmu:16563				
791	0.3250	[illegible]	0.1537	0.1609	1	P42932	T-complex protein 1 subunit theta	Cct8	mmu:12469				
792	0.0923	0.2073	0.1860	0.1619	1	Q78HU3	Multivesicular body subunit 12A	Mvb12a	mmu:73711				
793	0.0577	0.1637	0.2753	0.1656	1	Q8CII2	Cell division cycle protein 123 homolog	Cdc123	mmu:98828				
794	0.2568	0.1659	0.0785	0.1671	1	Q61316	Heat shock 70 kDa protein 4	Hspa4					
795	0.0529	0.2166	0.2370	0.1688	1	P62908	40S ribosomal protein S3	Rps3	mmu:27050	1			
796	0.0880	0.0742	0.3474	0.1699	1	Q9WV98	Mitochondrial import inner membrane translocase subunit Tim9	Timm9	mmu:30056				
797	0.3511	0.1023	0.0568	0.1701	1	Q61337	Bcl2-associated agonist of cell death	Bad	mmu:12015	1			1
798	0.0092	0.3358	0.1673	0.1708	1	Q91WC0	Histone-lysine N-methyltransferase setd3	Setd3	mmu:52690				
799	0.3509	0.0982	0.0920	0.1804	1	P84089	Enhancer of rudimentary homolog	Erh	mmu:13877				
800	0.0954	0.2376	0.2100	0.1810	1	Q8R5K4	Nucleolar protein 6	Nol6	mmu:230082				
801	0.1153	0.1295	0.3003	0.1817	1	Q8BY02	NF-kappa-B-repressing factor	Nkrf	mmu:77286				
802	0.1540	0.0905	0.3027	0.1824	1	Q9CZ30	Obg-like ATPase 1	Ola1	mmu:67059				
803	0.1752	0.0701	0.3057	0.1837	1	Q9D1C2	Protein chibby homolog 1	Cby1	mmu:73739				
804	0.2953	0.2334	0.0305	0.1864	1	Q9EQU5	Protein SET	Set	mmu:56086				
805	0.1787	0.2954	0.1176	0.1972	1	Q9CZ44	NSFL1 cofactor p47	Nsfl1c	mmu:386649				
806	0.1769	0.3809	0.0340	0.1973	1	Q7TMK9	Heterogeneous nuclear ribonucleoprotein Q	Syncrip	mmu:56403				
807	0.3383	0.0786	0.2017	0.2062	1	Q91VT1	E3 SUMO-protein ligase NSE2	Nsmce2	mmu:68501				
808	0.3153	0.2120	0.1118	0.2130	1	Q8K0C4	Lanosterol 14-alpha demethylase	Cyp51a1	mmu:13121				
809	0.1540	0.2530	0.2333	0.2134	1	Q8BFS6	Serine/threonine-protein phosphatase CPPED1	Cpped1	mmu:223978		1		
810	0.1830	0.0783	0.3814	0.2142	1	Q9DBT5	AMP deaminase 2	Ampd2	mmu:109674				
811	0.0962	0.3451	0.2043	0.2152	1	Q91WE2	Protein FAM192A	Fam192a	mmu:102122				
812	0.2255	0.3071	0.1163	0.2163	1	P58021	Transmembrane 9 superfamily member 2	Tm9sf2	mmu:68059				
813	0.1397	0.0471	0.4652	0.2173	1	Q9DBR3	Armadillo repeat-containing protein 8	Armc8	mmu:74125				
814	0.1923	[illegible]	0.4608	0.2197	1	P58252	Elongation factor 2	Eef2	mmu:13629	1			
815	0.2479	0.0656	0.3470	0.2202	1	Q8BTM8	Filamin-A	Flna					
816	0.2320	0.2403	0.1925	0.2216	1	Q8BU31	Ras-related protein Rap-2c	Rap2c	mmu:72065				
817	0.2326	0.2405	0.2016	0.2249	1	Q69ZW3	EH domain-binding protein 1	Ehbp1	mmu:216565				
818	0.4028	0.0595	0.2272	0.2298	1	Q9QY06	Unconventional myosin-IXb	Myo9b					
819	0.2780	0.1032	0.3234	0.2349	1	Q6P1F6	Serine/threonine-protein phosphatase 2A 55 kDa regulatory subunit B alpha isoform	Ppp2r2a	mmu:71978				
820	0.0624	0.0125	0.6376	0.2375	1	P62751	60S ribosomal protein L23a	Rpl23a	mmu:268449				
821	0.2310	0.1194	0.3705	0.2403	1	Q9R1P0	Proteasome subunit alpha type-4	Psma4	mmu:26441				
822	0.2334	0.2226	0.2722	0.2427	1	Q00612	Glucose-6-phosphate 1-dehydrogenase X;Glucose-6-phosphate 1-dehydrogenase	G6pdx	mmu:14381	1			
823	0.2858	0.1400	0.3047	0.2435	1	Q9JLB0	MAGUK p55 subfamily member 6	Mpp6	mmu:56524				
824	0.1929	0.2052	0.3615	0.2532	1	Q9QYF1	Retinol dehydrogenase 11	Rdh11	mmu:17252				
825	0.3740	0.2950	0.1031	0.2574	1	Q8BP27	Swi5-dependent recombination DNA repair protein 1 homolog	Sfr1	mmu:67788				
826	0.1381	0.3565	0.2918	0.2621	1	Q8R1B4	Eukaryotic translation initiation factor 3 subunit C	Eif3c	mmu:56347				
827	0.2868	0.2444	0.2635	0.2649	1	P49962	Signal recognition particle 9 kDa protein	Srp9	mmu:27058				
828	0.4052	0.2937	0.0961	0.2650	1	Q9CWL8	Beta-catenin-like protein 1	Ctnnbl1	mmu:66642				
829	0.0303	0.1096	0.6552	0.2650	1	F8WI14		Ecm1					
830	0.1440	0.1930	0.4614	0.2661	1	Q9Z108	Double-stranded RNA-binding protein Staufen homolog 1	Stau1	mmu:20853				
831	0.1963	0.6028	0.0076	0.2689	1	Q8C4Q6	Axin interactor, dorsalization-associated protein	Aida	mmu:108909				
832	0.4714	0.1383	0.1990	0.2696	1	P07724	Serum albumin	Alb	mmu:11657	1			
833	0.2018	0.5470	0.0617	0.2702	1	Q9CWJ9	Bifunctional purine biosynthesis protein PURH;Phosphoribosylaminoimidazolecarboxamide formy	Atic	mmu:108147				
834	0.0831	0.0126	0.7174	0.2710	1	Q9QUI0	Transforming protein RhoA	Rhoa	mmu:11848				
835	0.0835	0.2983	0.4391	0.2736	1	Q9DCX2	ATP synthase subunit d, mitochondrial	Gm10250;	mmu:71679				
836	0.3187	0.3325	0.1710	0.2741	1	Q9D0N7	Chromatin assembly factor 1 subunit B	Chaf1b	mmu:110749				
837	0.2642	0.3151	0.2472	0.2755	1	Q02614	SAP30-binding protein	Sap30bp	mmu:57230				1
838	0.0097	0.0261	0.7970	0.2776	1	Q923D4	Splicing factor 3B subunit 5	Sf3b5	mmu:66125				
839	0.3786	0.2908	0.1673	0.2789	1	Q9R0M6	Ras-related protein Rab-9A	Rab9a;Rab	mmu:56382				
840	0.0731	0.2787	0.4855	0.2791	1	P39447	Tight junction protein ZO-1	Tjp1	mmu:21872				
841	0.0622	0.4343	0.3467	0.2811	1	P29341	Polyadenylate-binding protein 1	Pabpc1	mmu:18458				
842	0.4618	0.2944	0.0900	0.2821	1	Q9R1P4	Proteasome subunit alpha type-1	Psma1	mmu:26440				
843	0.2226	0.1870	0.4382	0.2826	1	Q922D8	C-1-tetrahydrofolate synthase, cytoplasmic;Methylenetetrahydrofolate dehydrogenase;Methenylt	Mthfd1	mmu:108156				
844	0.5177	0.1966	0.1382	0.2842	1	A2ARP1	Inositol hexakisphosphate and diphosphoinositol-pentakisphosphate kinase 1	Ppip5k1	mmu:327655		1		
845	0.1671	0.4702	0.2188	0.2854	1	P26443	Glutamate dehydrogenase 1, mitochondrial	Glud1	mmu:14661				
846	0.0562	0.1298	0.6724	0.2861	1	Q8BUR4	Dedicator of cytokinesis protein 1	Dock1	mmu:330662				
847	0.2093	0.4551	0.1995	0.2880	1	P62878	E3 ubiquitin-protein ligase RBX1;E3 ubiquitin-protein ligase RBX1, N-terminally processed	Rbx1	mmu:56438			1	
848	0.2699	0.5291	0.0711	0.2900	1	O35382	Exocyst complex component 4	Exoc4	mmu:20336				
849	0.4392	0.3627	0.0683	0.2901	1	Q5U430	E3 ubiquitin-protein ligase UBR3	Ubr3	mmu:68795			1	
850	0.3330	0.2087	0.3420	0.2946	1	O70251	Elongation factor 1-beta	Eef1b2;Eef	mmu:55949				
851	0.0791	0.1526	0.6552	0.2956	1	Q99JI4	26S proteasome non-ATPase regulatory subunit 6	Psmd6	mmu:66413				
852	0.5930	0.2232	0.0722	0.2961	1	P45878	Peptidyl-prolyl cis-trans isomerase FKBP2	Fkbp2	mmu:14227				
853	0.2346	0.3955	0.2676	0.2992	1	Q9JIF0	Protein arginine N-methyltransferase 1	Prmt1	mmu:15469				
854	0.3416	0.3070	0.2566	0.3017	1	Q922B6	E3 ubiquitin-protein ligase TRAF7	Traf7				1	1
855	0.2489	0.5475	0.1228	0.3064	1	P10711	Transcription elongation factor A protein 1	Tcea1	mmu:21399				
856	0.5666	0.2762	0.0914	0.3114	1	P57791	CAAX prenyl protease 2	Rce1	mmu:19671				
857	0.0406	0.1835	0.7150	0.3130	1	Q99PT1	Rho GDP-dissociation inhibitor 1	Arhgdia	mmu:192662				
858	0.2211	0.1724	0.5554	0.3163	1	P51855	Glutathione synthetase	Gss	mmu:14854	1			
859	0.3792	0.2448	0.3260	0.3167	1	Q6Y7W8	PERQ amino acid-rich with GYF domain-containing protein 2	Gigyf2	mmu:227331				
860	0.0470	0.3525	0.5512	0.3169	1	Q8BIE6	FERM domain-containing protein 4A	Frmd4a	mmu:209630				
861	0.1865	0.2055	0.5599	0.3173	1	P48722	Heat shock 70 kDa protein 4L	Hspa4l	mmu:18415				
862	0.0776	0.1913	0.7034	0.3241	1	Q923D2	Flavin reductase (NADPH)	Blvrb	mmu:233016				
863	0.3050	0.1405	0.5286	0.3247	1	P59759	MKL/myocardin-like protein 2	Mkl2	mmu:239719				
864	0.2647	0.3581	0.3541	0.3256	1	Q6A0A9	Constitutive coactivator of PPAR-gamma-like protein 1	FAM120A	mmu:218236				
865	0.0923	0.3308	0.5569	0.3267	1	G5E870	E3 ubiquitin-protein ligase TRIP12	Trip12	mmu:14897			1	
866	0.4039	0.2207	0.3630	0.3292	1	Q60902	Epidermal growth factor receptor substrate 15-like 1	Eps15l1	mmu:13859				
867	0.1785	0.1272	0.6978	0.3345	1	P11157	Ribonucleoside-diphosphate reductase subunit M2	Rrm2	mmu:20135				
868	0.2102	0.5292	0.2658	0.3351	1	Q8JZQ9	Eukaryotic translation initiation factor 3 subunit B	Eif3b	mmu:27979				
869	0.3572	0.3221	0.3387	0.3393	1	Q3TCH7	Cullin-4A	Cul4a	mmu:99375			1	1
870	0.4789	0.1170	0.4337	0.3432	1	Q8VEH6	COBW domain-containing protein 1	Cbwd1	mmu:226043				
871	0.2606	0.6610	0.1106	0.3441	1	Q8C5L3	CCR4-NOT transcription complex subunit 2	Cnot2	mmu:72068				

872	0.0409	0.5982	0.3931	0.3441	1	P62307	Small nuclear ribonucleoprotein F	Snrpf	mmu:69878				
873	0.0713	0.0417	0.9296	0.3475	1	B2RUE8		Map4k4					
874	0.3472	0.2838	0.4220	0.3510	1	P61327	Protein mago nashi homolog	Magoh	mmu:17149				
875	0.2013	0.4799	0.3738	0.3517	1	Q6PDH0	Pleckstrin homology-like domain family B member 1	Phldb1	mmu:102693				
876	0.0845	0.5736	0.4060	0.3547	1	Q6PAJ1	Breakpoint cluster region protein	Bcr	mmu:110279				
877	0.3008	0.2073	0.5562	0.3548	1	P62270	40S ribosomal protein S18	Gm10260;R	mmu:20084				
878	0.0057	0.1564	0.9153	0.3591	1	Q8VBW1	Transporter;Sodium- and chloride-dependent creatine transporter 1	Slc6a8	mmu:102857				
879	0.3926	0.5005	0.1912	0.3614	1	Q3U2P1	Protein transport protein Sec24A	Sec24a	mmu:77371				
880	0.7673	0.1880	0.1477	0.3677	1	Q9CPY7	Cytosol aminopeptidase	Lap3	mmu:66988				
881	0.2933	0.2894	0.5223	0.3683	1	P51410	60S ribosomal protein L9	Rpl9	mmu:20005				
882	0.6847	0.1795	0.2431	0.3691	1	P70444	BH3-interacting domain death agonist;BH3-interacting domain death agonist p15;BH3-interacting	Bid	mmu:12122				1
883	0.4616	0.5383	0.1096	0.3698	1	Q9ERG2	Striatin-3	Strn3	mmu:94186				
884	0.2249	0.4973	0.4008	0.3743	1	Q9D358	Low molecular weight phosphotyrosine protein phosphatase	Acp1	mmu:11431		1		
885	0.1122	0.9538	0.0601	0.3754	1	O70252	Heme oxygenase 2	Hmox2	mmu:15369	1			
886	0.0827	0.7630	0.2935	0.3797	1	P62889	60S ribosomal protein L30	Rpl30	mmu:19946				
887	0.3406	0.3574	0.4422	0.3801	1	P97798	Neogenin	Neo1					
888	0.1557	0.1828	0.8037	0.3807	1	Q8BGD9	Eukaryotic translation initiation factor 4B	Eif4b	mmu:75705				
889	0.1210	0.5032	0.5293	0.3845	1	Q61792	LIM and SH3 domain protein 1	Lasp1	mmu:16796				
890	0.0142	0.9629	0.1817	0.3863	1	O70378	ER membrane protein complex subunit 8	Emc8	mmu:18117				
891	0.0109	0.7181	0.4520	0.3937	1	Q9CPR1	RWD domain-containing protein 4	Rwdd4;Rw	mmu:192174				
892	0.4597	0.1150	0.6103	0.3950	1	Q9CW79	Golgin subfamily A member 1	Golga1	mmu:76899				
893	0.2239	0.5607	0.4110	0.3985	1	Q5BU09	E2F-associated phosphoprotein	Eapp	mmu:66266				
894	0.6745	0.2427	0.2791	0.3988	1	Q8K2T4	Ubiquinol-cytochrome-c reductase complex assembly factor 3	Uqcc3	mmu:107197				
895	0.4645	0.3834	0.3533	0.4004	1	Q60972	Histone-binding protein RBBP4	Rbbp4	mmu:19646				
896	0.1790	0.2486	0.7793	0.4023	1	Q8K019	Bcl-2-associated transcription factor 1	Bclaf1	mmu:72567				1
897	0.4550	0.5600	0.2096	0.4082	1	Q6PDL0	Cytoplasmic dynein 1 light intermediate chain 2	Dync1li2	mmu:234663				
898	0.3909	0.4156	0.4209	0.4091	1	Q6PGF7	Exocyst complex component 8	Exoc8	mmu:102058				
899	1.0058	0.0474	0.1752	0.4095	1	Q91WZ8	Dysbindin	Dtnbp1	mmu:94245				
900	0.0101	0.5915	0.6322	0.4113	1	Q920B9	FACT complex subunit SPT16	Supt16;Sup	mmu:114741				
901	0.5856	0.4566	0.1922	0.4115	1	Q60973	Histone-binding protein RBBP7	Rbbp7	mmu:245688				
902	0.2038	0.2737	0.7581	0.4119	1	Q7TMB8	Cytoplasmic FMR1-interacting protein 1;Cytoplasmic FMR1-interacting protein 2	Cyfip1;Cyfi	mmu:20430				
903	1.1059	0.0602	0.0705	0.4122	1	Q31125	Zinc transporter SLC39A7	Slc39a7	mmu:14977				
904	0.2059	0.7392	0.3180	0.4210	1	Q8C1B7	Septin-11	Sept11	mmu:52398				
905	0.2324	0.5521	0.4800	0.4215	1	P34022	Ran-specific GTPase-activating protein	Ranbp1	mmu:19385				
906	0.2362	0.5587	0.4748	0.4232	1	Q9CR51	V-type proton ATPase subunit G 1	Atp6v1g1	mmu:66290				
907	0.4724	0.2450	0.5535	0.4236	1	O55131	Septin-7	Sept7	mmu:235072				
908	0.4413	0.0075	0.8280	0.4256	1	O88597	Beclin-1	Becn1	mmu:56208	1			1
909	0.0102	0.9842	0.2856	0.4267	1	Q61584	Fragile X mental retardation syndrome-related protein 1	Fxr1	mmu:14359				
910	0.3985	0.8118	0.0713	0.4272	1	Q6NVE8	WD repeat-containing protein 44	Wdr44	mmu:72404				
911	0.3662	0.7197	0.2124	0.4328	1	Q504M2		Pdp2	mmu:382051				
912	0.6115	0.1126	0.5759	0.4333	1	Q71FD5	E3 ubiquitin-protein ligase ZNRF2	Znrf2	mmu:387524			1	
913	0.4769	0.4334	0.3901	0.4335	1	Q9WUU8	TNFAIP3-interacting protein 1	Tnip1	mmu:57783				
914	0.5126	0.0593	0.7286	0.4335	1	Q91V04	Translocating chain-associated membrane protein 1	Tram1	mmu:72265				
915	0.1294	0.7238	0.4479	0.4337	1	Q9DAU1	Protein canopy homolog 3	Cnpy3	mmu:72029				
916	0.3499	0.6166	0.3528	0.4398	1	Q8R409	Protein HEXIM1	Hexim1	mmu:192231				
917	0.2868	0.1253	0.9082	0.4401	1	Q62179	Semaphorin-4B	Sema4b	mmu:20352				
918	0.0174	0.6908	0.6162	0.4415	1	P63325	40S ribosomal protein S10	Rps10	mmu:67097				
919	0.2159	0.6228	0.4868	0.4418	1	Q9CR29	Coiled-coil domain-containing protein 43	Ccdc43	mmu:52715				
920	0.0506	0.7615	0.5254	0.4458	1	Q6ZWY3	40S ribosomal protein S27;40S ribosomal protein S27-like	Rps27l					
921	0.4805	0.2977	0.5604	0.4462	1	P26883	Peptidyl-prolyl cis-trans isomerase FKBP1A;Peptidyl-prolyl cis-trans isomerase	Fkbp1a	mmu:14225				
922	0.3179	0.0957	0.9433	0.4523	1	Q922J3	CAP-Gly domain-containing linker protein 1	Clip1	mmu:56430				
923	0.2452	0.8150	0.3002	0.4535	1	P62869	Transcription elongation factor B polypeptide 2	Tceb2	mmu:67673			1	
924	0.1706	0.0344	1.1660	0.4570	1	Q9D0W5	Peptidyl-prolyl cis-trans isomerase-like 1	Ppil1	mmu:68816				
925	0.4443	0.1048	0.8387	0.4626	1	Q8C754	Vacuolar protein sorting-associated protein 52 homolog	Vps52	mmu:224705				
926	0.0289	0.7575	0.6227	0.4697	1	Q9WV32	Actin-related protein 2/3 complex subunit 1B	Arpc1b	mmu:11867				
927	0.7613	0.1517	0.4975	0.4702	1	Q60598	Src substrate cortactin	Cttn	mmu:13043				
928	0.9025	0.4754	0.0574	0.4784	1	Q8BKN5	Gamma-tubulin complex component 5	Tubgcp5	mmu:233276				
929	0.4592	0.2306	0.7525	0.4808	1	P63330	Serine/threonine-protein phosphatase 2A catalytic subunit alpha isoform;Serine/threonine-protei	Ppp2ca;Pp	mmu:19052		1		1
930	0.0177	0.8194	0.6333	0.4901	1	Q9QZD9	Eukaryotic translation initiation factor 3 subunit I	Eif3i	mmu:54709				
931	1.0248	0.1639	0.2888	0.4925	1	Q9DCA2	28S ribosomal protein S11, mitochondrial	Mrps11					
932	0.2596	0.8321	0.4110	0.5009	1	Q9CXP8	Guanine nucleotide-binding protein G(I)/G(S)/G(O) subunit gamma-10	Gng10	mmu:14700				
933	0.2459	0.8986	0.3605	0.5017	1	Q8R146	Acylamino-acid-releasing enzyme	Apeh	mmu:235606				
934	0.2475	0.5506	0.7167	0.5049	1	Q6NXJ0	Protein WWC2	Wwc2	mmu:52357				
935	0.5432	0.1055	0.8663	0.5050	1	Q8K2Y9	Cerebral cavernous malformations protein 2 homolog	Ccm2	mmu:216527				
936	1.0319	0.0291	0.4629	0.5080	1	P35980	60S ribosomal protein L18	Rpl18	mmu:19899				
937	0.7798	0.0723	0.6818	0.5113	1	E2JF22	Piezo-type mechanosensitive ion channel component 1;Piezo-type mechanosensitive ion channel	Piezo1					
938	0.2927	0.7597	0.4822	0.5115	1	Q923G2	DNA-directed RNA polymerases I, II, and III subunit RPABC3	Polr2h	mmu:245841				
939	0.7457	0.0173	0.7831	0.5154	1	Q6NVE9	Protein phosphatase PTC7 homolog	Pptc7	mmu:320717		1		
940	0.7015	0.6553	0.1917	0.5162	1	Q8K124	Pleckstrin homology domain-containing family O member 2	Plekho2	mmu:102595				
941	1.0448	0.2530	0.2516	0.5165	1	Q4VBD2	Transmembrane anterior posterior transformation protein 1	Tapt1	mmu:231225				
942	0.2158	0.7360	0.6042	0.5187	1	Q6NZB1	Protein arginine N-methyltransferase 6	Prmt6	mmu:99890				
943	0.6692	0.2917	0.5966	0.5192	1	Q5SFM8	RNA-binding protein 27	Rbm27	mmu:225432				
944	0.6955	0.7479	0.1343	0.5259	1	A2A432	Cullin-4B	Cul4b	mmu:72584			1	
945	0.6464	0.3404	0.6060	0.5309	1	Q8BH43	Wiskott-Aldrich syndrome protein family member 2	Wasf2	mmu:242687				
946	0.5150	0.7657	0.3160	0.5322	1	Q9JHQ5	Leucine zipper transcription factor-like protein 1	Lztfl1	mmu:93730				
947	0.2346	0.1496	1.2148	0.5330	1	Q61216	Double-strand break repair protein MRE11A	Mre11a	mmu:17535				
948	0.2571	0.9152	0.4350	0.5358	1	Q5F2E7	Nuclear fragile X mental retardation-interacting protein 2	Nufip2	mmu:68564				
949	0.8396	0.1220	0.6545	0.5387	1	Q8R4C2	RUN and FYVE domain-containing protein 2	Rufy2	mmu:70432				
950	0.0966	0.8079	0.7213	0.5419	1	Q3UHX2	28 kDa heat- and acid-stable phosphoprotein	Pdap1	mmu:231887				
951	0.3133	0.7004	0.6296	0.5478	1	Q9JKB1	Ubiquitin carboxyl-terminal hydrolase isozyme L3;Ubiquitin carboxyl-terminal hydrolase isozyme L	Uchl3;Uchl	mmu:50933				
952	0.4553	0.6127	0.5770	0.5483	1	Q9QWY8	Arf-GAP with SH3 domain, ANK repeat and PH domain-containing protein 1	Asap1	mmu:13196				
953	0.5244	0.6650	0.4757	0.5550	1	Q8VCE4	Uncharacterized protein C9orf40 homolog	D030056L2	mmu:225995				
954	0.4182	0.8571	0.3919	0.5557	1	Q921W4	Quinone oxidoreductase-like protein 1	Cryzl1	mmu:66609				
955	0.0385	0.6628	0.9672	0.5562	1	P02088	Hemoglobin subunit beta-1;Hemoglobin subunit beta-2	Hbb-b1;Hb	mmu:15129				
956	0.5397	0.6895	0.4487	0.5593	1	Q3UFS4	G patch domain-containing protein 11	Gpatch11	mmu:53951				
957	1.0169	0.2010	0.4774	0.5651	1	P47962	60S ribosomal protein L5	Rpl5	mmu:100503670				
958	0.6581	0.4220	0.6160	0.5654	1	Q8VDZ4	Palmitoyltransferase ZDHHC5	Zdhhc5	mmu:228136				
959	0.2598	0.2307	1.2061	0.5655	1	Q91YI1	Autophagy-related protein 13	Atg13	mmu:51897				
960	0.3515	0.6630	0.6844	0.5663	1	B2RUR8	OTU domain-containing protein 7B	Otud7b	mmu:229603				
961	0.9111	0.1746	0.6142	0.5666	1	Q9Z0Y1	Dynactin subunit 3	Dctn3	mmu:53598				
962	0.2611	0.3846	1.0672	0.5710	1	Q9CQX2	Cytochrome b5 type B	Cyb5b	mmu:66427				
963	0.2518	0.5579	0.9038	0.5712	1	P97480	Eyes absent homolog;Eyes absent homolog 3	Eya3	mmu:14050		1		
964	0.2923	0.6469	0.7862	0.5751	1	P63158	High mobility group protein B1	Hmgb1	mmu:15289				1
965	0.8377	0.3179	0.5804	0.5787	1	Q3UMF0	Cordon-bleu protein-like 1	Cobll1	mmu:319876				
966	1.1999	0.1277	0.4208	0.5828	1	P47963	60S ribosomal protein L13	Rpl13	mmu:270106				
967	0.7925	0.4949	0.4666	0.5847	1	O55229	Choline/ethanolamine kinase	Chkb	mmu:12651				
968	0.0323	1.0494	0.6907	0.5908	1	Q60575	Kinesin-like protein KIF1B;Kinesin-like protein	Kif1b	mmu:16561				
969	0.3332	0.9055	0.5497	0.5961	1	Q9D6Z1	Nucleolar protein 56	Nop56	mmu:67134				
970	1.2895	0.0205	0.4906	0.6002	1	Q8C6B9	Active regulator of SIRT1	Rps19bp1	mmu:66538				
971	0.3691	0.7842	0.6576	0.6036	1	Q8VHH5	Arf-GAP with GTPase, ANK repeat and PH domain-containing protein 3	Agap3		1			
972	0.6325	0.9123	0.2744	0.6064	1	E9PYH6	Histone-lysine N-methyltransferase	Setd1a	mmu:233904				
973	0.3094	1.2803	0.2308	0.6068	1	P68040	Guanine nucleotide-binding protein subunit beta-2-like 1;Guanine nucleotide-binding protein sub	Gnb2l1	mmu:14694				
974	0.3778	1.2561	0.1881	0.6073	1	Q8CJF7	Protein ELYS	Ahctf1	mmu:226747				
975	0.1641	0.8272	0.8346	0.6086	1	Q8K4R9	Disks large-associated protein 5	Dlgap5	mmu:218977				
976	0.0796	0.5987	1.1829	0.6204	1	P35550	rRNA 2-O-methyltransferase fibrillarin	Fbl	mmu:14113				
977	0.5904	0.9013	0.3919	0.6279	1	Q91XV3	Brain acid soluble protein 1	Basp1	mmu:70350				
978	0.1327	0.7260	1.0499	0.6362	1	Q8VCI5	Peroxisomal biogenesis factor 19	Pex19	mmu:19298				
979	0.4516	0.7538	0.7059	0.6371	1	O55091	Protein IMPACT	Impact	mmu:16210	1			
980	1.6141	0.3257	0.0072	0.6490	1	Q9D8E6	60S ribosomal protein L4	Rpl4	mmu:67891				
981	0.8086	0.5937	0.5459	0.6494	1	Q8K2J0	1-phosphatidylinositol 4,5-bisphosphate phosphodiesterase delta-3;Phosphoinositide phospholipa	Plcd3	mmu:72469				
982	0.2372	0.8034	0.9326	0.6577	1	B2RY56	RNA-binding protein 25	Rbm25	mmu:67039				
983	1.0609	0.2298	0.6953	0.6620	1	Q00899	Transcriptional repressor protein YY1	Yy1	mmu:22632				
984	0.3394	1.4608	0.1934	0.6645	1	E9PVX6		Mki67	mmu:17345				
985	0.8145	0.4587	0.7402	0.6711	1	Q6ZWV3	60S ribosomal protein L10;60S ribosomal protein L10-like	Rpl10;Rpl1	mmu:110954				
986	1.2630	0.4597	0.3185	0.6804	1	P14115	60S ribosomal protein L27a	Rpl27a	mmu:26451				
987	0.9138	0.3966	0.7390	0.6831	1	Q60809	CCR4-NOT transcription complex subunit 7	Cnot7	mmu:18983				
988	1.3381	0.6520	0.0679	0.6860	1	P47964	60S ribosomal protein L36	Rpl36					
989	0.7214	0.1768	1.1710	0.6897	1	Q9QZ88	Vacuolar protein sorting-associated protein 29	Vps29	mmu:56433				
990	0.5176	0.5282	1.0350	0.6936	1	Q8BR90	UPF0600 protein C5orf51 homolog		mmu:106064				
991	0.6055	0.7065	0.7880	0.7000	1	Q9JLV1	BAG family molecular chaperone regulator 3	Bag3	mmu:29810				1
992	0.3458	1.3880	0.3666	0.7001	1	Q3UMU9	Hepatoma-derived growth factor-related protein 2	Hdgfrp2	mmu:15193				
993	0.4385	1.0775	0.6194	0.7118	1	P07607	Thymidylate synthase	Tyms	mmu:22171				
994	0.6791	1.1404	0.3197	0.7131	1	Q9WVR4	Fragile X mental retardation syndrome-related protein 2	Fxr2					
995	0.9589	0.9049	0.2761	0.7133	1	Q8BJW6	Eukaryotic translation initiation factor 2A;Eukaryotic translation initiation factor 2A, N-terminally p	Eif2a	mmu:229317				
996	0.9342	0.9160	0.2999	0.7167	1	Q3U3R4	Lipase maturation factor;Lipase maturation factor 1	Lmf1	mmu:76483				
997	0.3479	1.2051	0.6056	0.7195	1	Q921T2	Torsin-1A-interacting protein 1	Tor1aip1	mmu:208263				

998	0.0174	0.6637	1.5195	0.7335	1	Q8BHL8	Proteasome inhibitor PI31 subunit	Psmf1	mmu:228769				
999	1.3940	0.5750	0.2443	0.7378	1	Q9CQR2	40S ribosomal protein S21	Rps21	mmu:66481				
1000	1.4207	0.5686	0.2418	0.7437	1	P59470	DNA-directed RNA polymerase III subunit RPC2	Polr3b	mmu:70428				
1001	0.6652	0.7812	0.7945	0.7470	1	Q8BVU0	Leucine-rich repeat and calponin homology domain-containing protein 3	Lrch3	mmu:70144				
1002	0.7481	1.0189	0.4951	0.7540	1	P47930	Fos-related antigen 2	Fosl2	mmu:14284				
1003	0.6368	0.7992	0.8326	0.7562	1	P84099	Ribosomal protein L19;60S ribosomal protein L19	Rpl19	mmu:19921				
1004	0.4070	0.5327	1.3601	0.7666	1	P45481	CREB-binding protein	Crebbp					
1005	0.7663	1.0491	0.5510	0.7888	1	Q9D2C6	DNA-directed RNA polymerase III subunit RPC8	Polr3h	mmu:78929				
1006	0.9373	0.6311	0.8007	0.7897	1	Q6ZWV7	60S ribosomal protein L35	Rpl35;Gm1	mmu:66489				
1007	0.3650	1.0517	0.9537	0.7901	1	P62892	60S ribosomal protein L39	Rpl39	mmu:67248				
1008	0.0722	1.1474	1.1632	0.7943	1	Q9CWU4	UPF0690 protein C1orf52 homolog	2410004B1	mmu:66421				
1009	0.6676	0.8130	0.9379	0.8062	1	Q03173	Protein enabled homolog	Enah	mmu:13800				
1010	1.2769	1.0398	0.1119	0.8095	1	Q9CZM2	Ribosomal protein L15;60S ribosomal protein L15	Gm10020;	mmu:66480				
1011	0.4532	1.3336	0.6517	0.8128	1	Q9QZH3	Peptidyl-prolyl cis-trans isomerase E	Ppie	mmu:56031				
1012	0.8088	0.5749	1.0584	0.8140	1	Q80XK6	Autophagy-related protein 2 homolog B	Atg2b	mmu:76559				
1013	0.0867	0.5160	1.8561	0.8196	1	Q8BIF0	CD99 antigen-like protein 2	Cd99l2	mmu:171486				
1014	0.8409	0.6273	1.0187	0.8290	1	Q7TSJ2	Microtubule-associated protein 6	Map6	mmu:17760				
1015	1.1295	0.7685	0.6047	0.8342	1	P14206	40S ribosomal protein SA	Rpsa	mmu:16785				
1016	0.6856	0.2081	1.6192	0.8376	1	Q9WV96	Mitochondrial import inner membrane translocase subunit Tim10 B	Timm10b	mmu:14356				
1017	0.5667	0.6940	1.2539	0.8382	1	Q9Z103	Activity-dependent neuroprotector homeobox protein	Adnp	mmu:11538				
1018	1.5014	0.4221	0.6064	0.8433	1	Q7TMX5	Protein SHQ1 homolog	Shq1	mmu:72171				
1019	0.1943	1.0124	1.3286	0.8451	1	Q9WTJ4	Flt3-interacting zinc finger protein 1	Fiz1	mmu:23877				
1020	1.8579	0.0441	0.6347	0.8456	1	O09167	60S ribosomal protein L21	Rpl21					
1021	0.9201	0.1585	1.4659	0.8482	1	Q9EPS3	D-glucuronyl C5-epimerase	Glce	mmu:93683				
1022	1.2160	0.5807	0.7590	0.8519	1	P19253	60S ribosomal protein L13a	Rpl13a	mmu:22121				
1023	1.1322	0.9224	0.5339	0.8628	1	Q6PAM1	Alpha-taxilin	Txlna	mmu:109658				
1024	0.0138	0.8492	1.7838	0.8823	1	Q8BKX1	Brain-specific angiogenesis inhibitor 1-associated protein 2	Baiap2	mmu:108100				
1025	1.3349	0.7081	0.6844	0.9091	1	Q6P6L0	Filamin A-interacting protein 1-like	Filip1l					
1026	0.7093	0.8679	1.1551	0.9108	1	Q8C0E2	Vacuolar protein sorting-associated protein 26B	Vps26b	mmu:69091				
1027	1.6152	0.5964	0.6411	0.9509	1	Q9ER67		Maged2	mmu:80884				
1028	0.2890	1.5797	1.0818	0.9835	1	P63073	Eukaryotic translation initiation factor 4E	Eif4e	mmu:13684				
1029	0.1827	2.0287	0.7921	1.0012	1	O35071	Kinesin-like protein;Kinesin-like protein KIF1C	Kif1c	mmu:16562				
1030	1.0593	0.6685	1.3305	1.0194	1	P10649	Glutathione S-transferase Mu 1	Gstm1	mmu:14862				
1031	0.1897	0.4551	2.4747	1.0398	1	P18572	Basigin	Bsg	mmu:12215				
1032	0.4213	2.2238	0.6006	1.0819	1	Q9DBM1	G patch domain-containing protein 1	Gpatch1	mmu:67471				
1033	0.7709	0.8885	1.6043	1.0879	1	Q8K387	Ubiquitin carboxyl-terminal hydrolase 45	Usp45	mmu:77593				
1034	0.1478	2.0306	1.1227	1.1004	1	P49769	Presenilin-1;Presenilin-1 NTF subunit;Presenilin-1 CTF subunit;Presenilin-1 CTF12	Psen1	mmu:19164		1		1
1035	1.0482	0.7767	1.5376	1.1208	1	Q8CG19	Latent-transforming growth factor beta-binding protein 1	Ltbp1	mmu:268977				
1036	2.3992	0.7477	0.2776	1.1415	1	Q80VA0	N-acetylgalactosaminyltransferase 7	Galnt7	mmu:108150				
1037	1.8928	1.3151	0.2167	1.1415	1	Q9QYH6	Melanoma-associated antigen D1	Maged1	mmu:94275				
1038	2.1330	0.5576	0.7504	1.1470	1	P14148	60S ribosomal protein L7	Rpl7	mmu:19989				
1039	1.4714	0.7692	1.2238	1.1548	1	P62918	60S ribosomal protein L8	Rpl8	mmu:26961				
1040	2.6397	0.3061	0.6540	1.1999	1	P47911	60S ribosomal protein L6	Rpl6;Gm54	mmu:19988				
1041	1.8524	1.8193	0.0656	1.2458	1	Q80TE0	RNA polymerase II-associated protein 1	Rpap1	mmu:68925				
1042	0.5689	1.8859	1.3580	1.2709	1	Q0P688		Eif4e2					
1043	1.7986	0.2364	1.8360	1.2903	1	Q3UA16	Kinetochore protein Spc25	Spc25	mmu:66442				
1044	2.6431	0.5434	0.7504	1.3123	1	Q8K1L5	Protein phosphatase 1 regulatory subunit 11	Ppp1r11	mmu:76497				
1045	1.1493	1.7594	1.5275	1.4787	1	Q3UMT1	Protein phosphatase 1 regulatory subunit 12C	Ppp1r12c	mmu:232807				
1046	2.8558	1.3210	0.4081	1.5283	1	Q8R0J7	Vacuolar protein sorting-associated protein 37B	Vps37b	mmu:330192				
1047	0.0413	4.7907	0.4590	1.7637	1	Q8BFZ3	Beta-actin-like protein 2	Actbl2	mmu:238880				
1048	4.2026	0.8901	0.5344	1.8757	1	Q9EQI8	39S ribosomal protein L46, mitochondrial	Mrpl46	mmu:67308				
1049	0.9647	2.2964	3.6969	2.3193	1	Q9CQZ0	ORM1-like protein 2;ORM1-like protein 1	Gm28778;	mmu:66844				
1050	-0.0585	0.1422	-0.0420	-0.9552	0	Q99J95	Cyclin-dependent kinase 9	Cdk9	mmu:107951	1			
1051	0.3741	-0.6619	-0.8765	-0.8486	0	P52332	Tyrosine-protein kinase;Tyrosine-protein kinase JAK1	Jak1		1			
1052	0.1761	-1.3860	-0.9331	-0.7924	0	Q61846	Maternal embryonic leucine zipper kinase	Melk	mmu:17279	1	1		
1053	-0.7819	0.0883	0.1461	-0.7218	0	P05622	Platelet-derived growth factor receptor beta	Pdgfrb	mmu:18596	1			
1054	0.7085	-0.2126	-0.4891	-0.4688	0	Q8C0P0	Serine/threonine-protein kinase greatwall	Mastl	mmu:67121	1			
1055	0.2285	-0.1663	-0.3802	-0.4529	0	P49025	Citron Rho-interacting kinase	Cit		1			
1056	1.2415	-0.1049	-1.5316	-0.4384	0	A2ASS6	Titin	Ttn		1	1		
1057	0.0961	-1.1508	-0.5087	-0.3237	0	P28867	Protein kinase C;Protein kinase C delta type;Protein kinase C delta type regulatory subunit;Protein	Prkcd	mmu:18753	1	1		
1058	0.0222	-0.7135	-0.3066	-0.2823	0	Q3UU96	Non-specific serine/threonine protein kinase;Serine/threonine-protein kinase MRCK alpha	Cdc42bpa		1			
1059	-0.1800	0.0879	0.2046	-0.2161	0	Q8BWW9	Serine/threonine-protein kinase N2	Pkn2	mmu:109333	1			
1060	0.0752	-0.7628	0.5865	-0.2053	0	P05132	cAMP-dependent protein kinase catalytic subunit alpha	Prkaca	mmu:18747	1			
1061	-0.1373	-0.5175	0.1234	-0.1901	0	Q7TSE6	Serine/threonine-protein kinase 38-like	Stk38l	mmu:232533	1			
1062	-0.6821	0.7360	-0.4610	-0.1566	0	Q9D2Y4	Mixed lineage kinase domain-like protein	Mlkl	mmu:74568	1			
1063	0.4539	-0.4213	-0.1308	-0.1543	0	Q9WVS7	Dual specificity mitogen-activated protein kinase kinase 5	Map2k5	mmu:23938	1			
1064	0.2010	-1.0197	-0.0152	-0.1517	0	Q8K0D0	Cyclin-dependent kinase 17	Cdk17	mmu:237459	1			
1065	0.0432	-0.0632	-0.1880	-0.1407	0	Q03145	Ephrin type-A receptor 2	Epha2	mmu:13836	1			
1066	0.2481	-0.2699	0.4748	-0.1252	0	Q03963	Interferon-induced, double-stranded RNA-activated protein kinase	Eif2ak2	mmu:19106	1			
1067	0.3512	-0.1461	0.3275	-0.1024	0	P83741	Serine/threonine-protein kinase WNK1	Wnk1	mmu:232341	1			
1068	-0.0435	0.0875	0.6517	-0.0943	0	Q9WV60	Glycogen synthase kinase-3 beta	Gsk3b	mmu:56637	1			
1069	-0.5739	0.9664	1.1812	-0.0904	0	Q9JLM8	Serine/threonine-protein kinase DCLK1	Dclk1	mmu:13175	1			
1070	0.0345	-0.6555	-0.9121	-0.0834	0	O70293	G protein-coupled receptor kinase 6	Grk6	mmu:26385	1			
1071	-0.0745	0.7668	0.1478	-0.0699	0	Q8BN21	Serine/threonine-protein kinase VRK2	Vrk2	mmu:69922	1	1		
1072	-0.3082	0.5545	0.3207	-0.0699	0	Q8BKC8	Phosphatidylinositol 4-kinase beta	Pi4kb	mmu:107650	1			
1073	[illegible]	0.3180	-0.1581	-0.0639	0	P24788	Cyclin-dependent kinase 11B	Cdk11b	mmu:12537	1			
1074	-0.4352	0.3475	-0.3313	-0.0637	0	Q64261	Cyclin-dependent kinase 6	Cdk6	mmu:12571	1			
1075	-0.1699	-0.0583	0.1894	-0.0516	0	P31938	Dual specificity mitogen-activated protein kinase kinase 1	Map2k1	mmu:26395	1			
1076	-0.9190	-0.2569	1.3094	-0.0371	0	P31750	RAC-alpha serine/threonine-protein kinase	Akt1	mmu:11651	1	1		1
1077	[illegible]	-0.1901	-0.2021	-0.0299	0	P18654	Ribosomal protein S6 kinase alpha-3	Rps6ka3	mmu:110651	1			
1078	-0.1188	0.5666	-1.3956	-0.0225	0	Q8BPM2	Mitogen-activated protein kinase kinase kinase kinase;Mitogen-activated protein kinase kinase kin	Map4k5	mmu:399510	1			
1079	0.8918	-0.0479	-0.3320	-0.0162	0	Q8K1R7	Serine/threonine-protein kinase Nek9	Nek9	mmu:217718	1			
1080	0.2192	0.1172	-0.1553	-0.0105	0	Q69Z38	Pseudopodium-enriched atypical kinase 1	Peak1	mmu:244895	1			
1081	0.2967	-0.3057	-0.3329	[illegible]	0	Q60751	Tyrosine-protein kinase receptor;Insulin-like growth factor 1 receptor;Insulin-like growth factor 1 r	Igf1r	mmu:16001	1			1
1082	-0.1789	1.1785	-0.4249	[illegible]	0	Q922H2	[Pyruvate dehydrogenase (acetyl-transferring)] kinase isozyme 3, mitochondrial	Pdk3	mmu:236900	1			
1083	-0.0189	0.2854	0.9239	0.0285	0	Q2NL51	Glycogen synthase kinase-3 alpha	Gsk3a	mmu:606496	1			
1084	0.3784	-0.5458	0.0267	0.0302	0	Q91XU3	Phosphatidylinositol 5-phosphate 4-kinase type-2 gamma	Pip4k2c	mmu:117150	1			
1085	-0.3125	[illegible]	-0.3003	0.0310	0	Q9Z2B9	Ribosomal protein S6 kinase alpha-4	Rps6ka4	mmu:56613	1			
1086	-0.0477	0.0473	-0.3326	0.0313	0	P54761	Receptor protein-tyrosine kinase;Ephrin type-B receptor 4	Ephb4	mmu:13846	1			
1087	0.1006	-0.0681	0.1036	0.0429	0	Q0KL02	Triple functional domain protein	Trio	mmu:223435	1			
1088	-1.8934	0.3645	-0.1709	0.0446	0	Q9D0L4	Uncharacterized aarF domain-containing protein kinase 1	Adck1	mmu:72113	1			
1089	0.0632	-0.0695	0.3297	0.0475	0	Q61136	Serine/threonine-protein kinase PRP4 homolog	Prpf4b	mmu:19134	1			
1090	0.2645	0.2601	-0.0391	0.0641	0	Q6P9R2	Serine/threonine-protein kinase OSR1	Oxsr1	mmu:108737	1	1		
1091	0.4755	0.4402	-0.3553	0.0872	0	Q8CEE6	PAS domain-containing serine/threonine-protein kinase	Pask	mmu:269224	1			
1092	0.0403	-0.1538	0.2945	0.1130	0	P97377	Cyclin-dependent kinase 2	Cdk2	mmu:12566	1			
1093	0.4607	0.1072	-0.0450	0.1370	0	P70336	Rho-associated protein kinase 2;Rho-associated protein kinase	Rock2	mmu:19878	1			
1094	-0.4874	-0.2751	0.4335	0.1507	0	Q91YS8	Calcium/calmodulin-dependent protein kinase type 1;Calcium/calmodulin-dependent protein kina	Camk1;Car	mmu:52163	1			
1095	-0.0223	-0.2066	0.3216	0.1510	0	Q9JI10	Serine/threonine-protein kinase 3;Serine/threonine-protein kinase 3 36kDa subunit;Serine/threor	Stk3	mmu:56274	1			
1096	1.1337	-0.0933	-0.7439	0.1579	0	Q6ZQ29	Serine/threonine-protein kinase TAO2	Taok2	mmu:381921	1			
1097	-0.0634	-0.3579	0.7366	0.1671	0	Q63932	Dual specificity mitogen-activated protein kinase kinase 2	Map2k2	mmu:26396	1			
1098	-0.4364	-0.0812	0.5986	0.1759	0	Q9EQC5	N-terminal kinase-like protein	Scyl1	mmu:78891	1			
1099	-0.2559	0.2530	0.2898	0.1771	0	P47809	Dual specificity mitogen-activated protein kinase kinase 4	Map2k4	mmu:26398	1			
1100	0.6633	-0.0394	-0.2865	0.1792	0	Q9JJ78	Lymphokine-activated killer T-cell-originated protein kinase	Pbk	mmu:52033	1			
1101	-0.0842	-0.0786	0.3567	0.1884	0	Q99KY4	Cyclin-G-associated kinase	Gak	mmu:231580	1		1	
1102	0.2427	0.8467	-0.0217	0.1900	0	Q7TT50	Serine/threonine-protein kinase MRCK beta	Cdc42bpb	mmu:217866	1			
1103	0.3227	-0.2728	-0.4576	0.1932	0	O54781	SRSF protein kinase 2;SRSF protein kinase 2 N-terminal;SRSF protein kinase 2 C-terminal	Srpk2	mmu:20817	1			
1104	0.0317	-0.9466	0.2888	0.1933	0	P68181	cAMP-dependent protein kinase catalytic subunit beta	Prkacb	mmu:18749	1			
1105	0.0449	-0.0087	0.3770	0.2188	0	Q63844	Mitogen-activated protein kinase 3;Mitogen-activated protein kinase	Mapk3	mmu:26417	1	1		
1106	0.3895	0.3868	-0.0607	0.2281	0	Q9JI11	Serine/threonine-protein kinase 4;Serine/threonine-protein kinase 4 37kDa subunit;Serine/threor	Stk4	mmu:58231	1			
1107	1.4151	-0.1552	-1.0224	0.2416	0	Q61083	Mitogen-activated protein kinase kinase kinase 2	Map3k2		1			
1108	-0.1618	-0.5961	0.1058	0.2456	0	P49615	Cyclin-dependent-like kinase 5	Cdk5	mmu:12568	1			
1109	-0.2059	-0.1162	0.3306	0.2494	0	Q8BKX6	Serine/threonine-protein kinase SMG1	Smg1	mmu:233789	1			
1110	-0.0123	0.2462	0.3710	0.2509	0	P11440	Cyclin-dependent kinase 1	Cdk1	mmu:12534	1	1		
1111	0.5983	-0.1039	[illegible]	0.2524	0	Q6P4S6	Serine/threonine-protein kinase SIK3	Sik3	mmu:70661	1			
1112	0.8436	-0.5561	-0.7358	0.2541	0	Q923T9	Calcium/calmodulin-dependent protein kinase type II subunit gamma	Camk2g	mmu:12325	1			
1113	0.5732	0.8479	-0.3287	0.2639	0	Q91VJ4	Serine/threonine-protein kinase 38	Stk38	mmu:106504	1			
1114	0.1398	-0.1573	-0.4073	0.2928	0	P47810	Wee1-like protein kinase	Wee1	mmu:22390	1			
1115	-0.1099	[illegible]	-0.4826	0.3086	0	P70451	Tyrosine-protein kinase Fer	Fer	mmu:14158	1	1		
1116	0.5541	0.0217	-0.0688	0.3092	0	Q8VD65	Phosphoinositide 3-kinase regulatory subunit 4	Pik3r4	mmu:75669	1			
1117	-0.3552	0.0722	0.2485	0.3140	0	Q99J45	Nuclear receptor-binding protein	Nrbp1	mmu:192292	1			
1118	2.2354	-0.5759	-1.0597	0.3157	0	Q5F2E8	Serine/threonine-protein kinase TAO1	Taok1	mmu:216965	1			
1119	0.2952	-0.2891	-0.0552	0.3236	0	Q9CQS5	Serine/threonine-protein kinase RIO2	Riok2	mmu:67045	1			
1120	1.0119	0.5947	-0.7811	0.3476	0	Q8BYC6	Serine/threonine-protein kinase TAO3	Taok3	mmu:330177	1			
1121	-0.1559	0.3736	0.6475	0.4087	0	P04627	Serine/threonine-protein kinase A-Raf	Araf	mmu:11836	1			
1122	-0.4920	0.1995	0.4774	0.4416	0	Q9JLN9	Serine/threonine-protein kinase mTOR	Mtor	mmu:56717	1			
1123	-0.2502	1.9079	0.8486	0.4611	0	Q61036	Serine/threonine-protein kinase PAK 3	Pak3	mmu:18481	1			

1124	0.1793	0.1847	[illegible]	0.5102	0	Q8K1Y2	Protein kinase C;Serine/threonine-protein kinase;Serine/threonine-protein kinase D3	Prkd3	mmu:75292	1				
1125	1.2407	-0.1391	-0.2554	0.5400	0	E9Q3L2		Pi4ka	mmu:224020	1				
1126	1.3991	-0.0787	0.8391	0.5959	0	Q922Q2	Serine/threonine-protein kinase RIO1	Riok1	mmu:71340	1				
1127	0.8513	0.4672	-0.1325	0.6532	0	O08648	Mitogen-activated protein kinase kinase kinase 4	Map3k4	mmu:26407	1				
1128	0.5415	0.5991	-0.1159	0.8209	0	Q6PDN3	Myosin light chain kinase, smooth muscle;Myosin light chain kinase, smooth muscle, deglutamylat	Mylk		1				
1129	0.4844	-0.4149	1.7645	0.9634	0	O70161	Phosphatidylinositol 4-phosphate 5-kinase type-1 gamma	Pip5k1c	mmu:18717	1				
1130	3.7124	-0.4321	-0.3406	1.0237	0	P16056	Receptor protein-tyrosine kinase;Hepatocyte growth factor receptor	Met		1				
1131	0.6352	-1.2626	1.5123	1.1516	0	Q80V03	Uncharacterized aarF domain-containing protein kinase 5	Adck5	mmu:268822	1				
1132	-0.0535	-0.7744	1.6606	-1.5988	0	P54071	Isocitrate dehydrogenase [NADP], mitochondrial	Idh2	mmu:269951					
1133	-0.8571	0.3169	0.3192	-1.5475	0	P62274	40S ribosomal protein S29	Rps29;Gm1	mmu:20090					
1134	-1.9727	0.3229	-3.0641	-1.3646	0	E9Q6J5		Bod1l	mmu:665775					
1135	-0.9586	-0.9827	0.2858	-1.3639	0	Q9CZR8	Elongation factor Ts, mitochondrial	Tsfm	mmu:66399					
1136	-0.2978	-0.2400	1.1053	-1.3441	0	F8VPZ5		Ercc6	mmu:319955					
1137	-3.5308	0.1204	-1.6626	-1.3397	0	Q9JHS9	Spliceosome-associated protein CWC15 homolog	Cwc15	mmu:66070					
1138	-0.8158	0.2550	-1.7890	-1.3068	0	Q925I1	ATPase family AAA domain-containing protein 3	Atad3;Atad	mmu:108888					
1139	0.1288	0.4524	-1.4943	-1.2801	0	Q8K2F8	Protein LSM14 homolog A	Lsm14a	mmu:67070					
1140	-1.3880	0.1830	-0.6996	-1.2739	0	P24527	Leukotriene A-4 hydrolase	Lta4h	mmu:16993					
1141	-1.3649	0.0110	-0.5402	-1.2689	0	P18760	Cofilin-1	Cfl1	mmu:12631					1
1142	0.1199	-1.2689	-2.3423	-1.2625	0	P48428	Tubulin-specific chaperone A	Tbca	mmu:21371					
1143	-0.1102	0.0439	-1.2504	-1.2403	0	P18242	Cathepsin D	Ctsd	mmu:13033					
1144	0.2745	-0.0374	-0.3516	-1.2266	0	Q8BG81	Polymerase delta-interacting protein 3	Poldip3	mmu:73826					
1145	-4.0702	0.1378	0.1891	-1.2145	0	Q9R053	Sodium channel protein type 11 subunit alpha;Sodium channel protein;Sodium channel protein ty	Scn11a;Scn	mmu:24046					
1146	-0.5479	0.3348	[illegible]	-1.2018	0	Q8BWT1	3-ketoacyl-CoA thiolase, mitochondrial	Acaa2	mmu:52538					
1147	-4.7309	0.7459	-0.5698	-1.1974	0	P62855	40S ribosomal protein S26	Rps26	mmu:27370					
1148	0.3927	-0.1762	-0.6593	-1.1567	0	Q9CQU0	Thioredoxin domain-containing protein 12	Txndc12	mmu:66073					
1149	-1.4424	0.4008	0.3822	-1.0955	0	P24452	Macrophage-capping protein	Capg						
1150	-2.7413	0.2130	-0.0297	-1.0756	0	E9PUA5	Kinesin-like protein	Kifc5b	mmu:16580					
1151	1.0783	-0.0330	0.3726	-1.0378	0	P61255	60S ribosomal protein L26	Rpl26	mmu:19941					
1152	1.1665	0.2827	-0.5258	-1.0314	0	P62281	40S ribosomal protein S11	Rps11	mmu:27207					
1153	1.3620	-0.3155	-1.0739	-1.0079	0	P43274	Histone H1,4	Hist1h1e	mmu:50709					
1154	-0.0746	-0.0312	0.4151	-0.9735	0	Q810A3	Tetratricopeptide repeat protein 9C	Ttc9c	mmu:70387					
1155	-0.3904	0.0101	-1.4620	-0.9730	0	Q9QX47	Protein SON	Son	mmu:20658					1
1156	0.2722	-0.6148	0.0054	-0.9702	0	Q9R1Q6	Transmembrane protein 176B	Tmem176b	mmu:65963					
1157	-2.1656	-0.1988	0.3852	-0.9529	0	P54227	Stathmin	Stmn1	mmu:16765					
1158	0.2147	-0.3515	-0.7737	-0.9504	0	P84244	Histone H3;Histone H3,3;Histone H3,2;Histone H3,1;Histone H3,3C	H3f3a;Hist	mmu:15081					
1159	0.1075	-0.6925	-0.5341	-0.9367	0	Q9CQ65	S-methyl-5-thioadenosine phosphorylase	Mtap	mmu:66902					
1160	-2.8252	-0.6525	0.5136	-0.9357	0	O08663	Methionine aminopeptidase 2	Metap2	mmu:56307					
1161	1.2923	-0.8461	-1.2705	-0.9341	0	Q99N95	39S ribosomal protein L3, mitochondrial	Mrpl3	mmu:94062					
1162	0.4453	-0.3790	-1.9878	-0.9182	0	Q9CYA0	Cysteine-rich with EGF-like domain protein 2	Creld2	mmu:76737					
1163	0.2596	0.9524	-0.4317	-0.9168	0	Q91X20	Set1/Ash2 histone methyltransferase complex subunit ASH2	Ash2l	mmu:23808					
1164	-2.5682	0.2991	-1.5825	-0.9152	0	O88796	Ribonuclease P protein subunit p30	Rpp30	mmu:54364					
1165	-0.8992	0.2178	-2.6860	-0.9081	0	Q8CAQ8	MICOS complex subunit Mic60	Immt	mmu:76614					
1166	-1.7319	-0.6295	0.3186	-0.9070	0	Q8R0W0	Epiplakin	Eppk1	mmu:223650					
1167	0.7619	-0.5831	-0.7734	-0.9067	0	Q921N6	Probable ATP-dependent RNA helicase DDX27	Ddx27	mmu:228889					
1168	0.9832	-0.1645	-2.0264	-0.9019	0	Q6P9Z1	SWI/SNF-related matrix-associated actin-dependent regulator of chromatin subfamily D member 3	Smarcd3	mmu:66993					
1169	-1.7251	0.3801	-0.2823	-0.8796	0	Q8BNV1	tRNA (uracil-5-)-methyltransferase homolog A	Trmt2a	mmu:15547					
1170	-0.8705	0.0384	1.0453	-0.8778	0	P62843	40S ribosomal protein S15	Rps15	mmu:20054					
1171	-0.0670	0.1816	-1.1524	-0.8746	0	Q9CWG8	NADH dehydrogenase [ubiquinone] complex I, assembly factor 7	Ndufaf7	mmu:73694					
1172	-0.9180	0.3350	-1.8210	-0.8682	0	Q9Z280	Phospholipase D1	Pld1						
1173	0.0455	-0.3865	-1.5355	-0.8645	0	P34884	Macrophage migration inhibitory factor	Mif	mmu:17319					
1174	-1.0341	0.1647	0.5530	-0.8626	0	P62301	40S ribosomal protein S13	Rps13	mmu:68052					
1175	-0.2653	-1.0081	0.1016	-0.8397	0	Q9WVM1	Rac GTPase-activating protein 1	Racgap1	mmu:26934					
1176	-0.2428	0.7782	-1.3991	-0.8383	0	O35451	Cyclic AMP-dependent transcription factor ATF-6 beta;Processed cyclic AMP-dependent transcript	Atf6b						
1177	-2.0826	-0.5909	1.0514	-0.8316	0	P35235	Tyrosine-protein phosphatase non-receptor type 11	Ptpn11	mmu:19247			1		
1178	-0.5447	0.2379	-1.3083	-0.8121	0	Q6NS46	Protein RRP5 homolog	Pdcd11	mmu:18572					
1179	-0.0550	-1.1170	0.2104	-0.8110	0	Q0P678	Zinc finger CCCH domain-containing protein 18	Zc3h18	mmu:76014					
1180	-0.3705	0.4930	-0.6561	-0.8038	0	Q8BIP0	Aspartate--tRNA ligase, mitochondrial	Dars2	mmu:226539					
1181	-1.7330	-0.6879	0.0202	-0.8001	0	Q9DCT5	Stromal cell-derived factor 2	Sdf2	mmu:20316					
1182	-2.5145	0.2779	-1.3204	-0.7899	0	Q8BMC4	Nucleolar protein 9	Nop9	mmu:67842					
1183	-1.4116	-0.6868	0.3661	-0.7888	0	Q6PGC1	ATP-dependent RNA helicase Dhx29	Dhx29	mmu:218629					
1184	0.9982	-0.0167	-0.5547	-0.7867	0	Q99KI0	Aconitate hydratase, mitochondrial	Aco2	mmu:11429					
1185	-0.6799	0.1235	-1.2650	-0.7657	0	Q61334	B-cell receptor-associated protein 29	Bcap29	mmu:12033					
1186	0.1427	-1.4190	-0.1309	-0.7644	0	Q9Z2I8	Succinyl-CoA ligase [GDP-forming] subunit beta, mitochondrial	Suclg2	mmu:20917					
1187	-1.2897	0.2512	0.4357	-0.7640	0	P32921	Tryptophan--tRNA ligase, cytoplasmic;T1-TrpRS;T2-TrpRS	Wars	mmu:22375					
1188	-0.6521	-0.1808	0.0937	-0.7616	0	Q8R1G6	PDZ and LIM domain protein 2	Pdlim2	mmu:213019					
1189	0.1518	-0.3650	-0.3466	-0.7592	0	Q91W90	Thioredoxin domain-containing protein 5	Txndc5	mmu:105245					
1190	-2.6690	0.0307	-0.2387	-0.7547	0	P16254	Signal recognition particle 14 kDa protein;Signal recognition particle 14 kDa protein, N-terminally	Srp14	mmu:20813					
1191	-0.1485	0.4460	-1.3045	-0.7456	0	Q921Y2	U3 small nucleolar ribonucleoprotein protein IMP3	Imp3	mmu:102462					
1192	-1.2317	0.2825	-1.2267	-0.7415	0	Q3U308	Cytoplasmic tRNA 2-thiolation protein 2	Ctu2	mmu:66965					
1193	0.6510	-1.6001	0.0554	-0.7312	0	P83093	Stromal interaction molecule 2	Stim2	mmu:116873					
1194	-0.0755	0.2923	0.3314	-0.7310	0	P25444	40S ribosomal protein S2	Rps2;Gm18	mmu:16898					
1195	0.4984	0.0146	-1.4261	-0.7306	0	G3X9J0		Sipa1l3	mmu:74206					
1196	-0.3209	0.1000	-1.2835	-0.7241	0	P97478	5-demethoxyubiquinone hydroxylase, mitochondrial	Coq7	mmu:12850		1			
1197	-1.0403	0.2044	-0.8156	-0.7213	0	Q9D4H2	GRIP and coiled-coil domain-containing protein 1	Gcc1	mmu:74375					
1198	-0.5116	-0.7041	0.3797	-0.7202	0	P62500	TSC22 domain family protein 1	Tsc22d1	mmu:21807					
1199	-1.7091	0.7381	0.0672	-0.7171	0	Q9CSH3	Exosome complex exonuclease RRP44	Dis3	mmu:72662					
1200	-1.1716	-1.8680	0.4618	-0.7117	0	Q3UMC0	Spermatogenesis-associated protein 5	Spata5	mmu:57815					
1201	-0.9776	-0.0674	0.3182	-0.7106	0	P61087	Ubiquitin-conjugating enzyme E2 K	Ube2k	mmu:53323				1	
1202	1.4882	-1.6930	-0.8835	-0.7092	0	O09111	NADH dehydrogenase [ubiquinone] 1 beta subcomplex subunit 11, mitochondrial	Ndufb11	mmu:104130					
1203	0.0829	-1.0521	-0.0670	-0.6980	0	Q9JKK1	Syntaxin-6	Stx6	mmu:58244					
1204	-0.1971	-0.5035	0.1327	-0.6961	0	Q9Z1M8	Protein Red	Ik	mmu:24010					
1205	0.0887	-0.5866	-1.1468	-0.6860	0	P63276	40S ribosomal protein S17	Rps17	mmu:20068					
1206	-0.5221	-0.8046	0.1942	-0.6825	0	P97807	Fumarate hydratase, mitochondrial	Fh	mmu:14194					
1207	-0.7355	0.2755	0.5238	-0.6825	0	P60867	40S ribosomal protein S20	Rps20	mmu:67427					
1208	-0.7230	0.9585	0.3082	-0.6811	0	B2RWS6	Histone acetyltransferase p300	Ep300	mmu:328572					
1209	0.6006	-0.8137	-0.7473	-0.6801	0	Q9ER73	Elongator complex protein 4	Elp4	mmu:77766					
1210	-0.2246	0.6500	-0.3785	-0.6762	0	Q9R0X4	Acyl-coenzyme A thioesterase 9, mitochondrial;Acyl-coenzyme A thioesterase 10, mitochondrial	Acot9;Acot	mmu:56360					
1211	-0.6928	0.2821	-1.8267	-0.6734	0	Q80U70	Polycomb protein Suz12	Suz12	mmu:52615					
1212	-0.1424	0.1155	-0.3387	-0.6727	0	Q9Z2L6	Multiple inositol polyphosphate phosphatase 1	Minpp1	mmu:17330			1		
1213	0.4689	0.1605	-0.1235	-0.6653	0	Q80ZM5		H1fx	mmu:243529					
1214	-0.9935	0.3613	-1.0550	-0.6496	0	Q8BH04	Phosphoenolpyruvate carboxykinase [GTP], mitochondrial	Pck2	mmu:74551					
1215	-0.4694	0.2642	0.2653	-0.6488	0	Q9DBL7	Bifunctional coenzyme A synthase;Phosphopantetheine adenylyltransferase;Dephospho-CoA kinas	Coasy	mmu:71743					
1216	-0.6058	-0.2283	0.0573	-0.6480	0	P42125	Enoyl-CoA delta isomerase 1, mitochondrial	Eci1	mmu:13177					
1217	-0.8589	0.1857	-0.7587	-0.6468	0	O88532	Zinc finger RNA-binding protein	Zfr	mmu:22763					
1218	-1.6682	0.9767	0.1854	-0.6465	0	Q925H1	Zinc finger transcription factor Trps1	Trps1						
1219	-0.1400	-0.0270	0.3305	-0.6463	0	P62858	40S ribosomal protein S28	Rps28	mmu:54127					
1220	-0.4314	1.0726	0.3458	-0.6455	0	Q08288	Cell growth-regulating nucleolar protein	Lyar	mmu:17089					
1221	0.1261	-0.1353	-0.6602	-0.6409	0	Q8K2B3	Succinate dehydrogenase [ubiquinone] flavoprotein subunit, mitochondrial	Sdha	mmu:66945					
1222	-0.3886	0.8587	0.3612	-0.6404	0	Q8CGF7	Transcription elongation regulator 1	Tcerg1	mmu:56070					
1223	-0.7794	-0.4020	0.1456	-0.6364	0	P19324	Serpin H1	Serpinh1	mmu:12406					
1224	-1.6571	0.1458	-0.9606	-0.6334	0	Q9DCS9	NADH dehydrogenase [ubiquinone] 1 beta subcomplex subunit 10	Ndufb10	mmu:68342					
1225	-0.3124	0.3553	-1.2744	-0.6320	0	P50543	Protein S100-A11	S100a11	mmu:20195					
1226	-0.8963	0.0456	-0.1798	-0.6315	0	P62082	40S ribosomal protein S7	Rps7;Gm94	mmu:20115					
1227	-0.0915	1.2091	0.4858	-0.6263	0	Q9D0T1	NHP2-like protein 1;NHP2-like protein 1, N-terminally processed	Nhp2l1	mmu:20826					
1228	-0.3046	-1.4581	0.1079	-0.6216	0	Q8R1Q8	Cytoplasmic dynein 1 light intermediate chain 1	Dync1li1	mmu:235661					
1229	0.5126	-1.2765	-0.6119	-0.6216	0	Q3UDE2	Tubulin--tyrosine ligase-like protein 12	Ttll12	mmu:223723					
1230	-1.0790	0.2317	-2.3858	-0.6156	0	Q8K1E0	Syntaxin-5	Stx5a;Stx5	mmu:56389					
1231	0.1548	-0.1568	-0.2747	-0.6156	0	Q9QXX4	Calcium-binding mitochondrial carrier protein Aralar2	Slc25a13	mmu:50799					
1232	0.4217	-0.7623	-1.0790	-0.6123	0	Q8CI08	SLAIN motif-containing protein 2	Slain2	mmu:75991					
1233	-1.1101	0.4744	-3.0698	-0.6048	0	Q8VDF2	E3 ubiquitin-protein ligase UHRF1	Uhrf1	mmu:18140				1	
1234	-0.6310	0.2419	-0.9836	-0.6020	0	Q60848	Lymphocyte-specific helicase	Hells	mmu:15201					
1235	-3.2865	-0.8780	1.0475	-0.5953	0	Q9CPV7	Palmitoyltransferase ZDHHC6	Zdhhc6	mmu:66980					
1236	1.2554	-0.6675	1.1360	-0.5938	0	P39689	Cyclin-dependent kinase inhibitor 1	Cdkn1a	mmu:12575					1
1237	-0.7968	-0.0313	0.3005	-0.5886	0	P62754	40S ribosomal protein S6	Rps6	mmu:20104					
1238	0.2502	0.4448	-1.1993	-0.5866	0	P59235	Nucleoporin Nup43	Nup43	mmu:69912					
1239	0.5553	-0.5270	-1.9410	-0.5842	0	P33174	Chromosome-associated kinesin KIF4	Kif4	mmu:16571					
1240	0.1277	-0.2519	-1.1286	-0.5842	0	Q6ZQK5	Arf-GAP with coiled-coil, ANK repeat and PH domain-containing protein 2	Acap2	mmu:78618					
1241	0.1731	-0.4569	0.4448	-0.5829	0	Q8CIH9	Amidophosphoribosyltransferase	Ppat	mmu:231327					
1242	-0.2655	0.9432	-0.6201	-0.5825	0	Q9DAR7	m7GpppX diphosphatase	Dcps	mmu:69305					
1243	0.0658	0.2497	-0.2890	-0.5783	0	Q8R3G1	Nuclear inhibitor of protein phosphatase 1	Ppp1r8	mmu:100336					
1244	-0.0495	1.0559	-1.3674	-0.5779	0	Q4FZC9	Nesprin-3	Syne3	mmu:212073					
1245	-0.4179	-0.5740	0.2279	-0.5775	0	Q3THW5	Histone H2A,V;Histone H2A,Z;Histone H2A	H2afv;H2af	mmu:77605					
1246	-0.6577	0.1595	0.0983	-0.5769	0	Q921J2	GTP-binding protein Rheb	Rheb	mmu:19744					
1247	-0.2748	0.2177	-0.8287	-0.5745	0	Q8K2T8	RNA polymerase II-associated factor 1 homolog	Paf1	mmu:54624					
1248	1.4553	-1.1027	-1.2993	-0.5717	0	G3XA30		Nsmce4a	mmu:67872					
1249	-0.4650	0.2544	-0.5436	-0.5708	0	P50518	V-type proton ATPase subunit E 1	Atp6v1e1	mmu:11973					

1250	-1.0544	0.1201	-1.6275	-0.5683	0	Q9D6K8	FUN14 domain-containing protein 2	Fundc2	mmu:67391				
1251	-0.5717	-0.2107	0.0587	-0.5662	0	E9Q1P8	Interferon regulatory factor 2-binding protein 2	Irf2bp2	mmu:270110				
1252	-0.4450	0.2437	-0.7845	-0.5662	0	Q8BGC4	Zinc-binding alcohol dehydrogenase domain-containing protein 2	Zadh2	mmu:225791				
1253	-0.7367	0.2092	0.0431	-0.5620	0	O70493	Sorting nexin-12	Snx12	mmu:55988				
1254	-1.3168	-0.1685	0.0883	-0.5615	0	Q9CQ10	Charged multivesicular body protein 3	Chmp3	mmu:66700				
1255	0.6397	-0.3915	-1.3211	-0.5600	0	Q8VDS8	Syntaxin-18	Stx18	mmu:71116				
1256	0.2188	-0.3564	-0.4426	-0.5572	0	Q9CZ13	Cytochrome b-c1 complex subunit 1, mitochondrial	Uqcrc1	mmu:22273				
1257	-0.1260	-0.9432	0.5765	-0.5515	0	Q505F5	Leucine-rich repeat-containing protein 47	Lrrc47	mmu:72946				
1258	-0.8788	0.0373	-0.3178	-0.5502	0	Q5SUF2	Luc7-like protein 3	Luc7l3	mmu:67684				
1259	-1.2060	0.2326	-0.4156	-0.5440	0	P10107	Annexin A1	Anxa1	mmu:16952	1			1
1260	-0.2056	0.1813	-0.3825	-0.5415	0	Q99LP6	GrpE protein homolog 1, mitochondrial	Grpel1	mmu:17713				
1261	0.4661	-0.4862	-1.3119	-0.5409	0	Q3TKT4	Transcription activator BRG1	Smarca4	mmu:20586				
1262	-0.0690	0.0305	-0.9348	-0.5392	0	Q8K2C8	Glycerol-3-phosphate acyltransferase 4	Agpat6	mmu:102247				
1263	-0.7694	0.6533	-2.5858	-0.5378	0	Q9CR68	Cytochrome b-c1 complex subunit Rieske, mitochondrial;Cytochrome b-c1 complex subunit 11	Uqcrfs1	mmu:66694				
1264	-0.1316	-0.2763	0.2258	-0.5369	0	P23506	Protein-L-isoaspartate O-methyltransferase;Protein-L-isoaspartate(D-aspartate) O-methyltransfera	Pcmt1					
1265	0.1847	-0.3445	0.3264	-0.5367	0	Q99J09	Methylosome protein 50	Wdr77	mmu:70465				
1266	0.3765	0.6934	0.0059	-0.5349	0	P62900	60S ribosomal protein L31	Rpl31	mmu:114641				
1267	0.2271	0.3146	-1.8536	-0.5344	0	Q6ZWQ0	Nesprin-2	Syne2	mmu:319565				
1268	-0.5528	-1.5963	0.3468	-0.5338	0	Q6PHQ8	N-alpha-acetyltransferase 35, NatC auxiliary subunit	Naa35	mmu:78689				
1269	1.8189	-0.6625	-1.2366	-0.5338	0	Q9CXT8	Mitochondrial-processing peptidase subunit beta	Pmpcb	mmu:73078				
1270	-3.0212	0.0173	-0.8883	-0.5330	0	Q91ZU1	Ankyrin repeat and SOCS box protein 6	Asb6	mmu:72323			1	
1271	-1.5208	0.8787	-1.0117	-0.5290	0	Q8BPB0	MOB kinase activator 1B	Mob1b	mmu:68473				
1272	-0.6014	0.4635	0.0338	-0.5263	0	Q9WUR2	Enoyl-CoA delta isomerase 2, mitochondrial	Eci2;Eci3	mmu:23986				
1273	0.0990	-0.3594	0.8378	-0.5237	0	Q9EP97	Sentrin-specific protease 3	Senp3	mmu:80886				
1274	0.2260	-0.8620	-0.4060	-0.5234	0	Q9D1Q6	Endoplasmic reticulum resident protein 44	Erp44	mmu:76299				
1275	-0.2963	-0.5650	0.1654	-0.5221	0	Q7TSC1	Protein PRRC2A	Prrc2a	mmu:53761				
1276	-0.7588	-1.6680	0.4054	-0.5174	0	P18052	Receptor-type tyrosine-protein phosphatase;Receptor-type tyrosine-protein phosphatase alpha	Ptpra	mmu:19262		1		
1277	-0.6683	-0.1889	0.1184	-0.5170	0	Q60770	Syntaxin-binding protein 3	Stxbp3	mmu:20912				
1278	0.2559	-0.3382	-0.0804	-0.5130	0	Q8BIJ6	Isoleucine--tRNA ligase, mitochondrial	Iars2	mmu:381314				
1279	-0.5925	-0.2851	1.5889	-0.5126	0	P97384	Annexin A11;Annexin	Anxa11	mmu:11744				
1280	-0.4070	0.0207	-0.5791	-0.5119	0	Q9D9Z5	DET1- and DDB1-associated protein 1	Dda1	mmu:66498				
1281	-0.7052	0.3589	0.0562	-0.5096	0	Q9D0E1	Heterogeneous nuclear ribonucleoprotein M	Hnrnpm	mmu:76936				
1282	0.5079	0.7433	-0.0444	-0.5066	0	Q9CPR4	60S ribosomal protein L17	Rpl17					
1283	-0.4989	-0.0964	0.1168	-0.5053	0	Q9DCL9	Multifunctional protein ADE2;Phosphoribosylaminoimidazole-succinocarboxamide synthase;Phos	Paics	mmu:67054				
1284	-0.0697	-1.4160	1.5898	-0.4980	0	A2BH40	AT-rich interactive domain-containing protein 1A	Arid1a	mmu:93760				
1285	-0.2615	-0.5059	0.1380	-0.4966	0	Q9WTX6	Cullin-1	Cul1	mmu:26965			1	1
1286	1.3729	0.7350	-1.5762	-0.4933	0	Q9CY52	tRNA(His) guanylyltransferase;Probable tRNA(His) guanylyltransferase	Thg1l	mmu:66628				
1287	-1.0802	0.6863	0.0120	-0.4895	0	Q62136	Protein-tyrosine-phosphatase;Tyrosine-protein phosphatase non-receptor type 21	Ptpn21			1		
1288	0.9565	-0.0795	-0.8165	-0.4830	0	P11438	Lysosome-associated membrane glycoprotein 1	Lamp1	mmu:16783				
1289	0.4728	-1.4484	0.2476	-0.4785	0	Q60649	Caseinolytic peptidase B protein homolog	Clpb	mmu:20480				
1290	-1.0810	0.2338	-0.6249	-0.4785	0	Q99LC5	Electron transfer flavoprotein subunit alpha, mitochondrial	Etfa	mmu:110842				
1291	-0.3452	-0.3199	0.8867	-0.4784	0	Q6XLQ8		Calu					
1292	0.9284	-0.5268	0.8023	-0.4741	0	P43275	Histone H1,1	Hist1h1a	mmu:80838				
1293	-0.6682	-0.2360	0.5668	-0.4723	0	P46467	Vacuolar protein sorting-associated protein 4B	Vps4b	mmu:20479				
1294	-1.3422	0.3212	0.0589	-0.4696	0	Q9QYI3	DnaJ homolog subfamily C member 7	Dnajc7	mmu:56354				
1295	-1.0993	-0.0954	0.9343	-0.4680	0	Q922H1	Protein arginine N-methyltransferase 3	Prmt3	mmu:71974				
1296	0.1416	0.3985	-1.8721	-0.4677	0	Q99LC2	Cleavage stimulation factor subunit 1	Cstf1	mmu:67337				
1297	0.0331	0.7206	-0.3942	-0.4652	0	O35129	Prohibitin-2	Phb2	mmu:12034				
1298	0.2631	-0.7458	-0.7390	-0.4648	0	Q8R2U0	Nucleoporin SEH1	Seh1l	mmu:72124				
1299	-0.3755	-0.3474	0.3592	-0.4621	0	P62264	40S ribosomal protein S14	Rps14	mmu:20044				
1300	-0.6033	0.2830	1.6453	-0.4620	0	Q9CR16	Peptidyl-prolyl cis-trans isomerase D	Ppid	mmu:67738				
1301	0.2772	-0.4129	-0.6886	-0.4618	0	P97493	Thioredoxin, mitochondrial	Txn2	mmu:56551	1			
1302	0.0611	-0.2420	-1.0319	-0.4604	0	Q91ZU6	Dystonin	Dst	mmu:13518				
1303	-0.9014	-0.0333	0.1578	-0.4584	0	Q80VD1	Protein FAM98B	Fam98b	mmu:68215				
1304	1.7945	-0.6958	-0.9978	-0.4570	0	Q8K224	N-acetyltransferase 10	Nat10	mmu:98956				
1305	0.0688	-0.4878	0.0540	-0.4563	0	Q922U1	U4/U6 small nuclear ribonucleoprotein Prp3	Prpf3	mmu:70767				
1306	-0.2159	0.0536	-0.1333	-0.4552	0	Q8VDP4	Cell cycle and apoptosis regulator protein 2	Ccar2	mmu:219158				
1307	-0.5687	-0.1446	0.3704	-0.4536	0	P97351	40S ribosomal protein S3a	Rps3a	mmu:20091				1
1308	-0.3338	-2.0162	0.3492	-0.4492	0	O35516	Neurogenic locus notch homolog protein 2;Notch 2 extracellular truncation;Notch 2 intracellular c	Notch2	mmu:18129				1
1309	-0.0723	0.2873	0.4658	-0.4490	0	P61514	60S ribosomal protein L37a	Rpl37a	mmu:19981				
1310	0.0123	-0.5806	0.3196	-0.4450	0	P30416	Peptidyl-prolyl cis-trans isomerase FKBP4;Peptidyl-prolyl cis-trans isomerase FKBP4, N-terminally p	Fkbp4	mmu:14228				
1311	-0.6082	0.0629	0.0838	-0.4435	0	P62702	40S ribosomal protein S4, X isoform	Rps4x	mmu:20102				
1312	-0.4808	0.6361	-0.1547	-0.4415	0	Q9WVA4	Transgelin-2	Tagln2	mmu:21346				
1313	0.2703	-0.5831	-0.4532	-0.4391	0	Q8BK72	28S ribosomal protein S27, mitochondrial	Mrps27	mmu:218506				
1314	0.6740	0.0713	-0.4305	-0.4390	0	P63323	40S ribosomal protein S12	Rps12					
1315	-0.0798	-1.5705	1.1864	-0.4365	0	Q9ERG0	LIM domain and actin-binding protein 1	Lima1	mmu:65970				
1316	-0.9610	0.1016	0.0508	-0.4350	0	P48024	Eukaryotic translation initiation factor 1;Eukaryotic translation initiation factor 1b	Eif1;Eif1b	mmu:20918				
1317	0.3940	-1.0366	-0.0982	-0.4246	0	Q6ZQB6	Inositol hexakisphosphate and diphosphoinositol-pentakisphosphate kinase 2	Ppip5k2	mmu:227399		1		
1318	-0.4516	-0.4811	0.7313	-0.4243	0	P13020	Gelsolin	Gsn	mmu:227753				
1319	0.4976	-2.5070	-0.8426	-0.4242	0	Q9CZH3	Proteasome assembly chaperone 3	Gm26938;I	mmu:66506				
1320	-0.0259	-0.4602	0.1796	-0.4226	0	P23591	GDP-L-fucose synthase	Tsta3	mmu:22122				
1321	-0.3092	0.0173	-0.9499	-0.4212	0	Q8CEC0	Nuclear pore complex protein Nup88	Nup88	mmu:19069				
1322	-0.5890	-0.3745	0.5101	-0.4198	0	Q9CQA6	Coiled-coil-helix-coiled-coil-helix domain-containing protein 1	Chchd1	mmu:66121				
1323	-0.5854	0.0466	-0.3676	-0.4196	0	Q61029	Lamina-associated polypeptide 2, isoforms beta/delta/epsilon/gamma;Lamina-associated polypep	Tmpo	mmu:21917				
1324	1.0696	-0.3929	-2.0017	-0.4195	0	Q9DBD5	Proline-, glutamic acid- and leucine-rich protein 1	Pelp1	mmu:75273				
1325	-0.9281	-0.2586	0.1732	-0.4117	0	P54822	Adenylosuccinate lyase	Adsl	mmu:11564				
1326	-1.8476	1.1078	-0.3018	-0.4115	0	Q9ERR7	15 kDa selenoprotein	Sep15	mmu:93684				
1327	2.0239	-1.0505	-1.8163	-0.4113	0	Q9R0Q3	Transmembrane emp24 domain-containing protein 2	Tmed2	mmu:56334				
1328	0.2529	0.2559	-1.0810	-0.4089	0	O35387	HCLS1-associated protein X-1	Hax1	mmu:23897				
1329	-0.4812	0.2678	-0.9118	-0.4085	0	Q9DCW4	Electron transfer flavoprotein subunit beta	Etfb	mmu:110826				
1330	0.1635	-0.6938	-0.2158	-0.4036	0	Q3UHB1	5-nucleotidase domain-containing protein 3	Nt5dc3	mmu:103466				
1331	0.0916	0.0458	-0.1474	-0.4025	0	Q60974	Nuclear receptor corepressor 1	Ncor1					
1332	-1.2627	0.0813	-0.2413	-0.4019	0	P35283	Ras-related protein Rab-12	Rab12	mmu:19328				
1333	0.1023	-0.2916	-0.3459	-0.4006	0	Q60854	Serpin B6	Serpinb6;S	mmu:20719				
1334	0.1530	-0.8422	0.5229	-0.4003	0	P61759	Prefoldin subunit 3	Vbp1	mmu:22327				
1335	0.1499	-0.8201	-0.2722	-0.3975	0	Q9QWV9	Cyclin-T1	Ccnt1	mmu:12455				
1336	-0.5292	-0.2349	0.6107	-0.3947	0	Q8R4E9	DNA replication factor Cdt1	Cdt1	mmu:67177				
1337	-2.1743	-0.5106	0.4645	-0.3923	0	Q8R3P6	von Willebrand factor A domain-containing protein 9	Vwa9	mmu:69882				
1338	-0.0412	0.1107	-0.0539	-0.3920	0	P43346	Deoxycytidine kinase	Dck	mmu:13178				
1339	-0.1971	1.2542	-1.0596	-0.3899	0	Q91ZW3	SWI/SNF-related matrix-associated actin-dependent regulator of chromatin subfamily A member	Smarca5	mmu:93762				
1340	0.0569	-0.2945	-0.4686	-0.3897	0	Q9QXK2	E3 ubiquitin-protein ligase RAD18	Rad18	mmu:58186			1	
1341	-0.5177	0.4648	0.4872	-0.3891	0	Q9CQ49	Nuclear cap-binding protein subunit 2	Ncbp2	mmu:68092				
1342	0.1484	0.0610	-0.3969	-0.3885	0	P97494	Glutamate--cysteine ligase catalytic subunit	Gclc	mmu:14629	1			
1343	0.1607	0.6042	-0.0828	-0.3884	0	P97789	5-3 exoribonuclease 1	Xrn1					
1344	-0.8876	0.1432	-0.0386	-0.3869	0	Q9CQ48	NudC domain-containing protein 2	Nudcd2	mmu:52653				
1345	0.7192	-1.5497	0.2966	-0.3867	0	P70303	CTP synthase 2	Ctps2	mmu:55936				
1346	0.1549	-0.3841	0.5618	-0.3861	0	Q9JKX6	ADP-sugar pyrophosphatase	Nudt5	mmu:53893				
1347	-2.7877	1.3720	0.0194	-0.3857	0	P39749	Flap endonuclease 1	Fen1					
1348	-0.5938	-0.6574	0.1683	-0.3828	0	Q9CQW1	Synaptobrevin homolog YKT6	Ykt6	mmu:56418				
1349	0.2237	0.7284	-1.0887	-0.3821	0	Q91ZJ5	UTP--glucose-1-phosphate uridylyltransferase	Ugp2	mmu:216558				
1350	-0.4260	0.2473	-0.1293	-0.3811	0	P62827	GTP-binding nuclear protein Ran	Ran;17000	mmu:19384				
1351	-0.7495	-0.1029	0.1677	-0.3809	0	Q8R574	Phosphoribosyl pyrophosphate synthase-associated protein 2	Prpsap2	mmu:212627				
1352	-0.9132	-0.7872	0.1006	-0.3805	0	Q8VE18	Protein SMG8	Smg8	mmu:74133				
1353	-0.7608	-0.6813	0.5185	-0.3799	0	Q9JLB2	MAGUK p55 subfamily member 5	Mpp5	mmu:56217				
1354	-0.1985	0.0914	0.3125	-0.3781	0	O08915	AH receptor-interacting protein	Aip	mmu:11632				
1355	-0.6756	0.3704	-1.0586	-0.3770	0	Q9D0R2	Threonine--tRNA ligase, cytoplasmic	Tars	mmu:110960				
1356	-1.0343	0.1869	-0.0917	-0.3767	0	P50396	Rab GDP dissociation inhibitor alpha	Gdi1	mmu:14567				
1357	0.8927	0.5484	-0.2219	-0.3766	0	Q8K114	Integrator complex subunit 9	Ints9	mmu:210925				
1358	-0.3446	0.1166	-0.6831	-0.3757	0	F2Z4A3		Fat1	mmu:14107				
1359	-0.0927	0.1195	0.0498	-0.3757	0	Q8BJS4	SUN domain-containing protein 2	Sun2	mmu:223697				
1360	0.9326	-1.1448	-0.5650	-0.3756	0	Q9CR70	EKC/KEOPS complex subunit Lage3	Lage3	mmu:66192				
1361	0.5536	-0.3804	-1.0063	-0.3745	0	Q6ZPF4	Formin-like protein 3	Fmnl3	mmu:22379				
1362	0.4719	-0.6056	-0.0698	-0.3741	0	Q3THG9	Alanyl-tRNA editing protein Aarsd1	Gm27029;,	mmu:69684				
1363	-2.1722	1.2026	0.2610	-0.3739	0	P97429	Annexin A4;Annexin	Anxa4	mmu:11746				1
1364	-0.6824	-0.6209	0.2664	-0.3733	0	A2AJ72		Fubp3					
1365	0.0323	0.0731	-0.3039	-0.3720	0	Q7TNC4	Putative RNA-binding protein Luc7-like 2	Luc7l2	mmu:192196				
1366	-0.1240	0.0969	0.4144	-0.3697	0	Q6ZWN5	40S ribosomal protein S9	Rps9	mmu:76846				
1367	-0.1066	0.2342	-0.0699	-0.3676	0	Q99LM2	CDK5 regulatory subunit-associated protein 3	Cdk5rap3	mmu:80280				
1368	-0.8529	0.3855	0.1241	-0.3662	0	P62242	40S ribosomal protein S8	Rps8	mmu:20116				
1369	-0.7127	0.1472	-0.2320	-0.3641	0	Q9D0B0	Serine/arginine-rich splicing factor 9	Srsf9	mmu:108014				
1370	-0.4402	0.0642	-1.3791	-0.3639	0	Q9DBG7	Signal recognition particle receptor subunit alpha	Srpr	mmu:67398				
1371	-0.7302	-0.0718	0.1858	-0.3635	0	P35700	Peroxiredoxin-1	Prdx1	mmu:18477	1			
1372	0.1017	-0.8074	0.5673	-0.3629	0	P62627	Dynein light chain roadblock-type 1	Dynlrb1	mmu:67068				
1373	0.0737	-0.9352	1.9190	-0.3611	0	Q9CS74	Protein SGT1 homolog	Ecd	mmu:70601				
1374	-1.0886	0.9404	0.4776	-0.3606	0	P17742	Peptidyl-prolyl cis-trans isomerase A;Peptidyl-prolyl cis-trans isomerase A, N-terminally processed	Ppia	mmu:268373				
1375	-0.6491	0.1653	-1.6994	-0.3594	0	A6H6A9	Rab GTPase-activating protein 1-like	Rabgap1l	mmu:29809				

1376	-0.5021	0.2531	0.2236	-0.3559	0	Q9CPS7	RNA-binding protein PNO1	Pno1	mmu:66249		
1377	0.1352	0.9856	-0.4611	-0.3557	0	Q9JL35	High mobility group nucleosome-binding domain-containing protein 5	Hmgn5	mmu:50887		
1378	-0.0087	-0.3588	0.3079	-0.3544	0	Q9DC71	28S ribosomal protein S15, mitochondrial	Mrps15	mmu:66407		
1379	0.2168	0.0201	-0.3111	-0.3527	0	P62267	40S ribosomal protein S23	Rps23	mmu:66475		
1380	-0.5073	1.0274	-1.9958	-0.3514	0	Q6NXI6	Regulation of nuclear pre-mRNA domain-containing protein 2	Rprd2	mmu:75137		
1381	0.0686	-0.6090	-0.7942	-0.3500	0	Q9DB77	Cytochrome b-c1 complex subunit 2, mitochondrial	Uqcrc2	mmu:67003		
1382	0.0922	-1.2538	-0.3863	-0.3495	0	Q8HWA3		Ulbp1	mmu:77777		
1383	-0.5487	-0.5981	0.1669	-0.3477	0	Q8K297	Procollagen galactosyltransferase 1	Colgalt1	mmu:234407		
1384	0.0512	-0.7771	-0.8032	-0.3451	0	Q9CZU4	GTPase Era, mitochondrial	Eral1	mmu:57837		
1385	-0.0527	-0.6204	0.5447	-0.3428	0	Q9DCC4	Pyrroline-5-carboxylate reductase 3	Pycrl	mmu:66194		
1386	-0.0027	0.1069	0.0245	-0.3426	0	P26638	Serine--tRNA ligase, cytoplasmic	Sars	mmu:20226		
1387	-1.4183	1.0409	-0.5772	-0.3408	0	Q8R151	NFX1-type zinc finger-containing protein 1	Znfx1	mmu:98999		
1388	-0.4213	0.2487	-0.0859	-0.3408	0	P27773	Protein disulfide-isomerase A3	Pdia3	mmu:14827		
1389	-0.2456	0.7108	-0.4858	-0.3407	0	Q9QYJ3	DnaJ homolog subfamily B member 1	Dnajb1	mmu:81489		
1390	-0.7248	-0.8880	0.8883	-0.3403	0	Q9DBJ1	Phosphoglycerate mutase 1	Pgam1	mmu:18648	1	
1391	0.0985	-0.3091	-0.2827	-0.3402	0	Q3V1V3	ESF1 homolog	Esf1	mmu:66580		
1392	-0.5552	0.2699	0.3172	-0.3399	0	Q3U0V1	Far upstream element-binding protein 2	Khsrp	mmu:16549		
1393	-0.9487	0.1652	0.3744	-0.3395	0	Q921I6	SH3 domain-binding protein 4	Sh3bp4	mmu:98402		
1394	-1.1732	0.0735	-0.2829	-0.3387	0	P20934	Protein EVI2A	Evi2a	mmu:14017		
1395	-1.7536	-0.2079	0.4931	-0.3374	0	Q07076	Annexin A7	Anxa7	mmu:11750		
1396	0.0516	-0.2860	-0.4478	-0.3364	0	Q62203	Splicing factor 3A subunit 2	Sf3a2			
1397	-3.4912	0.0521	-0.6054	-0.3359	0	Q61301	Catenin alpha-2	Ctnna2	mmu:12386		
1398	0.1936	0.1589	-1.0382	-0.3355	0	E9PWG6		Ncapg	mmu:54392		
1399	0.6639	-0.1161	-0.4955	-0.3341	0	P63280	SUMO-conjugating enzyme UBC9	Ube2i	mmu:22196		
1400	-1.7242	1.3717	0.1732	-0.3312	0	Q91YE7	RNA-binding protein 5	Rbm5	mmu:83486		
1401	[illegible]	0.1393	-0.4662	-0.3278	0	Q9CW03	Structural maintenance of chromosomes protein 3	Smc3	mmu:13006		
1402	-0.4739	-0.2436	0.0971	-0.3277	0	P58281	Dynamin-like 120 kDa protein, mitochondrial;Dynamin-like 120 kDa protein, form S1	Opa1	mmu:74143		1
1403	-0.5370	-0.6565	0.2649	-0.3274	0	P39098	Mannosyl-oligosaccharide 1,2-alpha-mannosidase IB	Man1a2	mmu:17156		
1404	-0.0023	0.1776	0.5323	-0.3271	0	Q8R180	ERO1-like protein alpha	Ero1l	mmu:50527		
1405	-1.4207	0.0856	0.6434	-0.3259	0	O70492	Sorting nexin-3	Snx3			
1406	-0.2458	-0.0318	0.3525	-0.3240	0	O35691	Pinin	Pnn	mmu:18949		
1407	0.0096	-0.2662	-0.1529	-0.3225	0	Q8CGY8	UDP-N-acetylglucosamine--peptide N-acetylglucosaminyltransferase 110 kDa subunit	Ogt	mmu:108155		
1408	0.3285	-0.7881	-0.5820	-0.3219	0	Q9CY16	28S ribosomal protein S28, mitochondrial	Mrps28	mmu:66230		
1409	-0.4956	-1.6722	0.0668	-0.3219	0	Q9QYB5	Gamma-adducin	Add3	mmu:27360		
1410	-0.0414	-0.1264	0.0683	-0.3210	0	Q6ZWU9	40S ribosomal protein S27	Rps27	mmu:57294		
1411	-0.0069	-0.1584	1.1186	-0.3202	0	Q6PGB6	N-alpha-acetyltransferase 50	Naa50	mmu:72117		
1412	-1.0943	0.0431	0.4124	-0.3173	0	Q61598	Rab GDP dissociation inhibitor beta	Gdi2	mmu:14569		
1413	0.0853	-0.3354	0.2631	-0.3167	0	Q91VW5	Golgin subfamily A member 4	Golga4	mmu:54214		
1414	-0.1296	0.7535	-3.0086	-0.3162	0	Q80YV3	Transformation/transcription domain-associated protein	Trrap			
1415	-0.6158	-0.1784	0.4189	-0.3154	0	Q9Z1Y4	Thyroid receptor-interacting protein 6	Trip6	mmu:22051		
1416	-0.3780	0.5995	-0.0938	-0.3131	0	Q9D4H1	Exocyst complex component 2	Exoc2	mmu:66482		
1417	0.0057	0.0090	-1.0759	-0.3129	0	P14733	Lamin-B1	Lmnb1	mmu:16906		
1418	-0.2386	0.0376	-1.7498	-0.3122	0	Q64012	RNA-binding protein Raly	Raly	mmu:19383		
1419	0.6735	-1.2690	1.1898	-0.3120	0	P47941	Crk-like protein	Crkl	mmu:12929		
1420	0.0640	-0.2753	-0.4168	-0.3093	0	Q8R361	Rab11 family-interacting protein 5	Rab11fip5	mmu:52055		
1421	0.1029	-0.9010	-0.8281	-0.3083	0	Q91Z31	Polypyrimidine tract-binding protein 2	Ptbp2	mmu:56195		
1422	-0.0469	-0.1601	0.2035	-0.3077	0	E9Q912		Rap1gds1	mmu:229877		
1423	0.5375	0.1492	-1.7786	-0.3056	0	Q8C0C7	Phenylalanine--tRNA ligase alpha subunit	Farsa	mmu:66590		
1424	-0.1949	-0.5466	0.1380	-0.3055	0	P07091	Protein S100-A4	S100a4	mmu:20198		
1425	-0.2176	0.2121	-0.2464	-0.3051	0	Q8BP92	Reticulocalbin-2	Rcn2	mmu:26611		
1426	-0.2699	0.5639	-0.3251	-0.3051	0	P60670	Nuclear protein localization protein 4 homolog	Nploc4	mmu:217365		
1427	0.1941	-0.1817	0.1942	-0.3040	0	Q99M31	Heat shock 70 kDa protein 14	Hspa14	mmu:50497		
1428	-0.3845	0.1533	0.5136	-0.3038	0	Q62348	Translin	Tsn	mmu:22099		
1429	-0.3195	-0.5839	0.2168	-0.3023	0	Q99LF4	tRNA-splicing ligase RtcB homolog	Rtcb	mmu:28088		
1430	0.3701	-0.0236	-1.0925	-0.3000	0	Q9EPU4	Cleavage and polyadenylation specificity factor subunit 1	Cpsf1	mmu:94230		
1431	-0.2899	-0.5486	1.0808	-0.2999	0	Q9WUA2	Phenylalanine--tRNA ligase beta subunit	Farsb	mmu:23874		
1432	-0.2137	-0.0122	0.1321	-0.2978	0	Q3TKY6	Peptidyl-prolyl cis-trans isomerase CWC27 homolog	Cwc27	mmu:67285		
1433	0.2723	-0.6832	-1.1492	-0.2977	0	O08715	A-kinase anchor protein 1, mitochondrial	Akap1	mmu:11640		
1434	0.0965	0.3591	-0.3198	-0.2967	0	Q99JY9	Actin-related protein 3	Actr3	mmu:74117		
1435	-0.2040	0.5365	-1.0839	-0.2941	0	Q9CRC8	Leucine-rich repeat-containing protein 40	Lrrc40	mmu:67144		
1436	0.0422	-0.3840	-0.2846	-0.2927	0	Q8K009	Mitochondrial 10-formyltetrahydrofolate dehydrogenase	Aldh1l2	mmu:216188		
1437	-0.4650	-0.4293	0.0606	-0.2921	0	Q8BH60	Golgi-associated PDZ and coiled-coil motif-containing protein	Gopc	mmu:94221		
1438	-0.2662	0.2638	0.5907	-0.2917	0	P14131	40S ribosomal protein S16	Rps16	mmu:20055		
1439	-0.1904	-0.7708	0.8574	-0.2916	0	Q5SSZ5	Tensin-3	Tns3	mmu:319939	1	
1440	-1.2085	0.0473	0.1896	-0.2908	0	P46664	Adenylosuccinate synthetase isozyme 2	Adss	mmu:11566		
1441	-0.3213	-0.1336	0.0231	-0.2869	0	O08583	THO complex subunit 4;Aly/REF export factor 2	Alyref;Alyr	mmu:21681		
1442	-0.6692	0.0324	-0.0079	-0.2862	0	Q8K1A6	Coiled-coil and C2 domain-containing protein 1A	Cc2d1a	mmu:212139		
1443	-0.5944	0.1761	-0.0516	-0.2854	0	Q8BMJ2	Leucine--tRNA ligase, cytoplasmic	Lars	mmu:107045		
1444	0.1742	-0.4038	0.0777	-0.2829	0	Q7TNE3	Sperm-associated antigen 7	Spag7	mmu:216873		
1445	-1.6125	0.4239	-0.0297	-0.2825	0	Q8C570	mRNA export factor	Rae1	mmu:66679		
1446	-0.2671	-0.4454	0.0217	-0.2823	0	Q99J62	Replication factor C subunit 4	Rfc4	mmu:106344		
1447	-0.0140	0.6041	0.4443	-0.2821	0	Q8R3Y8	Interferon regulatory factor 2-binding protein 1	Irf2bp1	mmu:272359		
1448	-0.1389	-0.7852	0.3356	-0.2806	0	Q8BW10	RNA-binding protein NOB1	Nob1	mmu:67619		
1449	0.4488	-0.3054	-0.9325	-0.2793	0	Q8VCD5	Mediator of RNA polymerase II transcription subunit 17	Med17	mmu:234959		
1450	-0.1999	0.2114	-0.7337	-0.2792	0	Q3V3R1	Monofunctional C1-tetrahydrofolate synthase, mitochondrial	Mthfd1l	mmu:270685		
1451	0.1623	-1.1853	-0.1060	-0.2788	0	Q61624	Zinc finger protein 148	Znf148	mmu:22661		
1452	-0.7604	-0.0668	0.6585	-0.2788	0	Q8R550	SH3 domain-containing kinase-binding protein 1	Sh3kbp1	mmu:58194		
1453	0.0476	-0.1247	0.1531	-0.2786	0	Q9CPV5	Polyamine-modulated factor 1	Pmf1	mmu:67037		
1454	-0.2457	0.5447	-1.0415	-0.2784	0	Q9JIX0	Transcription and mRNA export factor ENY2	Eny2	mmu:223527		
1455	0.0889	-0.4192	-0.2753	-0.2779	0	Q91VJ5	Polyglutamine-binding protein 1	Pqbp1	mmu:54633		
1456	0.2143	0.9460	-0.1444	-0.2774	0	Q8BK67	Protein RCC2	Rcc2	mmu:108911		
1457	-0.1836	0.3566	-0.4017	-0.2773	0	Q8JZM0	Dimethyladenosine transferase 1, mitochondrial	Tfb1m	mmu:224481		
1458	1.3182	-0.9528	-1.8262	-0.2766	0	Q923K4	tRNA modification GTPase GTPBP3, mitochondrial	Gtpbp3	mmu:70359		
1459	[illegible]	-0.0770	-0.7626	-0.2765	0	Q8CFX3		Pcdh1	mmu:75599		
1460	-1.2144	0.6907	-1.2485	-0.2745	0	Q922L6	Negative elongation factor D	Nelfcd	mmu:57314		
1461	0.6621	0.0530	-2.5424	-0.2733	0	Q920L1	Fatty acid desaturase 1	Fads1	mmu:76267		
1462	0.3570	-0.2067	-0.0430	-0.2724	0	P10852	4F2 cell-surface antigen heavy chain	Slc3a2	mmu:17254		
1463	-0.6752	0.1827	-0.0864	-0.2717	0	Q9WVK4	EH domain-containing protein 1	Ehd1	mmu:13660		
1464	-2.4733	0.4752	-0.1498	-0.2717	0	P53810	Phosphatidylinositol transfer protein alpha isoform	Pitpna	mmu:18738		
1465	-0.1637	0.0990	-0.2128	-0.2707	0	Q9JIH2	Nuclear pore complex protein Nup50	Nup50	mmu:18141		
1466	0.2349	-0.3003	-0.1697	-0.2705	0	E9PZP8		Herc1	mmu:235439		
1467	-0.8215	-0.3439	0.3261	-0.2697	0	P16858	Glyceraldehyde-3-phosphate dehydrogenase	Gm3839;G	mmu:14433		
1468	0.7985	2.0588	-1.7134	-0.2680	0	A2AR02	Peptidyl-prolyl cis-trans isomerase G;Peptidyl-prolyl cis-trans isomerase	Ppig	mmu:228005		
1469	-0.4576	0.7752	-0.3578	-0.2678	0	Q8CD10	Calcium uptake protein 2, mitochondrial	Micu2	mmu:68514		
1470	-0.4007	0.3351	-0.3907	-0.2673	0	Q9JI13	Something about silencing protein 10	Utp3	mmu:65961		
1471	-0.0866	0.0170	0.1286	-0.2671	0	Q8BGQ7	Alanine--tRNA ligase, cytoplasmic	Aars	mmu:234734		
1472	-0.8806	0.5579	-0.5187	-0.2669	0	Q921S7	39S ribosomal protein L37, mitochondrial	Mrpl37	mmu:56280		
1473	0.3271	0.3648	-0.0566	-0.2668	0	P70290	55 kDa erythrocyte membrane protein	Mpp1	mmu:17524		
1474	-0.5200	0.3615	-0.5666	-0.2663	0	Q64213	Splicing factor 1	Sf1			
1475	0.2815	-1.1680	-0.3970	-0.2639	0	P29758	Ornithine aminotransferase, mitochondrial	Oat	mmu:18242		
1476	-0.1667	0.6267	-2.4018	-0.2636	0	Q4VA53	Sister chromatid cohesion protein PDS5 homolog B	Pds5b	mmu:100710		
1477	0.1727	-0.2427	0.0481	-0.2629	0	P61222	ATP-binding cassette sub-family E member 1	Abce1	mmu:24015		
1478	-1.0814	0.5649	0.5233	-0.2612	0	O35309	N-myc-interactor	Nmi	mmu:64685		
1479	-0.0432	0.2965	-0.0791	-0.2607	0	Q8CI32	BAG family molecular chaperone regulator 5	Bag5	mmu:70369		
1480	-0.3000	0.2056	-0.2187	-0.2597	0	Q8R3V6	CUE domain-containing protein 1	Cuedc1	mmu:103841		
1481	-0.6315	-0.0025	0.0210	-0.2584	0	Q9R0N0	Galactokinase	Galk1	mmu:14635		
1482	-0.1597	0.9420	-0.6492	-0.2578	0	P70362	Ubiquitin fusion degradation protein 1 homolog	Ufd1l	mmu:22230		
1483	-0.5290	0.1917	0.2092	-0.2559	0	Q8C5H8	NAD kinase 2, mitochondrial	Nadk2	mmu:68646		
1484	-0.5130	0.2965	0.2240	-0.2553	0	Q8C3P7	N6-adenosine-methyltransferase subunit METTL3	Mettl3	mmu:56335		
1485	0.0530	-0.1124	-0.5703	-0.2553	0	Q9ERU9	E3 SUMO-protein ligase RanBP2	Ranbp2	mmu:19386		
1486	-0.8195	0.1148	0.1066	-0.2548	0	P62849	40S ribosomal protein S24	Rps24	mmu:20088		
1487	-0.2913	0.4295	-0.6913	-0.2547	0	Q3THK7	GMP synthase [glutamine-hydrolyzing]	Gmps	mmu:229363		
1488	0.1271	-0.0285	-0.8186	-0.2544	0	Q9CZU3	Superkiller viralicidic activity 2-like 2	Skiv2l2	mmu:72198		
1489	0.4873	-0.3337	-0.9611	-0.2543	0	Q61733	28S ribosomal protein S31, mitochondrial	Mrps31	mmu:57312		
1490	-0.2434	0.2164	-0.2840	-0.2536	0	Q6PDG5	SWI/SNF complex subunit SMARCC2	Smarcc2	mmu:68094		
1491	0.0054	0.0824	-0.9987	-0.2524	0	Q65Z40	Wings apart-like protein homolog	Wapal	mmu:218914		
1492	2.0030	0.0227	-0.0899	-0.2520	0	P43277	Histone H1,3	Hist1h1d	mmu:14957		
1493	-0.0859	0.6164	-0.3578	-0.2514	0	P59708	Splicing factor 3B subunit 6	Sf3b6	mmu:66055		
1494	0.5087	-0.1453	-0.9320	-0.2508	0	P21126	Ubiquitin-like protein 4A	Ubl4a	mmu:27643		
1495	-0.4438	-0.4468	0.0291	-0.2501	0	P49312	Heterogeneous nuclear ribonucleoprotein A1;Heterogeneous nuclear ribonucleoprotein A1, N-ter	Hnrnpa1	mmu:15382		
1496	-0.2532	-0.8792	1.0236	-0.2500	0	Q8BHL5	Engulfment and cell motility protein 2	Elmo2	mmu:140579		
1497	0.0253	0.4684	-0.5966	-0.2497	0	Q8K221	Arfaptin-2	Arfip2	mmu:76932		
1498	-0.4311	0.5556	0.4586	-0.2495	0	Q9CY58	Plasminogen activator inhibitor 1 RNA-binding protein	Serbp1	mmu:66870		
1499	0.0365	-0.1967	-0.0573	-0.2495	0	Q5SUR0	Phosphoribosylformylglycinamidine synthase	Pfas	mmu:237823		
1500	-0.5670	0.0653	0.3131	-0.2487	0	Q9CQU5	ZW10 interactor	Zwint	mmu:52696		
1501	-0.5559	-0.1033	0.1322	-0.2479	0	Q8C854	Myelin expression factor 2	Myef2	mmu:17876		

1502	0.2608	-0.3375	-0.7728	-0.2473	0	Q8BMS1	Trifunctional enzyme subunit alpha, mitochondrial;Long-chain enoyl-CoA hydratase;Long chain 3-h	Hadha	mmu:97212			
1503	-0.6698	0.3303	-0.2107	-0.2467	0	Q8BHE8	Uncharacterized protein C2orf47 homolog, mitochondrial		mmu:68115			
1504	0.0837	-0.2788	-0.1806	-0.2460	0	Q9D1K2	V-type proton ATPase subunit F	Atp6v1f	mmu:66144			
1505	-0.5947	-0.1701	0.1859	-0.2459	0	Q8VEH5	EPM2A-interacting protein 1	Epm2aip1	mmu:77781			
1506	0.6706	0.6529	-0.3163	-0.2448	0	Q3TEA8	Heterochromatin protein 1-binding protein 3	Hp1bp3	mmu:15441			
1507	-0.5775	0.1276	0.4405	-0.2447	0	Q7M6Y3	Phosphatidylinositol-binding clathrin assembly protein	Picalm	mmu:233489			
1508	-0.1228	[illegible]	0.3737	-0.2445	0	Q62084	Protein phosphatase 1 regulatory subunit 14B	Ppp1r14b	mmu:18938			
1509	-1.0995	0.5809	0.3201	-0.2404	0	O54946	DnaJ homolog subfamily B member 6	Dnajb6	mmu:23950			
1510	-0.2482	0.2905	-0.2261	-0.2391	0	P21278	Guanine nucleotide-binding protein subunit alpha-11	Gna11	mmu:14672			
1511	0.0269	-0.2521	-0.3941	-0.2387	0	Q61191	Host cell factor 1;HCF N-terminal chain 1;HCF N-terminal chain 2;HCF N-terminal chain 3;HCF N-te	Hcfc1	mmu:15161			
1512	0.4832	-0.2154	-0.1566	-0.2378	0	Q05920	Pyruvate carboxylase;Pyruvate carboxylase, mitochondrial	Pcx;Pc				
1513	2.0865	-0.3078	-0.6992	-0.2377	0	Q60710	Deoxynucleoside triphosphate triphosphohydrolase SAMHD1	Samhd1	mmu:56045			
1514	0.2789	1.2213	-0.5290	-0.2362	0	P98078	Disabled homolog 2	Dab2	mmu:13132			
1515	0.6802	1.1786	-2.7190	-0.2357	0	Q6P5D8	Structural maintenance of chromosomes flexible hinge domain-containing protein 1	Smchd1	mmu:74355			
1516	-0.4722	0.5641	0.0917	-0.2355	0	P97393	Rho GTPase-activating protein 5	Arhgap5				
1517	0.0068	-0.3014	0.2184	-0.2345	0	P47758	Signal recognition particle receptor subunit beta	Gm20425;S	mmu:20818			
1518	-0.6721	-0.3212	0.0598	-0.2339	0	Q91WK2	Eukaryotic translation initiation factor 3 subunit H	Eif3h	mmu:68135			
1519	0.3855	-0.6663	-0.5815	-0.2329	0	P19182	Interferon-related developmental regulator 1	Ifrd1	mmu:15982			
1520	0.1061	-0.2532	0.0115	-0.2328	0	O08756	3-hydroxyacyl-CoA dehydrogenase type-2	Hsd17b10				
1521	0.1264	-0.6302	-0.0657	-0.2326	0	Q9R0A0	Peroxisomal membrane protein PEX14	Pex14	mmu:56273			
1522	-0.2103	0.3631	-0.3642	-0.2325	0	Q91YD9	Neural Wiskott-Aldrich syndrome protein	Wasl	mmu:73178			
1523	-0.3077	-0.5212	0.0142	-0.2318	0	Q8JZS9	39S ribosomal protein L48, mitochondrial	Mrpl48	mmu:52443			
1524	-0.1610	0.5175	0.1062	-0.2313	0	Q921M7	Protein FAM49B	Fam49b	mmu:223601			
1525	0.1482	-0.7035	-0.0341	-0.2303	0	Q00493	Carboxypeptidase E	Cpe	mmu:12876			
1526	0.2031	0.1931	-0.1132	-0.2302	0	Q8K1A5	Transmembrane protein 41B	Tmem41b	mmu:233724			
1527	-0.1113	0.6819	-0.7769	-0.2295	0	Q8C5N3	Pre-mRNA-splicing factor CWC22 homolog	Cwc22;Gm	mmu:80744			
1528	-0.1330	0.1751	-0.0917	-0.2294	0	Q9WUK4	Replication factor C subunit 2	Rfc2	mmu:19718			
1529	0.0507	-0.4305	-0.4989	-0.2293	0	Q9JK23	Proteasome assembly chaperone 1	Psmg1	mmu:56088			
1530	-0.4768	0.4680	0.4050	-0.2284	0	Q6P1B1	Xaa-Pro aminopeptidase 1	Xpnpep1	mmu:170750			
1531	0.3890	0.0751	-0.4243	-0.2264	0	Q9DBR1	5-3 exoribonuclease 2	Xrn2	mmu:24128			
1532	-0.2455	0.6508	-0.5805	-0.2249	0	P13864	DNA (cytosine-5)-methyltransferase 1	Dnmt1	mmu:13433			
1533	-0.0322	-0.0202	0.4770	-0.2243	0	Q99J36	THUMP domain-containing protein 1	Thumpd1	mmu:233802			
1534	0.4679	-0.5143	-0.9500	-0.2223	0	Q9D7H3	RNA 3-terminal phosphate cyclase	RtcA	mmu:66368			
1535	0.2654	-2.0424	0.7392	-0.2216	0	Q9JM05	E3 SUMO-protein ligase PIAS4	Pias4	mmu:59004			
1536	0.2314	-0.4155	0.2371	-0.2190	0	Q8BWW4	La-related protein 4	Larp4	mmu:207214			
1537	-1.7497	0.1992	0.9943	-0.2179	0	Q9R061	Cytosolic Fe-S cluster assembly factor NUBP2	Nubp2	mmu:26426			
1538	0.3350	-0.0188	0.2618	-0.2173	0	P47856	Glutamine--fructose-6-phosphate aminotransferase [isomerizing] 1	Gfpt1	mmu:14583			
1539	-0.1750	-0.3029	0.0991	-0.2171	0	Q14B01		Rnf113a2	mmu:66381			
1540	-0.5959	0.1668	0.1828	-0.2170	0	Q91VA7	Isocitrate dehydrogenase [NAD] subunit, mitochondrial	Idh3b	mmu:170718			
1541	0.3105	-0.3363	-0.0269	-0.2168	0	Q8VDC1	FYVE and coiled-coil domain-containing protein 1	Fyco1	mmu:17281			
1542	0.3682	0.6202	-0.5362	-0.2167	0	Q6DFW4	Nucleolar protein 58	Nop58	mmu:55989			
1543	-0.2324	0.3208	-0.2202	-0.2166	0	Q9WTP6	Adenylate kinase 2, mitochondrial;Adenylate kinase 2, mitochondrial, N-terminally processed	Ak2	mmu:11637			
1544	0.9270	0.2160	-0.3789	-0.2165	0	P61089	Ubiquitin-conjugating enzyme E2 N	Ube2n	mmu:93765		1	
1545	-0.4741	-0.2631	0.7361	-0.2155	0	F6TFN2		Lmo7				
1546	-0.0190	-0.3739	0.1764	-0.2155	0	O08585	Clathrin light chain A	Clta				
1547	1.4858	-0.1961	0.5378	-0.2151	0	P43276	Histone H1,5	Hist1h1b	mmu:56702			
1548	-0.3472	0.2111	0.0410	-0.2148	0	P17182	Alpha-enolase;Enolase	Eno1	mmu:13806			
1549	0.5964	0.5275	-0.1595	-0.2147	0	O88952	Protein lin-7 homolog C	Lin7c	mmu:22343			
1550	-1.3947	0.6113	0.8043	-0.2125	0	Q9EQP2	EH domain-containing protein 4	Ehd4	mmu:98878			
1551	-0.0964	0.2213	-0.5082	-0.2120	0	O08579	Emerin	Emd	mmu:13726			
1552	0.0512	-0.3482	-0.4821	-0.2115	0	E9PYG6		Rasa1	mmu:218397			
1553	-0.1228	0.2737	-0.5440	-0.2109	0	Q6PD28		Ppp2r5b	mmu:225849			
1554	0.5189	-0.2948	0.3114	-0.2109	0	Q8CI51	PDZ and LIM domain protein 5	Pdlim5	mmu:56376			
1555	-0.6326	-0.3820	0.3365	-0.2105	0	P30681	High mobility group protein B2	Hmgb2	mmu:97165			
1556	-0.3612	0.0655	-0.2107	-0.2091	0	Q3UQ28	Peroxidasin homolog	Pxdn	mmu:69675	1		
1557	-0.9872	0.7138	-0.1867	-0.2082	0	Q9D1I6	39S ribosomal protein L14, mitochondrial	Mrpl14	mmu:68463			
1558	0.0885	-0.3394	1.0478	-0.2076	0	P56959	RNA-binding protein FUS	Fus	mmu:233908			
1559	-0.0224	-0.1096	0.3062	-0.2070	0	Q8CEE7	Retinol dehydrogenase 13	Rdh13	mmu:108841			
1560	-0.4057	0.2177	0.3117	-0.2059	0	Q9WV34	MAGUK p55 subfamily member 2	Mpp2	mmu:50997			
1561	-0.2758	-0.1594	0.1725	-0.2055	0	Q8BTS4	Nuclear pore complex protein Nup54	Nup54	mmu:269113			
1562	0.0089	0.2962	-0.1905	-0.2052	0	Q9DAW6	U4/U6 small nuclear ribonucleoprotein Prp4	Prpf4	mmu:70052			
1563	-0.8085	0.5531	-0.2918	-0.2047	0	P58059	28S ribosomal protein S21, mitochondrial	Mrps21	mmu:66292			
1564	-0.1720	0.2699	-1.2898	-0.2047	0	Q9Z0F6	Cell cycle checkpoint control protein RAD9A	Rad9a	mmu:19367			
1565	-0.2972	0.5608	-1.1034	-0.2045	0	Q5SXJ3	Fanconi anemia group J protein homolog	Brip1	mmu:237911			
1566	-0.8658	0.1500	-0.0661	-0.2044	0	Q07417	Short-chain specific acyl-CoA dehydrogenase, mitochondrial	Acads	mmu:11409			
1567	-0.5110	-0.1421	0.2024	-0.2035	0	Q9WV92	Band 4,1-like protein 3;Band 4,1-like protein 3, N-terminally processed	Epb41l3	mmu:13823			
1568	-0.3219	-0.0264	0.2061	-0.2033	0	Q5SUQ9	CST complex subunit CTC1	Ctc1	mmu:68964			
1569	-0.3786	0.0713	-0.1129	-0.2033	0	Q91VR5	ATP-dependent RNA helicase DDX1	Ddx1	mmu:104721			
1570	-0.2789	-0.3970	0.0158	-0.2017	0	Q6P9P6	Kinesin-like protein KIF11	Kif11	mmu:16551			
1571	-0.8185	0.9698	-1.8848	-0.1999	0	Q6DFV1	Condensin-2 complex subunit G2	Ncapg2	mmu:76044			
1572	0.1914	-0.0426	-0.2621	-0.1992	0	P40142	Transketolase	Tkt	mmu:21881			
1573	0.0413	-0.5079	-0.7113	-0.1991	0	Q8BQ47	Protein canopy homolog 4	Cnpy4	mmu:66455			
1574	0.6877	-0.5647	-0.2640	-0.1975	0	Q8BYL4	Tyrosine--tRNA ligase, mitochondrial	Yars2	mmu:70120			
1575	0.6971	-0.5797	-0.1963	-0.1968	0	P62317	Small nuclear ribonucleoprotein Sm D2	Snrpd2	mmu:107686			
1576	0.2035	-0.0522	-0.2291	-0.1957	0	Q03265	ATP synthase subunit alpha, mitochondrial;ATP synthase subunit alpha	Atp5a1	mmu:11946			
1577	0.6695	-0.6996	0.1747	-0.1947	0	Q3TJZ6	Protein FAM98A	Fam98a	mmu:72722			
1578	-0.4218	0.5630	-1.0038	-0.1939	0	Q6A026	Sister chromatid cohesion protein PDS5 homolog A	Pds5a				
1579	-0.2621	-0.9073	0.0884	-0.1932	0	P35585	AP-1 complex subunit mu-1	Ap1m1	mmu:11767			
1580	0.0784	0.3054	-0.4923	-0.1925	0	Q9ERA6	Tuftelin-interacting protein 11	Tfip11	mmu:54723			
1581	-0.3602	0.4071	0.7291	-0.1922	0	P09411	Phosphoglycerate kinase 1;Phosphoglycerate kinase	Pgk1	mmu:18655			
1582	-0.0775	0.0740	-0.9664	-0.1915	0	Q99K28	ADP-ribosylation factor GTPase-activating protein 2	Arfgap2	mmu:77038			
1583	-0.2387	-1.4725	0.0869	-0.1907	0	O54941	SWI/SNF-related matrix-associated actin-dependent regulator of chromatin subfamily E member 1	Smarce1	mmu:57376			
1584	-0.3066	0.2182	-1.5656	-0.1905	0	F7BJB9		Morc3	mmu:338467			
1585	-0.0860	0.1235	-1.7282	-0.1903	0	G3X9B1		Heatr1	mmu:217995			
1586	-0.8011	-0.3419	0.8254	-0.1903	0	P14069	Protein S100-A6	S100a6	mmu:20200			
1587	-0.4723	0.0385	-0.8955	-0.1900	0	P26369	Splicing factor U2AF 65 kDa subunit	U2af2	mmu:22185			
1588	0.0228	-0.5642	-0.4064	-0.1896	0	Q8CJ26	Death domain-containing membrane protein NRADD	Nradd	mmu:67169			
1589	-0.2373	0.1420	-0.2761	-0.1890	0	Q6ZPS6	Ankyrin repeat and IBR domain-containing protein 1	Ankib1	mmu:70797			
1590	-0.1526	0.1521	-0.1735	-0.1888	0	Q9CR27	WASH complex subunit CCDC53	Ccdc53	mmu:67282			
1591	-0.3464	0.1642	0.2592	-0.1885	0	Q99JX7	Nuclear RNA export factor 1	Nxf1				
1592	-0.3351	0.9832	0.1176	-0.1884	0	Q99K85	Phosphoserine aminotransferase	Psat1	mmu:107272			
1593	0.0313	-0.1178	-0.4427	-0.1880	0	Q8C7E9	Cleavage stimulation factor subunit 2 tau variant	Cstf2t	mmu:83410			
1594	0.6074	-0.1834	-0.0773	-0.1859	0	Q8BFR5	Elongation factor Tu, mitochondrial	Tufm	mmu:233870			
1595	-1.1439	-1.6922	0.5490	-0.1857	0	P97440	Histone RNA hairpin-binding protein	Slbp	mmu:20492			
1596	-0.4285	-0.0120	0.1794	-0.1855	0	Q9WU78	Programmed cell death 6-interacting protein	Pdcd6ip	mmu:18571			
1597	-0.0201	0.1352	0.1010	-0.1855	0	Q60692	Proteasome subunit beta type-6	Psmb6	mmu:19175			
1598	0.0217	-0.0640	-0.0241	-0.1854	0	Q99KV1	DnaJ homolog subfamily B member 11	Dnajb11	mmu:67838			
1599	0.3661	-0.0476	0.3454	-0.1848	0	Q3UFY8	Mitochondrial ribonuclease P protein 1	Trmt10c	mmu:52575			
1600	-0.1411	0.8764	-1.1436	-0.1847	0	Q61048	WW domain-binding protein 4	Wbp4	mmu:22380			
1601	-0.0184	-0.0756	0.2857	-0.1845	0	E9Q5C9		Nolc1				
1602	-0.3435	0.6187	0.7806	-0.1837	0	Q9CZX8	40S ribosomal protein S19	Rps19	mmu:20085			
1603	0.6778	0.2914	-0.1461	-0.1836	0	Q8CF66	Ragulator complex protein LAMTOR4;Ragulator complex protein LAMTOR4, N-terminally processe	Lamtor4	mmu:66096			
1604	0.1679	-0.1784	-0.3120	-0.1835	0	P16546	Spectrin alpha chain, non-erythrocytic 1	Sptan1				
1605	-0.0427	0.1026	-0.6294	-0.1833	0	Q64133	Amine oxidase [flavin-containing] A	Maoa	mmu:17161			
1606	-1.3211	0.0511	0.1324	-0.1828	0	Q8BQ30	Phostensin	Ppp1r18	mmu:76448			
1607	-0.1677	0.7069	-0.1505	-0.1814	0	Q9D287	Pre-mRNA-splicing factor SPF27	Bcas2	mmu:68183			
1608	-0.0740	0.2855	0.3337	-0.1799	0	P56812	Programmed cell death protein 5	Pdcd5	mmu:56330			1
1609	-0.6252	1.1093	0.0287	-0.1793	0	Q8BSQ9	Protein polybromo-1	Pbrm1				
1610	0.1324	0.3652	-1.0550	-0.1788	0	Q8BH59	Calcium-binding mitochondrial carrier protein Aralar1	Slc25a12	mmu:78830			
1611	0.4469	-0.0752	-0.1123	-0.1788	0	Q8CGC4	Protein LSM14 homolog B	Lsm14b	mmu:241846			
1612	-0.3189	0.1833	-0.2425	-0.1788	0	Q1HFZ0	tRNA (cytosine(34)-C(5))-methyltransferase	Nsun2	mmu:28114			
1613	0.8222	-0.7489	-0.9028	-0.1781	0	P02469	Laminin subunit beta-1	Lamb1				
1614	-0.0489	0.3687	-0.6453	-0.1777	0	Q9ESZ8	General transcription factor II-I	Gtf2i	mmu:14886			
1615	0.0680	-0.6529	0.0816	-0.1764	0	Q3ULJ0	Glycerol-3-phosphate dehydrogenase 1-like protein	Gpd1l	mmu:333433			
1616	-0.0685	-0.7993	0.6385	-0.1741	0	Q8BVF2	Phosducin-like protein 3	Pdcl3	mmu:68833			
1617	-0.8230	1.0637	1.7597	-0.1736	0	Q8C6L5	Cyclic GMP-AMP synthase	Mb21d1	mmu:214763			
1618	-0.0589	0.4260	-0.6093	-0.1730	0	Q9CXY6	Interleukin enhancer-binding factor 2	Ilf2	mmu:67781			
1619	0.2232	-0.5383	-0.5199	-0.1729	0	Q3UM18	Large subunit GTPase 1 homolog	Lsg1	mmu:224092			
1620	-0.1144	0.2182	-0.3244	-0.1726	0	Q91V89		Ppp2r5d	mmu:21770			
1621	0.0964	-0.3242	-0.8318	-0.1723	0	Q91VD9	NADH-ubiquinone oxidoreductase 75 kDa subunit, mitochondrial	Ndufs1	mmu:227197			
1622	0.0376	0.1124	-0.1127	-0.1719	0	Q9QXS1	Plectin	Plec	mmu:18810			
1623	-0.2324	-2.2805	0.1168	-0.1719	0	Q60766	Immunity-related GTPase family M protein 1	Irgm1	mmu:15944			
1624	0.0738	-0.1955	-0.0677	-0.1711	0	O89090	Transcription factor Sp1	Sp1	mmu:20683			
1625	-0.0794	0.0906	-0.3880	-0.1711	0	P17918	Proliferating cell nuclear antigen	Pcna	mmu:18538			
1626	-0.5696	0.5043	0.5542	-0.1704	0	Q9WV03	Protein FAM50A	Fam50a	mmu:108160			
1627	-0.8171	0.5318	0.2616	-0.1703	0	Q61545	RNA-binding protein EWS	Ewsr1	mmu:14030			

1628	-0.6699	-0.8457	0.1148	-0.1702	0	Q924T2	28S ribosomal protein S2, mitochondrial	Mrps2	mmu:118451				
1629	-0.4298	0.7514	-1.3194	-0.1701	0	Q8R2K4	TAF6-like RNA polymerase II p300/CBP-associated factor-associated factor 65 kDa subunit 6L	Taf6l	mmu:225895				
1630	[illegible]	-0.7139	0.3329	-0.1701	0	A0A0J9YTR2							
1631	-0.1305	1.2353	-0.7732	-0.1694	0	Q9JKX4	Protein AATF	Aatf	mmu:56321				1
1632	-0.2414	0.3989	0.7799	-0.1683	0	P26041	Moesin	Msn	mmu:17698				
1633	-0.9023	0.5759	-0.1362	-0.1679	0	Q6PDM2	Serine/arginine-rich splicing factor 1	Srsf1	mmu:110809				
1634	-0.3807	0.4022	-0.1532	-0.1678	0	Q9Z1X4	Interleukin enhancer-binding factor 3	Ilf3	mmu:16201				
1635	-0.5722	0.5088	-0.9354	-0.1662	0	Q99LG2	Transportin-2	Tnpo2	mmu:212999				
1636	0.0177	-0.9188	-0.1430	-0.1658	0	Q9CZW5	Mitochondrial import receptor subunit TOM70	Tomm70a	mmu:28185				
1637	0.2931	-0.0721	-0.2917	-0.1651	0	Q8BGC0	HIV Tat-specific factor 1 homolog	Htatsf1	mmu:72459				
1638	-0.9989	0.4453	-0.2862	-0.1644	0	Q2HXL6	alpha-1,2-Mannosidase;ER degradation-enhancing alpha-mannosidase-like protein 3	Edem3	mmu:66967				
1639	-2.3062	0.1036	1.0238	-0.1631	0	Q6PIP5	NudC domain-containing protein 1	Nudcd1	mmu:67429				
1640	[illegible]	-0.0668	-0.4615	-0.1620	0	P70388	DNA repair protein RAD50	Rad50					
1641	-0.0560	0.6829	0.1388	-0.1617	0	Q8CHY6	Transcriptional repressor p66 alpha	Gatad2a	mmu:234366				
1642	1.1972	-1.2539	0.2800	-0.1617	0	Q8R3S6	Exocyst complex component 1	Exoc1	mmu:69940				
1643	0.3623	-0.2456	-0.2582	-0.1610	0	Q9DC61	Mitochondrial-processing peptidase subunit alpha	Pmpca	mmu:66865				
1644	-0.9810	0.2032	0.4023	-0.1608	0	O89086	RNA-binding protein 3	Rbm3	mmu:19652				
1645	-0.0828	-0.2569	1.0887	-0.1605	0	P70441	Na(+)/H(+) exchange regulatory cofactor NHE-RF1	Slc9a3r1	mmu:26941				
1646	-0.4828	0.1998	-0.1026	-0.1591	0	Q99NH0	Ankyrin repeat domain-containing protein 17	Ankrd17	mmu:81702				
1647	0.3585	0.9534	-0.3294	-0.1588	0	Q810D6	Glutamate-rich WD repeat-containing protein 1	Grwd1	mmu:101612				
1648	0.0182	-1.0774	-0.3820	-0.1587	0	Q9CRD0	OCIA domain-containing protein 1	Ociad1	mmu:68095				
1649	-0.8156	0.8940	-0.6784	-0.1586	0	Q61937	Nucleophosmin	Npm1;Gm!	mmu:18148				1
1650	0.5187	-0.2107	-0.2873	-0.1586	0	Q3UV70	[Pyruvate dehydrogenase [acetyl-transferring]]-phosphatase 1, mitochondrial	Pdp1	mmu:381511		1		
1651	-0.0692	0.1588	-0.4074	-0.1586	0	Q8BRF7	Sec1 family domain-containing protein 1	Scfd1	mmu:76983				
1652	0.1439	0.9366	-1.4535	-0.1584	0	Q8K2C7	Protein OS-9	Os9	mmu:216440				
1653	0.0257	-0.6195	-0.6612	-0.1580	0	G5E8K5	Ankyrin-3	Ank3	mmu:11735				
1654	0.0721	0.3513	-0.0633	-0.1578	0	Q9CQI3	Glia maturation factor beta	Gmfb	mmu:63985				
1655	[illegible]	0.1826	-0.6145	-0.1568	0	Q8K298	Actin-binding protein anillin	Anln	mmu:68743				
1656	0.5044	0.3899	-0.5389	-0.1564	0	O70305	Ataxin-2	Atxn2	mmu:20239				
1657	-0.1679	-0.1701	0.3857	-0.1559	0	Q8BK58	HSPB1-associated protein 1	Hspbap1	mmu:66667				
1658	0.0089	0.0394	-0.0929	-0.1554	0	O35887	Calumenin	Calu	mmu:12321				
1659	0.3901	-0.5041	-0.2013	-0.1539	0	Q9CU62	Structural maintenance of chromosomes protein 1A	Smc1a	mmu:24061				
1660	-0.4483	0.3869	0.6175	-0.1520	0	Q8C2Q3	RNA-binding protein 14	Rbm14	mmu:56275				
1661	-0.0213	-0.0372	0.4062	-0.1519	0	Q02053	Ubiquitin-like modifier-activating enzyme 1	Uba1	mmu:22201			1	
1662	-0.0606	0.3534	0.4532	-0.1518	0	Q9Z1K5	E3 ubiquitin-protein ligase ARIH1	Arih1	mmu:23806			1	
1663	-0.8795	0.1372	0.4070	-0.1518	0	Q9CZV8	F-box/LRR-repeat protein 20	Fbxl20	mmu:72194				
1664	0.2574	0.4774	-0.2339	-0.1516	0	Q9DBY8	Nuclear valosin-containing protein-like	Nvl	mmu:67459				
1665	0.3005	0.3827	-0.1375	-0.1511	0	Q9DC51	Guanine nucleotide-binding protein G(k) subunit alpha	Gnai3	mmu:14679				
1666	0.0283	0.5937	-2.4988	-0.1510	0	Q7TNV0	Protein DEK	Dek	mmu:110052				
1667	0.0161	0.4018	-0.4561	-0.1505	0	Q61033	Lamina-associated polypeptide 2, isoforms alpha/zeta	Tmpo					
1668	1.2456	-0.2032	-0.4052	-0.1495	0	Q91VM5	RNA binding motif protein, X-linked-like-1	Rbmxl1	mmu:19656				
1669	0.5188	-0.3129	-0.3852	-0.1494	0	A2ADY9	Protein DDI1 homolog 2	Ddi2	mmu:68817				
1670	-0.0493	0.1540	-0.1586	-0.1488	0	Q8CGB6	Tensin-2	Tns2	mmu:209039		1		
1671	-0.0557	0.2170	0.5066	-0.1480	0	Q3U2I3	FTS and Hook-interacting protein	Fam160a2	mmu:74349				
1672	-0.3536	0.2484	-0.1596	-0.1475	0	Q9ERN0	Secretory carrier-associated membrane protein 2	Scamp2	mmu:24044				
1673	0.2145	-0.2326	-0.0710	-0.1474	0	P26039	Talin-1	Tln1	mmu:21894				
1674	-0.3219	1.4464	-1.6555	-0.1471	0	Q62241	U1 small nuclear ribonucleoprotein C	Snrpc	mmu:20630				
1675	-0.4277	0.2418	0.4705	-0.1469	0	Q61656	Probable ATP-dependent RNA helicase DDX5	Ddx5					
1676	-1.4461	0.3597	0.2087	-0.1465	0	Q8CIB5	Fermitin family homolog 2	Fermt2	mmu:218952				
1677	-0.1737	0.1485	-0.2661	-0.1462	0	Q9Z1Z2	Serine-threonine kinase receptor-associated protein	Strap	mmu:20901				
1678	-0.5113	0.1583	-0.4140	-0.1461	0	Q9CYI4	Putative RNA-binding protein Luc7-like 1	Luc7l	mmu:66978				
1679	0.2010	-0.7592	-0.6035	-0.1460	0	Q5SRX1	TOM1-like protein 2	Tom1l2	mmu:216810				
1680	0.2420	0.5229	[illegible]	-0.1458	0	Q3TLH4	Protein PRRC2C	Prrc2c	mmu:226562				
1681	-0.1785	0.0624	-0.4339	-0.1458	0	Q6ZQ08	CCR4-NOT transcription complex subunit 1	Cnot1	mmu:234594				
1682	0.4706	-0.2693	-1.0425	-0.1446	0	O54774	AP-3 complex subunit delta-1	Ap3d1	mmu:11776				
1683	0.0196	0.0911	-0.7613	-0.1442	0	P51881	ADP/ATP translocase 2;ADP/ATP translocase 2, N-terminally processed	Slc25a5	mmu:11740				
1684	0.3528	0.5693	-0.9400	-0.1435	0	P0DMN7	S-adenosylmethionine decarboxylase proenzyme 1;S-adenosylmethionine decarboxylase 1 alpha c	Amd1;Amc	mmu:11702				
1685	0.2280	-0.2405	-0.5788	-0.1421	0	P47802	Metaxin-1	Mtx1					
1686	-0.0429	-0.8975	1.2408	-0.1419	0	E9Q6U4	Prefoldin subunit 4	Pfdn4					
1687	-0.6856	0.2825	-0.0353	-0.1417	0	Q8C9B9	Death-inducer obliterator 1	Dido1	mmu:23856				
1688	0.5286	-2.2344	1.0691	-0.1416	0	Q69ZQ2	Pre-mRNA-splicing factor ISY1 homolog	Isy1	mmu:57905				
1689	0.0539	0.1828	-0.2610	-0.1409	0	Q14C51	Pentatricopeptide repeat domain-containing protein 3, mitochondrial	Ptcd3	mmu:69956				
1690	1.6134	0.2353	-0.1816	-0.1405	0	P10922	Histone H1,0;Histone H1,0, N-terminally processed	H1f0	mmu:14958				
1691	0.4528	-0.3490	-0.3207	-0.1405	0	Q3TWV4		Ap2m1					
1692	0.0757	-0.2862	0.0324	-0.1400	0	O08795	Glucosidase 2 subunit beta	Prkcsh	mmu:19089				
1693	0.1165	-0.1924	[illegible]	-0.1396	0	E9Q3G8		Nup153	mmu:218210				
1694	-0.0628	0.3649	-0.7623	-0.1394	0	Q80X71	Transmembrane protein 106B	Tmem106l	mmu:71900				
1695	0.6430	-0.3905	-2.0623	-0.1393	0	Q9CQC9	GTP-binding protein SAR1b	Sar1b	mmu:66397				
1696	-0.2120	0.2436	0.1669	-0.1391	0	P30999	Catenin delta-1	Ctnnd1	mmu:12388				
1697	-0.1588	0.4661	0.1335	-0.1391	0	Q501J6	Probable ATP-dependent RNA helicase DDX17	Ddx17	mmu:67040				
1698	-0.5945	0.0913	-0.5152	-0.1383	0	Q91VY9	Zinc finger protein 622	Znf622	mmu:52521	1			
1699	0.2862	-0.5076	0.1405	-0.1379	0	Q8BYY4	Tetratricopeptide repeat protein 39B	Ttc39b	mmu:69863				
1700	0.5380	-0.0594	-1.0705	-0.1373	0	Q6PG16	Holliday junction recognition protein	Hjurp	mmu:212427				
1701	-0.3510	-0.4144	0.2069	-0.1372	0	Q923D5	WW domain-binding protein 11	Wbp11	mmu:60321				
1702	-0.3552	-0.2411	0.2120	-0.1352	0	Q9D1J3	SAP domain-containing ribonucleoprotein	Sarnp	mmu:66118				
1703	-1.0800	0.3292	0.3354	-0.1351	0	O55236	mRNA-capping enzyme;Polynucleotide 5-triphosphatase;mRNA guanylyltransferase	Rngtt	mmu:24018		1		
1704	-0.2055	-1.0095	0.2698	-0.1345	0	Q8BGW1	Alpha-ketoglutarate-dependent dioxygenase FTO	Fto	mmu:26383				
1705	0.5843	-0.3484	-0.7572	-0.1344	0	Q9CQQ7	ATP synthase F(0) complex subunit B1, mitochondrial	Atp5f1	mmu:11950				
1706	-0.7407	0.2283	0.0810	-0.1342	0	P09405	Nucleolin	Ncl	mmu:17975				
1707	0.7350	0.6658	-0.2661	-0.1337	0	Q6KAR6	Exocyst complex component 3	Exoc3	mmu:211446				
1708	-0.0127	-0.4906	0.2375	-0.1337	0	Q9JMA1	Ubiquitin carboxyl-terminal hydrolase 14;Ubiquitin carboxyl-terminal hydrolase	Usp14	mmu:59025				
1709	-0.5987	0.5539	-0.0416	-0.1330	0	Q921J4	Ubiquitin-conjugating enzyme E2 S	Ube2s	mmu:77891			1	
1710	-0.4364	-0.5966	0.1397	-0.1329	0	Q9QYS9	Protein quaking	Qki	mmu:19317				
1711	-1.2485	0.0395	1.0628	-0.1321	0	Q8VHK9	ATP-dependent RNA helicase DHX36	Dhx36	mmu:72162				
1712	-0.2630	0.0664	0.2783	-0.1313	0	Q99K51	Plastin-3	Pls3	mmu:102866				
1713	-0.4114	0.2436	-0.1738	-0.1312	0	Q91XD6	Vacuolar protein-sorting-associated protein 36	Vps36	mmu:70160				
1714	0.0469	0.0618	-0.1665	-0.1309	0	Q9D2R8	28S ribosomal protein S33, mitochondrial	Mrps33	mmu:14548				
1715	-0.5412	0.2498	-0.2706	-0.1302	0	P07141	Macrophage colony-stimulating factor 1;Processed macrophage colony-stimulating factor 1	Csf1	mmu:12977				
1716	-0.0753	0.3042	-0.7079	-0.1301	0	Q6A068	Cell division cycle 5-like protein	Cdc5l	mmu:71702				
1717	0.1905	0.2250	-0.1353	-0.1300	0	Q9D5V5	Cullin-5	Cul5	mmu:75717			1	1
1718	0.1199	-0.2356	0.6523	-0.1297	0	Q6A028	Switch-associated protein 70	Swap70	mmu:20947				
1719	0.1575	0.3055	-0.6215	-0.1296	0	Q8R323	Replication factor C subunit 3	Rfc3	mmu:69263				
1720	-0.3715	0.6294	0.4431	-0.1292	0	P61967	AP-1 complex subunit sigma-1A	Ap1s1	mmu:11769				
1721	-0.2716	0.8081	-0.8856	-0.1291	0	Q8R3N6	THO complex subunit 1	Thoc1	mmu:225160				
1722	0.2393	-0.5977	-0.1352	-0.1287	0	Q6PDY2	2-aminoethanethiol dioxygenase	Ado	mmu:211488				
1723	-0.0073	0.1521	-1.3952	-0.1286	0	O35218	Cleavage and polyadenylation specificity factor subunit 2	Cpsf2	mmu:51786				
1724	-0.0917	0.4912	-0.0286	-0.1283	0	Q3TB82	Pleckstrin homology domain-containing family F member 1	Plekhf1	mmu:72287				1
1725	-0.5516	0.1668	0.4391	-0.1282	0	Q8BT07	Centrosomal protein of 55 kDa	Cep55	mmu:74107				
1726	-0.8821	0.0575	-0.2822	-0.1282	0	Q9WU56	tRNA pseudouridine synthase A, mitochondrial;tRNA pseudouridine synthase	Pus1	mmu:56361				
1727	-0.6048	0.5580	-0.0327	-0.1277	0	Q8BJW5	Nucleolar protein 11	Nol11	mmu:68979				
1728	-0.0451	0.7268	-0.5408	-0.1271	0	Q60930	Voltage-dependent anion-selective channel protein 2	Vdac2	mmu:22334				
1729	-0.0730	0.4390	-0.1639	-0.1270	0	Q91VC3	Eukaryotic initiation factor 4A-III;Eukaryotic initiation factor 4A-III, N-terminally processed	Eif4a3;Gm!	mmu:192170				
1730	0.2450	-0.0931	-0.2293	-0.1242	0	Q80XS6	Protein Smaug homolog 2	Samd4b					
1731	-0.2182	-0.0458	0.0737	-0.1232	0	D3YZP9	Coiled-coil domain-containing protein 6	Ccdc6	mmu:76551				
1732	-0.2003	0.4056	-0.4625	-0.1229	0	Q62376	U1 small nuclear ribonucleoprotein 70 kDa	Snrnp70	mmu:20637				
1733	-0.1225	0.3150	0.6745	-0.1221	0	Q7TQH0	Ataxin-2-like protein	Atxn2l	mmu:233871				
1734	0.2395	-0.0440	-0.0946	-0.1219	0	Q922R5	Serine/threonine-protein phosphatase 4 regulatory subunit 3B	Smek2	mmu:104570				
1735	0.0666	-1.0563	-0.2744	-0.1210	0	Q80UJ7	Rab3 GTPase-activating protein catalytic subunit	Rab3gap1	mmu:226407				
1736	0.5864	0.2749	-0.7332	-0.1205	0	Q9JI90	E3 ubiquitin-protein ligase RNF14	Rnf14	mmu:56736			1	
1737	0.6293	0.0773	-0.3690	-0.1205	0	P09103	Protein disulfide-isomerase	P4hb	mmu:18453				
1738	-0.1834	-0.2834	0.3059	-0.1194	0	Q99KQ4	Nicotinamide phosphoribosyltransferase	Nampt	mmu:59027				
1739	0.1822	-0.3426	0.4118	-0.1187	0	Q80U93	Nuclear pore complex protein Nup214	Nup214	mmu:227720				
1740	-0.4125	0.2423	-0.1930	-0.1187	0	A2AKG8	Focadhesin	Focad	mmu:230393				
1741	-0.0317	-0.3009	0.1540	-0.1177	0	P48193	Protein 4,1	Epb4,1;Epl	mmu:269587				
1742	-0.0815	-0.4121	0.0714	-0.1175	0	Q8VBV3	Exosome complex component RRP4	Exosc2	mmu:227715				
1743	0.0236	-0.1485	-0.1785	-0.1170	0	Q8BML9		Qars	mmu:97541				
1744	0.0394	-0.0193	-0.3413	-0.1165	0	Q8R0G9	Nuclear pore complex protein Nup133	Nup133	mmu:234865				
1745	-0.7192	0.2984	-0.2292	-0.1164	0	Q61140	Breast cancer anti-estrogen resistance protein 1	Bcar1	mmu:12927				
1746	[illegible]	-0.1047	-0.7349	-0.1163	0	E9Q0A3		Arhgef11					
1747	-0.7901	1.1488	0.3267	-0.1162	0	Q9DCT8	Cysteine-rich protein 2	Crip2	mmu:68337				
1748	-1.9909	0.4696	-0.4531	-0.1160	0	P53702	Cytochrome c-type heme lyase	Hccs	mmu:15159				
1749	-0.1880	0.1756	[illegible]	-0.1159	0	Q9D0M1	Phosphoribosyl pyrophosphate synthase-associated protein 1	Prpsap1	mmu:67763				
1750	0.1868	-0.4781	0.2985	-0.1157	0	Q9D0I9	Arginine--tRNA ligase, cytoplasmic	Rars	mmu:104458				
1751	0.3407	-0.5093	-0.3802	-0.1151	0	O08582	GTP-binding protein 1	Gtpbp1	mmu:14904				
1752	0.1406	0.3176	-0.4754	-0.1145	0	P08752	Guanine nucleotide-binding protein G(i) subunit alpha-2	Gnai2	mmu:14678				
1753	-0.8483	-0.2481	0.8424	-0.1144	0	P60335	Poly(rC)-binding protein 1	Pcbp1	mmu:23983				

1754	0.0613	-0.3354	-0.4338	-0.1141	0	Q8VEM8	Phosphate carrier protein, mitochondrial	Slc25a3	mmu:18674				
1755	0.1455	-0.5358	-0.0240	-0.1139	0	Q9JMH9	Unconventional myosin-XVIIIa	Myo18a	mmu:360013				1
1756	[illegible]	0.2384	-0.0610	-0.1138	0	Q08943	FACT complex subunit SSRP1	Ssrp1	mmu:20833				
1757	-0.3651	-0.3557	0.1620	-0.1135	0	Q99KJ8	Dynactin subunit 2	Dctn2	mmu:69654				
1758	-1.3621	1.0419	1.0079	-0.1133	0	Q9D7X3	Dual specificity protein phosphatase 3	Dusp3	mmu:72349		1		
1759	0.5472	0.1244	-0.4648	-0.1125	0	Q9Z2A5	Arginyl-tRNA--protein transferase 1	Ate1	mmu:11907				
1760	[illegible]	-0.1890	-0.7217	-0.1120	0	F8VQ29		Iqgap3	mmu:404710				
1761	0.2342	-0.2720	0.1999	-0.1117	0	O08788	Dynactin subunit 1	Dctn1	mmu:13191				
1762	0.2778	0.1634	-0.3064	-0.1105	0	Q3V1L4	Cytosolic purine 5-nucleotidase	Nt5c2	mmu:76952				
1763	0.2787	0.1384	-0.7391	-0.1103	0	Q8C0T5	Signal-induced proliferation-associated 1-like protein 1	Sipa1l1	mmu:217692				
1764	0.0485	-0.4436	-0.0609	-0.1096	0	P24547	Inosine-5-monophosphate dehydrogenase 2	Impdh2	mmu:23918				
1765	0.1726	0.0356	-0.3183	-0.1085	0	Q8K2Z2	Pre-mRNA-processing factor 39	Prpf39					
1766	0.4540	-0.1736	0.5138	-0.1082	0	Q6NZL6	Tonsoku-like protein	Tonsl	mmu:72749				
1767	0.1140	-0.0654	-0.6700	-0.1074	0	P13439	Uridine 5-monophosphate synthase;Orotate phosphoribosyltransferase;Orotidine 5-phosphate de	Umps	mmu:22247				
1768	0.3640	-0.4758	-0.2693	-0.1073	0	Q8BJ05	Zinc finger CCCH domain-containing protein 14	Zc3h14	mmu:75553				
1769	[illegible]	0.2386	-1.1322	-0.1048	0	Q9Z1N5	Spliceosome RNA helicase Ddx39b	Ddx39b	mmu:53817				
1770	-0.7688	0.5288	-0.4155	-0.1045	0	Q8BZR9	Uncharacterized protein C17orf85 homolog		mmu:66874				
1771	-0.0143	-0.1788	0.0569	-0.1044	0	Q60790	Ras GTPase-activating protein 3	Rasa3	mmu:19414				
1772	0.2397	-0.3086	-0.6987	-0.1041	0	Q60597	2-oxoglutarate dehydrogenase, mitochondrial	Ogdh	mmu:18293				
1773	0.3416	-0.3885	-0.1480	-0.1031	0	Q6P5E4	UDP-glucose:glycoprotein glucosyltransferase 1	Uggt1	mmu:320011				
1774	-0.3036	0.4499	-0.1057	-0.1029	0	Q9R0I7	YLP motif-containing protein 1	Ylpm1					
1775	-0.1409	0.6136	-0.0175	-0.1023	0	Q3U319	E3 ubiquitin-protein ligase BRE1B	Rnf40	mmu:233900			1	
1776	0.4698	-0.3473	-0.3612	-0.1018	0	Q9Z2G6	Protein sel-1 homolog 1	Sel1l	mmu:20338				
1777	-0.2150	-0.1568	0.0211	-0.1016	0	Q9D0R8	Protein LSM12 homolog	Lsm12	mmu:268490				
1778	-0.2822	0.0624	-0.0179	-0.1016	0	Q3UJB0		Sf3b2	mmu:319322				
1779	-1.1332	0.4705	-0.1947	-0.1007	0	Q8BVE3	V-type proton ATPase subunit H	Atp6v1h	mmu:108664				
1780	-0.6094	-0.3803	0.6743	-0.1004	0	Q6P9J9	Anoctamin-6	Ano6	mmu:105722				
1781	1.5128	-1.3591	-0.3716	-0.1000	0	Q9CPX7	28S ribosomal protein S16, mitochondrial	Mrps16	mmu:66242				
1782	0.1327	0.0533	-0.5885	-0.0999	0	E9Q555	E3 ubiquitin-protein ligase RNF213	Rnf213				1	
1783	-0.0698	0.0249	0.1299	-0.0996	0	Q9CSN1	SNW domain-containing protein 1	Snw1	mmu:66354				
1784	-0.0910	0.6509	-0.6022	-0.0987	0	Q3UEB3	Poly(U)-binding-splicing factor PUF60	Puf60	mmu:67959				
1785	-0.2286	0.0286	-0.2079	-0.0986	0	Q60715	Prolyl 4-hydroxylase subunit alpha-1	P4ha1	mmu:18451				
1786	1.1653	-0.1040	-1.0511	-0.0981	0	Q924T3	DNA repair protein XRCC4	Xrcc4	mmu:108138				
1787	0.3582	-0.5930	-0.8199	-0.0977	0	O54956	DNA polymerase epsilon subunit 2	Pole2	mmu:18974				
1788	0.1787	-0.4142	-0.3686	-0.0972	0	Q8CBY1	Protein Smaug homolog 1	Samd4a	mmu:74480				
1789	-0.0177	0.4129	0.1584	-0.0970	0	P70227	Inositol 1,4,5-trisphosphate receptor type 3	Itpr3	mmu:16440				
1790	0.2620	[illegible]	-0.2044	-0.0957	0	P08003	Protein disulfide-isomerase A4	Pdia4	mmu:12304				
1791	-0.2788	0.3056	0.2403	-0.0952	0	P52651	Homeobox protein Rhox5	Rhox5					
1792	0.5132	[illegible]	-0.3469	-0.0951	0	Q8K0D5	Elongation factor G, mitochondrial	Gfm1	mmu:28030				
1793	-1.9025	0.6255	-0.6410	-0.0949	0	P68368	Tubulin alpha-4A chain;Tubulin alpha-8 chain	Tuba4a;Tu	mmu:22145				
1794	-0.1114	0.3151	-0.2024	-0.0948	0	Q9CXK9	RNA-binding protein 33	Rbm33	mmu:381626				
1795	0.0767	-0.1263	-0.6741	-0.0947	0	Q91XU0	ATPase WRNIP1	Wrnip1	mmu:78903				
1796	0.2448	-0.1110	0.2296	-0.0944	0	Q80UZ2	Protein SDA1 homolog	Sdad1	mmu:231452				
1797	0.2200	0.0917	-0.1988	-0.0937	0	P06537	Glucocorticoid receptor	Nr3c1					
1798	-0.4507	0.2184	0.1521	-0.0937	0	Q99104	Unconventional myosin-Va	Myo5a	mmu:17918				
1799	-0.1068	0.7402	0.0207	-0.0926	0	Q9DBB4	N-alpha-acetyltransferase 15, NatA auxiliary subunit	Naa15	mmu:66897				
1800	-0.1290	0.6502	0.1509	-0.0925	0	P98083	SHC-transforming protein 1	Shc1	mmu:20416				
1801	-0.2377	-0.0762	0.6732	-0.0904	0	Q8BVQ5	Protein phosphatase methylesterase 1	Ppme1	mmu:72590				
1802	-0.5946	0.5503	-0.7779	-0.0901	0	Q8VDP6	CDP-diacylglycerol--inositol 3-phosphatidyltransferase	Cdipt	mmu:52858				
1803	0.4585	-0.2112	-0.1087	-0.0900	0	Q8BTW3	Exosome complex component MTR3	Exosc6	mmu:72544				
1804	0.5494	0.2879	-0.7896	-0.0892	0	Q01730	Ras suppressor protein 1	Rsu1					
1805	0.5362	-0.0463	-0.6865	-0.0891	0	P20664	DNA primase;DNA primase small subunit	Prim1	mmu:19075				
1806	-0.7893	1.0208	0.1772	-0.0887	0	P58501	PAX3- and PAX7-binding protein 1	Paxbp1	mmu:67367				
1807	-0.2901	0.6477	-0.6670	-0.0885	0	Q9CPQ8	ATP synthase subunit g, mitochondrial	Atp5l	mmu:27425				
1808	-0.1319	0.5048	-0.2183	-0.0883	0	Q8VDM1	Zinc finger CCCH-type with G patch domain-containing protein	Zgpat	mmu:229007				
1809	-0.4391	0.2602	-0.8418	-0.0878	0	B1AUN2		Eif2b3	mmu:108067				
1810	0.3993	0.2764	-0.1463	-0.0876	0	Q8VHM5		Hnrnpr	mmu:74326				
1811	0.3298	-0.0548	-0.4085	-0.0873	0	Q8BMS4	Ubiquinone biosynthesis O-methyltransferase, mitochondrial	Coq3	mmu:230027				
1812	0.2445	-0.7727	0.5088	-0.0864	0	P97770	THUMP domain-containing protein 3	Thumpd3	mmu:14911				
1813	-0.0801	-1.0501	0.2584	-0.0860	0	Q924H5	DNA repair protein RAD51 homolog 3	Rad51c	mmu:114714				
1814	-0.3371	0.0953	-0.2515	-0.0841	0	O35375	Neuropilin-2	Nrp2	mmu:18187				
1815	0.9416	0.5980	-0.1122	-0.0835	0	Q9CXW3	Calcyclin-binding protein	Cacybp	mmu:12301				
1816	-0.2460	0.4685	-0.0951	-0.0828	0	Q9R112	Sulfide:quinone oxidoreductase, mitochondrial	Sqrdl	mmu:59010				
1817	-0.5477	-0.0588	0.3980	-0.0827	0	P16110	Galectin;Galectin-3	Lgals3					
1818	0.1385	-0.2478	-0.4089	-0.0815	0	P52432	DNA-directed RNA polymerases I and III subunit RPAC1	Polr1c	mmu:20016				
1819	-1.0823	0.0420	-0.6650	-0.0813	0	Q5SW88		Rab1					
1820	-0.1399	0.1185	-0.1961	-0.0811	0	Q9CQZ5	NADH dehydrogenase [ubiquinone] 1 alpha subcomplex subunit 6	Ndufa6	mmu:67130	1			
1821	0.0640	-0.2617	-0.1356	-0.0810	0	O88384	Vesicle transport through interaction with t-SNAREs homolog 1B	Vti1b					
1822	-0.3336	0.6010	-0.2119	-0.0806	0	Q99LE6	ATP-binding cassette sub-family F member 2	Abcf2	mmu:27407				
1823	0.0403	-0.1965	0.2606	-0.0800	0	Q5XJE5	RNA polymerase-associated protein LEO1	Leo1	mmu:235497				
1824	0.0521	-0.0422	-0.9596	-0.0795	0	Q62018	RNA polymerase-associated protein CTR9 homolog	Ctr9	mmu:22083				
1825	-0.2686	-0.3069	0.5948	-0.0785	0	Q922Q4	Pyrroline-5-carboxylate reductase 2	Pycr2	mmu:69051	1			
1826	0.0726	0.8813	-1.4857	-0.0783	0	P83887	Tubulin gamma-1 chain;Tubulin gamma-2 chain	Tubg1;Tub	mmu:103733				
1827	-0.9790	0.4731	0.4284	-0.0775	0	Q8VEJ9	Vacuolar protein sorting-associated protein 4A	Vps4a	mmu:116733				
1828	1.2793	-0.0166	0.5661	-0.0772	0	P12970	60S ribosomal protein L7a	Rpl7a	mmu:27176				
1829	-0.3159	0.1626	0.3775	-0.0765	0	Q91YT7	YTH domain-containing family protein 2	Ythdf2	mmu:213541				
1830	-0.9734	0.2851	0.4236	-0.0761	0	Q9CQB5	CDGSH iron-sulfur domain-containing protein 2	Cisd2	mmu:67006				
1831	0.6685	[illegible]	-0.7873	-0.0756	0	P70193	Leucine-rich repeats and immunoglobulin-like domains protein 1	Lrig1	mmu:16206				
1832	-0.1132	[illegible]	-0.1074	-0.0756	0	A2AJT4	Arginine/serine-rich protein PNISR	Pnisr					
1833	0.0339	0.5161	-2.1223	-0.0755	0	D3YXK2	Scaffold attachment factor B1	Safb	mmu:224903				
1834	-1.4330	0.6972	-0.1987	-0.0753	0	Q80TZ9	Arginine-glutamic acid dipeptide repeats protein	Rere	mmu:68703				
1835	-0.0947	0.1559	-0.0612	-0.0752	0	P37913	DNA ligase 1;DNA ligase	Lig1	mmu:16881	1			
1836	0.1258	-0.1392	0.1369	-0.0751	0	Q7M739		Tpr					
1837	-0.5700	0.1006	0.9069	-0.0751	0	A2BDX3	Adenylyltransferase and sulfurtransferase MOCS3;Molybdopterin-synthase adenylyltransferase;M	Mocs3	mmu:69372				
1838	0.0231	0.4242	-0.8222	-0.0746	0	Q9D1H8	39S ribosomal protein L53, mitochondrial	Mrpl53	mmu:68499				
1839	0.3668	-0.0312	0.3557	-0.0738	0	Q00519	Xanthine dehydrogenase/oxidase;Xanthine dehydrogenase;Xanthine oxidase	Xdh	mmu:22436				
1840	0.0813	-0.3911	-0.2731	-0.0736	0	Q8BSN5	PHD finger protein 23	Phf23	mmu:78246				
1841	0.3510	-0.0770	-0.1659	-0.0736	0	Q9WV70	Nucleolar complex protein 2 homolog	Noc2l					
1842	-0.2191	0.2968	[illegible]	-0.0734	0	Q8VEK3	Heterogeneous nuclear ribonucleoprotein U	Gm28062;I	mmu:51810				
1843	-0.1898	0.3438	-0.1088	-0.0730	0	O70404	Vesicle-associated membrane protein 8	Vamp8	mmu:22320				
1844	0.1969	-0.2154	-0.6508	-0.0729	0	Q8BMF4	Dihydrolipoyllysine-residue acetyltransferase component of pyruvate dehydrogenase complex, mi	Dlat	mmu:235339				
1845	-0.3615	-0.5488	[illegible]	-0.0728	0	Q9DAM7	Transmembrane protein 263	Tmem263	mmu:103266				
1846	-0.6071	0.8569	0.1533	-0.0721	0	Q9CVB6	Actin-related protein 2/3 complex subunit 2	Arpc2	mmu:76709				
1847	0.1897	-0.7041	-0.1966	-0.0707	0	Q9CXT7	Transmembrane protein 192	Tmem192	mmu:73067				
1848	-0.4682	0.1293	-0.2823	-0.0707	0	Q9Z2X1	Heterogeneous nuclear ribonucleoprotein F;Heterogeneous nuclear ribonucleoprotein F, N-termi	Hnrnpf	mmu:98758				
1849	0.5491	-0.6524	-0.3834	-0.0705	0	Q8BHD7	Polypyrimidine tract-binding protein 3	Ptbp3	mmu:230257				
1850	-0.1061	0.9254	0.4048	-0.0703	0	Q8K3X4	Interferon regulatory factor 2-binding protein-like	Irf2bpl	mmu:238330				
1851	-0.0588	0.3249	-0.8482	-0.0702	0	Q8BSY0	Aspartyl/asparaginyl beta-hydroxylase	Asph	mmu:65973				
1852	-0.1607	-0.5353	0.3123	-0.0697	0	Q8BLU0		Flrt2	mmu:399558				
1853	0.4455	0.2638	-0.0861	-0.0697	0	Q6ZQ58	La-related protein 1	Larp1	mmu:73158				
1854	1.0216	-0.8443	-0.5969	-0.0689	0	Q9D0Q7	39S ribosomal protein L45, mitochondrial	Mrpl45	mmu:67036				
1855	0.6286	0.4705	-0.2735	-0.0686	0	Q80XC2	tRNA (adenine(58)-N(1))-methyltransferase catalytic subunit TRMT61A	Trmt61a	mmu:328162				
1856	-0.2138	[illegible]	-0.0400	-0.0684	0	G3XA17		Eif4g2					
1857	0.2135	-0.4094	0.0410	-0.0678	0	P46460	Vesicle-fusing ATPase	Nsf	mmu:18195				
1858	0.3348	-0.5666	0.3015	-0.0675	0	Q9D187	Mitotic spindle-associated MMXD complex subunit MIP18	Fam96b	mmu:68523				
1859	-0.2214	0.5157	-0.9766	-0.0671	0	P14869	60S acidic ribosomal protein P0	Rplp0	mmu:11837				
1860	0.5715	-0.3335	0.2610	-0.0670	0	Q6ZPY7	Lysine-specific demethylase 3B	Kdm3b					
1861	0.2436	-0.0443	-0.5982	-0.0665	0	Q61183	Poly(A) polymerase alpha	Papola	mmu:18789				
1862	-0.1523	0.3807	-0.0849	-0.0660	0	Q91YZ8	Polyadenylate-binding protein	Pabpc4;Gm10110					
1863	0.0626	0.3289	-1.1788	-0.0659	0	Q8K4B0	Metastasis-associated protein MTA1	Mta1					
1864	0.0938	-0.3682	0.4392	-0.0659	0	P12382	ATP-dependent 6-phosphofructokinase, liver type	Pfkl	mmu:18641				
1865	-0.6345	-1.1725	0.1579	-0.0657	0	Q9D0U6	Repressor of RNA polymerase III transcription MAF1;Repressor of RNA polymerase III transcriptior	Maf1	mmu:68877				
1866	0.6824	-0.1838	-0.3645	-0.0654	0	Q9CWZ3	RNA-binding protein 8A	RBM8;Rbm	mmu:60365				
1867	0.5786	-0.2434	0.8025	-0.0653	0	Q9CXW4	60S ribosomal protein L11	Rpl11;Gm1	mmu:67025				
1868	-0.0754	0.1797	0.1776	-0.0652	0	Q9JKB3	Y-box-binding protein 3	Ybx3	mmu:56449				
1869	-0.5905	0.3138	0.3070	-0.0648	0	O35737	Heterogeneous nuclear ribonucleoprotein H;Heterogeneous nuclear ribonucleoprotein H, N-termi	Hnrnph1	mmu:59013				
1870	0.1095	0.2130	-0.3724	-0.0648	0	Q6P5B0	RRP12-like protein	Rrp12	mmu:107094				
1871	0.1642	-0.6183	0.3329	-0.0644	0	P70452	Syntaxin-4	Stx4	mmu:20909	1			
1872	-0.0174	-0.1369	0.2500	-0.0641	0	Q9Z1Q9	Valine--tRNA ligase	Vars	mmu:22321				
1873	-1.1333	0.2818	0.3597	-0.0641	0	Q8CGU1	Calcium-binding and coiled-coil domain-containing protein 1	Calcoco1	mmu:67488				
1874	-0.1465	0.3865	0.2750	-0.0638	0	Q9R045	Angiopoietin-related protein 2	Angptl2	mmu:26360				
1875	0.1707	-0.0963	-0.4071	-0.0637	0	P56480	ATP synthase subunit beta, mitochondrial	Atp5b	mmu:11947				
1876	-0.2275	0.3204	0.2359	-0.0631	0	Q8BYK6	YTH domain-containing family protein 3	Ythdf3	mmu:229096				
1877	-0.0096	0.0226	-0.4517	-0.0629	0	Q9CZX0	Elongator complex protein 3	Elp3	mmu:74195				
1878	0.0399	-0.3954	-0.0863	-0.0622	0	Q6ZWX6	Eukaryotic translation initiation factor 2 subunit 1	Eif2s1	mmu:13665				
1879	-0.0292	-0.2111	0.1069	-0.0616	0	P97496	SWI/SNF complex subunit SMARCC1	Smarcc1	mmu:20588				

1880	0.0624	-0.5103	-0.1275	-0.0614	0	Q8R081	Heterogeneous nuclear ribonucleoprotein L	Hnrnpl	mmu:15388				
1881	-0.0840	0.6084	0.0995	-0.0613	0	O35134	DNA-directed RNA polymerase I subunit RPA1	Polr1a	mmu:20019				
1882	0.3061	-0.2914	-0.0317	-0.0610	0	Q9R1T2	SUMO-activating enzyme subunit 1;SUMO-activating enzyme subunit 1, N-terminally processed	Sae1	mmu:56459				
1883	0.4595	-0.1923	[illegible]	-0.0610	0	P35282	Ras-related protein Rab-21	Rab21	mmu:216344				
1884	-0.6814	0.2452	0.8066	-0.0602	0	Q9D1F4	Proline-rich AKT1 substrate 1	Akt1s1	mmu:67605				
1885	0.7586	-0.9533	0.0824	-0.0602	0	Q8K1X1	WD repeat-containing protein 11	Wdr11	mmu:207425				
1886	0.3253	-0.2897	-0.1221	-0.0601	0	P46061	Ran GTPase-activating protein 1	Rangap1	mmu:19387				
1887	0.1045	-0.0774	0.0945	-0.0599	0	Q9JIK9	28S ribosomal protein S34, mitochondrial	Mrps34	mmu:79044				
1888	-0.5530	-0.3727	0.0795	-0.0599	0	Q9CZ28	Vacuolar-sorting protein SNF8	Snf8	mmu:27681				
1889	0.1055	-0.2014	0.1178	-0.0594	0	Q922J9	Fatty acyl-CoA reductase 1	Far1	mmu:67420				
1890	-0.3699	-0.0732	[illegible]	-0.0587	0	Q8C1D8	Protein IWS1 homolog	Iws1	mmu:73473				
1891	0.1121	0.4532	-0.2038	-0.0562	0	Q8BMQ2	General transcription factor 3C polypeptide 4	Gtf3c4	mmu:269252				
1892	0.4532	-0.7360	-0.7355	-0.0560	0	Q924Z4	Ceramide synthase 2	Cers2	mmu:76893				
1893	0.2538	-0.4126	-0.5671	-0.0556	0	P60766	Cell division control protein 42 homolog	Cdc42	mmu:12540				
1894	0.0426	[illegible]	-0.3596	-0.0556	0	Q8BIJ7	RUN and FYVE domain-containing protein 1	Rufy1	mmu:216724				
1895	1.3491	-0.1072	0.4051	-0.0553	0	P15864	Histone H1,2	Hist1h1c	mmu:50708				
1896	-0.3741	-0.1507	0.4810	-0.0541	0	Q9ESN6	Tripartite motif-containing protein 2	Trim2	mmu:80890			1	
1897	-0.1081	-0.5664	0.4870	-0.0540	0	Q8K3C3	Protein LZIC	Lzic	mmu:69151				
1898	1.0156	0.5569	-0.1619	-0.0504	0	Q8BVY0	Ribosomal L1 domain-containing protein 1	Rsl1d1	mmu:66409				
1899	0.0726	0.0979	-0.1995	-0.0495	0	Q62261	Spectrin beta chain, non-erythrocytic 1	Sptbn1	mmu:20742				
1900	1.6561	-1.1349	-1.1734	-0.0484	0	Q8CG46	Structural maintenance of chromosomes protein 5	Smc5	mmu:226026				
1901	-0.2812	0.3310	[illegible]	-0.0484	0	Q9Z315	U4/U6,U5 tri-snRNP-associated protein 1	Sart1	mmu:20227				1
1902	-0.3213	1.0839	0.4345	-0.0482	0	O70274	Protein tyrosine phosphatase type IVA 2	Ptp4a2	mmu:19244		1		
1903	-0.0661	0.2861	0.3951	-0.0481	0	O08807	Peroxiredoxin-4	Prdx4	mmu:53381	1			
1904	-0.0777	-0.0257	0.0447	-0.0479	0	O88685	26S protease regulatory subunit 6A	Psmc3	mmu:19182				
1905	-0.0590	0.0648	-0.1138	-0.0475	0	Q8BH74	Nuclear pore complex protein Nup107	Nup107	mmu:103468				
1906	-0.1309	0.5344	0.2325	-0.0467	0	Q6P542	ATP-binding cassette sub-family F member 1	Abcf1	mmu:224742				
1907	-0.7540	0.1094	-0.4455	-0.0454	0	P35278	Ras-related protein Rab-5C	Rab5c	mmu:19345				
1908	[illegible]	-0.4911	0.1999	-0.0448	0	F8VQC1	Signal recognition particle subunit SRP72	Srp72	mmu:66661				
1909	-0.3319	0.0528	[illegible]	-0.0447	0	Q99KD5	Protein unc-45 homolog A	Unc45a	mmu:101869				
1910	-0.0052	0.5771	-0.3906	-0.0441	0	P61290	Proteasome activator complex subunit 3	Psme3	mmu:19192				
1911	-0.3315	-0.2989	0.2721	-0.0434	0	Q3UA37	Glutamine-rich protein 1	Qrich1	mmu:69232				
1912	0.4851	-0.4585	0.2921	-0.0433	0	Q8BKI2	Trinucleotide repeat-containing gene 6B protein	Tnrc6b	mmu:213988				
1913	0.2761	1.1737	-0.3780	-0.0432	0	P26645	Myristoylated alanine-rich C-kinase substrate	Marcks	mmu:17118				
1914	0.2675	0.2507	-0.0593	-0.0419	0	Q9JIK5	Nucleolar RNA helicase 2	Ddx21	mmu:56200				
1915	0.1786	-0.1020	0.1643	-0.0415	0	Q6PFD9	Nuclear pore complex protein Nup98-Nup96;Nuclear pore complex protein Nup98;Nuclear pore c	Nup98	mmu:269966				
1916	-0.4939	0.0398	0.1856	-0.0414	0	Q3TWW8	Serine/arginine-rich splicing factor 6	Srsf6	mmu:67996				
1917	0.0649	-0.2054	-0.1373	-0.0409	0	Q8VEH8	Endoplasmic reticulum lectin 1	Erlec1	mmu:66753				
1918	-0.6616	1.2959	0.7682	-0.0401	0	P07356	Annexin A2;Annexin	Anxa2	mmu:12306				
1919	-0.2788	0.2761	0.3109	-0.0400	0	D3Z7P3	Glutaminase kidney isoform, mitochondrial	Gls	mmu:14660				
1920	-0.3258	-0.7805	0.2842	-0.0396	0	Q62393	Tumor protein D52	Tpd52	mmu:21985				
1921	-0.1132	0.2652	0.8386	-0.0389	0	Q5DTM8	E3 ubiquitin-protein ligase BRE1A	Rnf20	mmu:109331			1	
1922	0.1320	-0.3689	-0.1810	-0.0385	0	Q8VI36	Paxillin	Pxn	mmu:19303				
1923	0.3190	-0.5461	-0.0395	-0.0382	0	Q3UMR0	Ankyrin repeat domain-containing protein 27	Ankrd27	mmu:245886				
1924	-0.1235	0.3299	0.4927	-0.0377	0	Q3URD3	Sarcolemmal membrane-associated protein	Slmap	mmu:83997				
1925	0.2129	0.0601	-0.2855	-0.0377	0	Q9D051	Pyruvate dehydrogenase E1 component subunit beta, mitochondrial	Pdhb	mmu:68263				
1926	-0.8100	0.2404	-0.0073	-0.0376	0	Q8BKF1	DNA-directed RNA polymerase;DNA-directed RNA polymerase, mitochondrial	Polrmt	mmu:216151				
1927	-0.0843	0.3131	-0.3692	-0.0363	0	Q9WVA3	Mitotic checkpoint protein BUB3	Bub3	mmu:12237				
1928	-0.3865	0.3469	-0.3822	-0.0357	0	Q9DCU6	39S ribosomal protein L4, mitochondrial	Mrpl4	mmu:66163				
1929	0.3033	0.3067	-0.0686	-0.0356	0	Q80ZX0		Sec24b	mmu:99683				
1930	-1.6569	1.4150	0.1529	-0.0355	0	Q9WTP7	GTP:AMP phosphotransferase AK3, mitochondrial	Ak3	mmu:56248				
1931	0.5000	0.1940	-0.0588	-0.0354	0	P50136	2-oxoisovalerate dehydrogenase subunit alpha, mitochondrial	Bckdha					
1932	-0.3440	-0.4495	0.0427	-0.0353	0	Q3U3W5	Putative protein arginine N-methyltransferase 9	Prmt10;Pr	mmu:102182				
1933	0.2379	-0.4111	0.6866	-0.0352	0	Q60759	Glutaryl-CoA dehydrogenase, mitochondrial	Gcdh	mmu:270076				
1934	0.3113	0.0280	-0.2273	-0.0352	0	Q8VC30	Bifunctional ATP-dependent dihydroxyacetone kinase/FAD-AMP lyase (cyclizing);ATP-dependent c	Dak	mmu:225913				
1935	0.4833	-0.6439	-0.7648	-0.0351	0	Q9D1P0	39S ribosomal protein L13, mitochondrial	Mrpl13	mmu:68537				
1936	-0.0315	-0.1454	0.2088	-0.0349	0	Q61187	Tumor susceptibility gene 101 protein	Tsg101	mmu:22088				
1937	0.5898	-0.2591	-0.4060	-0.0332	0	P17225	Polypyrimidine tract-binding protein 1	Ptbp1					
1938	0.4039	-0.5957	0.1511	-0.0326	0	Q8JZX4	Splicing factor 45	Rbm17	mmu:76938				
1939	-0.4519	[illegible]	0.3609	-0.0324	0	Q9CSV6	Vesicle transport protein SFT2C	Sft2d3					
1940	-0.0446	0.0545	0.4579	-0.0319	0	Q3TW96	UDP-N-acetylhexosamine pyrophosphorylase-like protein 1	Uap1l1	mmu:227620				
1941	-0.1346	0.1258	-0.4713	-0.0318	0	P01902	H-2 class I histocompatibility antigen, K-D alpha chain	H2-K1					
1942	-0.3959	0.0305	0.1776	-0.0312	0	Q9Z2G0	Protein fem-1 homolog B	Fem1b	mmu:14155			1	1
1943	-0.1635	-0.0096	0.3222	-0.0309	0	Q8K019	Bcl-2-associated transcription factor 1	Bclaf1	mmu:72567				1
1944	0.9803	-0.7056	0.0793	-0.0308	0	P28033	CCAAT/enhancer-binding protein beta	Cebpb	mmu:12608				1
1945	-0.1206	0.1711	-0.4703	-0.0301	0	Q8R3F9	Speckle targeted PIP5K1A-regulated poly(A) polymerase	Tut1	mmu:70044				
1946	-0.0409	0.1830	[illegible]	-0.0299	0	P25322	G1/S-specific cyclin-D1	Ccnd1	mmu:12443				
1947	0.0844	-0.5414	-0.1062	-0.0292	0	Q99LI8	Hepatocyte growth factor-regulated tyrosine kinase substrate	Hgs	mmu:15239				
1948	-1.1265	1.1123	0.7518	-0.0286	0	P22315	Ferrochelatase;Ferrochelatase, mitochondrial	Fech					
1949	-0.6202	0.2205	0.0295	-0.0278	0	P12815	Programmed cell death protein 6	Pdcd6	mmu:18570				1
1950	0.5725	0.1018	-0.6370	-0.0277	0	Q9CPX6	Ubiquitin-like-conjugating enzyme ATG3	Atg3	mmu:67841				
1951	0.1636	0.0642	-0.0948	-0.0269	0	Q99N96	39S ribosomal protein L1, mitochondrial	Mrpl1	mmu:94061				
1952	-0.0550	-0.1566	0.3264	-0.0259	0	Q6P9Q4	FH1/FH2 domain-containing protein 1	Fhod1	mmu:234686				
1953	-0.5173	0.5758	0.2539	-0.0257	0	Q08297	DNA repair protein RAD51 homolog 1	Rad51	mmu:19361				
1954	0.2281	-0.2572	0.3257	-0.0246	0	Q9ERI5	Bifunctional arginine demethylase and lysyl-hydroxylase JMJD6	Jmjd6	mmu:107817				
1955	0.9296	0.5159	-1.4474	-0.0245	0	Q9Z248	Zinc finger protein AEBP2	Aebp2	mmu:11569				
1956	-0.7358	-0.1337	0.2068	-0.0235	0	Q8VCH8	UBX domain-containing protein 4	Ubxn4	mmu:67812				
1957	0.1376	-0.0483	-0.2109	-0.0235	0	Q91VU7		Pus7					
1958	-1.0583	-0.1094	0.8736	-0.0234	0	Q9CR41	Huntingtin-interacting protein K	Hypk	mmu:67693				
1959	0.1472	0.3337	-0.0936	-0.0223	0	Q8CG72	Poly(ADP-ribose) glycohydrolase ARH3	Adprhl2	mmu:100206	1			
1960	-0.4200	-0.0232	0.1900	-0.0221	0	P62748	Hippocalcin-like protein 1	Hpcal1	mmu:53602				
1961	0.3186	0.5857	-0.2393	-0.0220	0	Q8BXA1	Golgi integral membrane protein 4	Golim4	mmu:73124				
1962	-0.1148	-0.4599	0.2638	-0.0217	0	Q91VS8	FERM, RhoGEF and pleckstrin domain-containing protein 2	Farp2	mmu:227377				
1963	0.6255	-0.4386	-0.4948	-0.0212	0	Q8R502	Volume-regulated anion channel subunit LRRC8C	Lrrc8c	mmu:100604				
1964	0.2411	-0.7511	-1.1542	-0.0210	0	Q99L02	PAXIP1-associated glutamate-rich protein 1A	Pagr1a;Pag	mmu:67278				
1965	-0.4405	0.1011	0.6494	-0.0210	0	Q91WJ8	Far upstream element-binding protein 1	Fubp1					
1966	-0.4373	0.6171	-0.1348	-0.0195	0	E9PZJ8	Activating signal cointegrator 1 complex subunit 3	Ascc3	mmu:77987				
1967	-0.1901	0.0182	-0.1324	-0.0195	0	P26231	Catenin alpha-1	Ctnna1	mmu:12385				
1968	1.5155	0.2075	-0.0647	-0.0192	0	Q9JHI5	Isovaleryl-CoA dehydrogenase, mitochondrial	Ivd	mmu:56357				
1969	-0.5122	-0.1528	0.0905	-0.0191	0	E9PYB0		Ahnak2					
1970	0.4922	0.3691	-0.4321	-0.0187	0	Q91WG4	Elongator complex protein 2	Elp2	mmu:58523				
1971	-0.1886	0.1886	0.0239	-0.0183	0	Q91V41	Ras-related protein Rab-14	Rab14	mmu:68365				
1972	0.1571	-0.2566	-0.4183	-0.0180	0	P58802	TBC1 domain family member 10A	Tbc1d10a	mmu:103724				
1973	0.2511	-0.0292	-0.0661	-0.0178	0	P62814	V-type proton ATPase subunit B, brain isoform	Atp6v1b2	mmu:11966				
1974	0.4059	-0.2631	-0.1973	-0.0173	0	Q922R8	Protein disulfide-isomerase A6	Pdia6	mmu:71853				
1975	-0.1576	0.3902	0.3219	-0.0169	0	Q9JIX8	Apoptotic chromatin condensation inducer in the nucleus	Acin1	mmu:56215				
1976	-0.5999	0.3724	-0.2260	-0.0166	0	Q91YM4	Protein TBRG4	Tbrg4	mmu:21379				
1977	0.2562	-0.1652	0.7331	-0.0164	0	Q8R5A3	Amyloid beta A4 precursor protein-binding family B member 1-interacting protein	Apbb1ip	mmu:54519				
1978	0.4371	-0.9382	-0.4993	-0.0162	0	Q91YJ5	Translation initiation factor IF-2, mitochondrial	Mtif2	mmu:76784				
1979	0.0290	0.0080	-0.3485	-0.0161	0	Q6P5H2	Nestin	Nes	mmu:18008				
1980	-2.1795	0.6514	-0.2471	-0.0161	0	P62320	Small nuclear ribonucleoprotein Sm D3	Snrpd3	mmu:67332				
1981	-0.0551	-0.3773	0.1205	-0.0160	0	P51655	Glypican-4;Secreted glypican-4	Gpc4	mmu:14735				
1982	-0.2420	0.5397	0.5871	-0.0157	0	P62852	40S ribosomal protein S25	Rps25	mmu:75617				
1983	0.2073	-0.4764	0.1350	-0.0148	0	Q9DB20	ATP synthase subunit O, mitochondrial	Atp5o	mmu:28080				
1984	-0.5294	0.5751	0.3457	-0.0148	0	Q9D7S7	60S ribosomal protein L22-like 1	Rpl22l1	mmu:68028				
1985	-1.0914	0.6755	-0.0190	-0.0134	0	P46718	Programmed cell death protein 2	Pdcd2	mmu:18567				
1986	-0.6368	0.0382	0.6757	-0.0134	0	Q922V4	Pleiotropic regulator 1	Plrg1	mmu:53317				
1987	0.1623	0.0876	-0.1615	-0.0132	0	Q8BR65	Sin3 histone deacetylase corepressor complex component SDS3	Suds3	mmu:71954				
1988	0.4592	-0.1749	-0.0928	-0.0129	0	Q8JZM7	Parafibromin	Cdc73	mmu:214498				
1989	0.3101	-0.0842	-0.2039	-0.0121	0	Q6P2K6	Serine/threonine-protein phosphatase 4 regulatory subunit 3A	Smek1	mmu:68734				
1990	-0.8970	0.1781	0.1006	-0.0120	0	Q03958	Prefoldin subunit 6	Pfdn6	mmu:14976				
1991	0.0108	0.2579	-0.2414	-0.0115	0	P62192	26S protease regulatory subunit 4	Psmc1	mmu:19179				
1992	0.2473	-0.0206	0.0807	-0.0110	0	Q8C7R4	Ubiquitin-like modifier-activating enzyme 6	Uba6	mmu:231380			1	
1993	-0.3633	-0.7938	0.8271	-0.0109	0	O35551	Rab GTPase-binding effector protein 1	Rabep1	mmu:54189				
1994	-0.0744	0.0703	-0.0575	-0.0106	0	Q62432	Mothers against decapentaplegic homolog;Mothers against decapentaplegic homolog 2	Smad2	mmu:17126				
1995	-0.5048	-0.1741	0.2878	-0.0105	0	P62830	60S ribosomal protein L23	Rpl23	mmu:65019				
1996	0.1471	-0.0748	0.7316	-0.0103	0	Q9QY66	Zinc finger HIT domain-containing protein 2	Znhit2	mmu:29805				
1997	-1.2614	0.1881	0.5437	-0.0103	0	Q4VAE3	Transmembrane protein 65	Tmem65	mmu:74868				
1998	-0.4325	0.0859	0.0129	-0.0100	0	Q8K2X3	CST complex subunit STN1	Obfc1	mmu:108689				
1999	-0.3547	0.3323	-0.0359	-0.0099	0	Q9R0U0	Serine/arginine-rich splicing factor 10	Srsf10	mmu:14105				
2000	-0.3092	0.3255	0.0749	-0.0095	0	P27612	Phospholipase A-2-activating protein	Plaa	mmu:18786				
2001	0.0678	-0.2132	-0.1542	-0.0093	0	Q921I2	Kelch domain-containing protein 4	Klhdc4					
2002	0.2297	0.0152	[illegible]	-0.0093	0	Q91V92	ATP-citrate synthase	Acly	mmu:104112				
2003	[illegible]	-0.0351	0.2084	-0.0087	0	Q80X90	Filamin-B	Flnb	mmu:286940				
2004	0.1081	-0.1900	-0.3109	-0.0078	0	Q8C8U0	Liprin-beta-1	Ppfibp1	mmu:67533				
2005	0.0170	-0.0951	0.4613	-0.0071	0	E9Q394		Akap13	mmu:75547				

2006	-0.0172	0.1954	-1.0182	-0.0062	0	P62880	Guanine nucleotide-binding protein G(I)/G(S)/G(T) subunit beta-2;Guanine nucleotide-binding pro	Gnb2;Gnb	mmu:14693			
2007	-0.0637	-0.0783	0.5979	-0.0054	0	Q811D0	Disks large homolog 1	Dlg1	mmu:13383			
2008	0.0565	-0.1279	-0.3060	-0.0052	0	Q6A4J8	Ubiquitin carboxyl-terminal hydrolase;Ubiquitin carboxyl-terminal hydrolase 7	Usp7	mmu:252870			
2009	-0.5768	0.3777	0.2350	-0.0051	0	A2AJ15	Endoplasmic reticulum mannosyl-oligosaccharide 1,2-alpha-mannosidase	Man1b1	mmu:227619			
2010	-0.2189	0.2493	-0.2711	-0.0048	0	Q8BH64	EH domain-containing protein 2	Ehd2	mmu:259300			
2011	0.1605	-0.4162	-0.3616	-0.0043	0	Q9DBG5	Perilipin-3	Plin3	mmu:66905			
2012	-0.1438	-0.1359	0.1721	-0.0019	0	Q9JLV5	Cullin-3	Cul3	mmu:26554		1	1
2013	0.1366	-0.3637	0.2558	-0.0016	0	Q8VDU3		Apol9a	mmu:223672			
2014	0.1401	0.2260	-0.4086	[illegible]	0	P43247	DNA mismatch repair protein Msh2	Msh2	mmu:17685			
2015	-0.1834	0.0568	0.6874	[illegible]	0	P50580	Proliferation-associated protein 2G4	Pa2g4	mmu:18813			
2016	-0.1082	0.2068	0.6864	[illegible]	0	P10126	Elongation factor 1-alpha 1	Eef1a1	mmu:13627			
2017	0.0917	0.0388	-0.0686	[illegible]	0	Q99KK7	Dipeptidyl peptidase 3	Dpp3	mmu:75221			
2018	0.3039	-0.5690	-0.3516	[illegible]	0	Q07113	Cation-independent mannose-6-phosphate receptor	Igf2r	mmu:16004			
2019	0.5478	-0.5595	0.3491	[illegible]	0	O70481	E3 ubiquitin-protein ligase UBR1	Ubr1	mmu:22222		1	
2020	0.4566	-0.3992	-1.1861	[illegible]	0	Q9JJ11	Transforming acidic coiled-coil-containing protein 3	Tacc3				
2021	-0.0600	0.1460	-0.3440	[illegible]	0	Q9R1X4	Protein timeless homolog	Timeless	mmu:21853			
2022	-0.1943	-0.2488	0.0987	[illegible]	0	Q02248	Catenin beta-1	Ctnnb1	mmu:12387			
2023	-0.6006	0.4553	-0.4109	[illegible]	0	Q60767	Lymphocyte antigen 75	Ly75	mmu:17076			
2024	1.1817	0.8137	-0.5884	[illegible]	0	F2Z3U3		Raph1	mmu:77300			
2025	-0.0395	0.0774	-0.7100	[illegible]	0	P59017	Bcl-2-like protein 13	Bcl2l13	mmu:94044			1
2026	-0.1127	0.0229	-0.0939	0.0033	0	P52479	Ubiquitin carboxyl-terminal hydrolase 10	Usp10	mmu:22224			
2027	-0.0234	-0.0479	0.1651	[illegible]	0	P80314	T-complex protein 1 subunit beta	Cct2	mmu:12461			
2028	[illegible]	0.2443	0.2689	0.0038	0	Q62523	Zyxin	Zyx	mmu:22793			
2029	0.1436	0.1244	-0.4355	0.0044	0	Q3UJB9	Enhancer of mRNA-decapping protein 4	Edc4	mmu:234699			
2030	-0.2191	0.5814	-1.2400	0.0045	0	O35638	Cohesin subunit SA-2	Stag2	mmu:20843			
2031	-0.5154	0.2121	-0.8615	0.0046	0	Q8R2Y8	Peptidyl-tRNA hydrolase 2, mitochondrial	Ptrh2	mmu:217057			
2032	0.1764	0.2502	-0.2106	0.0048	0	Q9CQV6	Microtubule-associated proteins 1A/1B light chain 3B;Microtubule-associated proteins 1A/1B light	Map1lc3b;	mmu:67443			
2033	-1.0506	0.2447	0.0680	0.0048	0	Q99020	Heterogeneous nuclear ribonucleoprotein A/B	Hnrnpab	mmu:15384			
2034	0.4847	0.1979	-0.1074	0.0053	0	Q3UMQ8	H/ACA ribonucleoprotein complex non-core subunit NAF1	Naf1	mmu:234344			
2035	0.1275	-0.1701	0.4459	0.0055	0	Q8CBW3	Abl interactor 1	Abi1	mmu:11308			
2036	-0.4779	0.3271	[illegible]	0.0056	0	Q8CG48	Structural maintenance of chromosomes protein 2	Smc2	mmu:14211			
2037	-0.5053	0.1370	0.0874	0.0056	0	P61979	Heterogeneous nuclear ribonucleoprotein K	Hnrnpk	mmu:15387			
2038	0.0845	0.0842	-0.1323	0.0058	0	Q8BTI8	Serine/arginine repetitive matrix protein 2	Srrm2	mmu:75956			
2039	-0.2311	-0.0675	0.6184	0.0063	0	Q78PY7	Staphylococcal nuclease domain-containing protein 1	Snd1	mmu:56463			
2040	-0.5059	0.6923	1.1701	0.0067	0	Q8VCQ8		Cald1	mmu:109624			
2041	-1.5806	0.0686	0.1431	0.0067	0	Q62165	Dystroglycan;Alpha-dystroglycan;Beta-dystroglycan	Dag1	mmu:13138			
2042	0.0094	-0.1228	-0.0349	0.0070	0	Q9JLI8	Squamous cell carcinoma antigen recognized by T-cells 3	Sart3	mmu:53890			
2043	0.8099	-0.0553	-0.0088	0.0074	0	Q9WV55	Vesicle-associated membrane protein-associated protein A	Vapa	mmu:30960			
2044	0.6281	-0.3360	0.5740	0.0075	0	Q9CQA3	Succinate dehydrogenase [ubiquinone] iron-sulfur subunit, mitochondrial	Sdhb	mmu:67680			
2045	0.0401	0.6315	-0.4653	0.0079	0	Q8K273	Membrane magnesium transporter 1	Mmgt1	mmu:236792			
2046	0.0343	0.4429	-0.8860	0.0079	0	Q8BHG1	Nardilysin	Nrd1	mmu:230598			
2047	-0.6037	0.1879	0.3170	0.0080	0	Q9Z127	Large neutral amino acids transporter small subunit 1	Slc7a5	mmu:20539			
2048	0.1192	0.0947	-0.0192	0.0090	0	Q9DCD2	Pre-mRNA-splicing factor SYF1	Xab2	mmu:67439			
2049	-0.3106	-0.3219	0.1968	0.0096	0	P35951	Low-density lipoprotein receptor	Ldlr	mmu:16835			
2050	-0.1033	-0.0534	0.1046	0.0102	0	Q68FF6	ARF GTPase-activating protein GIT1	Git1	mmu:216963			
2051	-0.2751	0.1183	0.7027	0.0103	0	Q9R0P4	Small acidic protein	Smap;111	mmu:56372			
2052	0.2363	-0.0430	-0.3057	0.0104	0	Q9D1H7	Golgi to ER traffic protein 4 homolog	Get4	mmu:67604			
2053	-0.0098	-0.0928	0.0544	0.0105	0	P58404	Striatin-4	Strn4	mmu:97387			
2054	0.0079	-0.1037	-0.4354	0.0106	0	G5E829	Plasma membrane calcium-transporting ATPase 1	Atp2b1	mmu:67972			
2055	0.3969	-0.4084	0.0896	0.0106	0	Q8CIE4		Parp10	mmu:671535			
2056	0.0113	-0.0580	-0.5770	0.0126	0	P19783	Cytochrome c oxidase subunit 4 isoform 1, mitochondrial	Cox4i1	mmu:12857			
2057	0.2833	-0.0626	-0.4024	0.0126	0	P25206	DNA replication licensing factor MCM3	Mcm3	mmu:17215			
2058	0.1284	-0.5012	-0.4077	0.0130	0	P27641	X-ray repair cross-complementing protein 5	Xrcc5	mmu:22596			
2059	-0.3393	-0.1770	0.2418	0.0131	0	Q9QUJ7	Long-chain-fatty-acid--CoA ligase 4	Acsl4	mmu:50790			
2060	-0.2852	0.3416	-0.2837	0.0132	0	Q9Z1B5	Mitotic spindle assembly checkpoint protein MAD2A	Mad2l1	mmu:56150			
2061	-0.0396	0.1435	0.2522	0.0146	0	Q569Z6	Thyroid hormone receptor-associated protein 3	Thrap3	mmu:230753			
2062	-0.2227	-0.3520	0.1128	0.0150	0	Q99LN9	Deoxyhypusine hydroxylase	Dohh	mmu:102115			
2063	-0.4740	-0.6913	0.7033	0.0154	0	Q8R010	Aminoacyl tRNA synthase complex-interacting multifunctional protein 2	Aimp2	mmu:231872			
2064	-0.3498	0.7191	-0.3747	0.0158	0	P26043	Radixin	Rdx	mmu:19684			
2065	[illegible]	0.1565	-0.5061	0.0160	0	Q9D753	Exosome complex component RRP43	Exosc8	mmu:69639			
2066	-0.2419	-0.1405	0.0844	0.0167	0	Q76MZ3	Serine/threonine-protein phosphatase 2A 65 kDa regulatory subunit A alpha isoform	Ppp2r1a	mmu:51792			1
2067	0.2520	-0.1033	-1.4852	0.0170	0	Q61164	Transcriptional repressor CTCF	Ctcf	mmu:13018			
2068	0.0354	-0.0101	0.3612	0.0183	0	O08739	AMP deaminase 3	Ampd3	mmu:11717			
2069	0.0368	-0.2383	0.0421	0.0184	0	Q9D842	Aprataxin and PNK-like factor	Aplf	mmu:72103			
2070	-0.1909	0.2862	-0.0339	0.0187	0	E9Q616		Ahnak				
2071	0.0575	-0.2266	-0.3568	0.0190	0	O55143	Sarcoplasmic/endoplasmic reticulum calcium ATPase 2	Atp2a2	mmu:11938	1		
2072	-0.3247	0.2876	0.4143	0.0198	0	Q9CPW4	Actin-related protein 2/3 complex subunit 5	Arpc5	mmu:67771			
2073	0.1713	0.0666	-0.1469	0.0199	0	A2RS43		Pcdh7				
2074	-0.2969	0.6443	0.3815	0.0201	0	P05064	Fructose-bisphosphate aldolase A;Fructose-bisphosphate aldolase	Aldoa;Aldc	mmu:11674			
2075	-0.1386	0.5128	0.1555	0.0201	0	Q9CZU6	Citrate synthase, mitochondrial	Cs	mmu:12974			
2076	-0.1273	0.2337	-0.3265	0.0202	0	Q9QZ06	Toll-interacting protein	Tollip	mmu:54473			
2077	1.1243	0.8880	-0.8952	0.0203	0	Q6ZQ03	Formin-binding protein 4	Fnbp4	mmu:55935			
2078	0.6324	-0.9252	0.3188	0.0204	0	Q91YD3	mRNA-decapping enzyme 1A	Dcp1a	mmu:75901			
2079	-0.3215	[illegible]	-0.9065	0.0205	0	P47740	Aldehyde dehydrogenase;Fatty aldehyde dehydrogenase	Aldh3a2	mmu:11671			
2080	-0.5661	0.7256	-0.1206	0.0209	0	Q8VH51	RNA-binding protein 39	Rbm39	mmu:170791			
2081	0.0716	-0.0325	-0.0994	0.0213	0	Q8BP47	Asparagine--tRNA ligase, cytoplasmic	Nars	mmu:70223			
2082	0.1505	0.1636	-0.0265	0.0215	0	Q9D554	Splicing factor 3A subunit 3	Sf3a3	mmu:75062			
2083	-0.4436	-0.0407	0.3571	0.0229	0	Q9Z2U0	Proteasome subunit alpha type-7;Proteasome subunit alpha type-7-like	Psma7;Psn	mmu:26444			
2084	-1.0410	0.6924	-0.5346	0.0230	0	Q8BYH7	TBC1 domain family member 17	Tbc1d17	mmu:233204			
2085	0.5788	0.2085	-0.2046	0.0230	0	Q61127	NGFI-A-binding protein 2	Nab2	mmu:17937			
2086	-0.7237	0.6248	-0.1199	0.0231	0	Q3TMP1		Gtf3c3	mmu:98488			
2087	0.6363	-0.2878	-0.2518	0.0233	0	Q6NV83	U2 snRNP-associated SURP motif-containing protein	U2surp	mmu:67958			
2088	0.3009	-0.5657	0.2570	0.0235	0	Q9QZF2	Glypican-1;Secreted glypican-1	Gpc1	mmu:14733			
2089	-0.0473	0.1487	0.3570	0.0237	0	Q8K268	ATP-binding cassette sub-family F member 3	Abcf3	mmu:27406			
2090	0.1136	-0.2243	0.0077	0.0238	0	Q8BUV3	Gephyrin;Molybdopterin adenylyltransferase;Molybdopterin molybdenumtransferase	Gphn	mmu:268566			
2091	-0.2438	1.0990	-0.7792	0.0238	0	Q9CQF0	39S ribosomal protein L11, mitochondrial	Mrpl11	mmu:66419			
2092	-0.3036	0.2907	-0.2750	0.0240	0	Q99KP6	Pre-mRNA-processing factor 19	Prpf19	mmu:28000		1	
2093	0.0405	-0.2058	-0.2975	0.0241	0	P97390	Vacuolar protein sorting-associated protein 45	Vps45	mmu:22365			
2094	0.5414	-0.1240	-0.0676	0.0244	0	Q9Z2H5	Band 4.1-like protein 1	Epb41l1;Ep	mmu:13821			
2095	-0.0509	0.1643	0.5088	0.0250	0	Q9CXX9	CUE domain-containing protein 2	Cuedc2	mmu:67116			
2096	0.2938	-0.9655	0.2995	0.0250	0	Q8K2M0	39S ribosomal protein L38, mitochondrial	Mrpl38	mmu:60441			
2097	0.3790	-0.1165	-0.2086	0.0258	0	Q9EQK5	Major vault protein	Mvp				
2098	-0.1845	0.1290	-0.0983	0.0261	0	Q8C0L0	Thioredoxin-related transmembrane protein 4	Tmx4	mmu:52837			
2099	-0.4889	-0.1204	[illegible]	0.0266	0	Q3TZZ7	Extended synaptotagmin-2	Esyt2	mmu:52635			
2100	0.0806	0.0856	-0.1976	0.0266	0	Q99MR6	Serrate RNA effector molecule homolog	Srrt	mmu:83701			
2101	-0.1527	0.2775	0.6244	0.0268	0	Q9EPJ9	ADP-ribosylation factor GTPase-activating protein 1	Arfgap1	mmu:228998			
2102	0.0484	-0.0056	-0.0509	0.0268	0	F7DBB3		Ahnak2				
2103	0.1220	-0.5066	-0.6771	0.0273	0	Q3UMB9	WASH complex subunit 7	Kiaa1033	mmu:319277			
2104	0.3565	-0.0552	0.4085	0.0277	0	Q9ER72	Cysteine--tRNA ligase, cytoplasmic	Cars	mmu:27267			
2105	-0.1029	0.3786	0.3106	0.0279	0	P59999	Actin-related protein 2/3 complex subunit 4	Arpc4	mmu:68089			
2106	-0.4244	0.1377	0.3566	0.0280	0	Q8K310	Matrin-3	Matr3	mmu:17184			
2107	0.2118	0.1981	-0.3338	0.0280	0	Q8K4Z5	Splicing factor 3A subunit 1	Sf3a1	mmu:67465			
2108	-0.3923	1.1613	-0.6015	0.0284	0	P54726	UV excision repair protein RAD23 homolog A	Rad23a	mmu:19358			
2109	-0.0975	0.0284	-0.1517	0.0287	0	Q9Z1T1	AP-3 complex subunit beta-1	Ap3b1	mmu:11774			
2110	-0.0766	-1.6096	0.4964	0.0288	0	Q9QXE7	F-box-like/WD repeat-containing protein TBL1X	Tbl1x	mmu:21372			
2111	0.1639	0.1228	-0.0712	0.0295	0	Q689Z5	Protein strawberry notch homolog 1	Sbno1				
2112	0.2235	-0.1678	-1.4527	0.0297	0	Q61735	Leukocyte surface antigen CD47	Cd47	mmu:16423			
2113	-0.0575	0.6347	0.6303	0.0298	0	O35639	Annexin A3;Annexin	Anxa3	mmu:11745			
2114	-0.1318	0.3066	-0.1849	0.0299	0	Q80TP3	E3 ubiquitin-protein ligase UBR5	Ubr5	mmu:70790		1	
2115	-0.0313	0.6606	-0.5204	0.0304	0	S4R1W5		Rbm6	mmu:19654			
2116	-0.4368	0.4116	0.0579	0.0305	0	Q99M28	RNA-binding protein with serine-rich domain 1	Rnps1	mmu:19826			
2117	-0.2479	-0.6891	0.1022	0.0307	0	P31230	Aminoacyl tRNA synthase complex-interacting multifunctional protein 1;Endothelial monocyte-act	Aimp1				
2118	0.2847	-0.4677	-0.0695	0.0313	0	P62874	Guanine nucleotide-binding protein G(I)/G(S)/G(T) subunit beta-1	Gnb1;Gnb	mmu:14688			
2119	-0.4807	-0.2555	0.3655	0.0320	0	Q9CX56	26S proteasome non-ATPase regulatory subunit 8	Psmd8	mmu:57296			
2120	0.0101	0.1435	-0.1419	0.0321	0	Q3UIX4		Srsf11	mmu:69207			
2121	0.5680	-0.1176	0.0486	0.0323	0	Q9WUM4	Coronin-1C	Coro1c	mmu:23790			
2122	-0.0390	0.2106	0.3718	0.0330	0	P20152	Vimentin	Vim	mmu:22352			
2123	-0.1643	0.2228	0.1676	0.0330	0	P48036	Annexin A5	Anxa5	mmu:11747			
2124	0.2116	-1.0570	0.2801	0.0331	0	Q9EPK6	Nucleotide exchange factor SIL1	Sil1	mmu:81500			
2125	-0.5635	0.4688	0.2911	0.0333	0	P52480	Pyruvate kinase PKM	Pkm	mmu:18746			
2126	0.1811	-0.2751	0.0951	0.0336	0	P80313	T-complex protein 1 subunit eta	Cct7	mmu:12468			
2127	-0.5810	-0.1381	0.3687	0.0336	0	Q9ER38	Torsin-3A	Tor3a	mmu:30935			
2128	0.0262	-0.1804	-0.2840	0.0359	0	Q8K2H2	OTU domain-containing protein 6B	Otud6b	mmu:72201			
2129	-0.3884	1.5550	-0.2755	0.0361	0	Q61823	Programmed cell death protein 4	Pdcd4	mmu:18569			
2130	0.2386	-0.0418	-0.5746	0.0364	0	Q80U72	Protein scribble homolog	Scrib	mmu:105782			
2131	-0.0766	-0.0812	2.0053	0.0365	0	Q8JZY2	COMM domain-containing protein 10	Commd10	mmu:69456			

2132	0.0726	-0.0214	-0.0439	0.0371	0	Q61074	Protein phosphatase 1G	Ppm1g	mmu:14208		1		
2133	-0.6585	0.0846	-0.5750	0.0380	0	Q64521	Glycerol-3-phosphate dehydrogenase;Glycerol-3-phosphate dehydrogenase, mitochondrial	Gpd2	mmu:14571				
2134	0.2111	-0.0395	-0.4374	0.0386	0	Q9CQC6	Basic leucine zipper and W2 domain-containing protein 1	Bzw1	mmu:66882				
2135	-0.4404	0.0996	-0.8589	0.0393	0	Q80ZW2	Protein THEM6	Them6	mmu:223626				
2136	-0.1241	0.1113	-0.0352	0.0408	0	Q02819	Nucleobindin-1	Nucb1	mmu:18220				
2137	0.0128	0.4635	-0.1763	0.0416	0	Q8C156	Condensin complex subunit 2	Ncaph	mmu:215387				
2138	-0.1494	-0.1372	0.2302	0.0423	0	P62334	26S protease regulatory subunit 10B	Psmc6	mmu:67089				
2139	0.2163	-0.2288	-0.3366	0.0425	0	Q9DC29	ATP-binding cassette sub-family B member 6, mitochondrial	Abcb6	mmu:74104				
2140	-0.0176	0.0061	-0.3119	0.0428	0	Q9CQW9	Interferon-induced transmembrane protein 3	Ifitm3	mmu:66141				
2141	0.1534	0.8186	-0.0331	0.0428	0	Q7TPM1	Protein PRRC2B	Prrc2b	mmu:227723				
2142	0.0102	0.1042	-0.3676	0.0429	0	Q8CHW4	Translation initiation factor eIF-2B subunit epsilon	Eif2b5	mmu:224045				
2143	-0.0556	-0.2762	0.0863	0.0429	0	O70318	Band 4,1-like protein 2	Epb41l2	mmu:13822				
2144	-0.0044	-0.3893	0.1857	0.0432	0	G5E8V9		Arfip1	mmu:99889				
2145	-0.4735	0.5475	-0.2927	0.0434	0	Q9CSU0	Regulation of nuclear pre-mRNA domain-containing protein 1B	Rprd1b	mmu:70470				
2146	0.3205	-0.1714	0.1419	0.0441	0	Q8CFI7	DNA-directed RNA polymerase II subunit RPB2	Polr2b	mmu:231329				
2147	0.0606	-0.0181	0.1219	0.0441	0	Q8CGC7	Bifunctional glutamate/proline--tRNA ligase;Glutamate--tRNA ligase;Proline--tRNA ligase	Eprs	mmu:107508				
2148	-0.5061	0.3569	-0.4309	0.0442	0	Q8BGE6	Cysteine protease ATG4B	Atg4b	mmu:66615				
2149	0.0179	0.6833	-0.7075	0.0444	0	Q9JJY4	Probable ATP-dependent RNA helicase DDX20	Ddx20	mmu:53975				1
2150	-0.3704	0.2659	0.3556	0.0454	0	P63094	Guanine nucleotide-binding protein G(s) subunit alpha isoforms XLas;Guanine nucleotide-binding	Gnas	mmu:14683				
2151	-0.5334	0.9093	-0.3077	0.0454	0	P70372	ELAV-like protein 1	Elavl1	mmu:15568				
2152	-0.7001	0.0135	0.1168	0.0456	0	Q9DBG6	Dolichyl-diphosphooligosaccharide--protein glycosyltransferase subunit 2	Rpn2	mmu:20014				
2153	0.5720	0.7333	-0.4252	0.0458	0	O54916	RalBP1-associated Eps domain-containing protein 1	Reps1	mmu:19707				
2154	0.1727	-0.2110	-0.7592	0.0460	0	Q99NH2	Partitioning defective 3 homolog	Pard3	mmu:93742				
2155	-0.2278	-0.0212	0.0304	0.0468	0	Q9D125	28S ribosomal protein S25, mitochondrial	Mrps25	mmu:64658				
2156	-0.2606	0.3472	-0.6706	0.0474	0	Q8K021	Secretory carrier-associated membrane protein 1	Scamp1	mmu:107767				
2157	-0.4465	0.8984	-0.1507	0.0474	0	P05627	Transcription factor AP-1	Jun	mmu:16476	1			
2158	0.0555	0.3119	-0.0690	0.0476	0	P63034	Cytohesin-2	Cyth2	mmu:19158				
2159	-0.0573	-0.2022	0.1962	0.0484	0	B2RQC6	CAD protein;Glutamine-dependent carbamoyl-phosphate synthase;Aspartate carbamoyltransferas	Cad	mmu:69719				
2160	-0.9073	0.1538	0.3695	0.0486	0	Q91VX2	Ubiquitin-associated protein 2	Ubap2	mmu:68926				
2161	0.6464	-0.3045	-0.3670	0.0491	0	Q9Z160	Conserved oligomeric Golgi complex subunit 1	Cog1	mmu:16834				
2162	0.4139	0.2128	-0.0204	0.0493	0	E9Q226		Ccnyl1	mmu:227210				
2163	0.1924	-0.1909	0.2456	0.0498	0	Q99JF8	PC4 and SFRS1-interacting protein	Psip1	mmu:101739	1			
2164	-0.0152	0.0941	0.0497	0.0498	0	P52293	Importin subunit alpha-1	Kpna2	mmu:16647				
2165	-0.6333	-0.0089	0.0082	0.0504	0	Q8VCE2	GPN-loop GTPase 1	Gpn1	mmu:74254				
2166	-0.0270	-0.3275	0.4514	0.0505	0	Q8BMZ5	tRNA-splicing endonuclease subunit Sen34	Tsen34	mmu:66078				
2167	-0.4122	0.0682	-0.3376	0.0510	0	O35972	39S ribosomal protein L23, mitochondrial	Mrpl23	mmu:19935				
2168	0.3863	-0.2326	[illegible]	0.0515	0	Q9D1M7	Peptidyl-prolyl cis-trans isomerase FKBP11	Fkbp11	mmu:66120				
2169	-0.1929	0.9174	-0.5207	0.0518	0	Q3TC93	HCLS1-binding protein 3	Hs1bp3	mmu:58240				
2170	0.0078	-0.1139	-0.0641	0.0519	0	Q9QYE6	Golgin subfamily A member 5	Golga5	mmu:27277				
2171	-0.5714	0.3726	0.4222	0.0530	0	Q8BTV2	Cleavage and polyadenylation specificity factor subunit 7	Cpsf7	mmu:269061				
2172	0.2687	-0.1203	-0.1961	0.0535	0	B9EJ86	Oxysterol-binding protein	Osbpl8	mmu:237542				
2173	-0.0661	0.3025	0.0470	0.0541	0	O88967	ATP-dependent zinc metalloprotease YME1L1	Yme1l1	mmu:27377				
2174	0.3034	-0.2715	-0.7795	0.0542	0	P14873	Microtubule-associated protein 1B;MAP1B heavy chain;MAP1 light chain LC1	Map1b	mmu:17755				
2175	0.2116	0.2848	-0.0896	0.0558	0	P48678	Prelamin-A/C;Lamin-A/C	Lmna	mmu:16905				
2176	-0.1397	0.3371	0.0144	0.0561	0	Q810A7	ATP-dependent RNA helicase DDX42	Ddx42	mmu:72047				
2177	-0.1511	-0.3230	0.0177	0.0565	0	P35279	Ras-related protein Rab-6A	Rab6a	mmu:19346				
2178	-0.2411	0.2529	0.0644	0.0566	0	P23116	Eukaryotic translation initiation factor 3 subunit A	Eif3a	mmu:13669				
2179	0.1269	-0.3870	0.1034	0.0569	0	P60710	Actin, cytoplasmic 1;Actin, cytoplasmic 1, N-terminally processed	Actb;Actg1	mmu:11461				
2180	0.5448	0.0204	-0.2396	0.0570	0	Q5SWD9	Pre-rRNA-processing protein TSR1 homolog	Tsr1	mmu:104662				
2181	0.2352	-0.1533	-0.4015	0.0571	0	O08810	116 kDa U5 small nuclear ribonucleoprotein component	Eftud2	mmu:20624				
2182	0.1231	0.5458	-0.9101	0.0571	0	B2RQ71		Dip2c	mmu:208440				
2183	-0.1880	-0.1265	0.2203	0.0577	0	Q8BU30	Isoleucine--tRNA ligase, cytoplasmic	Iars	mmu:105148				
2184	-0.3291	-0.1971	0.3368	0.0577	0	P14576	Signal recognition particle 54 kDa protein	Srp54;Srp5	mmu:24067				
2185	-0.1062	0.3544	-0.3560	0.0581	0	Q99M08	Uncharacterized protein C4orf3 homolog		mmu:67704				
2186	-0.5134	-0.4187	0.6107	0.0589	0	O88958	Glucosamine-6-phosphate isomerase 1	Gnpda1	mmu:26384				
2187	0.2710	0.8341	-0.3287	0.0596	0	A6H619	PHD and RING finger domain-containing protein 1	Phrf1	mmu:101471				
2188	-0.1595	0.3073	-0.1707	0.0600	0	Q99JX4	Eukaryotic translation initiation factor 3 subunit M	Eif3m	mmu:98221				
2189	-0.3581	-0.0351	0.0390	0.0606	0	O88271	Craniofacial development protein 1	Cfdp1	mmu:23837				1
2190	0.1537	-0.2216	0.3946	0.0612	0	Q8R311	cTAGE family member 5;Melanoma inhibitory activity protein 2	Ctage5;Mia2					
2191	-0.7323	0.0891	-0.7159	0.0612	0	Q9R1Q9	V-type proton ATPase subunit S1	Atp6ap1	mmu:54411				
2192	-1.1387	0.3613	0.6942	0.0613	0	P53026	Ribosomal protein;60S ribosomal protein L10a	Rpl10a					
2193	-0.2692	0.0643	0.0759	0.0622	0	Q8CFC7	CLK4-associating serine/arginine rich protein	Clasrp	mmu:53609				
2194	-0.1167	-0.3196	0.0189	0.0627	0	Q9D2G2	Dihydrolipoyllysine-residue succinyltransferase component of 2-oxoglutarate dehydrogenase com	Dlst	mmu:78920				
2195	0.4138	-0.1295	-1.1174	0.0629	0	Q80UM7	Mannosyl-oligosaccharide glucosidase	Mogs	mmu:57377				
2196	0.1424	-0.0833	0.0175	0.0635	0	P11983	T-complex protein 1 subunit alpha	Tcp1	mmu:21454				
2197	-0.2812	1.1119	0.8708	0.0650	0	Q6RHR9	Membrane-associated guanylate kinase, WW and PDZ domain-containing protein 1	Magi1	mmu:14924				
2198	-0.2130	0.8327	-0.0178	0.0654	0	Q01320	DNA topoisomerase 2-alpha	Top2a	mmu:21973				
2199	0.0344	-1.2446	-0.0303	0.0655	0	E9Q512		Trip11	mmu:109181				
2200	-0.4584	-0.1431	0.5453	0.0656	0	Q924K8	Metastasis-associated protein MTA3	Mta3	mmu:116871				
2201	0.3194	-0.2964	0.2375	0.0659	0	Q9CQJ4	E3 ubiquitin-protein ligase RING2	Rnf2	mmu:19821			1	
2202	-0.1534	0.6236	0.3556	0.0663	0	P70671	Interferon regulatory factor 3	Irf3	mmu:54131				
2203	0.1403	-0.1874	-0.2236	0.0666	0	Q91YQ5	Dolichyl-diphosphooligosaccharide--protein glycosyltransferase subunit 1	Rpn1	mmu:103963				
2204	-0.3112	0.1213	0.2370	0.0672	0	Q62167	ATP-dependent RNA helicase DDX3X;Putative ATP-dependent RNA helicase Pl10	Ddx3x;D1P	mmu:13205				
2205	-0.0998	0.6817	-0.2872	0.0675	0	Q61550	Double-strand-break repair protein rad21 homolog	Rad21	mmu:19357				
2206	0.4095	-0.3161	0.2611	0.0683	0	P80317	T-complex protein 1 subunit zeta	Cct6a	mmu:12466				
2207	-0.1847	-0.0976	0.3026	0.0685	0	Q8BKZ9	Pyruvate dehydrogenase protein X component, mitochondrial	Pdhx	mmu:27402				
2208	0.1901	0.5385	-0.9961	0.0685	0	Q8BZQ7	Anaphase-promoting complex subunit 2	Anapc2	mmu:99152			1	
2209	-0.1664	0.5398	0.4462	0.0694	0	Q62011	Podoplanin	Pdpn	mmu:14726				
2210	0.3873	-0.1271	0.2996	0.0698	0	Q8K3H0	DCC-interacting protein 13-alpha	Appl1	mmu:72993				
2211	0.1277	0.5804	-0.8001	0.0698	0	Q921M3	Splicing factor 3B subunit 3	Sf3b3	mmu:101943				
2212	0.3399	-0.2474	0.1319	0.0717	0	Q61753	D-3-phosphoglycerate dehydrogenase	Phgdh	mmu:236539				
2213	0.2087	1.5263	-0.7976	0.0717	0	Q8CH02	SURP and G-patch domain-containing protein 1	Sugp1	mmu:70616				
2214	0.8232	-0.0668	0.0786	0.0720	0	Q3UKC1	Tax1-binding protein 1 homolog	Tax1bp1	mmu:52440				1
2215	0.0470	-0.0502	-0.1091	0.0722	0	Q6P4T2	U5 small nuclear ribonucleoprotein 200 kDa helicase	Snrnp200	mmu:320632				
2216	0.8814	0.0786	-0.2884	0.0722	0	Q922B9	Sperm-specific antigen 2 homolog	Ssfa2	mmu:70599				
2217	-0.0835	0.3617	0.5106	0.0731	0	P63154	Crooked neck-like protein 1	Crnkl1	mmu:66877				
2218	-0.9848	0.6279	-0.0926	0.0735	0	O09005	Sphingolipid delta(4)-desaturase DES1	Degs1	mmu:13244				
2219	-0.0636	0.0891	0.0322	0.0742	0	Q9JHU4	Cytoplasmic dynein 1 heavy chain 1	Dync1h1	mmu:13424				
2220	1.2150	0.2804	-0.0217	0.0746	0	Q99LB6	Methionine adenosyltransferase 2 subunit beta	Mat2b	mmu:108645				
2221	0.1006	-0.0194	0.3993	0.0748	0	Q8CG47	Structural maintenance of chromosomes protein;Structural maintenance of chromosomes protein	Smc4	mmu:70099				
2222	0.1324	-0.4125	0.4961	0.0753	0	Q8CCJ3	E3 UFM1-protein ligase 1	Ufl1	mmu:67490				
2223	-0.5115	-0.3380	0.1511	0.0758	0	Q5SSZ5	Tensin-3	Tns3	mmu:319939		1		
2224	-0.4485	0.5198	-0.4677	0.0760	0	P06151	L-lactate dehydrogenase;L-lactate dehydrogenase A chain	Ldha	mmu:16828				
2225	0.3587	-0.5340	-0.5384	0.0766	0	Q9CQ79	Thioredoxin domain-containing protein 9	Txndc9	mmu:98258				
2226	0.1403	0.2733	-0.1883	0.0766	0	Q8K2Z4	Condensin complex subunit 1	Ncapd2	mmu:68298				
2227	-0.6417	0.0780	0.4974	0.0768	0	Q8VBV7	COP9 signalosome complex subunit 8	Cops8	mmu:108679				
2228	-0.0734	-0.0879	0.5195	0.0768	0	Q9R0Q7	Prostaglandin E synthase 3	Ptges3	mmu:56351				
2229	-0.5357	0.6588	0.1553	0.0769	0	Q99K48	Non-POU domain-containing octamer-binding protein	Nono	mmu:53610				
2230	0.9586	-0.3335	0.1138	0.0776	0	Q9CX34	Suppressor of G2 allele of SKP1 homolog	Sugt1	mmu:67955				
2231	-0.1669	0.9243	-0.9548	0.0778	0	Q9DBX2	Phosducin-like protein	Pdcl	mmu:67466				
2232	0.2873	-0.1383	0.1990	0.0778	0	Q8R570	Synaptosomal-associated protein 47	Snap47	mmu:67826				
2233	-0.0246	0.4435	-0.2970	0.0778	0	P51943	Cyclin-A2	Ccna2	mmu:12428	1			
2234	0.5028	-0.1631	-0.1515	0.0795	0	Q3UZ39	Leucine-rich repeat flightless-interacting protein 1	Lrrfip1	mmu:16978				
2235	0.4906	-0.3370	-0.0792	0.0800	0	O88447	Kinesin light chain 1	Klc1					
2236	-0.2992	0.0688	0.2315	0.0801	0	G3X922		Dnajc13	mmu:235567				
2237	-0.4781	0.2338	0.0863	0.0805	0	P70333	Heterogeneous nuclear ribonucleoprotein H2	Hnrnph2	mmu:56258				
2238	-0.0390	0.1563	-0.1278	0.0806	0	Q9Z0X1	Apoptosis-inducing factor 1, mitochondrial	Aifm1	mmu:26926	1			1
2239	-0.4325	0.1280	-0.4536	0.0821	0	P52825	Carnitine O-palmitoyltransferase 2, mitochondrial	Cpt2	mmu:12896				
2240	-0.3805	-0.2882	0.0395	0.0824	0	Q91WS0	CDGSH iron-sulfur domain-containing protein 1	Cisd1	mmu:52637				
2241	-0.0183	0.2544	[illegible]	0.0825	0	Q9R0E1	Procollagen-lysine,2-oxoglutarate 5-dioxygenase 3	Plod3	mmu:26433				
2242	-0.0890	-0.1049	0.5020	0.0828	0	Q62419	Endophilin-A2	Sh3gl1	mmu:20405				
2243	-0.0543	-0.4229	0.1106	0.0831	0	Q58A65	C-Jun-amino-terminal kinase-interacting protein 4	Spag9	mmu:70834				
2244	-1.2576	1.2182	0.4501	0.0841	0	Q3UHU5	Microtubule cross-linking factor 1	Mtcl1	mmu:68617				
2245	0.0988	-0.4868	1.1603	0.0845	0	Q5U3K5	Rab-like protein 6	Rabl6	mmu:227624				
2246	0.6780	0.1839	-0.3347	0.0848	0	P97304	DNA-directed RNA polymerases I and III subunit RPAC2	Polr1d	mmu:20018				
2247	-0.5114	-0.3753	0.2619	0.0854	0	Q91W50	Cold shock domain-containing protein E1	Csde1	mmu:229663				
2248	-0.0148	0.0157	-0.9121	0.0859	0	E9Q411		Nbas	mmu:71169				
2249	-0.0114	0.1220	-0.1861	0.0860	0	Q8C079	Striatin-interacting protein 1	Strip1	mmu:229707				
2250	0.3536	-0.6190	0.3682	0.0865	0	Q8VC70	RNA-binding motif, single-stranded-interacting protein 2	Rbms2	mmu:56516				
2251	-0.0653	0.8688	-0.4798	0.0874	0	Q8BW70	Ubiquitin carboxyl-terminal hydrolase 38	Usp38	mmu:74841				
2252	0.1949	0.1371	-0.0872	0.0874	0	O55201	Transcription elongation factor SPT5	Supt5h	mmu:20924				
2253	-0.4966	0.5660	0.2486	0.0883	0	P42232	Signal transducer and activator of transcription 5B;Signal transducer and activator of transcription	Stat5b;Stat	mmu:20851				1
2254	-0.0430	-0.6073	0.2802	0.0885	0	Q8VE47	Ubiquitin-like modifier-activating enzyme 5	Uba5	mmu:66663				
2255	0.0338	-0.3984	-0.2435	0.0885	0	P49718	DNA helicase;DNA replication licensing factor MCM5	Mcm5					
2256	0.0078	-0.6086	-1.1843	0.0887	0	O35166	Golgi SNAP receptor complex member 2	Gosr2	mmu:56494				
2257	-0.4880	0.0469	-0.4910	0.0891	0	Q61425	Hydroxyacyl-coenzyme A dehydrogenase, mitochondrial	Hadh	mmu:15107				

2258	0.2561	-0.1218	-0.1094	0.0897	0	Q6PAR5	GTPase-activating protein and VPS9 domain-containing protein 1	Gapvd1	mmu:66691				
2259	0.8135	0.1092	-0.3361	0.0904	0	P14211	Calreticulin	Calr	mmu:12317				
2260	-0.8772	0.0509	0.7595	0.0904	0	Q8VC31	Coiled-coil domain-containing protein 9	Ccdc9	mmu:243846				
2261	-0.3261	0.2040	-0.0699	0.0905	0	Q8C2E7	WASH complex subunit strumpellin	Kiaa0196	mmu:223593				
2262	0.0359	-0.4116	-0.2909	0.0905	0	Q60961	Lysosomal-associated transmembrane protein 4A	Laptm4a					
2263	0.5833	-0.7053	-0.4092	0.0908	0	Q80ZS3	28S ribosomal protein S26, mitochondrial	Mrps26	mmu:99045				
2264	0.0788	0.0866	-0.1347	0.0910	0	Q8K1M6	Dynamin-1-like protein	Dnm1l	mmu:74006				
2265	-0.7326	0.6211	0.0437	0.0914	0	Q8CFE3	REST corepressor 1	Rcor1	mmu:217864				
2266	0.4776	-0.0542	0.7316	0.0917	0	A0A0A6YWB0		Mbnl1					
2267	0.0095	0.1541	-0.1281	0.0922	0	Q9Z1K6	E3 ubiquitin-protein ligase ARIH2	Arih2	mmu:23807			1	
2268	0.4319	-0.2668	-0.4936	0.0925	0	Q80U35	Rho guanine nucleotide exchange factor 17	Arhgef17	mmu:207212				
2269	0.3449	-0.0777	-0.7891	0.0926	0	Q9QXK3	Coatomer subunit gamma-2	Copg2	mmu:54160				
2270	0.4098	-0.2701	-1.1184	0.0930	0	P27546	Microtubule-associated protein 4	Map4	mmu:17758				
2271	0.3379	-0.0549	0.3126	0.0943	0	P68510	14-3-3 protein eta	Ywhah	mmu:22629				
2272	0.6591	-0.1009	-0.2777	0.0943	0	Q3UHH1	Zinc finger SWIM domain-containing protein 8	Zswim8	mmu:268721				
2273	0.1148	0.6358	-0.2005	0.0945	0	P26450	Phosphatidylinositol 3-kinase regulatory subunit alpha	Pik3r1	mmu:18708				
2274	-0.1412	-0.0384	0.0897	0.0959	0	P70353	Nuclear transcription factor Y subunit gamma	Nfyc	mmu:18046				
2275	-0.0797	-0.0276	0.0242	0.0962	0	Q8VDN2	Sodium/potassium-transporting ATPase subunit alpha-1	Atp1a1	mmu:11928				
2276	-0.2759	0.2072	-0.3850	0.0968	0	O35609	Secretory carrier-associated membrane protein 3	Scamp3	mmu:24045				
2277	0.1269	-0.0785	-0.2807	0.0970	0	Q80SW1	Putative adenosylhomocysteinase 2	Ahcyl1	mmu:229709				
2278	-0.3712	-0.1201	0.6561	0.0970	0	Q6ZPE2	Myotubularin-related protein 5	Sbf1	mmu:77980		1		
2279	0.1537	0.2559	-0.0565	0.0972	0	Q920E5	Farnesyl pyrophosphate synthase	Fdps	mmu:110196				
2280	-0.0971	-0.5571	0.9974	0.0973	0	Q921C5	Protein bicaudal D homolog 2	Bicd2	mmu:76895				
2281	-0.3818	0.9845	-0.2001	0.0975	0	Q921E2	Ras-related protein Rab-31	Rab31	mmu:106572				
2282	0.6565	0.0439	-0.0315	0.0980	0	O88811	Signal transducing adapter molecule 2	Stam2	mmu:56324				
2283	-0.0357	0.3296	0.6729	0.0987	0	Q8CDG3	Deubiquitinating protein VCIP135	Vcpip1	mmu:70675				
2284	-0.1006	0.1004	-0.1727	0.0988	0	P54731	FAS-associated factor 1	Faf1	mmu:14084				
2285	-0.1325	0.0452	0.2178	0.0990	0	Q9QZQ1	Afadin	Mllt4	mmu:17356				
2286	0.5097	-0.1046	-0.2048	0.0993	0	Q9CXW2	28S ribosomal protein S22, mitochondrial	Mrps22	mmu:64655				
2287	1.5281	-0.5413	-1.2257	0.0994	0	Q9D483	DNA-directed RNA polymerase III subunit RPC3	Polr3c	mmu:74414				
2288	0.0328	-0.0251	-0.0249	0.0996	0	P37040	NADPH--cytochrome P450 reductase	Por	mmu:18984				
2289	0.3915	-0.4758	-0.0351	0.1010	0	Q3TXS7	26S proteasome non-ATPase regulatory subunit 1	Psmd1	mmu:70247				
2290	-0.7025	0.2207	0.2677	0.1014	0	Q3UPF5	Zinc finger CCCH-type antiviral protein 1	Zc3hav1	mmu:78781				
2291	0.2332	-0.3374	-0.0597	0.1014	0	O88738	Baculoviral IAP repeat-containing protein 6	Birc6	mmu:12211				1
2292	-0.1682	0.1782	0.2976	0.1019	0	P51660	Peroxisomal multifunctional enzyme type 2;(3R)-hydroxyacyl-CoA dehydrogenase;Enoyl-CoA hydra	Hsd17b4	mmu:15488				
2293	-0.4228	0.4968	[illegible]	0.1031	0	Q99KG3	RNA-binding protein 10	Rbm10	mmu:236732				
2294	-0.2783	0.3543	-0.2343	0.1033	0	Q8CFE6	Sodium-coupled neutral amino acid transporter 2	Slc38a2	mmu:67760				
2295	-0.1438	-0.0382	0.0388	0.1042	0	Q8BXZ1	Protein disulfide-isomerase TMX3	Tmx3	mmu:67988				
2296	0.0504	-0.2156	0.2701	0.1045	0	Q68FL6	Methionine--tRNA ligase, cytoplasmic	Mars	mmu:216443				
2297	0.2698	0.3598	-0.2212	0.1048	0	Q9D2E2	Target of EGR1 protein 1	Toe1	mmu:68276				
2298	0.0763	0.1743	-0.0481	0.1050	0	O08759	Ubiquitin-protein ligase E3A	Ube3a	mmu:22215			1	
2299	-0.3818	0.4000	-0.5007	0.1059	0	Q80X82	Symplekin	Sympk					
2300	0.3593	0.4560	-0.8524	0.1060	0	Q8CFD4	Sorting nexin-8	Snx8	mmu:231834				
2301	0.4957	0.1048	-0.5113	0.1064	0	Q3TC46	Protein PAT1 homolog 1	Patl1	mmu:225929				
2302	-0.1365	0.5146	0.5791	0.1064	0	P14824	Annexin A6	Anxa6	mmu:11749				
2303	-0.6097	0.5299	0.3168	0.1073	0	P67984	60S ribosomal protein L22	Rpl22	mmu:19934				
2304	-1.9084	-0.1241	0.5602	0.1076	0	P17809	Solute carrier family 2, facilitated glucose transporter member 1	Slc2a1	mmu:20525				
2305	-0.0116	1.0752	-0.2606	0.1077	0	Q8BG30	Negative elongation factor A	Nelfa	mmu:24116				
2306	-0.2174	0.0087	-0.0125	0.1082	0	Q68FD5	Clathrin heavy chain;Clathrin heavy chain 1	Cltc	mmu:67300				
2307	-0.2013	-0.0593	0.3652	0.1085	0	Q922Y1	UBX domain-containing protein 1	Ubxn1	mmu:225896				
2308	0.2547	-0.3295	0.0828	0.1086	0	Q08122	Transducin-like enhancer protein 3	Tle3	mmu:21887				
2309	0.7006	-0.5258	-0.2038	0.1086	0	Q99JY4	TraB domain-containing protein	Trabd	mmu:67976				
2310	-0.0228	-0.0092	0.0829	0.1087	0	Q7TT37	Elongator complex protein 1	Ikbkap	mmu:230233				
2311	0.0928	-0.1890	-0.2581	0.1089	0	P41216	Long-chain-fatty-acid--CoA ligase 1	Acsl1	mmu:14081				
2312	-0.1157	0.1230	0.0238	0.1090	0	P23188	Furin	Furin	mmu:18550				
2313	0.4700	-0.2638	1.6328	0.1098	0	Q99PP9	Tripartite motif-containing protein 16	Trim16	mmu:94092				
2314	0.1687	-0.5612	0.1530	0.1104	0	P60122	RuvB-like 1	Ruvbl1	mmu:56505				
2315	0.1529	-0.2423	-0.0204	0.1104	0	P81117	Nucleobindin-2;Nesfatin-1	Nucb2	mmu:53322				
2316	0.1278	-0.2309	-0.0674	0.1114	0	Q59J78	Mimitin, mitochondrial	Ndufaf2	mmu:75597				
2317	0.4678	0.0642	-0.2544	0.1117	0	Q8BHK9	DNA excision repair protein ERCC-6-like	Ercc6l	mmu:236930				
2318	-0.1292	-0.0741	0.3716	0.1118	0	Q5SWU9	Acetyl-CoA carboxylase 1;Biotin carboxylase	Acaca	mmu:107476				
2319	0.1006	0.5143	-0.5805	0.1124	0	Q9ER00	Syntaxin-12	Stx12	mmu:100226				
2320	0.4537	-0.1068	-0.2604	0.1127	0	Q8BL80	Rho GTPase-activating protein 22	Arhgap22	mmu:239027				
2321	0.0707	-0.2594	0.4544	0.1131	0	Q99JX3	Golgi reassembly-stacking protein 2	Gorasp2	mmu:70231				
2322	0.7345	-0.3736	0.0277	0.1134	0	Q9JIG7	Coiled-coil domain-containing protein 22	Ccdc22	mmu:54638				
2323	-0.1958	0.3043	0.3361	0.1146	0	Q924W5	Structural maintenance of chromosomes protein 6	Smc6	mmu:67241				
2324	0.8077	-1.7643	0.2157	0.1147	0	Q8K4P0	pre-mRNA 3 end processing protein WDR33	Wdr33	mmu:74320				
2325	-0.4616	-0.9440	1.0809	0.1153	0	O54962	Barrier-to-autointegration factor;Barrier-to-autointegration factor, N-terminally processed	Banf1	mmu:23825				
2326	0.4054	-1.1614	0.1507	0.1159	0	P57746	V-type proton ATPase subunit D	Atp6v1d	mmu:73834				
2327	-0.3444	0.3142	-0.3473	0.1159	0	Q99NB8	Ubiquilin-4	Ubqln4	mmu:94232				
2328	0.1888	-0.2462	-0.3123	0.1163	0	P54775	26S protease regulatory subunit 6B	Psmc4	mmu:23996				
2329	-0.1335	0.6741	0.2559	0.1166	0	P39054	Dynamin-2	Dnm2	mmu:13430	1			
2330	-0.0510	0.0162	-0.1783	0.1174	0	P10852	4F2 cell-surface antigen heavy chain	Slc3a2	mmu:17254				
2331	-0.4699	0.0645	0.2666	0.1176	0	Q8BWY9	Protein CIP2A	C330027C0	mmu:224171				
2332	0.4948	-0.4028	-0.2281	0.1179	0	Q9JJ28	Protein flightless-1 homolog	Flii	mmu:14248				
2333	-0.5340	0.7489	0.5308	0.1183	0	P11031	Activated RNA polymerase II transcriptional coactivator p15	Sub1	mmu:20024				
2334	0.1256	[illegible]	0.1238	0.1191	0	Q8CI59	Metalloreductase STEAP3	Steap3	mmu:68428				
2335	[illegible]	-0.5072	0.8761	0.1191	0	O35344	Importin subunit alpha-4	Kpna3	mmu:16648				
2336	0.4609	0.0589	-0.1083	0.1196	0	Q05CL8	La-related protein 7	Larp7	mmu:28036				
2337	-0.0847	0.1292	-0.1700	0.1197	0	Q9WUP7	Ubiquitin carboxyl-terminal hydrolase isozyme L5	Uchl5	mmu:56207				
2338	0.0924	0.4718	-0.3114	0.1199	0	Q6ZQ88	Lysine-specific histone demethylase 1A	Kdm1a	mmu:99982				
2339	0.5979	-0.1019	-1.4203	0.1205	0	Q99KU0	Vacuole membrane protein 1	Vmp1	mmu:75909				
2340	1.4540	0.1851	-0.1218	0.1207	0	Q922K7	Probable 28S rRNA (cytosine-C(5))-methyltransferase	Nop2					
2341	0.0922	0.0647	-0.1940	0.1210	0	O70503	Very-long-chain 3-oxoacyl-CoA reductase	Hsd17b12	mmu:56348				
2342	0.3437	-0.0139	0.4981	0.1211	0	Q922B2	Aspartate--tRNA ligase, cytoplasmic	Dars	mmu:226414				
2343	-0.1411	0.0294	0.1568	0.1211	0	P50516	V-type proton ATPase catalytic subunit A	Atp6v1a	mmu:11964				
2344	0.2313	-0.0701	-0.6926	0.1214	0	Q62351	Transferrin receptor protein 1	Tfrc	mmu:22042				
2345	-0.4616	-0.0866	0.1425	0.1216	0	Q8BH24	Transmembrane 9 superfamily member 4	Tm9sf4	mmu:99237				
2346	-0.0123	0.2924	-0.2644	0.1218	0	Q9QXK7	Cleavage and polyadenylation specificity factor subunit 3	Cpsf3	mmu:54451				
2347	-0.0638	0.4584	0.1504	0.1220	0	P58771	Tropomyosin alpha-1 chain	Tpm1	mmu:22003	1			
2348	-0.5520	0.1206	0.0460	0.1224	0	O88736	3-keto-steroid reductase	Hsd17b7	mmu:15490				
2349	-0.3308	0.1620	-0.1403	0.1238	0	P97855	Ras GTPase-activating protein-binding protein 1	G3bp1	mmu:27041				
2350	1.4041	-0.1949	0.1564	0.1241	0	Q9D0C1	E3 ubiquitin-protein ligase RNF115	Rnf115	mmu:67845			1	
2351	-0.3846	1.8544	-0.6649	0.1244	0	Q3TA68		Wdr36					
2352	0.1187	-0.0358	0.1878	0.1245	0	P62245	40S ribosomal protein S15a	Rps15a	mmu:267019				
2353	0.7709	-0.1884	-0.2729	0.1247	0	Q80YV2	Nuclear-interacting partner of ALK	Zc3hc1	mmu:232679			1	1
2354	-0.1245	0.2420	-0.1747	0.1251	0	Q8K2V1	Serine/threonine-protein phosphatase 4 regulatory subunit 1	Ppp4r1	mmu:70351				
2355	[illegible]	-0.2469	0.7794	0.1252	0	P35979	60S ribosomal protein L12	Rpl12	mmu:269261				
2356	0.4263	-0.5001	-0.5975	0.1255	0	Q6WKZ8	E3 ubiquitin-protein ligase UBR2	Ubr2	mmu:224826			1	
2357	1.0097	-0.9218	-0.1479	0.1255	0	P00405	Cytochrome c oxidase subunit 2	Mtco2	mmu:17709				
2358	-0.0957	-0.4241	0.2484	0.1257	0	P61202	COP9 signalosome complex subunit 2	Cops2	mmu:12848				
2359	-0.2614	0.4735	-0.2849	0.1263	0	Q7TPV4	Myb-binding protein 1A	Mybbp1a	mmu:18432				
2360	1.3885	-0.0179	-0.6450	0.1267	0	P00375	Dihydrofolate reductase	Dhfr	mmu:13361				
2361	-0.1567	0.0564	-0.4963	0.1276	0	Q8BLF1	Neutral cholesterol ester hydrolase 1	Nceh1	mmu:320024				
2362	0.0182	-0.0049	0.2649	0.1279	0	Q8VHX6	Filamin-C	Flnc	mmu:68794				
2363	0.0451	-0.1669	0.1064	0.1281	0	P51174	Long-chain specific acyl-CoA dehydrogenase, mitochondrial	Acadl	mmu:11363				
2364	-0.0840	0.2547	-0.9817	0.1286	0	Q91VK1	Basic leucine zipper and W2 domain-containing protein 2	Bzw2	mmu:66912				
2365	0.5244	-0.2499	0.3155	0.1288	0	P14685	26S proteasome non-ATPase regulatory subunit 3	Psmd3	mmu:22123				
2366	-0.2402	-0.0643	0.1282	0.1305	0	Q3UPL0	Protein transport protein Sec31A	Sec31a	mmu:69162				
2367	-0.3011	0.1728	-0.0454	0.1307	0	Q9EPL8	Importin-7	Ipo7	mmu:233726				
2368	0.0598	0.3853	-0.0839	0.1313	0	Q62383	Transcription elongation factor SPT6	Supt6h	mmu:20926				
2369	-0.2579	0.3026	0.1341	0.1315	0	Q9R049	E3 ubiquitin-protein ligase AMFR	Amfr	mmu:23802			1	
2370	0.2773	-0.2778	0.1447	0.1315	0	Q9Z0P4	Paralemmin-1	Palm	mmu:18483				
2371	0.5679	0.9331	-0.3025	0.1316	0	P27659	60S ribosomal protein L3	Rpl3	mmu:27367				
2372	0.1611	0.0374	-0.2914	0.1318	0	P42567	Epidermal growth factor receptor substrate 15	Eps15	mmu:13858				
2373	-0.3408	0.0111	0.0486	0.1326	0	P63037	DnaJ homolog subfamily A member 1	Dnaja1	mmu:15502				
2374	0.1651	0.6017	-0.0884	0.1335	0	Q91YR7	Pre-mRNA-processing factor 6	Prpf6	mmu:68879				
2375	-0.2600	0.2814	-0.0660	0.1336	0	Q61990	Poly(rC)-binding protein 2	Pcbp2	mmu:18521				
2376	-0.8703	1.0449	-0.3147	0.1342	0	Q80Z96	Vang-like protein 1	Vangl1	mmu:229658				
2377	0.1557	-0.2732	0.6171	0.1349	0	Q6NXL1		Sec24d	mmu:69608				
2378	-0.2726	0.2449	0.4067	0.1351	0	Q3THE2	Myosin regulatory light chain 12B	Myl12a;My	mmu:67938				
2379	-0.0517	-0.9704	1.5862	0.1352	0	Q8K3D3	DNA repair protein SWI5 homolog	Swi5	mmu:72931				
2380	0.3461	-0.4027	0.2872	0.1354	0	P70206	Plexin-A1	Plxna1	mmu:18844				
2381	0.3606	-0.0892	0.1620	0.1356	0	Q91WV0	Protein Dr1	Dr1	mmu:13486				
2382	-0.2817	0.4526	0.1053	0.1362	0	Q9QXT0	Protein canopy homolog 2	Cnpy2	mmu:56530				
2383	0.3497	0.0831	-0.0931	0.1373	0	Q8R1N4	NudC domain-containing protein 3	Nudcd3	mmu:209586				

2384	-0.0626	0.0876	0.5595	0.1375	0	Q9CVD2	Ataxin-3	Atxn3	mmu:110616			
2385	-0.0993	0.0606	-1.0745	0.1377	0	Q9JKZ2	Sodium/myo-inositol cotransporter	Slc5a3	mmu:53881			
2386	-0.4029	-1.3506	2.3558	0.1385	0	Q920Q4	Vacuolar protein sorting-associated protein 16 homolog	Vps16				
2387	0.0802	0.0311	-0.0925	0.1387	0	Q99N89	39S ribosomal protein L43, mitochondrial	Mrpl43				
2388	-0.7747	0.8136	-0.4263	0.1388	0	P62331	ADP-ribosylation factor 6	Arf6	mmu:11845			
2389	-0.3682	-0.2772	0.0377	0.1389	0	P22437	Prostaglandin G/H synthase 1	Ptgs1	mmu:19224	1		
2390	0.2050	-0.4818	0.1773	0.1391	0	Q6PE01	U5 small nuclear ribonucleoprotein 40 kDa protein	Snrnp40	mmu:66585			
2391	0.0413	-0.0810	0.3808	0.1391	0	Q8VBW6	NEDD8-activating enzyme E1 regulatory subunit	Nae1	mmu:234664			
2392	0.0657	-0.2085	0.1415	0.1392	0	Q09XV5	Chromodomain-helicase-DNA-binding protein 8	Chd8	mmu:67772			
2393	-0.2108	1.2730	-0.0539	0.1394	0	Q61103	Zinc finger protein ubi-d4	Dpf2	mmu:19708			1
2394	-0.0232	-0.0852	0.0662	0.1397	0	O35704	Serine palmitoyltransferase 1	Sptlc1	mmu:268656			
2395	-0.3697	0.5699	0.5449	0.1398	0	Q91Z67	SLIT-ROBO Rho GTPase-activating protein 2	Srgap2	mmu:14270			
2396	0.3053	-0.1977	0.3023	0.1403	0	O35226	26S proteasome non-ATPase regulatory subunit 4	Psmd4	mmu:19185			
2397	0.3807	-0.1100	-0.3046	0.1410	0	P55937	Golgin subfamily A member 3	Golga3	mmu:269682			
2398	-0.0181	0.3434	0.2078	0.1415	0	Q8VDJ3	Vigilin	Hdlbp	mmu:110611			
2399	0.1001	1.6570	-0.1903	0.1416	0	Q9R0H0	Peroxisomal acyl-coenzyme A oxidase 1	Acox1	mmu:11430			
2400	0.4582	0.8955	-0.2509	0.1417	0	Q9ESU6	Bromodomain-containing protein 4	Brd4	mmu:57261			
2401	-0.0858	0.0143	-0.3647	0.1420	0	P35293	Ras-related protein Rab-18	Rab18	mmu:19330			
2402	0.4528	-0.0127	0.6010	0.1434	0	P57776	Elongation factor 1-delta	Eef1d	mmu:66656			
2403	-0.4472	-0.6669	1.1747	0.1436	0	Q9CR98	Protein FAM136A	Fam136a	mmu:66488			
2404	0.6528	-0.1243	0.1029	0.1443	0	Q9DCL8	Protein phosphatase inhibitor 2	Ppp1r2	mmu:66849			
2405	0.3197	0.0566	-0.3123	0.1444	0	P21107	Tropomyosin alpha-3 chain	Tpm3;Tpm	mmu:59069			
2406	-0.0870	0.0690	1.0805	0.1444	0	Q9WUK2	Eukaryotic translation initiation factor 4H	Eif4h	mmu:22384			
2407	0.1108	-0.1051	0.0102	0.1446	0	P18872	Guanine nucleotide-binding protein G(o) subunit alpha	Gnao1	mmu:14681	1		
2408	-0.0982	0.4165	-0.4510	0.1446	0	Q04750	DNA topoisomerase 1	Top1	mmu:21969			
2409	0.2304	-0.1220	1.1451	0.1447	0	Q9CQ28	Diphthine--ammonia ligase	Dph6	mmu:66632			
2410	0.3034	-0.0255	0.2403	0.1448	0	Q3B7Z2	Oxysterol-binding protein 1	Osbp	mmu:76303			
2411	-0.2801	0.3996	0.0898	0.1448	0	Q8BG05	Heterogeneous nuclear ribonucleoprotein A3	Gm6793;G	mmu:229279			
2412	-0.4186	0.4303	0.9247	0.1454	0	O35621	Phosphomannomutase 1;Phosphomannomutase	Pmm1	mmu:29858			
2413	-0.1241	0.6724	-0.1029	0.1455	0	Q78IK2	Up-regulated during skeletal muscle growth protein 5	Usmg5	mmu:66477			
2414	-0.3998	0.5491	0.2017	0.1460	0	D3YXK1	Atherin	Samd1	mmu:666704			
2415	-0.1416	0.1405	0.3019	0.1469	0	D3Z0M9		Ddx23	mmu:74351			
2416	0.2349	-0.2929	-0.1697	0.1485	0	Q8BKC5	Importin-5	Ipo5	mmu:70572			
2417	-0.9348	0.6052	-0.3849	0.1490	0	Q9CXC3	Mitochondrial genome maintenance exonuclease 1	Mgme1	mmu:74528			
2418	-0.6011	0.5291	0.0342	0.1492	0	Q9Z204	Heterogeneous nuclear ribonucleoproteins C1/C2	Hnrnpc	mmu:15381			
2419	0.3225	0.3821	[illegible]	0.1496	0	P54276	DNA mismatch repair protein Msh6	Msh6	mmu:17688			1
2420	0.0930	0.2071	-0.3768	0.1498	0	P49717	DNA replication licensing factor MCM4	Mcm4	mmu:17217			
2421	[illegible]	0.2017	0.5492	0.1503	0	Q9Z1D1	Eukaryotic translation initiation factor 3 subunit G	Eif3g	mmu:53356			
2422	-0.0532	0.8892	-0.7788	0.1509	0	O35295	Transcriptional activator protein Pur-beta	Purb	mmu:19291			
2423	-0.0260	0.5571	-0.1844	0.1517	0	P46414	Cyclin-dependent kinase inhibitor 1B	Cdkn1b	mmu:12576			
2424	0.2252	-0.0676	0.0984	0.1520	0	Q5XJY5	Coatomer subunit delta	Arcn1	mmu:213827			
2425	-0.7172	0.2743	0.0961	0.1520	0	Q62093	Serine/arginine-rich splicing factor 2	Srsf2	mmu:20382			
2426	0.2605	-0.1476	-0.3018	0.1523	0	Q9CR26	Vacuolar protein sorting-associated protein VTA1 homolog	Vta1	mmu:66201			
2427	1.2796	0.6984	-1.0398	0.1524	0	Q8BI72	CDKN2A-interacting protein	Cdkn2aip	mmu:70925			
2428	0.4325	0.1388	-0.0907	0.1531	0	Q8VDD8	WAS protein family homolog 1	Wash1	mmu:68767			
2429	-0.1078	0.3469	0.2145	0.1532	0	Q8VDM6	Heterogeneous nuclear ribonucleoprotein U-like protein 1	Hnrnpul1	mmu:232989			
2430	1.3847	0.1284	-0.6436	0.1537	0	Q9CPP0	Nucleoplasmin-3	Npm3	mmu:18150			
2431	0.1998	0.4877	-0.2269	0.1544	0	Q3TDD9	Protein phosphatase 1 regulatory subunit 21	Ppp1r21	mmu:73825			
2432	0.2407	-0.5121	-0.1953	0.1548	0	Q91YE6	Importin-9	Ipo9				
2433	-0.0780	0.3702	-1.0811	0.1554	0	Q99LD9	Translation initiation factor eIF-2B subunit beta	Eif2b2	mmu:217715			
2434	0.1688	0.8273	-0.3739	0.1558	0	Q80X98		Dhx38	mmu:64340			
2435	-0.0539	0.0508	0.1499	0.1558	0	Q8QZY1	Eukaryotic translation initiation factor 3 subunit L	Eif3l	mmu:223691			
2436	-0.2750	0.4406	0.0413	0.1561	0	P53994	Ras-related protein Rab-2A	Rab2a	mmu:59021			
2437	-0.1659	0.2202	-0.0946	0.1562	0	Q64010	Adapter molecule crk	Crk	mmu:12928	1		
2438	0.0324	-0.0499	0.1027	0.1565	0	P62196	26S protease regulatory subunit 8	Psmc5	mmu:19184			
2439	0.5096	0.0458	-0.0468	0.1572	0	P80316	T-complex protein 1 subunit epsilon	Cct5	mmu:12465			
2440	-0.4668	0.3740	-0.3089	0.1573	0	O70133	ATP-dependent RNA helicase A	Dhx9	mmu:13211			
2441	-0.1593	0.8907	-1.5766	0.1574	0	P62806	Histone H4	Hist1h4a	mmu:97122			
2442	-0.0839	0.4451	-1.0170	0.1576	0	P42669	Transcriptional activator protein Pur-alpha	Pura	mmu:19290			
2443	-0.1883	0.6795	-0.3385	0.1576	0	Q9ER64	Oxysterol-binding protein;Oxysterol-binding protein-related protein 5	Osbpl5	mmu:79196			
2444	-0.2356	0.4442	-0.7041	0.1580	0	P42859	Huntingtin	Htt				1
2445	-1.1641	[illegible]	1.5228	0.1582	0	Q9D7E4	UPF0449 protein C19orf25 homolog		mmu:66374			
2446	0.3460	-0.4572	-0.3348	0.1583	0	E9Q1M6		Ankhd1				
2447	-0.7199	0.5759	-0.2421	0.1588	0	Q9JI19	Acidic fibroblast growth factor intracellular-binding protein	Fibp	mmu:58249			
2448	-0.0576	-0.4321	0.4250	0.1591	0	Q80TQ2	Ubiquitin carboxyl-terminal hydrolase CYLD	Cyld	mmu:74256			
2449	-0.5964	0.5104	-0.3886	0.1591	0	Q60634	Flotillin-2	Flot2	mmu:14252			
2450	1.0220	-0.1149	-1.4109	0.1598	0	Q9CQ71	Replication protein A 14 kDa subunit	Rpa3	mmu:68240			
2451	-0.0351	-0.1041	0.2560	0.1599	0	Q3UA06	Pachytene checkpoint protein 2 homolog	Trip13	mmu:69716			
2452	0.2196	-0.0947	0.3622	0.1601	0	P19096	Fatty acid synthase;[Acyl-carrier-protein] S-acetyltransferase;[Acyl-carrier-protein] S-malonyltransf	Fasn	mmu:14104			
2453	-0.3270	-0.0796	0.2497	0.1607	0	Q9QZM0	Ubiquilin-2	Ubqln2	mmu:54609			
2454	-0.0232	0.1558	0.0180	0.1611	0	O35286	Pre-mRNA-splicing factor ATP-dependent RNA helicase DHX15	Dhx15	mmu:13204			
2455	-0.0285	0.2597	-0.3156	0.1611	0	P97310	DNA replication licensing factor MCM2	Mcm2	mmu:17216			
2456	0.1989	0.4269	-0.8051	0.1614	0	O08539	Myc box-dependent-interacting protein 1	Bin1	mmu:30948			
2457	-0.0553	-0.1842	0.1872	0.1615	0	Q8R1U1	Conserved oligomeric Golgi complex subunit 4	Cog4	mmu:102339			
2458	-0.3255	-0.1215	0.1761	0.1616	0	P01897	H-2 class I histocompatibility antigen, L-D alpha chain	H2-L				
2459	0.0356	0.0423	-0.1464	0.1623	0	Q61881	DNA replication licensing factor MCM7	Mcm7	mmu:17220			
2460	0.1254	0.1415	-0.0669	0.1624	0	Q60668	Heterogeneous nuclear ribonucleoprotein D0	Hnrnpd	mmu:11991	1		
2461	0.0446	-0.0261	-0.0341	0.1625	0	P50544	Very long-chain specific acyl-CoA dehydrogenase, mitochondrial	Acadvl	mmu:11370			
2462	-0.3851	0.7234	0.0969	0.1625	0	Q9CS00	Cactin	Cactin	mmu:70312			
2463	-0.2514	-0.4976	0.6005	0.1630	0	Q9EST5	Acidic leucine-rich nuclear phosphoprotein 32 family member B	Anp32b	mmu:67628			
2464	-0.1887	-0.0592	0.3149	0.1640	0	P60843	Eukaryotic initiation factor 4A-I	Eif4a1	mmu:13681			
2465	-0.3233	0.4014	0.3115	0.1644	0	O88569	Heterogeneous nuclear ribonucleoproteins A2/B1	Hnrnpa2b1	mmu:53379			
2466	-0.2870	0.1341	0.1567	0.1646	0	O08992	Syntenin-1	Sdcbp	mmu:53378			
2467	-0.2481	-0.3906	0.4853	0.1656	0	Q8VE88	Protein FAM114A2	Fam114a2	mmu:67726			
2468	0.2274	-0.1081	0.6787	0.1658	0	Q9JHI7	Exosome complex component RRP45	Exosc9	mmu:50911			
2469	-0.1442	-0.2102	0.0868	0.1660	0	Q8BMA6	Signal recognition particle subunit SRP68	Srp68	mmu:217337			
2470	0.3512	0.0469	-0.3282	0.1668	0	P84084	ADP-ribosylation factor 5	Arf5	mmu:11844			
2471	-0.9646	0.2021	0.1127	0.1668	0	Q8VEH3	ADP-ribosylation factor-like protein 8A	Arl8a	mmu:68724			
2472	0.2286	-0.2048	-0.3489	0.1671	0	E9Q585		Slc4a1ap	mmu:20534			
2473	-0.0216	-0.2223	0.1506	0.1676	0	Q9QZE5	Coatomer subunit gamma-1	Copg1	mmu:54161			
2474	0.2675	-0.2084	0.3831	0.1681	0	Q61035	Histidine--tRNA ligase, cytoplasmic	Hars	mmu:15115			
2475	0.7245	-0.3785	0.0712	0.1693	0	P50247	Adenosylhomocysteinase	Ahcy	mmu:11615			
2476	0.0691	-0.3787	-0.1554	0.1697	0	Q8VBX6	Multiple PDZ domain protein	Mpdz	mmu:17475			
2477	0.2664	[illegible]	0.4649	0.1698	0	Q571I9	Aldehyde dehydrogenase family 16 member A1	Aldh16a1	mmu:69748			
2478	-0.2811	0.7400	0.3974	0.1703	0	Q9CWK3	CD2 antigen cytoplasmic tail-binding protein 2	Cd2bp2	mmu:70233			
2479	-1.0133	1.2664	-0.6605	0.1707	0	Q9Z1X9	Cell division control protein 45 homolog	Cdc45	mmu:12544			
2480	0.1897	-0.0761	0.0295	0.1710	0	Q60953	Protein PML	Pml	mmu:18854	1		1
2481	0.1314	-0.3065	0.3399	0.1712	0	Q8BI84	Melanoma inhibitory activity protein 3	Mia3	mmu:338366			
2482	-0.2668	0.2811	0.0513	0.1722	0	Q8BNU0	Armadillo repeat-containing protein 6	Armc6	mmu:76813			
2483	0.1712	-0.8521	0.3268	0.1741	0	Q9CY62	E3 ubiquitin-protein ligase RNF181	Rnf181	mmu:66510		1	
2484	-0.1843	-0.4230	0.6493	0.1746	0	Q8K3W0	BRCA1-A complex subunit BRE	Bre	mmu:107976			1
2485	-0.0744	0.5615	0.0640	0.1747	0	Q9CQV4	Protein FAM134C	Fam134c	mmu:67998			
2486	-0.3898	0.0413	0.3518	0.1748	0	Q9JIF7	Coatomer subunit beta	Copb1	mmu:70349			
2487	-0.1006	-0.0208	0.9478	0.1756	0	Q8BU03	Periodic tryptophan protein 2 homolog	Pwp2	mmu:110816			
2488	0.9372	-0.0228	0.0848	0.1763	0	P40124	Adenylyl cyclase-associated protein 1	Cap1	mmu:12331			
2489	1.2314	-0.2396	0.1706	0.1763	0	Q3UPH7	Rho guanine nucleotide exchange factor 40	Arhgef40	mmu:268739			
2490	-0.0531	0.0966	-0.1105	0.1763	0	Q8R0X7	Sphingosine-1-phosphate lyase 1	Sgpl1	mmu:20397			
2491	-0.0129	-0.0439	0.2945	0.1766	0	Q8VBT9	Tether containing UBX domain for GLUT4	Aspscr1	mmu:68938			
2492	-0.3406	-0.0932	1.1108	0.1768	0	P47857	ATP-dependent 6-phosphofructokinase, muscle type	Pfkm	mmu:18642			
2493	0.4430	-0.3418	0.1217	0.1770	0	Q8R5H1	Ubiquitin carboxyl-terminal hydrolase 15	Usp15	mmu:14479			
2494	0.0405	-0.3501	0.4791	0.1771	0	Q9DAW9	Calponin-3	Cnn3	mmu:71994			
2495	-0.0210	0.3383	0.3341	0.1771	0	Q9JMD0	BUB3-interacting and GLEBS motif-containing protein ZNF207	Znf207;Zfp	mmu:22680			
2496	0.5594	[illegible]	-0.2770	0.1775	0	Q9CZD3	Glycine--tRNA ligase	Gars	mmu:353172			
2497	-0.1492	0.5384	0.1916	0.1777	0	Q99MI1	ELKS/Rab6-interacting/CAST family member 1	Erc1	mmu:111173			1
2498	-0.1776	0.1093	0.0420	0.1779	0	P33611	DNA polymerase alpha subunit B	Pola2	mmu:18969			
2499	0.2510	0.0655	-0.4602	0.1783	0	Q64152	Transcription factor BTF3	Btf3	mmu:218490			
2500	-0.7880	0.5618	-0.5263	0.1784	0	Q8BL97	Serine/arginine-rich splicing factor 7	Srsf7	mmu:225027			
2501	-0.3039	-0.1471	0.4045	0.1784	0	Q8R0X2	CDK2-associated and cullin domain-containing protein 1	Cacul1	mmu:78832			
2502	0.5214	-0.4798	0.5471	0.1785	0	Q8C0E3	Tripartite motif-containing protein 47	Trim47	mmu:217333			
2503	0.3451	-0.0224	-0.0491	0.1786	0	Q501J7	Phosphatase and actin regulator 4	Phactr4	mmu:100169			
2504	-0.6883	0.1836	0.4284	0.1789	0	Q80XQ2	TBC1 domain family member 5	Tbc1d5	mmu:72238			
2505	-0.0709	0.1967	-0.0889	0.1796	0	Q64674	Spermidine synthase	Srm	mmu:20810			
2506	0.0391	0.2138	-0.3260	0.1797	0	Q7JJ13	Bromodomain-containing protein 2	Brd2	mmu:14312			
2507	-0.2920	0.4221	0.2467	0.1808	0	Q8K4Q8	Collectin-12	Colec12	mmu:140792			
2508	0.0941	-0.0710	0.2860	0.1815	0	Q9D8W5	26S proteasome non-ATPase regulatory subunit 12	Psmd12	mmu:66997			
2509	-0.7019	0.2968	0.0428	0.1818	0	Q9D4H8	Cullin-2	Cul2	mmu:71745		1	1

2510	0.6646	-0.1522	0.1329	0.1823	0	Q6NVG1	Lysophospholipid acyltransferase LPCAT4	Lpcat4	mmu:99010				
2511	0.1267	0.1583	-0.1267	0.1824	0	P56546	C-terminal-binding protein 2	Ctbp2	mmu:13017				
2512	1.5548	0.3302	-1.0401	0.1825	0	Q9WU28	Prefoldin subunit 5	Pfdn5	mmu:56612				
2513	-0.5953	0.0324	0.2777	0.1831	0	O55022	Membrane-associated progesterone receptor component 1	Pgrmc1	mmu:53328				
2514	0.6589	-0.4238	0.1920	0.1834	0	Q9JKP8	Chromatin accessibility complex protein 1	Chrac1	mmu:93696				
2515	0.2597	-0.4271	0.4328	0.1839	0	Q91WK0	Leucine-rich repeat flightless-interacting protein 2	Lrrfip2	mmu:71268				
2516	-0.0298	0.2768	0.2517	0.1842	0	Q99L45	Eukaryotic translation initiation factor 2 subunit 2	Eif2s2	mmu:67204				
2517	-0.4290	-0.1537	0.2940	0.1842	0	Q9CQW2	ADP-ribosylation factor-like protein 8B	Arl8b	mmu:67166				
2518	[illegible]	-0.0426	0.1914	0.1843	0	P23242	Gap junction alpha-1 protein	Gja1	mmu:14609				
2519	0.0862	-0.1174	0.0928	0.1848	0	P63017	Heat shock cognate 71 kDa protein	Hspa8	mmu:15481				
2520	-0.3161	0.3378	-1.1697	0.1853	0	P10833	Ras-related protein R-Ras	Rras	mmu:20130				
2521	-0.1180	0.2809	0.0952	0.1856	0	A6H5Z3	Exocyst complex component 6B	Exoc6b	mmu:75914				
2522	0.9137	0.4682	-0.5912	0.1865	0	Q9D7J4	Cytochrome c oxidase protein 20 homolog	Cox20	mmu:66359				
2523	-0.3255	0.2592	-0.2504	0.1866	0	Q9JHJ0	Tropomodulin-3	Tmod3	mmu:50875				
2524	-0.5791	0.0581	0.2090	0.1872	0	P43883	Perilipin-2	Plin2	mmu:11520				
2525	-0.4418	0.3048	0.5562	0.1873	0	Q8WTY4	Anamorsin	Ciapin1	mmu:109006				
2526	-0.6322	-1.2479	0.9906	0.1877	0	B2RXC6	DNA-directed RNA polymerase	Polr3a	mmu:218832				
2527	-0.0583	0.5476	0.4646	0.1880	0	P62960	Nuclease-sensitive element-binding protein 1	Ybx1	mmu:22608				
2528	0.3190	0.4646	-0.3641	0.1882	0	Q8R0G7	Protein spinster homolog 1	Spns1	mmu:73658				
2529	0.1393	0.6563	-0.3025	0.1883	0	Q8BYA0	Tubulin-specific chaperone D	Tbcd	mmu:108903				
2530	0.3132	-0.1907	-0.6325	0.1891	0	Q9EQ28	DNA polymerase delta subunit 3	Pold3					
2531	-0.2877	0.0823	0.1703	0.1896	0	Q62189	U1 small nuclear ribonucleoprotein A	Snrpa	mmu:53607				
2532	0.0372	0.4559	-2.5286	0.1907	0	P60060	Protein transport protein Sec61 subunit gamma	Sec61g	mmu:20335				
2533	0.9513	-0.2687	-1.5758	0.1922	0	Q9Z0H4	CUGBP Elav-like family member 2	Celf2	mmu:14007				
2534	1.0931	1.6428	-0.4825	0.1929	0	Q9WTV7	E3 ubiquitin-protein ligase RLIM	Rlim	mmu:19820			1	
2535	0.5804	-0.2842	0.5137	0.1945	0	Q8R1N0	Zinc finger protein 830	Znf830	mmu:66983				
2536	-0.4552	1.1665	0.6658	0.1952	0	Q80U78	Pumilio homolog 1	Pum1	mmu:80912				
2537	-0.2641	0.0482	-0.4017	0.1954	0	Q6A099		Gbf1					
2538	0.3227	0.3832	-0.1814	0.1956	0	P46938	Transcriptional coactivator YAP1	Yap1	mmu:22601				
2539	0.0446	0.0161	-0.1858	0.1957	0	Q8VEE4	Replication protein A 70 kDa DNA-binding subunit;Replication protein A 70 kDa DNA-binding subu	Rpa1	mmu:68275				
2540	-0.2431	0.0664	-0.1523	0.1962	0	O54940	BCL2/adenovirus E1B 19 kDa protein-interacting protein 2	Bnip2	mmu:12175				1
2541	1.3397	-0.6838	-1.5986	0.1965	0	Q8CC12	Codanin-1	Cdan1	mmu:68968				
2542	-0.3507	0.7454	-0.1851	0.1984	0	P27048	Small nuclear ribonucleoprotein-associated protein B;Small nuclear ribonucleoprotein-associated	Snrpb;Snrp	mmu:20638				
2543	-0.0626	0.0232	0.0407	0.1989	0	P08113	Endoplasmin	Hsp90b1	mmu:22027				1
2544	0.2681	-0.0176	0.1104	0.1989	0	Q99LT0	Protein dpy-30 homolog	Dpy30	mmu:66310				
2545	-0.2630	0.3595	1.0736	0.1990	0	Q6P8I4	PEST proteolytic signal-containing nuclear protein	Pcnp	mmu:76302				
2546	0.0459	-0.0798	-0.2127	0.1991	0	O70152	Dolichol-phosphate mannosyltransferase subunit 1	Dpm1	mmu:13480				
2547	-0.2758	0.3505	0.6247	0.1996	0	Q62433	Protein NDRG1	Ndrg1	mmu:17988				
2548	0.5628	0.5941	-0.4248	0.2007	0	P17047	Lysosome-associated membrane glycoprotein 2	Lamp2	mmu:16784				
2549	0.1268	0.2278	-0.0527	0.2010	0	Q9JJT9	Phosphorylated adapter RNA export protein	Phax	mmu:56698				
2550	-0.5864	-0.1070	0.1883	0.2013	0	Q8CFI0	E3 ubiquitin-protein ligase NEDD4-like	Nedd4l				1	
2551	-0.3087	0.2678	0.1120	0.2014	0	O08547	Vesicle-trafficking protein SEC22b	Sec22b	mmu:20333				
2552	0.2728	-0.5235	0.1057	0.2031	0	P56183	Ribosomal RNA processing protein 1 homolog A	Rrp1	mmu:18114				
2553	0.0261	-0.0162	0.1880	0.2034	0	P80318	T-complex protein 1 subunit gamma	Cct3	mmu:12462				
2554	-0.1035	0.0903	-0.4030	0.2037	0	Q9D024	Coiled-coil domain-containing protein 47	Ccdc47	mmu:67163				
2555	-0.1817	0.3889	0.2829	0.2038	0	Q9Z1R2	Large proline-rich protein BAG6	Bag6	mmu:224727				
2556	0.2632	0.4596	-0.1550	0.2047	0	P17710	Hexokinase;Hexokinase-1	Hk1					
2557	0.0970	-0.1402	0.1231	0.2054	0	P35123	Ubiquitin carboxyl-terminal hydrolase;Ubiquitin carboxyl-terminal hydrolase 4	Usp4	mmu:22258				
2558	0.1701	-1.1126	0.7211	0.2056	0	Q8R3H9	Tetratricopeptide repeat protein 4	Ttc4	mmu:72354				
2559	-0.1435	-0.0188	0.3024	0.2067	0	P70398	Ubiquitin carboxyl-terminal hydrolase;Probable ubiquitin carboxyl-terminal hydrolase FAF-X	Usp9x	mmu:22284				
2560	0.7682	-0.2785	0.2829	0.2085	0	P46978	Dolichyl-diphosphooligosaccharide--protein glycosyltransferase subunit STT3A	Stt3a	mmu:16430				
2561	0.1244	-0.1571	0.0500	0.2087	0	Q3TIV5	Zinc finger CCCH domain-containing protein 15	Zc3h15	mmu:69082				
2562	0.2551	-0.0769	0.0703	0.2096	0	P97311	DNA replication licensing factor MCM6;DNA helicase	Mcm6	mmu:17219				
2563	-0.3589	0.3592	-0.0782	0.2101	0	Q8C4Y3	Negative elongation factor B	Nelfb	mmu:58202				
2564	0.0689	0.5519	-0.2505	0.2101	0	Q8C2E4	Pentatricopeptide repeat-containing protein 1, mitochondrial	Ptcd1	mmu:71799				
2565	-0.0047	0.5970	-0.0109	0.2107	0	O35343	Importin subunit alpha-3	Kpna4	mmu:16649	1			
2566	-0.1473	0.5631	-0.1785	0.2109	0	Q8BX17	Gem-associated protein 5	Gemin5	mmu:216766				
2567	0.5183	-0.1823	-0.0040	0.2110	0	Q8K0V4	CCR4-NOT transcription complex subunit 3	Cnot3	mmu:232791				
2568	0.0193	0.9038	-0.0956	0.2111	0	P97333	Neuropilin-1	Nrp1	mmu:18186				
2569	-0.0900	-0.7502	1.1473	0.2115	0	Q9D6Y9	1,4-alpha-glucan-branching enzyme	Gbe1	mmu:74185				
2570	-0.1246	1.3397	0.5251	0.2117	0	F6Y616		Paip1					
2571	0.4083	0.3429	-0.5012	0.2124	0	O70439	Syntaxin-7	Stx7					
2572	-0.4861	0.2571	0.5048	0.2126	0	Q9D0L8	mRNA cap guanine-N7 methyltransferase	Rnmt	mmu:67897				
2573	0.2454	0.3632	-0.3954	0.2132	0	Q8C0I1	Alkyldihydroxyacetonephosphate synthase, peroxisomal	Agps	mmu:228061				
2574	-0.2109	-0.8084	0.6164	0.2144	0	Q4PZA2	Endothelin-converting enzyme 1	Ece1	mmu:230857				
2575	-0.2256	0.6156	-0.3254	0.2145	0	Q61749	Translation initiation factor eIF-2B subunit delta	Eif2b4	mmu:13667				
2576	0.1101	-0.2458	-0.2882	0.2147	0	Q9CQC8	Maspardin	Spg21	mmu:27965				
2577	-0.0641	0.0972	-0.0175	0.2148	0	Q62418	Drebrin-like protein	Dbnl	mmu:13169				
2578	-0.7193	-0.2953	0.3630	0.2158	0	Q8CI95	Oxysterol-binding protein;Oxysterol-binding protein-related protein 11	Osbpl11					
2579	0.6231	0.2081	-0.2091	0.2167	0	Q60605	Myosin light polypeptide 6	Myl6	mmu:17904				
2580	0.3652	0.0372	-0.4545	0.2168	0	Q3TDN2	FAS-associated factor 2	Faf2	mmu:76577				
2581	-0.2941	-0.5474	0.2573	0.2172	0	Q5NCJ0		Rapgef6					
2582	0.6300	-0.2587	0.3341	0.2174	0	Q6P8X1	Sorting nexin-6;Sorting nexin-6, N-terminally processed	Snx6	mmu:72183				
2583	0.0707	-0.3864	0.3635	0.2178	0	Q78HU7	Glycophorin-C	Gypc	mmu:71683				
2584	0.1747	-0.0850	0.2297	0.2179	0	P97452	Ribosome biogenesis protein BOP1	Bop1	mmu:12181				
2585	0.1022	-0.0570	-0.8928	0.2181	0	Q06138	Calcium-binding protein 39	Cab39	mmu:12283				
2586	0.9303	-0.0280	0.0798	0.2186	0	Q8C6E0	Cilia- and flagella-associated protein 36	Cfap36	mmu:216618				
2587	-0.1973	-0.2363	0.1090	0.2186	0	O08529	Calpain-2 catalytic subunit	Capn2	mmu:12334				
2588	-0.3269	-0.0259	0.3354	0.2189	0	Q5SW19	Clustered mitochondria protein homolog	Cluh	mmu:74148				
2589	-0.3721	0.5217	0.7257	0.2204	0	Q9D1L0	Coiled-coil-helix-coiled-coil-helix domain-containing protein 2	Chchd2;Zb	mmu:14004				
2590	-0.1050	0.9276	-0.0430	0.2207	0	Q8BZX4	Splicing regulatory glutamine/lysine-rich protein 1	Srek1	mmu:218543				
2591	0.0134	-0.4530	-0.3182	0.2218	0	O35658	Complement component 1 Q subcomponent-binding protein, mitochondrial	C1qbp					
2592	-0.0832	0.7865	0.2749	0.2228	0	O70475	UDP-glucose 6-dehydrogenase	Ugdh	mmu:22235				
2593	0.5229	-0.3527	0.4888	0.2237	0	O70591	Prefoldin subunit 2	Pfdn2	mmu:18637				
2594	0.0558	-0.1538	0.1163	0.2245	0	B2RXS4	Plexin-B2	Plxnb2	mmu:140570				
2595	-0.3421	0.3113	-0.1771	0.2250	0	O54734	Dolichyl-diphosphooligosaccharide--protein glycosyltransferase 48 kDa subunit	Ddost	mmu:13200				
2596	-0.0486	0.2402	-0.3686	0.2250	0	E9PVA8		Gcn1l1	mmu:231659				
2597	0.5043	0.4547	-0.0470	0.2251	0	Q8BH15	CCR4-NOT transcription complex subunit 10	Cnot10	mmu:78893				
2598	-0.6935	1.2502	0.1704	0.2256	0	P63028	Translationally-controlled tumor protein	Tpt1	mmu:22070				1
2599	0.2059	0.8520	-0.1806	0.2257	0	Q8CH25	SAFB-like transcription modulator	Sltm	mmu:66660				
2600	0.0788	-0.5655	-0.6281	0.2268	0	Q9CWI3	BRCA2 and CDKN1A-interacting protein	Bccip	mmu:66165				
2601	0.0213	0.8794	-1.0381	0.2268	0	P28659	CUGBP Elav-like family member 1	Celf1	mmu:13046				
2602	0.0075	0.1419	-0.4022	0.2269	0	Q9CQF9	Prenylcysteine oxidase	Pcyox1	mmu:66881				
2603	2.2196	-0.5814	0.7383	0.2273	0	Q9CR57	60S ribosomal protein L14	Rpl14	mmu:67115				
2604	-0.5244	0.1565	-0.0343	0.2275	0	Q8BU33	Acetolactate synthase-like protein	Ilvbl	mmu:216136				
2605	0.6904	-0.1249	[illegible]	0.2276	0	Q91W52	Transmembrane protein 19	Tmem19	mmu:67226				
2606	-0.1130	0.6538	-0.3409	0.2285	0	Q9WUD1	STIP1 homology and U box-containing protein 1	Stub1	mmu:56424			1	
2607	-0.1344	0.5707	0.3084	0.2308	0	Q8BTZ7	Mannose-1-phosphate guanyltransferase beta	Gmppb	mmu:331026				
2608	-0.1357	0.7198	0.9984	0.2319	0	P25976	Nucleolar transcription factor 1	Ubtf					
2609	-0.0720	0.4193	0.2504	0.2320	0	Q8R1A4	Dedicator of cytokinesis protein 7	Dock7					
2610	-0.0872	0.0437	-0.2471	0.2323	0	Q8CJ53	Cdc42-interacting protein 4	Trip10	mmu:106628				
2611	0.2465	0.7571	-0.2156	0.2323	0	Q6ZWY9	Histone H2B type 1-C/E/G;Histone H2B type 2-B;Histone H2B type 1-H;Histone H2B type 1-M	Hist1h2bc;	mmu:68024				
2612	-0.2336	-0.0061	0.3778	0.2332	0	G3X9X1		Kbtbd2	mmu:210973				
2613	0.0602	-0.2028	-0.0162	0.2341	0	P35564	Calnexin	Canx	mmu:12330				
2614	0.1455	-0.1747	0.3801	0.2351	0	Q69ZR2	E3 ubiquitin-protein ligase HECTD1	Hectd1				1	
2615	-0.3845	-0.0287	0.3393	0.2355	0	Q80UU9	Membrane-associated progesterone receptor component 2	Pgrmc2	mmu:70804				
2616	-0.0140	0.0292	0.1628	0.2369	0	O70435	Proteasome subunit alpha type-3	Psma3	mmu:19167				
2617	-0.0526	0.3379	0.2932	0.2374	0	Q9DBZ5	Eukaryotic translation initiation factor 3 subunit K	Eif3k	mmu:73830				
2618	0.2859	0.2384	-0.2077	0.2374	0	P28660	Nck-associated protein 1	Nckap1	mmu:50884				
2619	-0.3775	0.0394	-0.1331	0.2381	0	Q9ERK4	Exportin-2	Cse1l	mmu:110750				
2620	-0.2582	-0.7246	0.8448	0.2399	0	Q5SVR0	TBC1 domain family member 9B	Tbc1d9b	mmu:76795				
2621	0.1282	0.7318	-0.1396	0.2399	0	Q8K3A9	7SK snRNA methylphosphate capping enzyme	Mepce	mmu:231803				
2622	[illegible]	1.0389	0.0502	0.2406	0	Q91ZR2	Sorting nexin;Sorting nexin-18	Snx18					
2623	0.3173	0.6944	-0.3924	0.2417	0	Q9QWH1	Polyhomeotic-like protein 2	Phc2	mmu:54383				
2624	-0.8107	0.0924	0.2029	0.2420	0	Q8R422	CD109 antigen	Cd109	mmu:235505				
2625	-0.0344	0.0513	0.4314	0.2420	0	Q8VDD5	Myosin-9	Myh9	mmu:17886				
2626	0.3726	0.6765	-0.1474	0.2441	0	P13705	DNA mismatch repair protein Msh3	Msh3	mmu:17686				
2627	-1.1910	0.6355	0.2764	0.2459	0	O35239	Tyrosine-protein phosphatase non-receptor type 9	Ptpn9	mmu:56294		1		
2628	0.0942	0.0635	-0.0197	0.2461	0	Q69ZN7	Myoferlin	Myof	mmu:226101				
2629	-0.1838	0.9411	0.4848	0.2468	0	P54823	Probable ATP-dependent RNA helicase DDX6	Ddx6	mmu:13209				
2630	0.4783	-0.0930	0.0036	0.2472	0	Q64373	Bcl-2-like protein 1	Bcl2l1	mmu:12048				1
2631	0.1266	-0.7061	-0.0257	0.2487	0	F8VQ05		Fryl	mmu:72313				
2632	-0.0826	0.0640	-0.2781	0.2489	0	Q8VE97	Serine/arginine-rich splicing factor 4	Srsf4					
2633	0.0233	-0.5107	0.0409	0.2491	0	P26516	26S proteasome non-ATPase regulatory subunit 7	Psmd7	mmu:17463				
2634	0.3045	0.0218	-0.0982	0.2494	0	Q8BJY1	26S proteasome non-ATPase regulatory subunit 5	Psmd5	mmu:66998				
2635	-0.1047	0.1915	0.1574	0.2498	0	Q6Q899	Probable ATP-dependent RNA helicase DDX58	Ddx58	mmu:230073				

2636	0.0535	0.0645	-0.3662	0.2498	0	Q8VI75	Importin-4	Ipo4	mmu:75751				
2637	-0.4939	0.8130	1.0233	0.2498	0	Q8R0S1		Atf7					
2638	-0.3653	-0.4247	1.1945	0.2506	0	O35623	BET1 homolog	Bet1	mmu:12068				
2639	-0.3691	0.1054	0.6525	0.2506	0	Q99KN9	Clathrin interactor 1	Clint1					
2640	-0.5411	0.1022	0.8217	0.2521	0	D3YU17	Nicalin	Ncln					
2641	-0.0902	0.9374	0.0816	0.2521	0	O35226	26S proteasome non-ATPase regulatory subunit 4	Psmd4	mmu:19185				
2642	-0.0667	0.5249	-0.4002	0.2522	0	Q9D824	Pre-mRNA 3-end-processing factor FIP1	Fip1l1	mmu:66899				
2643	0.3617	-0.1985	0.3457	0.2524	0	Q9CXF4	TBC1 domain family member 15	Tbc1d15	mmu:66687				
2644	0.4673	0.5370	-0.5832	0.2533	0	Q9QXA5	U6 snRNA-associated Sm-like protein LSm4	Lsm4	mmu:50783				
2645	0.5495	-0.1592	-0.4043	0.2537	0	Q9CZP5	Mitochondrial chaperone BCS1	Bcs1l	mmu:66821				
2646	0.0712	-0.0597	0.1598	0.2544	0	Q3TIR3	Synembryn-A	Ric8a	mmu:101489				
2647	-0.2283	0.6786	0.4000	0.2548	0	P84104	Serine/arginine-rich splicing factor 3	Srsf3	mmu:20383				
2648	0.1976	-0.1434	0.7119	0.2556	0	Q6PB44	Tyrosine-protein phosphatase non-receptor type 23	Ptpn23	mmu:104831		1		
2649	-0.6029	0.8992	0.9915	0.2561	0	Q9QY36	N-alpha-acetyltransferase 10	Naa10	mmu:56292				
2650	0.1252	-0.0835	0.5114	0.2567	0	Q9D8U8	Sorting nexin-5	Snx5	mmu:69178				
2651	1.2410	-0.0106	0.4001	0.2571	0	Q6PFQ7	Ras GTPase-activating protein 4	Rasa4	mmu:54153				
2652	-0.0866	0.3396	0.4038	0.2571	0	Q9JJI8	60S ribosomal protein L38	Rpl38	mmu:67671				
2653	0.3984	-0.0350	-0.2157	0.2571	0	O09106	Histone deacetylase 1;Histone deacetylase	Hdac1;Gm	mmu:433759				1
2654	-0.6508	-0.2121	1.1724	0.2573	0	Q9D8T7	SRA stem-loop-interacting RNA-binding protein, mitochondrial	Slirp	mmu:380773				
2655	-0.4487	0.3216	0.2391	0.2577	0	P84078	ADP-ribosylation factor 1;ADP-ribosylation factor 3	Arf1;Arf3	mmu:11840				
2656	-0.1611	0.6151	0.8862	0.2583	0	Q3UIA2	Rho GTPase-activating protein 17	Arhgap17	mmu:70497				
2657	-0.0834	0.2247	-0.1807	0.2584	0	Q61235	Beta-2-syntrophin	Sntb2	mmu:20650				
2658	-0.1506	0.1881	-0.1333	0.2590	0	Q8R366	Immunoglobulin superfamily member 8	Igsf8	mmu:140559				
2659	0.0548	-0.3247	0.1110	0.2592	0	A2AWA9	Rab GTPase-activating protein 1	Rabgap1	mmu:227800				
2660	0.2719	0.3281	-0.0538	0.2600	0	Q8R1X6	Spartin	Spg20	mmu:229285				
2661	-0.2170	0.2708	0.0835	0.2600	0	O55029	Coatomer subunit beta	Copb2	mmu:50797				
2662	-0.4127	0.3043	-0.0289	0.2603	0	Q8BHF7	CDP-diacylglycerol--glycerol-3-phosphate 3-phosphatidyltransferase, mitochondrial	Pgs1	mmu:74451				
2663	-0.0688	-0.0461	0.6758	0.2605	0	B9EJ80		Pdzd8	mmu:107368				
2664	-0.1183	0.3403	0.4396	0.2618	0	P53986	Monocarboxylate transporter 1	Slc16a1	mmu:20501				
2665	-0.2243	-0.1318	0.3780	0.2620	0	P20108	Thioredoxin-dependent peroxide reductase, mitochondrial	Prdx3	mmu:11757	1			
2666	0.0864	0.2847	[illegible]	0.2624	0	Q8BX70	Vacuolar protein sorting-associated protein 13C	Vps13c	mmu:320528				
2667	0.6904	0.2089	-0.0473	0.2628	0	E9Q5K9	YTH domain-containing protein 1	Ythdc1	mmu:231386				
2668	-0.6096	-0.1754	0.5274	0.2630	0	Q9EPC1	Alpha-parvin	Parva	mmu:57342				
2669	0.3709	0.0716	-0.0499	0.2647	0	Q6NVF4	DNA helicase B	Helb	mmu:117599				
2670	0.6118	-0.3258	0.0426	0.2651	0	Q8R050	Eukaryotic peptide chain release factor GTP-binding subunit ERF3A	Gspt1	mmu:14852				
2671	0.9329	-0.2593	-0.1545	0.2664	0	Q70FJ1	A-kinase anchor protein 9	Akap9	mmu:100986				
2672	-0.0767	0.3900	-0.0599	0.2685	0	P62071	Ras-related protein R-Ras2	Rras2	mmu:66922				
2673	0.0477	0.7438	-0.5941	0.2691	0	Q5SF07	Insulin-like growth factor 2 mRNA-binding protein 2	Igf2bp2	mmu:319765				
2674	-0.4060	0.3065	0.0541	0.2701	0	Q921F4	Heterogeneous nuclear ribonucleoprotein L-like	Hnrnpll	mmu:72692				
2675	0.5364	0.0655	-0.1884	0.2706	0	Q2YDW2	Protein misato homolog 1	Msto1	mmu:229524				
2676	-0.2550	-0.6693	0.1948	0.2712	0	P63011	Ras-related protein Rab-3A	Rab3a	mmu:19339				
2677	0.6676	-0.3700	0.4502	0.2717	0	Q3TX08	tRNA (guanine(26)-N(2))-dimethyltransferase	Trmt1	mmu:212528				
2678	0.2321	0.1046	-0.5650	0.2719	0	Q9D880	Mitochondrial import inner membrane translocase subunit TIM50	Timm50	mmu:66525		1		
2679	0.3598	-0.1899	0.0145	0.2722	0	Q8C3I8	Protein HGH1 homolog	Hgh1	mmu:59053				
2680	-0.2871	0.6349	0.5800	0.2726	0	Q8R3V5	Endophilin-B2	Sh3glb2	mmu:227700				
2681	0.7117	0.0316	-0.1185	0.2728	0	O35643	AP-1 complex subunit beta-1	Ap1b1	mmu:11764				
2682	0.3034	0.1438	-0.2210	0.2730	0	O08784	Treacle protein	Tcof1	mmu:21453				
2683	0.4981	-0.0086	-0.3962	0.2731	0	P19426	Negative elongation factor E	Nelfe	mmu:27632				
2684	-0.3307	0.1704	-0.0747	0.2735	0	Q9D8V0	Minor histocompatibility antigen H13	H13;Hm13	mmu:14950				
2685	0.3241	-0.1541	0.1347	0.2738	0	O88544	COP9 signalosome complex subunit 4	Cops4	mmu:26891				
2686	0.0071	1.8652	-0.7218	0.2743	0	Q5XG71	Small subunit processome component 20 homolog	Utp20					
2687	-0.8857	-0.0597	0.9791	0.2749	0	Q921G8	Gamma-tubulin complex component 2	Tubgcp2	mmu:74237				
2688	-0.2082	0.5800	0.1588	0.2752	0	Q3UYV9	Nuclear cap-binding protein subunit 1	Ncbp1	mmu:433702				
2689	-0.4060	0.0574	0.2562	0.2753	0	Q9JKC8	AP-3 complex subunit mu-1	Ap3m1	mmu:55946				
2690	0.0995	0.2577	-0.0907	0.2761	0	P11688	Integrin alpha-5;Integrin alpha-5 heavy chain;Integrin alpha-5 light chain	Itga5	mmu:16402				
2691	1.0370	0.7187	-0.1180	0.2762	0	Q8K0B2	Probable lysosomal cobalamin transporter	Lmbrd1	mmu:68421				
2692	-0.3690	0.8386	0.1785	0.2764	0	Q8BG67	Protein EFR3 homolog A	Efr3a	mmu:76740				
2693	0.8546	0.1343	-0.1996	0.2774	0	Q921Q7	Ras and Rab interactor 1	Rin1	mmu:225870				
2694	-0.1105	0.5662	-0.0064	0.2778	0	Q99PP7	E3 ubiquitin-protein ligase TRIM33	Trim33	mmu:94093			1	
2695	1.9507	0.7324	-0.9296	0.2791	0	Q8BIH0	Histone deacetylase complex subunit SAP130	Sap130	mmu:269003				
2696	-0.2582	0.2267	-0.3510	0.2800	0	P51150	Ras-related protein Rab-7a	Rab7a	mmu:19349				
2697	0.3874	0.4049	-0.1783	0.2805	0	Q99LD4	COP9 signalosome complex subunit 1	Gps1					
2698	0.1662	0.0367	-0.0198	0.2813	0	Q9QUM9	Proteasome subunit alpha type-6	Psma6	mmu:26443				
2699	-0.3957	0.0705	0.4892	0.2814	0	O89079	Coatomer subunit epsilon	Cope	mmu:59042				
2700	-0.3975	0.3484	0.2685	0.2815	0	E9QAE3		Btaf1	mmu:107182				
2701	0.0621	0.9271	-0.0869	0.2819	0	P97450	ATP synthase-coupling factor 6, mitochondrial	Atp5j	mmu:11957				
2702	0.4731	-0.0809	0.3581	0.2827	0	Q8CCB4	Vacuolar protein sorting-associated protein 53 homolog	Vps53	mmu:68299				
2703	0.4853	-0.0840	0.1069	0.2827	0	Q922D4	Serine/threonine-protein phosphatase 6 regulatory subunit 3	Ppp6r3	mmu:52036				
2704	-0.0823	0.1936	0.4433	0.2834	0	Q8C129	Leucyl-cystinyl aminopeptidase	Lnpep	mmu:240028				
2705	-1.3410	1.0147	1.1190	0.2841	0	Q80T85	DDB1- and CUL4-associated factor 5	Dcaf5	mmu:320808			1	
2706	0.3017	0.0137	-0.0836	0.2844	0	G3X9K3	Brefeldin A-inhibited guanine nucleotide-exchange protein 1	Arfgef1	mmu:211673				
2707	-0.1963	0.9388	0.5508	0.2848	0	P45700	Mannosyl-oligosaccharide 1,2-alpha-mannosidase IA;alpha-1,2-Mannosidase	Man1a1;M	mmu:17155				
2708	0.3699	-0.5730	0.1874	0.2850	0	B9EKI3	TATA element modulatory factor	Tmf1	mmu:232286				
2709	-0.5037	-0.1487	0.1340	0.2851	0	Q8C547	HEAT repeat-containing protein 5B	Heatr5b	mmu:320473				
2710	0.1123	-0.1023	0.6660	0.2854	0	Q5SNZ0	Girdin	Ccdc88a	mmu:108686				
2711	0.8859	0.2189	-0.5495	0.2855	0	Q99N94	39S ribosomal protein L9, mitochondrial	Mrpl9	mmu:78523				
2712	0.8192	0.2880	-0.1080	0.2867	0	P09926	Surfeit locus protein 2	Surf2	mmu:20931				
2713	1.3100	-0.1981	-0.0385	0.2869	0	Q6DFX2	Anthrax toxin receptor 2	Antxr2	mmu:71914				
2714	0.4269	0.2244	-0.1474	0.2870	0	Q6NVF9	Cleavage and polyadenylation specificity factor subunit 6	Cpsf6	mmu:432508				
2715	-0.1275	0.1406	0.2486	0.2871	0	Q7TQ95	Protein lunapark	Lnp	mmu:69605				
2716	-0.0552	-0.9182	0.3723	0.2873	0	Q9DBS2	Tumor protein p63-regulated gene 1-like protein	Tprg1l;Tpr	mmu:67808				
2717	1.8015	1.3528	-0.4356	0.2877	0	Q9DB34	Charged multivesicular body protein 2a	Chmp2a	mmu:68953				
2718	0.0271	0.3459	-0.2202	0.2889	0	Q9JHE7	Protein TSSC4	Tssc4	mmu:56844				
2719	0.2207	0.9925	-0.3531	0.2900	0	Q60596	DNA repair protein XRCC1	Xrcc1	mmu:22594				
2720	0.4163	-0.0642	0.1397	0.2901	0	O35593	26S proteasome non-ATPase regulatory subunit 14	Psmd14	mmu:59029				
2721	0.7900	-0.1060	0.6714	0.2906	0	G5E8R2		Tpm1					
2722	-0.0219	1.1086	-0.5760	0.2907	0	Q60996	Serine/threonine-protein phosphatase 2A 56 kDa regulatory subunit gamma isoform	Ppp2r5c	mmu:26931				
2723	-1.1581	0.3635	-0.3532	0.2908	0	P55258	Ras-related protein Rab-8A	Rab8a	mmu:17274				
2724	-0.4552	0.1821	-0.1246	0.2911	0	Q8C878	NEDD8-activating enzyme E1 catalytic subunit	Uba3	mmu:22200				
2725	0.5615	0.3083	-0.1653	0.2914	0	Q9QXD8	LIM domain-containing protein 1	Limd1	mmu:29806				
2726	-0.3386	-0.8164	0.3478	0.2917	0	Q8BWP8	Beta-1,4-glucuronyltransferase 1	B4gat1	mmu:108902				
2727	-0.8030	0.6658	0.5536	0.2918	0	Q9DBP5	UMP-CMP kinase	Cmpk1	mmu:66588				
2728	-0.0667	-0.0903	0.4443	0.2950	0	O54825	Bystin	Bysl	mmu:53414				
2729	-0.1022	0.9923	0.0702	0.2958	0	Q8BP00	IQ calmodulin-binding motif-containing protein 1	Iqcb1	mmu:320299				
2730	0.2879	0.6705	-0.5737	0.2959	0	Q62186	Translocon-associated protein subunit delta	Ssr4	mmu:20832				
2731	-0.1223	0.7332	-0.1485	0.2965	0	Q6GQT9	Nodal modulator 1	Nomo1	mmu:211548				
2732	-0.2756	0.3804	0.9150	0.2979	0	Q9CQL7	MORF4 family-associated protein 1	Mrfap1;Gn	mmu:67568				
2733	[illegible]	-0.3303	-0.1305	0.2985	0	Q9WU00	Nuclear respiratory factor 1	Nrf1	mmu:18181				
2734	-0.9101	0.9387	0.7886	0.2985	0	Q7TSI3	Serine/threonine-protein phosphatase 6 regulatory subunit 1	Ppp6r1	mmu:243819				
2735	0.0938	0.4434	-0.2336	0.2996	0	P06795	Multidrug resistance protein 1B;Multidrug resistance protein 1A	Abcb1b;Ab	mmu:18669				
2736	0.5582	1.4563	-0.1479	0.3000	0	Q9R1K9	Centrin-2	Cetn2	mmu:26370				
2737	-0.0209	0.8785	-0.1248	0.3008	0	P62996	Transformer-2 protein homolog beta	Tra2b	mmu:20462	1			
2738	0.2869	-0.0585	-0.6199	0.3011	0	P70168	Importin subunit beta-1	Kpnb1	mmu:16211				
2739	0.2853	0.0159	-0.1478	0.3016	0	Q66GT5	Phosphatidylglycerophosphatase and protein-tyrosine phosphatase 1	Ptpmt1	mmu:66461		1		
2740	-0.2348	0.1879	-0.2670	0.3027	0	Q9EPK7	Exportin-7	Xpo7	mmu:65246				
2741	0.2781	-0.1091	0.4325	0.3028	0	Q91VI7	Ribonuclease inhibitor	Rnh1	mmu:107702				
2742	-0.1421	0.1300	0.4950	0.3051	0	Q01405	Protein transport protein Sec23A	Sec23a	mmu:20334				
2743	0.3452	-0.4381	1.1324	0.3066	0	Q8R5C5	Beta-centractin	Actr1b	mmu:226977				
2744	-0.4787	0.7569	[illegible]	0.3069	0	P62315	Small nuclear ribonucleoprotein Sm D1	Snrpd1	mmu:20641				
2745	0.0204	-0.0393	0.7149	0.3072	0	Q3UHD6	Sorting nexin-27	Snx27	mmu:76742				
2746	-0.1812	-0.0904	0.3195	0.3075	0	Q99M87	DnaJ homolog subfamily A member 3, mitochondrial	Dnaja3	mmu:83945				
2747	0.2779	0.6225	-0.2025	0.3080	0	P61924	Coatomer subunit zeta-1	Copz1	mmu:56447				
2748	0.1339	0.2497	-0.3157	0.3106	0	Q922Y2	Tripartite motif-containing protein 59	Trim59	mmu:66949				
2749	0.2436	0.7561	-0.6356	0.3109	0	Q99JN2	Kelch-like protein 22	Klhl22	mmu:224023			1	
2750	0.1581	-0.1470	0.3373	0.3110	0	P63242	Eukaryotic translation initiation factor 5A;Eukaryotic translation initiation factor 5A-1	Eif5a	mmu:276770				
2751	0.2796	0.1674	-0.0364	0.3113	0	A2AN08	E3 ubiquitin-protein ligase UBR4	Ubr4	mmu:69116			1	
2752	-0.1376	0.2855	0.2977	0.3122	0	Q6P5G6	UBX domain-containing protein 7	Ubxn7					
2753	-0.5295	0.7237	0.5451	0.3124	0	Q6DID3	Protein SCAF8	Scaf8	mmu:106583				
2754	0.2544	-0.2479	0.4946	0.3125	0	Q9D0D5	General transcription factor IIE subunit 1	Gtf2e1	mmu:74197				
2755	-0.1183	0.1323	-0.6704	0.3125	0	Q9Z0R9	Fatty acid desaturase 2	Fads2	mmu:56473				
2756	0.4117	0.4756	-0.1778	0.3130	0	Q640N1	Adipocyte enhancer-binding protein 1	Aebp1	mmu:11568				
2757	-0.8693	0.3594	0.1008	0.3148	0	Q8BJU2	Tetraspanin;Tetraspanin-9	Tspan9	mmu:109246				
2758	-0.0880	-0.4803	0.6172	0.3155	0	Q3TZX8	Polynucleotide 5-hydroxyl-kinase NOL9	Nol9	mmu:74035				
2759	1.2093	-0.4043	0.0762	0.3170	0	Q9D7A8	Armadillo repeat-containing protein 1	Armc1	mmu:74252				
2760	0.5821	0.1446	-0.4269	0.3185	0	Q9JK91	DNA mismatch repair protein Mlh1	Mlh1	mmu:17350				
2761	1.3304	0.8565	-1.1345	0.3195	0	Q9QY73	Transmembrane protein 59	Tmem59	mmu:56374				

2762	0.2867	[illegible]	0.9577	0.3196	0	Q8K012	Formin-binding protein 1-like	Fnbp1l					
2763	[illegible]	2.4139	0.9034	0.3199	0	Q9D1M0	Protein SEC13 homolog	Sec13	mmu:110379				
2764	-1.0672	0.2345	0.0655	0.3211	0	A8Y5H7		Sec14l1	mmu:74136				
2765	-0.0475	0.2698	-0.0371	0.3226	0	Q80WJ7	Protein LYRIC	Mtdh	mmu:67154				
2766	0.6446	0.1818	-0.1569	0.3230	0	O88543	COP9 signalosome complex subunit 3	Cops3	mmu:26572				
2767	-0.0760	0.8552	0.4154	0.3234	0	Q52KI8	Serine/arginine repetitive matrix protein 1	Srrm1	mmu:51796				
2768	-0.0984	0.9889	0.3704	0.3242	0	Q9DBC3	Cap-specific mRNA (nucleoside-2-O-)-methyltransferase 1	Cmtr1	mmu:74157				
2769	0.4106	-0.3633	0.3677	0.3247	0	Q9D662	Protein transport protein Sec23B	Sec23b	mmu:27054				
2770	0.0952	0.6530	-0.5036	0.3253	0	P97287	Induced myeloid leukemia cell differentiation protein Mcl-1 homolog	Mcl1	mmu:17210				1
2771	0.4240	0.5886	-0.1434	0.3261	0	Q9CQU1	Microfibrillar-associated protein 1	Mfap1	mmu:67532				
2772	0.5143	0.3779	-0.0834	0.3273	0	Q6ZWZ2	Ubiquitin-conjugating enzyme E2 R2	Ube2r2	mmu:67615			1	
2773	-0.0918	0.0552	-1.0133	0.3280	0	Q9JIG8	PRA1 family protein 2	Praf2	mmu:54637				
2774	0.6849	0.0960	-0.4944	0.3311	0	P61022	Calcineurin B homologous protein 1	Chp1	mmu:56398				
2775	0.6063	-0.4987	-0.7719	0.3313	0	E9Q9B7		Kidins220	mmu:77480				
2776	-0.2535	0.8086	0.0321	0.3322	0	Q60838	Segment polarity protein dishevelled homolog DVL-2	Dvl2	mmu:13543				
2777	0.2046	-0.3469	0.3137	0.3323	0	Q8CFI2	Ubiquitin-conjugating enzyme E2 R1	Cdc34	mmu:216150			1	
2778	0.0649	0.8784	-0.3551	0.3323	0	P97379	Ras GTPase-activating protein-binding protein 2	G3bp2	mmu:23881				
2779	0.4341	1.0238	-0.7492	0.3333	0	Q923B1	Lariat debranching enzyme	Dbr1	mmu:83703				
2780	0.3989	0.0127	-0.3246	0.3337	0	Q8C407	Protein YIPF4	Yipf4	mmu:67864				
2781	-0.3556	0.0723	0.3847	0.3341	0	P49586	Choline-phosphate cytidylyltransferase A	Pcyt1a	mmu:13026				
2782	-0.2253	-0.1231	0.2146	0.3343	0	O35598	Disintegrin and metalloproteinase domain-containing protein 10	Adam10	mmu:11487				
2783	0.0940	-0.5610	-0.5907	0.3347	0	Q9D071	MMS19 nucleotide excision repair protein homolog	Mms19	mmu:72199				
2784	-0.6268	1.2601	0.3447	0.3352	0	P62897	Cytochrome c, somatic	Cycs	mmu:13063	1			1
2785	-0.1030	-0.1838	0.0967	0.3357	0	P05213	Tubulin alpha-1B chain	Tuba1b	mmu:22143				
2786	0.2166	-0.2815	1.2709	0.3374	0	P57080	Ubiquitin carboxyl-terminal hydrolase 25	Usp25	mmu:30940				
2787	0.0988	-0.1789	-0.3033	0.3377	0	O88696	ATP-dependent Clp protease proteolytic subunit, mitochondrial	Clpp	mmu:53895				
2788	0.1640	2.2572	-0.6199	0.3379	0	Q8BKT7	THO complex subunit 5 homolog	Thoc5	mmu:107829				
2789	1.0260	0.5891	-0.6875	0.3384	0	P70257	Nuclear factor 1;Nuclear factor 1 X-type	Nfix	mmu:18032				
2790	-0.3874	0.9877	0.3376	0.3398	0	P11881	Inositol 1,4,5-trisphosphate receptor type 1	Itpr1	mmu:16438				
2791	-0.8083	0.4324	-0.2158	0.3405	0	Q00422	GA-binding protein alpha chain	Gabpa	mmu:14390				
2792	-0.1153	0.1501	0.3825	0.3408	0	P10923	Osteopontin	Spp1	mmu:20750				1
2793	0.2195	-0.0177	-0.1431	0.3414	0	Q9D379	Epoxide hydrolase 1	Ephx1	mmu:13849				
2794	-0.2768	0.1019	-0.8409	0.3420	0	Q9Z0L0	Trophoblast glycoprotein	Tpbg	mmu:21983				
2795	-0.0101	0.5065	-0.1237	0.3447	0	Q60865	Caprin-1	Caprin1	mmu:53872				
2796	0.4932	-0.4326	0.1601	0.3455	0	O89110	Caspase-8;Caspase-8 subunit p18;Caspase-8 subunit p10	Casp8	mmu:12370				1
2797	-0.4899	-0.5889	3.2216	0.3462	0	O09172	Glutamate--cysteine ligase regulatory subunit	Gclm	mmu:14630	1			
2798	-0.8924	0.7071	0.4306	0.3465	0	Q91YW3	DnaJ homolog subfamily C member 3	Dnajc3	mmu:100037258				
2799	-0.7009	0.6660	-0.2347	0.3469	0	P60904	DnaJ homolog subfamily C member 5	Dnajc5	mmu:13002				
2800	-0.0066	0.2688	-0.1544	0.3478	0	B2RRE7	OTU domain-containing protein 4	Otud4	mmu:73945				
2801	0.0784	0.3331	-0.0610	0.3483	0	P48453	Serine/threonine-protein phosphatase;Serine/threonine-protein phosphatase 2B catalytic subunit	Ppp3cb	mmu:19056		1		
2802	0.7291	0.1549	-0.1521	0.3491	0	Q8BX02	KN motif and ankyrin repeat domain-containing protein 2	Kank2	mmu:235041				
2803	0.3095	0.0554	-0.0494	0.3520	0	Q6ZQ38	Cullin-associated NEDD8-dissociated protein 1	Cand1	mmu:71902				
2804	-0.2968	0.8599	-0.8060	0.3531	0	Q8BLN5	Lanosterol synthase	Lss	mmu:16987				
2805	-1.2000	-0.2611	0.1527	0.3535	0	Q80TL7	Protein MON2 homolog	Mon2	mmu:67074				
2806	-0.2726	0.7816	-0.4394	0.3559	0	Q9CQE3	28S ribosomal protein S17, mitochondrial	Mrps17	mmu:66258				
2807	-0.6478	0.7771	0.9916	0.3560	0	Q9CQX8	28S ribosomal protein S36, mitochondrial	Mrps36	mmu:66128				
2808	-0.3064	-0.0709	0.0580	0.3567	0	Q8CCP0	Nuclear export mediator factor Nemf	Nemf	mmu:66244				
2809	-0.1432	0.0948	-0.8166	0.3573	0	Q9CQN7	39S ribosomal protein L41, mitochondrial	Mrpl41	mmu:107733				
2810	0.2779	-0.3694	0.3814	0.3587	0	Q6ZQH8	Nucleoporin NUP188 homolog	Nup188	mmu:227699				
2811	-0.2275	0.7068	0.1207	0.3589	0	P51125	Calpastatin	Cast	mmu:12380				
2812	-0.2571	-0.0679	0.1273	0.3601	0	Q8BY71	Histone acetyltransferase type B catalytic subunit	Hat1	mmu:107435				
2813	-0.2175	-0.0707	0.5803	0.3612	0	Q8VBT0	Thioredoxin-related transmembrane protein 1	Tmx1	mmu:72736				1
2814	-0.2797	0.1055	-0.2337	0.3618	0	Q61739	Integrin alpha-6;Integrin alpha-6 heavy chain;Integrin alpha-6 light chain	Itga6	mmu:16403				
2815	0.9766	0.2274	-0.1982	0.3631	0	Q91XC0	LIM domain-containing protein ajuba	Ajuba	mmu:16475				
2816	-0.3381	-0.1053	0.0660	0.3645	0	O70311	Glycylpeptide N-tetradecanoyltransferase 2;Glycylpeptide N-tetradecanoyltransferase	Nmt2	mmu:18108				
2817	-0.3998	0.9584	0.6358	0.3654	0	Q4VAA2	Protein CDV3	Cdv3	mmu:321022				
2818	0.2188	-0.1007	0.3207	0.3658	0	Q8BRN9	Coiled-coil and C2 domain-containing protein 1B	Cc2d1b	mmu:319965				
2819	-0.1306	0.4719	-0.3833	0.3663	0	Q99PU8	Putative ATP-dependent RNA helicase DHX30	Dhx30	mmu:72831				
2820	-2.3229	0.8747	-0.0626	0.3663	0	Q62086	Serum paraoxonase/arylesterase 2	Pon2	mmu:330260	1			
2821	0.1824	-0.0402	0.0495	0.3665	0	Q8VDQ8	NAD-dependent protein deacetylase sirtuin-2	Sirt2	mmu:64383	1			
2822	0.5052	-0.1055	0.2657	0.3667	0	Q9WV92	Band 4,1-like protein 3;Band 4,1-like protein 3, N-terminally processed	Epb41l3	mmu:13823				
2823	0.0823	-0.8156	0.8390	0.3672	0	Q62422	Osteoclast-stimulating factor 1	Ostf1	mmu:20409				
2824	1.0789	0.1895	-0.6527	0.3685	0	Q80U95	Ubiquitin-protein ligase E3C	Ube3c	mmu:100763			1	
2825	-0.5132	0.0539	1.8662	0.3689	0	Q9Z199	Transcription elongation factor SPT4-B;Transcription elongation factor SPT4-A	Supt4h1b;	mmu:100041294				
2826	-0.0380	0.2576	-0.2023	0.3691	0	Q61411	GTPase HRas;GTPase HRas, N-terminally processed	Hras	mmu:15461				
2827	-0.1388	1.4182	-0.0506	0.3705	0	Q9CWV6	PRKR-interacting protein 1	Prkrip1	mmu:66801				
2828	0.6382	-0.0058	0.6978	0.3721	0	Q5NCR9	Nuclear speckle splicing regulatory protein 1	Nsrp1	mmu:237859				
2829	0.2229	0.7344	-0.1663	0.3740	0	Q9DCG9	Multifunctional methyltransferase subunit TRM112-like protein	Trmt112	mmu:67674				
2830	0.4346	0.4972	-1.0050	0.3755	0	Q4VBE8	WD repeat-containing protein 18	Wdr18	mmu:216156				
2831	0.5054	-0.6244	-0.0255	0.3758	0	P17439	Glucosylceramidase	Gba	mmu:14466				
2832	-1.1106	-0.2215	0.2542	0.3763	0	A6H8H2	DENN domain-containing protein 4C	Dennd4c	mmu:329877				
2833	0.6168	0.5032	-1.1540	0.3765	0	Q04692	SWI/SNF-related matrix-associated actin-dependent regulator of chromatin subfamily A containing	Smarcad1	mmu:13990				
2834	0.1485	-0.1503	0.5730	0.3768	0	Q9DB05	Alpha-soluble NSF attachment protein	Napa	mmu:108124				
2835	0.5505	1.1051	-0.9441	0.3769	0	Q8CBY3	Leukocyte receptor cluster member 8 homolog	Leng8	mmu:232798				
2836	0.2401	1.6121	-0.2165	0.3771	0	A2RSJ4	UHRF1-binding protein 1-like	Uhrf1bp1l	mmu:75089				
2837	0.4047	0.8326	-0.2441	0.3771	0	Q9DB90	Protein SMG9	Smg9	mmu:71997				
2838	0.3643	-0.0044	-0.1615	0.3779	0	P68372	Tubulin beta-4B chain;Tubulin beta-4A chain	Tubb4b;Tu	mmu:227613				
2839	0.4067	0.2857	-0.1823	0.3793	0	Q8BGK6	Y+L amino acid transporter 2	Slc7a6	mmu:330836				
2840	0.2140	0.6408	-0.1294	0.3807	0	Q9Z0H3	SWI/SNF-related matrix-associated actin-dependent regulator of chromatin subfamily B member 1	Smarcb1	mmu:20587				
2841	0.7825	-0.1736	-0.1452	0.3842	0	Q64008	Ras-related protein Rab-34	Rab34	mmu:19376				
2842	0.8340	0.5870	-0.1296	0.3847	0	Q9ET30	Transmembrane 9 superfamily member 3	Tm9sf3	mmu:107358				
2843	0.1674	-0.5377	-0.3639	0.3850	0	Q8R0A0	General transcription factor IIF subunit 2	Gtf2f2	mmu:68705				
2844	-0.2329	-0.1423	0.0360	0.3869	0	A2A5R2	Brefeldin A-inhibited guanine nucleotide-exchange protein 2	Arfgef2	mmu:99371				
2845	0.3160	-0.1806	-0.1382	0.3879	0	Q9CXG3	Peptidyl-prolyl cis-trans isomerase-like 4	Ppil4	mmu:67418				
2846	-0.0599	0.0871	0.1671	0.3883	0	P99024	Tubulin beta-5 chain	Tubb5	mmu:22154				
2847	-0.3515	-0.1349	0.8905	0.3902	0	Q91WQ3	Tyrosine--tRNA ligase;Tyrosine--tRNA ligase, cytoplasmic;Tyrosine--tRNA ligase, cytoplasmic, N-ter	Yars	mmu:107271				
2848	1.2392	0.4518	-0.1684	0.3912	0	Q8CIG8	Protein arginine N-methyltransferase 5	Prmt5	mmu:27374				
2849	0.0359	0.8417	-0.2571	0.3918	0	Q8R480	Nuclear pore complex protein Nup85	Nup85	mmu:445007				
2850	0.2262	[illegible]	-0.2206	0.3920	0	Q99LC3	NADH dehydrogenase [ubiquinone] 1 alpha subcomplex subunit 10, mitochondrial	Ndufa10	mmu:67273				
2851	-0.3433	0.7505	-0.2373	0.3936	0	Q9CQF3	Cleavage and polyadenylation specificity factor subunit 5	Nudt21	mmu:68219				
2852	0.0398	0.2455	-0.0173	0.3939	0	O54984	ATPase Asna1	Asna1	mmu:56495				
2853	-0.3167	0.1833	0.1695	0.3960	0	Q3UM29	Conserved oligomeric Golgi complex subunit 7	Cog7	mmu:233824				
2854	0.4834	0.8375	-0.9661	0.3982	0	Q3TVC7	Cyclin-D1-binding protein 1	Ccndbp1	mmu:17151				
2855	-0.1625	0.3788	0.9302	0.3991	0	P51859	Hepatoma-derived growth factor	Hdgf	mmu:15191				
2856	2.3727	-0.1877	-0.5287	0.4011	0	Q9WUZ9	Ectonucleoside triphosphate diphosphohydrolase 5	Entpd5	mmu:12499				
2857	-0.6142	0.0236	-0.1277	0.4026	0	Q8BG51	Mitochondrial Rho GTPase 1	Rhot1	mmu:59040				
2858	-0.2484	0.9199	-0.2989	0.4036	0	Q6VNB8	WD repeat and FYVE domain-containing protein 3	Wdfy3	mmu:72145				
2859	-0.1245	2.2687	0.5633	0.4048	0	P23949	Zinc finger protein 36, C3H1 type-like 2	Zfp36l2	mmu:12193				
2860	0.2535	-0.0548	0.1464	0.4050	0	Q8BQM4	HEAT repeat-containing protein 3	Heatr3	mmu:234549				
2861	-0.1350	0.8694	0.2442	0.4057	0	O35326	Serine/arginine-rich splicing factor 5	Srsf5	mmu:20384				
2862	0.1874	0.2539	-0.0049	0.4058	0	Q9JI39	ATP-binding cassette sub-family B member 10, mitochondrial	Abcb10	mmu:56199				
2863	0.1325	0.3954	-0.2399	0.4090	0	Q01768	Nucleoside diphosphate kinase;Nucleoside diphosphate kinase B;Nucleoside diphosphate kinase A	Gm20390;	mmu:18103	1			
2864	0.8639	-0.5385	1.3166	0.4096	0	O88448	Kinesin light chain 2	Klc2					
2865	1.0127	-1.4588	1.1679	0.4099	0	P62204	Calmodulin	Calm1	mmu:12313				
2866	0.1927	0.0328	-0.0245	0.4103	0	Q8BHN3	Neutral alpha-glucosidase AB	Ganab	mmu:14376				
2867	0.0302	2.2462	-0.0057	0.4117	0	Q8K2F0	Bromodomain-containing protein 3	Brd3	mmu:67382				
2868	0.7498	0.2641	-0.1603	0.4131	0	Q9EQQ9	Protein O-GlcNAcase	Mgea5	mmu:76055				
2869	0.5089	0.3229	-0.0207	0.4134	0	Q8BTT6	Digestive organ expansion factor homolog	Diexf	mmu:215193				
2870	-0.1088	-0.2968	0.6059	0.4168	0	Q91VR2	ATP synthase subunit gamma;ATP synthase subunit gamma, mitochondrial	Atp5c1	mmu:11949				
2871	-0.8346	0.4075	-0.0731	0.4197	0	P11103	Poly [ADP-ribose] polymerase 1	Parp1		1			
2872	-0.0940	1.7095	0.1814	0.4199	0	Q8CIV2	Membralin	Tmem259	mmu:216157				
2873	0.7152	0.7724	-0.4574	0.4204	0	Q9CY97	RNA polymerase II subunit A C-terminal domain phosphatase SSU72	Ssu72	mmu:68991		1		
2874	0.0224	0.2257	-0.1339	0.4226	0	Q922F4	Tubulin beta-6 chain	Tubb6	mmu:67951				
2875	1.5690	0.0076	-0.1545	0.4227	0	Q8K4Q0	Regulatory-associated protein of mTOR	Rptor					
2876	-0.2579	0.3794	0.0841	0.4230	0	P01895	H-2 class I histocompatibility antigen, D-D alpha chain	H2-D1					
2877	-0.1168	0.4730	-0.2972	0.4231	0	E9Q4X2		Uggt2	mmu:66435				
2878	-0.5777	0.0873	-0.0472	0.4232	0	Q9CY94	DNA replication complex GINS protein PSF3	Gins3	mmu:78833				
2879	1.3235	-0.1523	-0.2069	0.4241	0	Q8R3P7	Clusterin-associated protein 1	Cluap1	mmu:76779				
2880	-1.0375	2.0068	-1.4745	0.4246	0	Q80W00	Serine/threonine-protein phosphatase 1 regulatory subunit 10	Ppp1r10	mmu:52040				
2881	-0.1266	0.6197	-0.2780	0.4270	0	Q9D6U8	Protein FAM162A	Fam162a	mmu:70186				
2882	-0.1157	0.3659	0.2422	0.4301	0	P40336	Vacuolar protein sorting-associated protein 26A	Vps26a	mmu:30930				
2883	-1.3050	0.1490	1.9499	0.4336	0	P28574	Protein max	Max					
2884	-0.7477	0.8180	0.2529	0.4347	0	Q91VH6	Protein MEMO1	Memo1	mmu:76890				
2885	0.2498	0.1708	-1.0070	0.4351	0	P68033	Actin, alpha cardiac muscle 1;Actin, gamma-enteric smooth muscle;Actin, alpha skeletal muscle;Ac	Actc1;Actg	mmu:11464				
2886	0.6354	0.5272	-0.2865	0.4364	0	Q6PDX6	E3 ubiquitin-protein ligase Rnf220	Rnf220	mmu:66743			1	
2887	0.5315	0.1424	-0.1487	0.4365	0	Q8VBZ3	Cleft lip and palate transmembrane protein 1 homolog	Clptm1	mmu:56457				

2888	-0.1142	0.3378	0.1709	0.4392	0	Q9WTX8	Mitotic spindle assembly checkpoint protein MAD1	Mad1l1	mmu:17120				
2889	1.4705	-0.8917	-0.2620	0.4401	0	A2BE28	Ribosomal biogenesis protein LAS1L	Las1l	mmu:76130				
2890	-0.4499	0.7254	0.4810	0.4411	0	Q8CH72	E3 ubiquitin-protein ligase TRIM32	Trim32	mmu:69807			1	
2891	-1.4628	0.3534	3.6455	0.4414	0	Q8K2T1	NmrA-like family domain-containing protein 1	Nmral1	mmu:67824				
2892	-0.5806	1.3909	-0.3337	0.4417	0	Q3TRM4	Neuropathy target esterase	Pnpla6	mmu:50767				
2893	0.6429	-0.1856	-0.2527	0.4432	0	P12367	cAMP-dependent protein kinase type II-alpha regulatory subunit	Prkar2a					
2894	0.1334	0.6433	-0.0891	0.4446	0	S4R1D4		Ppfia1					
2895	0.6533	0.3750	-0.5264	0.4447	0	Q9D8N2	Protein FAM45A	Fam45a	mmu:67894				
2896	-0.1935	1.1188	0.7264	0.4447	0	P61358	60S ribosomal protein L27	Rpl27	mmu:19942				
2897	0.2096	-0.3582	0.1334	0.4451	0	Q3UDR8	Protein YIPF3;Protein YIPF3, N-terminally processed	Yipf3	mmu:28064				
2898	0.2640	0.7400	-0.4554	0.4460	0	Q69ZS7	HBS1-like protein	Hbs1l	mmu:56422				
2899	-1.0126	-0.0222	0.9363	0.4460	0	Q61333	Tumor necrosis factor alpha-induced protein 2	Tnfaip2	mmu:21928				
2900	0.8961	-0.8138	1.2605	0.4464	0	Q61771	Kinesin-like protein KIF3B;Kinesin-like protein KIF3B, N-terminally processed	Kif3b	mmu:16569				
2901	0.3012	[illegible]	-0.3032	0.4498	0	P97742	Carnitine O-palmitoyltransferase 1, liver isoform	Cpt1a	mmu:12894				
2902	0.1327	-0.4203	0.5518	0.4506	0	B1AY13	Ubiquitin carboxyl-terminal hydrolase;Ubiquitin carboxyl-terminal hydrolase 24	Usp24	mmu:329908				
2903	-0.2090	0.8546	0.4065	0.4518	0	P16045	Galectin-1	Lgals1	mmu:16852				1
2904	0.1613	0.7245	-0.1422	0.4521	0	Q8C3Y4	Kinetochore-associated protein 1	Kntc1	mmu:208628				
2905	0.2256	0.3381	-0.1515	0.4527	0	Q9D6N5	Dr1-associated corepressor	Drap1	mmu:66556				
2906	0.0160	-0.2098	0.4053	0.4528	0	Q99L47	Hsc70-interacting protein	St13	mmu:70356				
2907	-0.0951	0.6560	0.2863	0.4530	0	Q8BP67	60S ribosomal protein L24	Rpl24	mmu:68193				
2908	-0.1691	0.1002	0.5401	0.4579	0	P63328	Serine/threonine-protein phosphatase 2B catalytic subunit alpha isoform	Ppp3ca	mmu:19055		1		
2909	-1.2871	-0.4750	0.6707	0.4599	0	Q6S5J6	Krev interaction trapped protein 1	Krit1	mmu:79264				
2910	-0.2270	1.4657	0.4253	0.4654	0	Q3TIX9	U4/U6,U5 tri-snRNP-associated protein 2	Usp39	mmu:28035				
2911	-0.2707	0.1289	0.3421	0.4663	0	Q91W86	Vacuolar protein sorting-associated protein 11 homolog	Vps11	mmu:71732				
2912	0.7969	-0.3111	0.7142	0.4665	0	Q91WG2	Rab GTPase-binding effector protein 2	Rabep2	mmu:70314				
2913	0.3016	-0.0426	0.5776	0.4676	0	Q78ZA7	Nucleosome assembly protein 1-like 4	Nap1l4	mmu:17955				
2914	-0.2778	2.1581	-1.4751	0.4683	0	P55200	Histone-lysine N-methyltransferase 2A;MLL cleavage product N320;MLL cleavage product C180	Kmt2a	mmu:214162				
2915	0.8238	-0.1196	0.8704	0.4714	0	Q9Z2L7	Cytokine receptor-like factor 3	Crlf3	mmu:54394				
2916	3.3170	3.6184	-2.6050	0.4720	0	Q9D0R4	Probable ATP-dependent RNA helicase DDX56	Ddx56	mmu:52513				
2917	0.5872	0.3721	-0.0476	0.4735	0	Q8BWU5	Probable tRNA N6-adenosine threonylcarbamoyltransferase	Osgep	mmu:66246				
2918	0.2104	-0.1623	0.9682	0.4744	0	P14901	Heme oxygenase 1	Hmox1	mmu:15368	1			
2919	0.0168	-0.1914	-0.0794	0.4756	0	Q64105	Sepiapterin reductase	Spr					
2920	-0.1870	1.4753	0.6067	0.4785	0	G3X8X0		Dhx16	mmu:69192				
2921	0.5987	0.5747	-0.8658	0.4805	0	O88939	Zinc finger and BTB domain-containing protein 7A	Zbtb7a	mmu:16969				
2922	-0.1372	-0.2434	0.0085	0.4815	0	Q9D0M3	Cytochrome c1, heme protein, mitochondrial	Cyc1	mmu:66445				
2923	0.5110	-0.2880	-0.3705	0.4825	0	Q5NCI0	Up-regulator of cell proliferation	Urgcp	mmu:72046				
2924	0.4474	-0.0053	0.0244	0.4829	0	Q8BW72	Lysine-specific demethylase 4A	Kdm4a	mmu:230674				
2925	0.4674	-0.1501	0.0173	0.4830	0	P52019	Squalene monooxygenase	Sqle	mmu:20775				
2926	1.1354	-0.3628	-0.3218	0.4836	0	Q80UK0	SEC14 domain and spectrin repeat-containing protein 1	Sestd1	mmu:228071				
2927	-0.5629	-0.1211	0.4619	0.4844	0	Q8VDL4	ADP-dependent glucokinase	Adpgk	mmu:72141				
2928	0.4044	0.3325	-0.0681	0.4863	0	Q8R1F1	Niban-like protein 1	Fam129b	mmu:227737				
2929	0.4130	0.1436	-0.3852	0.4866	0	Q9D902	General transcription factor IIE subunit 2	Gtf2e2	mmu:68153				
2930	-0.1325	0.2541	0.7923	0.4874	0	Q9CQJ2	PIH1 domain-containing protein 1	Pih1d1	mmu:68845				
2931	-0.0720	0.2995	0.4923	0.4876	0	Q8VIJ6	Splicing factor, proline- and glutamine-rich	Sfpq	mmu:71514				
2932	0.4564	0.4876	-0.4579	0.4898	0	Q9Z2V5	Histone deacetylase 6	Hdac6	mmu:15185	1			
2933	-0.2498	-0.2652	1.7102	0.4905	0	E9Q4K7	Kinesin-like protein	Kif13b	mmu:16554				
2934	0.3806	0.0799	-0.9584	0.4907	0	P97470	Serine/threonine-protein phosphatase 4 catalytic subunit	Ppp4c	mmu:56420		1		
2935	-0.9268	0.7051	1.9551	0.4907	0	Q3ZK22	Vezatin	Vezt	mmu:215008				
2936	-0.2163	0.4414	0.9662	0.4908	0	P24668	Cation-dependent mannose-6-phosphate receptor	M6pr	mmu:17113				
2937	1.9663	0.3586	-0.3921	0.4916	0	Q61239	Protein farnesyltransferase/geranylgeranyltransferase type-1 subunit alpha	Fnta	mmu:14272				
2938	-0.2025	0.9287	0.3158	0.4932	0	P70459	ETS domain-containing transcription factor ERF	Erf	mmu:13875				
2939	0.2200	0.2987	-0.0451	0.4945	0	Q9WTI7	Unconventional myosin-Ic	Myo1c	mmu:17913				
2940	0.2619	-0.2261	0.5007	0.4949	0	Q9WV80	Sorting nexin-1	Snx1	mmu:56440				
2941	0.4002	-0.1658	-0.1193	0.4972	0	E9Q137		Tex264	mmu:21767				
2942	-0.1476	0.7453	-0.2949	0.4978	0	Q62470	Integrin alpha-3;Integrin alpha-3 heavy chain;Integrin alpha-3 light chain	Itga3	mmu:16400				
2943	0.3524	-0.0095	0.6185	0.4989	0	Q8K0H5	Transcription initiation factor TFIID subunit 10	Taf10;Gm4	mmu:24075				
2944	0.5390	-0.0270	0.1513	0.4994	0	Q8CGA0	Protein phosphatase 1F	Ppm1f	mmu:68606		1		
2945	-0.4637	-0.3507	1.0779	0.5002	0	Q9WTK5	Nuclear factor NF-kappa-B p100 subunit;Nuclear factor NF-kappa-B p52 subunit	Nfkb2	mmu:18034				1
2946	0.1193	-0.4813	-0.8148	0.5013	0	O88545	COP9 signalosome complex subunit 6	Cops6	mmu:26893				
2947	-0.2641	-0.0423	0.4104	0.5016	0	Q9D1N9	39S ribosomal protein L21, mitochondrial	Mrpl21	mmu:353242				
2948	0.5603	-0.6107	0.7782	0.5046	0	Q8BYU6	Torsin-1A-interacting protein 2	Tor1aip2					
2949	0.2965	-0.0484	0.3241	0.5109	0	P56135	ATP synthase subunit f, mitochondrial	Atp5j2	mmu:57423				
2950	-0.1485	0.1605	-0.1618	0.5115	0	Q9DBH5	Vesicular integral-membrane protein VIP36	Lman2	mmu:66890				
2951	-0.0624	0.5062	0.3612	0.5198	0	Q8K1S3	Netrin receptor UNC5B	Unc5b	mmu:107449				
2952	0.3668	-0.1921	1.0320	0.5219	0	Q62077	Phosphoinositide phospholipase C;1-phosphatidylinositol 4,5-bisphosphate phosphodiesterase ga	Plcg1	mmu:18803				
2953	0.1314	0.5550	-0.4193	0.5228	0	Q6A065	Centrosomal protein of 170 kDa	Cep170					
2954	-0.9679	0.6206	-0.8000	0.5252	0	Q9CQV1	Mitochondrial import inner membrane translocase subunit TIM16	Pam16	mmu:66449				
2955	-0.4538	0.9779	0.9587	0.5259	0	Q8BMJ3	Eukaryotic translation initiation factor 1A, X-chromosomal;Eukaryotic translation initiation factor 1	Eif1ax;Eif1	mmu:66235				
2956	-0.4403	0.5912	0.3238	0.5308	0	O08749	Dihydrolipoyl dehydrogenase, mitochondrial	Dld	mmu:13382				
2957	-0.2890	0.8203	0.2321	0.5312	0	Q8CHG7	Rap guanine nucleotide exchange factor 2	Rapgef2					
2958	0.0417	-0.0903	0.8322	0.5323	0	P56395	Cytochrome b5	Cyb5a	mmu:109672				
2959	0.0370	0.8117	-0.0346	0.5368	0	Q61165	Sodium/hydrogen exchanger 1	Slc9a1	mmu:20544				
2960	0.1164	-0.5611	0.2905	0.5370	0	Q8VCN6	CD99 antigen	Cd99	mmu:673094				
2961	-0.3553	0.4557	0.0427	0.5372	0	Q9QYJ0	DnaJ homolog subfamily A member 2	Dnaja2	mmu:56445				
2962	0.4798	0.1801	-0.8275	0.5376	0	Q61941	NAD(P) transhydrogenase, mitochondrial	Nnt					
2963	0.3435	-0.7919	-0.0217	0.5378	0	Q8VCL2	Protein SCO2 homolog, mitochondrial	Sco2	mmu:100126824				
2964	-0.4413	0.5993	0.1967	0.5379	0	O88746	Target of Myb protein 1	Tom1	mmu:21968				
2965	0.6735	-0.3744	-0.3090	0.5394	0	Q8C0D4	Rho GTPase-activating protein 12	Arhgap12	mmu:75415				
2966	-0.1463	0.4968	0.2981	0.5417	0	P23198	Chromobox protein homolog 3	Cbx3					
2967	0.0962	1.3501	-0.0152	0.5445	0	Q8CCS6	Polyadenylate-binding protein 2	Pabpn1	mmu:54196				
2968	0.6120	-0.3234	0.5473	0.5446	0	P35831	Tyrosine-protein phosphatase non-receptor type 12	Ptpn12	mmu:19248		1		
2969	0.9547	-0.0694	0.2710	0.5448	0	Q6DID7	Protein wntless homolog	Wls	mmu:68151				
2970	0.2846	-0.1511	0.9009	0.5452	0	Q61081	Hsp90 co-chaperone Cdc37;Hsp90 co-chaperone Cdc37, N-terminally processed	Cdc37	mmu:12539				
2971	-0.5872	0.2134	0.2967	0.5489	0	Q8C052	Microtubule-associated protein 1S;MAP1S heavy chain;MAP1S light chain	Map1s	mmu:270058				
2972	-0.2226	0.1301	0.1829	0.5514	0	Q8BXA5	Cleft lip and palate transmembrane protein 1-like protein	Clptm1l	mmu:218335				
2973	-0.4026	0.4230	1.1693	0.5528	0	Q9CQH7	Transcription factor BTF3 homolog 4	Btf3l4	mmu:70533				
2974	1.2353	0.9483	-0.1956	0.5532	0	P13808	Anion exchange protein 2	Slc4a2	mmu:20535				
2975	-0.4499	1.5660	0.3795	0.5594	0	O09131	Glutathione S-transferase omega-1	Gsto1	mmu:14873				
2976	1.9977	0.0983	-0.2321	0.5609	0	Q8R003	Muscleblind-like protein 3	Mbnl3	mmu:171170				
2977	-1.0726	0.3888	2.3726	0.5654	0	Q9CQE8	UPF0568 protein C14orf166 homolog		mmu:68045				
2978	0.5095	-0.1606	[illegible]	0.5671	0	Q9DC40	Telomere length regulation protein TEL2 homolog	Telo2	mmu:71718				
2979	-0.1794	-0.0541	1.0681	0.5710	0	Q80VQ1	Leucine-rich repeat-containing protein 1	Lrrc1	mmu:214345				
2980	0.2808	0.3466	-0.1375	0.5712	0	P70670	Nascent polypeptide-associated complex subunit alpha;Nascent polypeptide-associated complex s	Naca	mmu:17938				
2981	1.9581	0.6476	-0.7821	0.5722	0	Q3UH60	Disco-interacting protein 2 homolog B	Dip2b	mmu:239667				
2982	0.2972	-0.7976	0.8956	0.5725	0	O88653	Ragulator complex protein LAMTOR3	Lamtor3	mmu:56692				
2983	0.8517	0.9415	-0.1402	0.5736	0	Q7TPD0	Integrator complex subunit 3	Ints3	mmu:229543				
2984	-0.2722	0.9727	-0.3004	0.5842	0	Q6ZQ73	Cullin-associated NEDD8-dissociated protein 2	Cand2	mmu:67088				
2985	-0.0370	0.5876	0.7735	0.5850	0	P19157	Glutathione S-transferase P 1;Glutathione S-transferase P 2	Gstp1;Gstp	mmu:14870	1			1
2986	-0.0162	0.1325	0.3721	0.5854	0	Q8BFY9	Transportin-1	Tnpo1	mmu:238799				
2987	-0.1212	-0.2570	0.8225	0.5904	0	Q8R4H2	Rho guanine nucleotide exchange factor 12	Arhgef12					
2988	0.0990	-0.6525	0.2210	0.5905	0	A2AWP8	Rho guanine nucleotide exchange factor 10-like protein	Arhgef10l	mmu:72754				
2989	-0.2028	0.2325	0.9232	0.5910	0	E9QAB6		Ptar1	mmu:72351				
2990	0.1316	0.1070	-1.0858	0.5964	0	P61226	Ras-related protein Rap-2b	Rap2b	mmu:74012				
2991	-0.3675	0.8710	0.6116	0.5998	0	Q80V53	Carbohydrate sulfotransferase 14	Chst14	mmu:72136				
2992	0.8435	-0.3009	-0.7286	0.6048	0	P97363	Serine palmitoyltransferase 2	Sptlc2	mmu:20773				
2993	1.1625	0.7056	-0.3856	0.6095	0	P41105	60S ribosomal protein L28	Rpl28	mmu:19943				
2994	0.4168	2.0860	-0.9310	0.6105	0	P58854	Gamma-tubulin complex component 3	Tubgcp3	mmu:259279				
2995	-0.3017	0.4028	-0.3694	0.6122	0	Q78T54	Vacuolar ATPase assembly integral membrane protein Vma21	Vma21	mmu:67048				
2996	2.9125	-0.2444	-0.5586	0.6174	0	Q9D1A2	Cytosolic non-specific dipeptidase	Cndp2	mmu:66054				
2997	-0.4049	0.7945	-0.0367	0.6218	0	Q00PI9	Heterogeneous nuclear ribonucleoprotein U-like protein 2	Hnrnpul2	mmu:68693				
2998	0.6420	0.9065	-0.0193	0.6239	0	P81069	GA-binding protein subunit beta-2	Gabpb2	mmu:213054				
2999	-0.2586	0.4144	0.1851	0.6253	0	P35922	Fragile X mental retardation protein 1 homolog	Fmr1					
3000	1.5427	0.2656	-0.5940	0.6253	0	Q9D2V5	Protein AAR2 homolog	Aar2	mmu:68295				
3001	0.2435	-0.1757	-0.3611	0.6305	0	O88622	Poly(ADP-ribose) glycohydrolase	Parg					
3002	0.3324	-0.0733	0.6273	0.6338	0	Q9DBS9	Oxysterol-binding protein;Oxysterol-binding protein-related protein 3	Osbpl3	mmu:71720				
3003	0.5452	1.0115	-1.5012	0.6351	0	Q9JLC8	Sacsin	Sacs	mmu:50720				
3004	0.9749	-0.3032	-0.3534	0.6381	0	Q64337	Sequestosome-1	Sqstm1	mmu:18412	1			
3005	-0.2431	0.9822	1.5156	0.6441	0	O70310	Glycylpeptide N-tetradecanoyltransferase 1	Nmt1	mmu:18107				
3006	-0.2981	2.3779	-0.2539	0.6464	0	Q922S4	cGMP-dependent 3,5-cyclic phosphodiesterase	Pde2a					
3007	1.0370	-0.0275	0.2904	0.6691	0	Q8K0C1	Importin-13	Ipo13	mmu:230673				
3008	-0.0908	0.6776	-1.2068	0.6711	0	Q99K74	Mediator of RNA polymerase II transcription subunit 24	Med24	mmu:23989				
3009	1.0353	-0.3643	0.5070	0.6779	0	G3UW40		Mcc					
3010	0.0917	-0.0097	-0.3865	0.6845	0	Q9JIW9	Ras-related protein Ral-B	Ralb	mmu:64143				
3011	0.5500	-0.0433	0.3464	0.6865	0	Q9EQN3	TSC22 domain family protein 4	Tsc22d4	mmu:78829				
3012	1.6014	-0.1793	0.1039	0.6875	0	Q8BZW8	NHL repeat-containing protein 2	Nhlrc2	mmu:66866				
3013	1.0296	-0.0965	0.2825	0.6981	0	Q4FK66	Pre-mRNA-splicing factor 38A	Prpf38a	mmu:230596				

3014	0.0690	0.5383	-0.0620	0.7012	0	Q9EP69	Phosphatidylinositide phosphatase SAC1	Sacm1l	mmu:83493			1		
3015	0.2400	-0.1799	0.0394	0.7043	0	P35821	Tyrosine-protein phosphatase non-receptor type 1	Ptpn1	mmu:19246			1		
3016	0.4432	-0.2753	-0.5538	0.7052	0	Q8BFV2	PCI domain-containing protein 2	Pcid2	mmu:234069					
3017	0.8742	-0.3127	1.2245	0.7070	0	D3Z0L8		Actn4						
3018	-0.0545	0.0303	-0.7194	0.7074	0	P15379	CD44 antigen	Cd44	mmu:12505					
3019	0.4196	-0.0708	-0.1523	0.7093	0	O35864	COP9 signalosome complex subunit 5	Cops5	mmu:26754					
3020	0.0678	0.0993	-0.0103	0.7161	0	Q3UFK8	FERM domain-containing protein 8	Frmd8	mmu:67457					
3021	-0.4691	0.2080	-1.2247	0.7243	0	O08734	Bcl-2 homologous antagonist/killer	Bak1	mmu:12018		1			1
3022	0.3534	0.8545	-1.3164	0.7245	0	Q8K368	Fanconi anemia group I protein homolog	Fanci	mmu:208836					
3023	0.0420	-0.3515	0.9101	0.7267	0	Q9CQ80	Vacuolar protein-sorting-associated protein 25	Vps25	mmu:28084					
3024	-0.2439	0.2718	0.0849	0.7324	0	P62862	40S ribosomal protein S30	Fau						
3025	0.8397	-0.1090	0.7595	0.7326	0	Q3UN02	Lysocardiolipin acyltransferase 1	Lclat1	mmu:225010					
3026	-0.0502	-0.0467	0.6284	0.7384	0	Q8R2R3	Alpha- and gamma-adaptin-binding protein p34	Aagab	mmu:66939					
3027	0.8593	-0.0127	-0.8681	0.7398	0	O35379	Multidrug resistance-associated protein 1	Abcc1	mmu:17250					
3028	0.3765	-0.0587	0.7377	0.7480	0	O09130	NFATC2-interacting protein	Nfatc2ip	mmu:18020					
3029	0.3898	0.1279	-0.2832	0.7586	0	P61804	Dolichyl-diphosphooligosaccharide--protein glycosyltransferase subunit DAD1	Dad1	mmu:13135					1
3030	-0.2398	0.5720	0.4512	0.7597	0	Q3THS6	S-adenosylmethionine synthase isoform type-2	Mat2a	mmu:232087					
3031	4.6375	-0.4061	-0.8357	0.7728	0	Q9D600	DNA replication complex GINS protein PSF2	Gins2	mmu:272551					
3032	-0.2468	0.1329	0.6770	0.7750	0	P84096	Rho-related GTP-binding protein RhoG	Rhog	mmu:56212					
3033	0.5513	0.7542	-1.0297	0.7820	0	Q921X6	DNA-directed RNA polymerase III subunit RPC6	Polr3f	mmu:70408					
3034	-0.2001	0.6180	1.4634	0.7951	0	Q60520	Paired amphipathic helix protein Sin3a	Sin3a	mmu:20466		1			
3035	0.1308	-0.0663	1.8577	0.8001	0	Q9D4F8	Gamma-tubulin complex component 4	Tubgcp4	mmu:51885					
3036	0.4781	-0.1888	-0.4258	0.8087	0	P61166	Transmembrane protein 258	Tmem258	mmu:69038					
3037	0.0622	0.7135	-0.0868	0.8105	0	P48377	MHC class II regulatory factor RFX1	Rfx1	mmu:19724					
3038	0.0431	0.5072	-0.3873	0.8271	0	Q8VCX5	Calcium uptake protein 1, mitochondrial	Micu1	mmu:216001					
3039	2.3861	0.7044	-0.4254	0.8328	0	F8VPP8		Zc3h7b	mmu:20286					
3040	-0.0192	0.2462	0.2490	0.8431	0	P47754	F-actin-capping protein subunit alpha-2	Capza2	mmu:12343					
3041	0.1980	1.9336	-0.6963	0.8480	0	A2AT37		Upf2	mmu:326622					
3042	2.5438	0.3045	-1.3408	0.8807	0	P61620	Protein transport protein Sec61 subunit alpha isoform 1	Sec61a1;Se	mmu:53421					
3043	-0.3810	3.3133	-0.9807	0.8895	0	Q8C2A2	tRNA-splicing endonuclease subunit Sen54	Tsen54	mmu:76265					
3044	0.3019	0.0209	-0.0508	0.8941	0	Q9DC16	Endoplasmic reticulum-Golgi intermediate compartment protein 1	Ergic1	mmu:67458					
3045	0.5827	-0.0454	1.1767	0.8974	0	Q9QXJ1	Amyloid beta A4 precursor protein-binding family B member 1	Apbb1	mmu:11785					
3046	-0.0968	1.2754	0.8177	0.9035	0	Q9JLV6	Bifunctional polynucleotide phosphatase/kinase;Polynucleotide 3-phosphatase;Polynucleotide 5-h	Pnkp			1			
3047	-0.3304	1.7839	0.5966	0.9081	0	Q05A36	RNA-binding E3 ubiquitin-protein ligase MEX3C;RNA-binding protein MEX3B;RNA-binding protein	Mex3c;Me	mmu:240396					
3048	0.3571	-0.2037	-0.1889	0.9093	0	Q9D7N9	Adipocyte plasma membrane-associated protein	Apmap	mmu:71881					
3049	-0.0247	0.5596	0.4067	0.9189	0	Q9D3W4	GPN-loop GTPase 3	Gpn3	mmu:68080					
3050	-0.0893	1.4448	1.5727	0.9291	0	Q60660	Killer cell lectin-like receptor 2	Klra2						
3051	1.0875	0.3174	-0.3235	0.9340	0	Q99K70	Ras-related GTP-binding protein C;Ras-related GTP-binding protein D	Rragc;Rrag	mmu:54170					
3052	-0.2447	-0.3213	[illegible]	0.9350	0	Q80V26	Inositol monophosphatase 3	Impad1	mmu:242291			1		
3053	2.1715	-0.0267	-0.4820	0.9580	0	Q64310	Surfeit locus protein 4	Surf4	mmu:20932					
3054	0.1863	-0.0219	2.0116	0.9614	0	Q9WVM3	Anaphase-promoting complex subunit 7	Anapc7	mmu:56317				1	
3055	1.0332	-0.9479	-0.1077	0.9651	0	O08808	Protein diaphanous homolog 1	Diap1;Diap	mmu:13367					
3056	-0.6954	-0.2363	1.9658	1.0248	0	P42227	Signal transducer and activator of transcription 3;Signal transducer and activator of transcription	Stat3	mmu:20848					
3057	0.2318	-0.3654	0.8082	1.0249	0	Q9Z247	Peptidyl-prolyl cis-trans isomerase FKBP9	Fkbp9	mmu:27055					
3058	0.7658	0.4348	-0.0716	1.0251	0	P47753	F-actin-capping protein subunit alpha-1	Capza1	mmu:12340					
3059	-0.1599	0.8109	0.1240	1.0296	0	Q80Y17	Lethal(2) giant larvae protein homolog 1	Llgl1	mmu:16897					
3060	-0.2422	0.2877	-0.8312	1.0338	0	Q8CHG3	GRIP and coiled-coil domain-containing protein 2	Gcc2						
3061	-0.7968	0.6858	4.4343	1.0488	0	P70460	Vasodilator-stimulated phosphoprotein	Vasp	mmu:22323					
3062	0.1990	2.7529	-0.9569	1.0538	0	Q8BMK4	Cytoskeleton-associated protein 4	Ckap4	mmu:216197					
3063	-0.2253	1.4215	1.1540	1.0756	0	Q9D1C8	Vacuolar protein sorting-associated protein 28 homolog	Vps28	mmu:66914					
3064	-0.3139	1.2699	-0.1645	1.0898	0	Q9JJK2	LanC-like protein 2	Lancl2	mmu:71835					
3065	-0.1733	1.0856	0.0550	1.1135	0	Q8K2V6	Importin-11	Ipo11	mmu:76582					
3066	0.3564	-0.2306	0.0299	1.1228	0	P97370	Sodium/potassium-transporting ATPase subunit beta-3	Atp1b3	mmu:11933					
3067	1.3348	1.7194	-0.0456	1.1374	0	Q8C1Y8	Vacuolar fusion protein CCZ1 homolog	Ccz1	mmu:231874					
3068	1.6650	0.4127	-0.3461	1.1660	0	Q78IK4	MICOS complex subunit Mic27	Apool	mmu:68117					
3069	0.4680	-0.5743	2.1788	1.1888	0	E9Q735	Ubiquitin conjugation factor E4 A	Ube4a	mmu:140630				1	
3070	3.1223	-0.4365	0.1993	1.2571	0	Q6ZWR6	Nesprin-1	Syne1	mmu:64009					
3071	-0.1831	1.0239	-0.2188	1.3071	0	Q9D883	Splicing factor U2AF 35 kDa subunit;Splicing factor U2AF 26 kDa subunit	U2af1;U2a	mmu:108121					
3072	-0.4867	1.7669	2.1305	1.3717	0	Q9R118	Serine protease HTRA1	Htra1	mmu:56213					
3073	-1.1778	0.0940	0.1018	1.4034	0	Q9JKV1	Proteasomal ubiquitin receptor ADRM1	Gm9774;A	mmu:56436					
3074	-0.1597	0.1093	-0.8318	1.4260	0	P12787	Cytochrome c oxidase subunit 5A, mitochondrial	Cox5a	mmu:12858					
3075	-0.2139	0.5275	0.2023	1.5812	0	Q60631	Growth factor receptor-bound protein 2	Grb2	mmu:14784					
3076	-0.9518	0.1599	-0.5328	1.5938	0	P62743	AP-2 complex subunit sigma	Ap2s1	mmu:232910					
3077	0.6600	-1.1089	0.2229	1.6823	0	Q9D281	Protein Noxp20	Fam114a1	mmu:68303					
3078	-0.4011	-1.0416	0.0250	-0.4726	0	Q8BSK8	Ribosomal protein S6 kinase beta-1	Rps6kb1	mmu:72508	1				
3079	-0.4159	-0.2161	0.0238	-0.2027	0	P70335	Rho-associated protein kinase 1	Rock1	mmu:19877	1				
3080	0.3830	-0.6297	-0.0120	-0.0862	0	Q80XP9	Serine/threonine-protein kinase WNK3	Wnk3	mmu:279561	1				
3081	0.6148	-0.2935	-0.1791	0.0474	0	Q6PF93	Phosphatidylinositol 3-kinase;Phosphatidylinositol 3-kinase catalytic subunit type 3	Pik3c3	mmu:225326	1				
3082	1.4993	-0.0856	-1.0333	0.1268	0	Q9DC28	Casein kinase I isoform delta	Csnk1d	mmu:104318	1				
3083	2.2733	-0.4411	-0.3197	0.5042	0	Q9DC28	Casein kinase I isoform delta	Csnk1d	mmu:104318	1				
3084	1.3960	0.5083	-0.1611	0.5811	0	Q9WTR2	Mitogen-activated protein kinase kinase kinase 6	Map3k6		1				
3085	-2.7247	0.0735	-1.5969	-1.4160	0	P62254	Ubiquitin-conjugating enzyme E2 G1;Ubiquitin-conjugating enzyme E2 G1, N-terminally processed	Ube2g1	mmu:67128				1	
3086	0.1690	-2.3199	-1.3359	-1.1623	0	Q8BFT2	HAUS augmin-like complex subunit 4	Haus4	mmu:219072					
3087	0.1057	-0.7628	-2.7694	-1.1422	0	Q9R0P5	Destrin	Dstn	mmu:56431					
3088	0.1556	-0.6774	-2.1870	-0.9029	0	Q791V5	Mitochondrial carrier homolog 2	Mtch2	mmu:56428					
3089	-1.6748	0.2496	-1.0873	-0.8375	0	Q8C142	Low density lipoprotein receptor adapter protein 1	Ldlrap1	mmu:100017					
3090	-3.0142	0.8082	-0.2998	-0.8353	0	P62340	TATA box-binding protein-like protein 1	Tbpl1	mmu:237336					
3091	-2.5277	1.7408	-1.6439	-0.8103	0	Q9D8C4	Interferon-induced 35 kDa protein homolog	Ifi35	mmu:70110					
3092	-1.2373	0.2427	-1.2623	-0.7523	0	Q8R3I3	Conserved oligomeric Golgi complex subunit 6	Cog6	mmu:67542					
3093	-1.1571	1.1265	-2.1920	-0.7409	0	Q8K2C9	Very-long-chain (3R)-3-hydroxyacyl-CoA dehydratase 3	Hacd3	mmu:57874					
3094	-2.2350	0.0590	-0.0274	-0.7345	0	E9Q7L1		Urb2	mmu:382038					
3095	-0.7327	-1.4618	0.1061	-0.6961	0	Q9CR59	Growth arrest and DNA damage-inducible proteins-interacting protein 1	Gadd45gip	mmu:102060					
3096	-0.9782	-1.0596	0.1016	-0.6454	0	Q60739	BAG family molecular chaperone regulator 1	Bag1	mmu:12017					1
3097	-1.0318	0.0176	-0.9069	-0.6404	0	Q8C3X2	Coiled-coil domain-containing protein 90B, mitochondrial	Ccdc90b	mmu:66365					
3098	-0.1983	0.1940	-1.9030	-0.6358	0	Q99LC8	Translation initiation factor eIF-2B subunit alpha	Eif2b1	mmu:209354					
3099	0.1222	-1.2267	-0.7885	-0.6310	0	Q3U0V2	Tumor necrosis factor receptor type 1-associated DEATH domain protein	Tradd	mmu:71609					1
3100	-0.3359	0.0942	-1.6470	-0.6296	0	P62322	U6 snRNA-associated Sm-like protein LSm5	Lsm5	mmu:66373					
3101	0.2245	-1.2552	-0.8335	-0.6214	0	Q9D945	Protein LLP homolog	Llph	mmu:66225					
3102	0.0501	-1.2403	-0.6533	-0.6145	0	Q8C6I2	Succinate dehydrogenase assembly factor 2, mitochondrial	Sdhaf2	mmu:66072					
3103	0.0807	-0.1697	-1.7203	-0.6031	0	Q00780	Collagen alpha-1(VIII) chain;Vastatin	Col8a1	mmu:12837					
3104	0.3248	-0.9552	-1.1436	-0.5913	0	P37889	Fibulin-2	Fbln2	mmu:14115					
3105	-0.6129	-1.2586	0.1797	-0.5639	0	Q61263	Sterol O-acyltransferase 1	Soat1	mmu:20652					
3106	-0.8078	0.3377	-1.2111	-0.5604	0	Q8R2Y9	SOSS complex subunit B1	Nabp2	mmu:69917					
3107	0.0116	0.4417	-2.1340	-0.5602	0	P47955	60S acidic ribosomal protein P1	Gm10073;I	mmu:56040					
3108	0.3548	-0.9499	-1.0799	-0.5583	0	Q9D6L8	Peptidyl-prolyl cis-trans isomerase-like 3	Ppil3	mmu:70225					
3109	0.3885	-1.1635	-0.8732	-0.5494	0	Q8VC57	BTB/POZ domain-containing protein KCTD5	Kctd5	mmu:69259					
3110	2.6164	-1.4124	-2.8376	-0.5445	0	P38060	Hydroxymethylglutaryl-CoA lyase, mitochondrial	Hmgcl	mmu:15356					
3111	-0.2644	-1.7653	0.5003	-0.5098	0	Q3V4B5	COMM domain-containing protein 6	Commd6	mmu:66200					
3112	-0.1956	0.6361	-1.9493	-0.5029	0	Q9DBT3	Coiled-coil domain-containing protein 97	Ccdc97	mmu:52132					
3113	-0.5681	-1.4791	0.6164	-0.4769	0	Q91WG8	Bifunctional UDP-N-acetylglucosamine 2-epimerase/N-acetylmannosamine kinase;UDP-N-acetylglu	Gne	mmu:50798					
3114	-1.2722	-0.2358	0.0953	-0.4709	0	Q8BH69	Selenide, water dikinase 1	Sephs1	mmu:109079					
3115	-1.3431	0.0203	-0.0738	-0.4655	0	Q9CUX1	52 kDa repressor of the inhibitor of the protein kinase	Prkrir	mmu:72981					
3116	-0.2193	-1.2069	0.0407	-0.4618	0	P62073	Mitochondrial import inner membrane translocase subunit Tim10	Timm10	mmu:30059					
3117	-0.1652	0.6773	-1.8880	-0.4586	0	O88393	Transforming growth factor beta receptor type 3	Tgfbr3	mmu:21814					
3118	0.3585	-0.4087	-1.2380	-0.4294	0	P05977	Myosin light chain 1/3, skeletal muscle isoform;Myosin light chain 3	Myl1;Myl3	mmu:17901					
3119	1.0070	-0.9662	-1.2609	-0.4067	0	Q91W53	Golgin subfamily A member 7	Golga7	mmu:57437					
3120	3.4441	-0.9638	-3.6980	-0.4059	0	Q923A2	Protein Spindly	Spdl1	mmu:70385					
3121	-0.5376	0.1601	-0.7703	-0.3826	0	Q9CPN8	Insulin-like growth factor 2 mRNA-binding protein 3	Igf2bp3	mmu:140488					
3122	0.5542	-0.4943	-1.1891	-0.3764	0	Q99N93	39S ribosomal protein L16, mitochondrial	Mrpl16	mmu:94063					
3123	-0.9503	0.2168	-0.3770	-0.3702	0	Q3UM45	Protein phosphatase 1 regulatory subunit 7	Ppp1r7	mmu:66385					
3124	0.1046	-1.8494	0.6407	-0.3680	0	Q99LH9	SH3 domain-binding protein 5-like	Sh3bp5l	mmu:79566					
3125	1.4327	-0.8679	-1.6638	-0.3663	0	Q99L85	Elongator complex protein 5	Elp5	mmu:54351					
3126	-1.3629	0.5961	-0.2708	-0.3459	0	Q99PI5	Phosphatidate phosphatase LPIN2	Lpin2	mmu:64898					
3127	-0.8818	-0.2904	0.1437	-0.3428	0	Q8VD62	UPF0696 protein C11orf68 homolog	AI837181;I	mmu:107242					
3128	-0.6070	-0.4204	0.0056	-0.3406	0	Q8K1R3	Polyribonucleotide nucleotidyltransferase 1, mitochondrial	Pnpt1	mmu:71701		1			
3129	0.0549	-0.8701	-0.1759	-0.3304	0	Q5U5Q9	BRCA1-A complex subunit RAP80	Uimc1	mmu:20184					
3130	0.0118	-0.1005	-0.8883	-0.3257	0	Q78WZ7	DNA-directed RNA polymerase I subunit RPA43	Twistnb	mmu:28071					
3131	-0.6856	0.7099	-1.0013	-0.3257	0	Q99JF5	Diphosphomevalonate decarboxylase	Mvd	mmu:192156					
3132	0.1071	-0.6265	-0.4359	-0.3184	0	P18826	Phosphorylase b kinase regulatory subunit alpha, skeletal muscle isoform	Phka1	mmu:18679					
3133	-0.4941	1.0616	-1.5219	-0.3181	0	Q5SS80	Dehydrogenase/reductase SDR family member 13	Dhrs13	mmu:70451					
3134	0.2291	-0.5881	-0.5878	-0.3156	0	Q99MR1	PERQ amino acid-rich with GYF domain-containing protein 1	Gigyf1	mmu:57330					
3135	-0.0145	0.0341	-0.9459	-0.3088	0	Q91VB4	Hermansky-Pudlak syndrome 3 protein homolog	Hps3	mmu:12807					
3136	-0.5364	0.1223	-0.4476	-0.2872	0	Q9WU42	Nuclear receptor corepressor 2	Ncor2	mmu:20602					
3137	-0.8965	0.1349	-0.0988	-0.2868	0	P32020	Non-specific lipid-transfer protein	Scp2	mmu:20280					
3138	-0.6286	0.1816	-0.4059	-0.2843	0	Q7TSI1	Pleckstrin homology domain-containing family M member 1	Plekhm1	mmu:353047					
3139	-0.4860	-0.3576	0.0268	-0.2723	0	Q8C5D8	E3 SUMO-protein ligase PIAS2;E3 SUMO-protein ligase PIAS1	Pias2;Pias:	mmu:17344					

3140	-0.8990	0.3535	-0.2431	-0.2629	0	Q9DBL9	1-acylglycerol-3-phosphate O-acyltransferase ABHD5	Abhd5	mmu:67469				
3141	0.5928	-0.9438	-0.4343	-0.2618	0	O89050	Muskelin	Mkln1	mmu:27418				
3142	-0.0808	0.0498	-0.7499	-0.2603	0	Q91V61	Sideroflexin-3	Sfxn3	mmu:94280				
3143	-1.0828	-0.1021	0.4269	-0.2527	0	Q91WE4	UPF0729 protein C18orf32 homolog		mmu:407819				
3144	0.2398	-0.7016	-0.2776	-0.2465	0	Q9Z2F7	BCL2/adenovirus E1B 19 kDa protein-interacting protein 3-like	Bnip3l	mmu:12177				1
3145	-0.9699	-0.0141	0.2579	-0.2420	0	Q7TSQ8	Pyruvate dehydrogenase phosphatase regulatory subunit, mitochondrial	Pdpr	mmu:319518				
3146	-0.2028	0.0359	-0.5529	-0.2399	0	Q9D3D9	ATP synthase subunit delta, mitochondrial	Atp5d	mmu:66043				
3147	0.1556	0.1395	-1.0000	-0.2350	0	Q9JHS3	Ragulator complex protein LAMTOR2	Lamtor2	mmu:83409				
3148	-0.3192	-0.5326	0.1507	-0.2337	0	Q8R1V4	Transmembrane emp24 domain-containing protein 4	Tmed4	mmu:103694				
3149	-0.2865	0.1447	-0.5526	-0.2315	0	Q62425	Cytochrome c oxidase subunit NDUFA4	Ndufa4	mmu:17992				
3150	-0.3916	0.3295	-0.6263	-0.2295	0	Q9Z0L8	Gamma-glutamyl hydrolase	Ggh	mmu:14590				
3151	-1.7376	-0.7581	1.8206	-0.2250	0	Q61147	Ceruloplasmin	Cp	mmu:12870				
3152	-0.4775	0.6250	-0.8113	-0.2213	0	Q920Q8	Influenza virus NS1A-binding protein homolog	Ivns1abp	mmu:117198				
3153	0.0530	0.0957	-0.8089	-0.2201	0	Q9JIB4	General transcription factor IIH subunit 2	Gtf2h2	mmu:23894				
3154	0.8283	-0.7611	-0.7262	-0.2197	0	S4R2G9		Akr1b10					
3155	-0.0452	0.5875	-1.1942	-0.2173	0	P49817	Caveolin;Caveolin-1;Caveolin-3	Cav1;Cav3	mmu:12389				
3156	0.1397	-0.3920	-0.3915	-0.2146	0	Q76LS9	Protein FAM63A	Fam63a	mmu:75007				
3157	-0.0417	0.4541	-1.0556	-0.2144	0	Q9D2Z4	Sentrin-specific protease 8	Senp8	mmu:71599				
3158	0.5835	0.6438	-1.8651	-0.2126	0	O08740	DNA-directed RNA polymerase II subunit RPB11	Polr2j					
3159	0.5756	-0.5902	-0.6229	-0.2125	0	P97760	DNA-directed RNA polymerase II subunit RPB3	Polr2c	mmu:20021				
3160	-0.5944	-0.2328	0.2150	-0.2041	0	P61027	Ras-related protein Rab-10	Rab10	mmu:19325				
3161	0.2555	-0.7904	-0.0772	-0.2040	0	A2AJL3	FGGY carbohydrate kinase domain-containing protein	Fggy	mmu:75578				
3162	-1.5446	0.0820	0.8640	-0.1995	0	Q9CZ83	39S ribosomal protein L55, mitochondrial	Mrpl55	mmu:67212				
3163	0.2602	-0.0648	-0.7900	-0.1982	0	Q9D720	Non-structural maintenance of chromosomes element 1 homolog	Nsmce1	mmu:67711				
3164	-0.4898	-0.3862	0.3048	-0.1904	0	A1L3P4	Sodium/hydrogen exchanger	Slc9a6	mmu:236794				
3165	0.8069	-0.9161	-0.4423	-0.1838	0	Q3UFY7	7-methylguanosine phosphate-specific 5-nucleotidase	Nt5c3b	mmu:68106				
3166	-0.5206	0.1355	-0.1625	-0.1825	0	Q9CQ06	39S ribosomal protein L24, mitochondrial	Mrpl24	mmu:67707				
3167	0.1854	0.1547	-0.8638	-0.1746	0	Q5SSW2	Proteasome activator complex subunit 4	Psme4	mmu:103554				
3168	0.3627	-1.1111	0.2319	-0.1722	0	Q8BTZ4	Anaphase-promoting complex subunit 5	Anapc5	mmu:59008			1	
3169	-0.2663	0.8918	-1.1253	-0.1666	0	Q9CYN2	Signal peptidase complex subunit 2	Spcs2	mmu:66624				
3170	0.3025	-0.8254	0.0253	-0.1659	0	Q8BGB7	Enolase-phosphatase E1	Enoph1	mmu:67870				
3171	-0.5333	0.1331	-0.0811	-0.1604	0	Q8R1J9	Torsin-2A	Tor2a	mmu:30933				
3172	-0.1623	0.2305	-0.5429	-0.1582	0	P19536	Cytochrome c oxidase subunit 5B, mitochondrial	Cox5b					
3173	-0.6065	-0.1495	0.2834	-0.1575	0	Q9Z179	SHC SH2 domain-binding protein 1	Shcbp1	mmu:20419				
3174	-1.3856	-1.2830	2.2013	-0.1558	0	Q3USH5	Splicing factor, suppressor of white-apricot homolog	Sfswap	mmu:231769				
3175	0.0569	0.2669	-0.7883	-0.1548	0	Q9CQQ4	Gem-associated protein 2	Gemin2	mmu:66603				
3176	0.2507	-0.0309	-0.6802	-0.1535	0	Q8BPI1	Protein kintoun	Dnaaf2	mmu:109065				
3177	-0.1171	-0.7435	0.4034	-0.1524	0	Q8JZL3	Thiamine-triphosphatase	Thtpa	mmu:105663				
3178	-0.2746	-0.6148	0.4416	-0.1493	0	Q8K296	Myotubularin-related protein 3	Mtmr3			1		
3179	-0.7092	0.8277	-0.5636	-0.1484	0	Q9CX13	Protein cornichon homolog 4	Cnih4	mmu:98417				
3180	-1.2390	0.3185	0.4844	-0.1454	0	P61082	NEDD8-conjugating enzyme Ubc12	Ube2m	mmu:22192				
3181	0.2034	-0.7447	0.1135	-0.1426	0	P50637	Translocator protein	Tspo	mmu:12257				
3182	0.1971	-0.0200	-0.6004	-0.1411	0	Q9JJG0	Transforming acidic coiled-coil-containing protein 2	Tacc2	mmu:57752				
3183	0.1704	-0.1224	-0.4680	-0.1400	0	O88522	NF-kappa-B essential modulator	Ikbkg	mmu:16151				1
3184	-0.3264	-0.6937	0.6032	-0.1390	0	Q3UGR5	Haloacid dehalogenase-like hydrolase domain-containing protein 2	Hdhd2	mmu:76987		1		
3185	-1.2310	-0.2741	1.0895	-0.1385	0	Q9Z130	Heterogeneous nuclear ribonucleoprotein D-like	Hnrnpdl					
3186	1.6185	0.1485	-2.1797	-0.1376	0	Q9CY27	Very-long-chain enoyl-CoA reductase	Tecr	mmu:106529				
3187	0.5467	-0.4442	-0.5088	-0.1354	0	Q6NSQ7	Protein LTV1 homolog	Ltv1	mmu:353258				
3188	0.3499	-0.6079	-0.1402	-0.1327	0	Q80UW8	DNA-directed RNA polymerases I, II, and III subunit RPABC1	Polr2e	mmu:66420				
3189	0.6457	-0.3585	-0.6831	-0.1320	0	Q3TMX7	Sulfhydryl oxidase 2	Qsox2	mmu:227638				
3190	-0.0624	-0.3784	0.0478	-0.1310	0	O55103	Periaxin	Prx	mmu:19153				
3191	-0.7461	0.1076	0.2543	-0.1281	0	Q8C6G8	WD repeat-containing protein 26	Wdr26	mmu:226757				
3192	1.5061	-0.4467	-1.4416	-0.1274	0	Q6PNC0	DmX-like protein 1	Dmxl1	mmu:240283				
3193	-1.0776	0.3188	0.3843	-0.1248	0	Q9D0I8	mRNA turnover protein 4 homolog	Mrto4	mmu:69902				
3194	-0.0515	0.3341	-0.6547	-0.1240	0	Q9DCF9	Translocon-associated protein subunit gamma	Ssr3	mmu:67437				
3195	-0.3103	-0.3439	0.2878	-0.1221	0	Q9CQM5	Thioredoxin domain-containing protein 17	Txndc17	mmu:52700				
3196	-0.7255	0.8460	-0.4861	-0.1219	0	Q9D819	Inorganic pyrophosphatase	Ppa1	mmu:67895				
3197	-0.0695	-0.4902	0.1985	-0.1204	0	Q8CCN5	Breast carcinoma-amplified sequence 3 homolog	Bcas3	mmu:192197				
3198	0.4827	-0.6091	-0.2321	-0.1195	0	B2RS91	RNA polymerase I-specific transcription initiation factor RRN3	Rrn3	mmu:106298				
3199	-0.5741	0.1540	0.0825	-0.1125	0	Q9CYZ6	Uncharacterized protein C19orf60 homolog	2810428I1	mmu:66462				
3200	0.5728	-1.1687	0.2615	-0.1115	0	O35427	DNA-directed RNA polymerase III subunit RPC9	Crcp	mmu:12909				
3201	-0.2262	0.3482	-0.4446	-0.1075	0	Q9CX97	WD repeat-containing protein 55	Wdr55	mmu:67936				
3202	0.2542	-0.3099	-0.2620	-0.1059	0	E9PY43		Erich1	mmu:234086				
3203	0.7657	-0.0206	-1.0288	-0.0946	0	Q9DC63	F-box only protein 3	Fbxo3	mmu:57443				
3204	-0.8058	0.1608	0.3778	-0.0891	0	Q8C7K6	Prenylcysteine oxidase-like	Pcyox1l	mmu:240334				
3205	0.2145	-0.0184	-0.4629	-0.0889	0	Q80W54	CAAX prenyl protease 1 homolog	Zmpste24	mmu:230709				
3206	-0.2695	0.2798	-0.2724	-0.0874	0	P99026	Proteasome subunit beta type-4	Psmb4	mmu:19172				
3207	0.2650	0.3081	-0.8281	-0.0850	0	Q570Y9	DEP domain-containing mTOR-interacting protein	Deptor	mmu:97998				
3208	-0.4798	-0.1144	0.3464	-0.0826	0	Q60714	Long-chain fatty acid transport protein 1	Slc27a1	mmu:26457				
3209	0.3087	-0.5528	[illegible]	-0.0819	0	Q6IE82	Protein Jade-3	Jade3	mmu:382207				
3210	0.0552	[illegible]	-0.2925	-0.0791	0	P26350	Prothymosin alpha;Prothymosin alpha, N-terminally processed;Thymosin alpha	Ptma	mmu:19231				
3211	-0.7710	-0.0620	0.5978	-0.0784	0	Q8CDM8	Protein FAM160B1	Fam160b1	mmu:226252				
3212	0.0503	-0.2792	[illegible]	-0.0759	0	Q3TPE9	Ankyrin repeat and MYND domain-containing protein 2	Ankmy2	mmu:217473				
3213	-0.1543	0.4457	-0.5168	-0.0751	0	Q9CWR7	Metalloreductase STEAP1	Steap1	mmu:70358				
3214	-0.3159	-0.3313	0.4401	-0.0690	0	O55057	Retinal rod rhodopsin-sensitive cGMP 3,5-cyclic phosphodiesterase subunit delta	Pde6d	mmu:18582				
3215	0.3484	-0.8960	0.3475	-0.0667	0	Q8C3R1	BRCA1-associated ATM activator 1	Brat1	mmu:231841				
3216	-1.0539	-0.3343	1.1977	-0.0635	0	A0A087WSL5		Tuba4a					
3217	-0.8150	0.2871	0.3391	-0.0629	0	O35435	Dihydroorotate dehydrogenase (quinone), mitochondrial	Dhodh	mmu:56749				
3218	-0.5454	0.3119	0.0537	-0.0599	0	A2RSY6	TRMT1-like protein	Trmt1l	mmu:98685				
3219	0.7261	0.1264	-1.0278	-0.0584	0	Q80XR2	Calcium-transporting ATPase;Calcium-transporting ATPase type 2C member 1	Atp2c1	mmu:235574				
3220	0.0963	0.2332	-0.4970	-0.0558	0	Q9CWY8	Ribonuclease;Ribonuclease H2 subunit A	Rnaseh2a	mmu:69724				
3221	-0.6546	0.1441	0.3533	-0.0524	0	Q78JE5	F-box only protein 22	Fbxo22	mmu:71999				
3222	0.4562	-0.3600	-0.2342	-0.0460	0	Q99KF1	Transmembrane emp24 domain-containing protein 9	Tmed9	mmu:67511				
3223	0.2147	-0.7554	0.4106	-0.0434	0	A3KGB4	TBC1 domain family member 8B	Tbc1d8b	mmu:245638				
3224	-0.6365	0.4032	0.1061	-0.0424	0	O70325	Glutathione peroxidase;Phospholipid hydroperoxide glutathione peroxidase, mitochondrial;Phosp	Gpx4	mmu:625249	1			
3225	0.1436	-0.1330	-0.1352	-0.0415	0	P63087	Serine/threonine-protein phosphatase PP1-gamma catalytic subunit	Ppp1cc	mmu:19047		1		
3226	0.5119	-0.0657	-0.5648	-0.0395	0	Q3U4G3	Xyloside xylosyltransferase 1	Xxylt1	mmu:268880				
3227	0.1372	0.1010	-0.3479	-0.0366	0	O88895	Histone deacetylase;Histone deacetylase 3	Hdac3					1
3228	-0.0268	-0.8011	0.7272	-0.0336	0	Q924D0	Reticulon-4-interacting protein 1, mitochondrial	Rtn4ip1	mmu:170728				
3229	-0.7163	0.1203	0.4976	-0.0328	0	P97376	Protein FRG1	Frg1	mmu:14300				
3230	-0.0212	0.1353	-0.1994	-0.0284	0	P63002	Amino-terminal enhancer of split	Aes	mmu:14797				
3231	0.6111	0.3460	-1.0392	-0.0274	0	Q8R2Q4	Ribosome-releasing factor 2, mitochondrial	Gfm2	mmu:320806				
3232	-0.5516	0.9471	-0.4763	-0.0269	0	Q3U276	Succinate dehydrogenase assembly factor 1, mitochondrial	Sdhaf1	mmu:68332				
3233	0.0194	-0.2300	0.1335	-0.0257	0	Q9JM13	Rab5 GDP/GTP exchange factor	Rabgef1	mmu:56715				
3234	0.1623	0.4753	-0.7020	-0.0215	0	Q99J23	GH3 domain-containing protein	Ghdc	mmu:80860				
3235	0.2128	-0.6819	0.4103	-0.0196	0	Q9Z2N8	Actin-like protein 6A	Actl6a	mmu:56456				
3236	[illegible]	0.3638	-0.4165	-0.0177	0	Q8BR63	Protein FAM177A1	Fam177a1	mmu:73385				
3237	-0.5100	0.1917	0.2812	-0.0124	0	Q8K215	LYR motif-containing protein 4	Lyrm4	mmu:380840				
3238	0.3745	-0.4329	0.0256	-0.0109	0	Q80W68	Kin of IRRE-like protein 1	Kirrel	mmu:170643				
3239	0.3869	-0.3216	-0.0955	-0.0101	0	Q63829	COMM domain-containing protein 3	Commd3	mmu:12238				
3240	0.1513	-0.2781	0.1000	-0.0089	0	Q9Z1J3	Cysteine desulfurase, mitochondrial	Nfs1	mmu:18041				
3241	-0.1011	0.2720	-0.1857	-0.0049	0	Q3UVG3	Protein FAM91A1	Fam91a1	mmu:210998				
3242	0.0970	-0.0460	-0.0640	[illegible]	0	Q3U182	CREB-regulated transcription coactivator 2	Crtc2	mmu:74343				
3243	0.1130	-0.1918	0.0684	[illegible]	0	Q921I9	Exosome complex component RRP41	Exosc4	mmu:109075				
3244	0.5859	-0.4115	-0.1777	[illegible]	0	Q9Z1E3	NF-kappa-B inhibitor alpha	Nfkbia	mmu:18035				
3245	-0.1005	-0.5498	0.6587	[illegible]	0	O54950	5-AMP-activated protein kinase subunit gamma-1	Prkag1	mmu:19082				
3246	0.1906	-0.2971	0.1164	[illegible]	0	Q8BMG7	Rab3 GTPase-activating protein non-catalytic subunit	Rab3gap2	mmu:98732				
3247	0.4293	0.0524	-0.4701	[illegible]	0	Q8BGF7	PAB-dependent poly(A)-specific ribonuclease subunit PAN2	Pan2	mmu:103135				
3248	0.6059	-0.2793	-0.3039	0.0076	0	Q8C636	Spermatogenesis-associated protein 16	Spata16	mmu:70862				
3249	-0.0830	-0.4027	0.5089	0.0077	0	O35345	Importin subunit alpha-7;Importin subunit alpha	Kpna6	mmu:16650				
3250	-0.0560	-0.1566	0.2372	0.0082	0	Q99LC9	Peroxisome assembly factor 2	Pex6	mmu:224824				
3251	0.0657	-0.6247	0.5841	0.0084	0	P28474	Alcohol dehydrogenase class-3	Adh5	mmu:11532				
3252	-0.0360	0.1019	-0.0355	0.0101	0	P62313	U6 snRNA-associated Sm-like protein LSm6	Lsm6	mmu:78651				
3253	0.4371	-0.0781	-0.3196	0.0131	0	Q9JK92	Heat shock protein beta-8	Hspb8	mmu:80888				
3254	-0.2148	0.6052	-0.3478	0.0142	0	Q6ZPY2	Protein SMG5	Smg5	mmu:229512				
3255	0.1415	0.1301	-0.2182	0.0178	0	P58242	Acid sphingomyelinase-like phosphodiesterase 3b	Smpdl3b	mmu:100340				
3256	0.3754	-0.9431	0.6375	0.0233	0	Q99KE1	NAD-dependent malic enzyme, mitochondrial	Me2	mmu:107029				
3257	0.1577	0.0633	-0.1442	0.0256	0	Q91YP3	Deoxyribose-phosphate aldolase	Dera	mmu:232449				
3258	0.5311	0.3705	-0.8230	0.0262	0	Q62095	ATP-dependent RNA helicase DDX3Y	Ddx3y	mmu:26900				
3259	0.4813	-0.2477	-0.1449	0.0296	0	Q9CR60	Vesicle transport protein GOT1B	Golt1b	mmu:66964				
3260	-0.0544	-0.0782	0.2239	0.0304	0	Q8BJL1	F-box only protein 30	Fbxo30	mmu:71865			1	
3261	-0.1211	0.0407	0.1748	0.0315	0	A0A1B0GRX3							
3262	-0.0954	-0.2872	0.4803	0.0326	0	Q8R105	Vacuolar protein sorting-associated protein 37C	Vps37c	mmu:107305				
3263	0.2197	-0.0559	-0.0658	0.0327	0	Q8BVI5	Syntaxin-16	Stx16	mmu:228960				
3264	-1.1612	1.4091	-0.1473	0.0335	0	O09061	Proteasome subunit beta type-1	Psmb1	mmu:19170				
3265	-0.1529	0.3205	-0.0613	0.0354	0	P62077	Mitochondrial import inner membrane translocase subunit Tim8 B	Timm8b	mmu:30057				

3266	0.1214	-0.0265	0.0153	0.0367	0	Q9EPK5	WW domain-containing transcription regulator protein 1	Wwtr1	mmu:97064				
3267	-0.2712	0.2405	0.1505	0.0399	0	Q64430	Copper-transporting ATPase 1	Atp7a	mmu:11977	1			
3268	0.0452	0.5691	-0.4936	0.0402	0	Q9D4B1	Phosphatidylcholine:ceramide cholinephosphotransferase 2	Sgms2	mmu:74442				
3269	0.9987	-0.8447	-0.0294	0.0415	0	Q8BGX2	Uncharacterized protein C19orf52 homolog		mmu:69773				
3270	-0.0180	-0.9222	1.0733	0.0444	0	Q9JKC7	AP-4 complex subunit mu-1	Ap4m1	mmu:11781				
3271	0.4382	-0.2092	-0.0932	0.0453	0	Q60989	E3 ubiquitin-protein ligase XIAP	Xiap	mmu:11798				1
3272	-0.0527	-0.0234	0.2124	0.0454	0	Q8R088	Golgi phosphoprotein 3-like	Golph3l	mmu:229593				
3273	-0.4173	0.8463	-0.2903	0.0462	0	Q9Z0E0	Neurochondrin	Ncdn	mmu:26562				
3274	-0.5913	0.6464	0.0906	0.0486	0	P83940	Transcription elongation factor B polypeptide 1	Tceb1	mmu:67923				
3275	0.1855	0.4160	-0.4374	0.0547	0	Q8BMB3	Eukaryotic translation initiation factor 4E type 2	Eif4e2	mmu:26987				
3276	-0.4790	0.9045	-0.2468	0.0596	0	Q9DCR2	AP-3 complex subunit sigma-1	Ap3s1	mmu:11777				
3277	0.7683	0.0772	-0.6652	0.0601	0	Q8CH62	Oral cancer-overexpressed protein 1 homolog	Oraov1	mmu:72284				
3278	-0.2477	0.2425	0.1857	0.0602	0	Q3TYX3	SET and MYND domain-containing protein 5	Smyd5	mmu:232187				
3279	0.3244	0.2629	-0.4058	0.0605	0	Q8K194	U4/U6,U5 small nuclear ribonucleoprotein 27 kDa protein	Snrnp27	mmu:66618				
3280	-0.5828	1.3121	-0.5426	0.0622	0	Q8N7N5	DDB1- and CUL4-associated factor 8	Dcaf8	mmu:98193			1	
3281	-0.2893	0.1843	0.3008	0.0653	0	Q9QZN4	F-box only protein 6	Fbxo6	mmu:50762			1	
3282	-0.1223	0.6400	-0.3216	0.0654	0	Q61207	Prosaposin	Psap	mmu:19156				
3283	-1.0851	0.0750	1.2270	0.0723	0	Q8BHD0	Ras-related protein Rab-39A	Rab39a	mmu:270160				
3284	0.7247	-0.1232	-0.3643	0.0791	0	Q9QUI1	Protein FAM89B	Fam89b	mmu:17826				
3285	0.4399	-0.0517	-0.1449	0.0811	0	Q8VD75	Huntingtin-interacting protein 1	Hip1	mmu:215114				1
3286	-0.0340	0.6977	-0.4092	0.0848	0	Q5XG73	Acyl-CoA-binding domain-containing protein 5	Acbd5	mmu:74159				
3287	-0.2637	0.7758	-0.2503	0.0873	0	Q9CWU9	Nucleoporin Nup37	Nup37	mmu:69736				
3288	-0.2152	0.6931	-0.2151	0.0876	0	Q9CQ54	NADH dehydrogenase [ubiquinone] 1 subunit C2	Ndufc2	mmu:68197				
3289	-0.1309	0.2487	0.1453	0.0877	0	Q8BGB2	Tetratricopeptide repeat protein 7A	Ttc7;Ttc7a	mmu:225049				
3290	1.5450	-1.0745	-0.1974	0.0910	0	Q8BVG4	Dipeptidyl peptidase 9	Dpp9	mmu:224897				
3291	0.0981	0.2548	-0.0398	0.1044	0	P56873	Sjoegren syndrome/scleroderma autoantigen 1 homolog	Sssca1	mmu:56390				
3292	-0.4771	-0.1314	0.9301	0.1072	0	Q9QY93	dCTP pyrophosphatase 1	Dctpp1	mmu:66422				
3293	-0.4814	0.3106	0.4980	0.1091	0	Q922H4	Mannose-1-phosphate guanyltransferase alpha	Gmppa	mmu:69080				
3294	0.5738	-0.1520	-0.0915	0.1101	0	Q9CQH3	NADH dehydrogenase [ubiquinone] 1 beta subcomplex subunit 5, mitochondrial	Ndufb5	mmu:66046				
3295	0.4825	-0.1909	0.0566	0.1161	0	Q9QYK7	RING finger protein 11	Rnf11	mmu:29864				
3296	0.2602	-0.1522	0.2411	0.1164	0	P28741	Kinesin-like protein;Kinesin-like protein KIF3A	Kif3a	mmu:16568				
3297	0.9033	-0.8754	0.3246	0.1175	0	O89023	Tripeptidyl-peptidase 1	Tpp1	mmu:12751				
3298	-0.1530	-0.0060	0.5161	0.1190	0	P0C0A3	Charged multivesicular body protein 6	Chmp6	mmu:208092				
3299	-0.1062	-0.3754	0.8529	0.1238	0	P70280	Vesicle-associated membrane protein 7	Vamp7	mmu:20955				
3300	0.0464	-0.1781	0.5383	0.1355	0	P52875	Transmembrane protein 165	Tmem165	mmu:21982				
3301	0.4616	-0.3005	0.2535	0.1382	0	P35456	Urokinase plasminogen activator surface receptor	Plaur	mmu:18793				
3302	0.2252	[illegible]	0.1941	0.1394	0	Q8K2L8	Trafficking protein particle complex subunit 12	Trappc12	mmu:217449				
3303	0.7333	-0.5804	0.2676	0.1402	0	P68369	Tubulin alpha-1A chain;Tubulin alpha-3 chain	Tuba1a;Tu	mmu:22142				
3304	0.7440	0.1024	-0.4213	0.1417	0	Q9Z1A1		Tfg					
3305	0.8447	0.3813	-0.7993	0.1422	0	Q91ZR1	Ras-related protein Rab-4B	Rab4b	mmu:19342				
3306	-0.4797	0.3976	0.5114	0.1431	0	O35682	Myeloid-associated differentiation marker	Myadm	mmu:50918				
3307	-0.7549	0.6655	0.5372	0.1493	0	Q9QUK4	Baculoviral IAP repeat-containing protein 1b;Baculoviral IAP repeat-containing protein 1g;Baculov	Naip2;Naip	mmu:17948				
3308	0.3846	0.1785	-0.0861	0.1590	0	Q9R0X0	Mediator of RNA polymerase II transcription subunit 20	Gm20517;I	mmu:56771				
3309	0.2867	-0.0646	0.2609	0.1610	0	Q61521	Proepiregulin;Epiregulin	Ereg	mmu:13874				
3310	-0.0859	-0.1724	0.7468	0.1628	0	Q9D1M4	Eukaryotic translation elongation factor 1 epsilon-1	Eef1e1	mmu:66143				
3311	-0.4444	0.7200	0.2168	0.1641	0	Q64437	Alcohol dehydrogenase class 4 mu/sigma chain	Adh7	mmu:11529				
3312	0.8845	-0.6723	0.2859	0.1660	0	Q8K2Q0	COMM domain-containing protein 9	Commd9	mmu:76501				
3313	1.0096	-0.5155	0.0048	0.1663	0	Q8BH58	TIP41-like protein	Tiprl	mmu:226591				
3314	-0.7426	0.7776	0.4703	0.1684	0	Q9CQC5	Cdc42 effector protein 3	Cdc42ep3	mmu:260409				
3315	0.3350	0.4456	-0.2750	0.1685	0	P62311	U6 snRNA-associated Sm-like protein LSm3	Lsm3	mmu:67678				
3316	-0.9802	0.4375	1.0612	0.1728	0	P01887	Beta-2-microglobulin	B2m	mmu:12010				
3317	1.3067	-0.6879	-0.0902	0.1762	0	Q7TN31	Angiogenic factor with G patch and FHA domains 1	Aggf1	mmu:66549				
3318	0.0747	-0.1158	0.5707	0.1765	0	Q9CQ56	Vesicle transport protein USE1	Use1	mmu:67023				
3319	-0.1248	0.5755	0.1086	0.1864	0	Q05816	Fatty acid-binding protein, epidermal	Fabp5	mmu:16592				
3320	-0.0339	0.1478	0.4469	0.1869	0	P08030	Adenine phosphoribosyltransferase	Aprt	mmu:11821				
3321	0.9553	0.0379	-0.4267	0.1888	0	F7BE84		Ankrd50					
3322	0.8680	-0.0328	-0.2525	0.1942	0	Q6TYB5	Fasciculation and elongation protein zeta-2	Fez2	mmu:225020				
3323	1.7770	-0.1315	-1.0564	0.1964	0	Q7M759	Alpha/beta hydrolase domain-containing protein 17B	Abhd17b	mmu:226016				
3324	-0.0445	0.8883	-0.2546	0.1964	0	Q8BGT6	MICAL-like protein 1	Micall1	mmu:27008				
3325	0.7711	-0.2557	0.0824	0.1993	0	Q6PHN9	Ras-related protein Rab-35	Rab35	mmu:77407				
3326	-0.1951	0.3140	0.4956	0.2048	0	Q9CQS8	Protein transport protein Sec61 subunit beta	Sec61b;Gm	mmu:66212				
3327	0.3510	0.8351	-0.5708	0.2051	0	Q8VE99	Coiled-coil domain-containing protein 115	Ccdc115	mmu:69668				
3328	0.4598	-0.4850	0.6425	0.2058	0	Q8CBY8	Dynactin subunit 4	Dctn4	mmu:67665				
3329	0.6761	-0.2498	0.1963	0.2075	0	Q80WT5	Aftiphilin	Aftph	mmu:216549				
3330	-0.1333	1.0141	-0.2573	0.2078	0	Q8BQZ4	Ral GTPase-activating protein subunit beta	Ralgapb					
3331	0.1561	-0.1760	0.6616	0.2139	0	O35625	Axin-1	Axin1	mmu:12005				
3332	0.8014	0.4965	-0.6518	0.2154	0	Q9JIQ3	Diablo homolog, mitochondrial	Diablo	mmu:66593	1			1
3333	-0.5779	0.4088	0.8159	0.2156	0	P54116	Erythrocyte band 7 integral membrane protein	Stom	mmu:13830				
3334	-0.3318	0.2417	0.7376	0.2158	0	Q8BZM1	Glomulin	Glmn	mmu:170823				
3335	0.5601	0.1586	-0.0538	0.2216	0	Q80X85	28S ribosomal protein S7, mitochondrial	Mrps7	mmu:50529				
3336	-0.2471	0.4867	0.4320	0.2239	0	Q8K1A0	Methyltransferase-like protein 5	Mettl5	mmu:75422				
3337	0.1491	0.6138	-0.0634	0.2332	0	Q8BTY2	Anion exchange protein;Sodium bicarbonate cotransporter 3	Slc4a7					
3338	0.3647	0.8834	-0.5428	0.2351	0	Q9JKF7	39S ribosomal protein L39, mitochondrial	Mrpl39	mmu:27393				
3339	0.9502	-0.0919	-0.1530	0.2351	0	O35740	Cbp/p300-interacting transactivator 2	Cited2	mmu:17684				
3340	-0.4025	1.4748	-0.3654	0.2356	0	Q921W0	Charged multivesicular body protein 1a	Chmp1a	mmu:234852				
3341	0.4856	0.2807	-0.0453	0.2403	0	B2RXC1	Trafficking protein particle complex subunit 11	Trappc11	mmu:320714				
3342	0.7748	0.6959	-0.7426	0.2427	0	Q9D8U0		Ifrd2	mmu:15983				
3343	-0.0182	0.4231	0.3255	0.2435	0	Q9D937	Uncharacterized protein C11orf98 homolog		mmu:66276				
3344	0.2303	-0.0656	0.5867	0.2505	0	Q8BP78	Protein FRA10AC1 homolog	Fra10ac1	mmu:70567				
3345	0.9179	-3.0744	2.9101	0.2512	0	Q99M74	Keratin, type II cuticular Hb2	Krt82	mmu:114566				
3346	-0.0956	0.3118	0.5414	0.2525	0	Q3USZ8	Deleted in autism protein 1 homolog		mmu:68861				
3347	-0.4770	0.2735	0.9723	0.2563	0	Q9CX00	IST1 homolog	Ist1	mmu:71955				
3348	-0.0934	0.0820	0.7833	0.2573	0	Q9EQS3	C-Myc-binding protein	Mycbp	mmu:56309				
3349	1.6457	-0.7180	-0.1527	0.2583	0	Q8K183	Pyridoxal kinase	Pdxk	mmu:216134				
3350	0.5466	-0.3202	0.5662	0.2642	0	Q9EQW7	Kinesin-like protein;Kinesin-like protein KIF13A	Kif13a					
3351	-0.1751	0.4104	0.5642	0.2665	0	A2AN61		Ciz1					
3352	1.0397	-0.8572	0.6229	0.2685	0	P35285	Ras-related protein Rab-22A	Rab22a	mmu:19334				
3353	1.1055	-0.1327	-0.1650	0.2693	0	Q8K211	High affinity copper uptake protein 1	Slc31a1	mmu:20529				
3354	0.6027	-0.2588	0.4868	0.2769	0	P09528	Ferritin heavy chain;Ferritin heavy chain, N-terminally processed	Fth1	mmu:14319	1			
3355	-0.5349	-0.3036	1.6706	0.2774	0	Q8BGH7	CDC42 small effector protein 2	Cdc42se2	mmu:72729				
3356	0.3934	-0.6208	1.0661	0.2796	0	Q8VE70	Programmed cell death protein 10	Pdcd10	mmu:56426	1			
3357	0.6634	-0.0070	0.1845	0.2803	0	Q08639	Transcription factor Dp-1;Transcription factor Dp-2	Tfdp1;Tfdp	mmu:21781				
3358	0.2344	0.8340	-0.2185	0.2833	0	Q8K0F1	TBC1 domain family member 23	Tbc1d23	mmu:67581				
3359	0.3092	-0.0283	0.5776	0.2862	0	Q64314	Hematopoietic progenitor cell antigen CD34	Cd34	mmu:12490				
3360	0.6466	-0.2910	0.5043	0.2866	0	Q99M54	Cell division cycle-associated protein 3	Cdca3	mmu:14793			1	
3361	0.8156	1.0232	-0.9727	0.2887	0	Q9CQE5	Regulator of G-protein signaling 10	Rgs10	mmu:67865				
3362	0.3981	0.5209	-0.0424	0.2922	0	Q8CDA1	Phosphatidylinositide phosphatase SAC2	Inpp5f	mmu:101490		1		
3363	0.1404	1.2196	-0.4701	0.2966	0	Q80TY0	Formin-binding protein 1	Fnbp1	mmu:14269				
3364	0.1055	0.9472	-0.1530	0.2999	0	Q99LX5	Multiple myeloma tumor-associated protein 2 homolog	Mmtag2	mmu:67862				
3365	-0.3154	1.4799	-0.2481	0.3055	0	Q8BQ46		Taf15	mmu:70439				
3366	0.2799	0.7012	-0.0644	0.3056	0	O35604	Niemann-Pick C1 protein	Npc1	mmu:18145				
3367	-0.5444	0.5515	0.9336	0.3136	0	Q8BL99	Protein dopey-1	Dopey1					
3368	-0.4017	0.5480	0.8074	0.3179	0	O08917	Flotillin-1	Flot1	mmu:14251				
3369	-0.0567	1.2635	-0.2062	0.3335	0	Q6PD26	GPI transamidase component PIG-S	Pigs	mmu:276846				
3370	0.4744	0.5915	-0.0636	0.3341	0	D3Z3J6		Paip1					
3371	1.6853	-0.7752	0.0944	0.3348	0	Q8BJ34	Meiosis arrest female protein 1	Marf1	mmu:223989				
3372	0.9994	-0.0584	0.0731	0.3380	0	Q9D1E6	Tubulin-folding cofactor B	Tbcb	mmu:66411				
3373	0.8896	0.7588	-0.6289	0.3398	0	Q920I9	WD repeat-containing protein 7	Wdr7	mmu:104082				
3374	0.7692	-0.5271	0.7855	0.3425	0	Q9D8L5	Coiled-coil domain-containing protein 91	Ccdc91	mmu:67015				
3375	-0.2661	-0.3607	1.6717	0.3483	0	Q3TXT3	SOSS complex subunit C	Inip	mmu:66209				
3376	1.0996	-0.6040	0.5535	0.3497	0	P40201	Chromodomain-helicase-DNA-binding protein 1	Chd1	mmu:12648				
3377	-0.1604	-0.1565	1.4038	0.3623	0	E9Q7G1		Tmed7					
3378	-0.3750	-0.3370	1.8023	0.3634	0	Q6VN19	Ran-binding protein 10	Ranbp10	mmu:74334				
3379	-0.1253	0.8462	0.3783	0.3664	0	Q8C180	Fibroblast growth factor receptor substrate 2	Frs2	mmu:327826				
3380	-0.5947	-0.0133	1.7104	0.3675	0	Q9DBS1	Transmembrane protein 43	Tmem43	mmu:74122				
3381	0.0063	-0.1443	1.2509	0.3710	0	Q8BV13	COP9 signalosome complex subunit 7b	Cops7b	mmu:26895				
3382	-0.3119	0.4057	1.0239	0.3726	0	Q8K5B2	Multiple coagulation factor deficiency protein 2 homolog	Mcfd2	mmu:193813				
3383	-0.1381	0.2530	1.0122	0.3757	0	Q8BG05	Heterogeneous nuclear ribonucleoprotein A3	Hnrnpa3	mmu:229279				
3384	0.5735	-0.5422	1.0986	0.3766	0	Q9CS42	Ribose-phosphate pyrophosphokinase 2;Ribose-phosphate pyrophosphokinase 1	Prps2;Prps	mmu:110639				
3385	-0.9148	1.4128	0.6350	0.3777	0	P62774	Myotrophin	Mtpn	mmu:14489				
3386	1.1846	0.9685	-1.0133	0.3799	0	Q9CWD8	Iron-sulfur protein NUBPL	Nubpl	mmu:76826				
3387	-0.5248	0.3983	1.3114	0.3950	0	O55125	Protein NipSnap homolog 1	Nipsnap1	mmu:18082				
3388	0.2659	1.1289	-0.2099	0.3950	0	Q922X9	Protein arginine N-methyltransferase 7	Prmt7	mmu:214572				
3389	1.3814	0.1082	-0.2864	0.4011	0	Q3UUX7	Uncharacterized protein C4orf46 homolog		mmu:75939				
3390	0.0253	-0.0460	1.2366	0.4053	0	Q91WE1	Sorting nexin-15	Snx15	mmu:69024				
3391	0.8277	0.6296	-0.2042	0.4177	0	P23492	Purine nucleoside phosphorylase	Pnp;Pnp2	mmu:18950				

3392	0.9790	0.4675	-0.1753	0.4237	0	Q9D5V6	Synapse-associated protein 1	Syap1	mmu:67043				
3393	0.8919	1.1442	-0.6722	0.4546	0	Q7TPS5	C2 domain-containing protein 5	C2cd5	mmu:74741				
3394	1.7153	0.1432	-0.4942	0.4548	0	Q8R0S2	IQ motif and SEC7 domain-containing protein 1	Iqsec1	mmu:232227				
3395	-0.2613	1.0058	0.6532	0.4659	0	P35288	Ras-related protein Rab-23	Rab23					
3396	0.5853	-0.3757	1.1936	0.4677	0	Q01721	Growth arrest-specific protein 1	Gas1	mmu:14451				
3397	0.9840	0.4937	-0.0517	0.4753	0	Q5SSH7	Zinc finger ZZ-type and EF-hand domain-containing protein 1	Zzef1	mmu:195018				
3398	0.2628	1.5080	-0.3289	0.4806	0	Q9CXU1	Mediator of RNA polymerase II transcription subunit 31	Med31	mmu:67279				
3399	2.5236	-1.0051	-0.0414	0.4924	0	Q8CDJ8	Stonin-1	Ston1	mmu:77057				
3400	0.7792	0.9696	-0.2693	0.4932	0	Q9JHF5	V-type proton ATPase subunit a	Tcirg1	mmu:27060				
3401	-0.3815	0.1104	1.7881	0.5057	0	Q80UP5	Ankyrin repeat domain-containing protein 13A	Ankrd13a	mmu:68420				
3402	1.7657	0.1002	-0.3241	0.5139	0	Q5SV85	Synergin gamma	Synrg	mmu:217030				
3403	2.0802	-0.0752	-0.4494	0.5185	0	Q6PEB6	MOB-like protein phocein	Mob4	mmu:19070				
3404	1.5589	-0.2619	0.2662	0.5211	0	Q8C0L8	Conserved oligomeric Golgi complex subunit 5	Cog5	mmu:238123				
3405	1.4088	0.2317	-0.0729	0.5225	0	Q9WUA3	ATP-dependent 6-phosphofructokinase;ATP-dependent 6-phosphofructokinase, platelet type	Pfkp	mmu:56421				
3406	-0.0690	0.7665	0.8957	0.5311	0	Q8BJL0	SWI/SNF-related matrix-associated actin-dependent regulator of chromatin subfamily A-like prote	Smarcal1	mmu:54380				
3407	0.8706	-0.3162	1.0391	0.5312	0	Q99MV1	Tudor domain-containing protein 1	Tdrd1	mmu:83561				
3408	-0.3652	1.0982	0.8631	0.5320	0	Q9JJK5	Homocysteine-responsive endoplasmic reticulum-resident ubiquitin-like domain member 1 protei	Herpud1	mmu:64209				
3409	0.0049	0.6484	0.9529	0.5321	0	Q9QXS1	Plectin	Plec	mmu:18810				
3410	1.1891	0.6610	-0.1930	0.5524	0	P97808	FXYD domain-containing ion transport regulator 5	Fxyd5	mmu:18301				
3411	1.4427	-0.3887	0.6673	0.5738	0	Q7TPV2	E3 ubiquitin-protein ligase DZIP3	Dzip3	mmu:224170			1	
3412	0.3776	1.5053	-0.1290	0.5846	0	Z4YLD0	UPF0428 protein CXorf56 homolog	C330007P06Rik					
3413	1.1348	-0.1777	0.8067	0.5879	0	Q9DCI9	39S ribosomal protein L32, mitochondrial	Mrpl32	mmu:75398				
3414	-2.0350	2.3740	1.4538	0.5976	0	P17095	High mobility group protein HMG-I/HMG-Y	Hmga1	mmu:15361				
3415	-0.2020	0.6710	1.3384	0.6025	0	Q6A009	E3 ubiquitin-protein ligase listerin	Ltn1	mmu:78913			1	
3416	2.5739	0.4123	-1.0598	0.6421	0	Q8C436	Protein-lysine methyltransferase METTL21D	Vcpkmt	mmu:207965				
3417	1.9053	0.0939	-0.0580	0.6471	0	Q6J756	Calpain-11	Capn11	mmu:268958				
3418	0.0409	3.0805	-1.1343	0.6624	0	P61028	Ras-related protein Rab-8B	Rab8b	mmu:235442				
3419	1.9899	-0.6138	0.6709	0.6823	0	Q9CQ25	Mitotic-spindle organizing protein 2	Mzt2	mmu:72083				
3420	-0.2003	1.4750	0.7941	0.6896	0	Q9EQN9		Slc19a2	mmu:116914				
3421	2.2129	0.1004	-0.2402	0.6910	0	Q9D0M0	Exosome complex exonuclease RRP42	Exosc7	mmu:66446				
3422	0.8904	1.2941	-0.0749	0.7032	0	Q8CI61	BAG family molecular chaperone regulator 4	Bag4	mmu:67384				1
3423	2.3625	0.4358	-0.6034	0.7316	0	Q9CR88	28S ribosomal protein S14, mitochondrial	Mrps14	mmu:64659				
3424	4.0429	-0.6703	-1.1300	0.7475	0	Q99LG4	Tetratricopeptide repeat protein 5	Ttc5	mmu:219022				
3425	0.8224	1.8209	-0.4006	0.7476	0	Q8K212	Phosphofurin acidic cluster sorting protein 1	Pacs1	mmu:107975				
3426	1.5071	1.1567	-0.3353	0.7762	0	Q91WR3	Activating signal cointegrator 1 complex subunit 2	Ascc2	mmu:75452				
3427	2.3378	-0.2345	0.4494	0.8509	0	Q3UVK0	Endoplasmic reticulum metallopeptidase 1	Ermp1	mmu:226090				
3428	2.0419	-0.4099	1.0208	0.8843	0	Q9JM96	Cdc42 effector protein 4	Cdc42ep4	mmu:56699				
3429	1.3255	2.5568	-1.2204	0.8873	0	P59672	Ankyrin repeat and SAM domain-containing protein 1A	Anks1;Ank	mmu:224650				
3430	1.5516	1.3092	-0.0946	0.9221	0	Q9D787	Peptidyl-prolyl cis-trans isomerase-like 2	Ppil2	mmu:66053			1	
3431	2.1664	1.6817	-0.8667	0.9938	0	P62484	Abl interactor 2	Abi2	mmu:329165				
3432	3.8168	-1.7036	0.9381	1.0171	0	Q60778	NF-kappa-B inhibitor beta	Nfkbib	mmu:18036				
3433	1.2100	4.2681	-2.4122	1.0220	0	P45377	Aldose reductase-related protein 2;Aldose reductase-related protein 1	Akr1b8;Ak	mmu:14187				
3434	4.2303	0.1370	-1.2647	1.0342	0	O70472	Transmembrane protein 131	Tmem131	mmu:56030				
3435	-0.4605	1.7482	1.8552	1.0476	0	P16627	Heat shock 70 kDa protein 1-like	Hspa1l	mmu:15482				
3436	2.5988	1.1973	-0.4715	1.1082	0	Q8BQR4	KAT8 regulatory NSL complex subunit 2	Kansl2	mmu:69612				
3437	3.5348	-0.0540	0.2985	1.2598	0	Q9D289	Trafficking protein particle complex subunit 6B	Trappc6b	mmu:78232				
3438	3.9423	0.6691	-0.6836	1.3093	0	P06745	Glucose-6-phosphate isomerase	Gpi	mmu:14751				
3439	4.1014	-0.2749	1.6405	1.8223	0	Q8C166	Copine-1	Cpne1	mmu:266692				
3440	-0.6014	NaN	NaN	-1.5529	0	Q566J8	AarF domain-containing protein kinase 4	Adck4		1			
3441	-0.8059	NaN	NaN	-0.9887	0	Q62388	Serine-protein kinase ATM	Atm	mmu:11920	1			
3442	-0.9671	NaN	NaN	-0.9671	0	P22518	Dual specificity protein kinase CLK1	Clk1	mmu:12747	1			
3443	-0.8695	NaN	NaN	-0.8695	0	Q8CBQ5	Phosphatidylinositol 4-kinase type 2-beta	Pi4k2b	mmu:67073	1			
3444	0.0476	-0.3686	NaN	-0.8043	0	O55028	[3-methyl-2-oxobutanoate dehydrogenase [lipoamide]] kinase, mitochondrial	Bckdk	mmu:12041	1			
3445	-0.4403	-1.0120	NaN	-0.7262	0	Q9JHU3	Cyclin-dependent kinase 20	Cdk20	mmu:105278	1			
3446	NaN	-0.6554	NaN	-0.6554	0	P26618	Platelet-derived growth factor receptor alpha	Pdgfra	mmu:18595	1	1		
3447	-0.5290	NaN	NaN	-0.5290	0	Q62407	Striated muscle-specific serine/threonine-protein kinase	Speg	mmu:11790	1			
3448	NaN	NaN	NaN	-0.5287	0	Q640Q5	PAB-dependent poly(A)-specific ribonuclease subunit PAN3	Pan3	mmu:72587	1			
3449	NaN	-0.2807	-0.7922	-0.5263	0	P0C5K0	Uncharacterized serine/threonine-protein kinase SBK3	Sbk3	mmu:381835	1			
3450	-0.4818	NaN	NaN	-0.4818	0	Q6J9G1	Tyrosine-protein kinase STYK1	Styk1	mmu:243659	1			
3451	-0.5082	NaN	NaN	-0.3996	0	Q62312	TGF-beta receptor type-2	Tgfbr2	mmu:21813	1			
3452	0.0642	NaN	0.4463	-0.3759	0	Q07832	Serine/threonine-protein kinase PLK1	Plk1	mmu:18817	1			
3453	NaN	-0.0363	-0.6508	-0.3435	0	Q9DBU3	Serine/threonine-protein kinase RIO3	Riok3	mmu:66878	1			
3454	-0.9628	NaN	0.3756	-0.3024	0	O70126	Aurora kinase B	Aurkb	mmu:20877	1			
3455	-0.5043	NaN	NaN	-0.2715	0	O55047	Serine/threonine-protein kinase tousled-like 2	Tlk2	mmu:24086	1			
3456	NaN	NaN	-0.2368	-0.2368	0	P70424	Receptor tyrosine-protein kinase erbB-2;Receptor tyrosine-protein kinase erbB-4;ERBB4 intracellul	Erbb2;Erbl	mmu:13866	1			
3457	NaN	-0.2246	NaN	-0.2246	0	Q99MK8	Beta-adrenergic receptor kinase 1;Beta-adrenergic receptor kinase 2	Adrbk1;Ad	mmu:110355	1			
3458	0.3070	-0.7220	NaN	-0.2075	0	O35099	Mitogen-activated protein kinase kinase kinase 5	Map3k5	mmu:26408	1	1		1
3459	NaN	-0.0681	[illegible]	-0.2014	0	Q9Z2A0	3-phosphoinositide-dependent protein kinase 1	Pdpk1	mmu:18607	1			
3460	-0.1240	NaN	-1.3324	-0.1389	0	Q69ZA1	Cyclin-dependent kinase 13	Cdk13	mmu:69562	1			
3461	NaN	NaN	0.3258	-0.1349	0	O54784	Death-associated protein kinase 3	Dapk3	mmu:13144	1			1
3462	NaN	NaN	-0.1244	-0.1244	0	O35280	Serine/threonine-protein kinase Chk1	Chek1	mmu:12649	1			
3463	NaN	-0.0635	-0.1555	-0.1095	0	P54763	Ephrin type-B receptor 2;Ephrin type-B receptor 1	Ephb2;Eph	mmu:13844	1			
3464	0.0084	NaN	0.1402	-0.0836	0	Q05512	Serine/threonine-protein kinase MARK2	Mark2	mmu:13728	1			
3465	-0.5419	NaN	NaN	-0.0677	0	Q80X41	Serine/threonine-protein kinase VRK1	Vrk1	mmu:22367	1			
3466	NaN	0.2862	-0.3746	-0.0442	0	Q60823	RAC-beta serine/threonine-protein kinase	Akt2	mmu:11652	1			
3467	-0.4382	0.5891	NaN	-0.0399	0	P70182	Phosphatidylinositol 4-phosphate 5-kinase type-1 alpha	Pip5k1a	mmu:18720	1			
3468	-0.1778	NaN	NaN	-0.0256	0	Q9JKK8	Serine/threonine-protein kinase ATR	Atr		1			
3469	-0.1148	0.0861	NaN	-0.0144	0	Q3UUJ4	STE20-related kinase adapter protein alpha	Strada	mmu:72149	1			
3470	NaN	-0.0313	NaN	0.0098	0	Q61532	Mitogen-activated protein kinase 6	Mapk6	mmu:50772	1			
3471	0.0351	NaN	NaN	0.0351	0	P97820	Mitogen-activated protein kinase kinase kinase kinase 4	Map4k4	mmu:26921	1			
3472	NaN	-0.6058	0.4039	0.0408	0	Q8C078	Calcium/calmodulin-dependent protein kinase kinase 2	Camkk2	mmu:207565	1			
3473	NaN	0.2077	-0.0191	0.0943	0	Q99PW4	TP53-regulating kinase	2810408M	mmu:76367	1			
3474	-0.5852	NaN	1.1031	0.0950	0	Q03141	MAP/microtubule affinity-regulating kinase 3	Mark3	mmu:17169	1			
3475	NaN	0.3843	-1.2873	0.1003	0	A2CG49	Kalirin	Kalrn	mmu:545156	1			
3476	0.3773	NaN	NaN	0.1315	0	Q14AX6	Cyclin-dependent kinase 12	Cdk12	mmu:69131	1			
3477	NaN	NaN	0.2497	0.2497	0	Q99N57	RAF proto-oncogene serine/threonine-protein kinase	Raf1	mmu:110157	1			
3478	0.2568	NaN	NaN	0.2568	0	Q61161	Mitogen-activated protein kinase kinase kinase kinase 2	Map4k2	mmu:26412	1			
3479	0.2639	NaN	NaN	0.2639	0	Q811L6	Microtubule-associated serine/threonine-protein kinase 4	Mast4	mmu:328329	1			
3480	0.2764	NaN	NaN	0.2764	0	Q04735	Cyclin-dependent kinase 16	Cdk16	mmu:18555	1			
3481	0.2880	NaN	NaN	0.2880	0	P97477	Aurora kinase A	Aurka	mmu:20878	1			
3482	NaN	0.3025	NaN	0.3025	0	P54754	Ephrin type-B receptor 3	Ephb3	mmu:13845	1			
3483	-0.4888	NaN	0.4584	0.3037	0	Q91Z96	BMP-2-inducible protein kinase	Bmp2k	mmu:140780	1			
3484	NaN	0.4237	0.1918	0.3078	0	O88351	Inhibitor of nuclear factor kappa-B kinase subunit beta	Ikbkb	mmu:16150	1			
3485	-0.0357	NaN	NaN	0.3088	0	Q62406	Interleukin-1 receptor-associated kinase 1	Irak1	mmu:16179	1			
3486	0.3544	NaN	NaN	0.3544	0	P16092	Fibroblast growth factor receptor;Fibroblast growth factor receptor 1;Fibroblast growth factor rec	Fgfr1;Fgfr3	mmu:14182	1			
3487	NaN	0.3765	NaN	0.3765	0	Q9QVP9	Protein-tyrosine kinase 2-beta	Ptk2b	mmu:19229	1	1		
3488	1.3180	NaN	NaN	0.4696	0	Q9JM52	Misshapen-like kinase 1	Mink1	mmu:50932	1			
3489	NaN	0.5527	0.4875	0.5266	0	Q62074	Protein kinase C iota type	Prkci	mmu:18759	1			
3490	0.5457	NaN	NaN	0.5457	0	Q8BG48	Serine/threonine-protein kinase 17B	Stk17b	mmu:98267	1			1
3491	NaN	0.2188	0.8958	0.5573	0	O35495	Cyclin-dependent kinase 14	Cdk14	mmu:18647	1			
3492	0.7125	NaN	NaN	0.7125	0	Q9Z2W1	Serine/threonine-protein kinase 25	Stk25	mmu:59041	1	1		
3493	0.7363	NaN	NaN	0.7363	0	Q9WTU6	Mitogen-activated protein kinase 9	Mapk9	mmu:26420	1	1		
3494	1.1845	NaN	0.3128	0.7487	0	Q9Z277	Tyrosine-protein kinase BAZ1B	Baz1b	mmu:22385	1			
3495	0.3465	NaN	0.7949	0.8513	0	Q5S006	Leucine-rich repeat serine/threonine-protein kinase 2	Lrrk2	mmu:66725	1	1		
3496	0.7515	-0.0544	NaN	0.8544	0	Q80XI4	Phosphatidylinositol 5-phosphate 4-kinase type-2 beta	Pip4k2b	mmu:108083	1			
3497	-4.8140	NaN	NaN	-4.8140	0	Q99MJ9	ATP-dependent RNA helicase DDX50	Ddx50	mmu:94213				
3498	-4.3444	NaN	NaN	-4.3444	0	Q3UZ01	RNA-binding protein 40	Rnpc3	mmu:67225				
3499	-4.3191	NaN	NaN	-4.3191	0	Q8BUH8	Sentrin-specific protease 7	Senp7	mmu:66315				
3500	NaN	NaN	-0.3139	-3.4357	0	Q93092	Transaldolase	Taldo1	mmu:21351				
3501	-3.0861	NaN	NaN	-3.0861	0	Q8K2J7	RELT-like protein 1	Rell1	mmu:100532				
3502	NaN	NaN	NaN	-2.9963	0	Q8CEC6	Peptidylprolyl isomerase domain and WD repeat-containing protein 1	Ppwd1	mmu:238831				
3503	-2.9455	NaN	NaN	-2.9455	0	P54099	DNA polymerase subunit gamma-1	Polg					
3504	-2.8825	NaN	NaN	-2.8825	0	Q9CZT4	DNA-directed RNA polymerase III subunit RPC5	Polr3e	mmu:26939				
3505	-4.1861	-1.5071	NaN	-2.8466	0	Q9ESK9	RB1-inducible coiled-coil protein 1	Rb1cc1	mmu:12421				
3506	-4.1492	NaN	NaN	-2.6420	0	P34056	Transcription factor AP-2-alpha;Transcription factor AP-2-epsilon;Transcription factor AP-2-beta	Tfap2a;Tfa	mmu:21418				
3507	NaN	1.1271	-6.3816	-2.6273	0	P97314	Cysteine and glycine-rich protein 2	Csrp2	mmu:13008				
3508	-2.6153	NaN	NaN	-2.6153	0	Q8C4J7	Transducin beta-like protein 3	Tbl3	mmu:213773				
3509	NaN	-2.5659	NaN	-2.5659	0	P62492	Ras-related protein Rab-11A	Rab11a	mmu:53869				
3510	-0.8033	NaN	NaN	-2.4415	0	E9PYL2		Prr12	mmu:233210				
3511	0.2915	NaN	-1.4432	-2.4262	0	Q6PAQ4	RNA exonuclease 4	Rexo4	mmu:227656				
3512	NaN	-2.5191	-1.9645	-2.2418	0	Q8CB62	Centrobin	Cntrob	mmu:216846				
3513	NaN	NaN	-2.1184	-2.1184	0	Q8R332	Nucleoporin p58/p45	Nupl1	mmu:71844				
3514	-1.9825	NaN	NaN	-1.9825	0	P98192	Dihydroxyacetone phosphate acyltransferase	Gnpat	mmu:14712				
3515	-1.8917	NaN	NaN	-1.9795	0	Q9CQX4	PCNA-associated factor	Paf	mmu:68026				
3516	-0.0999	NaN	NaN	-1.9210	0	Q9CQ75	NADH dehydrogenase [ubiquinone] 1 alpha subcomplex subunit 2	Ndufa2	mmu:17991				
3517	NaN	-1.9157	NaN	-1.9157	0	P85094	Isochorismatase domain-containing protein 2A, mitochondrial	Isoc2a	mmu:664994				

3518	NaN	NaN	NaN	-1.8664	0	Q9CRD2	ER membrane protein complex subunit 2	Emc2	mmu:66736				
3519	NaN	NaN	NaN	-1.8591	0	Q99L20		Gstt3	mmu:103140				
3520	NaN	NaN	NaN	-1.8080	0	Q3UZ24		Apol7e;Ap	mmu:666348				
3521	NaN	-1.5020	0.3737	-1.8062	0	Q80VJ2	Steroid receptor RNA activator 1	Sra1	mmu:24068				
3522	-1.7948	NaN	NaN	-1.7948	0	Q8R322	Nucleoporin GLE1	Gle1	mmu:74412				
3523	NaN	NaN	NaN	-1.7779	0	A0A0A6YXM5		Rcor3					
3524	-0.2471	NaN	NaN	-1.7597	0	Q80U63	Mitofusin-2	Mfn2	mmu:170731				
3525	-1.1277	NaN	NaN	-1.7321	0	E9Q7E2		Arid2	mmu:77044				
3526	NaN	-1.6229	-1.7779	-1.7004	0	Q6Q477	Calcium-transporting ATPase	Atp2b4	mmu:381290				
3527	-1.6784	NaN	NaN	-1.6784	0	Q8CIE2	Zinc finger MIZ domain-containing protein 2	Zmiz2	mmu:52915				
3528	-1.6662	NaN	NaN	-1.6662	0	Q80Y44	Probable ATP-dependent RNA helicase DDX10	Ddx10	mmu:77591				
3529	-1.2544	NaN	-1.9014	-1.5779	0	P70261	Paladin	Pald1	mmu:27355		1		
3530	NaN	-1.9972	-1.1212	-1.5592	0	Q7TMK6	Protein Hook homolog 2	Hook2	mmu:170833				
3531	NaN	-1.5587	NaN	-1.5587	0	Q8BGR8	GSK3-beta interaction protein	Gskip	mmu:66787				
3532	-1.5442	NaN	NaN	-1.5442	0	Q7TQC7-2							
3533	-1.0234	NaN	NaN	-1.5281	0	Q8R3C6	Probable RNA-binding protein 19	Rbm19	mmu:74111				
3534	NaN	-1.6775	-1.3181	-1.4978	0	Q9CWR1	WD repeat-containing protein 73	Wdr73	mmu:71968				
3535	NaN	-4.5618	2.7717	-1.4883	0	P10605	Cathepsin B;Cathepsin B light chain;Cathepsin B heavy chain	Ctsb	mmu:13030				
3536	-1.4847	NaN	NaN	-1.4847	0	Q6Y5D8	Rho GTPase-activating protein 10	Arhgap10	mmu:78514				
3537	-2.4107	NaN	NaN	-1.4804	0	Q9D2R6	Cytochrome c oxidase assembly factor 3 homolog, mitochondrial	Coa3	mmu:52469				
3538	-1.4733	NaN	NaN	-1.4733	0	Q9DBZ1	Inhibitor of nuclear factor kappa-B kinase-interacting protein	Ikbip	mmu:67454				
3539	-0.0115	-0.4714	NaN	-1.4398	0	Q3TN34	MICAL-like protein 2	Micall2	mmu:231830				1
3540	-0.6933	NaN	NaN	-1.4341	0	P63013	Paired mesoderm homeobox protein 1	Prrx1	mmu:18933				
3541	NaN	NaN	-1.4128	-1.4128	0	Q3UDP0	WD repeat-containing protein 41	Wdr41	mmu:218460				
3542	NaN	-1.6122	-1.1886	-1.4004	0	Q91WD5	NADH dehydrogenase [ubiquinone] iron-sulfur protein 2, mitochondrial	Ndufs2	mmu:226646	1			
3543	-1.3860	NaN	NaN	-1.3860	0	Q6DVA0	LEM domain-containing protein 2	Lemd2	mmu:224640				
3544	NaN	NaN	NaN	-1.3802	0	Q99JT1	Glutamyl-tRNA(Gln) amidotransferase subunit B, mitochondrial	Gatb	mmu:229487				
3545	-1.3500	NaN	NaN	-1.3500	0	Q8K396	Meiotic nuclear division protein 1 homolog	Mnd1	mmu:76915				
3546	-1.3385	NaN	NaN	-1.3385	0	Q6PCM2	Integrator complex subunit 6	Ints6	mmu:18130				
3547	-1.3234	NaN	NaN	-1.3234	0	Q0VDT2	Zinc finger protein 367	Znf367	mmu:238673				
3548	-1.3228	NaN	NaN	-1.3228	0	Q68FH0	Plakophilin-4	Pkp4	mmu:227937				
3549	NaN	NaN	-0.8828	-1.3140	0	Q80TA1	Ethanolaminephosphotransferase 1	Ept1	mmu:28042				
3550	-1.3115	NaN	NaN	-1.3115	0	Q8BGY7	Protein FAM210A	Fam210a	mmu:108654				
3551	NaN	-1.1196	-1.4849	-1.3023	0	P09925	Surfeit locus protein 1	Surf1	mmu:20930				
3552	-1.6577	NaN	NaN	-1.2935	0	Q5DW34	Histone-lysine N-methyltransferase EHMT1	Ehmt1	mmu:77683				
3553	-1.3044	NaN	-1.9841	-1.2912	0	Q8K284	General transcription factor 3C polypeptide 1	Gtf3c1	mmu:233863				
3554	-1.2894	NaN	NaN	-1.2894	0	Q91VE6	MKI67 FHA domain-interacting nucleolar phosphoprotein	Nifk	mmu:67949				
3555	-0.8623	NaN	-1.7070	-1.2847	0	Q3TTY0	Phospholipase B1, membrane-associated;Phospholipase A2;Lysophospholipase	Plb1	mmu:665270				
3556	NaN	-1.2828	NaN	-1.2828	0	Q8R1T1	Charged multivesicular body protein 7	Chmp7	mmu:105513				
3557	-1.7882	NaN	-0.1099	-1.2782	0	Q9CWN7	CCR4-NOT transcription complex subunit 11	Cnot11	mmu:52846				
3558	-1.2727	NaN	NaN	-1.2727	0	Q9DA03	Complex III assembly factor LYRM7	Lyrm7	mmu:75530				
3559	NaN	-0.2104	-1.4297	-1.2710	0	Q8VDC0	Probable leucine--tRNA ligase, mitochondrial	Lars2	mmu:102436				
3560	-1.2639	NaN	NaN	-1.2639	0	Q8C1S0	Mediator of RNA polymerase II transcription subunit 19	Med19	mmu:381379				
3561	-0.7240	NaN	NaN	-1.2501	0	Q9JLJ1	Selenoprotein K	Selk	mmu:80795	1			
3562	NaN	NaN	-1.2490	-1.2490	0	Q8BMI3	ADP-ribosylation factor-binding protein GGA3	Gga3	mmu:260302				
3563	NaN	NaN	-0.7936	-1.2180	0	Q9D1C1	Ubiquitin-conjugating enzyme E2 C	Ube2c	mmu:68612			1	
3564	NaN	-1.2135	NaN	-1.2135	0	Q9CQY5	Magnesium transporter protein 1	Magt1					
3565	-1.2125	NaN	NaN	-1.2125	0	Q7TMV3	FAST kinase domain-containing protein 5	Fastkd5	mmu:380601				
3566	NaN	NaN	NaN	-1.2073	0	Q8VE92	RNA-binding protein 4B	Rbm4b	mmu:66704				
3567	NaN	-1.2062	NaN	-1.2062	0	Q8VIG0	Zinc finger CCHC domain-containing protein 14	Zcchc14	mmu:142682				
3568	-0.7373	NaN	-1.6733	-1.2053	0	Q8VEG6	CCR4-NOT transcription complex subunit 6-like	Cnot6l	mmu:231464				
3569	-1.1862	NaN	NaN	-1.1862	0	Q61009	Scavenger receptor class B member 1	Scarb1	mmu:20778				
3570	-1.1855	NaN	NaN	-1.1855	0	Q32NY4	Metal transporter CNNM3	Cnnm3	mmu:94218				
3571	NaN	0.0087	-0.5974	-1.1787	0	Q9Z2I9	Succinyl-CoA ligase [ADP-forming] subunit beta, mitochondrial	Sucla2	mmu:20916				
3572	-1.1753	NaN	NaN	-1.1753	0	P97931	Uracil-DNA glycosylase	Ung	mmu:22256				
3573	0.0382	NaN	NaN	-1.1621	0	Q8BPM0	Disheveled-associated activator of morphogenesis 1	Daam1	mmu:208846				
3574	NaN	-1.4927	-0.8250	-1.1589	0	Q3TQI7	Uncharacterized protein C9orf78 homolog		mmu:227707				
3575	NaN	1.4985	-0.5354	-1.1442	0	P55302	Alpha-2-macroglobulin receptor-associated protein	Lrpap1	mmu:16976				
3576	-1.1440	NaN	NaN	-1.1440	0	Q9D8C3	Putative bifunctional UDP-N-acetylglucosamine transferase and deubiquitinase ALG13	Alg13	mmu:67574				
3577	-0.2874	NaN	NaN	-1.1327	0	Q8VCY6	U3 small nucleolar RNA-associated protein 6 homolog	Utp6	mmu:216987				
3578	-1.4212	NaN	-0.8143	-1.1167	0	Q80V86	Integrator complex subunit 8	Ints8	mmu:72656				
3579	-2.0510	NaN	NaN	-1.1135	0	B2RQG2		Phf3	mmu:213109				
3580	NaN	NaN	NaN	-1.1124	0	Q8C0Q2	Zinc fingers and homeoboxes protein 3	Zhx3	mmu:320799				
3581	NaN	-1.0292	-1.1934	-1.1113	0	Q9Z0P4	Paralemmin-1	Palm	mmu:18483				
3582	NaN	-1.0924	NaN	-1.0924	0	Q8R3U1	HRAS-like suppressor 3	Pla2g16	mmu:225845				
3583	NaN	NaN	NaN	-1.0909	0	Q9D915	Uncharacterized protein C8orf4 homolog		mmu:69068				
3584	NaN	-1.0888	NaN	-1.0888	0	Q6PB97	F-box/LRR-repeat protein 19	Fbxl19	mmu:233902				
3585	-1.0792	NaN	NaN	-1.0792	0	O35988	Syndecan-4	Sdc4	mmu:20971				
3586	-1.0781	NaN	NaN	-1.0781	0	Q8BGE4	Sushi domain-containing protein 6	Susd6	mmu:217684				
3587	NaN	NaN	NaN	-1.0753	0	Q8C0J6	Ankyrin repeat domain-containing protein SOWAHC	Sowahc	mmu:268301				
3588	NaN	-1.5328	-0.6107	-1.0718	0	Q80SZ7	Guanine nucleotide-binding protein G(I)/G(S)/G(O) subunit gamma-5	Gng5	mmu:14707				
3589	-1.0715	NaN	NaN	-1.0715	0	Q9D0J8	Parathymosin	Ptms	mmu:69202				
3590	-1.0609	NaN	NaN	-1.0609	0	Q7TMY4	THO complex subunit 7 homolog	Thoc7	mmu:66231				
3591	NaN	-0.0496	-2.0580	-1.0538	0	O35075	Down syndrome critical region protein 3 homolog	Dscr3	mmu:13185				
3592	NaN	NaN	-0.3752	-1.0382	0	Q9D8Y1	Transmembrane protein 126A	Tmem126a	mmu:66271				
3593	NaN	-1.0365	NaN	-1.0365	0	Q8VCP8	Adenylate kinase isoenzyme 6	Ak6	mmu:102216272				
3594	-0.5896	NaN	NaN	-1.0361	0	G3X9Z4		Pcf11	mmu:74737				
3595	-1.0298	NaN	NaN	-1.0298	0	Q62311	Transcription initiation factor TFIID subunit 6	Taf6	mmu:21343				
3596	NaN	-0.4405	-0.9089	-1.0209	0	Q99J99	Sulfurtransferase;3-mercaptopyruvate sulfurtransferase	Mpst					
3597	NaN	0.1213	-0.1947	-1.0133	0	Q80Y14	Glutaredoxin-related protein 5, mitochondrial	Glrx5	mmu:73046				
3598	-1.0116	NaN	NaN	-1.0116	0	Q3TPX4	Exocyst complex component 5	Exoc5	mmu:105504				
3599	NaN	NaN	NaN	-1.0048	0	Q80VI1	E3 ubiquitin-protein ligase TRIM56	Trim56	mmu:384309				
3600	NaN	-0.6165	-1.3914	-1.0040	0	Q8CIV8	Tubulin-specific chaperone E	Tbce	mmu:70430				
3601	-1.0039	NaN	NaN	-1.0039	0	Q9R099	Transducin beta-like protein 2	Tbl2	mmu:27368				
3602	-0.6020	NaN	NaN	-0.9972	0	Q5U4D9	THO complex subunit 6 homolog	Thoc6	mmu:386612				
3603	-0.9937	NaN	NaN	-0.9937	0	P30051	Transcriptional enhancer factor TEF-1;Transcriptional enhancer factor TEF-3;Transcriptional enhar	Tead1;Tea	mmu:21676				
3604	NaN	NaN	NaN	-0.9895	0	G5E837		Hcfc2	mmu:67933				
3605	NaN	NaN	-0.9799	-0.9799	0	Q80VP1	Epsin-1	Epn1	mmu:13854				
3606	NaN	NaN	-0.9779	-0.9779	0	Q61578	NADPH:adrenodoxin oxidoreductase, mitochondrial	Fdxr	mmu:14149				
3607	0.0124	-1.9577	NaN	-0.9727	0	P10630	Eukaryotic initiation factor 4A-II;Eukaryotic initiation factor 4A-II, N-terminally processed	Eif4a2	mmu:13682				
3608	NaN	-0.9676	NaN	-0.9676	0	Q9CWV0	Mitochondrial assembly of ribosomal large subunit protein 1	Malsu1	mmu:75593				
3609	NaN	-0.3335	-1.5765	-0.9550	0	Q8BGX0	E3 ubiquitin-protein ligase TRIM23	Trim23	mmu:81003			1	
3610	NaN	NaN	-0.9494	-0.9494	0	Q8C561	LMBR1 domain-containing protein 2	Lmbrd2	mmu:320506				
3611	-0.9485	NaN	NaN	-0.9485	0	Q8BU11	TOX high mobility group box family member 4	Tox4	mmu:268741				
3612	NaN	0.1492	-0.2072	-0.9454	0	Q99MZ7	Peroxisomal trans-2-enoyl-CoA reductase	Pecr	mmu:111175				
3613	-0.9369	NaN	NaN	-0.9369	0	S4R2K0		Pdf	mmu:68023				
3614	NaN	0.3468	-2.2128	-0.9330	0	P08122	Collagen alpha-2(IV) chain;Canstatin	Col4a2	mmu:12827				
3615	NaN	-0.2061	-1.6425	-0.9243	0	Q3TFD2	Lysophosphatidylcholine acyltransferase 1	Lpcat1	mmu:210992				
3616	-0.7123	NaN	NaN	-0.9240	0	P11276	Fibronectin;Anastellin	Fn1	mmu:14268				
3617	NaN	NaN	-0.9133	-0.9133	0	Q8BR70	Protein YIPF;Protein YIPF6	Yipf6	mmu:77929				
3618	-0.1660	NaN	NaN	-0.9081	0	Q60862	Origin recognition complex subunit 2	Orc2	mmu:18393				
3619	-0.8999	NaN	NaN	-0.8999	0	Q62141	Paired amphipathic helix protein Sin3b	Sin3b	mmu:20467				
3620	-1.0347	NaN	-0.0215	-0.8850	0	P05202	Aspartate aminotransferase, mitochondrial	Got2	mmu:14719				
3621	-0.8779	NaN	NaN	-0.8779	0	P63044	Vesicle-associated membrane protein 2	Vamp2	mmu:22318				
3622	-1.4594	NaN	NaN	-0.8749	0	Q3U1M7		Zmynd8	mmu:228880				
3623	NaN	1.2651	-2.0959	-0.8743	0	Q9Z0R6	Intersectin-2	Itsn2					
3624	NaN	NaN	0.2920	-0.8713	0	Q91WN1	DnaJ homolog subfamily C member 9	Dnajc9	mmu:108671				
3625	-0.8689	NaN	NaN	-0.8689	0	Q0HA38	Tetratricopeptide repeat protein 21B	Ttc21b	mmu:73668				
3626	NaN	NaN	NaN	-0.8676	0	Q8VCG3	WD repeat-containing protein 74	Wdr74	mmu:107071				
3627	0.4549	NaN	-0.1088	-0.8638	0	E9QAH1		Golgb1					
3628	NaN	-1.3585	0.1309	-0.8559	0	Q99M04	Lipoyl synthase, mitochondrial	Lias	mmu:79464	1			
3629	-0.8552	NaN	NaN	-0.8552	0	Q9CYA6	Zinc finger CCHC domain-containing protein 8	Zcchc8	mmu:70650				
3630	NaN	-1.3244	NaN	-0.8513	0	Q8BGR9	Ubiquitin-like domain-containing CTD phosphatase 1	Ublcp1	mmu:79560		1		
3631	NaN	NaN	NaN	-0.8510	0	Q8R3V2		Aimp2					
3632	NaN	-0.6205	-0.6692	-0.8503	0	Q8VCW8	Acyl-CoA synthetase family member 2, mitochondrial	Acsf2	mmu:264895				
3633	NaN	-1.1544	-1.3802	-0.8445	0	O35874	Neutral amino acid transporter A;Amino acid transporter	Slc1a4	mmu:55963				
3634	0.2095	-1.0844	NaN	-0.8421	0	P25425	POU domain protein;POU domain, class 2, transcription factor 1	Pou2f1	mmu:18986				
3635	-0.8417	NaN	NaN	-0.8417	0	Q9CXK8	60S ribosome subunit biogenesis protein NIP7 homolog	Nip7	mmu:66164				
3636	NaN	NaN	-0.8372	-0.8372	0	Q9D074	E3 ubiquitin-protein ligase MGRN1	Mgrn1	mmu:17237			1	
3637	NaN	-0.7029	-0.9517	-0.8273	0	Q99P58	Ras-related protein Rab-27B	Rab27b	mmu:80718				
3638	-0.2847	NaN	0.0493	-0.8270	0	Q8BX10	Serine/threonine-protein phosphatase PGAM5, mitochondrial	Pgam5	mmu:72542		1		
3639	-0.8260	NaN	NaN	-0.8260	0	Q9QYA2	Mitochondrial import receptor subunit TOM40 homolog	Tomm40	mmu:53333				
3640	-0.3355	NaN	NaN	-0.8184	0	Q922E6	FAST kinase domain-containing protein 2	Fastkd2	mmu:75619				
3641	NaN	NaN	-0.8081	-0.8081	0	Q9D8M3	Heme transporter HRG1	Slc48a1	mmu:67739				
3642	-0.1696	NaN	-0.3848	-0.8073	0	Q8BRH0	Transmembrane and TPR repeat-containing protein 3	Tmtc3	mmu:237500				
3643	NaN	NaN	-0.8061	-0.8061	0	Q9JKF6	Nectin-1	Pvrl1	mmu:58235				

3644	-1.9651	NaN	NaN	-0.8007	0	Q924H7	WW domain-containing adapter protein with coiled-coil	Wac	mmu:225131				
3645	NaN	-1.4520	-0.1446	-0.7983	0	D3Z6J0		N6amt1					
3646	-0.9722	NaN	NaN	-0.7950	0	Q810V0	U3 small nucleolar ribonucleoprotein protein MPP10	Mphosph1	mmu:67973				
3647	-0.7949	NaN	NaN	-0.7949	0	L7N236		Gm15127;Gm15114;Gm10439;Ott;Luzp4;Gm15097;Gm15107;Gm15091;Gm15093;Gm15080;Gm15128					
3648	-1.3306	NaN	-0.1620	-0.7934	0	P56960	Exosome component 10	Exosc10	mmu:50912				
3649	NaN	-0.5895	-0.9962	-0.7929	0	O35242	Protein FAN	Nsmaf	mmu:18201				
3650	NaN	NaN	-0.7890	-0.7890	0	Q6NSQ9	Glucose-6-phosphatase 3	G6pc3	mmu:68401				
3651	NaN	-0.9913	-0.0587	-0.7881	0	Q9JIY5	Serine protease HTRA2, mitochondrial	Htra2	mmu:64704	1			
3652	-0.7858	NaN	NaN	-0.7858	0	Q8BL74	General transcription factor 3C polypeptide 2	Gm29609;(	mmu:71752				
3653	-0.7811	NaN	NaN	-0.7811	0	Q9JLC6	Thyrotroph embryonic factor	Tef	mmu:21685				
3654	NaN	-2.0531	0.4988	-0.7771	0	O54754	Aldehyde oxidase 1	Aox1	mmu:11761	1			
3655	-0.7724	NaN	NaN	-0.7724	0	P84091	AP-2 complex subunit mu	Ap2m1	mmu:11773				
3656	-0.7647	NaN	NaN	-0.7647	0	G5E8P0		Tubgcp6	mmu:328580				
3657	-0.7609	NaN	NaN	-0.7609	0	Q9Z2E1	Methyl-CpG-binding domain protein 2	Mbd2	mmu:17191				
3658	NaN	-2.1497	0.6305	-0.7596	0	Q9CX60	Protein LBH	Lbh	mmu:77889				
3659	NaN	-2.4556	-0.5788	-0.7576	0	Q91VU0	Protein FAM3C	Fam3c	mmu:27999				
3660	-0.7559	NaN	NaN	-0.7559	0	Q6ZPR6	Inhibitor of Bruton tyrosine kinase	Ibtk	mmu:108837				
3661	-0.7539	NaN	NaN	-0.7539	0	Q8R2N2	Cirhin	Cirh1a	mmu:21771				
3662	NaN	-0.7532	NaN	-0.7532	0	Q3UDK1	TRAF-type zinc finger domain-containing protein 1	Trafd1	mmu:231712				
3663	NaN	-0.7518	NaN	-0.7518	0	P83877	Thioredoxin-like protein 4A	Txnl4a	mmu:27366				
3664	NaN	-0.7468	NaN	-0.7468	0	Q99J78	Differentially expressed in FDCP 8	Def8	mmu:23854				
3665	NaN	1.4244	1.0911	-0.7460	0	O54879	High mobility group protein B3	Hmgb3	mmu:15354				
3666	NaN	-0.2367	[illegible]	-0.7455	0	Q6NWW5	Kinesin-like protein KIF24	Kif24	mmu:109242				
3667	NaN	-0.3644	-1.1231	-0.7438	0	Q6ZWQ7		Spcs3	mmu:76687				
3668	-0.2666	NaN	-1.2112	-0.7407	0	Q9WU40	Inner nuclear membrane protein Man1	Lemd3	mmu:380664				
3669	-0.2585	NaN	-0.3330	-0.7315	0	P49135	TFIIH basal transcription factor complex helicase XPB subunit	Ercc3	mmu:13872	1			1
3670	NaN	NaN	-0.4141	-0.7200	0	Q8CJ96	Ras association domain-containing protein 8	Rassf8	mmu:71323				
3671	NaN	NaN	-0.7169	-0.7169	0	Q9D2N9	Vacuolar protein sorting-associated protein 33A	Vps33a	mmu:77573				
3672	NaN	NaN	NaN	-0.7162	0	Q91WT8	RNA-binding protein 47	Rbm47	mmu:245945				
3673	-0.7148	NaN	NaN	-0.7148	0	Q80UU1	Ankyrin repeat and zinc finger domain-containing protein 1	Ankzf1	mmu:52231				
3674	0.6721	NaN	NaN	-0.7145	0	O70157	DNA topoisomerase;DNA topoisomerase 3-alpha	Top3a	mmu:21975				
3675	-1.2494	0.5583	NaN	-0.7051	0	Q9D211	CDKN2AIP N-terminal-like protein	Cdkn2aipn	mmu:52626				
3676	NaN	-0.3189	-1.0884	-0.7037	0	Q8BXL7	ADP-ribosylation factor-related protein 1	Arfrp1	mmu:76688				
3677	NaN	-0.7131	-0.6913	-0.7022	0	Q8BXQ2	GPI transamidase component PIG-T	Pigt	mmu:78928				
3678	-0.3113	NaN	NaN	-0.6976	0	Q8JZY4	Mitochondrial ribonuclease P protein 3	1110008L1	mmu:66132				
3679	0.2845	-1.6633	NaN	-0.6894	0	Q9CQT5	Proteasome maturation protein	Pomp	mmu:66537				
3680	-0.6883	NaN	NaN	-0.6883	0	Q9JJZ4	Ubiquitin-conjugating enzyme E2 J1	Ube2j1	mmu:56228			1	
3681	NaN	-0.6867	NaN	-0.6867	0	P49446	Receptor-type tyrosine-protein phosphatase epsilon	Ptpre	mmu:19267		1		
3682	NaN	-1.2408	-0.1289	-0.6849	0	P97371	Proteasome activator complex subunit 1	Psme1	mmu:19186				
3683	NaN	NaN	NaN	-0.6847	0	Q9Z2Y8	Proline synthase co-transcribed bacterial homolog protein	Prosc	mmu:114863				
3684	-0.8263	NaN	NaN	-0.6823	0	E9Q0D4		Sbf2					
3685	NaN	-0.6820	NaN	-0.6820	0	Q9Z0R4	Intersectin-1	Itsn1	mmu:16443				
3686	NaN	-0.7853	-0.5737	-0.6795	0	Q8R179	Kelch repeat and BTB domain-containing protein 4	Kbtbd4	mmu:67136				
3687	-0.8347	NaN	NaN	-0.6765	0	Q5UAK0	Mesoderm induction early response protein 1	Mier1	mmu:71148				
3688	NaN	NaN	-0.6709	-0.6709	0	Q9QZW0	Phospholipid-transporting ATPase;Phospholipid-transporting ATPase 11C	Atp11c	mmu:320940				
3689	0.2842	NaN	-1.4836	-0.6686	0	P30276	G2/mitotic-specific cyclin-B2	Ccnb2	mmu:12442				
3690	NaN	0.2961	-1.6221	-0.6630	0	Q8BUV8	Protein GPR107	Gpr107	mmu:277463				
3691	-0.6569	NaN	NaN	-0.6569	0	Q9JJN0	DNA polymerase eta	Polh	mmu:80905				
3692	NaN	NaN	0.0920	-0.6564	0	Q8C0Z1	Protein ITFG3	Itfg3	mmu:106581				
3693	-0.6489	NaN	NaN	-0.6489	0	Q99M87	DnaJ homolog subfamily A member 3, mitochondrial	Dnaja3	mmu:83945				
3694	NaN	NaN	-0.4414	-0.6407	0	Q4VC33	Macrophage erythroblast attacher	Maea	mmu:59003				
3695	NaN	-0.1165	-0.0503	-0.6403	0	Q9CQR4	Acyl-coenzyme A thioesterase 13;Acyl-coenzyme A thioesterase 13, N-terminally processed	Acot13	mmu:66834				
3696	0.1003	-3.4016	NaN	-0.6396	0	Q8BHL7	CDC42 small effector protein 1	Cdc42se1	mmu:57912				
3697	-0.1952	NaN	-1.0766	-0.6359	0	Q8C398	Phosphatidylinositol-glycan biosynthesis class W protein	Pigw	mmu:70325				
3698	NaN	NaN	-0.6310	-0.6310	0	Q9D5T0	ATPase family AAA domain-containing protein 1	Atad1	mmu:67979				
3699	-0.6306	NaN	NaN	-0.6306	0	Q6PD03	Serine/threonine-protein phosphatase 2A 56 kDa regulatory subunit alpha isoform	Ppp2r5a	mmu:226849				
3700	-0.0667	NaN	NaN	-0.6287	0	Q91W59	RNA-binding motif, single-stranded-interacting protein 1	Rbms1	mmu:56878				
3701	NaN	0.1634	NaN	-0.6252	0	Q8VCN9	Tubulin-specific chaperone C	Tbcc	mmu:72726				
3702	NaN	0.4489	1.9695	-0.6222	0	E9QP09		Cul9					
3703	NaN	-0.0962	-0.2467	-0.6218	0	Q8BM72	Heat shock 70 kDa protein 13	Hspa13	mmu:110920				
3704	-0.6214	NaN	NaN	-0.6214	0	Q9Z0S9	Prenylated Rab acceptor protein 1	Rabac1	mmu:14470				
3705	-0.2955	NaN	NaN	-0.6212	0	Q9Z1G3	V-type proton ATPase subunit C 1	Atp6v1c1	mmu:66335				
3706	0.4434	NaN	NaN	-0.6189	0	Q9R0L6	Pericentriolar material 1 protein	Pcm1	mmu:18536				
3707	NaN	-0.2535	-0.9835	-0.6185	0	Q9DBN9	Probable ATP-dependent RNA helicase DDX59	Ddx59	mmu:67997				
3708	NaN	-0.1732	-1.0631	-0.6182	0	A2AR07		Ssb					
3709	NaN	-0.5204	-0.7140	-0.6172	0	Q8BMJ7	Cell growth regulator with RING finger domain protein 1	Cgrrf1	mmu:68755				
3710	0.1103	NaN	NaN	-0.6136	0	P60762	Mortality factor 4-like protein 1	Morf4l1	mmu:21761				
3711	-0.6132	NaN	NaN	-0.6132	0	Q8CHP6	Polyhomeotic-like protein 3	Phc3	mmu:241915				
3712	-0.6499	NaN	NaN	-0.6129	0	Q9DCZ4	Apolipoprotein O	Apoo	mmu:68316				
3713	NaN	-0.3198	-1.1719	-0.6113	0	A2RTL5	Arginine/serine-rich coiled-coil protein 2	Rsrc2	mmu:208606				
3714	-0.6748	0.3409	NaN	-0.6093	0	Q6P4S8	Integrator complex subunit 1	Ints1	mmu:68510				
3715	-0.2317	-0.9844	NaN	-0.6080	0	P10417	Apoptosis regulator Bcl-2	Bcl2	mmu:12043	1			1
3716	1.0989	NaN	1.0594	-0.6075	0	E9Q6R4		Arid1b					
3717	0.2254	NaN	-0.0837	-0.6070	0	P0DOV2		Ifi204					
3718	-0.4210	-0.7861	NaN	-0.6036	0	P21107	Tropomyosin alpha-3 chain	Tpm3	mmu:59069				
3719	4.1298	-5.3346	NaN	-0.6024	0	Q8R1S0	Ubiquinone biosynthesis monooxygenase COQ6, mitochondrial	Coq6	mmu:217707				
3720	0.1558	NaN	NaN	-0.5966	0	Q9D7J9	Enoyl-CoA hydratase domain-containing protein 3, mitochondrial	Echdc3	mmu:67856				
3721	-0.5937	NaN	NaN	-0.5937	0	P56581	G/T mismatch-specific thymine DNA glycosylase	Tdg	mmu:21665				
3722	-0.5872	NaN	NaN	-0.5872	0	Q80X32	UPF0461 protein C5orf24 homolog		mmu:78521				
3723	-2.9590	NaN	NaN	-0.5839	0	Q3UIU2	NADH dehydrogenase [ubiquinone] 1 beta subcomplex subunit 6	Ndufb6	mmu:230075				
3724	-0.5760	NaN	NaN	-0.5760	0	Q3U699	Protein C21orf2 homolog	1810043G(	mmu:67884				
3725	-0.5755	NaN	NaN	-0.5755	0	P56371	Ras-related protein Rab-4A	Rab4a	mmu:19341				
3726	NaN	NaN	-0.6540	-0.5726	0	Q8C735	Protein lin-9 homolog	Lin9					
3727	-0.5720	NaN	NaN	-0.5720	0	Q8BJS8	Mdm2-binding protein	Mtbp	mmu:105837				
3728	-1.2334	0.1684	NaN	-0.5708	0	Q8BZ36	RAD50-interacting protein 1	Rint1	mmu:72772				
3729	-0.2210	NaN	NaN	-0.5706	0	P97329	Kinesin-like protein KIF20A	Kif20a	mmu:19348				
3730	-0.5680	NaN	NaN	-0.5680	0	Q91YT0	NADH dehydrogenase [ubiquinone] flavoprotein 1, mitochondrial	Ndufv1	mmu:17995				
3731	0.4004	NaN	-0.5592	-0.5654	0	Q9Z207	Protein diaphanous homolog 3	Diap3;Diap	mmu:56419				
3732	NaN	-0.2452	-0.9470	-0.5652	0	P39429	TNF receptor-associated factor 2	Traf2	mmu:22030	1		1	1
3733	0.7161	NaN	-1.8774	-0.5647	0	Q9Z148	Histone-lysine N-methyltransferase EHMT2	Ehmt2	mmu:110147				
3734	0.1682	-0.6161	NaN	-0.5634	0	Q8C1B1	Calmodulin-regulated spectrin-associated protein 2	Camsap2					
3735	-0.5615	NaN	NaN	-0.5615	0	A0A1B0GS85		Zbtb45					
3736	NaN	-0.5042	-0.6146	-0.5594	0	Q9CV28	Protein FAM188A	Fam188a	mmu:66960				
3737	NaN	-0.2076	-0.7294	-0.5578	0	Q8BGT7	Survival of motor neuron-related-splicing factor 30	Smndc1	mmu:76479				1
3738	0.0299	NaN	NaN	-0.5572	0	O88559	Menin	Men1	mmu:17283				
3739	NaN	NaN	-0.4736	-0.5568	0	P42128	Forkhead box protein K1	Foxk1	mmu:17425				
3740	NaN	NaN	NaN	-0.5535	0	Q9JI44	DNA methyltransferase 1-associated protein 1	Dmap1	mmu:66233				
3741	0.6271	NaN	-1.0099	-0.5509	0	Q9CR62	Mitochondrial 2-oxoglutarate/malate carrier protein	Slc25a11	mmu:67863				
3742	-0.5504	NaN	NaN	-0.5504	0	Q8BGQ6	EF-hand calcium-binding domain-containing protein 14	Efcab14					
3743	-0.5499	NaN	NaN	-0.5499	0	A2A791	Zinc finger MYM-type protein 4	Zmym4	mmu:67785				
3744	-0.5476	NaN	NaN	-0.5476	0	Q9CYC5	Kinetochore-associated protein DSN1 homolog	Dsn1	mmu:66934				
3745	NaN	0.3092	-1.4020	-0.5464	0	Q61329	Zinc finger homeobox protein 3	Zfhx3					
3746	-0.5409	NaN	NaN	-0.5409	0	Q9D486	C-Maf-inducing protein	Cmip	mmu:74440				
3747	NaN	-1.2609	-0.1894	-0.5385	0	Q7TQK5	Coiled-coil domain-containing protein 93	Ccdc93	mmu:70829				
3748	-0.5349	NaN	NaN	-0.5349	0	Q8BTI7	Serine/threonine-protein phosphatase 6 regulatory ankyrin repeat subunit C	Ankrd52	mmu:237615				
3749	NaN	-0.5337	NaN	-0.5337	0	Q9CQE1	Protein NipSnap homolog 3B	Nipsnap3b	mmu:66536				
3750	0.1510	NaN	NaN	-0.5331	0	Q8BIG4	F-box only protein 28	Fbxo28	mmu:67948				
3751	NaN	-1.9071	0.8416	-0.5328	0	Q9CPZ6	ORM1-like protein 3	Ormdl3	mmu:66612				
3752	NaN	-0.2022	-0.8562	-0.5292	0	Q9Z0P7	Suppressor of fused homolog	Sufu	mmu:24069				
3753	-1.5056	NaN	-2.0549	-0.5284	0	P53798	Squalene synthase	Fdft1	mmu:14137				
3754	-1.5322	-0.1386	NaN	-0.5272	0	Q9CRA9	FGFR1 oncogene partner 2 homolog	Fgfr1op2	mmu:67529				
3755	NaN	NaN	-0.5256	-0.5256	0	Q9JMB0	G kinase-anchoring protein 1	Gkap1	mmu:56278				
3756	-0.5225	NaN	NaN	-0.5225	0	Q8R395	COMM domain-containing protein 5	Commd5	mmu:66398				
3757	NaN	-1.6336	0.5907	-0.5215	0	Q8BFW4	Tripartite motif-containing protein 65	Trim65	mmu:338364				
3758	-0.5192	NaN	NaN	-0.5192	0	A2ANX3	Phospholipid-transporting ATPase	Atp8b4	mmu:241633				
3759	-0.5181	NaN	NaN	-0.5181	0	Q99KN2	Probable cytosolic iron-sulfur protein assembly protein CIAO1	Ciao1	mmu:26371				
3760	0.3326	-1.4178	NaN	-0.5176	0	Q9CUB6	OTU domain-containing protein 1	Otud1	mmu:71198				
3761	NaN	-0.8423	-0.1919	-0.5171	0	A2AQ25	Sickle tail protein	Etl4;Skt	mmu:208618				
3762	-0.3891	NaN	-0.6442	-0.5166	0	Q80TE4	Signal-induced proliferation-associated 1-like protein 2	Sipa1l2	mmu:244668				
3763	-0.5158	NaN	NaN	-0.5158	0	Q64321	Zinc finger and BTB domain-containing protein 7B	Zbtb7b	mmu:22724				
3764	-0.5131	NaN	NaN	-0.5131	0	P28704	Retinoic acid receptor RXR-beta	Rxrb	mmu:20182				
3765	NaN	-0.3096	-0.7113	-0.5104	0	Q9D710	Thioredoxin-related transmembrane protein 2	Tmx2	mmu:66958				
3766	0.6737	-1.6848	NaN	-0.5055	0	Q7TN22	Thioredoxin domain-containing protein 16	Txndc16	mmu:70561				
3767	NaN	NaN	-0.9915	-0.5044	0	Q9CYY7	Protein slowmo homolog 2	Slmo2	mmu:66390				
3768	NaN	0.5145	-1.5174	-0.5014	0	Q8R3W5	tRNA-splicing endonuclease subunit Sen15	Tsen15	mmu:66637				
3769	NaN	NaN	-0.5004	-0.5004	0	P48758	Carbonyl reductase [NADPH] 1	Cbr1	mmu:12408				

3770	-0.3714	NaN	NaN	-0.4995	0	Q9CSP9	Tetratricopeptide repeat protein 14	Ttc14	mmu:67120			
3771	NaN	0.0587	-1.0491	-0.4952	0	Q8BH79	Anoctamin-10	Ano10	mmu:102566			
3772	NaN	-0.7270	-0.2549	-0.4910	0	Q8VD04	GRIP1-associated protein 1	Gripap1	mmu:54645			
3773	-0.6360	NaN	NaN	-0.4909	0	Q8BUI3	Leucine-rich repeat and WD repeat-containing protein 1	LRWD1	mmu:71735			
3774	NaN	-0.1946	-0.7868	-0.4907	0	Q61160	FAS-associated death domain protein	Fadd	mmu:14082			1
3775	NaN	-0.4517	-0.5296	-0.4907	0	Q99J47	Dehydrogenase/reductase SDR family member 7B	Dhrs7b	mmu:216820			
3776	-0.4884	NaN	NaN	-0.4884	0	Q9D6M3	Mitochondrial glutamate carrier 1	Slc25a22	mmu:68267			
3777	-1.0522	NaN	NaN	-0.4865	0	Q920A7	AFG3-like protein 1	Afg3l1	mmu:114896			
3778	-0.4862	NaN	NaN	-0.4862	0	Q9R0C0	Biogenesis of lysosome-related organelles complex 1 subunit 6	Bloc1s6	mmu:18457			
3779	-2.0651	NaN	NaN	-0.4860	0	O08696	Forkhead box protein M1	Foxm1		1		
3780	-0.4851	NaN	NaN	-0.4851	0	Q8BJ56	Patatin-like phospholipase domain-containing protein 2	Pnpla2	mmu:66853			
3781	NaN	-1.0416	0.0745	-0.4836	0	Q8BZN6	Dedicator of cytokinesis protein 10	Dock10	mmu:210293			
3782	NaN	NaN	NaN	-0.4831	0	O35216	Histone H3-like centromeric protein A	Cenpa	mmu:12615			
3783	NaN	NaN	-0.4827	-0.4827	0	P97315	Cysteine and glycine-rich protein 1	Csrp1	mmu:13007			
3784	0.2882	-1.2527	NaN	-0.4823	0	Q9DC70	NADH dehydrogenase [ubiquinone] iron-sulfur protein 7, mitochondrial	Ndufs7	mmu:75406			
3785	0.8855	NaN	NaN	-0.4822	0	Q91WM1	Spermatid perinuclear RNA-binding protein	Strbp	mmu:20744			
3786	0.2036	NaN	NaN	-0.4816	0	P97352	Protein S100-A13	S100a13				
3787	0.2481	-1.2064	NaN	-0.4791	0	Q80UK7	Spindle assembly abnormal protein 6 homolog	Sass6	mmu:72776			
3788	-0.4737	NaN	NaN	-0.4737	0	O35963	Ras-related protein Rab-33B	Rab33b	mmu:19338			
3789	NaN	-0.7711	-0.1739	-0.4725	0	Q78JW9	Ubiquitin domain-containing protein UBFD1	Ubfd1	mmu:28018			
3790	NaN	0.5590	-0.0097	-0.4706	0	Q99K30	Epidermal growth factor receptor kinase substrate 8-like protein 2	Eps8l2	mmu:98845			
3791	1.1010	-4.0418	NaN	-0.4697	0	Q9CQA5	Mediator of RNA polymerase II transcription subunit 4	Med4	mmu:67381			
3792	NaN	0.4944	0.2494	-0.4695	0	O55126	Protein NipSnap homolog 2	Gbas				
3793	0.1959	-0.3703	NaN	-0.4653	0	Q9EQH2	Endoplasmic reticulum aminopeptidase 1	Erap1	mmu:80898			
3794	NaN	-1.0953	0.1654	-0.4649	0	Q7TMW6	Cytosolic Fe-S cluster assembly factor NARFL	Narfl	mmu:67563			
3795	NaN	0.1522	-1.0798	-0.4638	0	Q9Z2Q6	Septin-5	Sept5	mmu:18951			
3796	-2.5253	NaN	NaN	-0.4632	0	Q9ESW4	Acylglycerol kinase, mitochondrial	Agk	mmu:69923			
3797	-0.9256	-0.2306	NaN	-0.4630	0	Q8CFQ3	Intron-binding protein aquarius	Aqr	mmu:11834			
3798	-0.4301	NaN	-0.4930	-0.4615	0	Q3UFB2	Box C/D snoRNA protein 1	Znhit6	mmu:229937			
3799	NaN	-0.2329	-0.6867	-0.4598	0	Q91W69	Epsin-3	Epn3;Epn1	mmu:71889			
3800	-0.0891	NaN	NaN	-0.4595	0	E9PWW9		Rsf1	mmu:233532			
3801	-0.2153	NaN	-0.5147	-0.4590	0	Q924A2	Protein capicua homolog	Cic	mmu:71722			
3802	-0.7220	-0.1953	NaN	-0.4587	0	Q9D666	SUN domain-containing protein 1	Sun1	mmu:77053			
3803	-0.4581	NaN	NaN	-0.4581	0	Q3TV65	MPN domain-containing protein	Mpnd	mmu:68047			
3804	NaN	-0.0042	-0.9100	-0.4571	0	P62717	60S ribosomal protein L18a	Rpl18a;Gm	mmu:76808			
3805	NaN	-0.2257	-0.6872	-0.4565	0	Q9CQ45	Neudesin	Nenf	mmu:66208			
3806	-0.4241	NaN	0.1858	-0.4558	0	Q9JII5	DAZ-associated protein 1	Dazap1	mmu:70248			
3807	0.2085	NaN	-0.4217	-0.4554	0	Q8BKT3	GC-rich sequence DNA-binding factor 2	Gcfc2	mmu:330361			
3808	NaN	-0.3715	-0.5379	-0.4547	0	Q9QZA0	Carbonic anhydrase 5B, mitochondrial	Ca5b	mmu:56078			
3809	[illegible]	NaN	NaN	-0.4544	0	Q8K2A7	Integrator complex subunit 10	Ints10	mmu:70885			
3810	NaN	NaN	-0.4536	-0.4536	0	Q8BFQ4	WD repeat-containing protein 82	Wdr82	mmu:77305			
3811	NaN	-0.4528	NaN	-0.4528	0	Q3USB7	Inactive phospholipase C-like protein 1	Plcl1	mmu:227120			
3812	NaN	-0.4518	NaN	-0.4518	0	Q8R2W9	Pantothenate kinase 3	Pank3	mmu:211347			
3813	-1.5874	NaN	NaN	-0.4489	0	Q9DBN5	Lon protease homolog 2, peroxisomal	Lonp2	mmu:66887			
3814	-0.4488	NaN	-0.4461	-0.4475	0	Q8K0D7	Tail-anchored protein insertion receptor WRB	Wrb	mmu:71446			
3815	NaN	-0.4467	NaN	-0.4467	0	Q8CIG0	DEP domain-containing protein 1A	Depdc1a	mmu:76131			
3816	-1.0321	NaN	NaN	-0.4464	0	Q64318	Zinc finger E-box-binding homeobox 1	Zeb1	mmu:21417			
3817	-0.4445	NaN	NaN	-0.4445	0	A6X8Z5	Rho GTPase-activating protein 31	Arhgap31	mmu:12549			
3818	-0.4433	NaN	NaN	-0.4433	0	Q7TT00	Transcription factor SPT20 homolog	Supt20h	mmu:56790			
3819	-0.4795	NaN	NaN	-0.4420	0	Q80YS6	Actin filament-associated protein 1	Afap1	mmu:70292			
3820	-0.1533	NaN	-0.3041	-0.4410	0	Q9CXJ1	Probable glutamate--tRNA ligase, mitochondrial	Ears2	mmu:67417			
3821	-0.6209	-0.2611	NaN	-0.4410	0	Q8VCH6	Delta(24)-sterol reductase	Dhcr24	mmu:74754	1		1
3822	-0.4388	NaN	NaN	-0.4388	0	Q62245	Son of sevenless homolog 1	Sos1	mmu:20662			
3823	0.1282	NaN	NaN	-0.4356	0	O70279	Protein DGCR14	Dgcr14	mmu:27886			
3824	-0.4346	NaN	NaN	-0.4346	0	Q9D8N1	Uncharacterized protein C11orf24 homolog		mmu:72056			
3825	-0.4332	NaN	NaN	-0.4332	0	Q8BUR3	Forkhead box protein J3	Foxj3	mmu:230700			
3826	NaN	-0.6185	0.0785	-0.4311	0	Q8BU88	39S ribosomal protein L22, mitochondrial	Mrpl22	mmu:216767			
3827	0.0887	NaN	NaN	-0.4310	0	Q9D864	Actin-related protein 6	Actr6	mmu:67019			
3828	NaN	-0.9053	0.2312	-0.4307	0	P62141	Serine/threonine-protein phosphatase PP1-beta catalytic subunit	Ppp1cb	mmu:19046		1	
3829	NaN	-0.6564	-0.2022	-0.4293	0	Q99MB7	RING finger protein 141	Rnf141	mmu:67150			
3830	-0.2845	NaN	NaN	-0.4269	0	Q8BQZ5	Cleavage and polyadenylation specificity factor subunit 4	Cpsf4	mmu:54188			
3831	-0.1277	-0.1616	NaN	-0.4253	0	Q6NZQ4	PAX-interacting protein 1	Paxip1	mmu:55982			
3832	NaN	-0.4248	NaN	-0.4248	0	Q8C3W1	Uncharacterized protein C1orf198 homolog		mmu:69551			
3833	[illegible]	NaN	NaN	-0.4183	0	Q91ZE0	Trimethyllysine dioxygenase, mitochondrial	Tmlhe	mmu:192289			
3834	-0.2040	-0.2972	NaN	-0.4182	0	Q9DAT5	Mitochondrial tRNA-specific 2-thiouridylase 1	Trmu	mmu:72026			
3835	0.0766	NaN	-0.9108	-0.4171	0	O54804	Choline kinase alpha	Chka	mmu:12660			
3836	-0.4150	NaN	NaN	-0.4150	0	P60882	Multiple epidermal growth factor-like domains protein 8	Megf8	mmu:269878			
3837	1.0089	NaN	NaN	-0.4135	0	Q6PFD6	Kinesin-like protein KIF18B	Kif18b	mmu:70218			
3838	NaN	-0.2075	-0.6185	-0.4130	0	P35279	Ras-related protein Rab-6A	Rab6a	mmu:19346			
3839	-0.4125	NaN	NaN	-0.4125	0	Q5HZI9	Solute carrier family 25 member 51	Slc25a51	mmu:230125			
3840	NaN	-0.2761	0.4045	-0.4115	0	Q9D0C4	tRNA (guanine(37)-N1)-methyltransferase	Trmt5	mmu:76357			
3841	NaN	0.2097	NaN	-0.4109	0	Q91WG3	Probable tRNA pseudouridine synthase 2	Trub2	mmu:227682			
3842	NaN	-1.5469	0.7282	-0.4094	0	Q8QZZ7	EKC/KEOPS complex subunit Tprkb	Tprkb	mmu:69786			
3843	NaN	1.1604	-0.4042	-0.4084	0	Q8K1J6	CCA tRNA nucleotidyltransferase 1, mitochondrial	Trnt1	mmu:70047			
3844	NaN	NaN	-0.4071	-0.4071	0	O35153	BET1-like protein	Bet1l	mmu:54399			
3845	NaN	-0.0967	1.0188	-0.4061	0	Q99M51	Cytoplasmic protein NCK1	Nck1	mmu:17973			
3846	NaN	-0.4049	NaN	-0.4049	0	Q91XL3	UDP-glucuronic acid decarboxylase 1	Uxs1	mmu:67883			
3847	NaN	NaN	-0.4027	-0.4027	0	Q9ESW8	Pyroglutamyl-peptidase 1	Pgpep1	mmu:66522			
3848	-0.4076	NaN	NaN	-0.4026	0	Q9CQJ8	NADH dehydrogenase [ubiquinone] 1 beta subcomplex subunit 9	Ndufb9	mmu:66218			
3849	NaN	-0.3228	-0.4817	-0.4023	0	Q3ULD5	Methylcrotonoyl-CoA carboxylase beta chain, mitochondrial	Mccc2	mmu:78038			
3850	NaN	-0.4748	-0.3293	-0.4021	0	Q99JR1	Sideroflexin-1	Sfxn1	mmu:14057			
3851	0.3171	NaN	NaN	-0.4002	0	Q3V300	Kinesin-like protein KIF22	Kif22	mmu:110033			
3852	-0.3981	NaN	NaN	-0.3981	0	Q61666	Protein HIRA	Hira	mmu:15260			
3853	NaN	-0.4435	-0.3515	-0.3975	0	P58774	Tropomyosin beta chain	Tpm2	mmu:22004			
3854	-0.3933	NaN	NaN	-0.3974	0	Q9QZQ8	Core histone macro-H2A,1	H2afy	mmu:26914			
3855	NaN	-0.3508	-0.1116	-0.3957	0	P61219	DNA-directed RNA polymerases I, II, and III subunit RPABC2	Polr2f	mmu:69833			
3856	NaN	-0.7256	-0.0645	-0.3950	0	Q6IRU5	Clathrin light chain B	Cltb	mmu:74325			
3857	-0.3945	NaN	NaN	-0.3945	0	O70133	ATP-dependent RNA helicase A	Dhx9	mmu:13211			
3858	-0.8168	NaN	0.0714	-0.3927	0	D3YWT1		Hnrnph3				
3859	NaN	0.3832	0.2402	-0.3916	0	P24369	Peptidyl-prolyl cis-trans isomerase B	Ppib	mmu:19035			
3860	-0.3892	NaN	NaN	-0.3892	0	Q9CQ91	NADH dehydrogenase [ubiquinone] 1 alpha subcomplex subunit 3	Ndufa3	mmu:66091			
3861	-0.3889	NaN	NaN	-0.3889	0	Q8R0Z5	Mitoferrin-2	Slc25a28	mmu:246696			
3862	NaN	-0.3972	-0.3768	-0.3870	0	Q811Q9	Choline-phosphate cytidylyltransferase B	Pcyt1b	mmu:236899			
3863	NaN	NaN	-0.3866	-0.3866	0	Q543M9		Trp53rk	mmu:76367			
3864	-0.6634	-0.0668	NaN	-0.3856	0	Q9EQJ0	Two pore calcium channel protein 1	Tpcn1	mmu:252972			
3865	NaN	NaN	NaN	-0.3844	0	Q7TSF4	Leucine-rich repeat-containing protein 75A	Lrrc75a	mmu:192976			
3866	NaN	NaN	NaN	-0.3830	0	Q99J27	Acetyl-coenzyme A transporter 1	Slc33a1	mmu:11416			
3867	-0.2367	NaN	0.5894	-0.3825	0	Q8CJ40	Rootletin	Crocc	mmu:230872			
3868	-0.3754	NaN	NaN	-0.3754	0	A0A087WS29		Ktn1				
3869	-0.3416	NaN	NaN	-0.3752	0	Q6ZQF0	DNA topoisomerase 2-binding protein 1	Topbp1	mmu:235559			
3870	-0.3698	NaN	NaN	-0.3698	0	Q9DCK3	Tetraspanin;Tetraspanin-4	Tspan4	mmu:64540			
3871	[illegible]	NaN	-0.6044	-0.3695	0	Q7TQK4	Exosome complex component RRP40	Exosc3	mmu:66362			
3872	0.2183	NaN	0.1681	-0.3695	0	Q3UFM5	Nucleolar MIF4G domain-containing protein 1	Nom1	mmu:433864			
3873	-0.3678	NaN	NaN	-0.3678	0	Q8BP97	Rhomboid domain-containing protein 3	Rhbdd3	mmu:279766			
3874	-0.3653	NaN	NaN	-0.3653	0	Q8BW94	Dynein heavy chain 3, axonemal	Dnah3;Dnah7a;Dnah7c;Dnah7b				
3875	-0.3651	NaN	NaN	-0.3651	0	Q01147	Cyclic AMP-responsive element-binding protein 1	Creb1	mmu:12912			
3876	NaN	-0.3917	-0.3341	-0.3629	0	E9Q5F9	Histone-lysine N-methyltransferase SETD2	Setd2	mmu:235626			
3877	NaN	-0.5116	[illegible]	-0.3627	0	Q9R0B9	Procollagen-lysine,2-oxoglutarate 5-dioxygenase 2	Plod2	mmu:26432			
3878	NaN	NaN	-0.3588	-0.3588	0	Q8C9S4	Coiled-coil domain-containing protein 186	Ccdc186	mmu:213993			
3879	0.3088	NaN	NaN	-0.3572	0	Q6PIJ4	Nuclear factor related to kappa-B-binding protein	Nfrkb	mmu:235134			
3880	NaN	-0.1470	-0.5617	-0.3544	0	Q62048	Astrocytic phosphoprotein PEA-15	Pea15	mmu:18611			1
3881	NaN	-1.2331	0.5293	-0.3519	0	Q80ZJ1	Ras-related protein Rap-2a	Rap2a	mmu:76108			
3882	-0.3519	NaN	NaN	-0.3519	0	Q99LM9	Transcriptional adapter 1	Tada1	mmu:27878			
3883	NaN	-0.3508	NaN	-0.3508	0	P63139	Nuclear transcription factor Y subunit beta	Nfyb	mmu:18045			
3884	-0.3301	NaN	-0.0779	-0.3487	0	Q922S8	Kinesin-like protein KIF2C;Kinesin-like protein	Kif2c	mmu:73804			
3885	0.0932	NaN	-0.2667	-0.3477	0	Q9JHC9	ETS-related transcription factor Elf-2	Elf2	mmu:69257			
3886	-0.3452	NaN	NaN	-0.3452	0	Q0VBL3		Rbm15	mmu:229700			
3887	NaN	-0.0987	-0.5906	-0.3447	0	Q8K4Z3	NAD(P)H-hydrate epimerase	Apoa1bp	mmu:246703			
3888	-0.3434	NaN	NaN	-0.3434	0	Q8R035	Peptidyl-tRNA hydrolase ICT1, mitochondrial	Ict1	mmu:68572			
3889	-0.6834	NaN	[illegible]	-0.3412	0	O88531	Palmitoyl-protein thioesterase 1	Ppt1	mmu:19063			
3890	0.2502	NaN	NaN	-0.3388	0	P51432	1-phosphatidylinositol 4,5-bisphosphate phosphodiesterase beta-3	Plcb3	mmu:18797			
3891	0.3007	NaN	NaN	-0.3380	0	Q8K327	Chromosome alignment-maintaining phosphoprotein 1	Champ1	mmu:101994			
3892	-0.2605	NaN	NaN	-0.3375	0	Q8R3F5	Malonyl-CoA-acyl carrier protein transacylase, mitochondrial	Mcat	mmu:223722			
3893	-0.2829	NaN	NaN	-0.3374	0	Q6DID5	PWWP domain-containing protein MUM1	Mum1	mmu:68114			
3894	-0.0429	NaN	0.2478	-0.3372	0	A2AM29	Protein AF-9	Mllt3	mmu:70122			
3895	0.1704	-1.0157	NaN	-0.3366	0	Q9CZV5	STAGA complex 65 subunit gamma	Supt7l	mmu:72195			

3896	NaN	NaN	-0.3362	-0.3362	0	Q8R0F5	RNA-binding motif protein, X-linked 2	Rbmx2	mmu:209003			
3897	0.0819	NaN	-0.7541	-0.3361	0	Q99J38	Tetratricopeptide repeat protein 30A1;Tetratricopeptide repeat protein 30B;Tetratricopeptide rep	Ttc30a1;Tt	mmu:78802			
3898	NaN	-0.1558	-0.2796	-0.3341	0	Q8CIH5	1-phosphatidylinositol 4,5-bisphosphate phosphodiesterase gamma-2	Plcg2	mmu:234779			
3899	NaN	-0.6351	-0.0246	-0.3299	0	P62821	Ras-related protein Rab-1A	Rab1A	mmu:19324			
3900	-0.2367	NaN	NaN	-0.3295	0	P09450	Transcription factor jun-B	Junb	mmu:16477			
3901	0.4010	NaN	-1.7120	-0.3286	0	Q8JZQ2	AFG3-like protein 2	Afg3l2	mmu:69597			
3902	-0.3267	NaN	NaN	-0.3267	0	A2A6T1	Cerebellar degeneration-related protein 2-like	Cdr2l	mmu:237988			
3903	NaN	-0.0725	-0.5807	-0.3266	0	O54786	DNA fragmentation factor subunit alpha	Dffa	mmu:13347			
3904	-0.3252	NaN	NaN	-0.3252	0	Q91W39	Nuclear receptor coactivator 5	Ncoa5	mmu:228869			
3905	-0.3232	NaN	NaN	-0.3232	0	Q99LJ8	Dehydrodolichyl diphosphate syntase complex subunit Nus1	Nus1	mmu:52014			
3906	NaN	-0.7160	0.5880	-0.3228	0	Q9WTS2	Alpha-(1,6)-fucosyltransferase	Fut8	mmu:53618			
3907	-0.0260	NaN	NaN	-0.3226	0	Q61466	SWI/SNF-related matrix-associated actin-dependent regulator of chromatin subfamily D member :	Smarcd1	mmu:83797			
3908	NaN	-0.3221	NaN	-0.3221	0	Q3U5F4	YrdC domain-containing protein, mitochondrial	Yrdc	mmu:230734			
3909	NaN	0.1631	-0.8062	-0.3216	0	O35900	U6 snRNA-associated Sm-like protein LSm2	Lsm2	mmu:27756			
3910	NaN	-0.0900	-0.5520	-0.3210	0	Q9D0G0	28S ribosomal protein S30, mitochondrial	Mrps30	mmu:59054			
3911	-0.3205	NaN	NaN	-0.3205	0	Q921L3	Transmembrane and coiled-coil domain-containing protein 1	Tmco1	mmu:68944			
3912	-0.7967	0.1564	NaN	-0.3202	0	F8VQ07		5830473C1	mmu:622307			
3913	0.0102	-0.1953	NaN	-0.3201	0	Q61464	Zinc finger protein 638	Znf638;Zfn	mmu:18139			
3914	NaN	NaN	-0.3194	-0.3194	0	Q91VX9	Transmembrane protein 168	Tmem168	mmu:101118			
3915	NaN	-0.6230	-0.0137	-0.3183	0	Q8VE42	Ankyrin repeat domain-containing protein 49	Ankrd49	mmu:56503			
3916	0.2192	NaN	-0.8553	-0.3181	0	Q9DB96	Neuroguidin	Ngdn	mmu:68966			
3917	-0.1041	NaN	NaN	-0.3179	0	Q8BIV3	Ran-binding protein 6	Ranbp6	mmu:240614			
3918	-0.3171	NaN	NaN	-0.3171	0	Q9D8B6	Protein FAM210B	Fam210b	mmu:67017			
3919	NaN	-0.0682	-0.5612	-0.3147	0	Q80ZK0	28S ribosomal protein S10, mitochondrial	Mrps10				
3920	-0.2180	0.6126	NaN	-0.3133	0	Q921X9	Protein disulfide-isomerase A5	Pdia5	mmu:72599			
3921	NaN	-0.8201	0.1939	-0.3131	0	Q9CYL5	Golgi-associated plant pathogenesis-related protein 1	Glipr2	mmu:384009			
3922	NaN	-0.1868	-0.4393	-0.3131	0	Q8CG79	Apoptosis-stimulating of p53 protein 2	Trp53bp2;	mmu:209456			1
3923	NaN	0.6004	-1.0837	-0.3122	0	Q9CXI0	2-methoxy-6-polyprenyl-1,4-benzoquinol methylase, mitochondrial	Coq5	mmu:52064			
3924	NaN	NaN	-0.5016	-0.3106	0	P59110	Sentrin-specific protease 1	Senp1	mmu:223870			
3925	NaN	-0.8962	0.2755	-0.3103	0	F6VFS9	Guanylate cyclase	Gucy2d				
3926	-0.3097	NaN	NaN	-0.3097	0	Q8K1N4	Spermatogenesis-associated serine-rich protein 2	Spats2	mmu:72572			
3927	-0.2072	NaN	NaN	-0.3096	0	O70546	Lysine-specific demethylase 6A	Kdm6a	mmu:22289			
3928	NaN	-0.7778	0.1612	-0.3083	0	Q9CQE6	Histone chaperone ASF1A	Asf1a	mmu:66403			
3929	NaN	-0.1514	-0.6778	-0.3071	0	Q8R307	Vacuolar protein sorting-associated protein 18 homolog	Vps18	mmu:228545			
3930	NaN	-0.3065	NaN	-0.3065	0	Q9D8X2	Coiled-coil domain-containing protein 124	Ccdc124	mmu:234388			
3931	-0.3039	NaN	NaN	-0.3039	0	Q8BKT8	HAUS augmin-like complex subunit 7	Haus7	mmu:73738			
3932	NaN	-0.5269	NaN	-0.3014	0	Q5SS00	DBF4-type zinc finger-containing protein 2 homolog	Zdbf2	mmu:73884			
3933	-0.3009	NaN	NaN	-0.3009	0	Q8C3X8	Lipase maturation factor 2	Lmf2	mmu:105847			
3934	0.3584	NaN	NaN	-0.3002	0	Q6PCN7	Helicase-like transcription factor	Hltf	mmu:20585		1	
3935	NaN	0.3094	-0.0883	-0.3000	0	Q91X78	Erlin-1	Erlin1	mmu:226144			
3936	NaN	-0.2860	-0.3129	-0.2994	0	Q9CR20	Immediate early response 3-interacting protein 1	Hdhd2;Ier3	mmu:66191			
3937	0.1981	NaN	-0.3471	-0.2993	0	Q6NZF1	Zinc finger CCCH domain-containing protein 11A	Zc3h11a	mmu:70579			
3938	NaN	NaN	NaN	-0.2985	0	Q60772	Cyclin-dependent kinase 4 inhibitor C	Cdkn2c	mmu:12580			
3939	NaN	NaN	NaN	-0.2979	0	Q9DBW3	Protein NATD1	Natd1	mmu:24083			
3940	-0.1472	NaN	0.9988	-0.2954	0	A2AGH6	Mediator of RNA polymerase II transcription subunit 12	Med12	mmu:59024			
3941	NaN	1.0845	-0.0657	-0.2933	0	Q9CQY2	RNMT-activating mini protein	Fam103a1;	mmu:67148			
3942	-0.2923	NaN	NaN	-0.2923	0	Q8K202	DNA-directed RNA polymerase I subunit RPA49	Polr1e	mmu:64424			
3943	-0.2921	NaN	NaN	-0.2921	0	Q810J8	Zinc finger FYVE domain-containing protein 1	Zfyve1	mmu:217695			
3944	-0.2913	NaN	NaN	-0.2913	0	Q60929	Myocyte-specific enhancer factor 2A	Mef2a	mmu:17258			
3945	NaN	-0.2470	-0.3350	-0.2910	0	Q6ZWM4	U6 snRNA-associated Sm-like protein LSm8	Lsm8	mmu:76522			
3946	-0.2884	NaN	NaN	-0.2884	0	Q8BHN5	RNA-binding protein 45	Rbm45	mmu:241490			
3947	-0.7156	0.1428	NaN	-0.2864	0	Q8BHS8	Syntabulin	Sybu	mmu:319613			
3948	NaN	-0.4854	-0.0852	-0.2853	0	Q9WVG6	Histone-arginine methyltransferase CARM1	Carm1	mmu:59035			
3949	-0.2847	NaN	NaN	-0.2847	0	P27790	Major centromere autoantigen B	Cenpb	mmu:12616			
3950	-0.2847	NaN	NaN	-0.2847	0	Q91V83	TELO2-interacting protein 1 homolog	Tti1	mmu:75425			
3951	NaN	NaN	0.5072	-0.2827	0	Q80WS3	rRNA/tRNA 2-O-methyltransferase fibrillarin-like protein 1	Fbll1	mmu:237730			
3952	NaN	-0.1397	-0.4251	-0.2824	0	Q640L5	Coiled-coil domain-containing protein 18	Ccdc18	mmu:73254			
3953	NaN	-0.2814	NaN	-0.2814	0	O08908	Phosphatidylinositol 3-kinase regulatory subunit beta	Pik3r2	mmu:18709			1
3954	-0.1862	NaN	NaN	-0.2813	0	Q9EQR6	Fanconi anemia group G protein homolog	Fancg	mmu:60534			
3955	NaN	NaN	-0.2803	-0.2803	0	Q8VEM1	E3 ubiquitin-protein ligase RNF130	Rnf130	mmu:59044		1	
3956	-0.2719	NaN	NaN	-0.2719	0	Q3TLR7	Denticleless protein homolog	Dtl	mmu:76843		1	
3957	NaN	-0.5080	-0.0340	-0.2710	0	Q91V24	ATP-binding cassette sub-family A member 7	Abca7	mmu:27403			
3958	0.8005	NaN	0.1098	-0.2709	0	Q8VCB2	Mediator of RNA polymerase II transcription subunit 25	Med25	mmu:75613			
3959	NaN	0.6755	0.5211	-0.2695	0	Q8BVL3	Sorting nexin-17	Snx17	mmu:266781			
3960	NaN	NaN	-0.2673	-0.2673	0	P50295	Arylamine N-acetyltransferase 2;Arylamine N-acetyltransferase 3;Arylamine N-acetyltransferase 1	Nat2;Nat3;	mmu:17961			
3961	NaN	0.0363	-0.5705	-0.2671	0	A2ADA5	tRNA pseudouridine synthase-like 1	Pusl1	mmu:433813			
3962	NaN	-0.2053	-0.3259	-0.2656	0	O55234	Proteasome subunit beta type-5	Psmb5	mmu:19173	1		
3963	NaN	-0.9132	0.3872	-0.2630	0	Q9D3B1	Very-long-chain (3R)-3-hydroxyacyl-CoA dehydratase 2	Hacd2	mmu:70757			
3964	NaN	-0.2975	-0.7823	-0.2624	0	Q3U1V6	Ubiquitin-conjugating enzyme E2 variant 3	Uevld	mmu:54122			
3965	-0.2618	NaN	NaN	-0.2618	0	Q5SXC3		Vezf1				
3966	NaN	0.4848	-1.0081	-0.2616	0	Q9CZT5	Vasorin	Vasn	mmu:246154			
3967	-0.2611	NaN	NaN	-0.2611	0	O88665	Bromodomain-containing protein 7	Brd7	mmu:26992			
3968	NaN	-0.1366	-0.3851	-0.2609	0	B1APX2	Uncharacterized protein KIAA0930 homolog	5031439G	mmu:223739			
3969	0.4894	NaN	0.0173	-0.2599	0	O35730	E3 ubiquitin-protein ligase RING1	Ring1	mmu:19763		1	
3970	-0.5680	NaN	1.2865	-0.2587	0	Q9D9K3	Cell death regulator Aven	Aven	mmu:74268			1
3971	-0.1319	-0.9050	NaN	-0.2582	0	Q8BK03	Protein FAM73B	Fam73b	mmu:108958			
3972	0.0915	NaN	NaN	-0.2577	0	Q5NC05	Transcription termination factor 2	Ttf2	mmu:74044			
3973	-0.1517	NaN	-0.3636	-0.2576	0	Q9EP72	ER membrane protein complex subunit 7	Emc7	mmu:73024			
3974	-0.2570	NaN	NaN	-0.2570	0	P58069	Ras GTPase-activating protein 2	Rasa2	mmu:114713			
3975	-0.0870	NaN	NaN	-0.2558	0	Q8BRG8	Transmembrane protein 209	Tmem209	mmu:72649			
3976	NaN	0.0226	-0.5341	-0.2557	0	Q9JJL8	Serine--tRNA ligase, mitochondrial	Sars2	mmu:71984			
3977	-0.2556	NaN	NaN	-0.2556	0	Q80TY5	Vacuolar protein sorting-associated protein 13B	Vps13b	mmu:666173			
3978	NaN	NaN	NaN	-0.2555	0	O08664	B-cell CLL/lymphoma 7 protein family member C	Bcl7c	mmu:12055			
3979	NaN	-0.1958	-0.3142	-0.2550	0	Q9CWG9	Biogenesis of lysosome-related organelles complex 1 subunit 2	Bloc1s2	mmu:73689			
3980	NaN	-0.5925	0.0860	-0.2532	0	P97355	Spermine synthase	Sms	mmu:20603			
3981	NaN	-0.2794	-1.2934	-0.2517	0	Q9CQ40	39S ribosomal protein L49, mitochondrial	Mrpl49	mmu:18120			
3982	-0.0285	NaN	0.4807	-0.2510	0	Q8CB77	Transcription elongation factor B polypeptide 3	Tceb3	mmu:27224			
3983	NaN	-1.0523	0.5513	-0.2505	0	Q8BWR4	Ubiquitin carboxyl-terminal hydrolase 40	Usp40	mmu:227334			
3984	-0.5399	0.0403	NaN	-0.2498	0	Q80Y98	Phospholipase DDHD2	Ddhd2	mmu:72108			
3985	-0.2565	NaN	NaN	-0.2495	0	P54818	Galactocerebrosidase	Galc	mmu:14420			
3986	-0.2490	NaN	NaN	-0.2490	0	A2AFQ9		Gemin5				
3987	-0.2480	NaN	NaN	-0.2480	0	P03975	IgE-binding protein	Iap				
3988	-0.4581	-0.0353	NaN	-0.2467	0	A0A0G2JE89	Phospholipid-transporting ATPase	Atp11b				
3989	-0.2457	NaN	NaN	-0.2457	0	Q8BHX1	HAUS augmin-like complex subunit 1	Haus1	mmu:225745			
3990	NaN	0.1598	-0.6505	-0.2454	0	P49282	Natural resistance-associated macrophage protein 2	Slc11a2	mmu:18174	1		
3991	-0.2429	NaN	NaN	-0.2429	0	P59481	VIP36-like protein	Lman2l	mmu:214895			
3992	NaN	0.0146	-0.4994	-0.2424	0	Q8K0Q5	Rho GTPase-activating protein 18	Arhgap18	mmu:73910			
3993	-0.0721	NaN	NaN	-0.2414	0	Q80US4	Actin-related protein 5	Actr5				
3994	-0.2405	NaN	NaN	-0.2405	0	Q8CE50	Sorting nexin-30	Snx30	mmu:209131			
3995	-1.3483	NaN	NaN	-0.2402	0	Q6ZQL4	WD repeat-containing protein 43	Wdr43	mmu:72515			
3996	NaN	0.0198	-0.4997	-0.2399	0	Q64331	Unconventional myosin-VI	Myo6				
3997	-0.6920	0.2123	NaN	-0.2399	0	Q9D081	UDP-N-acetylglucosamine transferase subunit ALG14 homolog	Alg14	mmu:66789			
3998	-0.0172	NaN	NaN	-0.2393	0	P21619	Lamin-B2	Lmnb2	mmu:16907			
3999	-0.3903	-0.0880	NaN	-0.2392	0	Q8R033	LYR motif-containing protein 2	Lyrm2	mmu:108755			
4000	NaN	-0.2979	-0.1662	-0.2320	0	Q6PAV2	Probable E3 ubiquitin-protein ligase HERC4	Herc4	mmu:67345		1	
4001	-0.2308	NaN	NaN	-0.2308	0	Q91Y03		Pcdhb16	mmu:93887			
4002	NaN	-0.9204	0.1447	-0.2295	0	Q99PL6	UBX domain-containing protein 6	Ubxn6	mmu:66530			
4003	-0.2282	NaN	NaN	-0.2282	0	P54751	CMP-N-acetylneuraminate-beta-galactosamide-alpha-2,3-sialyltransferase 1	St3gal1	mmu:20442			
4004	NaN	0.1127	-0.5676	-0.2274	0	Q9QY33	Tetraspanin-3	Tspan3	mmu:56434			
4005	-0.2265	NaN	NaN	-0.2265	0	Q9WUU9	Germinal-center associated nuclear protein	Mcm3ap	mmu:54387			
4006	-1.7154	NaN	0.4397	-0.2231	0	Q64511	DNA topoisomerase 2-beta	Top2b	mmu:21974			
4007	-0.2215	NaN	NaN	-0.2215	0	Q99JP6	Homer protein homolog 3	Homer3	mmu:26558			
4008	-0.2310	NaN	NaN	-0.2194	0	Q6PR54	Telomere-associated protein RIF1	Rif1	mmu:51869			
4009	-0.2192	NaN	NaN	-0.2192	0	Q8BFX1	E3 ubiquitin-protein ligase RNF187	Rnf187	mmu:108660		1	
4010	-0.6801	0.2431	NaN	-0.2185	0	Q64735	Complement component receptor 1-like protein	Cr1l	mmu:12946			
4011	-0.1545	NaN	NaN	-0.2145	0	Q80WE4	Kinesin-like protein KIF20B	Kif20b	mmu:240641			
4012	-0.6580	NaN	0.3179	-0.2144	0	O54791	Transcription factor MafF	Maff	mmu:17133			
4013	NaN	-0.0137	-0.4136	-0.2136	0	P49282	Natural resistance-associated macrophage protein 2	Slc11a2	mmu:18174	1		
4014	-0.4444	NaN	NaN	-0.2127	0	E9PZ11		Zfp651	mmu:270210			
4015	NaN	0.1045	-0.5289	-0.2122	0	Q8K4X7	1-acyl-sn-glycerol-3-phosphate acyltransferase delta	Agpat4	mmu:68262			
4016	-0.2112	NaN	NaN	-0.2112	0	Q922P9	Putative oxidoreductase GLYR1	Glyr1	mmu:74022			
4017	-0.2097	NaN	NaN	-0.2097	0	Q61730	Interleukin-1 receptor accessory protein	Il1rap	mmu:16180			
4018	-0.2089	NaN	NaN	-0.2089	0	E9Q467		Abcc4				
4019	NaN	0.7189	-0.0478	-0.2088	0	Q9JI99	Sphingosine-1-phosphate phosphatase 1	Sgpp1	mmu:81535			
4020	NaN	-0.3049	-0.1073	-0.2061	0	Q08274	Dystrophia myotonica WD repeat-containing protein	Dmwd	mmu:13401			
4021	-0.6577	NaN	NaN	-0.2055	0	P40630	Transcription factor A, mitochondrial	Tfam	mmu:21780			

4022	1.1603	0.6857	NaN	-0.2039	0	O88291	DBIRD complex subunit ZNF326	Znf326	mmu:54367				
4023	-0.0068	NaN	NaN	-0.2037	0	Q3TYY8		Pwwp2b	mmu:101631				
4024	NaN	NaN	NaN	-0.2035	0	A2CG63	AT-rich interactive domain-containing protein 4B	Arid4b	mmu:94246				
4025	-1.0224	0.3174	NaN	-0.2025	0	Q8R107	PRELI domain-containing protein 1, mitochondrial	Prelid1	mmu:66494				
4026	NaN	-0.4931	0.0933	-0.1999	0	Q80ZK9	WD and tetratricopeptide repeats protein 1	Wdtc1	mmu:230796			1	
4027	NaN	-1.7348	0.1010	-0.1984	0	Q9QZM4	Tumor necrosis factor receptor superfamily member 10B	Tnfrsf10b	mmu:21933				1
4028	NaN	-0.1977	NaN	-0.1977	0	O88396	GrpE protein homolog 2, mitochondrial	Grpel2	mmu:17714				
4029	0.3643	NaN	NaN	-0.1966	0	Q6ZPR5	Sphingomyelin phosphodiesterase 4	Smpd4	mmu:77626				
4030	NaN	0.1987	0.9756	-0.1957	0	Q9D6T0	Nitric oxide synthase-interacting protein	Nosip	mmu:66394				
4031	NaN	0.1160	-0.6304	-0.1950	0	P35441	Thrombospondin-1	Thbs1		1			
4032	-0.2092	-0.1802	NaN	-0.1947	0	B0V2N1	Receptor-type tyrosine-protein phosphatase S	Ptprs	mmu:19280		1		
4033	-0.1940	NaN	NaN	-0.1940	0	Q8BVE8	Histone-lysine N-methyltransferase NSD2	Whsc1	mmu:107823				
4034	NaN	-0.6499	0.2648	-0.1925	0	P15702	Leukosialin	Spn	mmu:20737				1
4035	0.4399	NaN	-0.8248	-0.1925	0	P49722	Proteasome subunit alpha type-2	Psma2	mmu:19166				
4036	NaN	-0.3795	[illegible]	-0.1901	0	Q9D020	Cytosolic 5-nucleotidase 3A	Nt5c3a	mmu:107569				
4037	-1.6505	0.1119	NaN	-0.1897	0	Q9JKP5	Muscleblind-like protein 1	Mbnl1	mmu:56758				
4038	NaN	-0.2837	-0.0926	-0.1882	0	Q68FG2		Sptbn2	mmu:20743				
4039	0.3330	NaN	NaN	-0.1870	0	O35954	Membrane-associated phosphatidylinositol transfer protein 1	Pitpnm1	mmu:18739				
4040	-0.1868	NaN	NaN	-0.1868	0	Q8BX09	Retinoblastoma-binding protein 5	Rbbp5	mmu:213464				
4041	NaN	-0.1858	NaN	-0.1858	0	Q9WUU7	Cathepsin Z	Ctsz	mmu:64138				
4042	-0.3066	NaN	NaN	-0.1852	0	Q99ML1	Bcl-2-binding component 3	Bbc3	mmu:170770				1
4043	0.7421	-0.6858	NaN	-0.1841	0	Q9JK30	Origin recognition complex subunit 3	Orc3	mmu:50793				
4044	NaN	NaN	-0.1836	-0.1836	0	Q80TC5	Pogo transposable element with KRAB domain	Pogk	mmu:71592				1
4045	NaN	-0.5116	0.1465	-0.1825	0	Q8BUN5	Mothers against decapentaplegic homolog 3	Smad3	mmu:17127				
4046	NaN	-1.7925	1.4303	-0.1811	0	Q9QXG2	Rab proteins geranylgeranyltransferase component A 1	Chm					
4047	NaN	0.3396	-0.6977	-0.1791	0	Q9EPT5	Solute carrier organic anion transporter family member 2A1	Slco2a1	mmu:24059				
4048	NaN	NaN	NaN	-0.1789	0	Q9D2G5	Synaptojanin-2	Synj2	mmu:20975		1		
4049	NaN	-0.6300	0.2784	-0.1758	0	P51791	Chloride channel protein;H(+)/Cl(-) exchange transporter 3	Clcn3	mmu:12725				
4050	NaN	0.1097	-0.4593	-0.1748	0	Q91ZP6	NEDD4 family-interacting protein 2	Ndfip2					
4051	-0.1746	NaN	NaN	-0.1746	0	E9Q4Z2	Acetyl-CoA carboxylase 2;Biotin carboxylase	Acacb	mmu:100705				
4052	NaN	0.9037	-1.2486	-0.1725	0	P36552	Oxygen-dependent coproporphyrinogen-III oxidase, mitochondrial	Cpox	mmu:12892				
4053	-0.3591	NaN	NaN	-0.1724	0	Q9CZ96	Zinc finger CCHC-type and RNA-binding motif-containing protein 1	Zcrb1	mmu:67197				
4054	NaN	NaN	-0.1690	-0.1690	0	Q8BS95	Golgi pH regulator	Gpr89a	mmu:67549				
4055	NaN	-0.0382	-0.2996	-0.1689	0	Q80UY1	UPF0586 protein C9orf41 homolog		mmu:67383				
4056	0.1526	NaN	-0.4904	-0.1689	0	Q9CWX9	Probable ATP-dependent RNA helicase DDX47	Ddx47	mmu:67755				
4057	-0.7269	NaN	NaN	-0.1679	0	P43135	COUP transcription factor 2	Nr2f2;Nr2f	mmu:11819				
4058	NaN	0.2914	-0.6256	-0.1671	0	Q8BNY6	Neuronal calcium sensor 1	Ncs1	mmu:14299				
4059	-0.1651	NaN	NaN	-0.1651	0	Q8R205	Zinc finger CCCH domain-containing protein 10	Zc3h10	mmu:103284				
4060	-0.1645	NaN	NaN	-0.1645	0	Q9CQL5	39S ribosomal protein L18, mitochondrial	Mrpl18	mmu:67681				
4061	NaN	-1.6110	-0.3993	-0.1627	0	Q9WVA2	Mitochondrial import inner membrane translocase subunit Tim8 A;Putative mitochondrial import	Timm8a1;T	mmu:30058				
4062	NaN	-0.4251	-0.2777	-0.1618	0	Q8C7E7	Starch-binding domain-containing protein 1	Stbd1	mmu:52331				
4063	-0.1616	NaN	NaN	-0.1616	0	Q5SWW4	Mediator of RNA polymerase II transcription subunit 13	Med13	mmu:327987				
4064	NaN	NaN	-0.1605	-0.1605	0	Q9WUB0	RanBP-type and C3HC4-type zinc finger-containing protein 1	Rbck1	mmu:24105				
4065	0.0288	NaN	0.3022	-0.1598	0	Q8C147	Dedicator of cytokinesis protein 8	Dock8	mmu:76088				
4066	NaN	0.0797	-0.0689	-0.1589	0	Q9WUL7	ADP-ribosylation factor-like protein 3	Arl3	mmu:56350				
4067	-0.1589	NaN	NaN	-0.1589	0	P70700	DNA-directed RNA polymerase I subunit RPA2	Polr1b	mmu:20017				
4068	NaN	0.2996	0.4443	-0.1573	0	Q9D8Y0	EF-hand domain-containing protein D2	Efhd2					
4069	-0.1566	NaN	NaN	-0.1566	0	Q8CGC6	RNA-binding protein 28	Rbm28	mmu:68272				
4070	-0.6688	NaN	NaN	-0.1555	0	Q8BTY8	Sec1 family domain-containing protein 2	Scfd2	mmu:212986				
4071	-0.5593	NaN	0.2497	-0.1548	0	O08586	Phosphatidylinositol 3,4,5-trisphosphate 3-phosphatase and dual-specificity protein phosphatase I	Pten	mmu:19211		1		1
4072	-0.1529	NaN	NaN	-0.1529	0	A2ADA6		Acap3					
4073	-0.1507	NaN	NaN	-0.1507	0	Q9CPY1	39S ribosomal protein L51, mitochondrial	Mrpl51	mmu:66493				
4074	-0.1498	NaN	NaN	-0.1498	0	Q3TUF7	YEATS domain-containing protein 2	Yeats2	mmu:208146				
4075	-0.1492	NaN	NaN	-0.1492	0	Q5KU39	Vacuolar protein sorting-associated protein 41 homolog	Vps41	mmu:218035				
4076	0.0901	NaN	NaN	-0.1487	0	P29595	NEDD8	Nedd8	mmu:18002				
4077	-0.2837	-0.0127	NaN	-0.1482	0	Q9QXG9	TERF1-interacting nuclear factor 2	Tinf2					
4078	NaN	0.2348	-0.5293	-0.1473	0	Q9JJA4	Ribosome biogenesis protein WDR12	Wdr12	mmu:57750				
4079	-0.1443	NaN	NaN	-0.1443	0	Q6NZQ6	Zinc finger protein 740	Zfp740;Znf	mmu:68744				
4080	NaN	-0.2620	-0.0262	-0.1441	0	F7CFA5		Glud1					
4081	-0.1437	NaN	NaN	-0.1437	0	A2AHC3	Calmodulin-regulated spectrin-associated protein 1	Camsap1	mmu:227634				
4082	-0.1436	NaN	NaN	-0.1436	0	Q32P12-2	Uncharacterized protein C17orf53 homolog						
4083	NaN	-0.1082	-0.1754	-0.1418	0	Q6A037	NEDD4-binding protein 1	N4bp1	mmu:80750				
4084	-0.1398	NaN	NaN	-0.1398	0	Q9JIA7	Sphingosine kinase 2	Sphk2	mmu:56632				
4085	-0.1374	NaN	NaN	-0.1374	0	D3Z2R5		Sepn1	mmu:74777	1			
4086	0.2018	-0.4760	NaN	-0.1371	0	Q9JLJ5	Elongation of very long chain fatty acids protein 1	Elovl1	mmu:54325				
4087	-0.1361	NaN	NaN	-0.1361	0	Q91ZM2	SH2B adapter protein 1	Sh2b1	mmu:20399				
4088	NaN	0.1465	-0.4159	-0.1347	0	Q8C4P0-2							
4089	-0.1343	NaN	NaN	-0.1343	0	Q3UEB3	Poly(U)-binding-splicing factor PUF60	Puf60	mmu:67959				
4090	NaN	NaN	-0.1341	-0.1341	0	Q9CZX9	ER membrane protein complex subunit 4	Emc4	mmu:68032				
4091	-0.2969	0.0339	NaN	-0.1315	0	Q8R2U2		BC027231	mmu:212547				
4092	NaN	-0.2839	0.0212	-0.1313	0	Q91V81	RNA-binding protein 42	Rbm42	mmu:68035				
4093	NaN	-0.2187	-0.5202	-0.1312	0	P54923	[Protein ADP-ribosylarginine] hydrolase	Adprh	mmu:11544				
4094	NaN	0.0971	-0.3551	-0.1290	0	Q02013	Aquaporin-1	Aqp1	mmu:11826	1			
4095	NaN	-0.7538	0.4985	-0.1276	0	Q8BRH4	Histone-lysine N-methyltransferase;Histone-lysine N-methyltransferase 2C	Kmt2c					
4096	NaN	0.7760	-0.1750	-0.1246	0	Q9D2R0	Acetoacetyl-CoA synthetase	Aacs	mmu:78894				
4097	[illegible]	-0.1441	NaN	-0.1244	0	Q8BLY7	Hermansky-Pudlak syndrome 6 protein homolog	Hps6	mmu:20170				
4098	-0.1243	NaN	NaN	-0.1243	0	Q99KW3	TRIO and F-actin-binding protein	Triobp	mmu:110253				
4099	-0.1242	NaN	NaN	-0.1242	0	Q91VV4	DENN domain-containing protein 2D	Dennd2d	mmu:72121				
4100	NaN	-0.1209	NaN	-0.1209	0	Q8BKE9	Intraflagellar transport protein 74 homolog	Ift74	mmu:67694				
4101	-0.2455	[illegible]	NaN	-0.1180	0	Q9DD02	Protein Hikeshi	L7rn6	mmu:67669				
4102	-0.3288	NaN	-0.1594	-0.1170	0	P08249	Malate dehydrogenase, mitochondrial	Mdh2	mmu:17448				
4103	-0.0265	NaN	-0.2000	-0.1133	0	Q9WTQ8	Mitochondrial import inner membrane translocase subunit Tim23	Timm23					
4104	-0.1111	NaN	NaN	-0.1111	0	Q3UHX0	Nucleolar protein 8	Nol8	mmu:70930				
4105	-0.1111	NaN	NaN	-0.1111	0	Q810S1	Calcium uniporter regulatory subunit MCUb, mitochondrial	Ccdc109b	mmu:66815				
4106	NaN	0.5939	-0.8159	-0.1110	0	A0A087WPH7		Psmc3					
4107	NaN	-0.8834	-0.1059	-0.1107	0	Q9QWT9	Kinesin-like protein KIFC1	Kifc1	mmu:16580				
4108	NaN	0.3848	-0.6059	-0.1106	0	Q6PB93	Polypeptide N-acetylgalactosaminyltransferase 2;Polypeptide N-acetylgalactosaminyltransferase 2	Galnt2	mmu:108148				
4109	NaN	NaN	-0.1103	-0.1103	0	Q8C522	Endonuclease domain-containing 1 protein	Endod1	mmu:71946				
4110	-0.1092	NaN	NaN	-0.1092	0	O35207	Cyclin-dependent kinase 2-associated protein 1	Cdk2ap1	mmu:13445				
4111	-0.1772	NaN	-0.0399	-0.1085	0	Q80TN7	Neuron navigator 3;Neuron navigator 1	Nav3;Nav2;Nav1					
4112	NaN	NaN	-0.1063	-0.1063	0	Q8C5W3	Tubulin-specific chaperone cofactor E-like protein	Tbcel	mmu:272589				
4113	-0.1058	NaN	NaN	-0.1058	0	Q9R078	5-AMP-activated protein kinase subunit beta-1	Prkab1	mmu:19079				
4114	-0.0532	0.5631	NaN	-0.1040	0	Q9D1R2	Protein KTI12 homolog	Kti12	mmu:100087				
4115	-0.1030	NaN	NaN	-0.1030	0	O35250	Exocyst complex component 7	Exoc7	mmu:53413				
4116	NaN	NaN	NaN	-0.1028	0	P29452	Caspase-1;Caspase-1 subunit p20;Caspase-1 subunit p10	Casp1	mmu:12362				1
4117	NaN	0.1127	0.3173	-0.1017	0	P47226	Testin	Tes					
4118	NaN	-0.0135	-0.1883	-0.1009	0	A0A140LI20							
4119	-0.1148	NaN	NaN	-0.1006	0	Q8K1C9	Leucine-rich repeat-containing protein 41	Lrrc41	mmu:230654			1	
4120	-0.3395	NaN	NaN	-0.0990	0	Q3TZM9	GDP-Man:Man(3)GlcNAc(2)-PP-Dol alpha-1,2-mannosyltransferase	Alg11	mmu:207958				
4121	-1.1711	NaN	NaN	-0.0986	0	Q3U6U5	Putative GTP-binding protein 6	Gtpbp6	mmu:107999				
4122	NaN	NaN	-0.0971	-0.0971	0	A0A0G2JEY4	Uncharacterized protein C7orf50 homolog						
4123	0.3252	NaN	-0.0284	-0.0964	0	Q99JB8	Protein kinase C and casein kinase II substrate protein 3	Pacsin3	mmu:80708				
4124	NaN	0.3075	-0.4993	-0.0959	0	Q8BFY6	Peflin	Pef1	mmu:67898				
4125	NaN	NaN	-0.0951	-0.0951	0	Q9D114	Guanosine-3,5-bis(diphosphate) 3-pyrophosphohydrolase MESH1	Hddc3	mmu:68695				
4126	NaN	NaN	-0.0949	-0.0949	0	Q5EBP3	Armadillo repeat-containing protein 5	Armc5	mmu:233912				
4127	NaN	0.1924	-0.3787	-0.0931	0	Q9R1P3	Proteasome subunit beta type-2	Psmb2	mmu:26445				
4128	-0.0931	NaN	NaN	-0.0931	0	Q9EQH4	Transcription initiation factor TFIID subunit 8	Taf8	mmu:63856				
4129	-1.9223	NaN	NaN	-0.0921	0	O88708	Origin recognition complex subunit 4	Orc4	mmu:26428				
4130	0.3799	-0.8258	NaN	-0.0917	0	Q80U04	E3 ubiquitin-protein ligase Praja-2	Pja2	mmu:224938			1	
4131	NaN	-0.3358	0.1540	-0.0909	0	Q91UZ5	Inositol monophosphatase 2	Impa2	mmu:114663				
4132	NaN	NaN	-0.0908	-0.0908	0	Q9WVK0	Type-1 angiotensin II receptor-associated protein	Agtrap	mmu:11610				
4133	-0.4603	NaN	0.8822	-0.0894	0	Q6A0A2	La-related protein 4B	Larp4b	mmu:217980				
4134	NaN	[illegible]	-0.1834	-0.0891	0	Q91ZB7	Mas-related G-protein coupled receptor member E	Mrgpre	mmu:244238				
4135	NaN	-0.0110	-0.1651	-0.0881	0	Q8QZS3	Folliculin	Flcn	mmu:216805				
4136	NaN	0.1005	-0.2765	-0.0880	0	P58466	Carboxy-terminal domain RNA polymerase II polypeptide A small phosphatase 1	Ctdsp1	mmu:227292		1		
4137	-0.4898	NaN	2.7102	-0.0870	0	Q9DBE9	pre-rRNA processing protein FTSJ3	Ftsj3	mmu:56095				
4138	-0.0870	NaN	NaN	-0.0870	0	E9Q3P4		Cenpf	mmu:108000				
4139	-0.0861	NaN	NaN	-0.0861	0	Q00420	GA-binding protein subunit beta-1	Gabpb1	mmu:14391				
4140	0.3012	NaN	-0.4685	-0.0837	0	Q01237	3-hydroxy-3-methylglutaryl coenzyme A reductase;3-hydroxy-3-methylglutaryl-coenzyme A reduct	Hmgcr	mmu:15357				
4141	NaN	-0.6566	-0.2571	-0.0833	0	Q9CR25	Diphthamide biosynthesis protein 2	Dph2	mmu:67728				
4142	-0.0833	NaN	NaN	-0.0833	0	Q91WK7	Ankyrin repeat domain-containing protein 54	Ankrd54	mmu:223690				
4143	-0.3642	NaN	NaN	-0.0827	0	Q7TMF2	3-5 exoribonuclease 1	Eri1	mmu:67276				
4144	NaN	0.2326	-0.3970	-0.0822	0	Q91VN0	Low-density lipoprotein receptor-related protein 5	Lrp5	mmu:16973				
4145	NaN	2.5427	-0.2685	-0.0811	0	Q9D8S3	ADP-ribosylation factor GTPase-activating protein 3	Arfgap3	mmu:66251				
4146	NaN	NaN	NaN	-0.0804	0	Q8R2R6	Mitochondrial ribosome-associated GTPase 1	Mtg1	mmu:212508				
4147	NaN	0.2171	1.3131	-0.0796	0	Q64FW2	All-trans-retinol 13,14-reductase	Retsat	mmu:67442				

4148	NaN	-0.1276	-0.0299	-0.0788	0	Q9D1F3	Uncharacterized protein CXorf40 homolog		mmu:68618			
4149	0.8363	-0.2760	NaN	-0.0787	0	Q0VGY8	Protein TANC1	Tanc1	mmu:66860			
4150	-0.0758	NaN	NaN	-0.0758	0	P06797	Cathepsin L1;Cathepsin L1 heavy chain;Cathepsin L1 light chain	Ctsl	mmu:13039			
4151	-0.9473	NaN	NaN	-0.0757	0	Q3UVL4	Vacuolar protein sorting-associated protein 51 homolog	Vps51	mmu:68505			
4152	1.1268	NaN	-0.6923	-0.0752	0	P48725	Pericentrin	Pcnt	mmu:18541			
4153	-0.0740	NaN	NaN	-0.0740	0	Q921E6	Polycomb protein EED	Eed	mmu:13626			
4154	0.6440	NaN	0.5763	-0.0734	0	Q9D786	HAUS augmin-like complex subunit 5	Haus5	mmu:71909			
4155	NaN	-1.0010	-0.6676	-0.0722	0	Q3U487	E3 ubiquitin-protein ligase HECTD3	Hectd3	mmu:76608			1
4156	0.9222	-0.3009	NaN	-0.0717	0	Q61543	Golgi apparatus protein 1	Glg1	mmu:20340			
4157	-0.6012	-0.7196	NaN	-0.0712	0	Q9QZH6	Evolutionarily conserved signaling intermediate in Toll pathway, mitochondrial	Ecsit	mmu:26940			
4158	0.2339	NaN	-0.3763	-0.0712	0	A2ACQ1		Dhx35				
4159	-0.5481	0.4062	NaN	-0.0710	0	Q7TPE5	Probable RNA polymerase II nuclear localization protein SLC7A6OS	Slc7a6os	mmu:66432			
4160	NaN	NaN	-0.5183	-0.0699	0	Q6PE15	Mycophenolic acid acyl-glucuronide esterase, mitochondrial	Abhd10	mmu:213012			
4161	NaN	0.2757	-0.4136	-0.0689	0	Q8BGS0	Protein MAK16 homolog	Mak16	mmu:67920			
4162	NaN	-0.8014	0.6643	-0.0686	0	Q8VCQ3	Nuclear receptor-binding factor 2	Nrbf2	mmu:641340			
4163	-0.0684	NaN	NaN	-0.0684	0	Q91VT4	Carbonyl reductase family member 4	Cbr4	mmu:234309			
4164	-0.4895	NaN	NaN	-0.0679	0	Q6ZQK0	Condensin-2 complex subunit D3	Ncapd3	mmu:78658			
4165	-0.8564	NaN	NaN	-0.0662	0	Q8CCM6-2						
4166	-0.0641	NaN	NaN	-0.0641	0	Q9CQL4	39S ribosomal protein L20, mitochondrial	Mrpl20	mmu:66448			
4167	NaN	-0.1392	0.2795	-0.0637	0	Q9CR00	26S proteasome non-ATPase regulatory subunit 9	Psmd9	mmu:67151			
4168	NaN	0.2515	-0.3756	-0.0620	0	Q8VEC4	Calcium homeostasis modulator protein 2	Calhm2	mmu:72691			
4169	NaN	-0.0333	-0.0896	-0.0614	0	P56389	Cytidine deaminase	Cda	mmu:72269			
4170	NaN	NaN	NaN	-0.0614	0	Q9CY64	Biliverdin reductase A	Blvra	mmu:109778			
4171	-0.0607	NaN	NaN	-0.0607	0	Q9JLV2	Short transient receptor potential channel 4-associated protein	Trpc4ap	mmu:56407			1
4172	NaN	0.2386	-0.3595	-0.0604	0	Q9CR80	Protein FAM32A	Fam32a	mmu:67922			
4173	-0.0602	NaN	NaN	-0.0602	0	Q99MS8	Tubulin polyglutamylase complex subunit 1	Tpgs1	mmu:110012			
4174	0.4511	NaN	-0.5667	-0.0578	0	A0A0A0MQH5		Macf1				
4175	NaN	NaN	-0.0577	-0.0577	0	Q3TNH5	Protein FAM172A	Fam172a	mmu:68675			
4176	-0.0955	NaN	NaN	-0.0567	0	O08789	Max-binding protein MNT	Mnt	mmu:17428			
4177	0.2388	NaN	-0.1926	-0.0542	0	Q91WS7	DEP domain-containing protein 7	Depdc7	mmu:211896			
4178	NaN	-0.0604	-0.0449	-0.0527	0	Q9CZG3	COMM domain-containing protein 8	Commd8	mmu:27784			
4179	0.1231	NaN	-0.3993	-0.0508	0	Q9D3E6	Cohesin subunit SA-1	Stag1	mmu:20842			
4180	-0.0496	NaN	NaN	-0.0496	0	Q8C7Q4	RNA-binding protein 4	Gm21992;I	mmu:19653			
4181	-0.0472	NaN	NaN	-0.0472	0	H3BJI7		Methig1;Mettl7a1;UbiE2;Mettl7a2				
4182	0.3398	NaN	NaN	-0.0450	0	P59997	Lysine-specific demethylase 2A	Kdm2a	mmu:225876			
4183	NaN	-0.0052	-0.0832	-0.0442	0	A2AH22	Activating molecule in BECN1-regulated autophagy protein 1	Ambra1	mmu:228361			
4184	-0.2228	0.1355	NaN	-0.0437	0	Q3UHA3	Spatacsin	Spg11	mmu:214585			
4185	-0.0731	-0.0934	NaN	-0.0428	0	Q3TL26	Dimethyladenosine transferase 2, mitochondrial	Gm1818;Tf	mmu:15278			
4186	-0.0425	NaN	NaN	-0.0425	0	E9PVP1		Aim1	mmu:11630			
4187	-0.3275	0.0086	NaN	-0.0416	0	Q68FL4	Adenosylhomocysteinase;Putative adenosylhomocysteinase 3	Ahcyl2	mmu:74340			
4188	NaN	0.2419	-0.3238	-0.0409	0	Q9CRB0	Sorting nexin-24	Snx24	mmu:69226			
4189	0.9558	-1.0331	NaN	-0.0386	0	Q9CRB9	MICOS complex subunit Mic19	Chchd3	mmu:66075			
4190	NaN	-0.4673	0.3909	-0.0382	0	Q9JHZ2	Progressive ankylosis protein	Ankh	mmu:11732			
4191	-0.0370	NaN	NaN	-0.0370	0	Q8BGB8	Ubiquinone biosynthesis protein COQ4 homolog, mitochondrial	Coq4	mmu:227683			
4192	NaN	NaN	-0.0365	-0.0365	0	Q9R0M8	UDP-galactose translocator	Slc35a2	mmu:22232			
4193	NaN	-0.9146	-0.5851	-0.0358	0	Q3ULF4	Paraplegin	Spg7	mmu:234847			
4194	NaN	0.5497	-0.6175	-0.0339	0	Q9CWS4	Integrator complex subunit 11	Cpsf3l	mmu:71957			
4195	-0.0332	NaN	NaN	-0.0332	0	F6ZDS4	Nucleoprotein TPR	Tpr	mmu:108989			
4196	-0.0321	NaN	NaN	-0.0321	0	Q6URW6	Myosin-14	Myh14	mmu:71960			
4197	NaN	0.3013	-0.3655	-0.0321	0	O08601	Microsomal triglyceride transfer protein large subunit	Mttp	mmu:17777			
4198	NaN	0.1648	-0.2287	-0.0320	0	Q9CQL0	Protein N-lysine methyltransferase METTL21A	Mettl21A	mmu:67099			
4199	-0.0311	NaN	NaN	-0.0311	0	Q03347	Runt-related transcription factor 1	Runx1				
4200	-0.0306	NaN	NaN	-0.0306	0	Q9DBU6	Serine/Arginine-related protein 53	Rsrc1	mmu:66880			
4201	NaN	-0.1370	0.0767	-0.0302	0	Q9CQR6	Serine/threonine-protein phosphatase 6 catalytic subunit;Serine/threonine-protein phosphatase (	Ppp6c	mmu:67857		1	
4202	-0.0296	NaN	NaN	-0.0296	0	P62700	Protein yippee-like 5	Ypel5	mmu:383295			
4203	-0.0294	NaN	NaN	-0.0294	0	Q9DBA9	General transcription factor IIH subunit 1	Gtf2h1	mmu:14884			
4204	NaN	0.4184	-0.4709	-0.0263	0	Q60866	Phosphotriesterase-related protein	Pter	mmu:19212			
4205	NaN	-0.5272	-0.1548	-0.0247	0	P59114	Phosphorylated CTD-interacting factor 1	Pcif1	mmu:228866			
4206	-0.2135	0.1642	NaN	-0.0247	0	Q792Z1		Try10	mmu:436522			
4207	1.0111	NaN	NaN	-0.0235	0	Q924H2	Mediator of RNA polymerase II transcription subunit 15	Med15				
4208	NaN	NaN	-0.0232	-0.0232	0	Q8K4D3	Proton-coupled amino acid transporter 1	Slc36a1	mmu:215335			
4209	-0.0224	NaN	NaN	-0.0224	0	Q9D011	M-phase-specific PLK1-interacting protein	Gm7102;N	mmu:66308			
4210	1.1120	NaN	1.1048	-0.0218	0	Q925N2	Sideroflexin-2	Sfxn2	mmu:94279			
4211	-0.0210	NaN	NaN	-0.0210	0	P29352	Protein-tyrosine-phosphatase;Tyrosine-protein phosphatase non-receptor type 22	Ptpn22	mmu:19260		1	
4212	0.0913	-0.2593	NaN	-0.0203	0	A0A087WQY5		Srcap				
4213	NaN	NaN	-0.0192	-0.0192	0	Q8R080	G2 and S phase-expressed protein 1	Gtse1	mmu:29870			
4214	-0.3177	-0.0963	NaN	-0.0181	0	Q7TME2	Sperm-associated antigen 5	Spag5	mmu:54141			
4215	-0.0657	0.0305	NaN	-0.0176	0	Q8K3G9	DCC-interacting protein 13-beta	Appl2	mmu:216190			
4216	NaN	1.0915	-1.1250	-0.0168	0	P63260	Actin, cytoplasmic 2;Actin, cytoplasmic 2, N-terminally processed	Actg1	mmu:11465			
4217	-0.0158	NaN	NaN	-0.0158	0	Q9Z1Q2	Abhydrolase domain-containing protein 16A	Abhd16a	mmu:193742			
4218	NaN	0.1084	-0.1355	-0.0136	0	Q9Z1G4	V-type proton ATPase subunit a;V-type proton ATPase 116 kDa subunit a isoform 1	Atp6v0a1	mmu:11975			
4219	-0.0126	NaN	NaN	-0.0126	0	Q8VCI7	ATPase SWSAP1	Swsap1	mmu:66962			
4220	NaN	[illegible]	-0.0200	-0.0118	0	Q6NS82	Protein FAM134A	Fam134a	mmu:227298			
4221	NaN	0.1996	-0.2224	-0.0114	0	Q9CQ85	Mitochondrial import inner membrane translocase subunit Tim22	Timm22	mmu:56322			
4222	-0.0114	NaN	NaN	-0.0114	0	Q9CY28	GTP-binding protein 8	Gtpbp8	mmu:66067			
4223	NaN	0.1032	0.1526	-0.0107	0	Q8CE96	tRNA (adenine(58)-N(1))-methyltransferase non-catalytic subunit TRM6	Trmt6	mmu:66926			
4224	NaN	-0.4925	0.4089	-0.0104	0	Q8K3A0	Iron-sulfur cluster co-chaperone protein HscB, mitochondrial	Hscb	mmu:100900			
4225	NaN	0.5861	-0.0659	-0.0084	0	Q9D0V7	Receptor-binding cancer antigen expressed on SiSo cells	Ebag9	mmu:55960			
4226	NaN	-0.0726	0.0621	-0.0053	0	Q9D727	Uncharacterized protein C6orf226 homolog		mmu:67101			
4227	NaN	0.4140	-0.4243	-0.0052	0	P62069	Ubiquitin carboxyl-terminal hydrolase 12;Ubiquitin carboxyl-terminal hydrolase 46	Usp46;Usp	mmu:69727			
4228	-0.0030	NaN	NaN	-0.0030	0	Q8K190	SAYSvFN domain-containing protein 1	Saysd1	mmu:67509			
4229	0.2567	-0.2930	NaN	[illegible]	0	Q3UMY5	Echinoderm microtubule-associated protein-like 4	Eml4	mmu:78798			
4230	-0.1526	NaN	NaN	[illegible]	0	Q571H0	Nucleolar pre-ribosomal-associated protein 1	Urb1	mmu:207932			
4231	NaN	-0.1180	0.1179	[illegible]	0	Q921Y4	Molybdate-anion transporter	Mfsd5	mmu:106073			
4232	NaN	0.5807	0.3011	[illegible]	0	Q9D7B1	tRNA-dihydrouridine(20) synthase [NAD(P)+]-like	Dus2	mmu:66369			
4233	[illegible]	NaN	NaN	[illegible]	0	B8JJ67		2310061I04Rik				
4234	NaN	NaN	0.4461	[illegible]	0	P23708	Nuclear transcription factor Y subunit alpha	Nfya	mmu:18044			
4235	[illegible]	NaN	NaN	0.0039	0	Q9CWQ0	Diphthine synthase	Dph5	mmu:69740			
4236	NaN	-0.8785	0.8889	0.0052	0	P10810	Monocyte differentiation antigen CD14	Cd14	mmu:12475			
4237	0.8350	NaN	-0.8240	0.0055	0	Q3UJU9	Regulator of microtubule dynamics protein 3	Rmdn3	mmu:67809			
4238	NaN	0.4750	-0.2021	0.0089	0	E9PWG2		Trappc8				
4239	0.1760	NaN	-0.1551	0.0105	0	Q6P3A8	2-oxoisovalerate dehydrogenase subunit beta, mitochondrial	Bckdhb	mmu:12040			
4240	0.2556	NaN	-0.2285	0.0136	0	Q8CHY3	Dymeclin	Dym	mmu:69190			
4241	NaN	0.5909	-0.5597	0.0156	0	P61963	DDB1- and CUL4-associated factor 7	Dcaf7	mmu:71833			1
4242	NaN	0.1754	-0.0315	0.0157	0	Q8BH55	Threonine synthase-like 1	Thnsl1	mmu:208967			
4243	NaN	-0.3184	0.3504	0.0160	0	Q9D394	Protein RUFY3	Rufy3	mmu:52822			
4244	-0.4633	NaN	0.0687	0.0165	0	Q80TV8	CLIP-associating protein 1	Clasp1	mmu:76707			
4245	-0.2567	0.2902	NaN	0.0167	0	Q8R2L5	28S ribosomal protein S18c, mitochondrial	Mrps18c	mmu:68735			
4246	0.0485	NaN	NaN	0.0183	0	Q04690	Neurofibromin	Nf1	mmu:18015			
4247	0.0187	NaN	NaN	0.0187	0	Q9D032	Single-stranded DNA-binding protein 3	Ssbp3	mmu:72475			
4248	0.2047	NaN	NaN	0.0202	0	Q66X05	NACHT, LRR and PYD domains-containing protein 4F	Nlrp4f				
4249	NaN	0.0785	-0.0368	0.0208	0	Q8BH65	Protein DENND6A	Dennd6a	mmu:211922			
4250	0.0220	NaN	NaN	0.0220	0	Q6PDK2	Histone-lysine N-methyltransferase;Histone-lysine N-methyltransferase 2D	Kmt2d				
4251	0.0221	NaN	NaN	0.0221	0	Q6DFV5	Probable helicase with zinc finger domain	Helz	mmu:78455			
4252	0.0222	NaN	NaN	0.0222	0	Q8BLB7	Lethal(3)malignant brain tumor-like protein 3	L3mbtl3	mmu:237339			
4253	-0.0089	0.0533	NaN	0.0222	0	Q9D0M5	Dynein light chain 2, cytoplasmic	Dynll2	mmu:68097			
4254	0.8109	NaN	NaN	0.0227	0	P97360	Transcription factor ETV6	Etv6	mmu:14011			
4255	NaN	0.2472	-0.4149	0.0227	0	Q9QYB1	Chloride intracellular channel protein 4	Clic4	mmu:29876			
4256	NaN	0.4916	-0.4428	0.0244	0	P10518	Delta-aminolevulinic acid dehydratase	Alad	mmu:17025	1		
4257	1.0010	-0.9515	NaN	0.0248	0	P97432	Next to BRCA1 gene 1 protein	Nbr1	mmu:17966			
4258	NaN	-0.0642	0.1139	0.0249	0	Q6NZB0	DnaJ homolog subfamily C member 8	Dnajc8	mmu:68598			
4259	NaN	0.0251	NaN	0.0251	0	Q60773	Cyclin-dependent kinase 4 inhibitor D	Cdkn2d	mmu:12581			
4260	-0.2524	0.3044	NaN	0.0260	0	Q9EST3	Eukaryotic translation initiation factor 4E transporter	Eif4enif1	mmu:74203			
4261	0.5970	NaN	NaN	0.0272	0	Q9ESC8	AF4/FMR2 family member 4	Aff4	mmu:93736			
4262	NaN	0.3508	-0.2944	0.0282	0	Q8BHI7	Elongation of very long chain fatty acids protein;Elongation of very long chain fatty acids protein 5	Elovl5	mmu:68801			
4263	0.0917	NaN	-0.0321	0.0298	0	Q80UQ2	Ras association domain-containing protein 6	Rassf6	mmu:73246			
4264	0.3206	NaN	0.3133	0.0312	0	P70340	Mothers against decapentaplegic homolog;Mothers against decapentaplegic homolog 1;Mothers a	Smad1;Sm	mmu:17125			
4265	0.0316	NaN	NaN	0.0316	0	Q9D084	Centromere protein S	Apitd1	mmu:69928			
4266	-0.0341	NaN	0.1006	0.0332	0	Q8R2U6	Diphosphoinositol polyphosphate phosphohydrolase 2	Nudt4	mmu:71207			
4267	0.0340	NaN	NaN	0.0340	0	Q3URS9	Coiled-coil domain-containing protein 51	Ccdc51	mmu:66658			
4268	0.3453	NaN	-0.3047	0.0364	0	Q8CES0	N-alpha-acetyltransferase 30	Naa30	mmu:70646			
4269	0.0071	NaN	0.0670	0.0371	0	Q9R1R2	Tripartite motif-containing protein 3	Trim3	mmu:55992			
4270	NaN	0.0373	NaN	0.0373	0	Q8BMK0	Centrosomal protein of 85 kDa	Cep85	mmu:70012			
4271	NaN	0.0385	NaN	0.0385	0	Q3UHK6	Teneurin-4;Teneurin-3;Teneurin-1;Ten-1 intracellular domain;Teneurin C-terminal-associated pep	Tenm4;Ter	mmu:23966			
4272	-0.1041	0.1835	NaN	0.0397	0	Q9D8T4	Golgi apparatus membrane protein TVP23 homolog B	Tvp23b	mmu:67510			
4273	0.0413	NaN	NaN	0.0413	0	Q9WU62	Inner centromere protein	Incenp	mmu:16319			

4274	NaN	0.4343	-1.2029	0.0420	0	Q8QZT1	Acetyl-CoA acetyltransferase, mitochondrial	Acat1	mmu:110446				
4275	NaN	0.0802	0.0069	0.0435	0	Q99JH8	ER lumen protein-retaining receptor 1	Kdelr1	mmu:68137				
4276	0.0671	NaN	NaN	0.0464	0	O88286	Protein Wiz	Wiz	mmu:22404				
4277	NaN	0.0394	0.0541	0.0467	0	Q8C1Z8	tRNA methyltransferase 10 homolog A	Trmt10a	mmu:108943				
4278	NaN	0.0488	NaN	0.0488	0	P97819	85/88 kDa calcium-independent phospholipase A2	Pla2g6	mmu:53357				
4279	0.0888	NaN	0.0108	0.0498	0	Q9Z2C4	Myotubularin-related protein 1	Mtmr1	mmu:53332		1		
4280	0.0162	0.0837	NaN	0.0500	0	Q80SY4	E3 ubiquitin-protein ligase MIB1	Mib1	mmu:225164			1	
4281	NaN	0.3360	-0.2319	0.0521	0	P99029	Peroxiredoxin-5, mitochondrial	Prdx5	mmu:54683	1			
4282	NaN	0.0530	0.0512	0.0521	0	Q9QYY0	GRB2-associated-binding protein 1	Gab1	mmu:14388	1			
4283	0.0524	NaN	NaN	0.0524	0	Q9DBF7	Pre-mRNA-splicing factor CWC25 homolog	Cwc25	mmu:67480				
4284	NaN	0.7546	-0.6460	0.0543	0	Q9ER81	Torsin-1A-interacting protein 2, isoform IFRG15	Tor1aip2	mmu:240832				
4285	-0.6167	0.7291	NaN	0.0562	0	Q640M1	U3 small nucleolar RNA-associated protein 14 homolog A	Utp14a	mmu:72554				
4286	NaN	0.0998	-0.8512	0.0563	0	P69566	Ran-binding protein 9	Ranbp9	mmu:56705				
4287	NaN	-0.4153	0.5297	0.0572	0	Q5S555		Ehbp1					
4288	NaN	0.0581	NaN	0.0581	0	Q9CXY9	GPI-anchor transamidase	Pigk	mmu:329777				
4289	0.0581	NaN	NaN	0.0581	0	P24860	G2/mitotic-specific cyclin-B1	Ccnb1	mmu:268697				
4290	NaN	NaN	0.0591	0.0591	0	Q61879	Myosin-10	Myh10	mmu:77579				
4291	0.0402	NaN	NaN	0.0599	0	Q3U1T3	Breast cancer metastasis-suppressor 1-like protein	Brms1l	mmu:52592				
4292	NaN	-0.6120	0.7357	0.0619	0	O08709	Peroxiredoxin-6	Prdx6	mmu:11758	1			
4293	0.3088	0.0336	NaN	0.0621	0	Q9CQK7	RWD domain-containing protein 1	Rwdd1	mmu:66521	1			
4294	-0.6096	NaN	0.7348	0.0626	0	Q5SUE8	Ankyrin repeat domain-containing protein 40	Ankrd40	mmu:71452				
4295	0.1464	NaN	0.8053	0.0632	0	E9Q723		Ttc13					
4296	-0.4566	NaN	NaN	0.0653	0	P41230	Lysine-specific demethylase 5C	Kdm5c	mmu:20591				
4297	NaN	0.8968	-0.8082	0.0670	0	Q5HZH2	Ribosome biogenesis protein TSR3 homolog	Tsr3	mmu:68327				
4298	-0.1846	NaN	NaN	0.0679	0	Q99N92	39S ribosomal protein L27, mitochondrial	Mrpl27	mmu:94064				
4299	NaN	-0.6272	0.7636	0.0682	0	Q8CF89	TGF-beta-activated kinase 1 and MAP3K7-binding protein 1	Tab1	mmu:66513		1		
4300	0.4539	NaN	0.1110	0.0683	0	Q9D735	Uncharacterized protein C19orf43 homolog		mmu:68544				
4301	NaN	-0.0818	0.2191	0.0686	0	Q8C351	Layilin	Layn	mmu:244864				
4302	0.0689	NaN	NaN	0.0689	0	Q9ERA0	Alpha-globin transcription factor CP2;Upstream-binding protein 1	Tfcp2;Ubp	mmu:21422				
4303	NaN	0.6706	-0.5314	0.0696	0	Q8BXN9	Transmembrane protein 87A	Tmem87a	mmu:211499				
4304	0.0707	NaN	NaN	0.0707	0	Q99MK9	Ras association domain-containing protein 1	Rassf1	mmu:56289				
4305	0.0717	NaN	NaN	0.0717	0	Q9EQM6	Microprocessor complex subunit DGCR8	Dgcr8	mmu:94223				
4306	NaN	-0.1891	0.3340	0.0724	0	P70349	Histidine triad nucleotide-binding protein 1	Hint1	mmu:15254				
4307	0.4985	-0.7465	NaN	0.0747	0	Q80UK8	Integrator complex subunit 2	Ints2	mmu:70422				
4308	0.0750	NaN	NaN	0.0750	0	Q8R1Z9	RING finger protein 121	Rnf121	mmu:75212				
4309	0.0752	NaN	NaN	0.0752	0	A2AFR3	FERM and PDZ domain-containing protein 4	Frmpd4	mmu:333605				
4310	0.0777	NaN	NaN	0.0777	0	Q8BN78	Transcriptional regulator Kaiso	Zbtb33	mmu:56805				
4311	0.0796	NaN	NaN	0.0796	0	O88196	E3 ubiquitin-protein ligase TTC3	Ttc3				1	
4312	-0.0494	NaN	-0.6518	0.0811	0	Q9D9I4	TBC1 domain family member 20	Tbc1d20	mmu:67231				
4313	0.0813	NaN	NaN	0.0813	0	Q9CQ69	Cytochrome b-c1 complex subunit 8	Uqcrq	mmu:22272				
4314	NaN	-0.2950	-1.0647	0.0813	0	O35083	1-acyl-sn-glycerol-3-phosphate acyltransferase alpha;1-acyl-sn-glycerol-3-phosphate acyltransferas	Agpat1	mmu:55979				
4315	NaN	0.0823	NaN	0.0823	0	Q9QXM1	Junction-mediating and -regulatory protein	Jmy	mmu:57748				1
4316	-0.3320	-0.3260	NaN	0.0824	0	Q80YR5	Scaffold attachment factor B2	Safb2	mmu:224902				
4317	NaN	0.0900	0.0772	0.0836	0	Q9JII6	Alcohol dehydrogenase [NADP(+)]	Akr1a1	mmu:58810				
4318	-0.2464	-0.0145	NaN	0.0843	0	Q9WUJ8	Origin recognition complex subunit 6	Orc6					
4319	NaN	NaN	NaN	0.0848	0	Q9Z175	Lysyl oxidase homolog 3	Loxl3	mmu:16950				
4320	NaN	-0.2360	0.4062	0.0851	0	Q9R0P3	S-formylglutathione hydrolase	Esd	mmu:13885				
4321	0.4391	NaN	-0.2679	0.0856	0	A2AJI0	MAP7 domain-containing protein 1	Map7d1	mmu:245877				
4322	0.0858	NaN	NaN	0.0858	0	P53762	Aryl hydrocarbon receptor nuclear translocator	Arnt	mmu:11863	1			
4323	0.4090	NaN	NaN	0.0860	0	Q99P69	Kinetochore protein Nuf2	Nuf2	mmu:66977				
4324	NaN	0.0113	0.1658	0.0886	0	O88848	ADP-ribosylation factor-like protein 6	Arl6	mmu:56297				
4325	NaN	NaN	0.0893	0.0893	0	A2APB8	Targeting protein for Xklp2	Tpx2	mmu:72119				
4326	NaN	0.1080	0.0708	0.0894	0	Q9D0S9	Histidine triad nucleotide-binding protein 2, mitochondrial	Hint2	mmu:68917				
4327	0.1200	NaN	0.2207	0.0900	0	Q9R207	Nibrin	Nbn	mmu:27354				
4328	0.0915	NaN	NaN	0.0915	0	Q5SQY2	Biorientation of chromosomes in cell division protein 1	Bod1	mmu:69556				
4329	0.0916	NaN	NaN	0.0916	0	Q3UHD2	Glucose-fructose oxidoreductase domain-containing protein 1	Gfod1	mmu:328232				
4330	-0.0673	0.2529	NaN	0.0928	0	Q3TBW2	39S ribosomal protein L10, mitochondrial	Mrpl10	mmu:107732				
4331	NaN	0.3014	-0.1153	0.0930	0	Q69ZJ7	RAB6A-GEF complex partner protein 1	Ric1					
4332	0.0962	NaN	NaN	0.0962	0	O88508	DNA (cytosine-5)-methyltransferase 3A	Dnmt3a	mmu:13435				
4333	0.4276	NaN	-0.2322	0.0977	0	Q5ND52	rRNA methyltransferase 3, mitochondrial	Rnmtl1	mmu:67390				
4334	NaN	0.1763	0.0203	0.0983	0	Q8VEB2	Protein salvador homolog 1	Sav1	mmu:64010				
4335	NaN	0.0984	NaN	0.0984	0	Q9R1P1	Proteasome subunit beta type-3	Psmb3	mmu:26446				
4336	0.0986	NaN	NaN	0.0986	0	P61294	Ras-related protein Rab-6B	Rab6b	mmu:270192				
4337	NaN	1.0216	-0.3028	0.0987	0	Q9D2N4	Dystrobrevin;Dystrobrevin alpha	Dtna	mmu:13527				
4338	NaN	NaN	0.0987	0.0987	0	A5A4Y9		Ppp1r11	mmu:76497				
4339	0.0991	NaN	NaN	0.0991	0	Q4U2R1	E3 ubiquitin-protein ligase HERC2	Herc2	mmu:15204			1	
4340	NaN	NaN	0.1011	0.1011	0	Q91ZZ2		Rapgef1					
4341	0.1036	NaN	NaN	0.1036	0	O35144	Telomeric repeat-binding factor 2	Terf2	mmu:21750				
4342	0.1058	NaN	NaN	0.1058	0	Q8VDY4	EF-hand calcium-binding domain-containing protein 7	Efcab7	mmu:230500				
4343	0.1059	NaN	NaN	0.1059	0	Q9CQQ0	Small integral membrane protein 8	Smim8	mmu:66291				
4344	NaN	0.2177	0.0028	0.1067	0	Q99MR8	Methylcrotonoyl-CoA carboxylase subunit alpha, mitochondrial	Mccc1	mmu:72039				
4345	0.1077	NaN	NaN	0.1077	0	Q80YT7	Myomegalin	Pde4dip	mmu:83679				
4346	NaN	0.1091	NaN	0.1091	0	Q9D2V7	Coronin-7	Coro7	mmu:78885				
4347	NaN	1.2214	1.7391	0.1102	0	P17095	High mobility group protein HMG-I/HMG-Y	Hmga1	mmu:15361				
4348	NaN	NaN	0.1103	0.1103	0	P05531	X-linked lymphocyte-regulated protein PM1	Xlr;383040	mmu:22441				
4349	NaN	-0.0940	0.3150	0.1105	0	Q9R059	Four and a half LIM domains protein 3	Fhl3	mmu:14201				
4350	0.1113	NaN	NaN	0.1113	0	Q9JJC9	Homocysteine-responsive endoplasmic reticulum-resident ubiquitin-like domain member 2 protei	Herpud2	mmu:80517				
4351	NaN	NaN	0.1138	0.1138	0	Q9QXS1	Plectin	Plec	mmu:18810				
4352	NaN	0.0318	0.2018	0.1168	0	Q61584	Fragile X mental retardation syndrome-related protein 1	Fxr1	mmu:14359				
4353	0.1759	-0.7356	NaN	0.1192	0	P61961	Ubiquitin-fold modifier 1	Ufm1	mmu:67890				
4354	NaN	-0.1056	0.3462	0.1203	0	Q8C0Y0	Serine/threonine-protein phosphatase 4 regulatory subunit 4	Ppp4r4	mmu:74521				
4355	NaN	0.9018	-0.6578	0.1220	0	E9Q9C3		Mllt4					
4356	NaN	-0.1356	0.3807	0.1226	0	Q9D0I4	Syntaxin-17	Stx17	mmu:67727				
4357	0.1236	NaN	NaN	0.1236	0	E9QAP7		Taf4a	mmu:228980				
4358	0.1245	NaN	NaN	0.1245	0	Q8BGZ4	Cell division cycle protein 23 homolog	Cdc23	mmu:52563			1	
4359	0.8978	-0.6482	NaN	0.1248	0	Q99PN3	Tripartite motif-containing protein 26	Trim26	mmu:22670				
4360	NaN	0.3788	-0.1270	0.1259	0	Q8K094		Pvr	mmu:52118				
4361	-0.2540	NaN	0.3600	0.1278	0	Q8BZS9	Putative pre-mRNA-splicing factor ATP-dependent RNA helicase DHX32	Dhx32	mmu:101437				
4362	0.0308	NaN	NaN	0.1291	0	Q8BHZ4	Zinc finger protein 592	Znf592	mmu:233410				
4363	0.1297	NaN	NaN	0.1297	0	Q6RT24	Kinesin-like protein;Centromere-associated protein E	Cenpe	mmu:229841				
4364	-5.0509	NaN	NaN	0.1313	0	Q9DA08	SAGA-associated factor 29 homolog	Ccdc101	mmu:75565				
4365	NaN	-0.0665	-0.1628	0.1327	0	P26040	Ezrin	Ezr	mmu:22350				
4366	0.1351	NaN	NaN	0.1351	0	Q8C0S1	DIS3-like exonuclease 1	Dis3l	mmu:213550				
4367	NaN	0.6759	-0.3993	0.1383	0	Q9QXM0	Abhydrolase domain-containing protein 2	Abhd2	mmu:54608				
4368	0.1393	NaN	NaN	0.1393	0	Q9WV30	Nuclear factor of activated T-cells 5	Nfat5	mmu:54446				
4369	NaN	-0.1026	0.3813	0.1394	0	P45952	Medium-chain specific acyl-CoA dehydrogenase, mitochondrial	Acadm	mmu:11364				
4370	NaN	0.1533	0.4692	0.1409	0	Q9R008	Mevalonate kinase	Mvk	mmu:17855				
4371	NaN	0.0663	0.2167	0.1415	0	O88427	Voltage-dependent T-type calcium channel subunit alpha-1H	Cacna1h					
4372	NaN	NaN	-0.0870	0.1415	0	Q9CWP6	Motile sperm domain-containing protein 2	Mospd2	mmu:76763				
4373	0.1432	NaN	NaN	0.1432	0	F6R0S9		Plec					
4374	0.1257	0.5869	NaN	0.1433	0	Q99ME2	WD repeat-containing protein 6	Wdr6	mmu:83669				
4375	0.1438	NaN	NaN	0.1438	0	F6XLV1		E030010N(	mmu:381284				
4376	0.2241	NaN	0.0645	0.1443	0	Q9CQT2	RNA-binding protein 7	Rbm7	mmu:67010				
4377	0.1444	NaN	NaN	0.1444	0	Q80Z37	E3 ubiquitin-protein ligase Topors	Topors	mmu:106021				
4378	-0.3803	NaN	0.1859	0.1445	0	Q9Z0F8	Disintegrin and metalloproteinase domain-containing protein 17	Adam17	mmu:11491				
4379	NaN	0.1450	NaN	0.1450	0	Q8BLR9	Hypoxia-inducible factor 1-alpha inhibitor	Hif1an	mmu:319594				
4380	0.1457	NaN	NaN	0.1457	0	Q8R3L2	Transcription factor 25	Tcf25	mmu:66855				
4381	-0.0502	NaN	0.5415	0.1496	0	Q8VC03	Echinoderm microtubule-associated protein-like 3	Eml3	mmu:225898				
4382	0.1497	NaN	NaN	0.1497	0	Q5U431	G-protein coupled receptor 39	Gpr39					
4383	0.1505	NaN	NaN	0.1505	0	Q31093		H2-M3;H2-	mmu:14991				
4384	0.0616	NaN	-0.0472	0.1520	0	Q8BP71	RNA binding protein fox-1 homolog 2	Rbfox2	mmu:93686				
4385	0.1526	NaN	NaN	0.1526	0	Q5RJG1	Nucleolar protein 10	Nol10	mmu:217431				
4386	0.6360	NaN	-0.0579	0.1532	0	Q9JIZ5		Tfap4	mmu:83383				
4387	NaN	0.2098	0.3869	0.1548	0	Q9D1R9	60S ribosomal protein L34	Rpl34	mmu:68436				
4388	NaN	0.7221	0.2614	0.1554	0	Q9D892	Inosine triphosphate pyrophosphatase	Itpa	mmu:16434				
4389	0.9551	NaN	NaN	0.1557	0	Q6P9R1	ATP-dependent RNA helicase DDX51	Ddx51	mmu:69663				
4390	0.3870	NaN	NaN	0.1563	0	Q9CQ20	Mid1-interacting protein 1	Mid1ip1	mmu:68041				
4391	0.1605	NaN	NaN	0.1605	0	Q9ERH4	Nucleolar and spindle-associated protein 1	Nusap1	mmu:108907				
4392	0.1607	NaN	NaN	0.1607	0	Q6ZQF7	Protein Jade-2	Jade2	mmu:76901				
4393	NaN	0.5479	-0.2246	0.1617	0	Q8C3Q9	Caspase;Caspase-9;Caspase-9 subunit p35;Caspase-9 subunit p10	Casp9	mmu:12371				1
4394	NaN	0.1278	0.4939	0.1660	0	Q3TQQ9-3	Uncharacterized protein C1orf112 homolog						
4395	-0.5753	-0.2076	NaN	0.1666	0	Q61586	Glycerol-3-phosphate acyltransferase 1, mitochondrial	Gpam	mmu:14732				
4396	NaN	0.2769	-0.2145	0.1672	0	Q3UZW7	Protein-lysine N-methyltransferase EEF2KMT	Eef2kmt	mmu:70511				
4397	NaN	-0.2251	-0.2289	0.1675	0	Q9R0P6	Signal peptidase complex catalytic subunit SEC11;Signal peptidase complex catalytic subunit SEC1	Sec11a	mmu:56529				
4398	NaN	-0.2751	0.6124	0.1687	0	Q9QZI8	Serine incorporator 1	Serinc1	mmu:56442				
4399	0.1691	NaN	NaN	0.1691	0	Q9D8N6	Protein lin-37 homolog	Lin37	mmu:75660				

4400	[illegible]	NaN	0.5453	0.1691	0	P97863	Nuclear factor 1;Nuclear factor 1 B-type	Nfib	mmu:18028		
4401	0.1700	NaN	NaN	0.1700	0	A0A0U1RNZ3		4931406P16Rik			
4402	0.2363	0.3235	NaN	0.1702	0	Q8VI63	MOB kinase activator 2	Mob2	mmu:101513		
4403	-0.1423	0.4853	NaN	0.1715	0	Q9D868	Peptidyl-prolyl cis-trans isomerase;Peptidyl-prolyl cis-trans isomerase H	Ppih	mmu:66101		
4404	0.1719	NaN	NaN	0.1719	0	Q6A058	Armadillo repeat-containing X-linked protein 2	Armcx2	mmu:67416		
4405	0.2975	0.0473	NaN	0.1724	0	Q99MP8	BRCA1-associated protein	Brap	mmu:72399		1
4406	NaN	0.1724	NaN	0.1724	0	O35350	Calpain-1 catalytic subunit	Capn1	mmu:12333		
4407	NaN	0.1952	0.1511	0.1732	0	Q9WV91	Prostaglandin F2 receptor negative regulator	Ptgfrn	mmu:19221		
4408	0.1528	0.1936	NaN	0.1732	0	Q9JI78	Peptide-N(4)-(N-acetyl-beta-glucosaminyl)asparagine amidase	Ngly1	mmu:59007		
4409	0.1773	NaN	NaN	0.1773	0	A2AKX3	Probable helicase senataxin	Setx	mmu:269254	1	
4410	-0.3708	NaN	0.7268	0.1780	0	Q8BSF4	Phosphatidylserine decarboxylase proenzyme;Phosphatidylserine decarboxylase alpha chain;Phos	Pisd;Gm20	mmu:320951		
4411	0.6459	-0.7268	NaN	0.1787	0	Q60960	Importin subunit alpha-5;Importin subunit alpha-5, N-terminally processed	Kpna1	mmu:16646		
4412	0.1788	NaN	NaN	0.1788	0	Q80Y83	Dixin	Dixdc1	mmu:330938		
4413	NaN	0.1354	0.2223	0.1789	0	Q91WD1	DNA-directed RNA polymerase III subunit RPC4	Polr3d	mmu:67065		
4414	NaN	0.4474	-0.0884	0.1795	0	Q8VHL1	Histone-lysine N-methyltransferase SETD7	Setd7	mmu:73251		
4415	0.1799	NaN	NaN	0.1799	0	P60824	Cold-inducible RNA-binding protein	Cirbp	mmu:12696		
4416	NaN	NaN	0.1801	0.1801	0	Q9CQX6		Gm16286			
4417	-0.4481	1.3981	NaN	0.1802	0	Q8BQX5	Transmembrane and coiled-coil domain-containing protein 6	Tmco6	mmu:71983		
4418	0.1810	NaN	NaN	0.1810	0	Q8C167	Prolyl endopeptidase-like	Prepl	mmu:213760		
4419	0.1816	NaN	NaN	0.1816	0	Q8BYK4	Retinol dehydrogenase 12	Rdh12	mmu:77974		
4420	-0.6786	NaN	1.8724	0.1820	0	Q58FA4	Transcription factor E2F8	E2f8	mmu:108961		
4421	NaN	0.4103	-0.0423	0.1840	0	Q9CZA6	Nuclear distribution protein nudE homolog 1	Nde1	mmu:67203		
4422	0.1854	NaN	NaN	0.1854	0	A8Y5G5		Zfyve9			
4423	NaN	0.3437	0.0338	0.1888	0	Q9ESJ4	NCK-interacting protein with SH3 domain	Nckipsd	mmu:80987		
4424	0.3366	NaN	NaN	0.1901	0	P33215	Protein NEDD1	Nedd1	mmu:17997		
4425	0.1901	NaN	NaN	0.1901	0	O35594	Intraflagellar transport protein 81 homolog	Ift81	mmu:12589		
4426	NaN	0.2880	0.0936	0.1908	0	Q5NCQ5	Diphthamide biosynthesis protein 1	Dph1	mmu:116905		
4427	NaN	0.1940	NaN	0.1940	0	Q91V09	WD repeat-containing protein 13	Wdr13	mmu:73447		
4428	0.1949	NaN	NaN	0.1949	0	G3XA66		Mtx1			
4429	0.1965	NaN	NaN	0.1965	0	Q8C7V8	Coiled-coil domain-containing protein 134	Ccdc134	mmu:76457		
4430	0.3300	NaN	NaN	0.1971	0	Q8CFE2	UPF0609 protein C4orf27 homolog	2700029M	mmu:72612		
4431	NaN	0.1974	NaN	0.1974	0	Q9JIZ9	Phospholipid scramblase 3	Plscr3	mmu:70310		
4432	NaN	0.8227	NaN	0.1982	0	Q6KAQ7	ZZ-type zinc finger-containing protein 3	Zzz3	mmu:108946		
4433	NaN	NaN	NaN	0.1985	0	Q9D0L7	Armadillo repeat-containing protein 10	Armc10	mmu:67211		
4434	0.2876	0.1095	NaN	0.1986	0	P63082	V-type proton ATPase 16 kDa proteolipid subunit	Atp6v0c	mmu:11984		
4435	NaN	0.1429	0.2566	0.1997	0	Q923S9	Ras-related protein Rab-30	Rab30	mmu:75985		
4436	NaN	0.2016	NaN	0.2016	0	Q8R4Z4	ETS translocation variant 3	Etv3	mmu:27049		
4437	-1.1237	0.7723	NaN	0.2019	0	Q8VEG4	Exonuclease 3-5 domain-containing protein 2	Exd2	mmu:97827		
4438	NaN	0.4076	[illegible]	0.2026	0	Q6PB51	Coiled-coil domain-containing protein 117	Ccdc117	mmu:104479		
4439	0.2039	NaN	NaN	0.2039	0	Q9WUQ2	Prolactin regulatory element-binding protein	Preb	mmu:50907		
4440	NaN	1.3178	0.7718	0.2054	0	Q9R0Y5	Adenylate kinase isoenzyme 1	Ak1	mmu:11636		
4441	0.6612	NaN	NaN	0.2055	0	Q8BSZ2	AP-3 complex subunit sigma-2	Ap3s2	mmu:11778		
4442	NaN	0.3818	0.0295	0.2057	0	P62488	DNA-directed RNA polymerase II subunit RPB7	Polr2g	mmu:67710		
4443	-0.9990	-0.2322	NaN	0.2057	0	Q8R418	Endoribonuclease Dicer	Dicer1			
4444	NaN	0.4631	-0.0515	0.2058	0	Q921H9	Cytochrome c oxidase assembly factor 7	Coa7	mmu:69893		
4445	-0.2867	NaN	-0.8547	0.2099	0	Q9CRB2	H/ACA ribonucleoprotein complex subunit 2	Nhp2	mmu:52530		
4446	NaN	0.9415	-0.5213	0.2101	0	Q5XKN4	Protein jagunal homolog 1	Jagn1	mmu:67767		
4447	0.2135	NaN	NaN	0.2135	0	Q920Q6	RNA-binding protein Musashi homolog 2	Msi2	mmu:76626		
4448	NaN	0.3770	0.0506	0.2138	0	Q9EPL4	Methyltransferase-like protein 9	Mettl9	mmu:59052		
4449	NaN	0.4367	[illegible]	0.2154	0	Q5SRY7	F-box/WD repeat-containing protein 11	Fbxw11	mmu:103583		
4450	NaN	0.2159	NaN	0.2159	0	Q9D924	Iron-sulfur cluster assembly 1 homolog, mitochondrial	AK157302;	mmu:69046		
4451	-0.3578	NaN	-0.1903	0.2167	0	Q8K305	Kinetochore-associated protein NSL1 homolog	Nsl1			
4452	0.1202	NaN	NaN	0.2201	0	Q8BT14	CCR4-NOT transcription complex subunit 4	Cnot4	mmu:53621		1
4453	NaN	[illegible]	0.4392	0.2205	0	P58058	NAD kinase	Nadk	mmu:192185		
4454	NaN	-0.4383	0.8793	0.2205	0	Q8C0J2	Autophagy-related protein 16-1	Atg16l1	mmu:77040		
4455	0.2232	NaN	NaN	0.2232	0	Q9D2D7	Zinc finger protein 687	Znf687	mmu:78266		
4456	NaN	0.1360	0.3119	0.2239	0	Q61206	Platelet-activating factor acetylhydrolase IB subunit beta	Pafah1b2	mmu:18475		
4457	0.2247	NaN	NaN	0.2247	0	Q8R4Y8	Rotatin	Rttn	mmu:246102		
4458	NaN	0.1501	0.3002	0.2252	0	Q5U5M8	Biogenesis of lysosome-related organelles complex 1 subunit 3	Bloc1s3	mmu:232946		
4459	NaN	-0.1192	0.5520	0.2257	0	Q8BX94	Oxysterol-binding protein-related protein 2	Osbpl2	mmu:228983		
4460	NaN	0.3667	-0.1131	0.2265	0	Q80UP8	Sodium-dependent phosphate transporter 2	Slc20a2	mmu:20516		
4461	-0.0635	0.5226	NaN	0.2296	0	Q9CQJ7	Securin	Pttg1	mmu:30939		
4462	0.2299	NaN	NaN	0.2299	0	Q9DAZ9	Abscission/NoCut checkpoint regulator	Zfyve19	mmu:72008		
4463	NaN	NaN	0.2303	0.2303	0	Q5RL51	Glutathione S-transferase C-terminal domain-containing protein	Gstcd	mmu:67553		
4464	0.2327	NaN	NaN	0.2327	0	O55102	Biogenesis of lysosome-related organelles complex 1 subunit 1	Bloc1s1	mmu:14533		
4465	NaN	1.4020	-0.3728	0.2335	0	Q9CRA5	Golgi phosphoprotein 3	Golph3	mmu:66629		
4466	NaN	NaN	0.2338	0.2338	0	P61406	Telomerase-binding protein EST1A	Smg6	mmu:103677		
4467	NaN	-0.1620	-0.3033	0.2345	0	Q9WUP7	Ubiquitin carboxyl-terminal hydrolase isozyme L5	Uchl5	mmu:56207		
4468	NaN	0.0204	0.4492	0.2348	0	Q6NXN1	SUZ domain-containing protein 1	Szrd1	mmu:213491		
4469	0.2359	NaN	NaN	0.2359	0	B1AVY7	Kinesin-like protein KIF16B	Kif16b	mmu:16558		
4470	-0.1393	NaN	NaN	0.2359	0	Q9D0F1	Kinetochore protein NDC80 homolog	Ndc80	mmu:67052		
4471	NaN	0.0872	0.5805	0.2367	0	Q8BJF9	Charged multivesicular body protein 2b	Chmp2b	mmu:68942		
4472	NaN	0.3276	0.1489	0.2382	0	Q9D0R9	WD repeat-containing protein 89	Wdr89	mmu:72338		
4473	NaN	NaN	NaN	0.2404	0	P58064	28S ribosomal protein S6, mitochondrial	Mrps6	mmu:121022		
4474	NaN	0.7285	0.0523	0.2409	0	Q9DCD0	6-phosphogluconate dehydrogenase, decarboxylating	Pgd	mmu:110208		
4475	0.3269	NaN	NaN	0.2433	0	Q9CPQ3	Mitochondrial import receptor subunit TOM22 homolog	Tomm22	mmu:223696		
4476	-0.5504	-1.0173	NaN	0.2434	0	Q8CIM8	Integrator complex subunit 4	Ints4	mmu:101861		
4477	0.1726	0.2208	NaN	0.2441	0	O70480	Vesicle-associated membrane protein 4	Vamp4			
4478	NaN	0.0896	0.4047	0.2472	0	Q5EBG6	Heat shock protein beta-6	Hspb6	mmu:243912		
4479	0.2475	NaN	NaN	0.2475	0	Q80UV9	Transcription initiation factor TFIID subunit 1	Taf1	mmu:270627		
4480	0.2482	NaN	NaN	0.2482	0	Q99LQ1	MAP3K12-binding inhibitory protein 1	Mbip	mmu:217588		
4481	NaN	NaN	0.8308	0.2524	0	P97348	Rho-related GTP-binding protein RhoD	Rhod	mmu:11854		
4482	0.2525	NaN	NaN	0.2525	0	Q8JZN7	Mitochondrial Rho GTPase 2	Rhot2	mmu:214952		
4483	NaN	1.1081	0.7438	0.2545	0	Q62446	Peptidyl-prolyl cis-trans isomerase FKBP3	Fkbp3	mmu:30795		
4484	0.3067	0.2044	NaN	0.2551	0	Q9DBV4	Matrix-remodeling-associated protein 8	Mxra8	mmu:74761		
4485	NaN	-0.6292	-0.0383	0.2566	0	Q9CY73	39S ribosomal protein L44, mitochondrial	Mrpl44	mmu:69163		
4486	NaN	0.3002	0.2145	0.2574	0	Q9CPT5	Nucleolar protein 16	Nop16	mmu:28126		
4487	NaN	0.9115	-0.3957	0.2579	0	O09117	Synaptophysin-like protein 1	Sypl1	mmu:19027		
4488	NaN	0.2645	NaN	0.2645	0	Q9JHP7	KDEL motif-containing protein 1	Kdelc1	mmu:72050		
4489	NaN	0.0161	0.5150	0.2656	0	O89109	Intermediate conductance calcium-activated potassium channel protein 4	Kcnn4	mmu:16534		
4490	0.2735	NaN	NaN	0.2735	0	Q99LJ5	CKLF-like MARVEL transmembrane domain-containing protein 3	Cmtm3	mmu:68119		
4491	NaN	NaN	0.2753	0.2753	0	O09000	Nuclear receptor coactivator 3	Ncoa3			
4492	NaN	-0.3148	NaN	0.2755	0	Q61468	Mesothelin;Megakaryocyte-potentiating factor;Mesothelin, cleaved form	Msln	mmu:56047		
4493	-0.1388	NaN	NaN	0.2760	0	Q8BIW9	Chromosome transmission fidelity protein 18 homolog	Chtf18	mmu:214901		
4494	NaN	-0.1699	0.7259	0.2780	0	P70245	3-beta-hydroxysteroid-Delta(8),Delta(7)-isomerase	Ebp	mmu:13595		
4495	NaN	1.0247	-0.6830	0.2788	0	Q8BYR1	tRNA wybutosine-synthesizing protein 4	Lcmt2	mmu:329504		
4496	0.2800	NaN	NaN	0.2800	0	Q8CFK2	Transcription factor IIIB 90 kDa subunit	Brf1			
4497	NaN	0.8959	-0.3344	0.2808	0	Q9CQE7	Endoplasmic reticulum-Golgi intermediate compartment protein 3	Ergic3	mmu:66366		
4498	0.2833	NaN	NaN	0.2833	0	Q6NXH3	Vasculin	Gpbp1	mmu:73274		
4499	0.6362	NaN	NaN	0.2897	0	Q8BHL3	TBC1 domain family member 10B	Tbc1d10b	mmu:68449		
4500	0.6525	-0.3891	NaN	0.2899	0	Q6DIC0	Probable global transcription activator SNF2L2	Smarca2	mmu:67155		
4501	0.2925	NaN	NaN	0.2925	0	Q91YN1	Protein FAM118A	Fam118a	mmu:73225		
4502	1.1692	NaN	0.8942	0.2927	0	P59326	YTH domain-containing family protein 1	Ythdf1	mmu:228994		
4503	0.5256	-0.3562	NaN	0.2932	0	Q9DBR0	A-kinase anchor protein 8	Akap8	mmu:56399		
4504	NaN	0.2948	NaN	0.2948	0	Q9D4V0	Ethanolamine kinase 1	Etnk1	mmu:75320		
4505	0.0387	NaN	NaN	0.2959	0	P70399	Tumor suppressor p53-binding protein 1	Trp53bp1;	mmu:27223		
4506	0.2973	NaN	NaN	0.2973	0	P49117	Nuclear receptor subfamily 2 group C member 2	Nr2c2	mmu:22026		
4507	0.1403	NaN	0.6357	0.2986	0	Q3URQ0	Testis-expressed sequence 10 protein	Tex10	mmu:269536		
4508	0.2253	NaN	NaN	0.2989	0	Q8BSP2	Condensin-2 complex subunit H2	Ncaph2	mmu:52683		
4509	0.2991	NaN	NaN	0.2991	0	Q91VL8	Telomeric repeat-binding factor 2-interacting protein 1	Terf2ip	mmu:57321		
4510	NaN	NaN	0.3001	0.3001	0	O88630	Golgi SNAP receptor complex member 1	Gosr1	mmu:53334		
4511	0.3016	NaN	NaN	0.3016	0	Q8CHI8	E1A-binding protein p400	Ep400	mmu:75560		
4512	0.4007	NaN	0.2065	0.3036	0	Q9JM93	ADP-ribosylation factor-like protein 6-interacting protein 4	Arl6ip4	mmu:65105		
4513	0.3050	NaN	NaN	0.3050	0	Q8R2T8	General transcription factor 3C polypeptide 5	Gtf3c5	mmu:70239		
4514	0.3053	NaN	NaN	0.3053	0	Q6P2L6	Histone-lysine N-methyltransferase;Histone-lysine N-methyltransferase NSD3	Whsc1l1	mmu:234135		
4515	0.1714	NaN	NaN	0.3056	0	Q8C092	Transcription initiation factor TFIID subunit 5	Taf5	mmu:226182		
4516	NaN	0.3068	NaN	0.3068	0	P97765	WW domain-binding protein 2	Wbp2	mmu:22378		
4517	0.3075	NaN	NaN	0.3075	0	Q9CZ91	Serum response factor-binding protein 1	Srfbp1	mmu:67222		
4518	NaN	0.2970	0.3194	0.3082	0	A0A0G2JGI9		Agl			
4519	NaN	-0.3968	1.0186	0.3109	0	Q8K363	ATP-dependent RNA helicase DDX18	Ddx18	mmu:66942		
4520	NaN	0.8322	-0.7460	0.3140	0	I7HJS5		Mpdz			
4521	0.4376	NaN	1.3909	0.3155	0	Q9ER39	Torsin-1A	Tor1a	mmu:30931	1	
4522	NaN	1.0197	-0.3870	0.3164	0	Q3ZAQ4		Atg9a	mmu:245860		
4523	-0.1168	NaN	NaN	0.3198	0	Q3UM83	Killer cell lectin-like receptor subfamily G member 2	Klrg2			
4524	NaN	0.0992	0.5486	0.3239	0	Q80Y55	BSD domain-containing protein 1	Bsdc1	mmu:100383		
4525	NaN	0.7387	-0.0858	0.3264	0	Q8C4B4	Protein unc-119 homolog B	Unc119b	mmu:106840		

4526	0.3267	NaN	NaN	0.3267	0	Q8BG17	Nucleolar protein 12	Nol12	mmu:97961				
4527	-0.8383	NaN	0.4247	0.3274	0	Q3TUH1	Phosphatidate cytidylyltransferase, mitochondrial	Tamm41					
4528	0.3283	NaN	NaN	0.3283	0	P03930	ATP synthase protein 8	Mtatp8	mmu:17706				
4529	NaN	-0.0563	0.7187	0.3312	0	P38532	Heat shock factor protein 1	Hsf1	mmu:15499	1			
4530	NaN	0.0139	0.6564	0.3352	0	Q61205	Platelet-activating factor acetylhydrolase IB subunit gamma	Pafah1b3	mmu:18476				
4531	0.3360	NaN	NaN	0.3360	0	P21460	Cystatin-C	Cst3	mmu:13010	1			
4532	0.3385	NaN	NaN	0.3385	0	Q99J10	Cytoplasmic tRNA 2-thiolation protein 1	Ctu1	mmu:233189				
4533	NaN	0.3396	NaN	0.3396	0	Q9JKT3	Taste receptor type 2 member 4	Tas2r4	mmu:57253				
4534	NaN	-0.0076	-0.0617	0.3402	0	Q8CHP5	Partner of Y14 and mago	Wibg	mmu:78428				
4535	NaN	0.5116	0.4059	0.3433	0	Q6P0X2	Zinc finger protein 511	Znf511	mmu:69752				
4536	NaN	NaN	0.3459	0.3459	0	O54714	E3 SUMO-protein ligase PIAS3	Pias3	mmu:229615				
4537	NaN	0.8734	-1.3984	0.3483	0	Q8VDG3	Poly(A)-specific ribonuclease PARN	Parn	mmu:74108				
4538	0.3501	NaN	NaN	0.3501	0	Q8VBU8	Protein BANP	Banp	mmu:53325				
4539	0.3502	NaN	NaN	0.3502	0	O88888	Amyloid beta A4 precursor protein-binding family A member 3	Apba3	mmu:57267				
4540	NaN	0.3504	NaN	0.3504	0	Q91X52	L-xylulose reductase	Dcxr	mmu:67880				
4541	0.1226	NaN	NaN	0.3505	0	P58462	Forkhead box protein P1	Foxp1	mmu:108655				
4542	0.3505	NaN	NaN	0.3505	0	Q9JKK0	Calcipressin-3	Rcan3	mmu:53902				
4543	0.1824	-0.0862	NaN	0.3508	0	Q52KR3	Protein prune homolog 2	Prune2	mmu:353211				1
4544	1.2525	NaN	-0.5496	0.3515	0	Q8VE91	Protein FAM134B	Fam134b	mmu:66270				
4545	NaN	-0.0469	0.5681	0.3529	0	P27601	Guanine nucleotide-binding protein subunit alpha-13	Gna13	mmu:14674				
4546	-0.4890	NaN	1.1980	0.3545	0	P81068	Iroquois-class homeodomain protein IRX-1	Irx1	mmu:16371				
4547	0.0596	0.6511	NaN	0.3554	0	Q62276	Mediator of RNA polymerase II transcription subunit 22	Med22	mmu:20933				
4548	-0.0177	NaN	1.6948	0.3556	0	Q61398	Procollagen C-endopeptidase enhancer 1	Pcolce	mmu:18542				
4549	NaN	NaN	0.3576	0.3576	0	Q7TSZ8	Nucleus accumbens-associated protein 1	Nacc1	mmu:66830				
4550	NaN	0.3378	0.3790	0.3584	0	Q9DBQ9	Transcriptional protein SWT1	Swt1	mmu:66875				
4551	NaN	0.8989	-0.1815	0.3587	0	Q9EPR4	Solute carrier family 23 member 2	Slc23a2	mmu:54338	1			
4552	NaN	-0.1242	0.8427	0.3592	0	Q9D2V8	Major facilitator superfamily domain-containing protein 10	Mfsd10	mmu:68294				
4553	NaN	0.0429	0.6760	0.3594	0	Q8BVA2	Transmembrane protein 222	Tmem222	mmu:52174				
4554	0.4051	NaN	0.3181	0.3616	0	Q5I043	Ubiquitin carboxyl-terminal hydrolase 28	Usp28	mmu:235323				
4555	NaN	0.2567	0.4710	0.3639	0	Q80V94	AP-4 complex subunit epsilon-1	Ap4e1	mmu:108011				
4556	NaN	0.5609	0.1681	0.3645	0	Q8VCG9	Regulatory factor X-associated protein	Rfxap	mmu:170767				
4557	NaN	NaN	0.3645	0.3645	0	Q8CA72	Gigaxonin	Gan	mmu:209239			1	
4558	NaN	0.7577	-0.0240	0.3668	0	Q6ZQI3	Malectin	Mlec	mmu:109154				
4559	[illegible]	0.0968	NaN	0.3677	0	E9Q682		Ift140					
4560	0.3693	NaN	NaN	0.3693	0	A1A5B6	TBC1 domain family member 25	Tbc1d25	mmu:209815				
4561	0.3701	NaN	NaN	0.3701	0	Q8BH48	Ubiquitin-associated protein 1	Ubap1	mmu:67123				
4562	0.3705	NaN	NaN	0.3705	0	Q6P1H6	Ankyrin repeat and LEM domain-containing protein 2	Ankle2	mmu:71782				
4563	NaN	NaN	0.3709	0.3709	0	Q9CX66	Uncharacterized protein C12orf45 homolog	D10Wsu10	mmu:28109				
4564	NaN	NaN	NaN	0.3735	0	Q8R4X3	RNA-binding protein 12	Gm28036;I	mmu:75710				
4565	-0.6659	1.1666	NaN	0.3737	0	Q9JL62	Glycolipid transfer protein	Gltp	mmu:56356				
4566	1.9761	NaN	-1.8397	0.3787	0	Q505F1	Nuclear receptor subfamily 2 group C member 1	Nr2c1	mmu:22025				
4567	NaN	2.5901	0.2341	0.3796	0	P97930	Thymidylate kinase	Dtymk	mmu:21915				
4568	0.7956	-0.0348	NaN	0.3804	0	Q69ZC8	GPALPP motifs-containing protein 1	Gpalpp1	mmu:67467				
4569	NaN	0.6508	0.1103	0.3805	0	Q3TDQ1	Dolichyl-diphosphooligosaccharide--protein glycosyltransferase subunit STT3B	Stt3b	mmu:68292				
4570	NaN	NaN	0.0656	0.3807	0	Q9CQ43		Dut	mmu:110074				
4571	NaN	NaN	0.3811	0.3811	0	E9QA15		Cald1					
4572	NaN	-0.0250	0.7889	0.3820	0	P50431	Serine hydroxymethyltransferase;Serine hydroxymethyltransferase, cytosolic	Shmt1	mmu:20425				
4573	1.5431	-0.7760	NaN	0.3836	0	A6H5Y3	Methionine synthase	Mtr	mmu:238505	1			
4574	0.2779	NaN	0.8447	0.3842	0	F7AI27	Leucine-rich repeat-containing protein 16A	Lrrc16a					
4575	NaN	0.9448	-0.1757	0.3845	0	A0A140LIW3		Frmpd3	mmu:245643				
4576	0.3853	NaN	NaN	0.3853	0	A2RSY1	KAT8 regulatory NSL complex subunit 3	Kansl3	mmu:226976				
4577	0.3873	NaN	NaN	0.3873	0	Q7TPD1	F-box only protein 11	Fbxo11	mmu:225055			1	
4578	0.1886	0.4069	NaN	0.3889	0	Q9JKY0	Cell differentiation protein RCD1 homolog	Rqcd1	mmu:58184				
4579	NaN	0.2776	0.5034	0.3905	0	Q9Z0E6	Interferon-induced guanylate-binding protein 2	Gbp2	mmu:14469				
4580	0.9061	NaN	NaN	0.3928	0	Q9JJF3	Bifunctional lysine-specific demethylase and histidyl-hydroxylase NO66	No66	mmu:71952				
4581	0.3933	NaN	NaN	0.3933	0	Q9JJZ6	Krueppel-like factor 13	Klf13	mmu:50794				
4582	NaN	NaN	0.3937	0.3937	0	Q3TSG4	RNA demethylase ALKBH5	Alkbh5	mmu:268420				
4583	NaN	NaN	0.3946	0.3946	0	Q91ZF0	DnaJ homolog subfamily C member 24	Dnajc24	mmu:99349				
4584	0.1806	0.9499	NaN	0.3978	0	Q64127	Transcription intermediary factor 1-alpha	Trim24	mmu:21848			1	
4585	NaN	0.7853	0.0108	0.3981	0	Q8CGY8	UDP-N-acetylglucosamine--peptide N-acetylglucosaminyltransferase 110 kDa subunit	Ogt	mmu:108155				
4586	NaN	0.5127	0.2835	0.3981	0	Q4PJX1	Protein odr-4 homolog	BC003331;	mmu:226499				
4587	0.4798	0.3308	NaN	0.4053	0	P25119	Tumor necrosis factor receptor superfamily member 1B	Tnfrsf1b	mmu:21938				1
4588	NaN	0.3948	0.4160	0.4054	0	P70295	Ancient ubiquitous protein 1	Aup1					
4589	0.4060	NaN	NaN	0.4060	0	Q99KK1	Receptor expression-enhancing protein 3	Reep3	mmu:28193				
4590	-0.1964	NaN	NaN	0.4116	0	Q9Z204	Heterogeneous nuclear ribonucleoproteins C1/C2	Hnrnpc	mmu:15381				
4591	NaN	1.1717	-0.3475	0.4121	0	Q5Y5T1	Probable palmitoyltransferase ZDHHC20	Zdhhc20	mmu:75965				
4592	NaN	0.4134	NaN	0.4134	0	P97473	RISC-loading complex subunit TARBP2	Tarbp2	mmu:21357				
4593	0.4146	NaN	NaN	0.4146	0	Q3UZ18	Little elongation complex subunit 2	Ice2	mmu:93697				
4594	NaN	0.8431	-0.0130	0.4151	0	Q99J56	Derlin-1	Derl1	mmu:67819				
4595	0.5553	0.8180	NaN	0.4168	0	Q6P1G0	HEAT repeat-containing protein 6	Heatr6	mmu:217026				
4596	NaN	0.1171	0.5977	0.4190	0	O88879	Apoptotic protease-activating factor 1	Apaf1	mmu:11783				1
4597	0.4202	NaN	NaN	0.4202	0	Q9CYX7	RRP15-like protein	Rrp15	mmu:67223				
4598	0.8166	NaN	NaN	0.4241	0	Q923Q2	StAR-related lipid transfer protein 13	Stard13	mmu:243362				
4599	NaN	0.4864	0.3665	0.4264	0	Q9CQL1	Protein mago nashi homolog 2	Magohb	mmu:66441				
4600	0.4281	NaN	NaN	0.4281	0	Q8BH07	ADP-ribosylation factor-like protein 6-interacting protein 6	Arl6ip6	mmu:65103				
4601	NaN	NaN	0.4283	0.4283	0	A6H6A9	Rab GTPase-activating protein 1-like	Rabgap1l	mmu:29809				
4602	NaN	1.2678	-0.4104	0.4287	0	Q3TWL2	Type 1 phosphatidylinositol 4,5-bisphosphate 4-phosphatase	Tmem55b	mmu:219024		1		
4603	0.0281	NaN	0.3942	0.4291	0	Q3V0J1	Transmembrane protein 237	Tmem237	mmu:381259				
4604	NaN	0.6004	0.2581	0.4293	0	P97823	Acyl-protein thioesterase 1	Lypla1	mmu:18777				
4605	NaN	NaN	0.4299	0.4299	0	E9Q8V6		Dennd4a	mmu:102442				
4606	0.4302	NaN	NaN	0.4302	0	E9Q5G3	Kinesin-like protein KIF23	Kif23	mmu:71819				
4607	NaN	0.0948	0.7579	0.4332	0	Q9JHR7	Insulin-degrading enzyme	Ide					
4608	1.4220	NaN	1.5710	0.4359	0	E9Q2I4		Elmsan1	mmu:238317				
4609	NaN	0.2918	0.5810	0.4364	0	O08599	Syntaxin-binding protein 1	Stxbp1	mmu:20910				
4610	0.7292	0.1455	NaN	0.4374	0	D3YUS5		Rasal2					
4611	NaN	0.5219	0.3532	0.4376	0	P83882	60S ribosomal protein L36a	Gm6525;R	mmu:19982				
4612	NaN	NaN	0.4410	0.4410	0	Q9DCE5	p21-activated protein kinase-interacting protein 1	Pak1ip1	mmu:68083				
4613	NaN	NaN	0.4436	0.4436	0	Q8BH93	MAPK-interacting and spindle-stabilizing protein-like	Mapk1ip1l	mmu:218975				
4614	1.8916	NaN	-0.3372	0.4462	0	Q66JV4	RNA-binding protein 12B-B;RNA-binding protein 12B-A	Rbm12b2;I	mmu:77604				
4615	NaN	-0.1711	1.0647	0.4468	0	Q9JKL4	NADH dehydrogenase [ubiquinone] 1 alpha subcomplex assembly factor 3	Ndufaf3	mmu:66706				
4616	0.9974	NaN	-0.1036	0.4469	0	Q8VEK0	Cell cycle control protein 50A	Tmem30a	mmu:69981				
4617	0.2510	NaN	-0.0069	0.4469	0	P52480	Pyruvate kinase PKM	Pkm	mmu:18746				
4618	NaN	0.7243	0.1703	0.4473	0	Q9CWR2	Histone-lysine N-methyltransferase SMYD3	Smyd3	mmu:69726				
4619	1.1283	NaN	NaN	0.4497	0	P41778	Pre-B-cell leukemia transcription factor 1	Pbx1	mmu:18514				
4620	0.2248	NaN	1.6329	0.4517	0	P52927	High mobility group protein HMGI-C	Hmga2	mmu:15364				
4621	0.3156	NaN	0.6025	0.4533	0	Q9DBL2	Ganglioside-induced differentiation-associated protein 2	Gdap2	mmu:14547				
4622	NaN	0.3959	0.5158	0.4559	0	Q80U56	Late secretory pathway protein AVL9 homolog	Avl9	mmu:78937				
4623	0.7406	0.1719	NaN	0.4563	0	A2A690	Protein TANC2	Tanc2	mmu:77097				
4624	0.8031	NaN	0.7424	0.4572	0	Q9DBZ1	Inhibitor of nuclear factor kappa-B kinase-interacting protein	Ikbip	mmu:67454				1
4625	NaN	0.4594	NaN	0.4594	0	Q8BH47	Vesicle-trafficking protein SEC22a	Sec22a	mmu:317717				
4626	0.4631	NaN	NaN	0.4631	0	Q60954	Homeobox protein Meis3;Homeobox protein Meis1;Homeobox protein Meis2	Meis1;Mei	mmu:17268				
4627	0.5522	NaN	0.3773	0.4648	0	Q8VDG5	Phosphopantothenate--cysteine ligase	Ppcs	mmu:106564				
4628	0.4655	NaN	NaN	0.4655	0	Q9CQP0	39S ribosomal protein L33, mitochondrial	Mrpl33	mmu:66845				
4629	NaN	0.2661	0.6650	0.4656	0	P32507	Nectin-2	Pvrl2	mmu:19294				
4630	NaN	NaN	NaN	0.4658	0	Q6PFF0		Scaf4	mmu:224432				
4631	NaN	0.0387	0.8938	0.4663	0	Q99JP4	Anaphase-promoting complex subunit CDC26	Cdc26	mmu:66440			1	
4632	0.4700	NaN	NaN	0.4700	0	Q8C263	Spindle and kinetochore-associated protein 3	Ska3	mmu:219114				
4633	0.4707	NaN	NaN	0.4707	0	F8VPQ2		Arid4a	mmu:238247				
4634	0.4725	NaN	NaN	0.4725	0	A0A0G2JH12							
4635	NaN	0.4782	NaN	0.4782	0	Q8N7N6		Traf3ip2	mmu:103213				
4636	0.4003	0.4540	NaN	0.4823	0	Q9D855	Cytochrome b-c1 complex subunit 7	Uqcrb					
4637	-0.1432	NaN	1.1172	0.4870	0	Q8R5K2	Ubiquitin carboxyl-terminal hydrolase 33	Usp33	mmu:170822				
4638	NaN	0.6149	0.3614	0.4882	0	Q99PQ2	E3 ubiquitin-protein ligase TRIM11	Trim11	mmu:94091			1	
4639	NaN	0.6005	0.6855	0.4891	0	Q99JW4	LIM and senescent cell antigen-like-containing domain protein 1	Lims1	mmu:110829				
4640	-1.0677	2.0579	NaN	0.4951	0	Q8BJ03	Cytochrome c oxidase assembly protein COX15 homolog	Cox15	mmu:226139				
4641	-0.2754	NaN	1.2731	0.4989	0	Q05BC3	Echinoderm microtubule-associated protein-like 1	Eml1	mmu:68519				
4642	0.4994	NaN	NaN	0.4994	0	P59113	Fermitin family homolog 1	Fermt1	mmu:241639				
4643	NaN	0.7609	0.2380	0.4995	0	Q9D6V8	Polyadenylate-binding protein-interacting protein 2	Paip2	mmu:67869				
4644	NaN	NaN	NaN	0.5020	0	Q6PGG6	Guanine nucleotide-binding protein-like 3-like protein	Gnl3l	mmu:237107				
4645	NaN	NaN	0.5033	0.5033	0	Q8CIA9	Hippocampus abundant transcript-like protein 1	Hiatl1	mmu:66631				
4646	-0.0964	NaN	1.8879	0.5040	0	Q80U58	Pumilio homolog 2	Pum2	mmu:80913				
4647	0.5045	NaN	NaN	0.5045	0	Q5RJH6	Protein SMG7	Smg7	mmu:226517				
4648	-0.2912	NaN	1.6351	0.5073	0	Q61037	Tuberin	Tsc2					
4649	0.4666	NaN	1.0246	0.5085	0	P70288	Histone deacetylase 2	Hdac2	mmu:15182				
4650	0.5209	NaN	NaN	0.5209	0	O70230	Zinc finger protein 143	Znf143	mmu:20841				
4651	0.4041	NaN	0.4209	0.5234	0	Q8R349	Cell division cycle protein 16 homolog	Cdc16	mmu:69957			1	

4652	0.5236	NaN	NaN	0.5236	0	Q8C569	Protein FAM118B	Fam118b	mmu:109229				
4653	NaN	NaN	0.5277	0.5277	0	Q5SSI6	U3 small nucleolar RNA-associated protein 18 homolog	Utp18	mmu:217109				
4654	NaN	1.1859	-0.1269	0.5295	0	Q9DA69	Intraflagellar transport protein 43 homolog	Ift43	mmu:76411				
4655	NaN	0.3633	0.6960	0.5297	0	P98195	Phospholipid-transporting ATPase;Probable phospholipid-transporting ATPase IIB	Atp9b	mmu:50771				
4656	NaN	0.9293	0.1410	0.5352	0	P53612	Geranylgeranyl transferase type-2 subunit beta	Rabggtb	mmu:19352				
4657	0.7267	NaN	NaN	0.5366	0	O88379	Bromodomain adjacent to zinc finger domain protein 1A	Baz1a					
4658	NaN	0.4758	1.1495	0.5395	0	Q9EPK2	Protein XRP2	Rp2	mmu:19889				
4659	NaN	1.6619	-0.7528	0.5413	0	Q921Z5	Tumor necrosis factor alpha-induced protein 8	Tnfaip8	mmu:106869				1
4660	0.4722	NaN	0.6120	0.5421	0	Q62440	Transducin-like enhancer protein 1	Tle1	mmu:21885				
4661	NaN	1.0037	0.0870	0.5453	0	Q8BH31	Major facilitator superfamily domain-containing protein 8	Mfsd8	mmu:72175				
4662	0.5458	NaN	NaN	0.5458	0	Q99PI4	Phosphatidate phosphatase LPIN3	Lpin3	mmu:64899				
4663	1.0181	NaN	NaN	0.5470	0	Q8C0C0	Zinc fingers and homeoboxes protein 2	Zhx2	mmu:387609				
4664	0.8767	0.2184	NaN	0.5475	0	Q61818	Retinoic acid-induced protein 1	Rai1	mmu:19377				
4665	0.0096	NaN	0.9043	0.5483	0	Q61072	Disintegrin and metalloproteinase domain-containing protein 9	Adam9	mmu:11502	1			
4666	0.5485	NaN	NaN	0.5485	0	Q8VEK6	Inhibitor of growth protein;Inhibitor of growth protein 3	Ing3	mmu:71777				
4667	0.2645	NaN	2.3514	0.5526	0	Q69ZK6	Probable JmjC domain-containing histone demethylation protein 2C	Jmjd1c	mmu:108829				
4668	NaN	-0.0052	NaN	0.5531	0	Q8C145	Zinc transporter ZIP6	Slc39a6	mmu:106957				
4669	0.1470	0.9617	NaN	0.5544	0	Q9CWS1	E3 ubiquitin-protein ligase RNF135	Rnf135	mmu:71956			1	
4670	0.7323	1.3982	NaN	0.5550	0	P16675	Carboxypeptidase;Lysosomal protective protein;Lysosomal protective protein 32 kDa chain;Lysoso	Ctsa	mmu:19025				
4671	NaN	NaN	0.5579	0.5579	0	Q08093	Calponin-2;Calponin	Cnn2	mmu:12798				
4672	NaN	0.5590	NaN	0.5590	0	Q80Z60-3							
4673	0.7265	NaN	0.3947	0.5606	0	Q69ZB8	Zinc finger CCHC domain-containing protein 2	Zcchc2	mmu:227449				
4674	0.8229	NaN	NaN	0.5647	0	Q9JKB3	Y-box-binding protein 3	Ybx3	mmu:56449				
4675	0.5664	NaN	NaN	0.5664	0	Q52KR2	Leucine-rich repeats and immunoglobulin-like domains protein 2;Leucine-rich repeats and immun	Lrig2;Lrig3	mmu:269473				
4676	0.5704	NaN	NaN	0.5704	0	Q9CQS2	H/ACA ribonucleoprotein complex subunit 3	Nop10	mmu:66181				
4677	0.5746	NaN	NaN	0.5746	0	Q3TIV9		Ap1s2					
4678	1.9435	NaN	-0.7890	0.5772	0	Q9CY57	Chromatin target of PRMT1 protein	Chtop	mmu:66511				
4679	1.0598	NaN	NaN	0.5810	0	O88874	Cyclin-K	Ccnk					
4680	0.5813	NaN	NaN	0.5813	0	Q4VBF2	R3H domain-containing protein 4	R3hdm4	mmu:109284				
4681	0.5823	NaN	NaN	0.5823	0	O08736	Caspase;Caspase-12	Casp12	mmu:12364				
4682	0.5823	NaN	NaN	0.5823	0	Q8BVR6	RING finger and SPRY domain-containing protein 1	Rspry1	mmu:67610				
4683	NaN	0.5714	0.6002	0.5858	0	O55023	Inositol monophosphatase 1	Impa1			1		
4684	NaN	0.5873	NaN	0.5873	0	Q9CPW9	Methionine aminopeptidase 1D, mitochondrial	Metap1d	mmu:66559				
4685	1.2291	0.7910	NaN	0.5909	0	O88974	Histone-lysine N-methyltransferase SETDB1	Setdb1					
4686	-0.3895	NaN	NaN	0.5955	0	F7ACR9		Macf1					
4687	0.5976	NaN	NaN	0.5976	0	Q8CI71	Coiled-coil domain-containing protein 132	Ccdc132	mmu:73288				
4688	0.6005	NaN	NaN	0.6005	0	Q99P31	Hsp70-binding protein 1	Hspbp1	mmu:66245				
4689	0.6018	NaN	NaN	0.6018	0	O88845	A-kinase anchor protein 10, mitochondrial	Akap10	mmu:56697				
4690	NaN	NaN	NaN	0.6036	0	P49442	Inositol polyphosphate 1-phosphatase	Inpp1	mmu:16329		1		
4691	0.6044	NaN	NaN	0.6044	0	D3Z6G3		Mapre3					
4692	NaN	0.9489	0.2651	0.6070	0	Q7TMF3	NADH dehydrogenase [ubiquinone] 1 alpha subcomplex subunit 12	Ndufa12	mmu:66414	1			
4693	0.6081	NaN	NaN	0.6081	0	Q9CWW7	CXXC-type zinc finger protein 1	Cxxc1	mmu:74322				
4694	NaN	-0.8168	2.0361	0.6097	0	Q5SW75	Protein phosphatase Slingshot homolog 2	Ssh2	mmu:237860		1		
4695	0.6117	NaN	NaN	0.6117	0	B1ART1		Vps13d					
4696	0.4558	NaN	0.5481	0.6118	0	Q68ED3	Non-canonical poly(A) RNA polymerase PAPD5	Papd5	mmu:214627				
4697	NaN	-1.3912	2.0836	0.6149	0	Q8BHN0	Protein phosphatase 1L	Ppm1l	mmu:242083		1		
4698	NaN	1.0605	0.1738	0.6172	0	Q3UDW8	Heparan-alpha-glucosaminide N-acetyltransferase	Hgsnat	mmu:52120				
4699	NaN	0.6642	0.5711	0.6176	0	Q8VE62	Polyadenylate-binding protein-interacting protein 1	Paip1	mmu:218693				
4700	NaN	0.6201	NaN	0.6201	0	A6BLY7	Keratin, type I cytoskeletal 28	Krt28	mmu:70843				
4701	-0.3922	NaN	0.6405	0.6223	0	P56695	Wolframin	Wfs1	mmu:22393				
4702	0.6241	NaN	NaN	0.6241	0	Q6P3Y5	Zinc finger protein 280C	Znf280c	mmu:208968				
4703	1.3148	NaN	NaN	0.6257	0	O09053	Werner syndrome ATP-dependent helicase homolog	Wrn	mmu:22427	1			
4704	0.8441	NaN	0.4121	0.6281	0	Q62280	Protein SSXT	Ss18	mmu:268996				
4705	0.6354	NaN	NaN	0.6354	0	Q5SV77	Gametogenetin-binding protein 2	Ggnbp2	mmu:217039				
4706	NaN	NaN	NaN	0.6415	0	A0A0J9YU44							
4707	NaN	1.2396	0.0484	0.6440	0	P97300	Neuroplastin	Nptn	mmu:20320				
4708	NaN	0.0273	-0.1669	0.6485	0	Q61738	Integrin alpha-7;Integrin alpha-7 heavy chain;Integrin alpha-7 light chain	Itga7					
4709	NaN	-0.1090	0.6684	0.6517	0	Q91YT8	CSC1-like protein 1	Tmem63a	mmu:208795				
4710	0.6522	NaN	NaN	0.6522	0	Q3U3N6	AP-4 complex accessory subunit tepsin	Enthd2	mmu:78777				
4711	0.6522	NaN	NaN	0.6522	0	Q8R184	Protein FAM110A	Fam110a	mmu:73847				
4712	NaN	0.5755	0.7302	0.6528	0	Q80XU3	Nuclear ubiquitous casein and cyclin-dependent kinase substrate 1	Nucks1	mmu:98415				
4713	NaN	NaN	0.6555	0.6555	0	Q5NBX1	Protein cordon-bleu	Cobl	mmu:12808				
4714	0.6587	NaN	NaN	0.6587	0	Q8VDY9	Caspase activity and apoptosis inhibitor 1	Caap1	mmu:67770				
4715	NaN	-0.0355	0.8009	0.6668	0	Q9D0K2	Succinyl-CoA:3-ketoacid coenzyme A transferase 1, mitochondrial	Oxct1	mmu:67041				
4716	0.6707	NaN	NaN	0.6707	0	Q80WG5	Volume-regulated anion channel subunit LRRC8A	Lrrc8a	mmu:241296				
4717	NaN	0.9208	0.4265	0.6737	0	P32883	GTPase KRas;GTPase KRas, N-terminally processed	Kras	mmu:16653				
4718	0.6781	NaN	NaN	0.6781	0	Q3U2S4	OTU domain-containing protein 5	Otud5	mmu:54644				
4719	0.6800	NaN	NaN	0.6800	0	D6RI42		Zc3h7a					
4720	0.6805	NaN	NaN	0.6805	0	Q5SGK3	Aldehyde oxidase 2	Aox2	mmu:213043				
4721	0.9658	NaN	NaN	0.6835	0	Q9D2I5	LisH domain-containing protein ARMC9	Armc9	mmu:78795				
4722	NaN	1.1374	0.2414	0.6894	0	Q8JZK9	Hydroxymethylglutaryl-CoA synthase, cytoplasmic	Hmgcs1	mmu:208715				
4723	NaN	0.6901	NaN	0.6901	0	Q8CJ19	Protein-methionine sulfoxide oxidase MICAL3	Mical3	mmu:194401				
4724	NaN	NaN	0.6902	0.6902	0	P15066	Transcription factor jun-D	Jund	mmu:16478				
4725	0.3062	NaN	NaN	0.6903	0	Q8CJG0	Protein argonaute-2	Ago2	mmu:239528				
4726	NaN	0.7018	0.6870	0.6944	0	O88342	WD repeat-containing protein 1	Wdr1	mmu:22388				
4727	NaN	NaN	0.6968	0.6968	0	A2A6A1	G patch domain-containing protein 8	Gpatch8	mmu:237943				
4728	0.6979	NaN	NaN	0.6979	0	Q80ZM8	Cardiolipin synthase (CMP-forming)	Crls1	mmu:66586				
4729	-0.0799	1.4776	NaN	0.6988	0	Q61188	Histone-lysine N-methyltransferase EZH2	Ezh2	mmu:14056	1			
4730	NaN	0.4330	0.9913	0.7121	0	Q9QY76	Vesicle-associated membrane protein-associated protein B	Vapb					
4731	2.2670	-0.4287	NaN	0.7133	0	Q9CWY4	Gem-associated protein 7	Gemin7	mmu:69731				
4732	NaN	NaN	0.7209	0.7209	0	Q91XB7	Protein YIF1A	Yif1a	mmu:68090				
4733	0.1083	NaN	1.3423	0.7253	0	A2A9C3	Protein SZT2	Szt2	mmu:230676				
4734	0.7296	0.7212	NaN	0.7254	0	Q9D6K5	Synaptojanin-2-binding protein	Synj2bp;Gr	mmu:24071				
4735	0.5621	NaN	1.2594	0.7338	0	Q62511	E3 ubiquitin-protein ligase ZFP91	Zfp91	mmu:109910			1	
4736	NaN	NaN	0.0348	0.7344	0	Q9Z2D1	Myotubularin-related protein 2	Mtmr2	mmu:77116		1		
4737	NaN	NaN	0.7495	0.7495	0	Q09199	Beta-1,4 N-acetylgalactosaminyltransferase 2	B4galnt2	mmu:14422				
4738	NaN	1.4652	0.0341	0.7497	0	Q91ZN5	Adenosine 3-phospho 5-phosphosulfate transporter 1	Slc35b2					
4739	-0.1003	NaN	NaN	0.7519	0	Q80YQ2	Mediator of RNA polymerase II transcription subunit 23	Med23	mmu:70208				
4740	NaN	0.6419	0.9083	0.7669	0	P97465	Docking protein 1	Dok1	mmu:13448				
4741	0.8863	NaN	0.3747	0.7675	0	E9Q784		Zc3h13	mmu:67302				
4742	NaN	NaN	NaN	0.7697	0	Q7TT18	Activating transcription factor 7-interacting protein 1	Atf7ip	mmu:54343				
4743	-0.1532	NaN	NaN	0.7714	0	Q8K072	Receptor expression-enhancing protein 4	Reep4	mmu:72549				
4744	0.7737	NaN	NaN	0.7737	0	Q9QXL2	Kinesin-like protein;Kinesin-like protein KIF21A;Kinesin-like protein KIF7;Kinesin-like protein KIF27	Kif21a;Kif7	mmu:16564				
4745	-0.1963	NaN	NaN	0.7744	0	Q99PM3	Transcription initiation factor IIA subunit 1;Transcription initiation factor IIA alpha chain;Transcrip	Gtf2a1	mmu:83602				
4746	NaN	-0.0601	1.6101	0.7750	0	Q9JMH6	Thioredoxin reductase 1, cytoplasmic	Txnrd1	mmu:50493	1			
4747	0.7810	NaN	NaN	0.7810	0	Q5U4C3	Splicing factor, arginine/serine-rich 19	Scaf1	mmu:233208				
4748	2.9717	NaN	1.3364	0.7812	0	Q64512	Tyrosine-protein phosphatase non-receptor type 13	Ptpn13	mmu:19249		1		
4749	0.7237	0.3761	NaN	0.7846	0	Q99JH1	Ribonuclease P protein subunit p25-like protein	Rpp25l	mmu:69961				
4750	NaN	NaN	0.7849	0.7849	0	B7ZMP1	Probable Xaa-Pro aminopeptidase 3	Xpnpep3	mmu:321003				
4751	1.1162	0.4579	NaN	0.7870	0	Q5IRJ6	Zinc transporter 9	Slc30a9	mmu:109108				
4752	0.7884	NaN	NaN	0.7884	0	Q8BPZ8	BRCA1-A complex subunit Abraxas	Fam175a	mmu:70681				
4753	0.7892	NaN	NaN	0.7892	0	Q9JLR9	HIG1 domain family member 1A, mitochondrial	Higd1a	mmu:56295				
4754	NaN	NaN	-0.8860	0.7912	0	Q8R404	Protein QIL1	Qil1	mmu:224904				
4755	1.2894	NaN	NaN	0.7937	0	Q925J9	Mediator of RNA polymerase II transcription subunit 1	Med1	mmu:19014				
4756	0.8001	NaN	NaN	0.8001	0	Q924T7	E3 ubiquitin-protein ligase RNF31	Rnf31	mmu:268749			1	
4757	0.5110	NaN	NaN	0.8024	0	O08856	RNA polymerase II elongation factor ELL	Ell	mmu:13716				
4758	NaN	1.3314	0.2789	0.8051	0	Q9R1S3	GPI ethanolamine phosphate transferase 1	Pign					
4759	0.8118	NaN	NaN	0.8118	0	Q9DCM7	Nucleus accumbens-associated protein 2	Nacc2	mmu:67991				
4760	0.8120	NaN	NaN	0.8120	0	Q8K1N1	Calcium-independent phospholipase A2-gamma	Pnpla8	mmu:67452				
4761	0.5084	NaN	0.2214	0.8149	0	Q61712	DnaJ homolog subfamily C member 1	Dnajc1	mmu:13418				
4762	1.4873	NaN	NaN	0.8204	0	Q8K389	CDK5 regulatory subunit-associated protein 2	Cdk5rap2	mmu:214444				
4763	0.8236	NaN	NaN	0.8236	0	Q9D4H9	PHD finger protein 14	Phf14	mmu:75725				
4764	1.7189	NaN	NaN	0.8285	0	O88327	Alpha-catulin	Ctnnal1	mmu:54366				
4765	NaN	NaN	NaN	0.8333	0	Q99N84	28S ribosomal protein S18b, mitochondrial	Mrps18b	mmu:66973				
4766	0.8342	NaN	NaN	0.8342	0	Q80U87	Ubiquitin carboxyl-terminal hydrolase;Ubiquitin carboxyl-terminal hydrolase 8	Usp8	mmu:84092				
4767	0.8376	NaN	NaN	0.8376	0	Q8BXQ8	Protein FAM53C	Fam53c	mmu:66306				
4768	-0.9123	NaN	NaN	0.8378	0	Q9DBE8	Alpha-1,3/1,6-mannosyltransferase ALG2	Alg2	mmu:56737				
4769	0.8403	NaN	NaN	0.8403	0	Q9R1C0	Transcription initiation factor TFIID subunit 7	Taf7	mmu:24074				
4770	NaN	0.8411	NaN	0.8411	0	Q3TFQ1	SPRY domain-containing protein 7	Spryd7	mmu:66674				
4771	-0.5266	NaN	NaN	0.8446	0	O88700	Bloom syndrome protein homolog	Blm	mmu:12144				
4772	0.8487	NaN	NaN	0.8487	0	Q9JME5	AP-3 complex subunit beta-2	Ap3b2	mmu:11775				
4773	0.8526	0.8467	NaN	0.8497	0	P20826	Kit ligand;Soluble KIT ligand	Kitlg	mmu:17311				
4774	0.8557	NaN	NaN	0.8557	0	Q9D4C5	ELL-associated factor 1	Eaf1	mmu:74427				
4775	NaN	0.2629	NaN	0.8566	0	P13011	Acyl-CoA desaturase 2	Scd2	mmu:20250				
4776	2.1836	NaN	-0.3382	0.8573	0	F7CVJ5		Ahnak2					
4777	1.3806	NaN	0.3382	0.8594	0	B2RR83	Probable ATP-dependent RNA helicase YTHDC2	Ythdc2	mmu:240255				

4778	NaN	0.7649	0.9558	0.8603	0	Q8BFQ8	Parkinson disease 7 domain-containing protein 1	Pddc1	mmu:213350			
4779	NaN	2.4744	0.5845	0.8604	0	Q9ESP1	Stromal cell-derived factor 2-like protein 1	Sdf2l1	mmu:64136			
4780	NaN	1.3280	NaN	0.8629	0	Q3UBZ5	MIF4G domain-containing protein	Mif4gd	mmu:69674			
4781	NaN	NaN	0.8649	0.8649	0	Q9WU84	Copper chaperone for superoxide dismutase	Ccs	mmu:12460	1		
4782	0.8720	NaN	NaN	0.8720	0	Q505D1	Serine/threonine-protein phosphatase 6 regulatory ankyrin repeat subunit A	Ankrd28	mmu:105522			
4783	0.8729	NaN	NaN	0.8729	0	Q8VEJ4	Notchless protein homolog 1	Nle1	mmu:217011			
4784	0.8763	NaN	NaN	0.8763	0	Q01705	Neurogenic locus notch homolog protein 1;Notch 1 extracellular truncation;Notch 1 intracellular c	Notch1	mmu:18128			
4785	NaN	NaN	0.8782	0.8782	0	Q8BIZ6	Smad nuclear-interacting protein 1	Snip1	mmu:76793			
4786	NaN	1.2161	0.6652	0.8880	0	Q6PAM0	5-AMP-activated protein kinase subunit beta-2	Prkab2	mmu:108097			
4787	NaN	NaN	0.7327	0.8932	0	Q9D1G2	Phosphomevalonate kinase	Pmvk	mmu:68603			
4788	NaN	NaN	0.8970	0.8970	0	Q8BJM7	S-adenosyl-L-methionine-dependent tRNA 4-demethylwyosine synthase	Tyw1	mmu:100929			
4789	NaN	0.9771	0.8198	0.8984	0	Q3USJ8	F-BAR and double SH3 domains protein 2	Fchsd2	mmu:207278			
4790	NaN	2.1544	-0.3436	0.9054	0	Q9DAS9	Guanine nucleotide-binding protein G(I)/G(S)/G(O) subunit gamma-12	Gng12	mmu:14701			
4791	0.5847	-0.7875	NaN	0.9054	0	Q9D2U5	N-alpha-acetyltransferase 38, NatC auxiliary subunit	Naa38	mmu:78304			
4792	0.9146	NaN	NaN	0.9146	0	Q7TNB8	Protein strawberry notch homolog 2	Sbno2	mmu:216161			
4793	NaN	NaN	0.9177	0.9177	0	Q8CGB3	Uveal autoantigen with coiled-coil domains and ankyrin repeats	Uaca	mmu:72565	1		
4794	0.9757	NaN	NaN	0.9191	0	Q8BHB4	WD repeat-containing protein 3	Wdr3	mmu:269470			
4795	NaN	NaN	NaN	0.9226	0	Q60715	Prolyl 4-hydroxylase subunit alpha-1	P4ha1	mmu:18451			
4796	1.5870	0.2629	NaN	0.9250	0	Q8C2B3	Histone deacetylase 7	Hdac7	mmu:56233			
4797	0.1620	0.4267	NaN	0.9253	0	Q9D1H6	NADH dehydrogenase [ubiquinone] 1 alpha subcomplex assembly factor 4	Ndufaf4	mmu:68493			
4798	0.2244	2.4844	NaN	0.9259	0	G3X9A3		Zbtb21				
4799	NaN	1.1507	0.7262	0.9385	0	Q9Z2W0	Aspartyl aminopeptidase	Dnpep	mmu:13437			
4800	-0.4011	NaN	-0.2963	0.9521	0	Q9CR67	Transmembrane protein 33	Tmem33	mmu:67878			
4801	NaN	0.5024	1.4065	0.9545	0	P45376	Aldose reductase	Akr1b1;Akı	mmu:11677	1		
4802	0.9569	NaN	NaN	0.9569	0	P62911	60S ribosomal protein L32	Rpl32	mmu:19951			
4803	NaN	2.8203	-0.9054	0.9575	0	E9Q941		Cdh18				
4804	0.1590	0.7655	NaN	0.9704	0	Q3UC65	Arginine/serine-rich protein 1	Rsrp1	mmu:27981			
4805	NaN	0.2488	NaN	0.9734	0	O88520	Leucine-rich repeat protein SHOC-2	Shoc2	mmu:56392			
4806	NaN	NaN	0.9739	0.9739	0	Q9DAY6		Yae1d1	mmu:67008			
4807	NaN	1.4881	0.4806	0.9843	0	Q8BKR5	Protein phosphatase 1 regulatory subunit 37	Ppp1r37	mmu:232947			
4808	0.8095	NaN	NaN	0.9863	0	Q80SY5	Pre-mRNA-splicing factor 38B	Prpf38b	mmu:66921			
4809	1.0026	NaN	NaN	1.0026	0	Q8C7V3	U3 small nucleolar RNA-associated protein 15 homolog	Utp15	mmu:105372			
4810	1.0038	NaN	NaN	1.0038	0	Q7TMK9	Heterogeneous nuclear ribonucleoprotein Q	Syncrip	mmu:56403			
4811	0.7528	NaN	NaN	1.0051	0	Q8BTZ5	Ankyrin repeat domain-containing protein 46	Ankrd46	mmu:68839			
4812	0.3209	1.7110	NaN	1.0160	0	Q8VCR7	Alpha/beta hydrolase domain-containing protein 14B	Abhd14b				
4813	NaN	NaN	1.0241	1.0241	0	Q9CR64	Protein kish;Protein kish-A	Tmem167;	mmu:66074			
4814	NaN	NaN	1.0280	1.0280	0	Q505B7	Protein archease	Zbtb8os	mmu:67106			
4815	-0.2058	0.0919	NaN	1.0284	0	Q9DB43	Zinc finger protein-like 1	Zfpl1	mmu:81909			
4816	NaN	1.7392	0.3314	1.0353	0	Q9DB42	Zinc finger protein 593	Znf593	mmu:68040			
4817	1.0382	NaN	NaN	1.0382	0	Q9CQJ6	Density-regulated protein	Denr	mmu:68184			
4818	NaN	0.1973	NaN	1.0400	0	Q9D1Q4	Dolichol-phosphate mannosyltransferase subunit 3	Dpm3	mmu:68563			
4819	NaN	NaN	1.7595	1.0439	0	Q9BCZ4	Selenoprotein S	Vimp	mmu:109815	1		
4820	NaN	3.4998	0.3929	1.0605	0	Q8K4J6	MKL/myocardin-like protein 1	Mkl1	mmu:223701			1
4821	NaN	0.2391	NaN	1.0756	0	P61600	N-alpha-acetyltransferase 20	Naa20	mmu:67877			
4822	NaN	NaN	NaN	1.0798	0	Q9R0G7	Zinc finger E-box-binding homeobox 2	Zeb2	mmu:24136			
4823	-0.0136	NaN	2.1826	1.0845	0	Q99KG3	RNA-binding protein 10	Rbm10	mmu:236732			
4824	NaN	1.0855	NaN	1.0855	0	P28571	Transporter;Sodium- and chloride-dependent glycine transporter 1	Slc6a9	mmu:14664			
4825	NaN	NaN	NaN	1.0930	0	Q9D7E3	Ovarian cancer-associated gene 2 protein homolog	Ovca2	mmu:246257			
4826	0.0398	NaN	NaN	1.1029	0	Q9CXV9	DCN1-like protein 5	Dcun1d5	mmu:76863			
4827	0.3636	NaN	NaN	1.1044	0	Q9DB91	Mediator of RNA polymerase II transcription subunit 29	Med29	mmu:67224			
4828	2.1550	0.0727	NaN	1.1139	0	Q9WV76	AP complex subunit beta;AP-4 complex subunit beta-1	Ap4b1	mmu:67489			
4829	1.2332	NaN	NaN	1.1143	0	P70255	Nuclear factor 1 C-type	Nfic	mmu:18029			
4830	1.1150	NaN	NaN	1.1150	0	Q9WV02	RNA-binding motif protein, X chromosome;RNA-binding motif protein, X chromosome, N-terminal	Rbmx	mmu:19655			
4831	1.1196	NaN	NaN	1.1196	0	Q9CXF7	Chromodomain-helicase-DNA-binding protein 1-like	Chd1l	mmu:68058			
4832	1.2542	NaN	1.0259	1.1376	0	Q9ERS2	NADH dehydrogenase [ubiquinone] 1 alpha subcomplex subunit 13	Ndufa13	mmu:67184			1
4833	1.1407	NaN	NaN	1.1407	0	Q9CWT6	Probable ATP-dependent RNA helicase DDX28	Ddx28	mmu:71986			
4834	NaN	2.0595	0.2788	1.1692	0	Q99K01	Pyridoxal-dependent decarboxylase domain-containing protein 1	Pdxdc1	mmu:94184			
4835	NaN	1.1830	NaN	1.1830	0	Q9ERD7	Tubulin beta-3 chain	Tubb3	mmu:22152			
4836	0.8709	NaN	NaN	1.1867	0	Q6ZWR6	Nesprin-1	Syne1	mmu:64009			
4837	NaN	-0.1407	1.0918	1.2031	0	B1AVZ0	Uracil phosphoribosyltransferase homolog	Uprt	mmu:331487			
4838	NaN	NaN	1.2230	1.2230	0	P83917	Chromobox protein homolog 1	Cbx1	mmu:12412			
4839	NaN	NaN	NaN	1.2507	0	Q8BGP6	Solute carrier family 25 member 40	Slc25a40	mmu:319653			
4840	1.2682	NaN	NaN	1.2682	0	Q8VFT5	Olfactory receptor	Olfr732	mmu:258659			
4841	0.6094	1.9833	NaN	1.2964	0	O35066	Kinesin-like protein KIF3C	Kif3c	mmu:16570			
4842	NaN	NaN	1.3016	1.3016	0	Q9CQ13	Coordinator of PRMT5 and differentiation stimulator	Coprs	mmu:66423			
4843	2.6728	NaN	-0.0431	1.3149	0	Q9JI58	Retinoic acid early-inducible protein 1-delta;Retinoic acid early-inducible protein 1-epsilon;Retinoi	Raet1d;Rae	mmu:56554			
4844	0.4307	NaN	NaN	1.3575	0	P59108	Copine-2	Cpne2	mmu:234577			
4845	1.3604	NaN	NaN	1.3604	0	Q8BPY9	Fidgetin-like protein 1	Fignl1	mmu:60530			
4846	1.2143	1.5450	NaN	1.3797	0	Q61458	Cyclin-H	Ccnh	mmu:66671			
4847	NaN	NaN	1.3907	1.3907	0	Q5DTW7	Uncharacterized protein KIAA1551	2810474O1	mmu:67246			
4848	0.7369	NaN	NaN	1.3955	0	Q8BHY2	Nucleolar complex protein 4 homolog	Noc4l	mmu:100608			
4849	NaN	NaN	NaN	1.4400	0	Q61510	E3 ubiquitin/ISG15 ligase TRIM25	Trim25	mmu:217069		1	
4850	NaN	NaN	NaN	1.4483	0	Q3UFS0	Protein zyg-11 homolog B	Zyg11b	mmu:414872			
4851	NaN	1.7201	1.2008	1.4605	0	P48771	Cytochrome c oxidase subunit 7A2, mitochondrial	Cox7a2	mmu:12866			
4852	1.2447	NaN	0.9006	1.4656	0	Q8K3W3	Protein CASC3	Casc3	mmu:192160			
4853	1.4716	NaN	NaN	1.4716	0	Q8BSL7	ADP-ribosylation factor 2	Arf2	mmu:11841			
4854	NaN	NaN	NaN	1.4738	0	Q9CR46	Spindle and kinetochore-associated protein 2	Ska2	mmu:66140			
4855	1.4752	NaN	NaN	1.4752	0	Q5FWH2	Putative E3 ubiquitin-protein ligase UNKL	Unkl	mmu:74154		1	
4856	NaN	NaN	1.4934	1.4934	0	Q33DR2	Decaprenyl-diphosphate synthase subunit 1	Pdss1	mmu:56075			
4857	NaN	1.7068	1.2886	1.4977	0	Q9D809		2200002D(	mmu:72275			
4858	1.5357	NaN	NaN	1.5357	0	Q9D1I2	Bcl10-interacting CARD protein		mmu:68480			
4859	0.7886	-0.2542	NaN	1.5465	0	Q80WW9	DDRGK domain-containing protein 1	Ddrgk1	mmu:77006			
4860	NaN	NaN	NaN	1.5708	0	Q8VDH1	F-box only protein 21	Fbxo21	mmu:231670			
4861	NaN	NaN	1.5827	1.5827	0	Q6ZPI0	Protein Jade-1	Jade1	mmu:269424			
4862	1.6086	NaN	NaN	1.6086	0	Q8K301	Probable ATP-dependent RNA helicase DDX52	Ddx52	mmu:78394			
4863	NaN	2.4519	0.8122	1.6321	0	Q3UL36	Arginine and glutamate-rich protein 1	Arglu1	mmu:234023			
4864	1.7341	NaN	NaN	1.7341	0	Q8K0U3	Transmembrane protein 17	Tmem17	mmu:103765			
4865	0.3114	NaN	NaN	1.8152	0	Q8BGS7	Choline/ethanolaminephosphotransferase 1	Cept1	mmu:99712			
4866	1.8255	NaN	NaN	1.8255	0	Q91VL9	Zinc finger and BTB domain-containing protein 1	Zbtb1	mmu:268564			
4867	NaN	2.2091	1.4919	1.8505	0	Q6TXD4	Dynamin-binding protein	Dnmbp	mmu:71972			
4868	NaN	2.6053	1.2664	1.9359	0	Q9ET54	Palladin	Palld	mmu:72333			
4869	0.0276	NaN	NaN	1.9455	0	Q8CHT3	Integrator complex subunit 5	Ints5	mmu:109077			
4870	1.9540	NaN	NaN	1.9540	0	Q9DBG9	Tax1-binding protein 3	Tax1bp3	mmu:76281			
4871	NaN	NaN	NaN	1.9753	0	O70494	Transcription factor Sp3	Sp3	mmu:20687			
4872	NaN	NaN	NaN	2.1243	0	Q9Z0G0	PDZ domain-containing protein GIPC1	Gipc1	mmu:67903			
4873	0.8699	NaN	NaN	2.1491	0	Q8BJQ2	Ubiquitin carboxyl-terminal hydrolase 1	Usp1	mmu:230484			
4874	-0.3133	NaN	NaN	2.1527	0	Q6KCD5	Nipped-B-like protein	Nipbl	mmu:71175			
4875	-1.2376	NaN	NaN	2.3459	0	Q9WTI7	Unconventional myosin-Ic	Myo1c	mmu:17913			
4876	NaN	NaN	NaN	2.5537	0	Q923Z0	G-protein coupled receptor family C group 5 member B	Gprc5b	mmu:64297			
4877	2.5567	NaN	NaN	2.5567	0	Q61602	Transcriptional activator GLI3;Transcriptional repressor GLI3R	Gli3	mmu:14634			
4878	2.6696	NaN	NaN	2.6696	0	P30561	Aryl hydrocarbon receptor	Ahr	mmu:11622			
4879	NaN	3.2038	2.7279	2.9659	0	Q8BWG8	Beta-arrestin-1	Arrb1	mmu:109689			
4880	3.0139	NaN	NaN	3.0139	0	Q8CGP2	Histone H2B;Histone H2B type 1-P;Histone H2B type 1-K;Histone H2B type 1-B;Histone H2B type 1-	Hist1h2br;	mmu:319188			
4881	3.0459	NaN	NaN	3.0459	0	Q9ESV0	ATP-dependent RNA helicase DDX24	Ddx24	mmu:27225			
4882	3.2871	NaN	NaN	3.2871	0	Q505D7	Optic atrophy 3 protein homolog	Opa3	mmu:403187			
4883	NaN	NaN	NaN	3.6632	0	Q9CY50	Translocon-associated protein subunit alpha	Ssr1	mmu:107513			
4884	4.1644	NaN	NaN	4.1644	0	Q5DTT3	Protein FAM208B	Fam208b	mmu:105203			
4885	NaN	NaN	NaN	4.3552	0	Q9JI18	Low-density lipoprotein receptor-related protein 1B	Lrp1b				
