# Supplementary material for: Anti-Cancer Potential of Homemade Fresh Garlic Extract Is Related to Increased Endoplasmic Reticulum Stress
Source: Nutrients. 2018 Apr 5;10(4):450. doi: 10.3390/nu10040450 (PMC5946235; doi:10.3390/nu10040450)
Supplement: Supplementary file 1 [file nutrients-10-00450-s001.zip › Supplementary materials Figre S1-7 and Table S2. .pdf]

Supplementary Information

Supplementary Figure S1

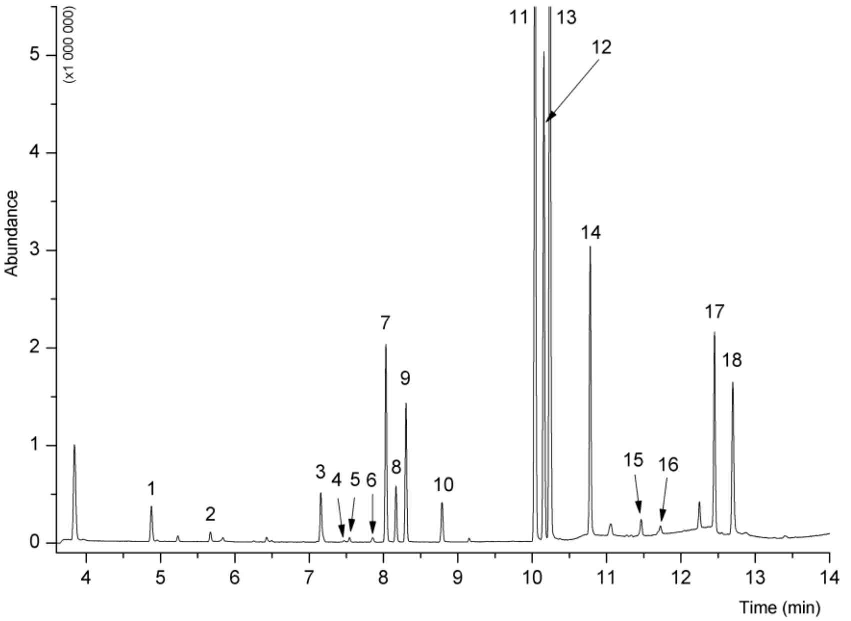

Peak no.	t_R [min]	Compound tentative identification
1	4.88	Allyl methyl sulfide
2	5.67	Dimethyl disulfide
3	7.15	Diallyl Sulfide
4	7.47	Isomer of Diallyl sulfide[1)]
5	7.54	Isomer of Diallyl sulfide[1)]
6	7.86	3,4-Dimethyl thiophene
7	8.03	Methyl-2-propenyl disulfide
8	8.17	Methyl-1-propenyl disulfide
9	8.30	1,3-Dithiane
10	8.80	Methyl isothiocyanate
11	10.04	Diallyl disulfide
12	10.15	Isomer of Diallyl disulfide[2)]
13	10.24	Isomer of Diallyl disulfide[2)]
14	10.8	Allyl methyl trisulfide
15	11.47	3-Vinyl-1,2-dithiacyclohex-4-ene
16	11.73	3-Vinyl-1,2-dithiacyclohex-5-ene
17	12.45	di-2-propenyl trisulfide
18	12.7	Isomer of di-2-propenyl trisulfide

Supplementary Figure S1: Headspace GC-MS chromatogram of unfractionated garlic extract (GE). The table shows tentative identification of the major peaks in the chromatogram by using EI mass spectra information and searching the NIST library. *Table notes: 1) probably 1,1'-thiobis-1-propene or Allyl(prop-1-en-1-yl)sulfane; 2) probably 1-propenyl allyl disulfide or dipropenyl disulfide. Compound identified are in accordance with precious reported data (*Shan, C., Wang, C.,Liu, J., Wu, P. (2013), The analysis of volatile flavor components of Jin Xiang garlic and Tai'an garlic. Agricultural Science, 4, 744-748.*)

Materials and methods related to Supplementary Figure S1: GE was diluted 1:1 with pure water (resistivity 18 MΩ/cm^2) to yield the final volume of 5 mL, and analyzed using a HT3 (Teledyne Tekmar, OH, USA) headspace autosampler unit coupled with a GC/MS system (Agilent, CA, USA). GC/MS system consisted of the 7890A Series Gas Chromatograph and the 7000 Series Triple Quad Mass Spectrometer. Helium was used as the GC carrier gas as well as the HT3 pressurization gas. The sample was thermostated at 70°C for 15 min. The valve oven and transfer line temperatures were 105°C and 110°C, respectively. The vial was pressurized for 2 min. to a pressure of 9 psig. The loop was filled for 1 min. to a pressure of 7 psig. The flow from the headspace unit was introduced into the GC using split mode (split ratio 5:1, inject time 0.50 min, inlet 220°C). An Agilent J&W DB-624 UI analytical column (30 m × 0.25 mm I.D., 1.4 µm) was operated at the following temperature program: 35°C for 1 min, 15°C/min to 220°C, 30°C/min to 240°C and 5 min final hold. The column pressure used was 15 psi and the flow rate of the carrier gas was 1.8 mL/min. The mass spectrometer operated in scan mode (35 – 350 m/z) and the collected data were evaluated using NIST 11 MS library for tentative identification of sulfur volatile and semi-volatile compounds.

Figures S2-6: Overlay of MIB-data on the pathway maps generated using KEGG database.

Supplementary Figure S2: ER-processing

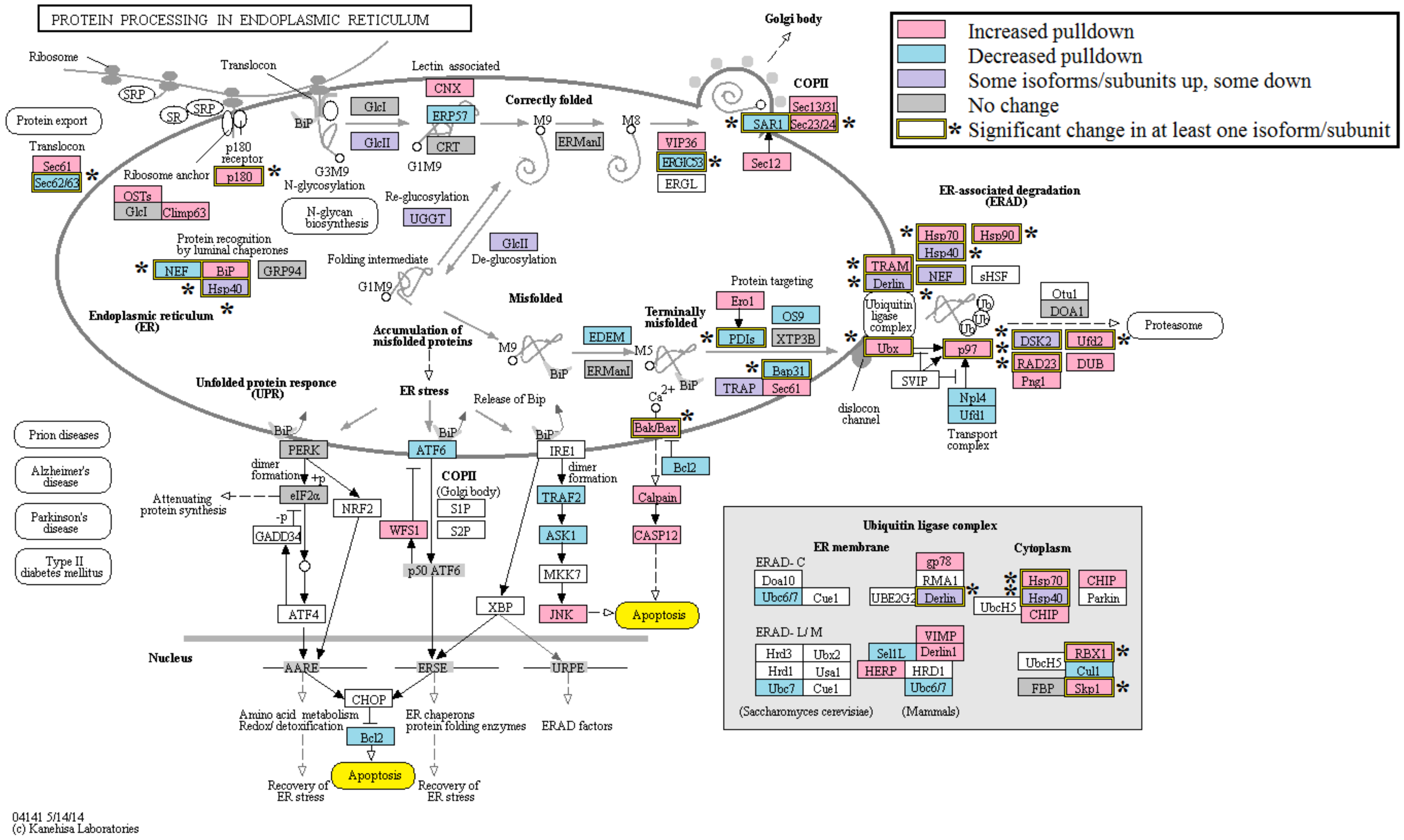

Figure S2 - Protein processing in endoplasmic reticulum (KEGG ID mmu:04141). We found an increase in proteins engaged in the processing of misfolded proteins and protein degradation, e.g. BiP, several heat shock proteins and proteasome associated proteins. We also see an upregulation of at least one proapoptotic pathway regulated by the ER.

Supplementary Figure S3: PI3K-Akt signaling

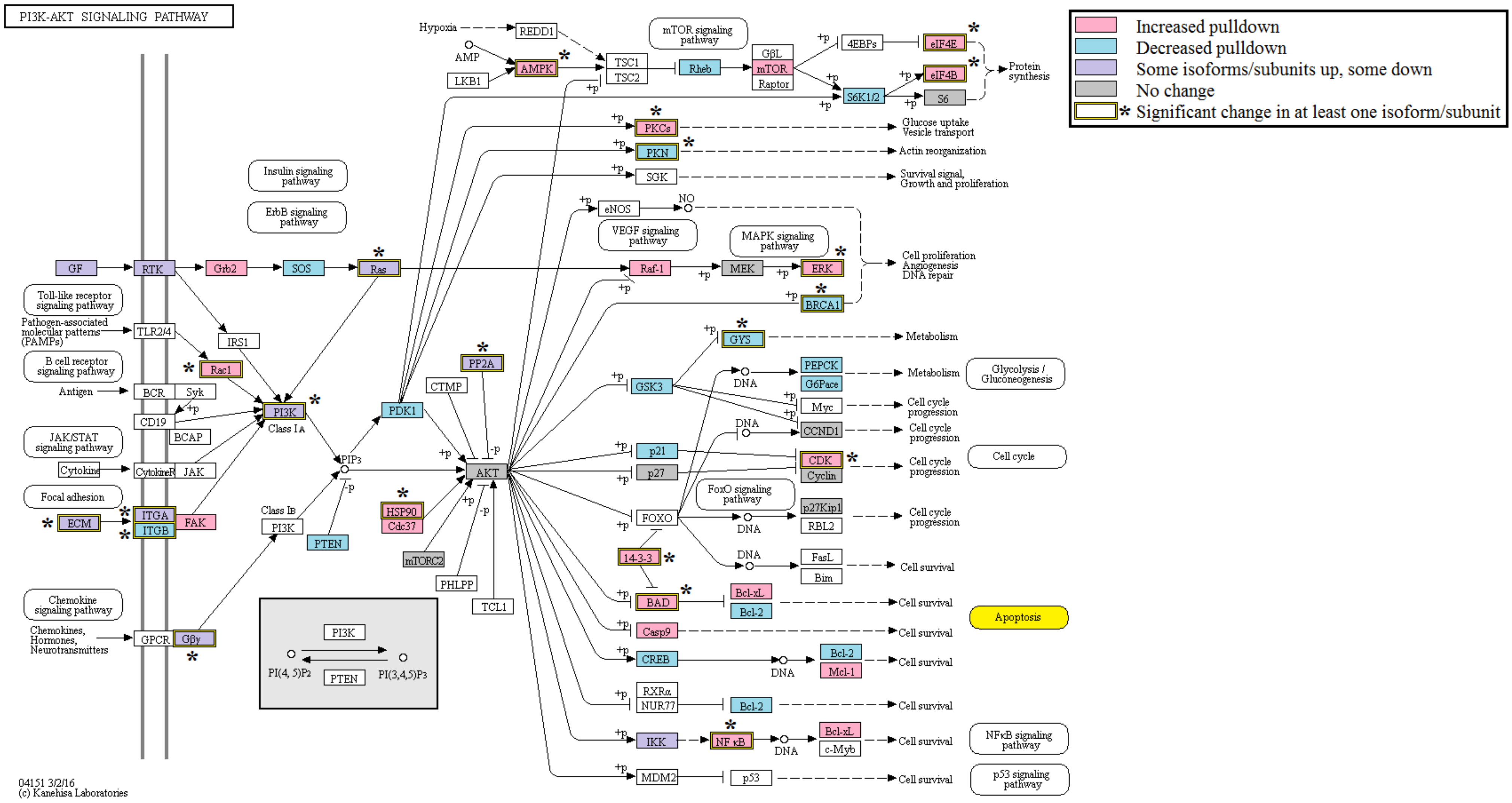

Figure S3 – PI3K-Akt signaling pathway (KEGG ID mmu:04151). Several upstream activators of AKT are significantly upregulated, however there is no significant change in AKT pulldown. Furthermore, the cellular response downstream of AKT is both up and down regulated.

Supplementary Figure S4: MAPK signaling

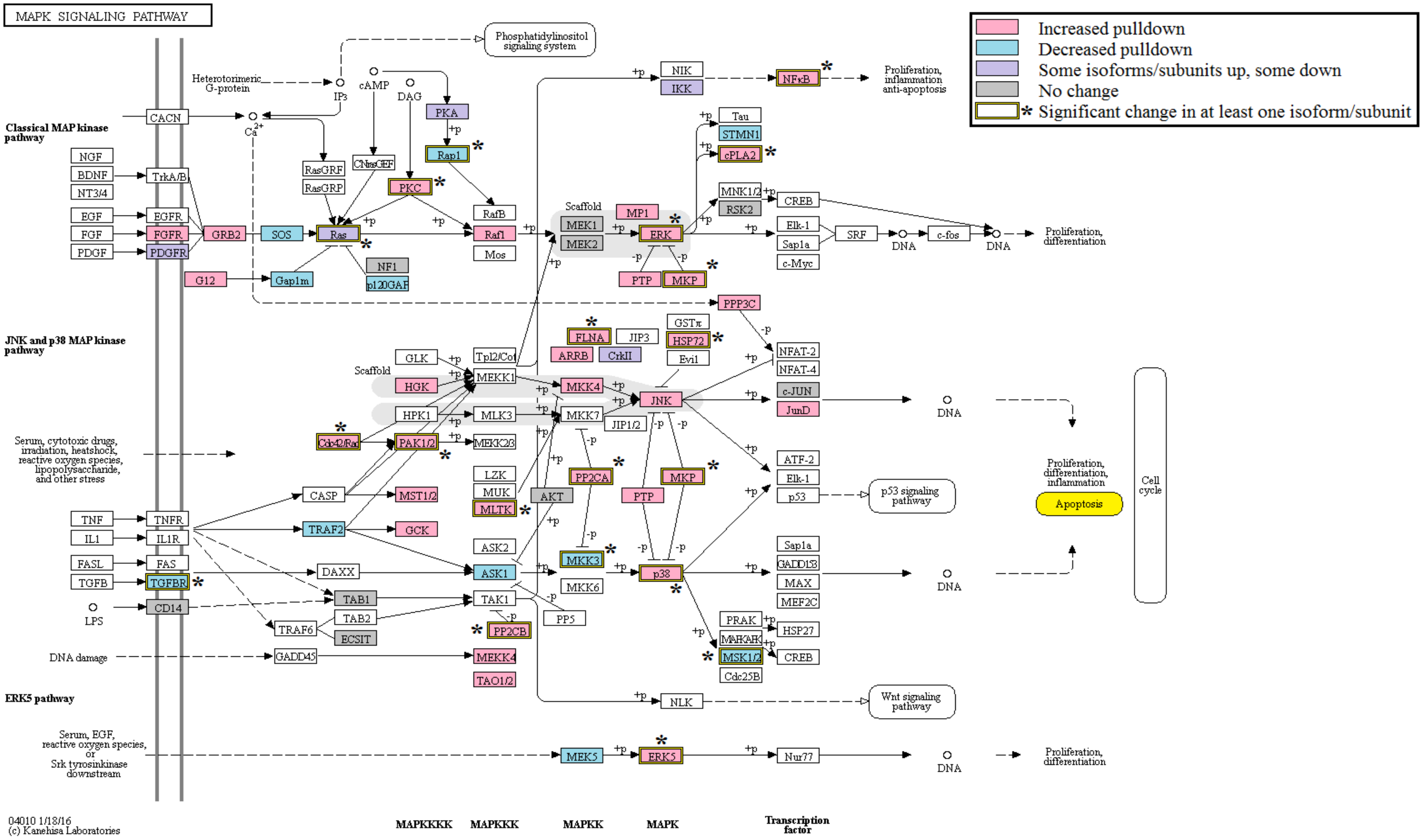

Figure S4 – MAPK pathway (KEGG ID mmu:04010). Several MAPKs are upregulated, not so much in the ERK sub-pathway as upstream of p38 and JNK. This activation does not seem to originate from the membrane bound receptors, but rather somewhere in the middle of the cascade, indicating activation by a cytosol sensing mechanism.

Supplementary Figure S5: Apoptosis

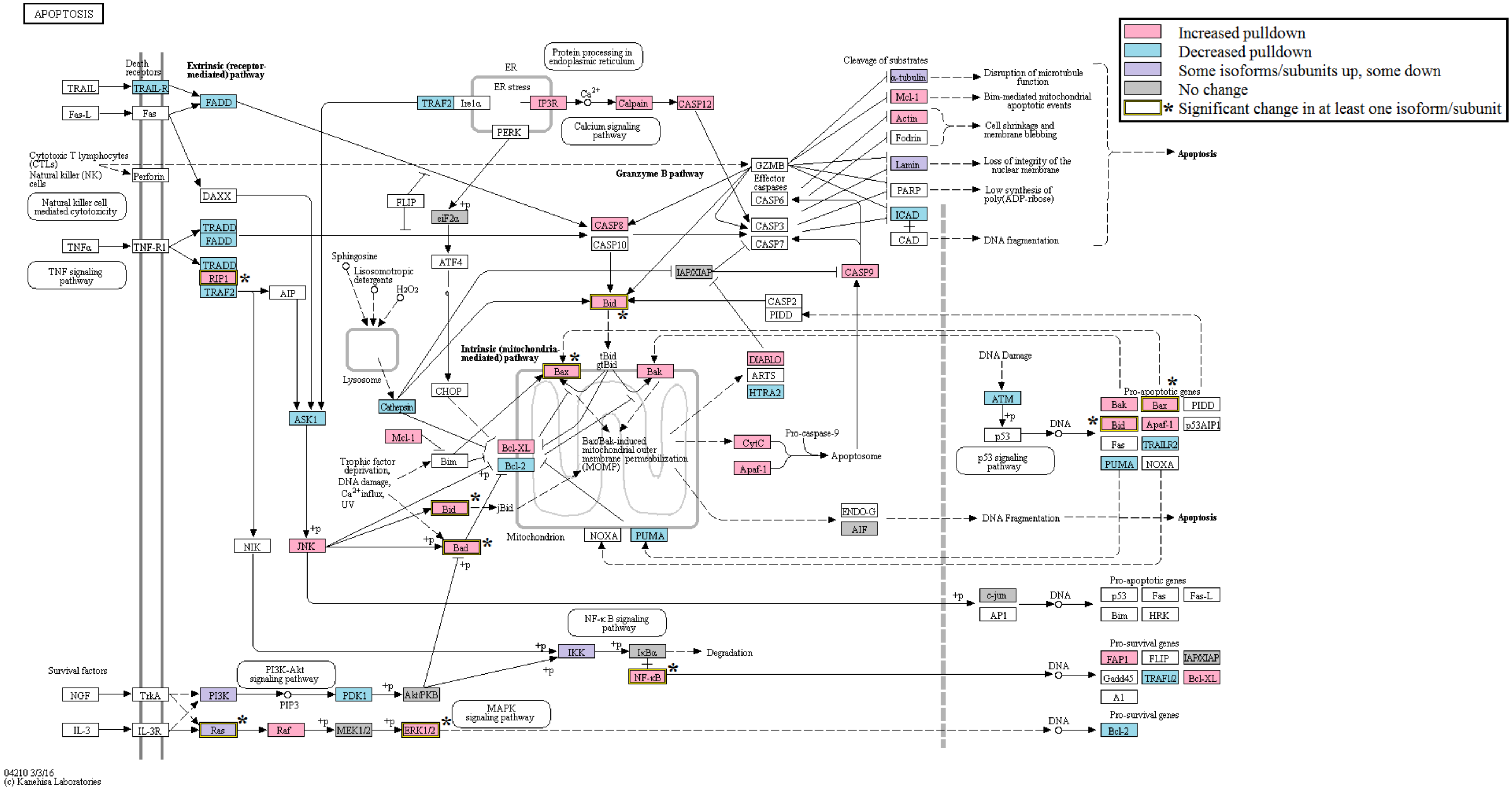

Figure S5 – Apoptosis pathways (KEGG ID mmu:04210). Two separate mechanisms leading to cell death are upregulated: i) the mitochondrial mechanism, relying on the increase of Bax/Bcl2 ratio, leading to the release of cytC and subsequent activation of caspase 9 and ii) the mitochondria independent activation of caspase 12 through ER stress induced signaling.

Supplementary Figure S6: WNT signaling

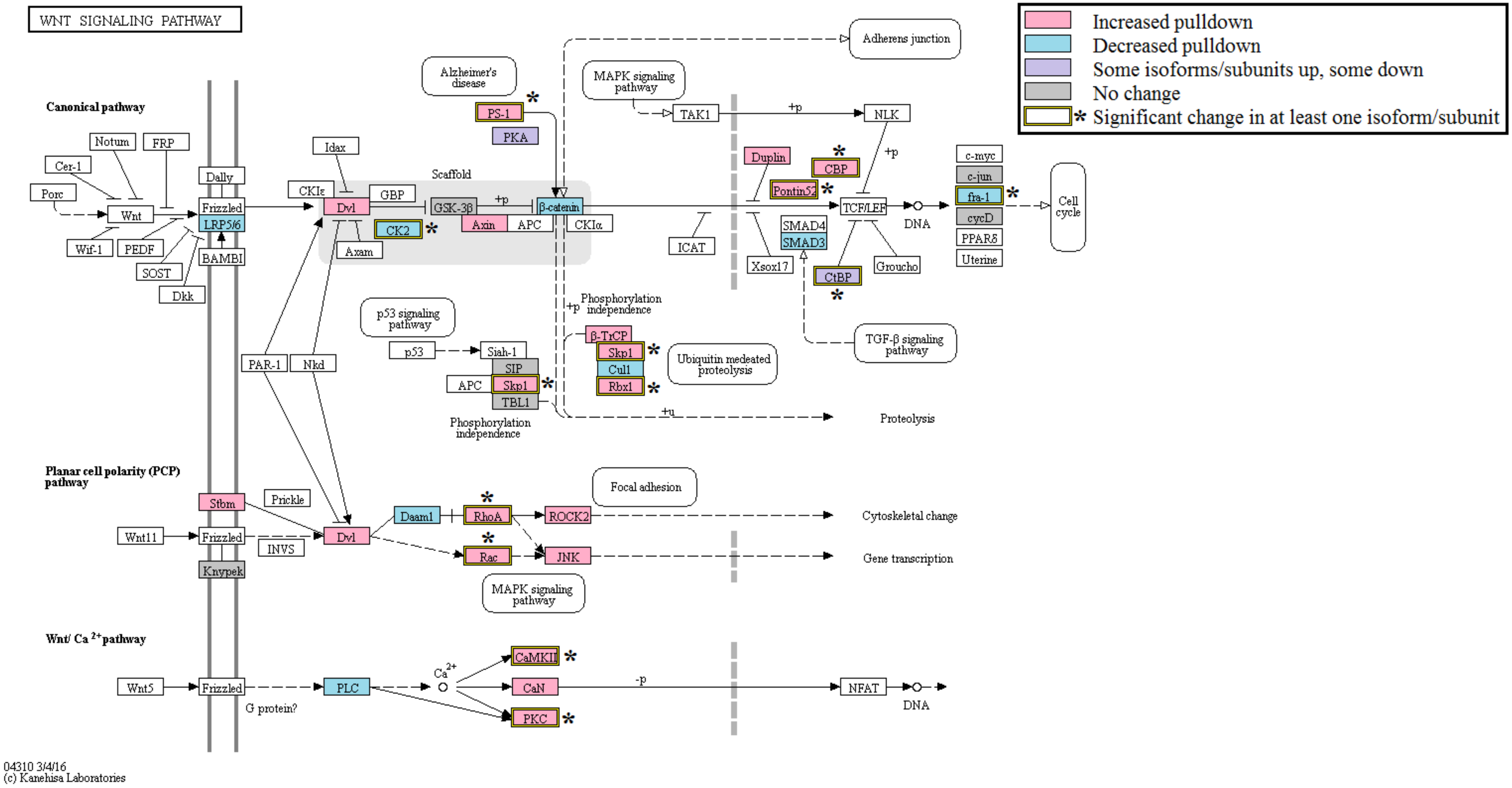

Figure S6 –WNT signaling pathway (KEGG ID mmu:04310). There are no significant changes in the main branch of the pathway, which is beta-catenin dependent, however, the signaling proteins downstream of Wnt11 and Wnt5 are strongly upregulated. The release of calcium from the ER is probably at least partially responsible for this activation.

Supplementary Figure S7: GSH

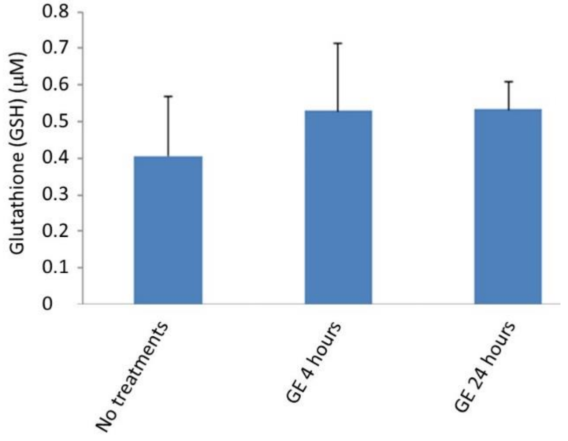

Figure S7: GE does not affect glutathione (GSH) levels in multiple myeloma cells (JJN3). JJN-3 seeded in cell culture flasks and treated with GE diluted 1:1500 for 4 and 24 hours. 7000 cells/well were seeded in 96-well plates and GSH levels were measured using the GSH-Glo^{TM} Glutathione Assay (Promega) according to the manufacturer's instructions.

Supplementary Table S2: Metabolite analysis

(A) Normalized m/z abundances

	NAD^+	NADH	$NADP^+$	NADPH	FAD^+
Control 1	314310	368207	263112	103525	25514
Control 2	256103	393135	230437	171517	22834
Control 3	224614	454309	213654	149258	25504
Control 4	218410	500044	215974	113123	25069
Average	253359	428924	230794	134355	24730
RSD (%)	17	14	10	24	5
GE 1	239131	379778	235724	107284	24949
GE 2	195020	475797	283761		20094
GE 3	244293	332462	243172	142226	25742
GE 4	219010	529801	188781		23233
Average	224363	429459	237859	124755	23504
RSD (%)	10	21	16	20	11
Log2 fold change	-0,2	0,0	0,0	-0,1	-0,1

(B) Average specific glucose consumption and lactate production

	Production/consumption (ng/cell/24h)	
	Glucose	Lactate
Control	0,67	1,65
GE	0,65	1,60

Table S2 – Metabolite analysis (A-B). (A) Normalized m/z abundances of intracellular NAD- and FAD-metabolites in 67NR cells 24 hours after addition of GE (1:1000). Normalized to cell density of separate dishes treated identically to sampled dishes. (B) Average specific glucose consumption and lactate production of 67NR cells in 24 hour interval following addition of GE (1:1000). Calculated using estimated average cell concentration in 24h interval (Control: 3,75E5 cells/ml, GE: 3.25E5 cells/ml), based on cell density of separate dishes treated identically to sampled dishes and reported doubling time of the cell line. (Simões RV, Serganova IS, Kruchevsky N, et al. Metabolic Plasticity of Metastatic Breast Cancer Cells: Adaptation to Changes in the Microenvironment. *Neoplasia (New York, NY)*. 2015;17(8):671-684. doi:10.1016/j.neo.2015.08.005.)

Materials and methods related to Supplementary Table S2 A-B, Metabolite analysis

67NR cells were grown to 60% confluency and treated with GE (1:1000). The cells were mechanically detached after 24 hours and pelleted by a 3-minute cold centrifugation at 913 rpm. The pellets were immediately transferred to liquid nitrogen. Pellets were thawed on ice prior to extraction of intracellular metabolites, performed in 300 μL pre-heated water-acetonitrile (80°C, 10-90%) by shaking at 1500 rpm in a 80°C heat block for 2 minutes. Soluble metabolites were separated from particulate by a cold centrifugation at 14000 rpm for two minutes. NAD^+-, NADH-, $NADP^+$-, NADPH- and FAD^+-pools were semi-quantified by LC-MS/MS. The analysis was performed on a Waters Acquity UPLC connected to a Waters TQ-S triple quadropol mass spectrometer operated in negative electrospray mode. 5 uL-samples were injected onto a Waters Acquity UPLC BEH Amide 1.7 □m 2.1x100 mm column, maintained at 40°C and eluted with mobile phases (A) water-acetonitrile (60-40%), and (B) water-acetonitrile (10-90%), both added 10 mM acetic acid and adjusted to pH 9.0 with a 25% ammonium hydroxide solution. The following gradient was applied with a flow rate of 0.4 mL/min: 0-0.5 min: 95% B, 0.5-2 min: 95-70% B, 2-6.5 min: 70-40 % B, 6.5-7 min: 40-1%B, 7-8 min: 1% B, 11 min: end. As reduced cofactors are unstable, standards (Sigma-Aldrich) were prepared fresh in mobile phase B on the day of analysis, and both standards and extracts were analyzed within four hours. The supernatants of the pelleted cells were lyophilized and up-concentrated five times in deuterium oxide (Sigma-Aldrich). 1D proton spectra were recorded at 25°C on a Bruker Ascend 400 MHz Avance III HD equipped with a 5 mm Z-gradient SmartProbe (Bruker). To quantify extracellular glucose, lactate and glutamine, the anomeric proton of α-glucose (5.2 ppm), methyl H^β of lactate (1.3 ppm) and methylene H^γ of glutamine (2.4 ppm) were integrated and quantified by ERETIC2 (Electronic Reference To access *In vivo* Concentrations, Topspin 3.5, Bruker). The methylamine H of a creatine (3.0 ppm) external standard (Sigma-Aldrich) was defined as eretic reference.
